# Electrochemical Synthesis of Isoxazolines: Method and Mechanism

**DOI:** 10.1002/chem.202103728

**Published:** 2022-02-10

**Authors:** Samuel D. L. Holman, Alfie G. Wills, Neal J. Fazakerley, Darren L. Poole, Diane M. Coe, Leonard A. Berlouis, Marc Reid

**Affiliations:** ^1^ WestCHEM Department of Pure and Applied Chemistry University of Strathclyde Royal College Building 204 George Street Glasgow G1 1XW UK; ^2^ GlaxoSmithKline Medicines Research Centre Gunnels Wood Road Stevenage SG1 2NY UK

**Keywords:** density functional calculations, electrochemistry, isoxazoline, mechanisms

## Abstract

An electrochemical method for the green and practical synthesis of a broad range of substituted isoxazoline cores is presented. Both aryl and more challenging alkyl aldoximes are converted to the desired isoxazoline in an electrochemically enabled regio‐ and diastereoselective reaction with electron‐deficient alkenes. Additionally, in‐situ reaction monitoring methods compatible with electrochemistry equipment have been developed in order to probe the reaction pathway. Supporting analyses from kinetic (time‐course) modelling and density functional theory support a stepwise, radical‐mediated mechanism, and discounts hypothesised involvement of closed shell [3+2] cycloaddition pathways.

## Graphical abstract

**Isoxazolines without the [3+2]**: An electrochemical method for the synthesis of isoxazoline cores is presented. Both aryl and more challenging alkyl aldoximes are converted to the desired isoxazoline through an electrochemically enabled regio‐ and diastereoselective reaction with electron‐deficient alkenes. Supporting analyses from kinetic (time‐course) modelling and density functional theory support a stepwise, radical‐mediated mechanism, and discount hypothesised involvement of closed‐shell [3+2] cycloaddition pathways.

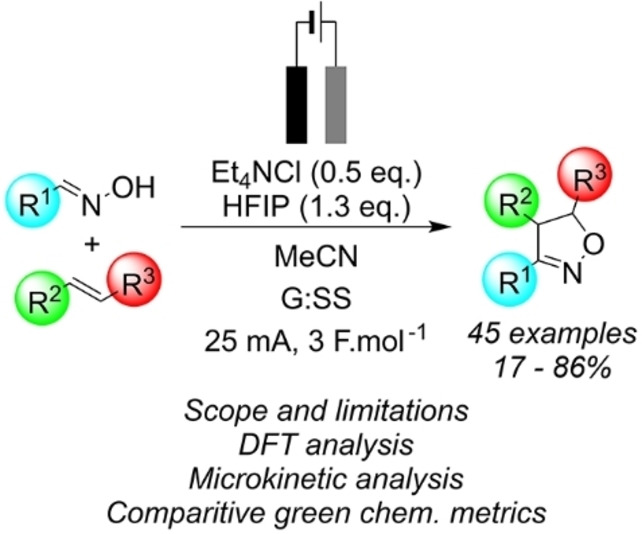

## Introduction

**Figure 1:**
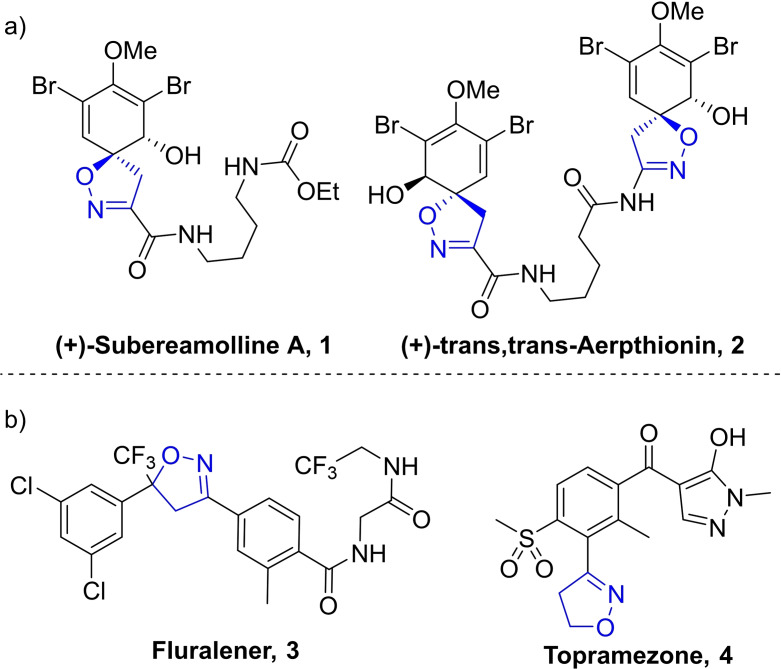
Examples of isoxazoline‐containing natural products and marketed drugs and pesticides.

Isoxazolines, their derivatives, and related N,O‐heterocycles are among the most important structural motifs in natural products.[^1^, ^2^] They possess many properties of value to the pharmaceutical industry, including antifungal and antibacterial activities.^[3]^ In particular, isoxazoline‐containing natural products have received significant interest for their potential to exhibit potent anticancer activity (**1** and **2**, Figure 1a).[^4^, ^5^, ^6^, ^7^]

**Scheme 1:**
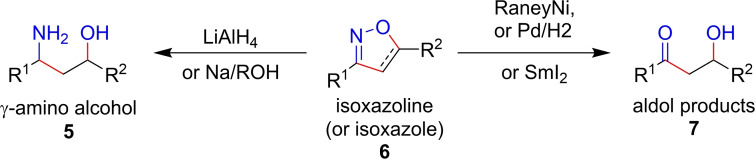
Isoxazolines and Isoxazoles as masked structural motifs.

Beyond natural products, isoxazolines are found to be important pharmacophores and can be seen in several marketed drugs, pesticides, and insecticides. Isoxazoline‐containing drugs include Fluralener (**3**), an oral insecticide and acaricide for use in canine flea removal, and Topramezone (**4**), a pesticide that is currently used and marketed in the UK (Figure 1b). However, it is not only their biological properties that make these heterocycles desirable to the chemistry community, but also their use as masked structural motifs (Scheme 1).[^8^, ^9^] Much attention has therefore been paid to the synthesis of substituted isoxazoles and isoxazolines.

**Scheme 2:**
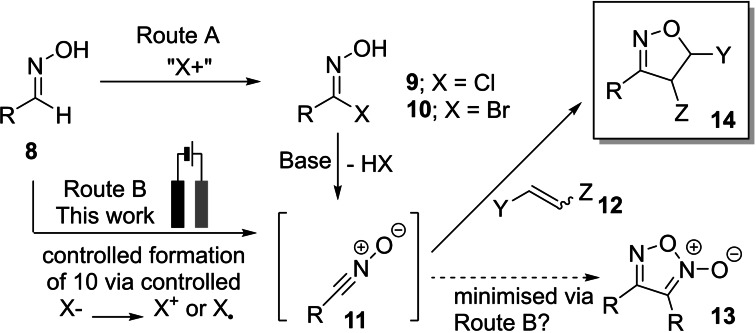
Rationale for the electrochemical route to in‐situ preparation of nitrile oxide intermediates towards isoxazolines.

Isoxazolines can be prepared from corresponding oximes by several methods, the most common of which is cyclisation of a nitrile oxide with a dipolarophile in a 1,3‐dipolar cycloaddition (1,3‐DC) reaction. Formation of nitrile oxides are most commonly achieved in two ways: halogenation of oximes using electrophilic sources of halogen^[10]^ and subsequent base‐promoted loss of HX (Scheme 2, route A); or dehydration of nitroalkanes.[^11^, ^12^] Electrophilic chlorination of oximes has been the most explored method of in‐situ preparation of hydroxyimoyl halides **9** and **10** (X=Cl and Br, respectively), which are precursors to nitrile oxides **11**.[^13^, ^14^, ^15^, ^16^] It is well known that nitrile oxides are highly reactive intermediates and can dimerise rapidly to form furoxan **13**,[^17^, ^18^] and alkyl nitrile oxides are most notorious for this undesired dimerisation event.[^19^, ^20^] We envisaged that the competing dimerisation reaction could be minimised by establishing a controlled electrochemical synthesis of isoxazolines wherein concentration of the intermediate nitrile oxide dipole **11** was kept strategically low versus the reaction partner **12** (Scheme 2, route B).

Electro‐organic synthesis has recently re‐emerged as a thriving field of synthetic chemistry due, in part, to the drive for reactions with improved environmental profiles.[^21^, ^22^, ^23^, ^24^, ^25^] Treating electrons as reagents and electrodes as reactants that are not consumed during the course of the reaction, electrochemistry holds promise in being able to comply with the 12 principles of green chemistry,^[26]^ as well as safety, environmental, legal, economic, control, and throughput (S.E.L.E.C.T.) criteria for process scale‐up towards manufacture.^[27]^ Electrochemistry can also circumvent the use of strong oxidising or reducing agents, avoiding toxic waste generation. Additionally, the use of electrons as “catalysts”^[28]^ in redox transformations can offer a powerful design strategy versus synthetic methods based on traditional catalysts/reagents.

Part of the barrier to broad adoption of electrochemical synthesis can be attributed to some extent to the lack standardised enabling technologies.^[21]^ Prior to IKA's release of the ElectraSyn 2.0,[^29^, ^30^] electrochemistry required bespoke glassware and equipment and, in terms of necessary entry‐level expertise, represented a barrier to broad uptake of the electrochemical cell as a powerful synthesis‐enabling tool. The electrochemical method development and mechanistic investigation of isooxazoline synthesis discussed in this work has been conducted primarily on the ElectraSyn 2.0 device. An initial version of this work was deposited in ChemRxiv on 22 December, 2020.^[31]^

## Results and Discussion

### Reaction optimisation

By revisiting previous work from Shono^[32]^ and Waldvogel,^[33]^ it was envisioned that the chlorination of oximes could be achieved through the electrochemical oxidation of chloride anions to an electrophilic chlorinating species. In‐situ generation of the nitrile oxide and 1,3‐DC with a dipolarophile partner could occur, furnishing the desired substituted isoxazolines. This hypothesis served as the basis for our method development and was only challenged during later mechanistic studies.

Benzaldehyde oxime **15** **a** and *tert*‐butyl acrylate **16** **a** were chosen as model substrates for the exploration of our electrochemically enabled 1,3‐DC reaction, with initial conditions of reticulated vitreous carbon (RVC) as both anode and cathode, NaCl as mediator, and a charge transfer of 4.5 F mol^−1^ at a current of 25 mA in MeOH (Table 1, entry 1). As shown in entry 1, the initial conditions provided isoxazoline **17** **a** in 31 % isolated yield. The methyl (rather than *tert*‐butyl) product of **17** **a** was also observed, with the assumption that the methanolic solvent is non‐innocent. Changing the electrode materials to a graphite (G) anode and stainless steel (SS) cathode, gave an increase in yield, but with a poorer ratio of desired/undesired products (entry 2 vs. entry 1). NaI as mediator in place of NaCl also gave an increase in yield, with a more favourable ratio of products (entry 3 vs. entry 2). The use of Et_4_NCl as mediator and electrolyte gave a reaction profile that evidenced negligible quantities of observable impurities, with only a small decrease in yield (entry 4). The improved solubility profile of a tetraalkylammonium salt as mediator allowed the exploration of other solvents, potentially eliminating the undesired transesterification of the target isoxazoline. To this end, reducing the charge transferred (entry 5) and switching the solvent to MeCN with 1.3 equiv of HFIP (entry 9) gave notably improved yields (63 and 78 %, respectively) and no observable side‐products (by LCMS or ^1^H NMR). HFIP has been shown to possess properties that can stabilise radical and reactive intermediates.^[34]^ Interestingly, the reaction proceeded, albeit less efficiently, with only 10 mol% of HFIP in MeCN (entry 10 vs. entry 9). Transferring more than 3 F mol^−1^ of charge (entry 11) and using TFE instead of HFIP (entry 12) furnished the desired isoxazoline in only slightly lower yields (73 and 74 % vs. 78 %). As was expected, in the presence of no electricity, no reaction was observed (entry 14). Importantly, no dimerisation or polymerisation products were detected in our NMR or LCMS analyses. Full optimisation tables and preparative details can be found in the experimental supporting information (Section 4). Mass balance beyond desired product included both unreacted starting materials (oxime and alkene), as well as unidentifiable polymeric side products.

**Table 1:** Optimisation of electrochemically enabled 1,3‐dipolar cycloaddition of aldoxime **15** **a** with alkene **16** **a**.^[a]^

	Electrode A : C	Mediator	Solvent	Additive (equiv)	F mol^−1^	Ratio^[b]^ **17 a** : **18**	Yield^[c]^ (**17 a**+**18**) [%]
1	RVC : RVC	NaCl	MeOH	–	4.5	–	31
2	G : SS	NaCl	MeOH	–	4.5	2 : 1	49
3	G : SS	NaI	MeOH	–	4.5	1.5 : 1	57
4	G : SS	Et_4_NCl	MeOH	–	4.5	1 : 1	48
5	G : SS	Et_4_NCl	MeOH	–	3	7 : 1	63
6	G : SS	Et_4_NCl	MeOH	–	5	6 : 1	39
7	G : SS	Et_4_NCl	MeCN	MeOH (34)	3	22 : 1	45
8	G : SS	Et_4_NCl	HFIP	–	3	–	36
9	G : SS	Et_4_NCl	MeCN	HFIP (1.3)	3	–	78
10	G : SS	Et_4_NCl	MeCN	HFIP (0.1)	3	–	49
11	G : SS	Et_4_NCl	MeCN	HFIP (1.3)	5	–	73
12	G : SS	Et_4_NCl	MeCN	TFE (1.3)	3	–	74
13	G : SS	Et_4_NCl	MeCN	IPA (1.3)	3	–	41
14	G : SS	Et_4_NCl	MeCN	HFIP (1.3)	0	–	0

[a] Conditions: **15** **a** (0.5 mmol), **16** **a** (5 equiv), mediator (0.5 equiv), additive (0.5 equiv), solvent (7 mL), anode/cathode, 25 mA, [CT] F mol^−1^. [b] Ratio determined from isolated yields of products. [c] Isolated yield. A=anode; C=cathode; RVC=reticulated vitreous carbon, G=graphite; SS=stainless steel; HFIP=1,1,1,3,3,3‐hexafluoroisopropanol; TFE=1,1,1‐trifluoroethanol; IPA=isopropanol.

### Aldoxime scope

**Scheme 3:**
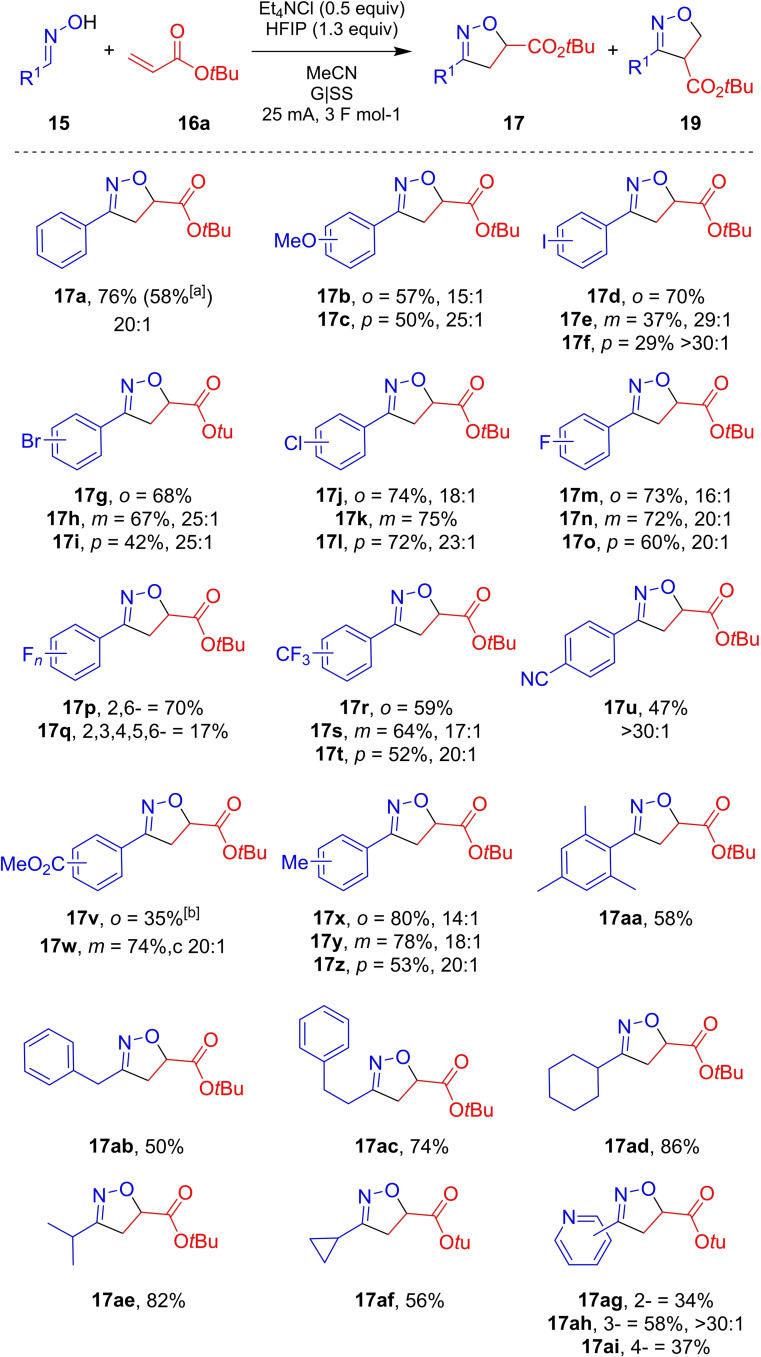
Scope of electrochemically enabled 1,3‐dipolar cycloaddition of aldoximes **15** and *tert*‐butyl acrylate **16** **aa**. Conditions: **15** (0.5 mmol), *tert*‐butyl acrylate **16** **a** (5 equiv), Et_4_NCl (0.5 equiv), HFIP (1.3 equiv), MeCN (7 mL), G anode, SS cathode, 25 mA, 3 F mol^−1^. Isolated yields. Regioisomeric ratios were determined by ^1^H NMR spectroscopy of chromatographed products. [b] 5 mmol scale (Experimental Supporting Information; Section 2); [c] 5 F mol^−1^ charge was transferred.

With optimised conditions in hand, attention was turned to a substrate scope in the oxime partner. Both (at least presumably) electron‐rich (forming products **17** **b**–**17** **c**, and **17** **x**–**17** **z**; Scheme 3) and electron‐poor (**17** **d**–**17** **w**) benzaldehyde oximes were well tolerated, with moderate to good yields achieved. Interestingly, the substitution on the phenyl ring had a marked effect on the yield of the reaction; in general, *meta*‐substitution gave the highest yield but with poorer observed regioselectivity. Furthermore, it was demonstrated that potentially electroactive groups such as I and Br were tolerated to a useful extent, with these moieties providing a chemical handle for further downstream chemistry (**17** **d**–**17** **f** and **17** **g**–**17** **i**, respectively). Mesityl oxime was smoothly converted to the corresponding isoxazoline **17** **aa** in 58 % yield. Alkyl oximes were well tolerated with **17** **ab**–**17** **af** isolated in good yields. The number of methylene units between the oxime functionality and the phenyl group has a large influence on the outcome of the reaction, with **17** **ab** (one methylene spacer) isolated in 50 % and **17** **ac** (two methylene spacers) in 74 % yield.

Of note, cyclopropyl‐substituted isoxazoline **17** **af** was isolated in a serviceable 56 % yield, suggesting that any radical intermediates generated under the reaction conditions can be used before intractable levels of competing side reactions that might otherwise ring open such strained functionality.^[35]^ Pyridyl aldoximes (**17** **ag**–**17** **ai**) were tolerated under the electrochemical reaction conditions, with no N‐oxide or Minisci‐type side products observed in the crude reaction mixture (by LCMS or ^1^H NMR). Encouragingly, the model reaction also worked on a 5 mmol scale, providing **17** **a** in an unoptimised 58 % isolated yield. We suspect the lower yield versus the smaller‐scale optimised process result from changes in reaction mixing profile and vessel geometry changes on scale‐up; all of which are being considered as part of longer‐term investigations. The ratios with which the current and concentration were scaled were influenced by the scale‐up procedure from Wang and co‐workers.^[36]^

### Dipolarophile scope

**Scheme 4:**
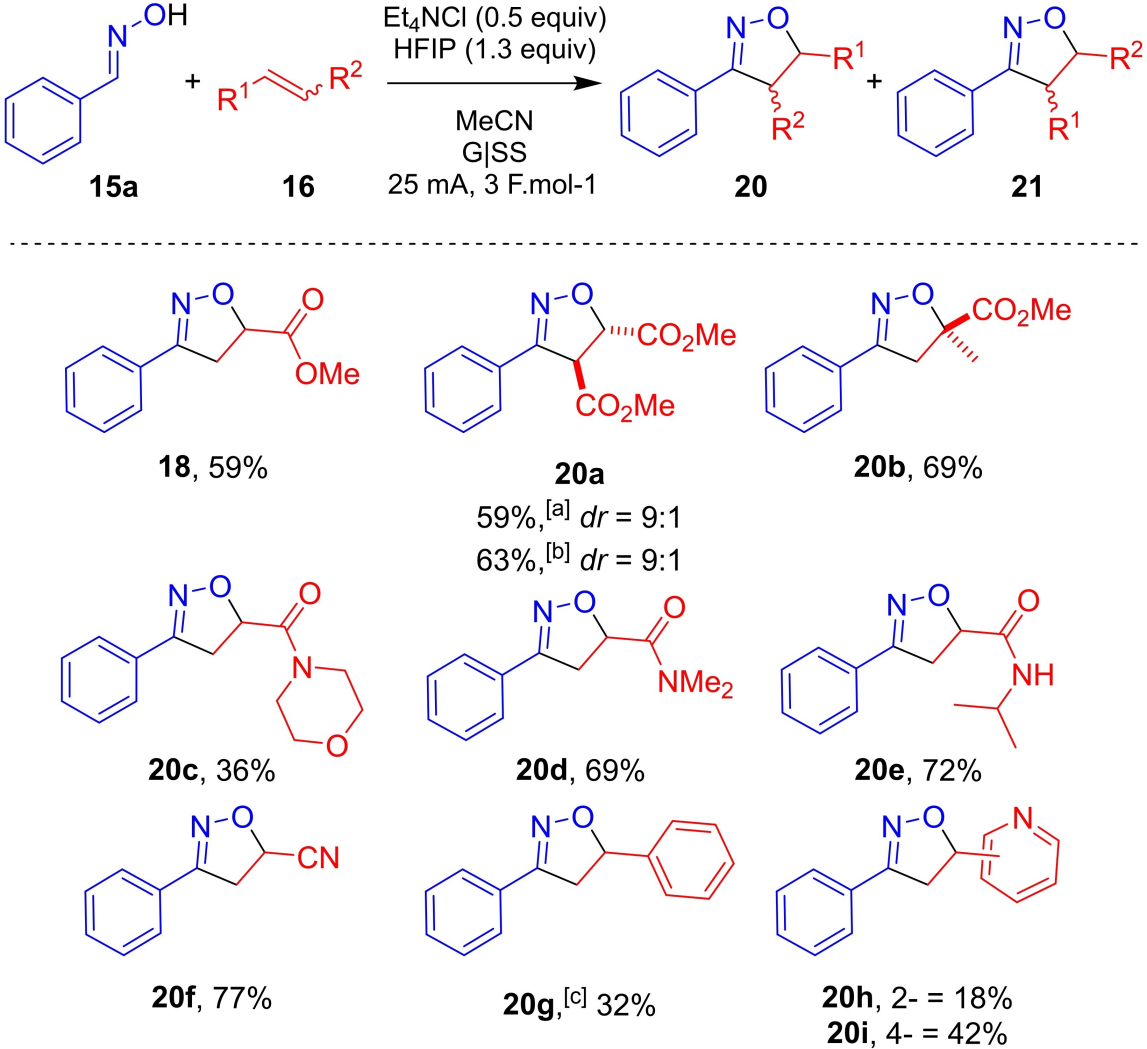
Substrate scope of electrochemically enabled 1,3‐dipolar cycloaddition of (*E*)‐benzaldehyde oxime **15** **a** with dipolarophiles **16** **a** Conditions: **15** **a** (0.5 mmol), **16** (5 equiv), Et_4_NCl (0.5 equiv), HFIP (1.3 equiv), MeCN (7 mL), G anode, SS cathode, 25 mA, 3 F mol^−1^. Isolated yields. Diastereomeric ratios determine by ^1^H NMR spectroscopy of chromatographed products. [a] Dimethyl fumarate employed as dipolarophile. [b] Dimethyl maleate employed as dipolarophile. [c] Et_4_NI (0.5 equiv) as mediator, no HFIP, MeOH (7 mL) as solvent, 5 F mol^−1^.

Attention was then turned to scope in dipolarophile reaction partner (Scheme 4). Methyl acrylate and amides were tolerated, with the corresponding isoxazolines **18** and **20** **c**–**20** **e** isolated in good yields. Pleasingly, medicinally relevant amide substituted isoxazoline **20** **c** was obtained in 36 % isolated yield. Acrylonitrile participated in the electrochemical reaction without incident, providing **20** **f** in a good 77 % isolated yield. 1,3‐DC with disubstituted alkene dimethyl fumarate (*trans*‐alkene) gave **20** **a** in 59 % with a diastereomeric of 9 : 1 in favour of the expected *anti*‐diastereoisomer. However, when employing dimethyl maleate (*cis*‐alkene) as the dipolarophile, the same diastereomeric ratio as observed for dimethyl fumarate (*trans*‐alkene), 9 : 1 in favour of the *anti*‐diastereoisomer, was observed. Pericyclic 1,3‐DC reactions are known to proceed stereospecifically such that *cis*‐alkene dipolarophiles would be expected to give rise to *syn*‐diastereomeric isoxazolines, and not *trans*‐isoxazolines, as observed with **20** **a**.^[37]^ Through reaction monitoring and synthesis by non‐electrochemical methods, it was observed that the expected *syn*‐**20** **a** is chemically unstable and rapidly isomerises to the more thermodynamically favourable *anti*‐diastereomeric product. As such, the apparently stereoselective formation of *trans*‐**20** **a** does not rule out a pericyclic 1,3‐DC reaction mechanism (see mechanistic discussion below). Under the optimised conditions, styrene was not tolerated, however, upon switching the mediator to Et_4_NI, solvent to MeOH and transferring a charge of 5 F mol^−1^, the phenyl‐substituted isoxazoline **20** **g** was isolated in 32 % yield. It is suspected that styryl derived dipolarophiles are not well tolerated in this reaction due to their propensity to polymerise under electrochemical conditions.[^38^, ^39^] The limitations of using styrenes in this electrochemical method are further exemplified in the experimental and computational Supporting Information, Sections 1.4.2/3 and 2.5, respectively, and discussed further in the Computational Studies section. The broad applicability of the methodology is exemplified by isolation of **20** **h** and **20** **i** in poor to moderate yields having employed vinylpyridines as dipolarophiles. The results of our electrochemical method, which is selective for α,β‐unsaturated systems, differs, for example, from the *tert*‐butylhypoiodite mediated synthesis of isoxazolines reported by Minakata and co‐workers, which also tolerates styrenes.^[40]^

### Process mass intensity (PMI)

The PMI tool is an open access web tool from the ACS website which can be used to determine the efficiency and greenness of a given reaction.^[41]^ It can be shown that the electrochemical methodology described herein is significantly more process‐friendly (PMI=187) versus a comparable non‐electrochemical reaction^[42]^ (PMI=2167).

Comparison was made between the reaction developed in this work and the publication used as comparator,^[43]^ excluding chromatography which was identical in both cases.

### Product derivatisation

**Scheme 5:**
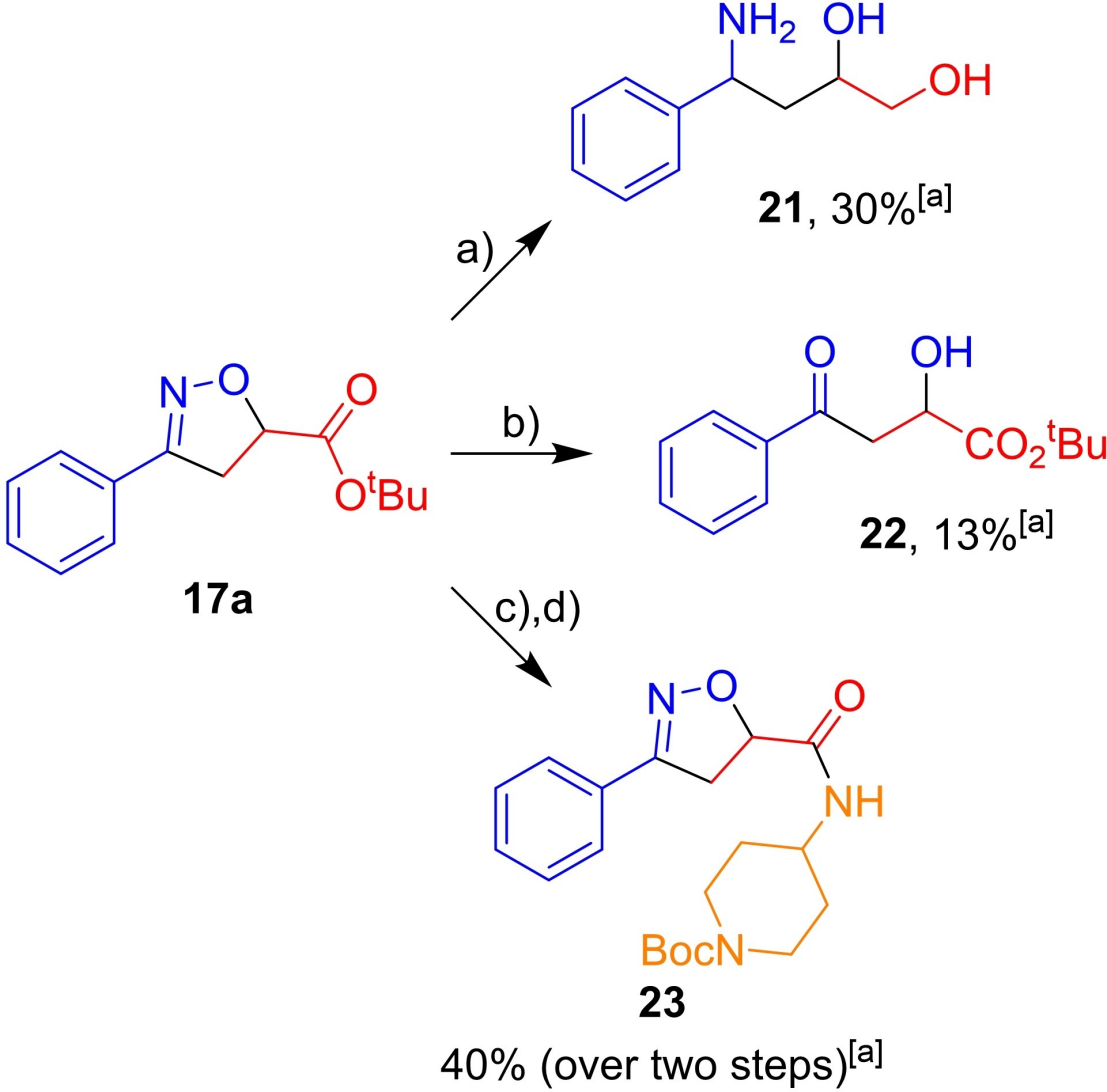
Derivatisation of isoxazoline **17** **a** demonstrating its versatility as a masked structural motif. Conditions: a) LiAlH_4_, THF, 0 °C–RT. b) Et_4_NCl, Fe, EtOH/H_2_O, 90 °C, 13 %. c) LiOH_(aq)_, EtOH, RT, 57 %, d) TCFH, NMI, 4‐amino‐(*N*‐Boc)piperazine, MeCN, RT, 70 %. [a] Unoptimised results. THF=tetrahydrofuran; RT=room temperature; TCFH=*N*,*N*,*N’*,*N’*‐tetramethylchloroformamidinium hexafluorophosphate; NMI=*N*‐methyimidazole.

The utility of isoxazolines as masked structural motifs or medicinally relevant fragments is shown in Scheme 5. Using isoxazolines isolated from our emergent electrochemical method, reduction by LiAlH_4_ ^[44]^ gave the amino diol **21** directly in 30 %. Hydrogenation using iron powder and ammonium chloride gave the aldol product **22** in 13 %.^[45]^ Furthermore, amide **23** was accessed through hydrolysis with LiOH and amide coupling^[46]^ with 4‐amino‐(*N*‐Boc)piperazine in 40 % over the two steps.

### IR monitoring of the bulk medium

**Figure 2:**
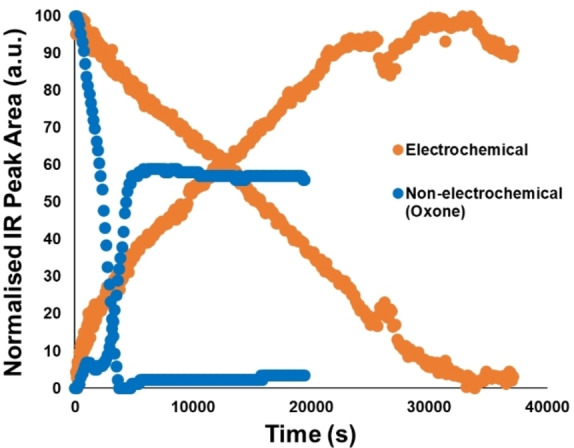
Reaction time courses assessing relative profile shape (not absolute yield) of electrochemical (orange) and non‐electrochemical (blue) reactions between benzaldehyde oxime **15** **a** and *tert*‐butyl acrylate **16** **a** to yield isoxazoline **17** **a**. See Section 8 in the Experimental Supporting Information.

In‐situ reaction monitoring was employed to compare a non‐electrochemical oxone‐mediated method for producing isoxazolines^[42]^ with our electrochemical method. In the non‐electrochemical profile (Figure 2; blue points), an induction period for oxime consumption and isoxazoline generation was observed, consistent with the formation of an intermediate prior to product formation. Once all oxime was consumed, the rate of product formation increases to a maximum.

In the electrochemical reaction (Figure 2; orange points), clear differences are observed. An initial fast rate of product formation slowed to a linear rate over the majority of the time‐course. Oxime decay was immediately linear, with no induction period, and remained linear until the reaction was near completion. This is consistent with the observed pseudo‐zero‐order decay of oxime in the bulk. Note that the IR probe is only able to monitor the bulk solution. As the electrochemical reaction is surface‐mediated, reactive intermediates are presumably present in low concentrations, below the detection limits of the IR device measuring the bulk medium. It is also worth noting that the value of this analysis is limited to the important qualitative analysis of the differences in reaction kinetic profiles for the electro‐ versus non‐electrochemical processes. Further details on the methods used to monitor the non‐electrochemical and electrochemical reactions‐including methods compatible with the ElectraSyn 2.0‐are available in the experimental supporting information, Section 8. Offline sampling NMR validation for the electrochemical process developed herein can be found in the Experimental supporting information (Section 9).

### NMR monitoring of the bulk medium

**Figure 3:**
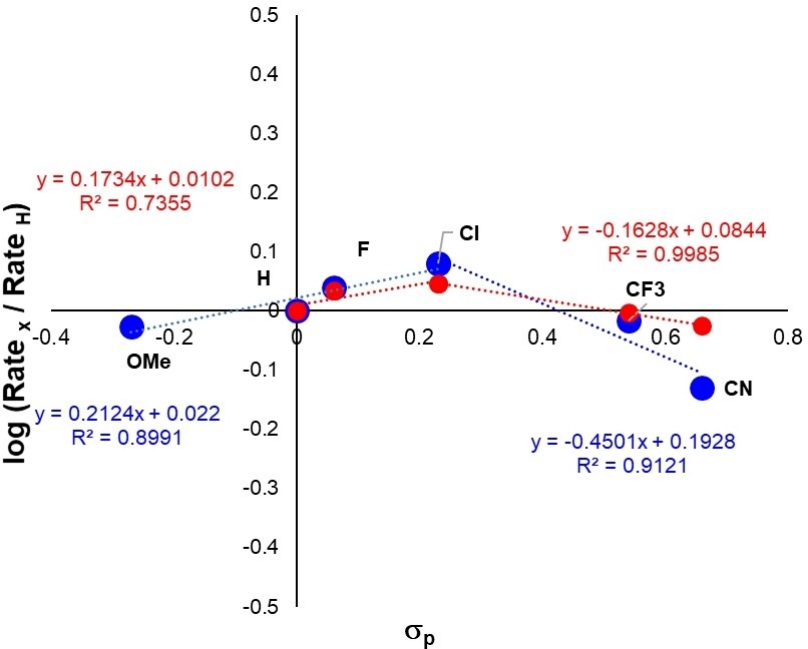
Hammett analysis of observed rates of the decay of oxime **15** (red) and the formation of isoxazoline **17** (blue). Data were analysed from *t*_0_ along the linear pseudo‐zero‐order regime of ^1^H NMR time courses. Swain‐Lupton analysis: F/R=68 : 32.

^1^H NMR kinetic analysis enabled a more detailed investigation of the electrochemical isoxazoline synthesis. Using a range of substituted aldoximes, Hammett analysis of the initial rates (Figure 3) from the majority pseudo‐zero‐order linear profiles (Experimental Supporting Information, Section 9). The analysis evidenced a very shallow inverse V‐shaped plot (well within the arguably negligible range of a single order of magnitude rate difference; Rate(p‐Cl)/Rate(p‐CN)=1.6). Tentatively deeper interpretation of these data may suggest that there is a change in the contributions from the elementary steps of the reaction upon going from electron‐rich to electron‐poor benzaldehyde oximes. Complementary Swain‐Lupton analysis suggested a 62 : 38 split in the field (F) and resonance (R) contributions of aldoxime substituents, respectively.

In contrast, mechanistic studies on related photochemical methods of making isoxazolines from aldoximes by Leonori et al. revealed a non‐inverse V‐shaped Hammett plot, with rates again spanning less than one order of magnitude.^[47]^ In relation to our initial [3+2] cycloaddition hypothesis, the Hammett plots could suggest a change in dominant orbital interactions as the substituent on the oxime is varied.^[48]^

Overall, our data are consistent with a mechanistic process that is pseudo‐zero‐order in aldoxime and is minimally affected by aldoxime aryl ring substitution.

### Kinetic isotope effects

**Scheme 6:**
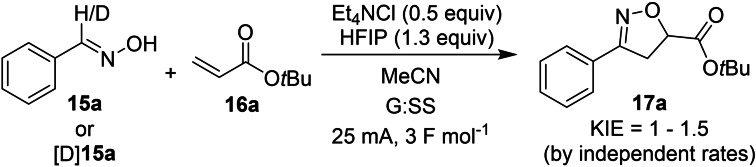
KIE measurements. Conditions: **15** **a** or [D]**15 a** (0.5 mmol), **16** **a** (5 equiv), Et4NCl (0.5 equiv), HFIP (1.3 equiv), MeCN (7 mL), G anode, SS cathode, 25 mA, 3 F mol‐1.

Kinetic isotope effects (KIEs) by independent rate experiments were assessed using aldoxime **15** **a** and deuterated aldoxime [D_1_]**15a**. A small normal KIE of 1.0–1.5 was observed (see Experimental Supporting Information Section 9.5 for detailed reaction profiles and KIE calculations). The negligible KIE suggests that C−H bond breaking (or bond forming) might not feature in the rate‐limiting process of the overall reaction, though this is not in itself conclusive. The small KIE is also reflected in the similar isolated yields from the electrochemical reactions, with [D_1_]**15a** furnishing **17** **a** in 70 % (vs. 78 % for **15** **a**, Scheme 6).

### Cyclic voltammetry and potentiometric data

**Figure 4:**
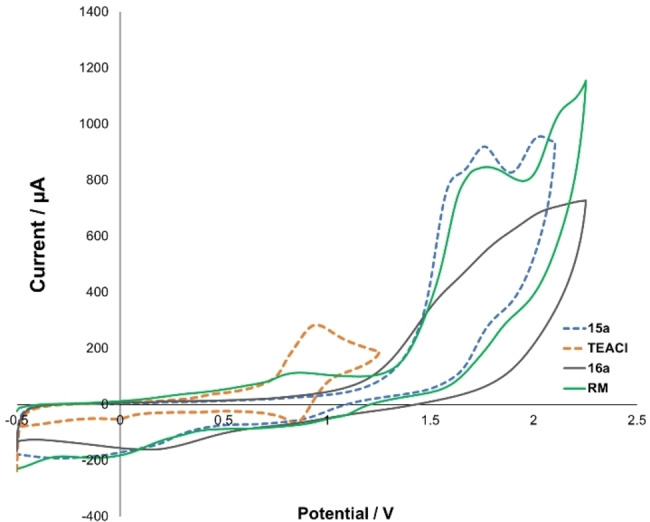
Cyclic voltammetry of the reaction components, with the ferrocene redox couple as external reference. Example conditions: 10 mM Fc, 0.1 M Et_4_NBF_4_‐MeCN, Pt chip electrode, 100 mV s^−1^; RM=reaction mixture; TEACl=tetraethylammonium chloride.

Cyclic voltammetry is a powerful analytical tool for probing electrochemical reactions.^[49]^ All cyclic voltammograms (CVs) herein used the ferrocene/ferrocenium (Fc/Fc^+^) redox couple as a reference voltammogram (Figure 4, and Experimental Supporting Information Section 10). The Et_4_NCl additive gave an electrochemically reversible redox couple with an *E*_1/2_=0.734 V (vs. Fc/Fc^+^, orange line). *tert*‐Butyl acrylate **16** **a** showed no oxidative behaviour within the swept potential range (Figure 4, grey line). Interestingly, the CV of (*E*)‐benzaldehyde oxime **15** **a** shows three distinct irreversible electrochemical oxidative events, all of which are single‐electron oxidations. The oxidation potentials of the first two events are in close proximity to one another. The second peak, being only slightly larger in amplitude than the first, suggests that either the potentials for the two reversible processes (*E*_r_, *E*_q_) are separated by <100 mV or that only the first electron transfer step is reversible and the second quasi‐reversible, with a substantially lower rate constant than that of the first process (*E*_r_, *E*_q_). Both steps might also be quasi‐reversible (*E*_q_, *E*_q_).^[50]^ The rate of the electron transfer for the first step might therefore be substantially lower than that of the second step.

When analysing the reaction mixture as a whole, only two distinct oxidations near those associated with **15** **a** were observed. The oxidation of the chloride anion was also observable in the reaction mixture. Presumed to be a result of changes in solution physical properties and resulting diffusion properties of the reaction components, there was an appreciable decrease in both the reductive and oxidative waves of the chloride couple in the whole reaction versus measurements with the chloride alone (Figure 4, green vs. orange lines). Importantly, the magnitude of the reductive wave appeared to be slightly more attenuated than the oxidative wave for chloride. This difference in oxidative versus reductive peak heights was evident for CV measurements of TEACl alone (|[ox. wave]/[red. wave]|=3.9) and for TEACl in the reaction mixture (|[ox. wave]/[red. wave]|=1.3).^[51]^ These data are consistent with the oxidised chloride species being partly electrochemically reversible, and with known literature on chloride oxidation in acetonitrile,^[52]^ which supports a mechanism manifesting either partial or catalytic oxidative consumption of chloride.

**Figure 5:**
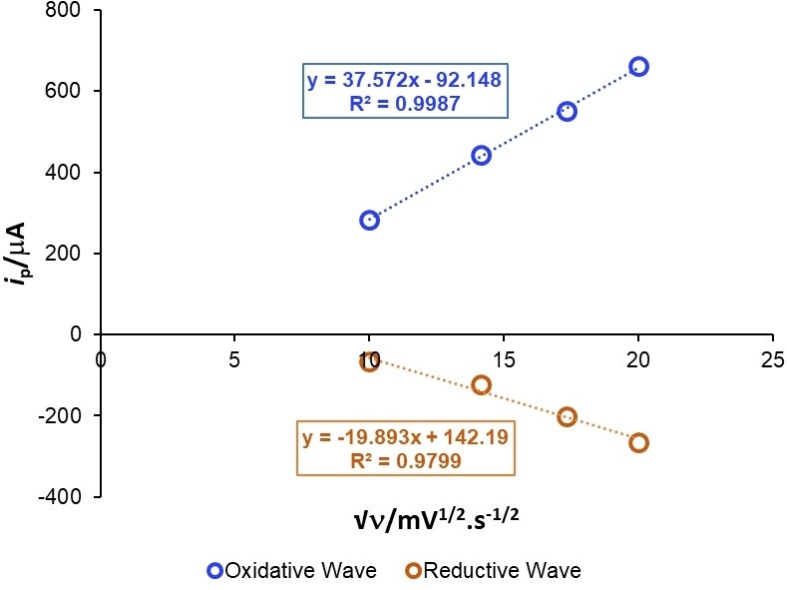
Analysis of the scan rates and plotting *i*_p_ vs. √*ν* for both the oxidative (blue) and reductive (orange) waves of Et_4_NCl. See plots for Solution 2 in Section 10 of the Experimental Supporting Information.

To further probe the reversibility of chloride oxidation, in‐depth analysis of CV scan rates versus current was carried out for solutions of TEACl. Using the Randles‐Sevcik relationship,[^50^, ^53^] exclusively linear plots of the square root of scan rate (√*ν*) versus output current (*i*_p_) of the redox events obtained from the CV experiments were observed. These data, though not conclusive on their own, remained consistent with the interpretation that all key reacting chloride‐derived species diffused freely in solution, with no irreversible chemical adsorption onto the electrodes (exemplified in Figure 5). Complementary analysis of reaction kinetics versus stirrer speed confirmed that rate of isoxazoline formation was independent of stirrer speed at >200 rpm (see Experimental Supporting Information, Section 9.4).

**Figure 6:**
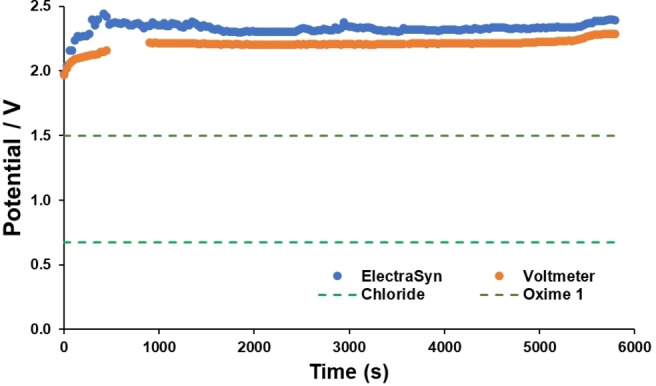
Potential displayed by Electrasyn potentiostat (blue) and by external multimeter (red) over time during the electrochemical synthesis of isoxazolines. Dashed lines represent first CV‐measured oxidation peak currents for TEACl (green) and oxime **15** **a** (orange). The dashed lines are provided solely as an approximate guide to the eye.

Having established the oxidation potentials of the separate components of the reaction, attention was turned to measuring the potential of the electrochemical reaction over time. This electro‐kinetic measurement serves to part‐contextualise the oxidation potentials evidenced in the CV data, and identify which oxidation events are likely to occur under the applied conditions based on the stability of the measured potential within a chosen timeframe.^[54]^ The results are displayed in Figure 6, with the calculated potential at the anode plotted versus time.^[55]^ The measured potential at the anode is approximately steady (as expected under constant current electrolysis), and is always greater than the measured oxidation potentials of all reaction components. These kinetic measurements support at least two mechanisms en route to isoxazoline product **17**; one through mediated consumption of the oxime by an oxidised chlorine species, and another by direct oxime oxidation.

### Additive effects

**Scheme 7:**
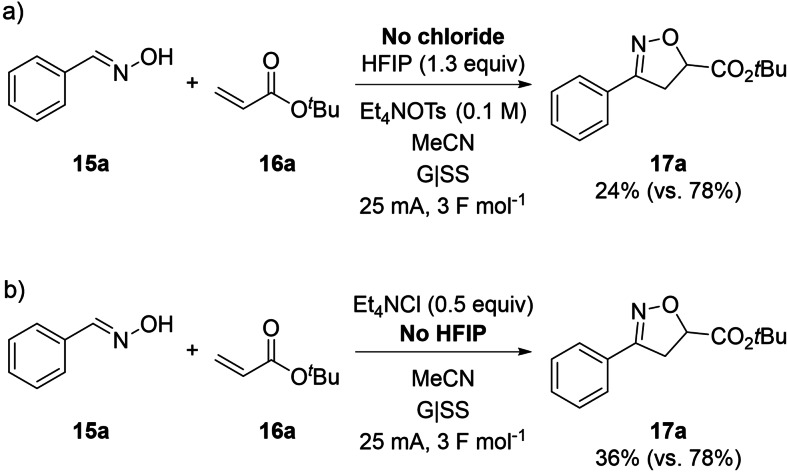
Control experiments to investigate the positive additive effects of chloride and HFIP.

Control experiments performed in the absence of chloride salt – thought to play a dual role of both mediator and electrolyte – showed that the reaction still occurred, albeit with a greatly diminished yield of 24 versus 78 % (Scheme 7a). This result implied possible direct oxidation of the oxime at the anode in the absence of chloride. In this control experiment, Et_4_NCl was replaced with a halide‐free alternative electrolyte, Et_4_NOTs. The resulting decrease in product yield in Scheme 7a could alternatively be interpreted as a simple electrolyte effect. In a second control experiment, the reaction was shown to proceed less efficiently in the absence of the HFIP co‐solvent additive (Scheme 7b). This observation suggested that HFIP acts to stabilise electrochemically‐generated radical intermediates. ^[34]^ The stabilising role of HFIP is also supported by DFT calculations (see below).

### Stereochemical analysis

**Scheme 8:**
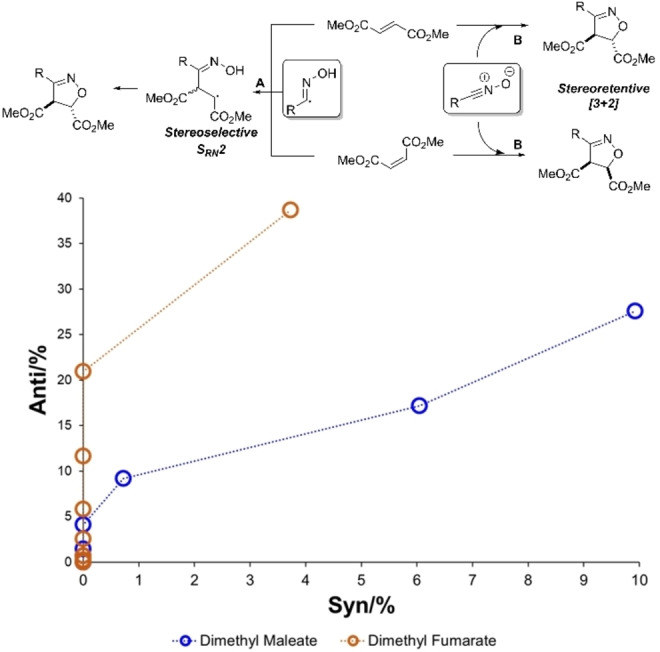
Kinetic analysis of reaction diastereoselectivity, supporting a stepwise mechanism (path A) over a concerted pericyclic mechanism (path B).

The use of 1,2‐disubstituted alkenes as nominal dipolarophiles provides mechanistic insight on account of the observed reaction diastereoselectivity. From Scheme 4, product observed that the reaction was stereoselective (rather than stereospecific) when using *E*‐ and *Z*‐alkenes dimethylfumarate and dimethylmaleate, respectively. Both alkenes selectively produced the *anti*‐ as opposed to the *syn*‐diastereomer of the isoxazoline product. In monitoring the kinetics of this process by ^1^H NMR spectroscopy, it became clear that, for both alkene starting materials, the *anti*‐diastereomer formed first, while the *syn*‐diastereomer emerged later in the reaction time course (Scheme 8, bottom).

Together with the electrochemical data (Figures 4–6), these data supported stepwise mechanism(s) of isoxazoline formation (Scheme 8, path A) rather than the initially hypothesised [3+2] pericyclic mechanism (Scheme 8, path B).

### Computational studies

To support our experiments, a computational mechanistic study was conducted. Using density functional theory (DFT), mechanisms were investigated with single point energy corrections at the M06‐2X/Def2TZVP level of theory, using Truhlar's SMD variation of the integral‐equation‐formalism polarisable continuum model (IEF‐PCM) for acetonitrile. M06‐2X/6‐31+G(d,p) was used to obtain gas phase optimised geometries and free energy corrections. A subset of optimisations were repeated at the M06‐2X/Def2TZVP/SMD(MeCN) level of theory to check assumptions made using the more practicable gas phase calculations. Transition states were characterised by using a single negative vibrational frequency, and their connection to intermediates shown to be consistent with intrinsic reaction coordinate (IRC) calculations.

**Scheme 9:**
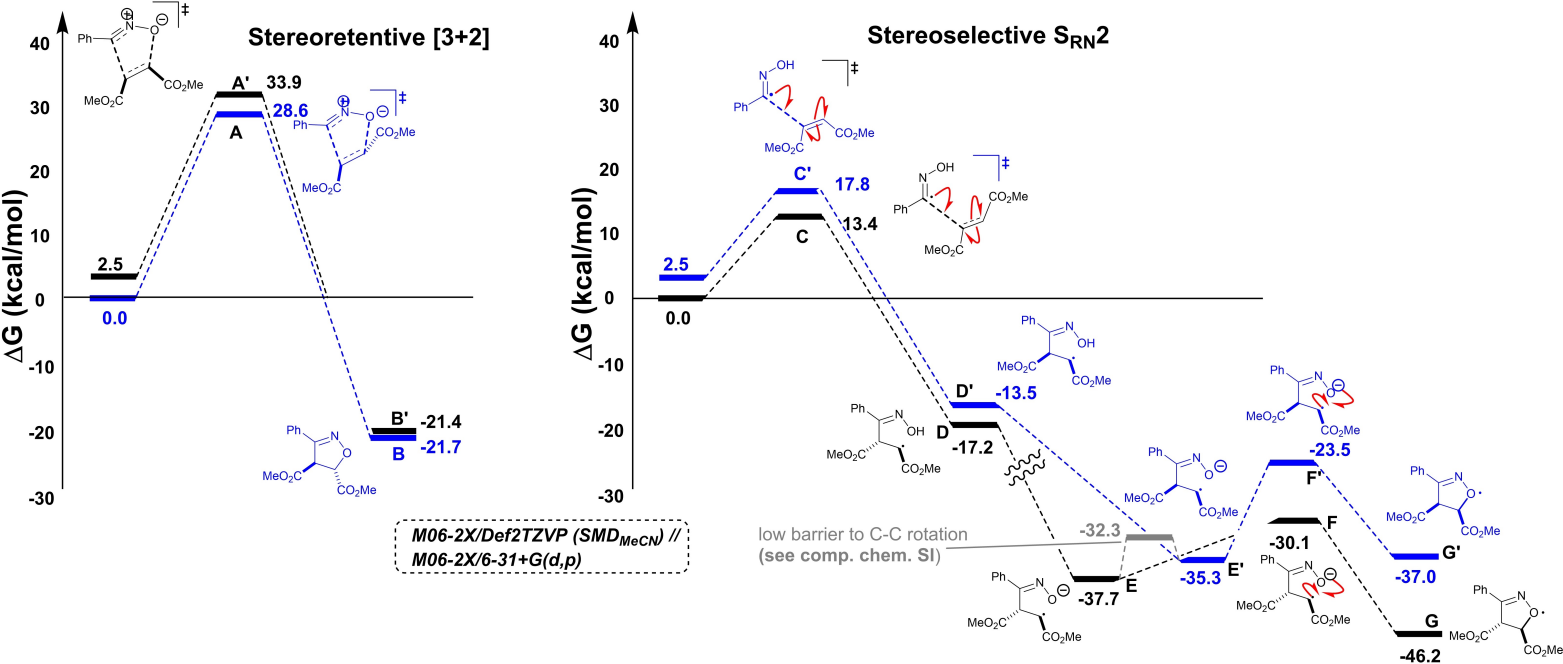
Representative DFT calculations supporting an open‐shell radical mechanism over a closed‐shell [3+2] one towards isoxazoline products.

In relation to Scheme 8, viable transition states were found for both path A (stepwise radical‐mediated) and path B (concerted [3+2] cycloaddition). All calculations were consistent with radical mechanism (path A) and not the initial [3+2] mechanistic hypothesis (path B). Across combinations of substituted oximes and mono‐1,2‐disubstituted alkenes reaction partners used experimentally, the [3+2] cycloaddition was shown to consistently yield calculated barriers 2.5 times higher than for the stepwise radical mechanism (Scheme 9). Consistent with a favoured radical pathway, additional calculations investigating the origins of the nitrile oxide and oximyl radical intermediates key to each investigated pathway shown in Scheme 9 showed that the barrier to oxime N‐chlorination and subsequent E2 elimination was very high (ca. 182 kcal mol^−1^). See Computational Supporting Information, Section 2.6 for full details.

Consistent with the observations of diastereoselectivity, calculations along the radical pathway suggested an approximately 25‐fold rate enhancement for reaction via the *E*‐ over the *Z*‐alkene (C vs. C’, Scheme 9). Potential energy surface scans suggested a low barrier to interconversion of intermediates arising from hydroxyimoyl radical attack on either the *E*‐ or *Z*‐alkene, supporting a diastereoselective process (see Computational Supporting Information, Section 2.3.1). The emergence of the *syn* diastereomer after formation of the *anti*‐diastereomer is consistent with epimerisation of the kinetic *anti* to the near thermodynamically equivalent *syn* product (Scheme 8, and B versus B’, Scheme 9).

**Scheme 10:**
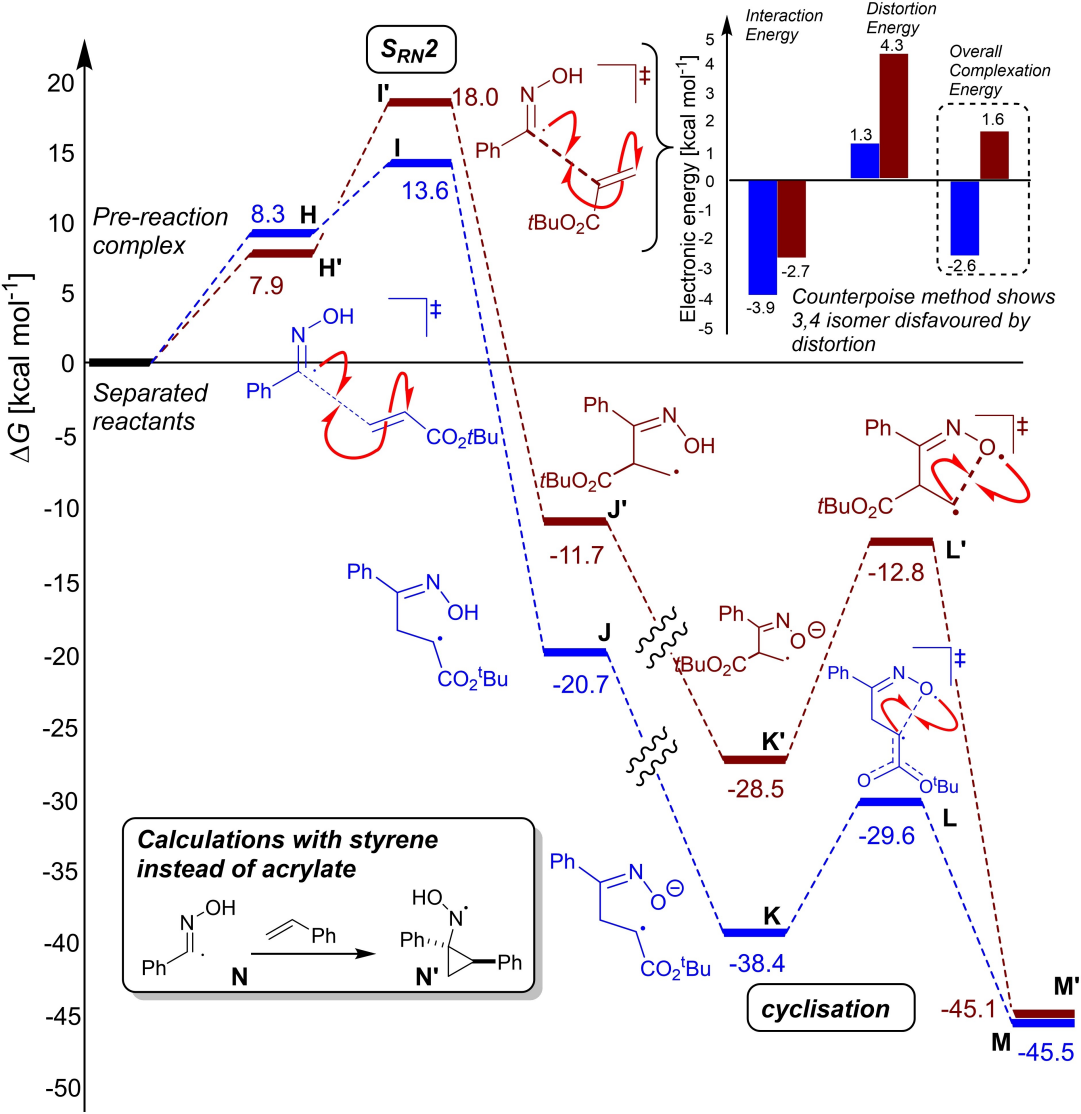
DFT calculations supporting open‐shell radical towards isoxazoline products.

The exclusive formation of the 3,5‐ rather than the 3,4‐regioisomer of the isoxazoline products (i. e., **17** over **18**) was supported by proposed radical mechanism, path A (Scheme 8). Free energy barriers towards the observed 3,5‐product isomer were 5–7 kcal mol^−1^ lower than for the unobserved 3,4‐isomer (H→M vs. H’→M’, Scheme 10). Additional calculations using the Boys‐Bernardi counterpoise scheme were applied to the regioselectivity‐determining S_RN_2 step, thus suggesting that the root cause of the regioselectivity lies in the dominant distortion of the reactive fragments, disfavouring the 3,4‐pathway (Scheme 10 inset; see Computational Supporting Information, Section 2.5). At the SRN2 transition state, the steric clash between the acrylate tert‐butyl and oxime phenyl groups forces the acrylate C−C−C−O dihedral further out of plane (26°) than for the favoured 3,5 isomer (4°). This distortion, in turn, as evidenced by natural bond order (NBO) calculations, reduces the stabilisation energy from oximyl radical to acrylate π*, from about 18 kcal mol^−1^ in the 3,5‐isomer to about 1 kcal mol^−1^ in the 3,4‐isomer. Conversely, calculations along the [3+2] cycloaddition pathway suggested a near‐equal barrier (within 1 kcal mol^−1^) for both regioisomers, with a slight preference for the unobserved isomer. In line with poor compatibility of styrenes with the optimised electrochemical methods, S_RN_2 and cyclisation transition states towards the isoxazoline could not be found; in all cases, the input structures converged on a cyclopropylnitroso radical (Scheme 10 inset; see Computational Supporting Information, Section 2.5).

**Scheme 11:**
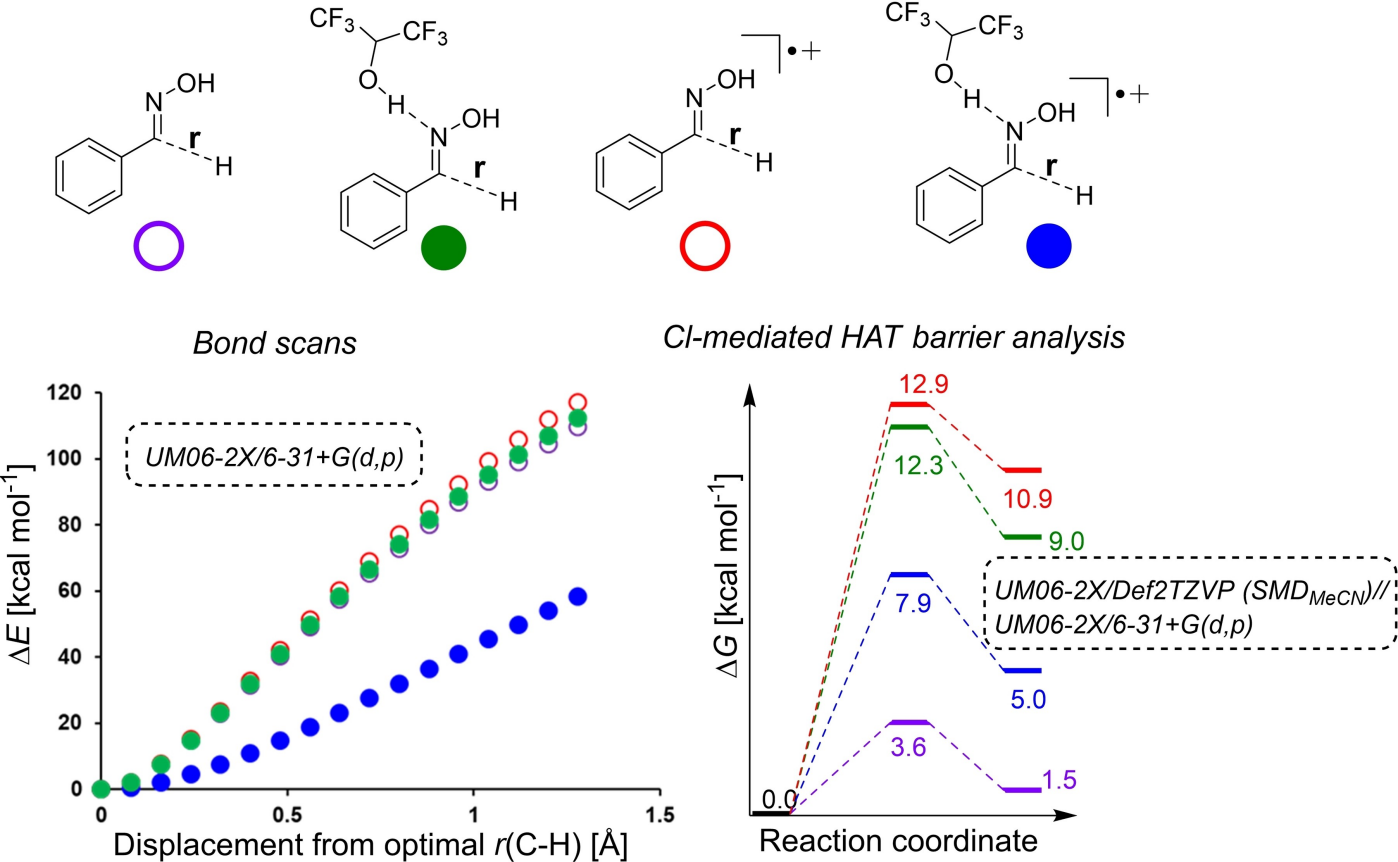
Potential energy surface scans of hydroxyimoyl C−H bonds in different oxidation states with and without HFIP binding (bottom left), and barrier heights for chlorine radical‐mediated H‐atom abstraction (bottom right).

DFT analysis of the radical pathway was also consistent with the observed positive influence of HFIP and chloride additives. As a simplified model of oxime oxidation and H‐atom abstraction, potential energy surface scans were performed to monitor energy changes as a function of hydroxyimoyl C−H bond length. Whilst similar bond length versus electronic energy profiles were evidenced for the neutral singlet oxime, the singlet oxime‐HFIP dimer, and the oxime radical cation, a significant energy stabilisation was shown for the oxime radical cation‐HFIP dimer (Scheme 11, bottom left). This result supports the original hypothesis that HFIP primarily serves as a radical stabiliser, namely the oxime radical cation. Moreover, HFIP is known to be electrochemically stable through a large potential window.^[56]^

Towards an understanding of the role of chloride as mediator, we found viable transition states for chlorine‐mediated H‐atom abstraction relating to all four C−H bond scan structures shown at the top of Scheme 11. The lowest barrier from the pre‐activation complex (3.6 kcal mol^−1^) was found for the neutral singlet oxime in the absence of HFIP. However, there was once again a significant HFIP stabilisation effect for chlorine‐mediated H‐atom abstraction from the oxime radical cation (7.9 vs. 12.9 kcal mol^−1^; Scheme 11, blue vs. red).

In line with the Hammett analysis from Figure 3, DFT analysis of the S_RN_2 and cyclisation steps of the proposed radical mechanism show a dynamic oxime substituent dependence. As the *para*‐substituent becomes more electron‐donating (as quantified by Hammett *σ*_p_), the barrier to nucleophilic attack goes down, whilst the barrier to the secondary cyclisation step goes up.

Though the S_RN_2 step varies little between 13.4–13.8 kcal mol^−1^ (5.2–6.4 kcal mol^−1^ when referenced from the pre‐activation complex), cyclisation appeared appreciably more sensitive, with substituents causing a 7.0–10.5 kcal mol^−1^ spread in barriers. The latter DFT calculated step is primarily field (F) driven, and is consistent with the experimental Swain‐Lupton analysis which inferred 68 : 32 weighting in favour of field of resonance contributions. See experimental and computational supporting information Sections 9.2 and 2.3.3, respectively, for full details.

The chlorine radical‐mediated H‐atom abstraction step was calculated to have a large primary KIE of 5.2. The KIE result appeared to contradict the negligible overall KIE observed experimentally (Scheme 6). Nonetheless, the result fit with the proposed radical mechanism, assuming all steps in the bulk (off‐electrode) are limited by the slow production of chlorine radical on the anode (see Experimental and Computational Supporting Information Sections 9.5 and 1.1.1/2.4, respectively, for KIE data, and Figure 13S inset, below, for additional kinetic analysis). In other words, the observed lack of any KIE could be a false negative result. We also recognise the possible current‐dependent nature of such mechanisms. Therefore, the experimental KIE data presented herein can, beyond any mechanistic consideration, be used as a point of reference for method reproducibility, under the reaction conditions provided.

### Proposed mechanistic pathway

**Scheme 12:**
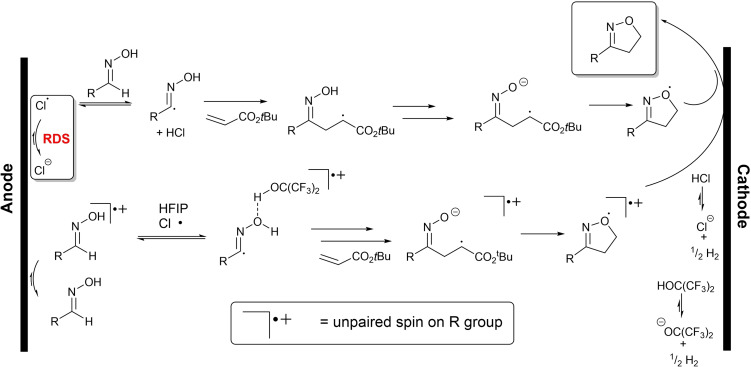
Proposed mechanisms of electrochemical isoxazoline formation.

Through the combination of experimental observations and computational support, the following mechanistic model (running counter to our initial hypothesis) is proposed. In Scheme 12, two contributing radical mechanisms are shown, with the major contributor on top. Chloride, from the Et_4_NCl additive, is oxidised on the anode, generating fleetingly small concentrations of chlorine radical. The chlorine radical concentration is on the order of about 10^−11^ M and is consistent with our geometric model of spherical chloride monolayer coverage of the five exposed faces of the approximately cuboidal electrode (see Computational Supporting Information, Section 1.3). The chlorine radical then participates in H‐atom abstraction from the oxime, generating the nucleophilic hydroxyimoyl radical. The radical reacts with the Michael acceptor, forming the C−C bond of the isoxazoline product. Subsequent formal 5‐exo‐tet cyclisation and cathodic reduction reveals the final isoxazoline product. A secondary pathway involving direct oxidation of the oxime to its radical cation, followed by HFIP‐stabilised and chloride‐mediated HAT, is also proposed, accounting for all experimental observations and DFT support These anode‐ and chloride‐initiated processes are also consistent with avoiding chloride‐free cathodic reduction of a nitrile oxide to nitrile, the likes of which have been reported by Waldvogel and co‐workers.^[33]^

**Figure 7:**
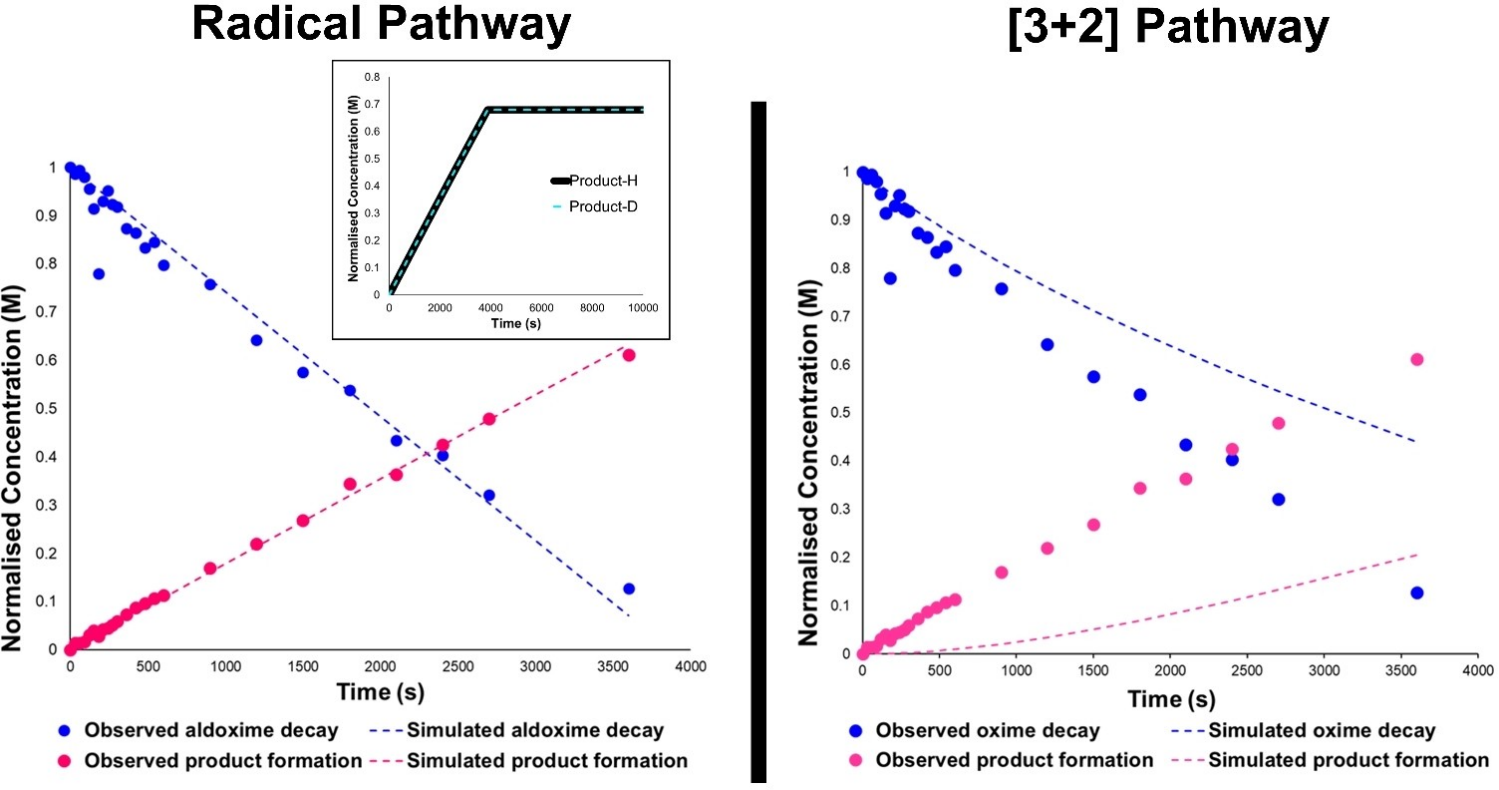
Kinetic simulation of oxime decay and isoxazoline product formation. Dashed lines represent COPASI simulations of the proposed mechanism. Left: radical‐mediated mechanism; right: closed‐shell [3+2] pathway.

Using the COPASI microkinetic modelling software,^[57]^ simulation of the chloride‐mediated mechanism delineated in Scheme 12, with the inclusion of competing hydroxyimoyl radical degradation step, proved an attractive fit to the experimentally‐determined NMR data (Figure 7, top). This mechanism, dominated by a fleeting concentration of surface‐generated oxidised chlorine species, was also consistent with there being no experimentally observable KIE, in spite of there being a KIE of ∼5 for the HAT elementary step (Figure 7, top inset). Conversely, comparative simulations on closed shell mechanisms resulted in no fit to the observed concentration data (Figure 7, bottom). Further details of computational chemistry and kinetics simulations are available in the Supporting Information.

### Limitations of the mechanistic investigation

While endeavouring to provide an experimental and computational (with DFT and kinetic simulations) mechanistic model that is self‐consistent, we note several noteworthy challenges faced that should be considered to avoid over‐ or mis‐interpretation of the data provided. Principally, proton transfer steps involving O−H bonds have not been modelled by DFT and thus do not feature in the potential energy surfaces described herein.

## Conclusions

We have developed a broadly applicable electrochemical methodology for the synthesis of substituted isoxazolines that is quantifiably greener than comparable non‐electrochemical methods. The method enables short reaction times, minimal waste generation, and avoids use of toxic or expensive oxidising reagents. Both aromatic and alkyl substrates are tolerated, and example heteroaryl aldoxime reaction partners have been applied successfully. Mechanistic analysis supported a surface‐mediated electrochemical reaction and a stepwise radical process. The proposed mechanism, which ran counter to our hypothesised design of a genuine pericyclic 1,3‐dipolar cycloaddition towards the targeted isoxazolines, accounted for the observed substituent, additive, and deuterium isotope effects, as well as diastereo‐ and regioselective reaction outcomes.

## Conflict of interest

The authors declare no conflict of interest.

1

## Supporting information

As a service to our authors and readers, this journal provides supporting information supplied by the authors. Such materials are peer reviewed and may be re‐organized for online delivery, but are not copy‐edited or typeset. Technical support issues arising from supporting information (other than missing files) should be addressed to the authors.

Supporting Information

## Data Availability

The data that support the findings of this study are available in the supplementary material of this article.
