# Supplementary material for: Electrochemical Synthesis of Isoxazolines: Method and Mechanism
Source: Chemistry. 2022 Feb 10;28(13):e202103728. doi: 10.1002/chem.202103728 (PMC9303936; doi:10.1002/chem.202103728)
Supplement: Supplementary file 1 — Supporting Information [file CHEM-28-0-s001.pdf]

Chemistry–A European Journal

Supporting Information

Electrochemical Synthesis of Isoxazolines: Method and Mechanism

Samuel D. L. Holman, Alfie G. Wills, Neal J. Fazakerley, Darren L. Poole, Diane M. Coe, Leonard A. Berlouis, and Marc Reid*

Contents

1. COPASI Reaction Kinetics Simulations 1
1.1 Radical Mechanism 2
1.1.1 Simulation of KIE Experiments 3
1.2 Cycloaddition Mechanism 5
1.3 Estimating Maximum Concentration of Chloride Anions at Electrode Surface 6
2. DFT Calculations 10
2.1 [3+2] cycloaddition calculations 11
2.2 [3+2] pseudo exo- versus pseudo endo- (gas phase) 11
2.3 [3+2] versus Radical Pathways 12
2.3.1 Explaining observed diastereoselectivity – [3+2] versus $S_{RN}2$ for dimethyl fumarate and dimethyl maleate 13
2.3.2 Explaining observed diastereoselectivity – [3+2] versus $S_{RN}2$ for dimethyl fumarate and dimethyl maleate 14
2.3.3 Investigating aldoxime substituent effects in the stepwise radical pathway 15
2.4 KIE calculations for radical pathway 17
2.4.1 Finding viable pathways of H-atom abstraction 17
2.4.2 Neutral singlet oxime + chlorine radical (no HFIP): 18
2.4.3 HFIP-bound neutral singlet oxime + chlorine radical: 19
2.4.4 Hydroximoyl radical cation + chlorine radical (no HFIP): 19
2.4.5 HFIP-bound hydroximoyl radical cation + chlorine radical: 19
2.5 Counterpoise calculations to investigate reaction regioselectivity 20
2.6 Additional Calculations with Solvent-inclusive Optimisations 23
2.7 Output Coordinates and thermal data 25
3. References 310

1. COPASI Reaction Kinetics Simulations

All proposed multi-step reaction pathways were simulated by codifying proposed elementary chemical steps in COPASI.[1] All observable species were enumerated with representative reaction concentrations. Where possible, DFT-calculated transition state barriers were used to guide rate constants for some elementary steps in proposed multi-step pathways. All unidentified rate constants were then optimized for best-fit with available experimental NMR kinetics data.

Proposed multi-step reaction pathways were simulated by codifying proposed elementary chemical steps as shown below. Herein, the two main mechanistic hypotheses, as modelled by COPASI, are described.

Direct outputs from COPASI, including rate constant estimates and statistical diagnostics are available in the following two Excel files, included as part of the broader electronic supporting information package:

COPASI_Cycloaddition-Pathway_PARAMETER ESTIMATION.xlsx

COPASI_Radical-Pathway_PARAMETER ESTIMATION.xlsx

Essential details are provided immediately below.

1.1 Radical Mechanism

COPASI encoding

Anode-mediated chloride oxidation

A + B ⟶ C + B

Hydrogen-atom Transfer (HAT) between aldoxime and chlorine radical

C + D ⟶ E + A

Nucleophilic radical attack on acrolate (SRN2)

E + F ⟶ G

Side reaction attenuating desired cyclisation to isoxazoline product

E ⟶ H

Chemical mechanism being represented by COPASI encoding

Anode-mediated chloride oxidation

$Cl^{\ominus}$ + **Anode** $\xrightarrow{-e^{\ominus}}$ Cl· + **Anode**

Hydrogen-atom Transfer (HAT) between aldoxime and chlorine radical

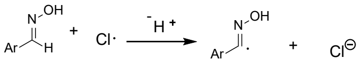

Nucleophilic radical attack on acrolate (SRN2)

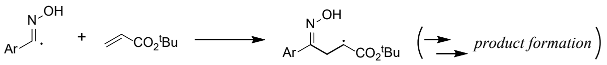

Side reaction attenuating desired cyclisation to isoxazoline product

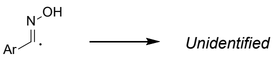

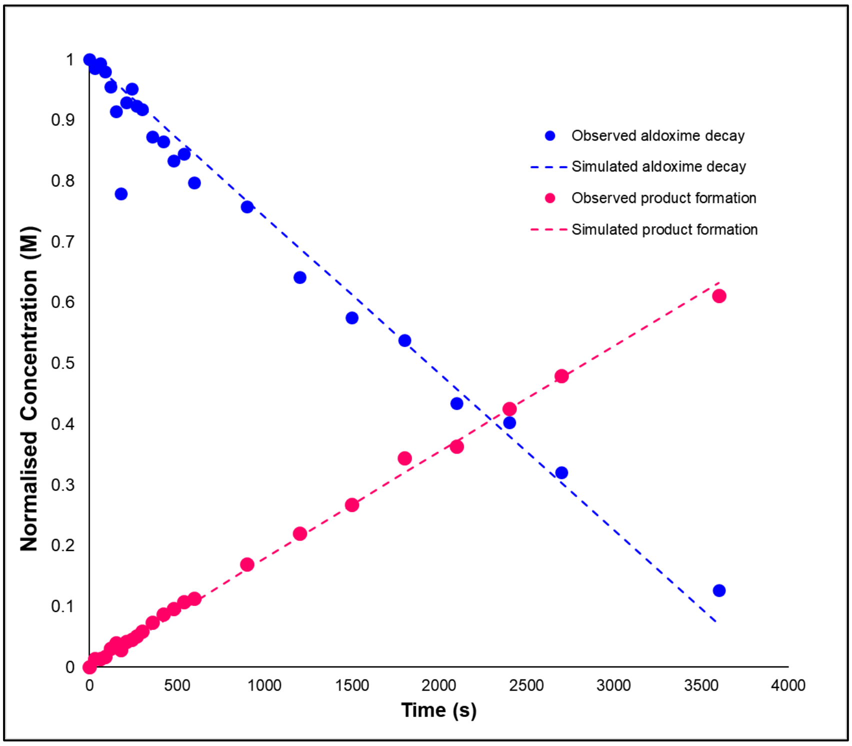

Parameter	Value	Gradient	Standard Deviation
k(ANODE).k1:	1.00E+07	5.62E-11	1.87E+05
k(HAT):	5.22E+07	-1.51E-17	4.45E+13
k(Nu attack):	3.58E+06	-1.29E-11	1.53E+10
k(Side reaction):	7.87E+06	3.63E-11	3.36E+10
[Anode] (active surface site concentration):	5.16E-11	5.95E+09	4.94E-12

1.1.1 Simulation of KIE Experiments

The key and most subtle point in our mechanistic proposal is that the active concentration of oxidised chlorine generated at the electrode surface, which is comparatively and vanishingly small; ca. **5.2 x 10^{-11} M**. This simulated chlorine concentration, borne out of the above-mentioned COPASI simulations, is consistent with the chlorine concentration derived from first principles calculation of available electrode sites, explained in **Section 1.3** of this document (below).

The magnitude of the chlorine concentration dominates the rate expressions. In other words, it is the concentration of chlorine, and not the rate constants (derived from the $\Delta G^{\ddagger}$ of any elementary step) that identify oxidised chlorine generation as the turnover limiting process in this case.

We re-simulated the kinetics of the radical mechanism and modified the **k(HAT)** to simulate the reaction with and without deuteration on the oximyl C–H position. Because KIE calculations (**Section 2.4**; below) revealed a KIE for the HAT elementary step of ca. 5, we simulated the reaction with:

k_H (HAT) ≈ 5.1 x 10^7 a.u.
k_D (HAT) ≈ 1.0 x 10^7 a.u.

When compared, these H and D simulations lead to the same rate of change of product formation. In other words, there was no measurable KIE. This kinetic simulation is consistent with the experimental observation in manuscript **Scheme 6**. Again, it is the fleeting concentration of chlorine that dominates the kinetics, not any one rate constant from any of the subsequent elementary steps.

The scheme below shows the coplotted COPASI-simulated product evolution curves for the deuterated and non-deuterated aldoxime-containing reactions.

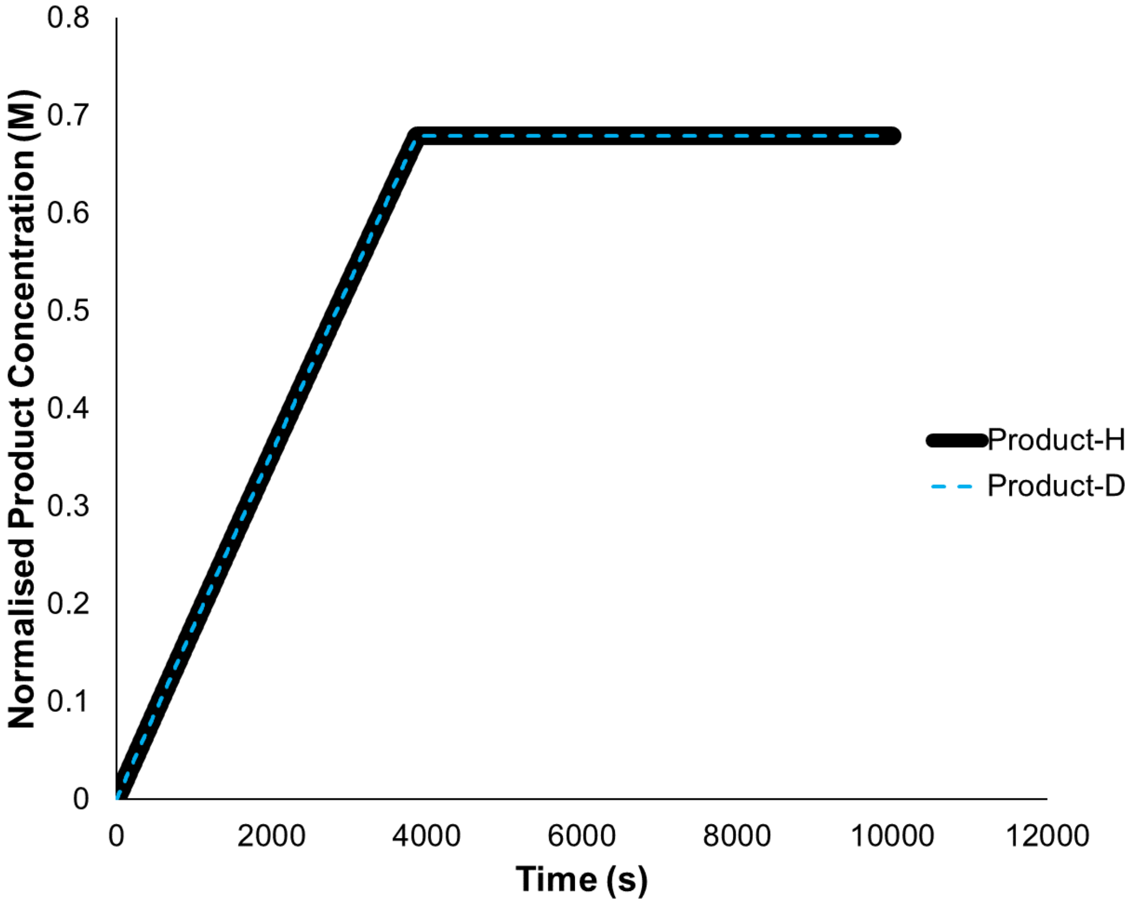

1.2 Cycloaddition Mechanism

COPASI encoding

Anode-mediated chloride oxidation

2A + B ⟶ C + B

Cathode-mediated alkoxide generation

D + E ⟶ F + E

Aldoxime chlorination

G + C ⟶ H + A

Alkoxide-mediated elimination and nitrile N-oxide formation

F + H ⟶ I + J + A

Cycloaddition

I + K ⟶ P

Chemical mechanism being represented by COPASI encoding

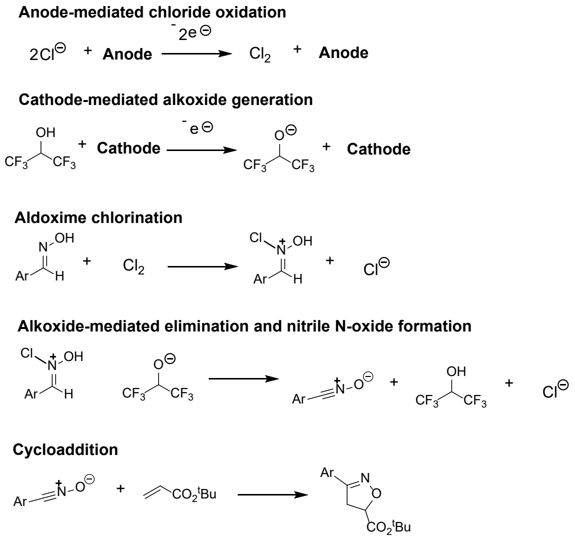

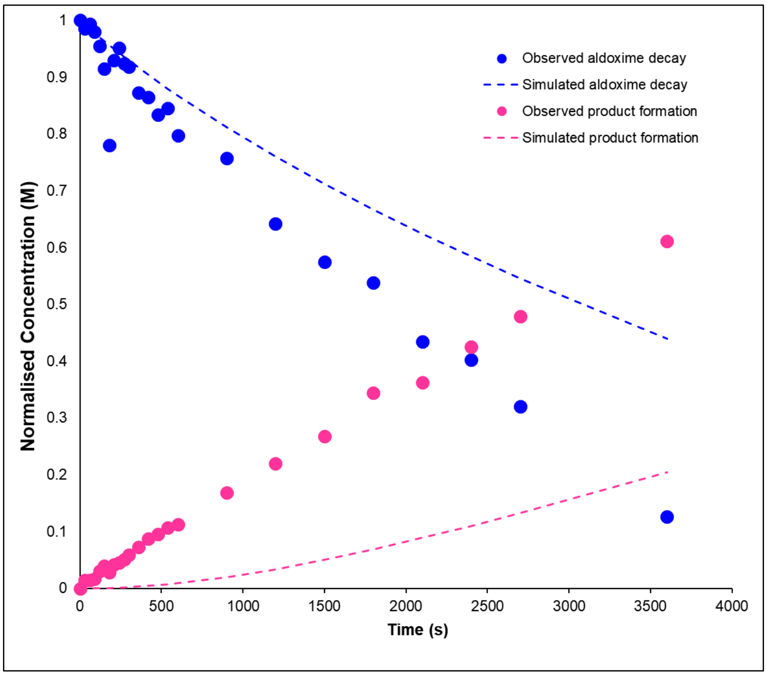

Parameter	Value	Gradient	Standard Deviation
k(ANODE: chlorine generation):	1.00E+07	-4.07E-09	3.40E+07
k(BULK: N-chloro-oximinium elimination):	16205.9	3.16E-08	1.09E+07
k(BULK: cycloaddition):	9.00E-05	-3405.85	0.000291
k(BULK: oxime chlorination):	119.524	3.32E-06	92242
k(CATHODE: base generation):	1.00E+07	-4.45E-08	2.36E+07
[Anode] (active surface site concentration):	1.00E-10	2.95E+07	1.03E-09

1.3 Estimating Maximum Concentration of Chloride Anions at Electrode Surface

Using the following formulae to calculate the number of circles (i.e. chloride anions) that can be packed into a rectangle (one electrode face), **Figure 1**:

$$h = (2m + 1) \cdot r$$

Equation 1; where *h* = height of rectangle, *m* = number of circles in column, *r* = radius of circle.

$$w = \left(2 + (n - 1)\sqrt{3}\right) \cdot r$$

Equation 2; where *w* = width of rectangle, *n* = number of circles in row, *r* = radius of circle.

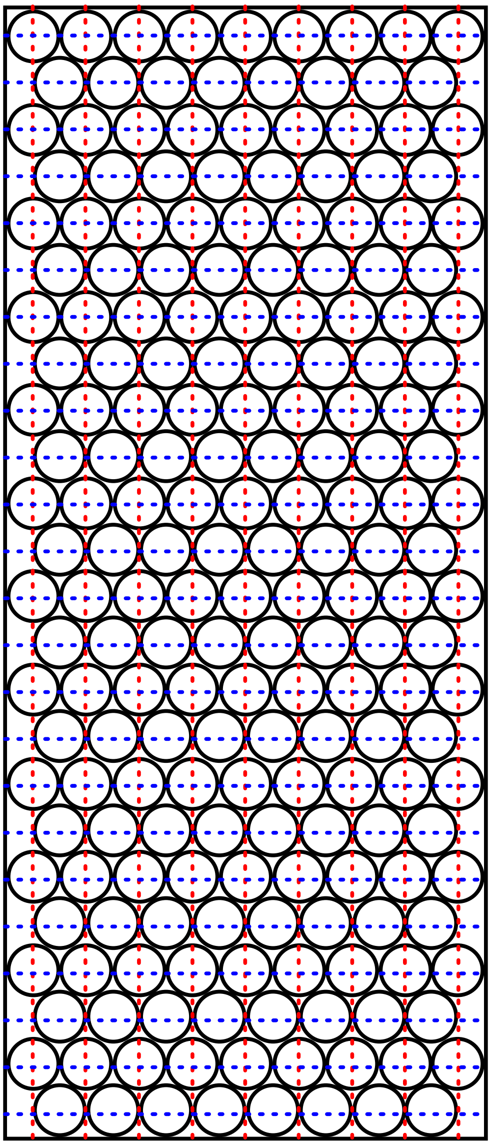

Figure 1: Triangular packing of circles into a rectangle.

The active electrode surface is made up of 5 rectangles (shown in **Figure 2**).

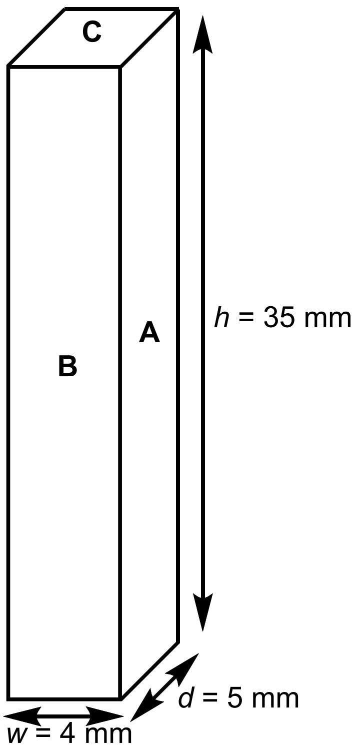

Figure 2: Dimensions of an IKA graphite electrode.

There are two active **A**-faces, two active **B**-faces and one active **C**-face. The final **C**-face is part of the cell cap and is thus not part of the reaction.

Assumptions:

The following assumptions were made:

1. The ionic radius of chloride anion is 0.181 nm
2. In the well-mixed reaction vessel, there is a monolayer of chloride anions on the surface of the electrode
3. There is no competition for anode surface sites from other species (i.e. only chloride is on the surface).
4.

Number of Chloride Anions on the A-Faces:

Firstly, the number of circles in a column *m* in a rectangle of height *h* (in mm):

$$m = \frac{1}{2}\left(\frac{h}{r} - 1\right)$$

$$m = \frac{1}{2}\left(\frac{35\ mm}{0.181\ nm} - 1\right)$$

$$m = 96{,}684$$

Secondly, the number of circles in a row *n* in a rectangle of width *w* (in mm):

$$n = \left(\frac{\frac{w}{r} - 2}{\sqrt{3}}\right) + 1$$

$$n = \left(\frac{\frac{5\ mm}{0.181\ nm} - 2}{\sqrt{3}}\right) + 1$$

$$n = 15948$$

To get the total number of circles T_A in a rectangle of 35 mm x 4 mm:

$$T_A = m \times n$$

$$T_A = 96{,}684 \times 15948$$

$$T_A = 1{,}541{,}916{,}432$$

Number of Chloride Anions on the B-Faces:

Firstly, the number of circles in a column *m* in a rectangle of height *h* (in mm):

$$m = \frac{1}{2}\left(\frac{h}{r} - 1\right)$$

$$m = \frac{1}{2}\left(\frac{35\ mm}{0.181\ nm} - 1\right)$$

$$m = 96{,}684$$

Secondly, the number of circles in a row *n* in a rectangle of width *w* (in mm):

$$n = \left(\frac{\frac{w}{r} - 2}{\sqrt{3}}\right) + 1$$

$$n = \left(\frac{\frac{4\ mm}{0.181\ nm} - 2}{\sqrt{3}}\right) + 1$$

$$n = 12{,}758$$

To get the total number of circles T_B in a rectangle of 35 mm x 4 mm:

$$T_B = m \times n$$

$$T_B = 96{,}684 \times 12{,}758$$

$$T_B = 1{,}233{,}494{,}472$$

Number of Chloride Anions on the C-Faces:

Firstly, the number of circles in a column *m* in a rectangle of height *h* (in mm):

$$m = \frac{1}{2}\left(\frac{h}{r} - 1\right)$$

$$m = \frac{1}{2}\left(\frac{5\,mm}{0.181\,nm} - 1\right)$$

$$m = 13{,}811$$

Secondly, the number of circles in a row *n* in a rectangle of width *w* (in mm):

$$n = \left(\frac{\frac{w}{r} - 2}{\sqrt{3}}\right) + 1$$

$$n = \left(\frac{\frac{4\,mm}{0.181\,nm} - 2}{\sqrt{3}}\right) + 1$$

$$n = 12{,}758$$

To get the total number of circles T_C in a rectangle of 35 mm x 4 mm:

$$T_C = m \times n$$

$$T_C = 13{,}811 \times 12{,}758$$

$$T_C = 176{,}200{,}738$$

Total Number of Chloride Anions Over Whole Active Surface:

The total number of chloride anions over the whole active service of the electrode, N_{Cl}^{S}, can be calculated by:

$$N_{Cl}^{S} = 2T_A + 2T_B + T_C$$

$$N_{Cl}^{S} = 5{,}727{,}022{,}546$$

Calculations of Molarity and Percentage of Chloride Anions at Electrode Surface:

We know that there is 0.25 mmol of chloride in the bulk solution and so the number of chloride anions in solution, N_{Cl}^{B}, is calculated by:

$$N_{Cl}^{B} = Mole \times N_A$$

$$N_{Cl}^{B} = 0.25 \times 10^{-3} \times 6.02214 \times 10^{23}$$

$$N_{Cl}^{B} = 1.506 \times 10^{20}$$

Therefore, percentage of bulk chloride anions, *%Chloride*, at the electrode surface is:

$$\%Chloride = \frac{N_{Cl}^{S}}{N_{Cl}^{B}} \times 100\%$$

$$\%Chloride = \frac{5{,}727{,}022{,}546}{1.506 \times 10^{20}} \times 100\%$$

$$\%Chloride = 3.8 \times 10^{-11}\%$$

Where N_A = Avogadro's number.

We can also calculate the concentration of chloride anions at the active surface of the electrode, $[Cl^-]^S$,:

$$mol_{Cl}^{S} = \frac{N_{Cl}^{S}}{N_A}$$

$$mol_{Cl}^{S} = \frac{5{,}727{,}022{,}546}{6.02214 \times 10^{23}}$$

$$mol_{Cl}^{S} = 9.510 \times 10^{-12} mmol$$

$$[Cl^-]^S = \frac{mol_{Cl}^{S}}{V}$$

$$[Cl^-]^S = \frac{9.510 \times 10^{-12}}{7}$$

$$[Cl^-]^S = 1.359 \times 10^{-12} M$$

Where V = volume of solution (7 mL).

2. DFT Calculations

Using density functional theory (DFT), mechanisms were investigated with single point energy corrections at the M06-2X/Def2TZVP level of theory, using Truhlar's SMD variation of the integral-equation-formalism polarizable continuum model (IEF-PCM) solvent model for acetonitrile. M06-2X/6-31+G(d,p) was used to obtain gas phase free energy corrections and optimized geometries. Transition states were characterized *via* a single negative vibrational frequency, and their connection to expected intermediates confirmed by intrinsic reaction coordinate (IRC) calculations.

Density functional theory (DFT),[2,3] was employed to calculate the gas-phase electronic structures and energies for all species. All structures thus far have been optimized with the hybrid meta-GGA exchange correlation functional M06-2X.[4] The M06-2X density functional was used in conjunction with the 6-31+G(d,p)[5,6] basis set for all atoms. Harmonic vibrational frequencies were at the same level of theory to characterize minima (no imaginary frequencies) and transition states (one negative frequency). All calculations were run at the default temperature of 298.15 K. Single point energy corrections to gas phase geometry optimizations at the M06-2X/Def2TZVP level of theory, using Truhlar's SMD variation[7] of the integral-equation-formalism polarizable continuum model (IEF-PCM) solvent model[8] for acetonitrile (ε = 35.688). For all calculations on structures with unpaired electrons, the unrestricted functionals were used applied the prefix "U" for the input specification (see Table S1, below). All calculations, except for kinetic isotope effects, were exclusively performed using Gaussian 09 quantum chemistry program package.[9] All coordinates provided below are listed in Cartesian format in a separate SI file, with charge and multiplicity of each system given at the top of the coordinate list (e.g. 0 1 = neutral closed shell singlet; 1 1 = 1+ charged closed shell singlet; -1 2 = radical anion; 0 2 = neutral doublet/single unpaired electron). Thermal data presented above the coordinates are those stripped from the associated gas-phase frequency calculation. Further computational details specific to certain parts of the manuscript are described below in the relevant sections. All rendered 3D images were produced using CYLview (version 1.0.561).

Calculation Type	Key Details	Reference(s)
Quantum Chemistry Package	Gaussian 09, Revision D0.1	9
Visualizations	CYLview v1.0.561 BETA	10
Gas phase calculations	`# um062x/6-31+G(d,p) opt=(tight) freq Int(UltraFineGrid) pop=(nboread,SaveNBOs) gfoldprint scf=(maxcycle=1000)`<br><br>*Note: "u" dropped from "um062x" for closed shell [3+2] cycloaddition calculations.*	4,11,12
Single point corrections to gas phase optimizations	`# um062x/Def2TZVP sp Int(UltraFineGrid) nosymm pop=(full,nboread,SaveNBOs) density=current scf=(maxcycle=1000) scrf=(solvent=Acetonitrile,smd)`<br><br>*Note: "u" dropped from "um062x" for closed shell [3+2] cycloaddition calculations.*	4,7,11,12
Post-coordinate input for NBO calculations	`$NBO PLOT BNDIDX $END`	11
Dihedral scan calculations	`# um062x/6-31G(d) opt=(ModRedundant) Int(UltraFineGrid) nosymm`	N/A
Post-coordinate input for dihedral scan calculations	`D 17 16 15 18 S 36 10.0`	13
KIE calculations	KINISOT.py v 1.0.1	14,15

2.1 [3+2] cycloaddition calculations

As shown in the scheme below, pseudo-exo and pseudo-endo geometries were explored for all dipole-dipolarophile reaction partners.

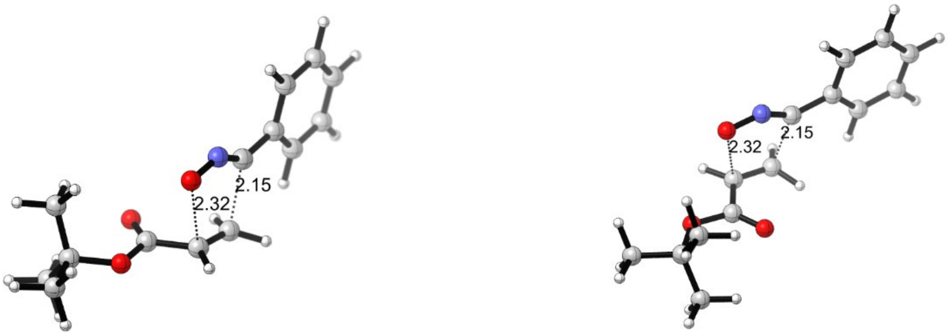

2.2 [3+2] pseudo exo- versus pseudo endo- (gas phase)

Exploration of pseudo-exo and pseudo-endo transition state geometries for the [3+2] pathway towards the isooxazoline products revealed a less than 1 kcal/mol deviation in energy for all cases explored.

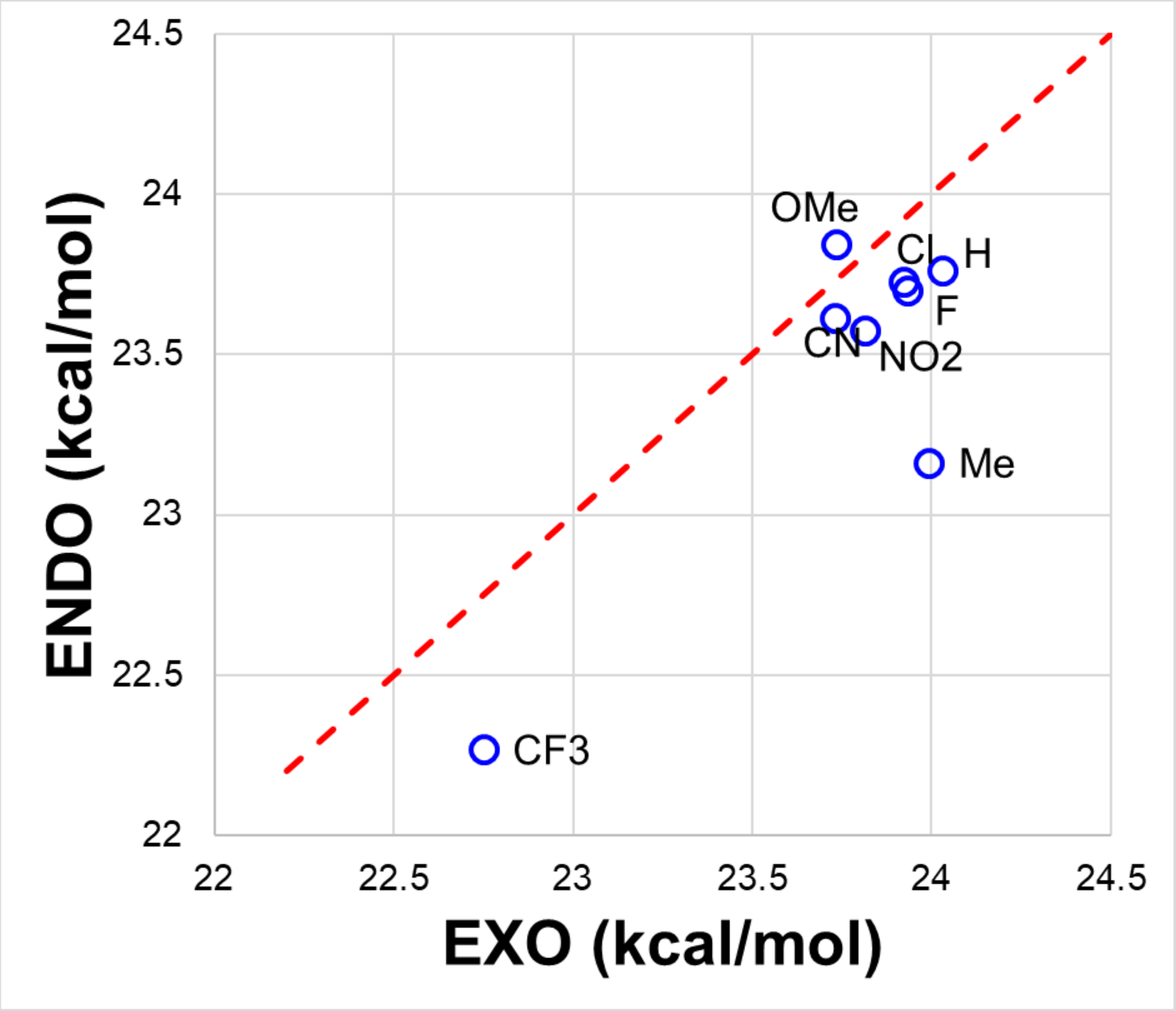

2.3 [3+2] versus Radical Pathways

2.3.1 Explaining observed diastereoselectivity – [3+2] versus $S_{RN}2$ for dimethyl fumarate and dimethyl maleate

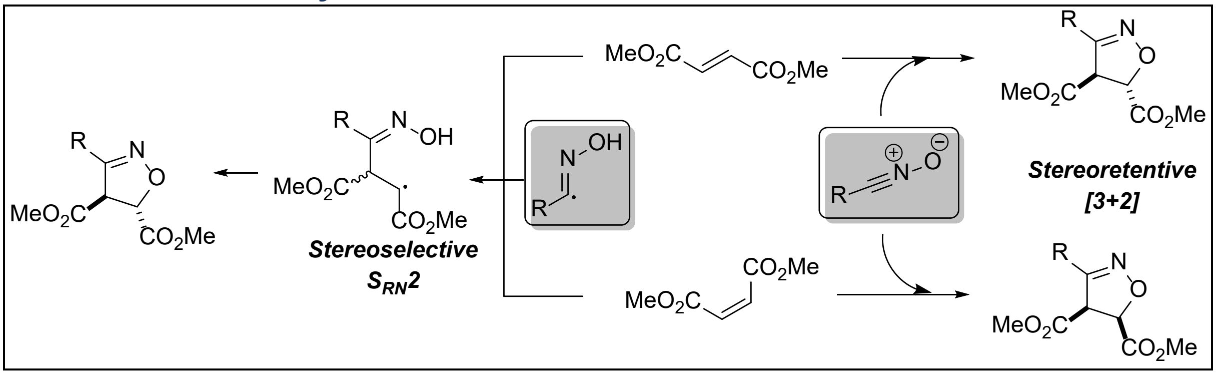

Comparing the key bond-forming steps towards the isooxazoline product reveled that the radical pathway was lower for both the fumarate and the maleate case.

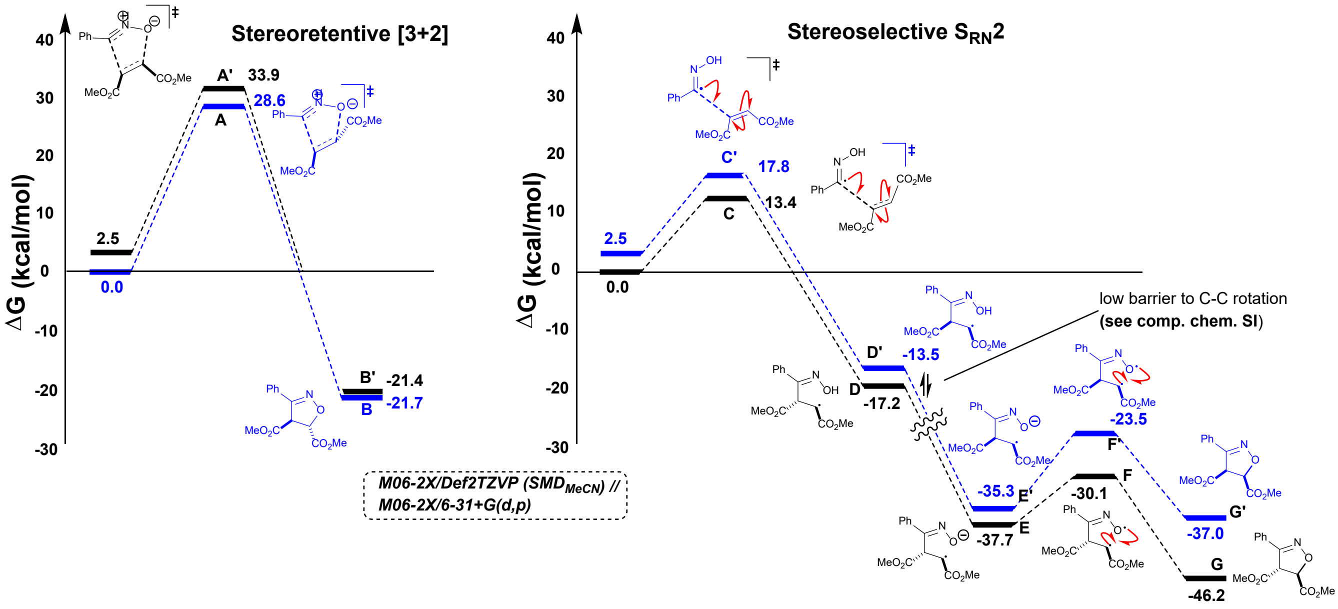

Consistent with the observed diastereoselectivity of the reaction, radical pathway calculations remained consistent with rotation around the key C-C bond being energetically accessible to enable both possible diastereomeric intermediates to interconvert. The following data relate to discussion surrounding **Scheme 9** (right, structures **D** and **D'**) in the manuscript.

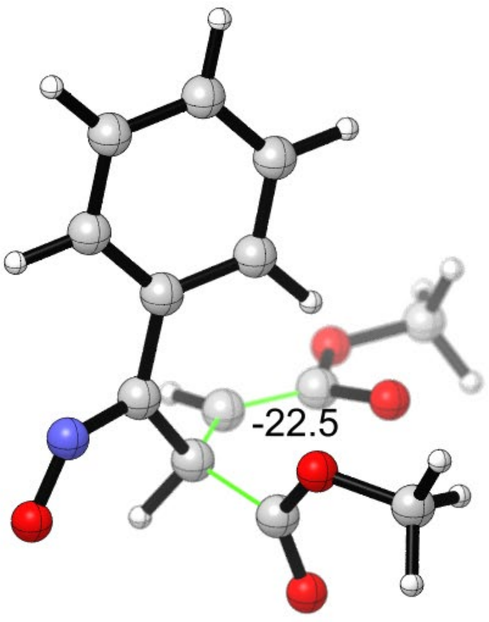

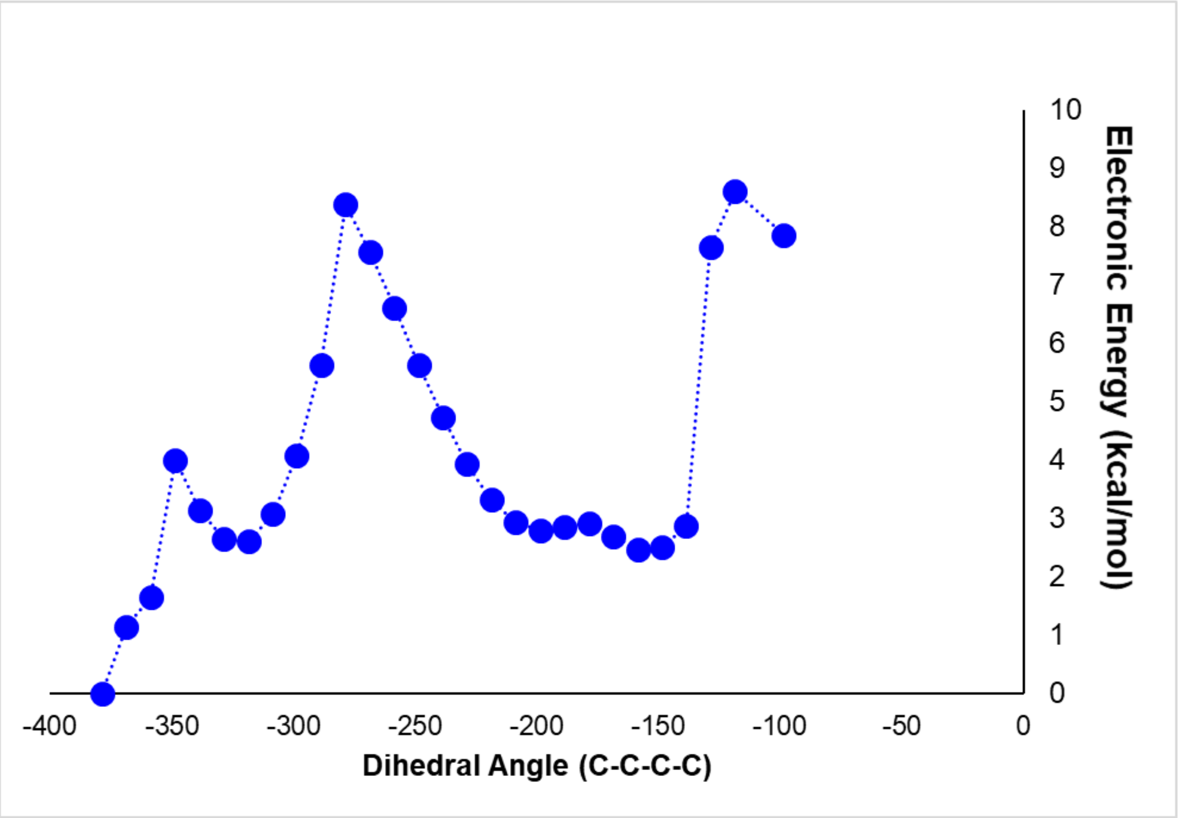

2.3.2 Explaining observed diastereoselectivity – [3+2] versus $S_{RN}2$ for dimethyl fumarate and dimethyl maleate

Beyond the fumerate/maleate substrates, the library of para-substituted aldoxime reactants were investigated via DFT. A comparison of gas phase transition state free energies for key steps in [3+2] and stepwise radical revealed that the radical pathway was unanimously lower in energy.

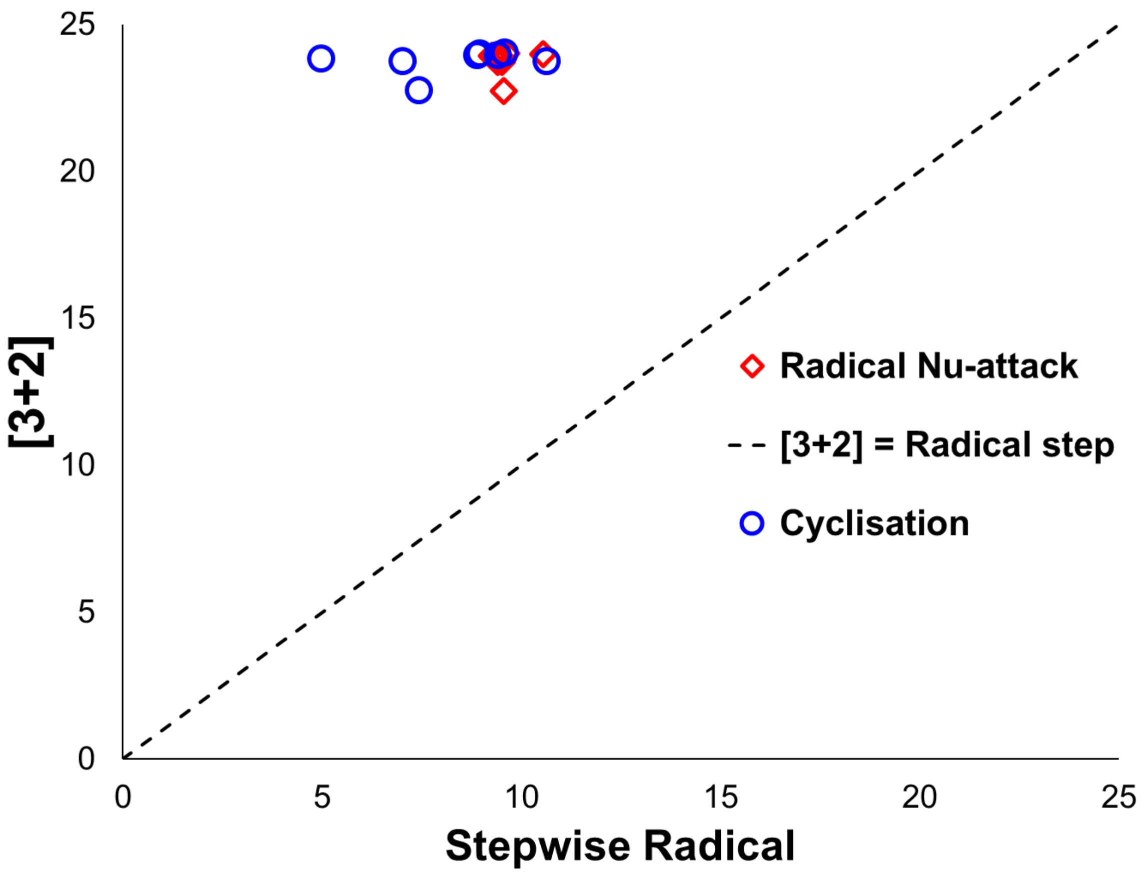

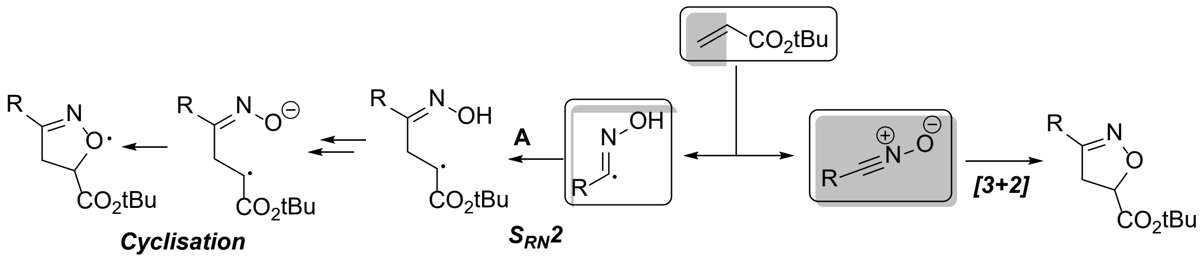

2.3.3 Investigating aldoxime substituent effects in the stepwise radical pathway

A deeper investigation of the two-step radical mechanism revealed that the $S_{RN}2$ step is less sensitive to aldoxime substation than the secondary cyclisation step. This point was made more definitive in solution phase calculations versus gas phase calculations.

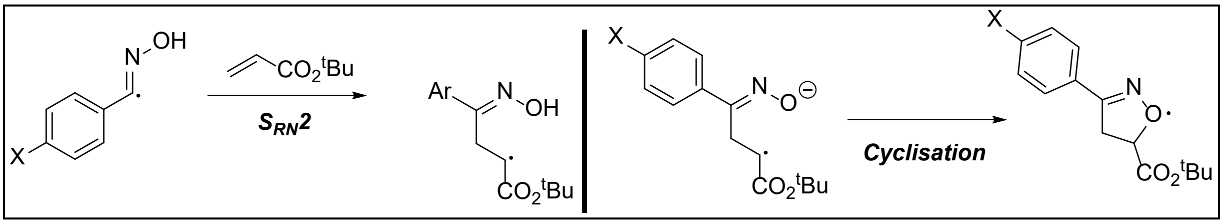

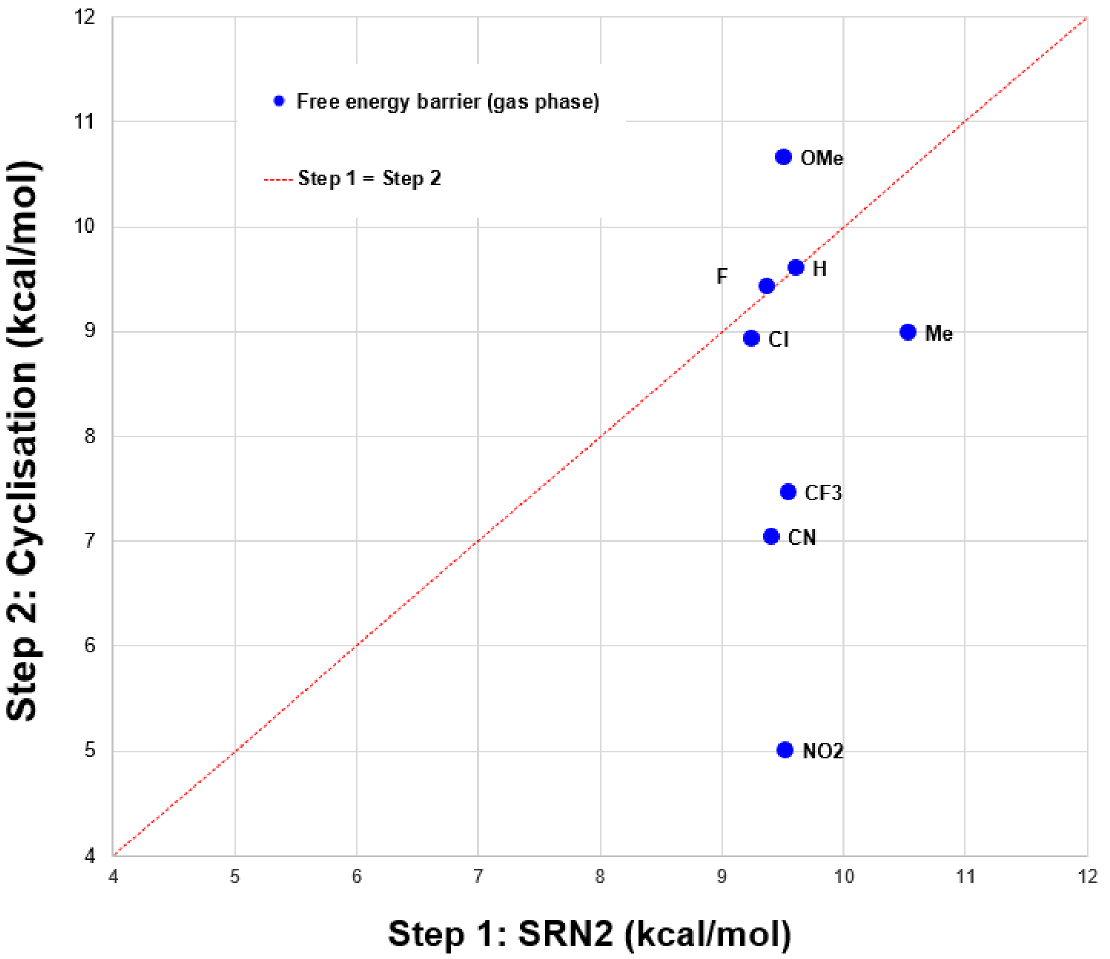

The $S_{RN}2$ step is only more energetically demanding versus the cyclisation if all calculations consider the oxime and acrylate as completely separated in determining the reference energy ground state prior to the $S_{RN}2$ transition state. If, instead, the pre-activation complex (determined as a local minimum from IRC calculations from the transition state back to reactants), then one would come to the opposite conclusion – that the cyclisation and NOT the $S_{RN}2$ is more energetically demanding. See below.

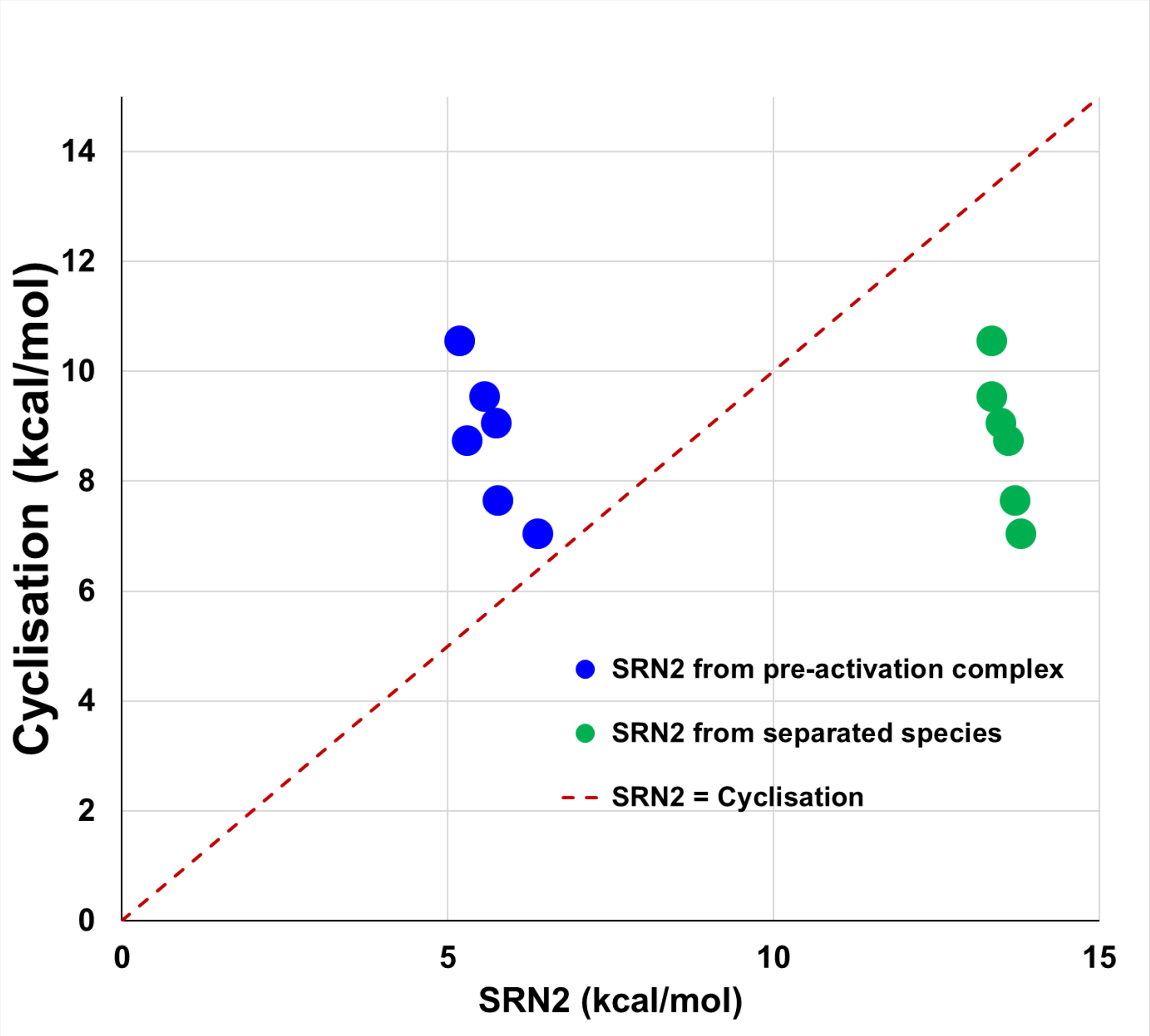

2.4 KIE calculations for radical pathway[14,15]

Output KIE values found for the hydrogen atom transfer elementary step are all highlighted in red in the tables below. All calculations used the reactant and transition state DFT calculation results and Paton et. al's Python refactoring[15] of Rzepa's KINISOT program.[14]

2.4.1 Finding viable pathways of H-atom abstraction

Part reproduced from **Scheme 11**, the figure below summarises the 4 identified chlorine-mediated H-atom abstraction pathways identified and subsequently applied to KIE investigations.

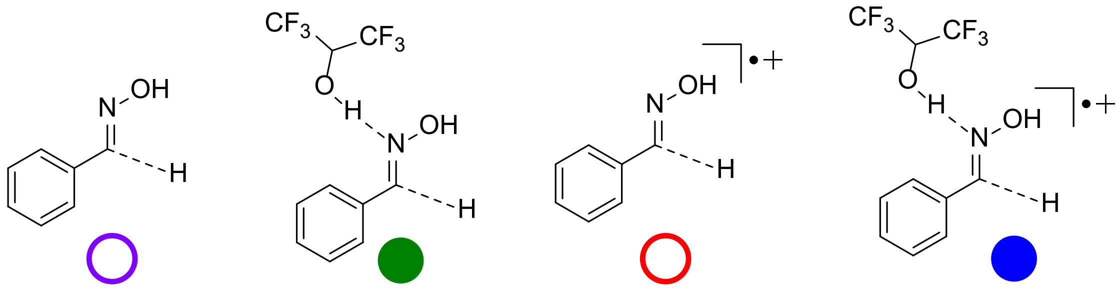

Chlorine-mediated H-atom abstraction free energies:

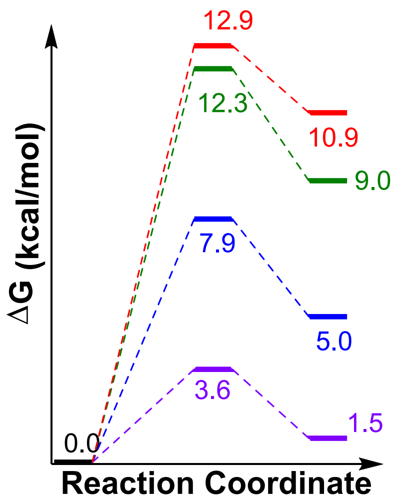

In **sections 2.4.2 – 2.4.5** below, the four mechanisms identified above were applied to KINISOT. Identifying the abstracted H-atom as that to be labelled as deuterium. Within the KINISOT calculation framework. In all cases, a normal primary KIE of >5 was found.

2.4.2 Neutral singlet oxime + chlorine radical (no HFIP):

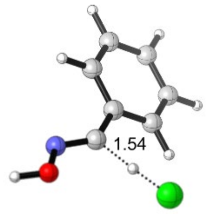


```
KINISOT.py v 1.0.1: 2020-08-31 12:04
Unable to find vibrational scaling factor for UM062X/6-31+G(d,p); using value of 1.0


                                                     Temp = 298.15K / Vib. scale factor = 1.0
                                                    V-ratio         ZPE         EXC        TRPF         KIE     1D-tunn    corr-KIE
o R                                                 ----------------------------------------------------------------------------
o TS                                                 159.9
o R: iso @ 16                                                3.099e+01   1.133e+00   2.652e+00
o TS: iso @ 16                                       152.0   5.834e+00   1.157e+00   2.783e+00
                                                    ----------------------------------------------------------------------------
  KIE @ 298.15 K                                  1.052222    5.311292    0.979316    0.952783    5.214637    1.002427    5.227292
                                                    ----------------------------------------------------------------------------
```

2.4.3 HFIP-bound neutral singlet oxime + chlorine radical:

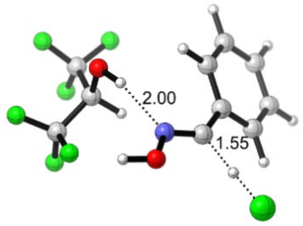


```
KINISOT.py v 1.0.1: 2020-10-09 17:14
Unable to find vibrational scaling factor for UM062X/6-31+G(d,p); using value of 1.0

                                                 Temp = 298.15K / Vib. scale factor = 1.0
                                                V-ratio      ZPE        EXC        TRPF        KIE     1D-tunn    corr-KIE
o R                                             -----------------------------------------------------------------------
o TS                                              186.0
o R: iso @ 2                                             3.174e+01  1.127e+00  2.646e+00
o TS: iso @ 2                                     175.8  5.809e+00  1.159e+00  2.795e+00
                                                -----------------------------------------------------------------------
  KIE @ 298.15 K                               1.057967   5.463665   0.972121   0.946646   5.319420   1.003630   5.338730
                                                -----------------------------------------------------------------------
```

2.4.4 Hydroximoyl radical cation + chlorine radical (no HFIP):

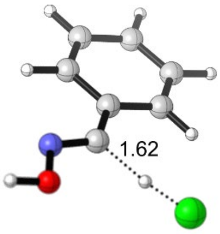


```
KINISOT.py v 1.0.1: 2020-08-31 12:36
Unable to find vibrational scaling factor for UM062X/6-31+G(d,p); using value of 1.0

                                                 Temp = 298.15K / Vib. scale factor = 1.0
                                                V-ratio      ZPE        EXC        TRPF        KIE     1D-tunn    corr-KIE
o R                                             -----------------------------------------------------------------------
o TS                                              235.9
o R: iso @ 16                                            3.186e+01  1.119e+00  2.628e+00
o TS: iso @ 16                                    222.3  5.417e+00  1.255e+00  2.783e+00
                                                -----------------------------------------------------------------------
  KIE @ 298.15 K                               1.061018   5.881243   0.892088   0.944074   5.255400   1.006176   5.287855
                                                -----------------------------------------------------------------------
```

2.4.5 HFIP-bound hydroximoyl radical cation + chlorine radical:

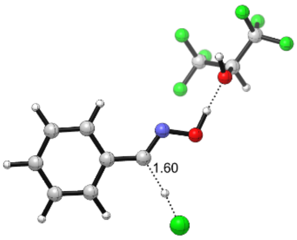


```
KINISOT.py v 1.0.1: 2020-10-09 16:58
Unable to find vibrational scaling factor for UM062X/6-31+G(d,p); using value of 1.0

                                                 Temp = 298.15K / Vib. scale factor = 1.0
                                                V-ratio      ZPE        EXC        TRPF        KIE     1D-tunn    corr-KIE
o R                                             -----------------------------------------------------------------------
o TS                                              218.3
o R: iso @ 2                                             3.250e+01  1.118e+00  2.659e+00
o TS: iso @ 2                                     207.1  5.605e+00  1.229e+00  2.803e+00
                                                -----------------------------------------------------------------------
  KIE @ 298.15 K                               1.053875   5.798449   0.909715   0.948611   5.273446   1.004700   5.298234
                                                -----------------------------------------------------------------------
```

2.5 Counterpoise calculations to investigate reaction regioselectivity

Further to the potential energy surface calculations described in manuscript **Scheme 10**, additional calculations were carried out to assess the possible original of the calculated and observed regioselectivity for the production of 3,5- over 3,4-isomers of the isoxazolines.

Deconstructed binding energy calculations required three separate calculations per oximyl radical-acrylate pair: the oximyl radical-acrylate transition state complex, the oximyl radical fragment, and the acrylate fragment. This generalised in **Scheme S1** (left). **NOTE** – following DFT optimization of relevant fragments and complexes in each oximyl radical-acrylate pair, only the electronic energies (not enthalpies or free energies) were required for the follow-up Boys-Bernardi counterpoise analysis, described in detail in the references provided. [16,17]

The key calculation is to produce the counterpoise corrected electronic energy for the oximyl radical-acrylate transition state complex. This was performed in Gaussian09 using the keyword *counterpoise=2* in a separate calculation *after* the gas phase transition state complex had already been formally optimized. **NOTE** – to allow for this calculation, the given oximyl radical-acrylate complex was split into two *atom groups* using *GaussView* visualization software.

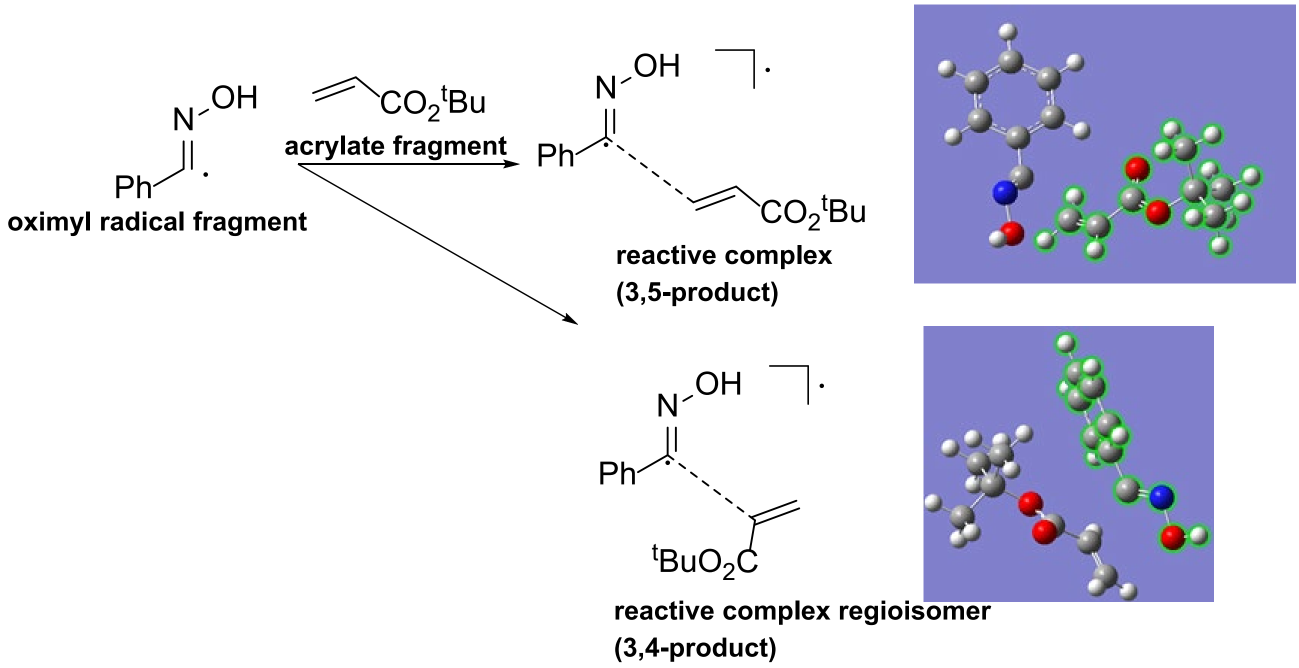

The equation below represents the key electronic energy values calculated:

$$\boldsymbol{E_{bind}} = \underbrace{\left[E_{AB}^{\alpha\beta}(AB) - E_{AB}^{\alpha\beta}(A) - E_{AB}^{\alpha\beta}(B)\right]}_{\boldsymbol{E_{int}}} + \underbrace{[E_{AB}^{\alpha}(A) - E_{A}^{\alpha}(A)] + \left[E_{AB}^{\beta}(B) - E_{B}^{\beta}(B)\right]}_{\boldsymbol{E_{dist}}}$$

key: $$Energy_{geometry}^{basis\ set}(fragment)$$

When comparing the results for the 3,5-isomer (blue) versus the 3,4-isomer (red), the counterpoise calculated 'binding' energy of the fragments in the transition state geometries revealed a dominant distortion term for the 3,4-isomer, rendering the overall complexation energy positive. The more favourable interaction energy and low distortion of the fragments en route to the 3,5-isomer resulted in an overall favourable (negative) binding energy for the graments. These data remain consistent with the calculated pathways (scheme 10) and experimental findings.

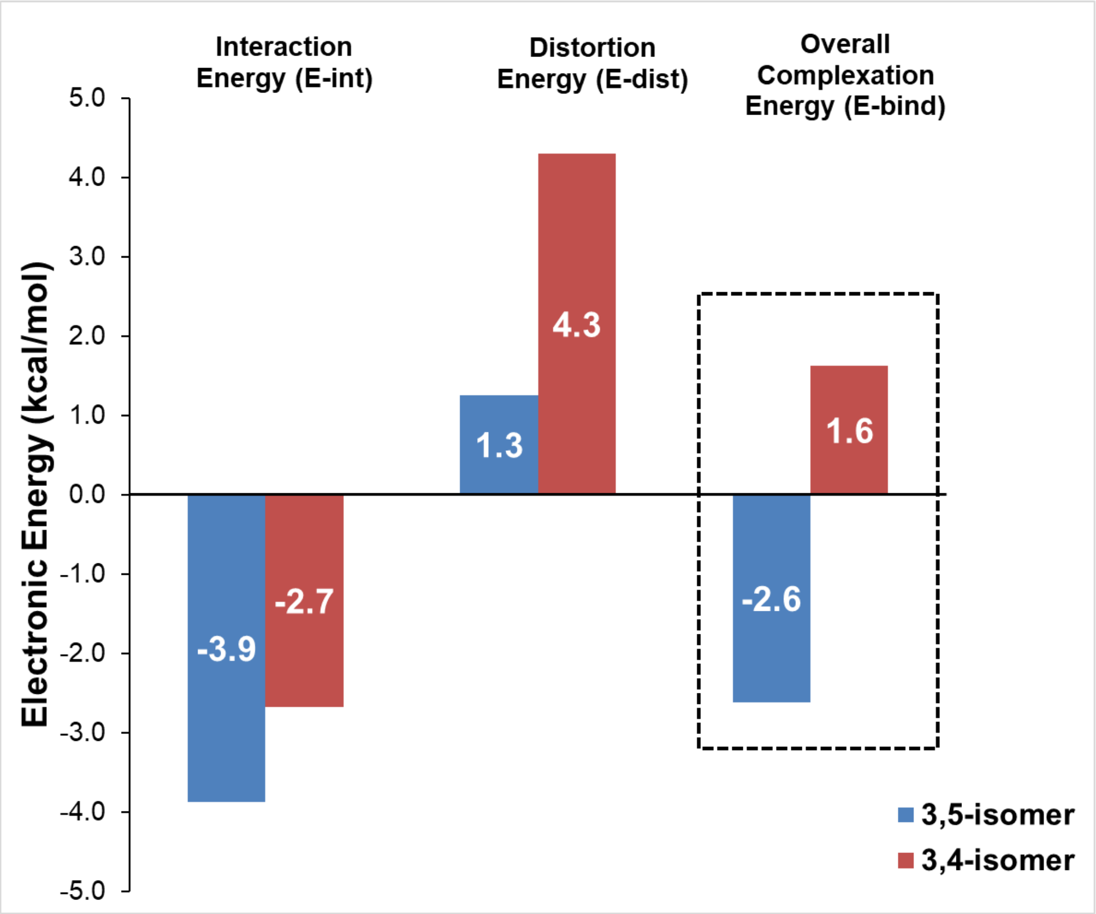

Analysing the 3D geometries in combination with Natural Bond Order (NBO) analysis outputs from the calculations reveal the following with regards to the original of the 3,5 over 3,4 isomer selectivity:

- In the $S_{RN}2$ transition state, ompared to the 3,5-isomer, the 3,4-isomer shows a steric clash between the acrylate tert-butyl group and the oximyl radical phenyl group.
- The above clash results in the alkene and carbonyl group on the acrylate being forced out of full conjugation. This is evident in the higher C=O stretching frequency in the 3,4-isomer (1837 cm^{-1}) versus the 3,5-isomer (1798 cm^{-1}).
- Consistent with the above two points, the C-C-C-O acrylate dihedral in the 3,5-isomer transition state in 4 degrees. In the more distorted 3,4-isomer, the same dihedral is 26 degrees.
- Regarding the favourable interactions, NBO calculations on each isomer's transition state reveal the effectiveness of orbital overlap from from the unpaired electron on the oximyl radical to the C-C pi-star orbital on the acrylate:
    - From oximyl radical to acrylate in 3,4-isomer
        - 60. LP (1) C 12 /414. BD*(2) C 16 - C 35 1.37 0.42 0.032
        - 60. LP (1) C 12 /412. BD*(2) C 16 - C 17 17.87 0.39 0.109

2.6 Additional Calculations with Solvent-inclusive Optimisations

2.6.1

Selected barriers were recomputed to include solvation effects and provide a quality control measure for our broader suite of single-point corrected gas phase calculations.

We calculated solvent-optimised geometries and frequency calculations for:

1. Fumerate and maleate [3+2] cycloadditon steps.
2. t-Butylacrylate + N-phenyl nitrile oxide [3+2] cycloaddition step.
3. t-Butylacrylate + Z-phenylaldoxime $S_{RN}2$ radical step.
4. Fumerate + maleate $S_{RN}2$ radical steps.

All calculations were performed at the (U)M06-2X/Def2TZVP/SMD(MeCN) level.

In all cases, the interpretation of the comparisons between energy profiles of the radical versus closed shell [3+2] potential energy surfaces remained unchanged from those reported in our revised manuscript. In the figure below, the values outside parenthesis represent recalculated barriers via solvent-inclusive optimisations. The values in parenthesis are the originally calculated values using single point corrections, detailed above, and used more broadly in the computational section of the paper.

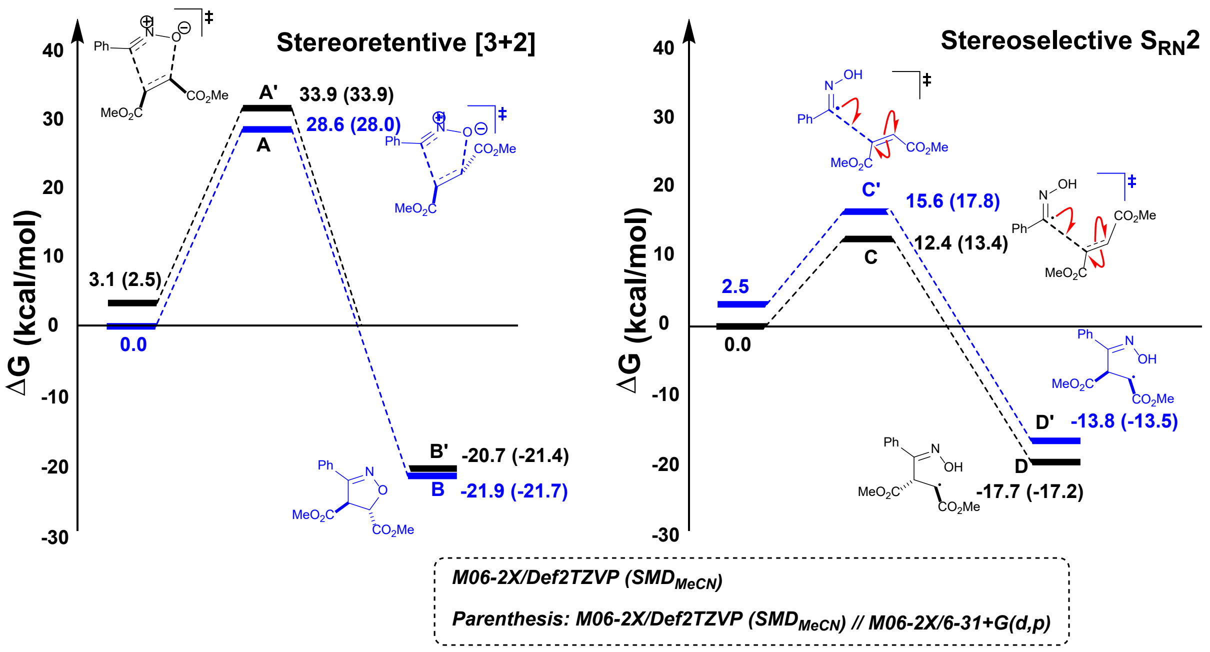

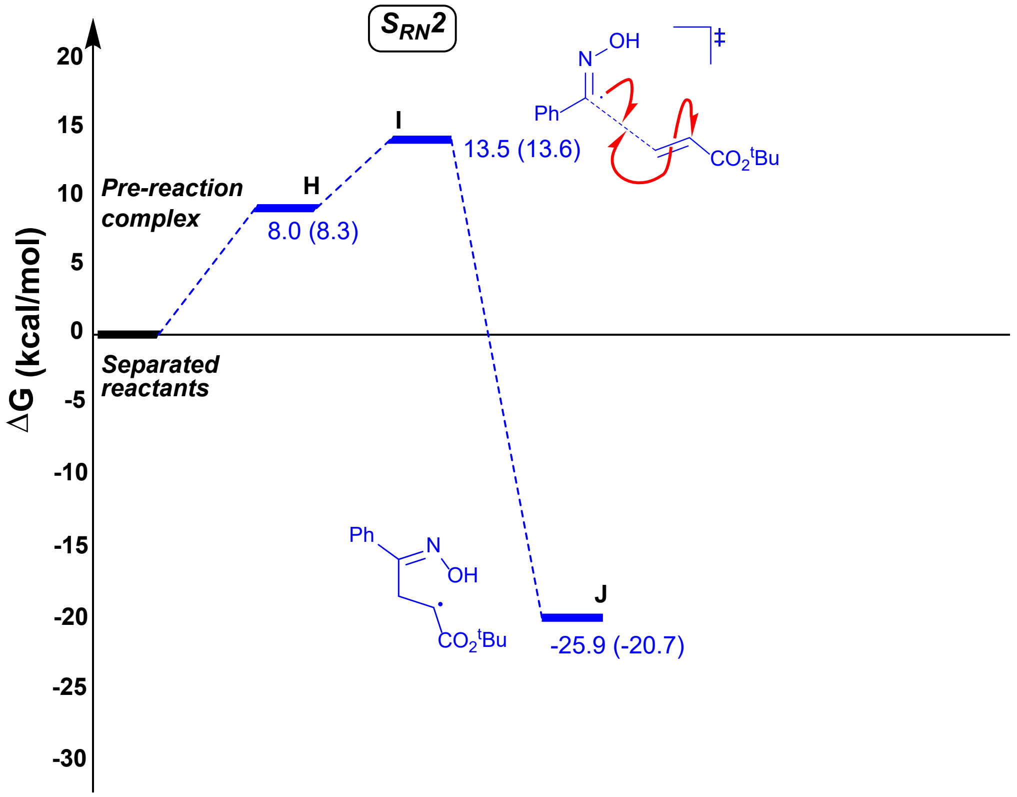

Cl-mediated HAT barrier analysis

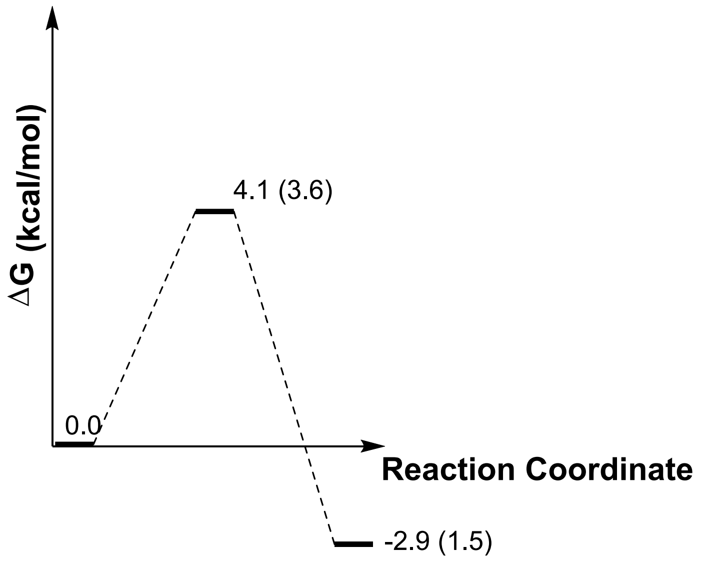

UM06-2X/Def2TZVP (SMD_{MeCN})
Parenthesis: UM06-2X/Def2TZVP (SMD_{MeCN}) // UM06-2X/6-31+G(d,p)

The additional solvent calculations facilitated exploration of the energetics in the formation of key proposed intermediates, namely the nitrile oxide (for the [3+2] path) and the oximyl radical (for the $S_{RN}2$) pathway.

While the elementary steps leading to the oximyl radical and nitrile oxide were computed to be comparable (4.1 versus 3.2 kcal/mol), the [3+2] pathway is computed to be disfavoured by the large barrier (181 kcal/mol) leading to the N-chlorinated oximinium cation that serves as the precursor to an E2 elimination step (facilitated by HFIP alkoxide anion produced at the cathode) that, in turn, produces the nitrile oxide.

These data remain consistent with the proposed radical pathway dominating over the originally hypothesised [3+2] pathway.

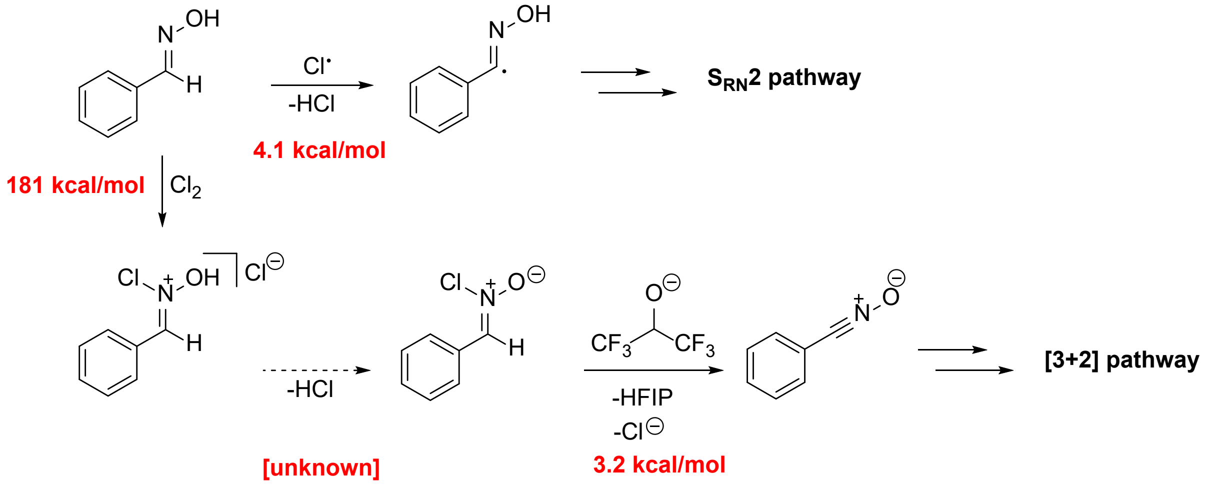

2.7 Output Coordinates and thermal data

Original calculation filenames are maintained for ease of sources raw data on request.

dipolarophile-isomer-1

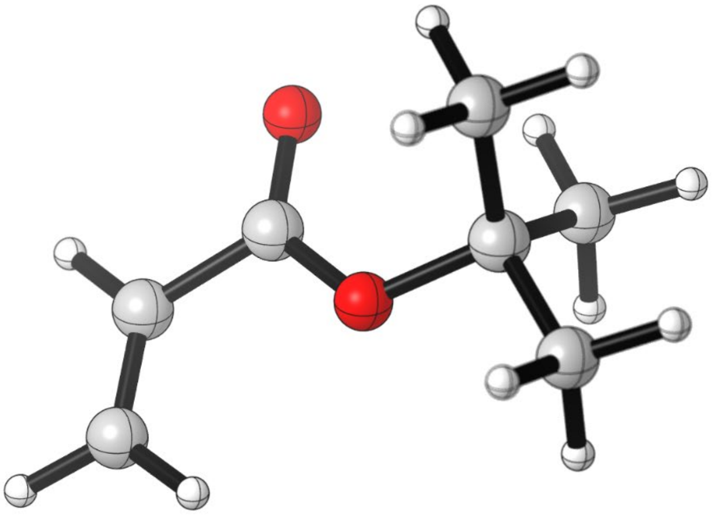

Sum of Electronic and Zero-point Energies = -424.066850 Hartree
Sum of Electronic and Thermal Energies = -424.056464 Hartree
Sum of Electronic and Thermal Enthalpies = -424.055520 Hartree
Sum of Electronic and Thermal Free Energies = -424.102075 Hartree

Dipole Moment = 2.2666 Debye

0 1

C	-2.25966	0.22038	0.03172
H	-1.62966	-0.66314	0.03342
H	-3.33570	0.08082	0.03172
C	-1.72695	1.44113	0.02971
H	-2.33372	2.34093	0.02799
C	-0.26230	1.71037	0.02959
O	0.18938	2.83496	0.02766
O	0.46769	0.58742	0.03181
C	1.92667	0.63724	0.03227
C	2.42782	1.32637	1.29901
H	3.51499	1.21686	1.35850
H	2.18047	2.38830	1.29933
H	1.98752	0.85389	2.18236
C	2.31422	-0.83725	0.03504
H	1.91695	-1.33732	-0.85266
H	3.40312	-0.93660	0.03569
H	1.91618	-1.33418	0.92417
C	2.42882	1.32181	-1.23654
H	3.51600	1.21179	-1.29495
H	1.98897	0.84638	-2.11853
H	2.18180	2.38380	-1.24075

dipolarophile-isomer-2

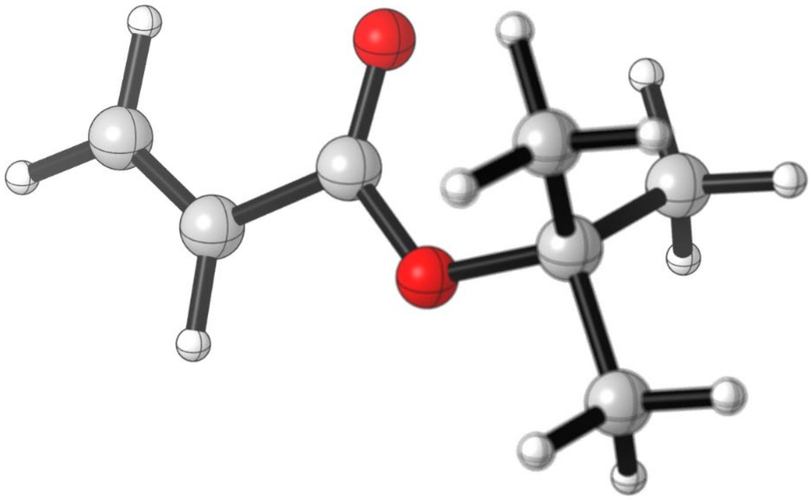

Sum of Electronic and Zero-point Energies = -424.067676 Hartree
Sum of Electronic and Thermal Energies = -424.057295 Hartree
Sum of Electronic and Thermal Enthalpies = -424.056351 Hartree
Sum of Electronic and Thermal Free Energies = -424.102849 Hartree

Dipole Moment = 1.5642 Debye

0 1

C	-2.52964	0.48180	0.03374
H	-2.17292	-0.54426	0.03556
H	-3.59973	0.65821	0.03321
C	-1.65816	1.48801	0.03220
H	-1.95979	2.53019	0.03034
C	-0.19412	1.20630	0.03295
O	0.28884	0.09566	0.03503
O	0.50188	2.35191	0.03098
C	1.96165	2.34754	0.03105
C	2.48368	1.67334	-1.23539
H	3.56720	1.81474	-1.29399
H	2.26740	0.60467	-1.23583
H	2.03043	2.13281	-2.11905
C	2.30449	3.83302	0.02840
H	1.89144	4.32092	0.91553
H	3.38999	3.96468	0.02821
H	1.89152	4.31776	-0.86050
C	2.48362	1.67791	1.29993
H	3.56711	1.81968	1.35814
H	2.03021	2.14044	2.18190
H	2.26751	0.60923	1.30414

p-Cl-TS-endo_SM-min-ii

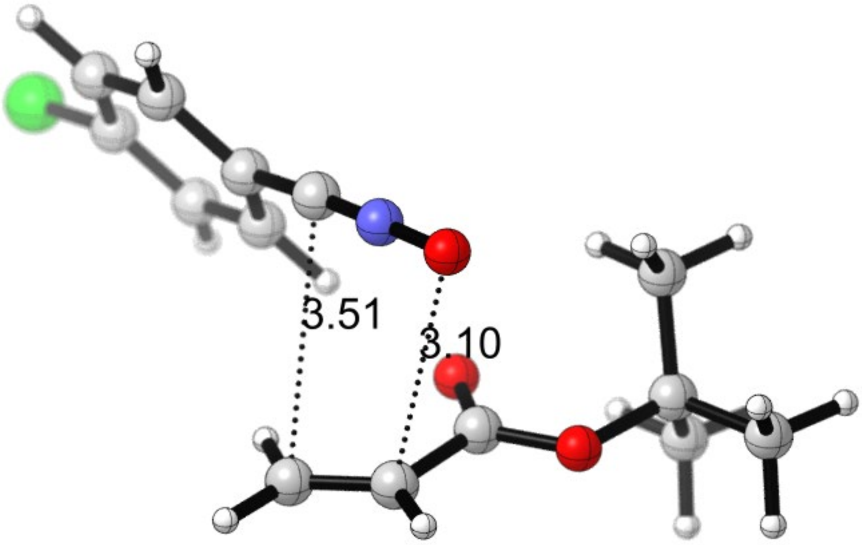


```
Sum of Electronic and Zero-point Energies = -1283.037076 Hartree
Sum of Electronic and Thermal Energies = -1283.016522 Hartree
Sum of Electronic and Thermal Enthalpies = -1283.015578 Hartree
Sum of Electronic and Thermal Free Energies = -1283.089136 Hartree

Dipole Moment = 0.9233 Debye

0 1
O                -0.98961      2.91287     -0.21723
N                -1.19393      1.73970      0.02785
C                -1.37228      0.62226      0.27149
C                -1.43838     -0.78940      0.51848
C                -0.31310     -1.58299      0.25056
C                -2.61062     -1.37081      1.01800
C                -0.36579     -2.95184      0.48586
H                 0.58506     -1.11101     -0.13646
C                -2.66122     -2.73940      1.25237
H                -3.47683     -0.75042      1.22092
C                -1.53746     -3.51726      0.98374
H                 0.49473     -3.57985      0.28387
H                -3.56174     -3.20310      1.63873
C                 1.81003      2.01512     -0.19528
O                 1.60842      0.88308     -0.59447
O                 2.15977      3.04691     -0.96023
C                 2.23563      2.92180     -2.41418
C                 0.87812      2.50395     -2.97460
H                 0.90086      2.60117     -4.06447
H                 0.64205      1.46958     -2.71980
H                 0.09339      3.15323     -2.57686
C                 2.58238      4.33928     -2.85488
H                 3.52676      4.65788     -2.40492
H                 2.68136      4.37626     -3.94321
H                 1.79618      5.03421     -2.54816
C                 3.34993      1.95051     -2.79447
```

H	3.48553	1.97005	-3.88022
H	4.29090	2.25460	-2.32593
H	3.10914	0.93200	-2.48804
C	1.44152	1.51471	2.17150
H	1.31911	0.46831	1.90302
H	1.34526	1.79028	3.21593
C	1.70293	2.42267	1.23364
H	1.82894	3.47749	1.45161
Cl	-1.60094	-5.23136	1.27574

p-CN-TS-endo_SM-min-ii

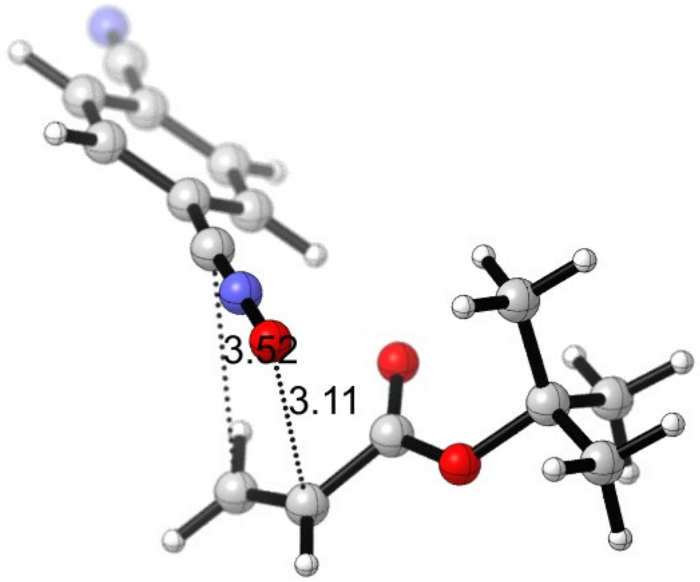

Sum of Electronic and Zero-point Energies = -915.674957 Hartree
Sum of Electronic and Thermal Energies = -915.653789 Hartree
Sum of Electronic and Thermal Enthalpies = -915.652845 Hartree
Sum of Electronic and Thermal Free Energies = -915.727474 Hartree

Dipole Moment = 2.3857 Debye

0 1

O	-1.01628	2.81885	-0.04407
N	-1.25776	1.65567	0.19951
C	-1.47431	0.54423	0.44154
C	-1.58376	-0.86569	0.68052
C	-0.49152	-1.69338	0.37693
C	-2.76555	-1.40495	1.20711
C	-0.58838	-3.05909	0.60549
H	0.41058	-1.24673	-0.02998
C	-2.85719	-2.77064	1.43391
H	-3.60118	-0.75255	1.43477
C	-1.76835	-3.59785	1.13303
H	0.24560	-3.71348	0.37666

H	-3.76478	-3.20197	1.84135
C	1.75042	1.81423	-0.11002
O	1.47894	0.69578	-0.50722
O	2.12600	2.83153	-0.88068
C	2.15562	2.71198	-2.33751
C	0.76330	2.36810	-2.86123
H	0.76098	2.46877	-3.95095
H	0.48070	1.34622	-2.60359
H	0.02475	3.05613	-2.44086
C	2.56252	4.11263	-2.78025
H	3.53462	4.37902	-2.35626
H	2.63214	4.15176	-3.87073
H	1.82252	4.84532	-2.44755
C	3.20733	1.68727	-2.75350
H	3.31548	1.70904	-3.84222
H	4.17478	1.93821	-2.30812
H	2.92227	0.67985	-2.44853
C	1.43911	1.31816	2.26655
H	1.26301	0.27916	1.99920
H	1.39168	1.59216	3.31471
C	1.70850	2.21826	1.32338
H	1.89031	3.26495	1.54126
C	-1.86414	-5.01546	1.36928
N	-1.94214	-6.15441	1.56022

p-F-TS-endo_SM-min-iii

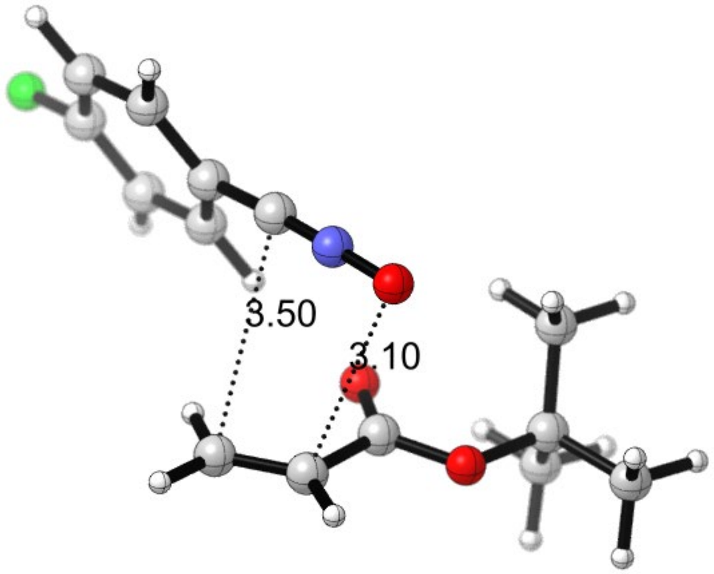

Sum of Electronic and Zero-point Energies = -922.678897 Hartree
Sum of Electronic and Thermal Energies = -922.658701 Hartree
Sum of Electronic and Thermal Enthalpies = -922.657756 Hartree
Sum of Electronic and Thermal Free Energies = -922.730087 Hartree

Dipole Moment = 1.1266 Debye

0 1

O	-1.11829	2.57958	0.03535
N	-1.33261	1.40403	0.26426
C	-1.51933	0.28473	0.49211
C	-1.59774	-1.13054	0.71492
C	-0.49469	-1.93431	0.38632
C	-2.75892	-1.70335	1.25119
C	-0.55632	-3.30669	0.59798
H	0.39325	-1.46549	-0.02711
C	-2.82256	-3.07529	1.46332
H	-3.60532	-1.07237	1.49951
C	-1.71804	-3.84706	1.13128
H	0.27617	-3.95745	0.35598
H	-3.70515	-3.54955	1.87663
C	1.66884	1.63749	-0.05118
O	1.43771	0.51839	-0.47030
O	2.01165	2.68182	-0.80224
C	2.03919	2.59092	-2.26036
C	0.65729	2.20987	-2.78624
H	0.64692	2.33265	-3.87373
H	0.41107	1.17394	-2.54799
H	-0.10255	2.86255	-2.34792
C	2.39654	4.01273	-2.67805
H	3.36047	4.30426	-2.25180
H	2.46081	4.07485	-3.76784
H	1.63302	4.71300	-2.32908
C	3.12356	1.61068	-2.69971
H	3.22499	1.65492	-3.78846
H	4.08415	1.88712	-2.25444
H	2.87467	0.58908	-2.41047
C	1.36288	1.08705	2.31333
H	1.21431	0.04954	2.02438
H	1.30245	1.33936	3.36629
C	1.61149	2.01265	1.38936
H	1.76163	3.05984	1.62779
F	-1.77710	-5.17445	1.33577

p-H-TS-endo-IRC-regioisomer-SM-min

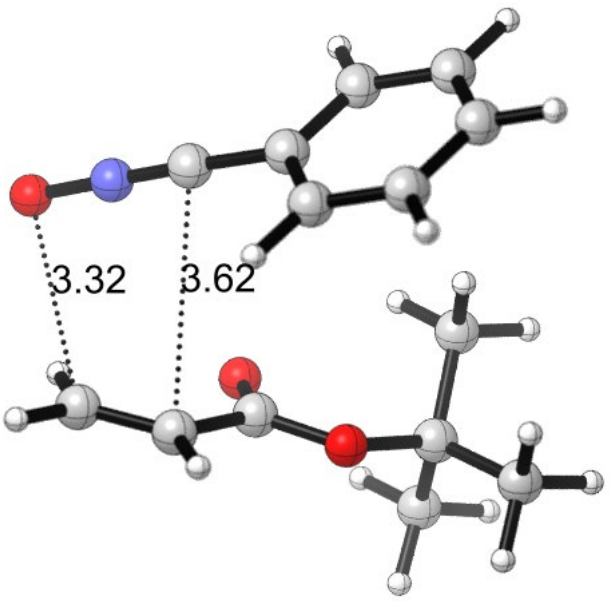


```
Sum of Electronic and Zero-point Energies = -823.457448 Hartree
Sum of Electronic and Thermal Energies = -823.438174 Hartree
Sum of Electronic and Thermal Enthalpies = -823.437230 Hartree
Sum of Electronic and Thermal Free Energies = -823.506419 Hartree

Dipole Moment = 5.7279 Debye

0 1
O                -0.55194      3.74421     -1.55982
N                -0.74323      2.54525     -1.58214
C                -0.91511      1.40050     -1.60253
C                -1.08027     -0.02457     -1.61582
C                 0.04028     -0.85361     -1.76695
C                -2.35824     -0.58628     -1.48633
C                -0.12290     -2.23527     -1.78917
H                 1.02428     -0.40710     -1.86469
C                -2.50873     -1.96852     -1.51342
H                -3.21791      0.06386     -1.36461
C                -1.39447     -2.79474     -1.66605
H                 0.74633     -2.87473     -1.90333
H                -3.49846     -2.40180     -1.41352
H                -1.51751     -3.87278     -1.68651
C                 1.18233      0.81058      0.99447
O                 0.24899      1.52379      1.29082
O                 1.37188     -0.43933      1.44169
C                 0.45410     -1.03974      2.40589
C                -0.95065     -1.13890      1.81720
H                -1.58155     -1.71510      2.50158
H                -1.39470     -0.15330      1.67202
H                -0.91859     -1.66347      0.85757
C                 1.04360     -2.43050      2.61082
H                 2.06735     -2.36003      2.98855
H                 0.44007     -2.98880      3.33177
H                 1.05630     -2.97841      1.66396
```

C	0.48142	-0.24247	3.70754
H	-0.09439	-0.77877	4.46798
H	1.51062	-0.14106	4.06510
H	0.04825	0.74958	3.57388
C	2.29217	2.42063	-0.47941
H	1.49396	3.13003	-0.27382
H	3.08859	2.72835	-1.14899
C	2.28558	1.20845	0.07456
H	3.06512	0.47396	-0.10367

p-H-TS-endo-IRC-SM-min-freq

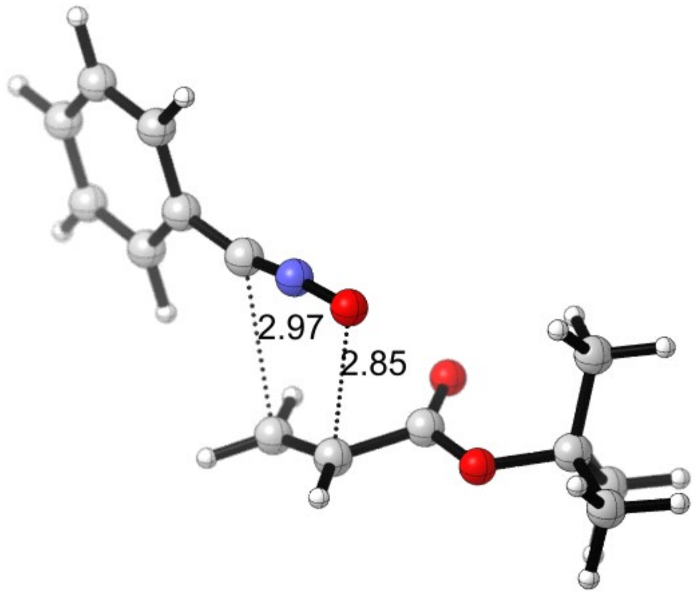

Sum of Electronic and Zero-point Energies = -823.450577 Hartree
Sum of Electronic and Thermal Energies = -823.432928 Hartree
Sum of Electronic and Thermal Enthalpies = -823.431984 Hartree
Sum of Electronic and Thermal Free Energies = -823.498043 Hartree

Dipole Moment = 3.8788 Debye

0 1

O	-1.29683	1.36521	-0.18798
N	-1.42203	0.26257	0.32150
C	-1.42694	-0.75588	0.84382
C	-1.77251	-2.04895	1.37868
C	-0.85136	-2.82739	2.08767
C	-3.07816	-2.51859	1.16759
C	-1.23793	-4.07152	2.57762
H	0.15334	-2.45134	2.24202
C	-3.45227	-3.76109	1.66203
H	-3.78915	-1.91278	0.61496
C	-2.53397	-4.53911	2.36610
H	-0.52092	-4.67376	3.12587

H	-4.46024	-4.13060	1.50061
H	-2.82536	-5.51056	2.75275
C	1.54954	1.21169	-0.51274
O	1.65897	0.22632	-1.21098
O	1.66488	2.47643	-0.92723
C	1.97590	2.79197	-2.31481
C	0.87896	2.26112	-3.23448
H	1.01182	2.68173	-4.23643
H	0.90580	1.17225	-3.29571
H	-0.10114	2.56685	-2.85371
C	1.99067	4.31623	-2.32004
H	2.75014	4.69512	-1.63058
H	2.21573	4.68115	-3.32675
H	1.01502	4.70547	-2.01563
C	3.35297	2.23868	-2.67224
H	3.59954	2.53482	-3.70045
H	4.11528	2.65360	-2.00126
H	3.36578	1.14755	-2.61032
C	1.33658	0.01537	1.60795
H	1.54242	-0.90720	1.05508
H	1.20277	-0.03447	2.68478
C	1.30932	1.17588	0.95818
H	1.13687	2.12555	1.45245

p-Me-TS-endo-IRC-SM-min-iv-from_H

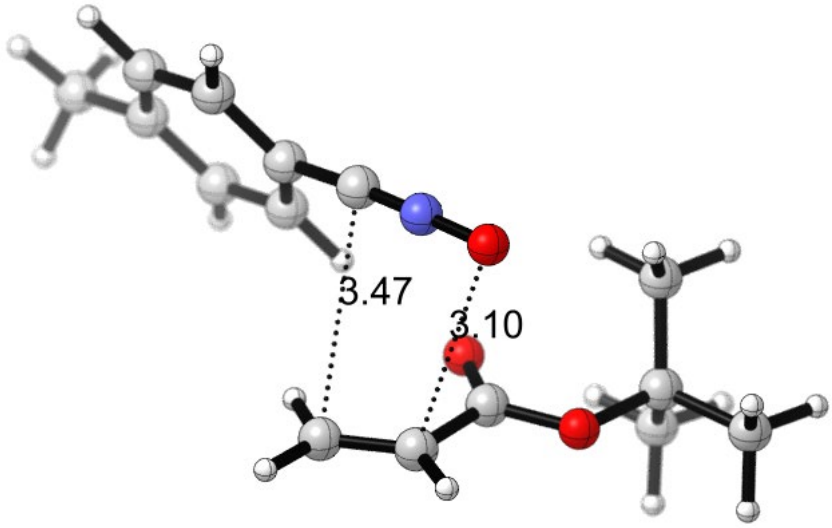

Sum of Electronic and Zero-point Energies = -862.731639 Hartree
Sum of Electronic and Thermal Energies = -862.710348 Hartree
Sum of Electronic and Thermal Enthalpies = -862.709404 Hartree
Sum of Electronic and Thermal Free Energies = -862.784738 Hartree

Dipole Moment = 3.5153 Debye

0 1

O	-1.40557	2.13399	-0.01043
N	-1.60965	0.96194	0.25106
C	-1.78603	-0.15161	0.51250
C	-1.84424	-1.55805	0.79319
C	-0.70630	-2.34807	0.56576
C	-3.01489	-2.14153	1.28926
C	-0.75749	-3.70820	0.84044
H	0.19386	-1.87619	0.18299
C	-3.04341	-3.50631	1.55739
H	-3.89127	-1.52622	1.46375
C	-1.92100	-4.30952	1.33739
H	0.12467	-4.31882	0.66556
H	-3.95423	-3.95429	1.94460
C	1.39765	1.28781	0.17583
O	1.23748	0.15767	-0.24511
O	1.77897	2.33310	-0.55692
C	1.94385	2.22430	-2.00389
C	0.62872	1.79587	-2.65052
H	0.71510	1.90997	-3.73564
H	0.39326	0.75485	-2.42470
H	-0.18814	2.42700	-2.29023
C	2.29822	3.65066	-2.40894
H	3.20953	3.97620	-1.89952
H	2.46203	3.70075	-3.48894
H	1.48585	4.33225	-2.14315
C	3.09179	1.27129	-2.32652
H	3.29238	1.30484	-3.40188
H	3.99883	1.58153	-1.79868
H	2.84586	0.24660	-2.04585
C	0.89609	0.75894	2.51134
H	0.80193	-0.28583	2.22539
H	0.73361	1.02276	3.55064
C	1.20085	1.67985	1.59956
H	1.29935	2.73400	1.83437
C	-1.95502	-5.79056	1.61250
H	-2.87020	-6.07616	2.13570
H	-1.90876	-6.35896	0.67801
H	-1.10147	-6.09503	2.22491

p-NO2-TS-endo_SM-min-ii

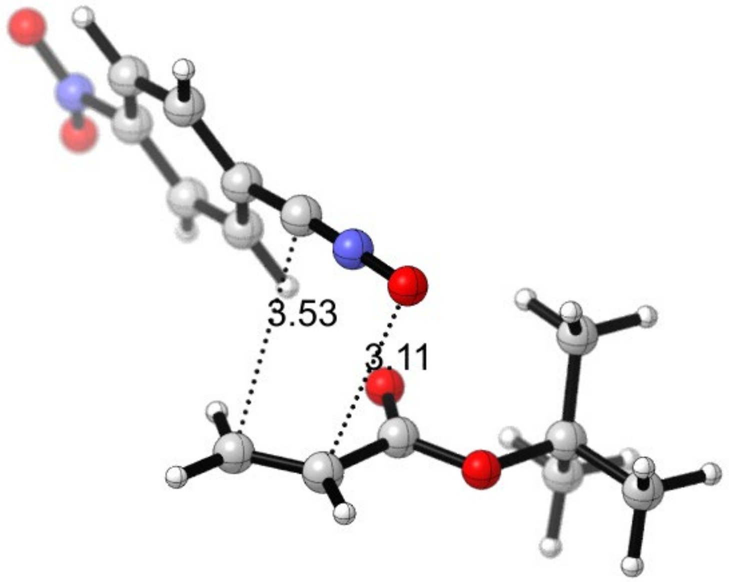

Sum of Electronic and Zero-point Energies = -1027.887704 Hartree
Sum of Electronic and Thermal Energies = -1027.865837 Hartree
Sum of Electronic and Thermal Enthalpies = -1027.864892 Hartree
Sum of Electronic and Thermal Free Energies = -1027.941780 Hartree

Dipole Moment = 2.7738 Debye

0 1

O	-0.82968	3.19379	-0.19783
N	-1.08078	2.03548	0.05420
C	-1.30672	0.92759	0.30408
C	-1.42979	-0.47931	0.55287
C	-0.34325	-1.31825	0.25760
C	-2.61958	-1.00153	1.08030
C	-0.45107	-2.68250	0.49581
H	0.56364	-0.88258	-0.15022
C	-2.72787	-2.36487	1.31829
H	-3.44879	-0.33878	1.30050
C	-1.63964	-3.17604	1.02023
H	0.36507	-3.36162	0.28136
H	-3.63095	-2.80276	1.72523
C	1.92835	2.16169	-0.26265
O	1.64096	1.04288	-0.64772
O	2.31353	3.16637	-1.04465
C	2.33774	3.03163	-2.50050
C	0.93958	2.70050	-3.01676
H	0.93566	2.78880	-4.10753
H	0.64419	1.68539	-2.74695
H	0.21133	3.40320	-2.60268
C	2.76152	4.42229	-2.95850
H	3.73845	4.68006	-2.54035
H	2.82804	4.44953	-4.04952
H	2.03234	5.16802	-2.63090
C	3.37484	1.98913	-2.90876

H	3.48105	1.99938	-3.99783
H	4.34646	2.23132	-2.46767
H	3.07719	0.98839	-2.59400
C	1.62179	1.69237	2.12005
H	1.43408	0.65260	1.86382
H	1.58212	1.97704	3.16567
C	1.89662	2.58021	1.16686
H	2.09101	3.62687	1.37379
N	-1.75229	-4.62514	1.27306
O	-2.80434	-5.03551	1.72867
O	-0.78646	-5.31782	1.01020

p-OMe-TS-endo_SM-min-iii

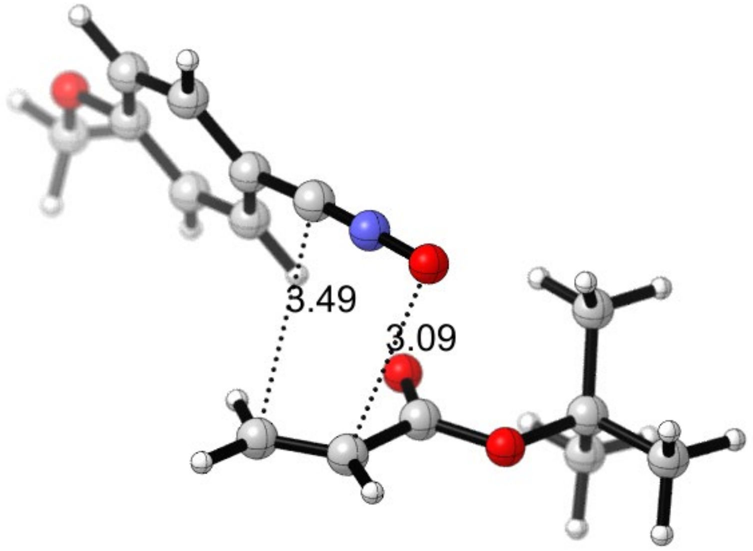

Sum of Electronic and Zero-point Energies = -937.910606 Hartree
Sum of Electronic and Thermal Energies = -937.888703 Hartree
Sum of Electronic and Thermal Enthalpies = -937.887759 Hartree
Sum of Electronic and Thermal Free Energies = -937.963708 Hartree

Dipole Moment = 3.9567 Debye

0 1

O	-1.13043	2.88080	-0.28209
N	-1.36963	1.74313	0.08628
C	-1.57633	0.66300	0.44696
C	-1.68537	-0.70918	0.84804
C	-0.59028	-1.56239	0.67620
C	-2.87465	-1.20512	1.40827
C	-0.67530	-2.89843	1.06065
H	0.32245	-1.16458	0.24232
C	-2.96030	-2.53110	1.78999
H	-3.72397	-0.54313	1.53986
C	-1.86153	-3.38545	1.61834

H	0.18385	-3.54235	0.91877
H	-3.86742	-2.93651	2.22437
C	1.64356	1.91507	-0.13018
O	1.43567	0.75076	-0.41631
O	2.01565	2.86149	-0.99050
C	2.09823	2.59210	-2.42349
C	0.74089	2.13097	-2.94950
H	0.77031	2.12310	-4.04360
H	0.49679	1.12750	-2.59745
H	-0.04159	2.81963	-2.61922
C	2.45929	3.95623	-3.00051
H	3.40326	4.31115	-2.57748
H	2.56557	3.88453	-4.08647
H	1.67648	4.68351	-2.76948
C	3.20683	1.57952	-2.69879
H	3.34883	1.49022	-3.78028
H	4.14763	1.92114	-2.25636
H	2.95552	0.59837	-2.29450
C	1.22461	1.66213	2.26669
H	1.08040	0.59743	2.10041
H	1.11484	2.04303	3.27613
C	1.52287	2.46616	1.24849
H	1.66679	3.53501	1.36180
O	-2.04527	-4.66470	2.02279
C	-0.96917	-5.57070	1.87044
H	-1.32684	-6.52652	2.25026
H	-0.68977	-5.67668	0.81607
H	-0.09760	-5.24844	2.45144

p-CF3-TS-exo-IRC-SM-min-ii

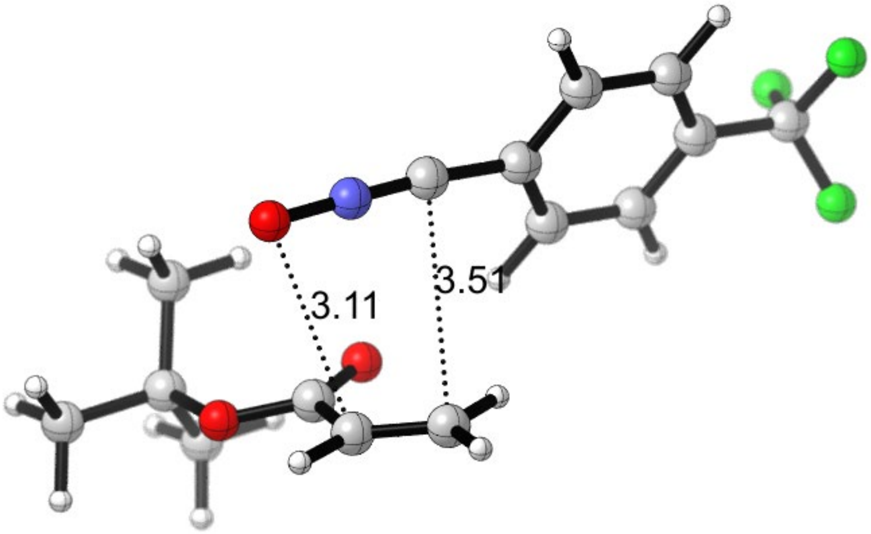

Sum of Electronic and Zero-point Energies = -1160.407996 Hartree

Sum of Electronic and Thermal Energies = -1160.385025 Hartree
Sum of Electronic and Thermal Enthalpies = -1160.384081 Hartree
Sum of Electronic and Thermal Free Energies = -1160.464377 Hartree

Dipole Moment = 0.6908 Debye

0 1

O	-0.05193	2.22148	-2.92755
N	-0.52103	1.50799	-2.06508
C	-0.95631	0.81455	-1.24663
C	-1.33636	-0.05296	-0.16836
C	-0.35460	-0.47281	0.74400
C	-2.66450	-0.47256	-0.03175
C	-0.71553	-1.31506	1.78637
H	0.66797	-0.13301	0.61117
C	-3.01585	-1.31629	1.01659
H	-3.41104	-0.13985	-0.74432
C	-2.04074	-1.73311	1.91821
H	0.03173	-1.65122	2.49841
H	-4.04078	-1.65068	1.13052
C	2.47255	1.06781	-1.94797
O	2.12353	0.93080	-0.78983
O	3.15417	2.10582	-2.42638
C	3.47280	3.25901	-1.58673
C	4.42128	2.84031	-0.46671
H	4.76003	3.73393	0.06642
H	3.92887	2.17373	0.24200
H	5.29872	2.33730	-0.88435
C	4.17196	4.19972	-2.56136
H	3.49826	4.46590	-3.38014
H	4.47546	5.11335	-2.04295
H	5.06147	3.72163	-2.98090
C	2.18777	3.89269	-1.05942
H	2.43254	4.85238	-0.59386
H	1.49043	4.06649	-1.88342
H	1.70460	3.25620	-0.31658
C	1.59422	-1.07858	-2.72971
H	1.36878	-1.81104	-3.49696
H	1.32060	-1.30942	-1.70311
C	2.19196	0.07441	-3.02229
H	2.48212	0.34997	-4.03024
C	-2.39732	-2.62685	3.07433
F	-3.64539	-3.11028	2.97957
F	-2.31282	-1.97313	4.24805
F	-1.56408	-3.67906	3.16104

p-Cl-TS-exo-IRC-SM-min-ii

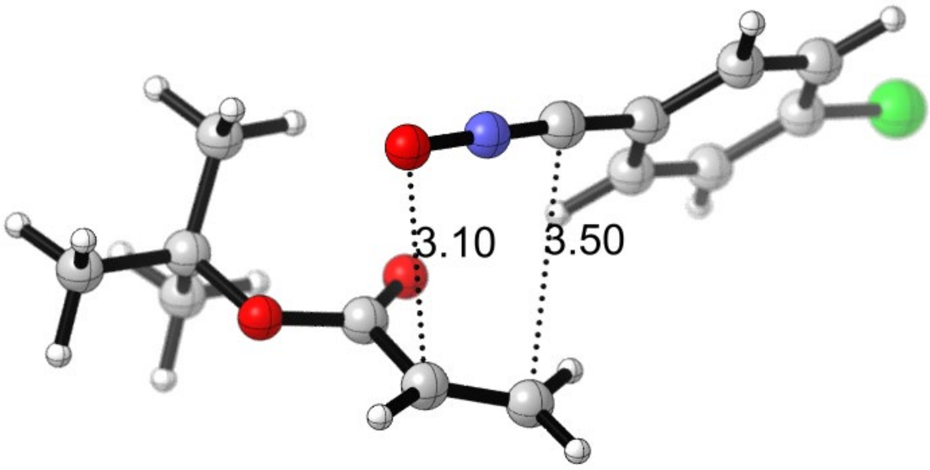

Sum of Electronic and Zero-point Energies = -1283.036995 Hartree
Sum of Electronic and Thermal Energies = -1283.016475 Hartree
Sum of Electronic and Thermal Enthalpies = -1283.015531 Hartree
Sum of Electronic and Thermal Free Energies = -1283.088666 Hartree

Dipole Moment = 0.9235 Debye

0 1

O	-0.49895	1.77812	-2.48118
N	-0.92063	1.06929	-1.58810
C	-1.30953	0.38122	-0.74234
C	-1.62560	-0.47376	0.36556
C	-0.60075	-0.86076	1.24177
C	-2.93803	-0.91633	0.57367
C	-0.89354	-1.68961	2.31829
H	0.40969	-0.50658	1.06062
C	-3.22860	-1.74499	1.65063
H	-3.72500	-0.61125	-0.10749
C	-2.20271	-2.12383	2.51315
H	-0.11340	-1.99826	3.00508
H	-4.23975	-2.09563	1.82366
C	2.08382	0.68220	-1.59189
O	1.79105	0.55546	-0.41728
O	2.72550	1.72507	-2.11411
C	3.05655	2.89567	-1.30446
C	4.06027	2.51141	-0.22068
H	4.40225	3.41871	0.28671
H	3.61184	1.84400	0.51595
H	4.92972	2.02100	-0.66900
C	3.69438	3.83621	-2.32048
H	2.98078	4.07826	-3.11251
H	4.00200	4.76247	-1.82747
H	4.57416	3.36948	-2.77218
C	1.78280	3.51169	-0.73072

H	2.02736	4.48396	-0.29177
H	1.04625	3.65648	-1.52570
H	1.34610	2.87791	0.04258
C	1.20128	-1.48583	-2.30453
H	0.95253	-2.23225	-3.05087
H	0.97216	-1.70336	-1.26422
C	1.77218	-0.33065	-2.63895
H	2.01578	-0.06657	-3.66217
Cl	-2.56438	-3.16176	3.86209

p-CN-TS-exo-IRC-SM-min-ii

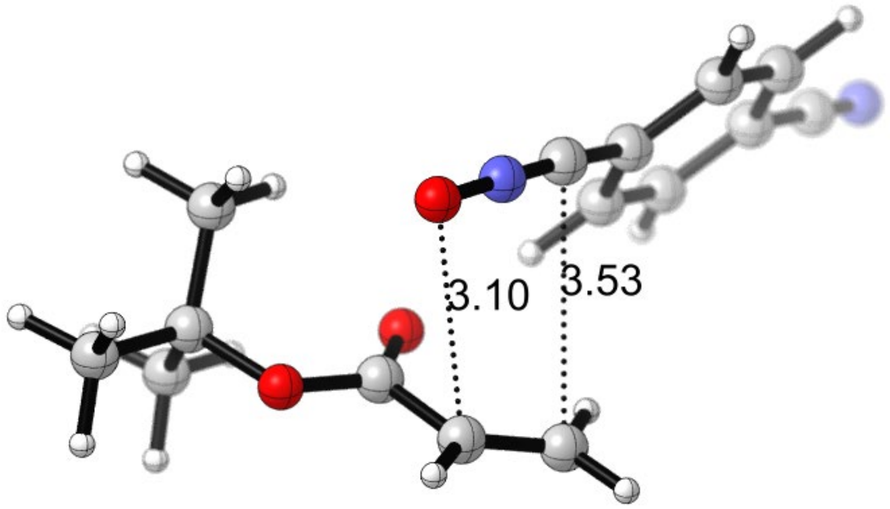

Sum of Electronic and Zero-point Energies = -915.674892 Hartree
Sum of Electronic and Thermal Energies = -915.653751 Hartree
Sum of Electronic and Thermal Enthalpies = -915.652807 Hartree
Sum of Electronic and Thermal Free Energies = -915.727159 Hartree

Dipole Moment = 2.3843 Debye

0 1

O	-0.58687	1.71197	-2.49681
N	-1.01388	1.00506	-1.60886
C	-1.41026	0.31837	-0.76501
C	-1.73762	-0.52921	0.34471
C	-0.72217	-0.89647	1.24163
C	-3.05216	-0.97931	0.52888
C	-1.02940	-1.71635	2.31872
H	0.28670	-0.53230	1.07295
C	-3.35391	-1.79888	1.60702
H	-3.82577	-0.68544	-0.17174
C	-2.34239	-2.16736	2.50209
H	-0.25754	-2.00983	3.02174

H	-4.36624	-2.15518	1.76200
C	1.99761	0.69388	-1.52374
O	1.66371	0.55775	-0.36077
O	2.62822	1.75433	-2.02112
C	2.89981	2.93237	-1.19898
C	3.88028	2.57291	-0.08610
H	4.17765	3.48716	0.43660
H	3.42992	1.88761	0.63278
H	4.77824	2.11243	-0.50908
C	3.54069	3.89375	-2.19320
H	2.84410	4.11967	-3.00492
H	3.80867	4.82600	-1.68859
H	4.44550	3.45286	-2.62058
C	1.59153	3.50933	-0.66339
H	1.79184	4.49206	-0.22556
H	0.87251	3.62511	-1.47901
H	1.15548	2.86686	0.10309
C	1.21101	-1.50095	-2.26834
H	1.01309	-2.25337	-3.02375
H	0.95707	-1.73053	-1.23639
C	1.75301	-0.32609	-2.58171
H	2.02211	-0.05207	-3.59591
C	-2.65650	-3.01854	3.62073
N	-2.90996	-3.70320	4.51892

p-F-TS-exo-IRC-SM-min-ii

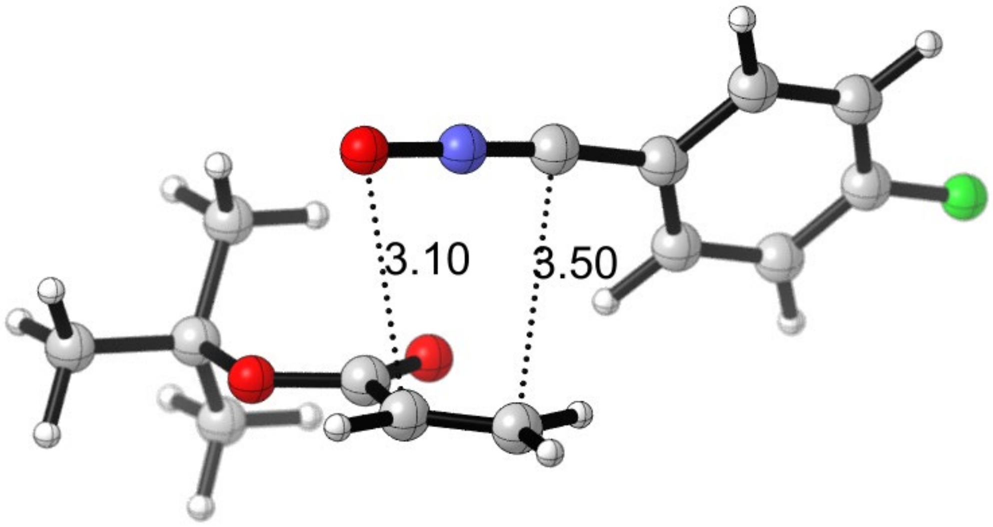

Sum of Electronic and Zero-point Energies = -922.678783 Hartree
Sum of Electronic and Thermal Energies = -922.658635 Hartree
Sum of Electronic and Thermal Enthalpies = -922.657691 Hartree
Sum of Electronic and Thermal Free Energies = -922.729613 Hartree

Dipole Moment = 1.1267 Debye

0 1

O	-0.75314	1.47714	-2.27223
N	-1.16568	0.77309	-1.36987
C	-1.54408	0.08988	-0.51564
C	-1.85108	-0.75541	0.60244
C	-0.82029	-1.12098	1.48250
C	-3.16017	-1.20809	0.81528
C	-1.10215	-1.93964	2.56989
H	0.18556	-0.75780	1.29418
C	-3.44403	-2.02659	1.90202
H	-3.94850	-0.91776	0.12942
C	-2.40696	-2.37416	2.75611
H	-0.33113	-2.24151	3.26952
H	-4.44529	-2.39404	2.09478
C	1.84089	0.42232	-1.37451
O	1.55394	0.30059	-0.19794
O	2.46694	1.46976	-1.90658
C	2.78307	2.65106	-1.10676
C	3.79662	2.28950	-0.02430
H	4.12538	3.20504	0.47705
H	3.36135	1.61884	0.71735
H	4.67246	1.81081	-0.47286
C	3.40252	3.59386	-2.13204
H	2.68171	3.82025	-2.92217
H	3.69913	4.52773	-1.64668
H	4.28674	3.13674	-2.58489
C	1.50239	3.25205	-0.53230
H	1.73330	4.23280	-0.10495
H	0.75924	3.37609	-1.32468
H	1.08031	2.61938	0.24999
C	0.98386	-1.76204	-2.06760
H	0.74235	-2.51734	-2.80735
H	0.75953	-1.97369	-1.02505
C	1.53967	-0.60267	-2.41270
H	1.77719	-0.34360	-3.43862
F	-2.67896	-3.16599	3.80793

p-H-TS-exo-IRC-SM-min-iii

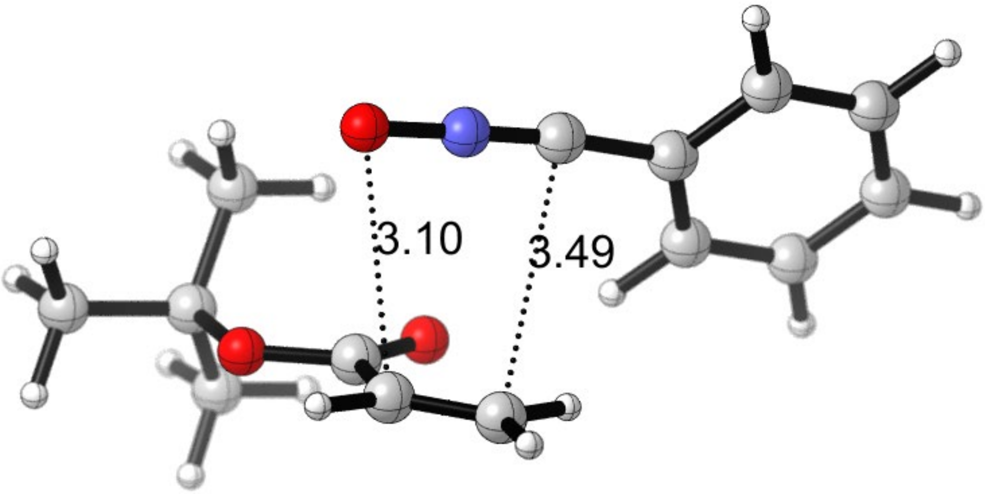

Sum of Electronic and Zero-point Energies = -823.459324 Hartree
Sum of Electronic and Thermal Energies = -823.439988 Hartree
Sum of Electronic and Thermal Enthalpies = -823.439044 Hartree
Sum of Electronic and Thermal Free Energies = -823.508900 Hartree

Dipole Moment = 2.8741 Debye

0 1

O	-0.95149	1.43132	-1.93017
N	-1.40771	0.64550	-1.12047
C	-1.82655	-0.11547	-0.35563
C	-2.19500	-1.07074	0.65141
C	-1.20539	-1.56124	1.51645
C	-3.52214	-1.50432	0.76729
C	-1.55716	-2.48778	2.49300
H	-0.18513	-1.20732	1.40224
C	-3.85679	-2.43097	1.74896
H	-4.27566	-1.11423	0.09137
C	-2.87719	-2.92312	2.61145
H	-0.79580	-2.87017	3.16505
H	-4.88387	-2.76856	1.84077
H	-3.14328	-3.64567	3.37631
C	1.58960	0.20872	-1.10231
O	1.27448	-0.01867	0.05067
O	2.25859	1.28321	-1.51739
C	2.59138	2.36945	-0.59929
C	3.56483	1.87133	0.46587
H	3.91165	2.72323	1.05889
H	3.08914	1.14960	1.13075
H	4.43507	1.40607	-0.00718
C	3.26689	3.38572	-1.51294
H	2.57490	3.71038	-2.29453
H	3.57836	4.25842	-0.93235
H	4.14882	2.94544	-1.98658

C	1.31587	2.95499	0.00191
H	1.56775	3.87662	0.53584
H	0.60088	3.18785	-0.79176
H	0.84928	2.26010	0.70184
C	0.68254	-1.86837	-2.02327
H	0.43389	-2.53907	-2.83840
H	0.43006	-2.17392	-1.01086
C	1.27963	-0.69884	-2.24233
H	1.54588	-0.34764	-3.23319

p-H-TS-exo-regio-isomer-ii-IRC-SM-min

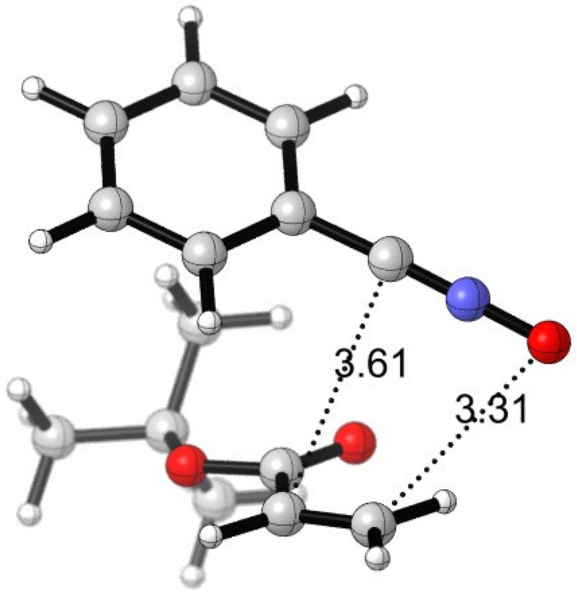

Sum of Electronic and Zero-point Energies = -823.457528 Hartree
Sum of Electronic and Thermal Energies = -823.438205 Hartree
Sum of Electronic and Thermal Enthalpies = -823.437261 Hartree
Sum of Electronic and Thermal Free Energies = -823.506737 Hartree

Dipole Moment = 5.7310 Debye

0 1

O	-2.15245	-2.85300	1.99464
N	-2.09366	-1.84112	1.32592
C	-2.03152	-0.88128	0.68148
C	-1.91732	0.28676	-0.14383
C	-1.33212	0.18748	-1.41410
C	-2.39523	1.52219	0.31566
C	-1.22949	1.32028	-2.21554
H	-0.96366	-0.77327	-1.75853
C	-2.28975	2.64639	-0.49604
H	-2.84164	1.58888	1.30197
C	-1.70920	2.54857	-1.76128
H	-0.77313	1.24156	-3.19689

H	-2.66030	3.60169	-0.13907
H	-1.62915	3.42902	-2.39065
C	1.32963	-1.09213	0.29430
O	1.00403	-0.98640	1.45634
O	1.97856	-0.15668	-0.41442
C	2.40885	1.09298	0.20664
C	3.41924	0.79532	1.31168
H	3.84276	1.73731	1.67319
H	2.94935	0.27737	2.14879
H	4.23520	0.18056	0.91969
C	3.07242	1.83233	-0.94954
H	2.34915	2.00304	-1.75250
H	3.45057	2.79912	-0.60593
H	3.90720	1.24909	-1.34790
C	1.20582	1.88016	0.71964
H	1.54568	2.86466	1.05686
H	0.48099	2.02657	-0.08676
H	0.71839	1.37068	1.55174
C	0.42152	-3.34260	-0.03553
H	0.21994	-4.22492	-0.63404
H	0.08336	-3.33733	0.99802
C	1.06050	-2.29158	-0.54875
H	1.41497	-2.25997	-1.57458

p-Me-TS-exo-IRC-SM-min-ii-freq

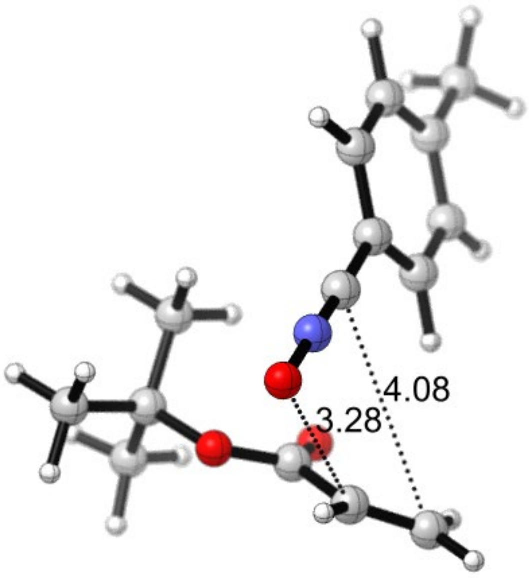

Sum of Electronic and Zero-point Energies = -862.729966 Hartree
Sum of Electronic and Thermal Energies = -862.708613 Hartree
Sum of Electronic and Thermal Enthalpies = -862.707669 Hartree
Sum of Electronic and Thermal Free Energies = -862.785185 Hartree

Dipole Moment = 4.0599 Debye

0 1

O	-0.83263	1.09753	-3.47634
N	-1.10998	0.56493	-2.41906
C	-1.36112	0.04625	-1.41352
C	-1.67076	-0.55865	-0.14898
C	-0.91176	-1.63941	0.31832
C	-2.72237	-0.05613	0.62935
C	-1.20901	-2.20181	1.55381
H	-0.08736	-2.01676	-0.27756
C	-3.00470	-0.63362	1.86091
H	-3.30998	0.77960	0.26361
C	-2.25315	-1.71058	2.34450
H	-0.61330	-3.03657	1.91250
H	-3.82340	-0.24161	2.45825
C	1.89711	-0.21513	-1.34559
O	2.12262	-0.80436	-0.30793
O	1.82685	1.11189	-1.47999
C	2.00772	2.00560	-0.34055
C	3.39846	1.81928	0.26147
H	3.58333	2.62010	0.98399
H	3.49050	0.85757	0.76683
H	4.15751	1.88627	-0.52386
C	1.87330	3.38544	-0.97430
H	0.90700	3.47654	-1.47803
H	1.94859	4.15827	-0.20416
H	2.66426	3.54460	-1.71225
C	0.89856	1.77479	0.68296
H	1.00939	2.49738	1.49742
H	-0.08027	1.93173	0.21899
H	0.93998	0.76645	1.09964
C	1.76176	-2.20807	-2.75565
H	1.57479	-2.72175	-3.69231
H	2.05825	-2.79791	-1.89209
C	1.64296	-0.88538	-2.65359
H	1.34961	-0.24744	-3.48214
C	-2.54254	-2.30611	3.69774
H	-3.60050	-2.21114	3.95411
H	-2.27405	-3.36473	3.72940
H	-1.96462	-1.79343	4.47412

p-OMe-TS-exo-IRC-SM-min-ii

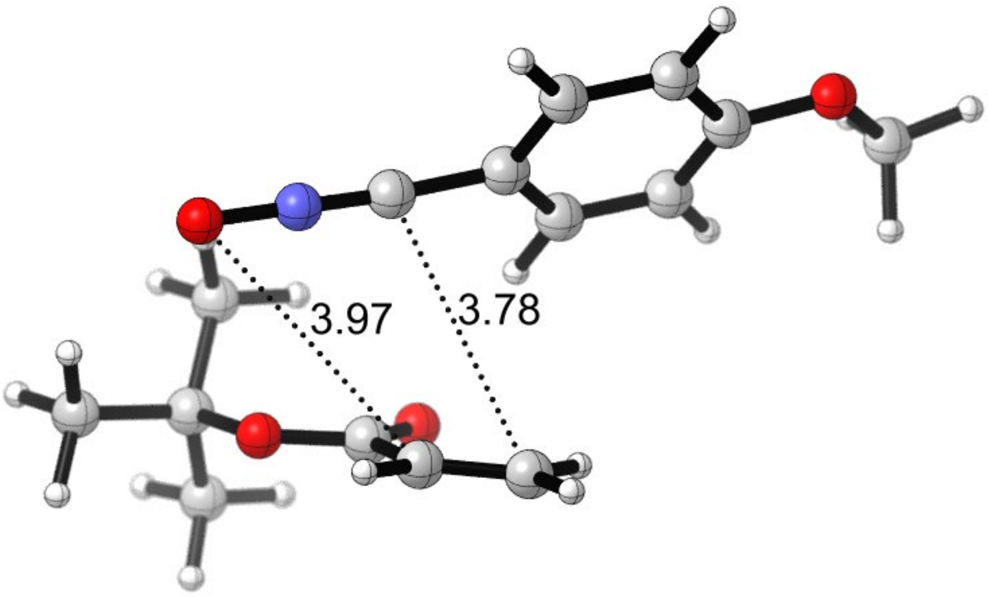


```
Sum of Electronic and Zero-point Energies = -937.910244 Hartree
Sum of Electronic and Thermal Energies = -937.888385 Hartree
Sum of Electronic and Thermal Enthalpies = -937.887441 Hartree
Sum of Electronic and Thermal Free Energies = -937.963045 Hartree

Dipole Moment = 4.2675 Debye

0 1
O              -0.70265      2.72805     -2.14421
N              -0.97572      1.78483     -1.42348
C              -1.21857      0.89350     -0.72276
C              -1.64433     -0.19543      0.10848
C              -0.76184     -0.78092      1.02050
C              -2.96126     -0.67897      0.00765
C              -1.18139     -1.83669      1.82648
H               0.26038     -0.42039      1.08875
C              -3.37929     -1.72691      0.80589
H              -3.64724     -0.22521     -0.69987
C              -2.49196     -2.31180      1.72129
H              -0.47927     -2.27451      2.52526
H              -4.39009     -2.11485      0.74487
C               1.93706      0.26590     -1.13867
O               2.22476     -0.00455      0.01201
O               2.11707      1.44808     -1.72250
C               2.68582      2.58674     -1.00173
C               4.11090      2.25788     -0.56496
H               4.58287      3.16620     -0.17819
H               4.12406      1.49411      0.21373
H               4.69463      1.90815     -1.42193
C               2.67277      3.68765     -2.05508
H               1.65023      3.84937     -2.40573
H               3.05800      4.61630     -1.62441
H               3.30055      3.40811     -2.90595
C               1.79100      2.96721      0.17529
```

H	2.20008	3.86424	0.65055
H	0.78528	3.20213	-0.18374
H	1.74702	2.17016	0.91918
C	1.06792	-1.94944	-1.71626
H	0.60177	-2.66220	-2.38783
H	1.33457	-2.28012	-0.71614
C	1.31258	-0.69566	-2.09203
H	1.06439	-0.31343	-3.07713
O	-2.99607	-3.32959	2.45770
C	-2.14517	-3.95137	3.40230
H	-1.80534	-3.23413	4.15789
H	-2.74318	-4.72670	3.87863
H	-1.27792	-4.40721	2.91131

p-CF3-dipole-iii

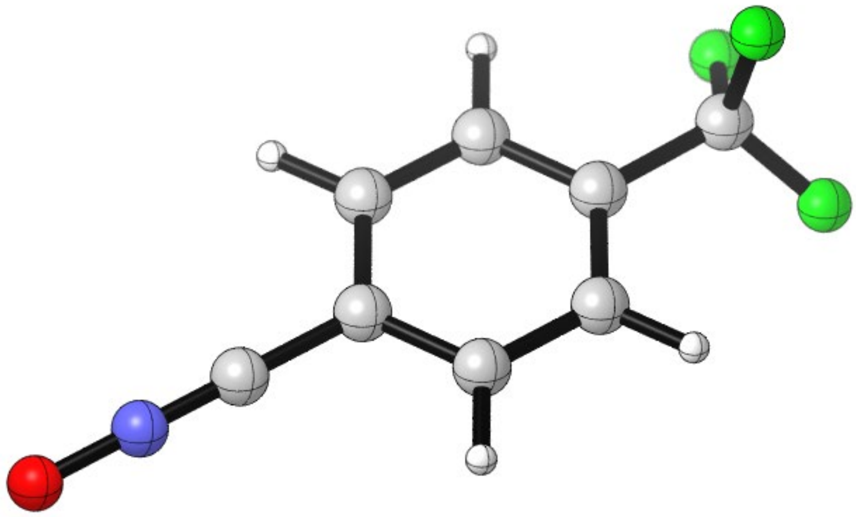

Sum of Electronic and Zero-point Energies = -736.328482 Hartree
Sum of Electronic and Thermal Energies = -736.318690 Hartree
Sum of Electronic and Thermal Enthalpies = -736.317746 Hartree
Sum of Electronic and Thermal Free Energies = -736.365191 Hartree
One immovable negative frequency at -9.35 cm^{-1}

Dipole Moment = 1.7646 Debye

0 1

C	-3.75822	0.27496	-0.01403
C	-2.36944	0.27159	-0.00079
C	-1.67045	1.48698	-0.00373
C	-2.36921	2.70220	-0.02147
C	-3.75819	2.69896	-0.03453
C	-4.44602	1.48703	-0.03128

H	-4.30677	-0.66082	-0.01754
H	-1.82084	-0.66352	0.00908
H	-1.82050	3.63728	-0.02758
H	-4.30644	3.63465	-0.05388
C	-0.23619	1.48695	0.00770
N	0.92327	1.48686	0.01802
O	2.13268	1.48679	0.02865
C	-5.95023	1.48572	0.00813
F	-6.46830	2.57237	-0.58607
F	-6.46591	0.40612	-0.60120
F	-6.40929	1.47601	1.27363

p-Cl-dipole

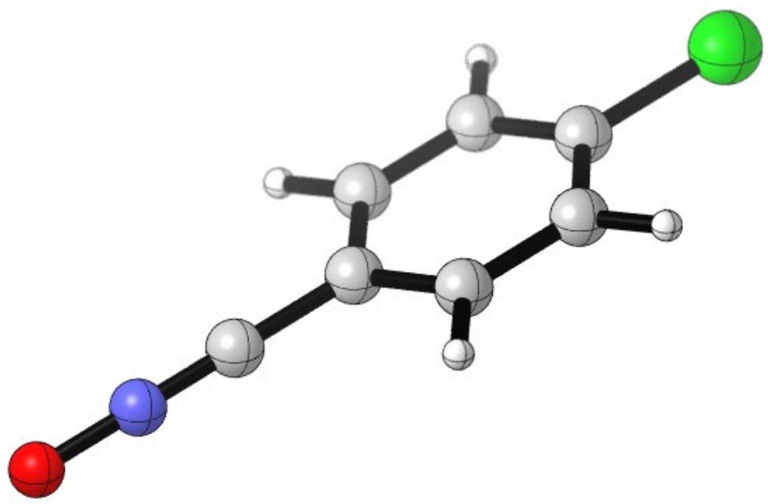

Sum of Electronic and Zero-point Energies = -858.957809 Hartree
Sum of Electronic and Thermal Energies = -858.949504 Hartree
Sum of Electronic and Thermal Enthalpies = -858.948559 Hartree
Sum of Electronic and Thermal Free Energies = -858.992391 Hartree

Dipole Moment = 3.2439 Debye

0 1

C	-3.75311	0.27328	-0.00357
C	-2.36390	0.27414	-0.00299
C	-1.66107	1.48694	-0.00347
C	-2.36382	2.69979	-0.00454
C	-3.75303	2.70075	-0.00512
C	-4.43630	1.48704	-0.00463
H	-4.30589	-0.65931	-0.00321
H	-1.81864	-0.66327	-0.00215
H	-1.81849	3.63717	-0.00491
H	-4.30574	3.63337	-0.00596

C	-0.22713	1.48689	-0.00286
N	0.93222	1.48686	-0.00237
O	2.14366	1.48682	-0.00185
Cl	-6.17453	1.48710	-0.00537

p-CN-dipole

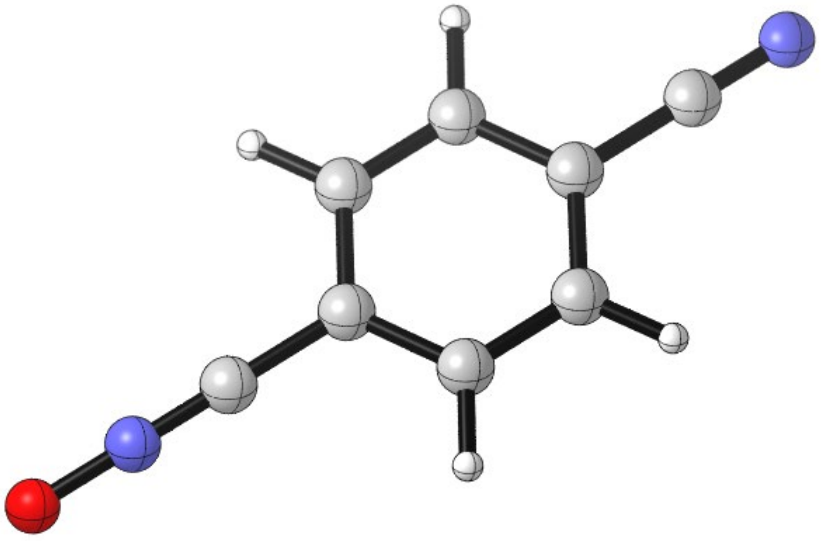

Sum of Electronic and Zero-point Energies = -491.595259 Hartree
Sum of Electronic and Thermal Energies = -491.586331 Hartree
Sum of Electronic and Thermal Enthalpies = -491.585387 Hartree
Sum of Electronic and Thermal Free Energies = -491.630381 Hartree

Dipole Moment = 0.0772 Debye

0 1

C	-3.76351	0.27162	-0.00346
C	-2.37620	0.27090	-0.00294
C	-1.67700	1.48695	-0.00346
C	-2.37610	2.70305	-0.00450
C	-3.76341	2.70245	-0.00502
C	-4.45813	1.48706	-0.00450
H	-4.31379	-0.66269	-0.00307
H	-1.82750	-0.66415	-0.00213
H	-1.82732	3.63806	-0.00489
H	-4.31362	3.63681	-0.00582
C	-0.24378	1.48689	-0.00292
N	0.91599	1.48685	-0.00248
O	2.12446	1.48680	-0.00202
C	-5.89823	1.48713	-0.00505
N	-7.05558	1.48718	-0.00548

p-F-dipole

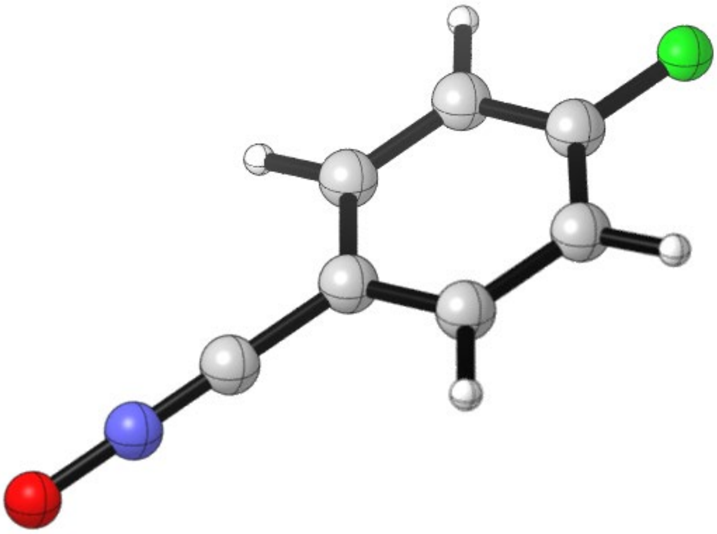

Sum of Electronic and Zero-point Energies = -498.599551 Hartree
Sum of Electronic and Thermal Energies = -498.591622 Hartree
Sum of Electronic and Thermal Enthalpies = -498.590677 Hartree
Sum of Electronic and Thermal Free Energies = -498.633193 Hartree

Dipole Moment = 3.4000 Debye

0 1

C	-3.75261	0.26935	-0.00359
C	-2.36322	0.27253	-0.00299
C	-1.66141	1.48694	-0.00346
C	-2.36315	2.70140	-0.00454
C	-3.75253	2.70466	-0.00515
C	-4.41804	1.48703	-0.00465
H	-4.32109	-0.65344	-0.00325
H	-1.81551	-0.66334	-0.00215
H	-1.81537	3.63723	-0.00491
H	-4.32095	3.62749	-0.00599
C	-0.22715	1.48690	-0.00285
N	0.93206	1.48687	-0.00235
O	2.14432	1.48683	-0.00181
F	-5.76113	1.48707	-0.00525

p-H-dipole

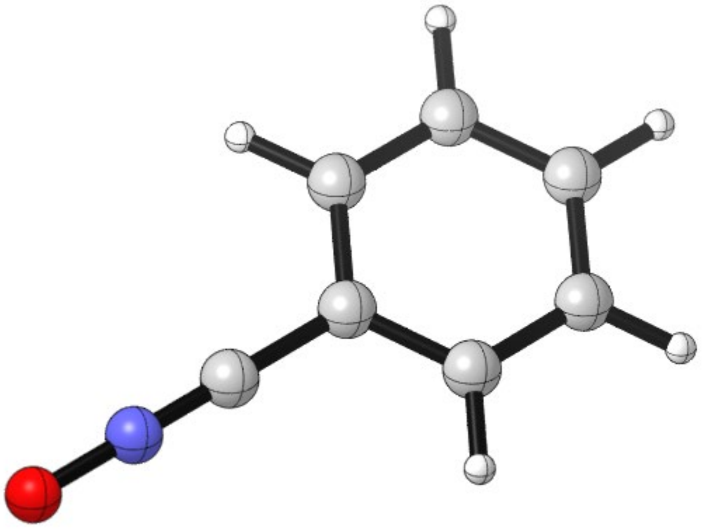

Sum of Electronic and Zero-point Energies = -399.380653 Hartree
Sum of Electronic and Thermal Energies = -399.373532 Hartree
Sum of Electronic and Thermal Enthalpies = -399.372587 Hartree
Sum of Electronic and Thermal Free Energies = -399.412995 Hartree

Dipole Moment = 5.0584 Debye

0 1

C	-3.75348	0.27896	-0.00361
C	-2.36287	0.27209	-0.00300
C	-1.66318	1.48694	-0.00347
C	-2.36279	2.70183	-0.00455
C	-3.75341	2.69504	-0.00515
C	-4.45033	1.48702	-0.00469
H	-4.29380	-0.66189	-0.00325
H	-1.81237	-0.66248	-0.00215
H	-1.81224	3.63637	-0.00491
H	-4.29367	3.63592	-0.00600
H	-5.53543	1.48706	-0.00516
C	-0.22765	1.48690	-0.00284
N	0.93149	1.48687	-0.00233
O	2.14434	1.48683	-0.00180

p-Me-dipole

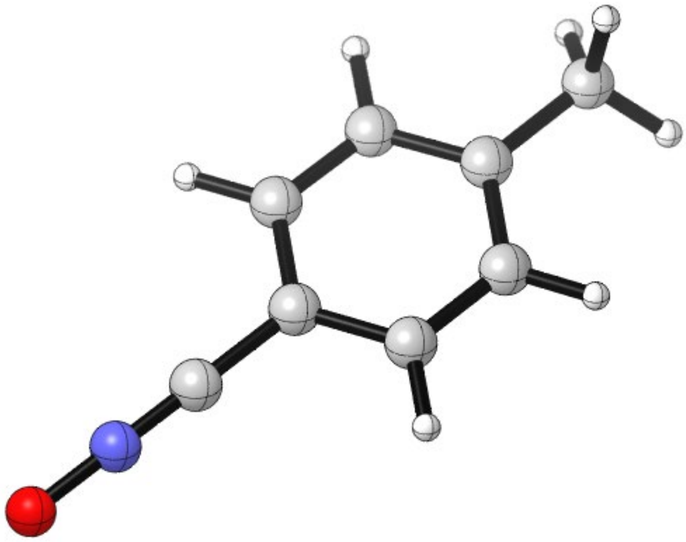

```
Sum of Electronic and Zero-point Energies = -438.652998 Hartree
Sum of Electronic and Thermal Energies = -438.643978 Hartree
Sum of Electronic and Thermal Enthalpies = -438.643034 Hartree
Sum of Electronic and Thermal Free Energies = -438.688529 Hartree

Dipole Moment = 5.7127 Debye

0 1
C                -3.75414      0.28580      0.00904
C                -2.36478      0.27594      0.00017
C                -1.65972      1.48698     -0.00408
C                -2.36467      2.69806      0.00206
C                -3.75404      2.68832      0.01091
C                -4.47117      1.48710      0.01167
H                -4.29278     -0.65782      0.01586
H                -1.81952     -0.66186     -0.00005
H                -1.81932      3.63582      0.00330
H                -4.29258      3.63197      0.01920
C                -0.22489      1.48691     -0.01004
N                 0.93422      1.48684     -0.01576
O                 2.14810      1.48677     -0.02174
C                -5.97741      1.48712     -0.01207
H                -6.38083      2.37344      0.48339
H                -6.34547      1.48626     -1.04358
H                -6.38086      0.60162      0.48484
```

p-NO2-dipole

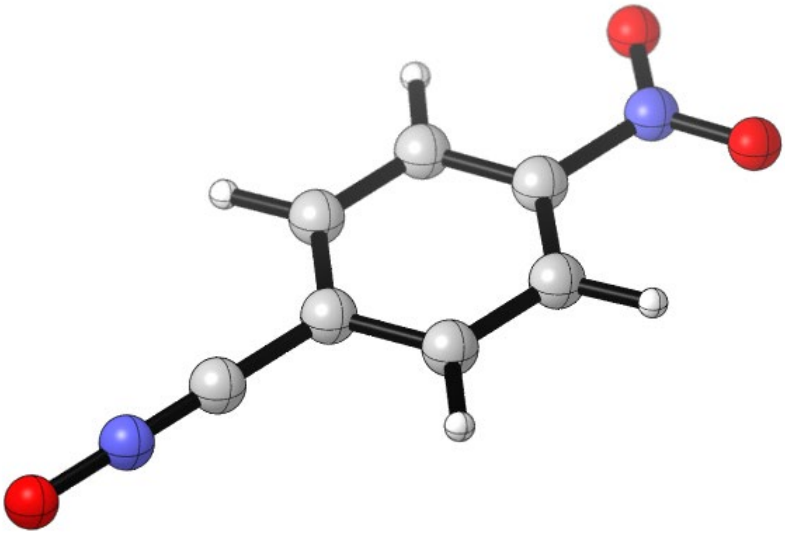

Sum of Electronic and Zero-point Energies = -603.807878 Hartree
Sum of Electronic and Thermal Energies = -603.798264 Hartree
Sum of Electronic and Thermal Enthalpies = -603.797319 Hartree
Sum of Electronic and Thermal Free Energies = -603.844539 Hartree

Dipole Moment = 0.2990 Debye

0 1

C	-3.77766	0.26701	-0.00344
C	-2.38951	0.26923	-0.00294
C	-1.69208	1.48695	-0.00345
C	-2.38940	2.70473	-0.00446
C	-3.77755	2.70708	-0.00496
C	-4.44218	1.48707	-0.00444
H	-4.34551	-0.65531	-0.00307
H	-1.83997	-0.66513	-0.00215
H	-1.83977	3.63904	-0.00485
H	-4.34531	3.62945	-0.00575
C	-0.25906	1.48689	-0.00293
N	0.90079	1.48684	-0.00251
O	2.10829	1.48678	-0.00207
N	-5.91753	1.48714	-0.00498
O	-6.47774	0.40660	-0.00451
O	-6.47765	2.56772	-0.00584

p-OMe-dipole

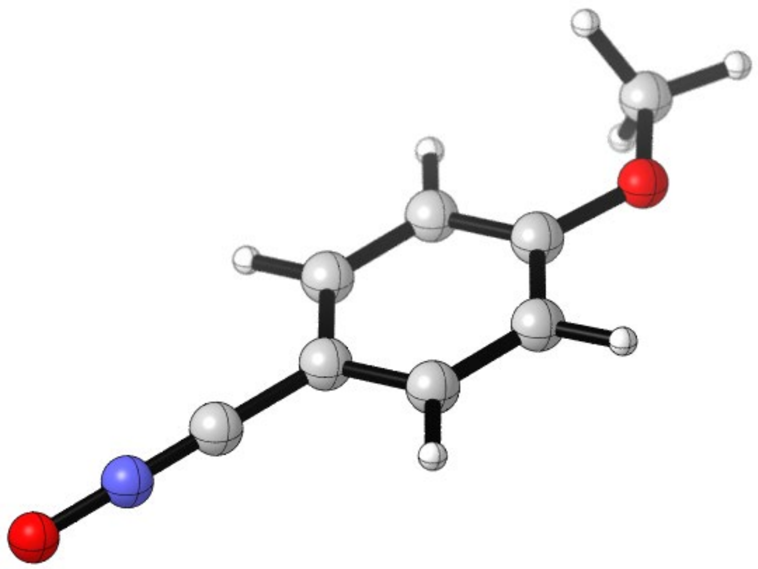

Sum of Electronic and Zero-point Energies = -513.831704 Hartree
Sum of Electronic and Thermal Energies = -513.822069 Hartree
Sum of Electronic and Thermal Enthalpies = -513.821125 Hartree
Sum of Electronic and Thermal Free Energies = -513.867475 Hartree

Dipole Moment = 6.2293 Debye

0 1

C	-3.75118	0.36007	-0.00376
C	-2.37005	0.30894	-0.00312
C	-1.61720	1.49684	-0.00339
C	-2.28112	2.72664	-0.00430
C	-3.67244	2.78375	-0.00495
C	-4.41171	1.59714	-0.00468
H	-4.34907	-0.54461	-0.00357
H	-1.85979	-0.64824	-0.00241
H	-1.70475	3.64565	-0.00451
H	-4.16131	3.74999	-0.00565
C	-0.18467	1.44581	-0.00272
N	0.97356	1.39946	-0.00218
O	2.18763	1.35246	-0.00162
O	-5.76358	1.54100	-0.00525
C	-6.48034	2.76136	-0.00617
H	-7.53452	2.48900	-0.00651
H	-6.25455	3.35096	0.88956
H	-6.25373	3.35010	-0.90226

HFIP-anion

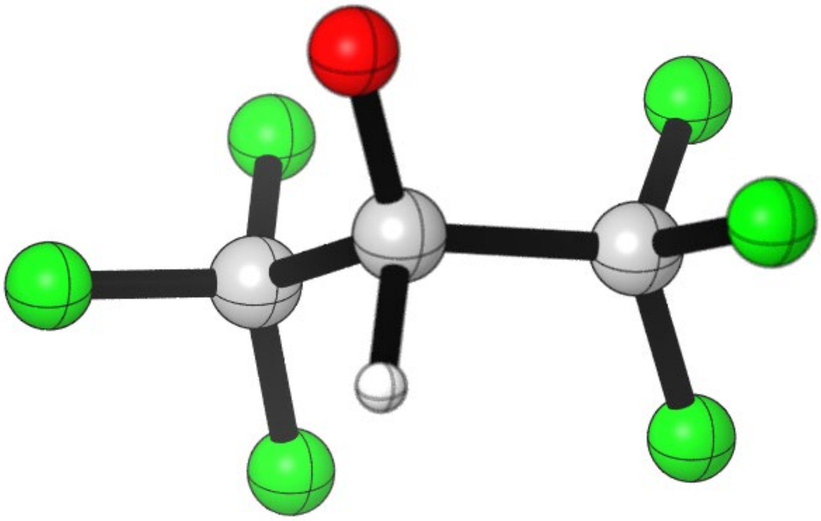

Sum of Electronic and Zero-point Energies = -788.972375 Hartree
Sum of Electronic and Thermal Energies = -788.963584 Hartree
Sum of Electronic and Thermal Enthalpies = -788.962640 Hartree
Sum of Electronic and Thermal Free Energies = -789.007738 Hartree

Dipole Moment = 19.3022 Debye

-1 1

C	-3.22677	-0.27560	0.12453
C	-5.40911	0.98913	0.12258
C	-3.90716	1.06514	-0.26832
H	-3.94767	0.99326	-1.39485
F	-5.61880	0.88714	1.44870
F	-6.09081	-0.04751	-0.44392
F	-6.05120	2.10325	-0.28410
F	-3.78592	-1.38319	-0.44217
F	-1.94024	-0.27846	-0.28008
F	-3.21313	-0.50769	1.45079
O	-3.30356	2.10748	0.25058

HFIP-OH

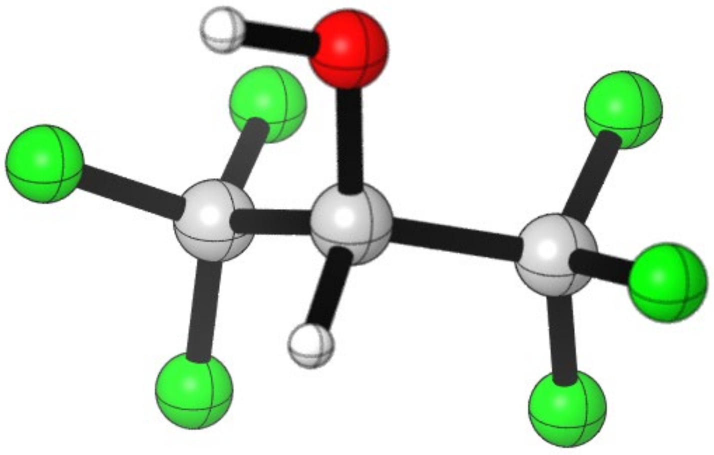

```
Sum of Electronic and Zero-point Energies = -789.514064 Hartree
Sum of Electronic and Thermal Energies = -789.504967 Hartree
Sum of Electronic and Thermal Enthalpies = -789.504023 Hartree
Sum of Electronic and Thermal Free Energies = -789.549125 Hartree

Dipole Moment = 2.7718 Debye

0 1
C               -3.25065     -0.25911      0.14529
C               -5.48573      0.96204      0.20971
C               -4.01235      1.02781     -0.19204
H               -3.98533      1.13579     -1.28481
F               -5.64648      0.94360      1.53084
F               -6.06066     -0.13569     -0.30055
F               -6.13119      2.02879     -0.27376
F               -3.61255     -1.26938     -0.65025
F               -1.93697     -0.03210     -0.05545
F               -3.40842     -0.63981      1.41133
O               -3.46771      2.11500      0.48487
H               -2.57154      2.28170      0.16825
```

oxime-H-atom-abstraction-Cl-HFIP-complex-triplet-R-from-TS-iv

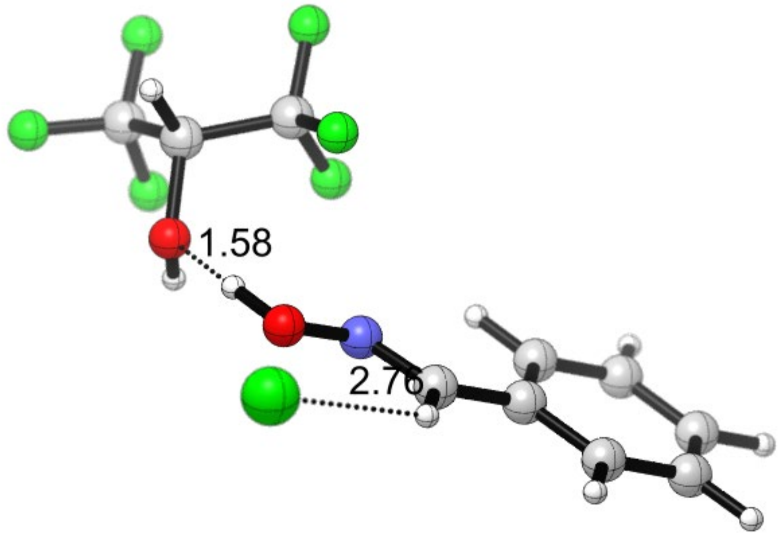


```
Sum of Electronic and Zero-point Energies = -1649.880646 Hartree
Sum of Electronic and Thermal Energies = -1649.860271 Hartree
Sum of Electronic and Thermal Enthalpies = -1649.859327 Hartree
Sum of Electronic and Thermal Free Energies = -1649.936807 Hartree

Dipole Moment = 21.7280 Debye

1 3
C              -4.67043     -1.49337     -1.13444
H              -5.07459     -1.77990     -2.10717
C              -4.75070     -2.34700     -0.01427
C              -5.32174     -3.64296     -0.18146
C              -4.28440     -1.93026      1.27307
C              -5.40997     -4.49462      0.89372
H              -5.67618     -3.94386     -1.16254
C              -4.38421     -2.79205      2.33806
H              -3.87524     -0.93237      1.38716
C              -4.94102     -4.07137      2.15182
H              -5.83680     -5.48421      0.78049
H              -4.04054     -2.49241      3.32116
H              -5.01416     -4.74644      2.99868
N              -4.08754     -0.30778     -0.99138
O              -4.12821      0.38948     -2.09181
H              -3.66492      1.26197     -1.86494
C              -4.20292      2.69751      0.66793
C              -2.19976      4.21807      0.21680
C              -3.34399      3.39898     -0.38225
H              -3.98138      4.05687     -0.97679
F              -1.29705      3.36944      0.74150
F              -2.62741      5.03325      1.16663
F              -1.59968      4.91944     -0.73508
F              -4.75480      3.54532      1.51818
F              -5.17697      2.02198      0.04130
F              -3.46649      1.80725      1.34937
```

O	-2.78643	2.41842	-1.23659
H	-1.99984	2.04813	-0.80257
Cl	-5.29522	-0.43235	-4.50349

oxime-H-atom-abstraction-Cl-HFIP-complex-triplet-TS-iv

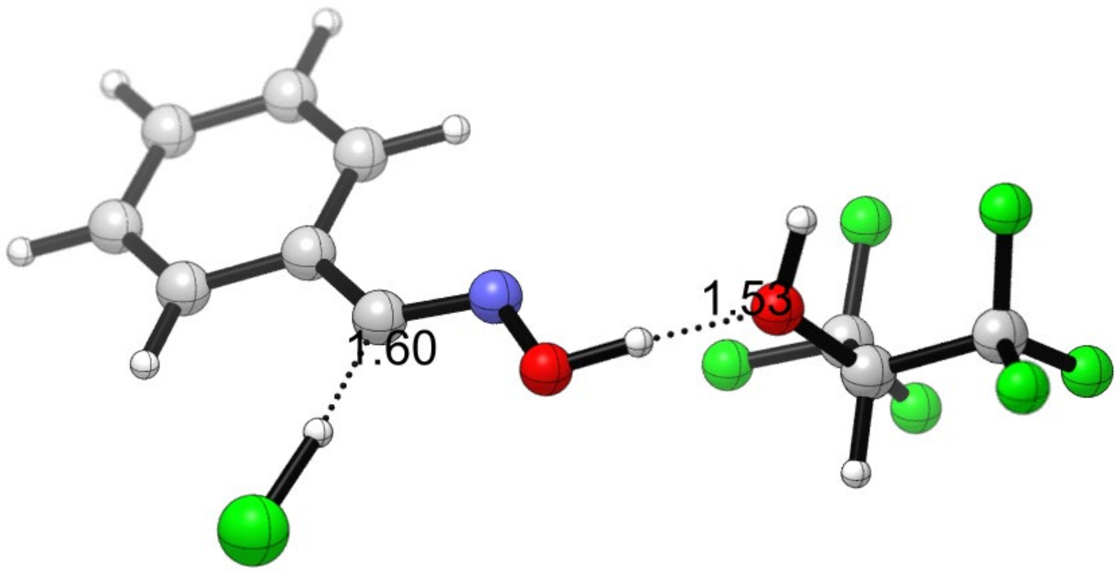

Sum of Electronic and Zero-point Energies = -1649.902087 Hartree
Sum of Electronic and Thermal Energies = -1649.881776 Hartree
Sum of Electronic and Thermal Enthalpies = -1649.880832 Hartree
Sum of Electronic and Thermal Free Energies = -1649.958776 Hartree

Dipole Moment = 20.9827 Debye

1 3

C	-4.53587	-1.50158	-0.86015
H	-5.13802	-1.62567	-2.34193
C	-4.76593	-2.53919	0.05424
C	-5.49888	-3.68779	-0.37165
C	-4.27407	-2.44787	1.39861
C	-5.72704	-4.71108	0.52010
H	-5.86418	-3.73592	-1.39329
C	-4.51526	-3.48098	2.27145
H	-3.72339	-1.56368	1.70130
C	-5.23829	-4.60965	1.83605
H	-6.28031	-5.59159	0.21425
H	-4.15329	-3.43161	3.29224
H	-5.42328	-5.42067	2.53389
N	-3.88107	-0.39947	-0.65138
O	-3.79253	0.43131	-1.63982
H	-3.26978	1.24933	-1.31269
C	-3.94056	3.26008	0.89965
C	-2.26785	4.81532	-0.27219

C	-3.23899	3.63518	-0.40966
H	-4.00146	3.89183	-1.14952
F	-1.16092	4.40129	0.36954
F	-2.81005	5.81583	0.40653
F	-1.90593	5.24359	-1.47783
F	-4.76707	4.20896	1.31017
F	-4.65270	2.13681	0.68631
F	-3.05266	2.99627	1.86272
O	-2.52256	2.51183	-0.88433
H	-1.67370	2.42819	-0.41793
Cl	-5.72591	-1.85794	-3.54858

R (neutral singlet oxime)

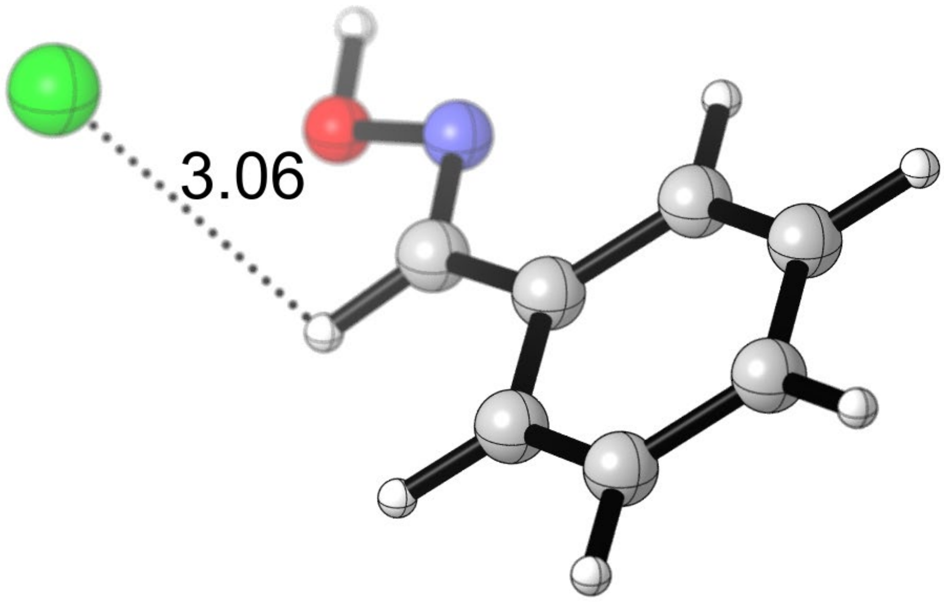

Sum of Electronic and Zero-point Energies = -860.706656 Hartree
Sum of Electronic and Thermal Energies = -860.696797 Hartree
Sum of Electronic and Thermal Enthalpies = -860.695853 Hartree
Sum of Electronic and Thermal Free Energies = -860.745009 Hartree

Dipole Moment = 2.4026 Debye

0 2

C	-2.53216	1.53420	1.05349
C	-1.15899	1.37139	0.92444
C	-0.63800	0.14434	0.49024
C	-1.50881	-0.90807	0.19192
C	-2.88582	-0.74113	0.32232
C	-3.39877	0.47970	0.75313
H	-2.93242	2.48529	1.38980

H	-0.47808	2.18397	1.15550
H	-1.10468	-1.85880	-0.14559
H	-3.55481	-1.56258	0.08752
H	-4.47108	0.61258	0.85591
N	1.64293	0.86216	0.55188
O	2.94517	0.40534	0.38168
H	3.48547	1.20695	0.40795
C	0.80551	-0.07468	0.33694
H	1.15914	-1.06030	0.02275
Cl	3.42786	-0.44509	-1.93760

TS (neutral singlet oxime)

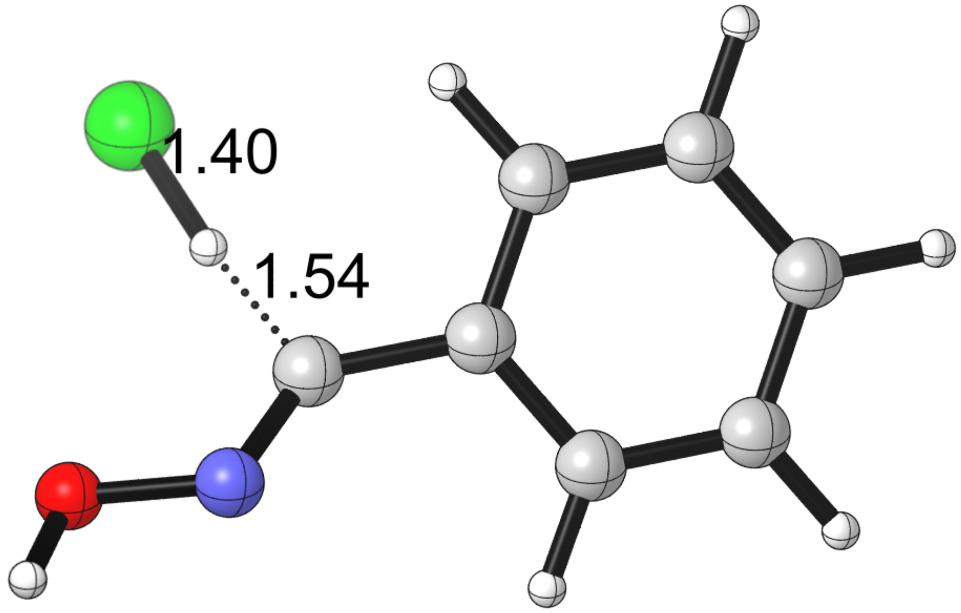

Sum of Electronic and Zero-point Energies = -860.697162 Hartree
Sum of Electronic and Thermal Energies = -860.687492 Hartree
Sum of Electronic and Thermal Enthalpies = -860.686548 Hartree
Sum of Electronic and Thermal Free Energies = -860.734997 Hartree

Dipole Moment = 4.4804 Debye

0 2

C	-1.98709	1.18199	0.44346
C	-0.60765	1.17884	0.27833
C	0.03063	0.02307	-0.19887
C	-0.72043	-1.11012	-0.53491
C	-2.10106	-1.09623	-0.36444
C	-2.73388	0.04616	0.12416
H	-2.48246	2.07189	0.81794
H	-0.01260	2.05395	0.51781

H	-0.21873	-1.98837	-0.92982
H	-2.68355	-1.97521	-0.61926
H	-3.81187	0.05552	0.25013
N	2.37309	0.57976	0.26325
O	3.65863	0.52245	-0.22546
H	4.19376	0.91529	0.47675
C	1.47428	0.00476	-0.37196
H	1.96907	-0.89671	-1.51943
Cl	2.31163	-1.78427	-2.55049

R (radical cation)

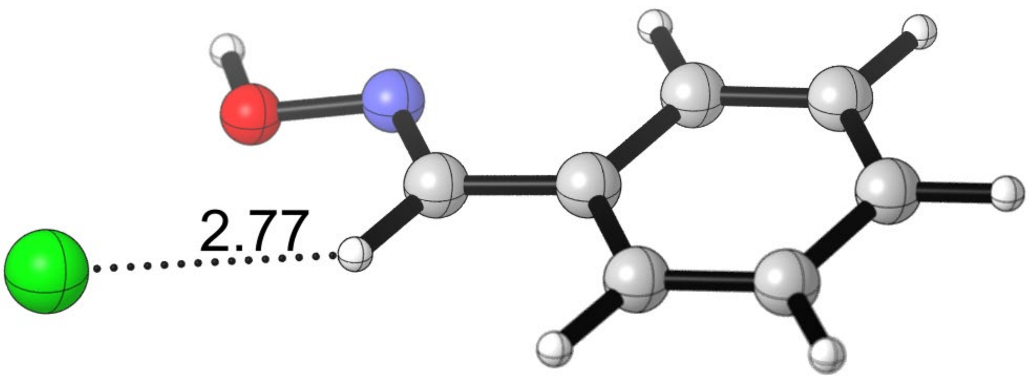

Sum of Electronic and Zero-point Energies = -860.390701 Hartree
Sum of Electronic and Thermal Energies = -860.380600 Hartree
Sum of Electronic and Thermal Enthalpies = -860.379656 Hartree
Sum of Electronic and Thermal Free Energies = -860.431098 Hartree

Dipole Moment = 4.3474 Debye

1 3

C	-2.51953	1.58165	1.15465
C	-1.16589	1.46751	0.95712
C	-0.64577	0.31188	0.28375
C	-1.53538	-0.70899	-0.17546
C	-2.88698	-0.58027	0.03051
C	-3.38148	0.56364	0.69482
H	-2.93084	2.44740	1.66097
H	-0.47952	2.23383	1.29981
H	-1.12837	-1.57801	-0.68382

H	-3.57433	-1.34574	-0.31113
H	-4.45090	0.66145	0.85532
N	1.57254	1.08390	0.47852
O	2.81780	0.77121	0.18097
H	3.37980	1.49338	0.51698
C	0.73899	0.14617	0.05514
H	1.13214	-0.73214	-0.46110
Cl	3.57039	-1.45333	-1.57248

TS (radical cation)

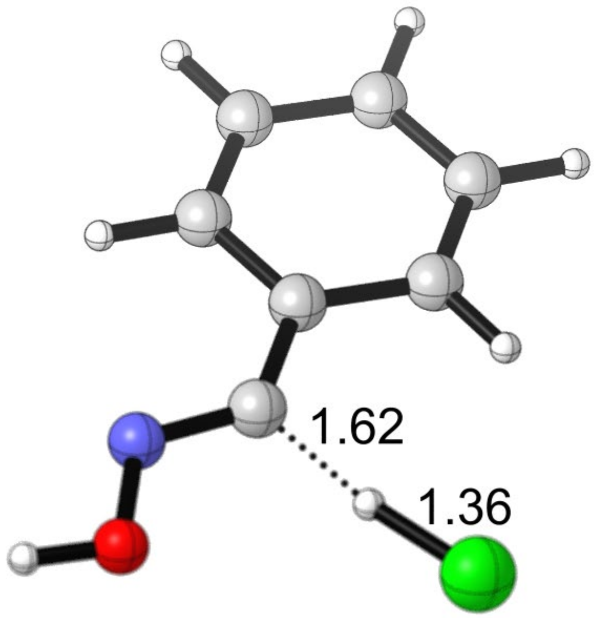

Sum of Electronic and Zero-point Energies = -860.365201 Hartree
Sum of Electronic and Thermal Energies = -860.355461 Hartree
Sum of Electronic and Thermal Enthalpies = -860.354517 Hartree
Sum of Electronic and Thermal Free Energies = -860.403346 Hartree

Dipole Moment = 3.3037 Debye

1 3

C	-1.92877	1.13082	0.63690
C	-0.56792	1.11994	0.46923
C	0.04215	0.02593	-0.23880
C	-0.75832	-1.03800	-0.76242
C	-2.11970	-1.00575	-0.58236
C	-2.70615	0.07321	0.11435
H	-2.41090	1.94447	1.16705
H	0.06012	1.91497	0.85690
H	-0.27547	-1.85331	-1.29312
H	-2.74484	-1.80165	-0.97085

H	-3.78315	0.09080	0.25233
N	2.29144	0.86470	-0.01744
O	3.54743	0.62393	-0.30511
H	4.07078	1.36247	0.06189
C	1.43416	-0.01039	-0.42481
H	2.07934	-1.26415	-1.21981
Cl	2.42158	-2.39524	-1.88674

R (radical cation)

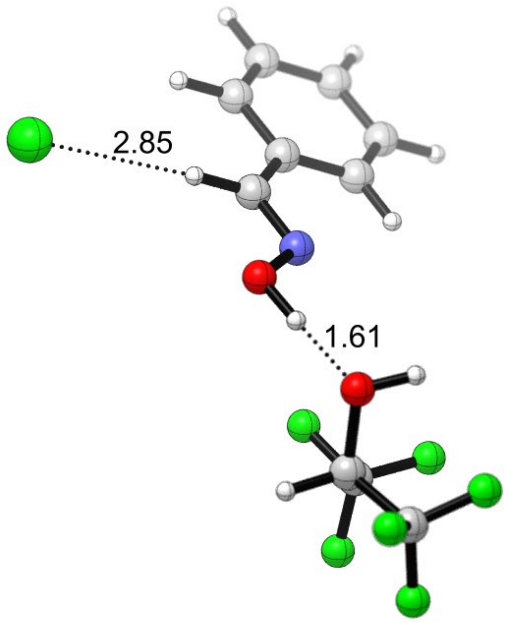

Sum of Electronic and Zero-point Energies = -1649.923290 Hartree
Sum of Electronic and Thermal Energies = -1649.902383 Hartree
Sum of Electronic and Thermal Enthalpies = -1649.901439 Hartree
Sum of Electronic and Thermal Free Energies = -1649.982920 Hartree

Dipole Moment = 12.1727 Debye

1 3

C	-0.69209	-2.41855	-0.79496
H	-1.13356	-2.53287	-1.78675
C	-0.83847	-3.41780	0.19034
C	-1.56647	-4.59695	-0.14867
C	-0.28482	-3.27296	1.50155
C	-1.73069	-5.59406	0.78507
H	-1.98263	-4.69234	-1.14733
C	-0.45873	-4.27782	2.42410
H	0.26166	-2.36951	1.74828
C	-1.17786	-5.43575	2.07032
H	-2.28032	-6.49550	0.53946
H	-0.04700	-4.18359	3.42243

H	-1.30992	-6.22393	2.80519
N	-0.00763	-1.31341	-0.51052
O	0.01108	-0.50168	-1.53020
H	0.53070	0.31027	-1.23859
C	-0.10457	2.32849	1.01676
C	1.75980	3.81165	0.06068
C	0.71545	2.72284	-0.21595
H	0.02744	3.08795	-0.98267
F	2.77731	3.28222	0.76268
F	1.24550	4.82286	0.74686
F	2.25056	4.26618	-1.08965
F	-0.86076	3.32512	1.45362
F	-0.90683	1.30307	0.67865
F	0.68942	1.90920	2.00805
O	1.37128	1.57862	-0.71858
H	2.16485	1.39130	-0.19024
Cl	-2.72770	-2.91522	-4.11918

TS (radical cation)

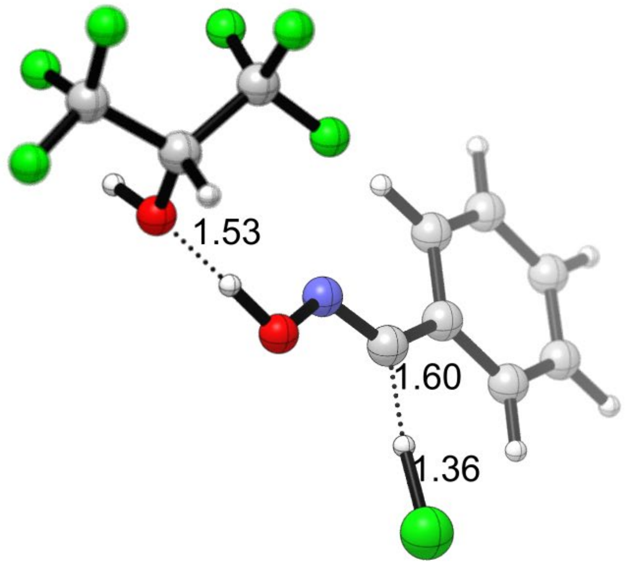

Sum of Electronic and Zero-point Energies = -1649.902087 Hartree
Sum of Electronic and Thermal Energies = -1649.881776 Hartree
Sum of Electronic and Thermal Enthalpies = -1649.880832 Hartree
Sum of Electronic and Thermal Free Energies = -1649.958776 Hartree

Dipole Moment = 20.9827 Debye

1 3

C	-4.53587	-1.50158	-0.86015
H	-5.13802	-1.62567	-2.34193

C	-4.76593	-2.53919	0.05424
C	-5.49888	-3.68779	-0.37165
C	-4.27407	-2.44787	1.39861
C	-5.72704	-4.71108	0.52010
H	-5.86418	-3.73592	-1.39329
C	-4.51526	-3.48098	2.27145
H	-3.72339	-1.56368	1.70130
C	-5.23829	-4.60965	1.83605
H	-6.28031	-5.59159	0.21425
H	-4.15329	-3.43161	3.29224
H	-5.42328	-5.42067	2.53389
N	-3.88107	-0.39947	-0.65138
O	-3.79253	0.43131	-1.63982
H	-3.26978	1.24933	-1.31269
C	-3.94056	3.26008	0.89965
C	-2.26785	4.81532	-0.27219
C	-3.23899	3.63518	-0.40966
H	-4.00146	3.89183	-1.14952
F	-1.16092	4.40129	0.36954
F	-2.81005	5.81583	0.40653
F	-1.90593	5.24359	-1.47783
F	-4.76707	4.20896	1.31017
F	-4.65270	2.13681	0.68631
F	-3.05266	2.99627	1.86272
O	-2.52256	2.51183	-0.88433
H	-1.67370	2.42819	-0.41793
Cl	-5.72591	-1.85794	-3.54858

Ph-oxime-HFIP-OH-complex

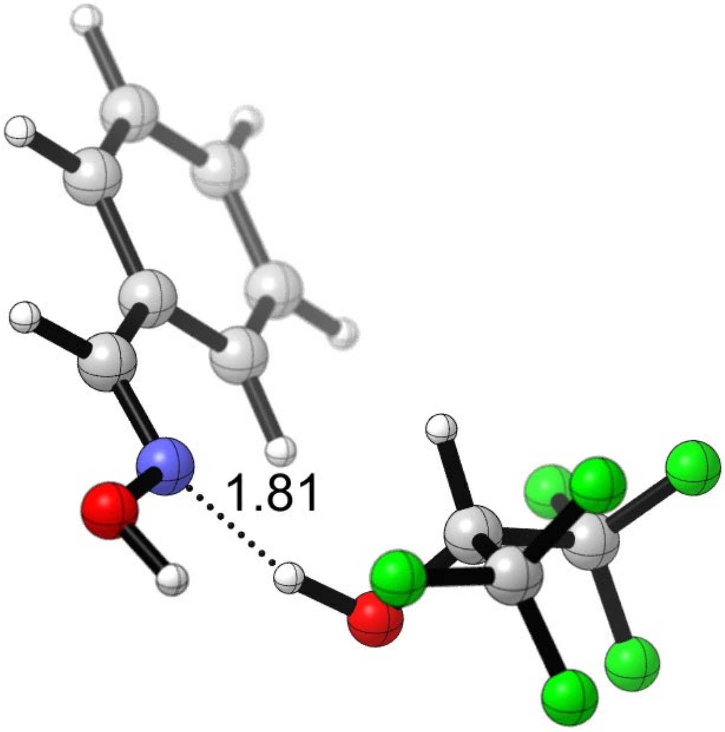

Sum of Electronic and Zero-point Energies = -1190.129019 Hartree
Sum of Electronic and Thermal Energies = -1190.110788 Hartree
Sum of Electronic and Thermal Enthalpies = -1190.109844 Hartree
Sum of Electronic and Thermal Free Energies = -1190.179464 Hartree

Dipole Moment = 4.6627 Debye

0 1

C	-3.13580	-1.36986	-1.00702
H	-2.48190	-2.22704	-1.18301
C	-4.40624	-1.58484	-0.30219
C	-4.63858	-2.84628	0.25711
C	-5.37406	-0.57963	-0.15771
C	-5.81054	-3.10012	0.96510
H	-3.89369	-3.62910	0.14108
C	-6.53988	-0.83507	0.55400
H	-5.22717	0.39692	-0.60855
C	-6.76117	-2.09358	1.11766
H	-5.97926	-4.08128	1.39662
H	-7.28022	-0.04939	0.66440
H	-7.67475	-2.28776	1.67029
N	-2.75577	-0.21594	-1.39483
O	-1.53106	-0.25592	-2.02952
H	-1.27577	0.67231	-2.12348
C	-2.01202	3.13784	0.08687
C	-4.44641	3.40519	0.78995
C	-3.41424	2.52140	0.09297
H	-3.34397	1.59124	0.67738

F	-4.67404	4.53791	0.12618
F	-4.05486	3.72133	2.03265
F	-5.60818	2.74155	0.88419
F	-1.48657	3.21019	1.31403
F	-1.19880	2.34591	-0.64904
F	-1.98199	4.35406	-0.45503
O	-3.84495	2.31194	-1.20731
H	-3.55256	1.40453	-1.45391

Ph-oxime-HFIP-OH-complex_radical-cation-freq

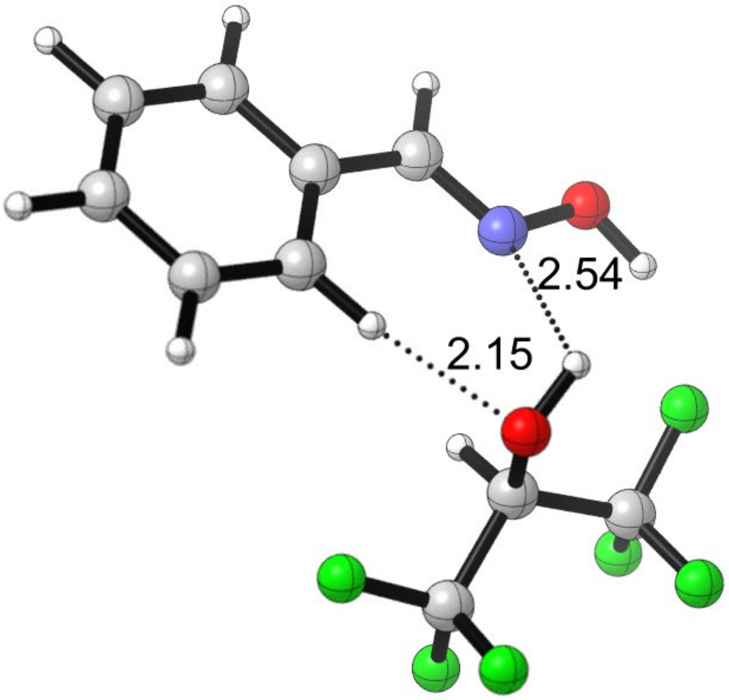

Sum of Electronic and Zero-point Energies = -1189.762123 Hartree
Sum of Electronic and Thermal Energies = -1189.743839 Hartree
Sum of Electronic and Thermal Enthalpies = -1189.742895 Hartree
Sum of Electronic and Thermal Free Energies = -1189.811966 Hartree

Dipole Moment = 21.2414 Debye

1 2

C	-3.01642	-1.48611	-0.65312
H	-2.32174	-2.31940	-0.76373
C	-4.32971	-1.68636	-0.17603
C	-4.71753	-3.01660	0.17765
C	-5.25299	-0.60086	-0.00729
C	-5.96845	-3.25007	0.68983
H	-4.01060	-3.82933	0.04190

C	-6.49857	-0.85249	0.51204
H	-4.97232	0.40625	-0.30331
C	-6.85972	-2.16870	0.86179
H	-6.27550	-4.25221	0.96527
H	-7.20391	-0.04134	0.65134
H	-7.84756	-2.35779	1.27052
N	-2.59906	-0.26122	-0.94474
O	-1.33840	-0.27348	-1.31519
H	-1.09856	0.66284	-1.47052
C	-2.18263	3.27748	-0.40400
C	-4.29140	3.44243	1.00755
C	-3.46415	2.59102	0.05150
H	-3.15663	1.69428	0.60969
F	-4.74671	4.53979	0.42447
F	-3.55370	3.78037	2.06421
F	-5.33348	2.71412	1.42717
F	-1.28944	3.35580	0.56833
F	-1.63710	2.48765	-1.39420
F	-2.38237	4.46678	-0.93179
O	-4.28327	2.30377	-1.03969
H	-3.75253	1.86988	-1.72074

Ph-oxime

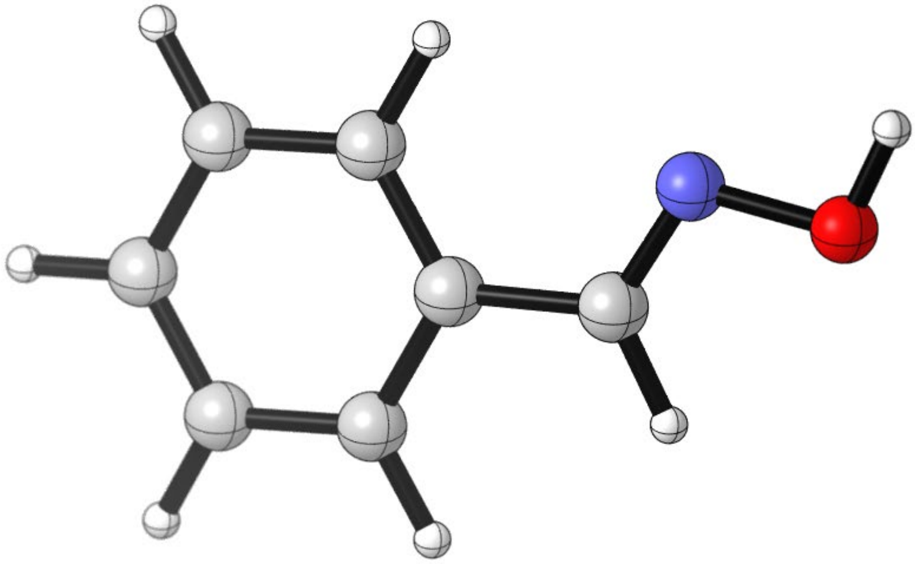

Sum of Electronic and Zero-point Energies = -400.597031 Hartree
Sum of Electronic and Thermal Energies = -400.589374 Hartree

Sum of Electronic and Thermal Enthalpies = -400.588430 Hartree
Sum of Electronic and Thermal Free Energies = -400.629581 Hartree

Dipole Moment = 0.6361 Debye

0 1

C	-3.17812	-1.98685	-0.12974
H	-2.66770	-2.95326	-0.10801
C	-4.64720	-1.96183	-0.06783
C	-5.33737	-3.17439	0.02026
C	-5.36952	-0.76073	-0.09448
C	-6.72956	-3.19305	0.08141
H	-4.77978	-4.10722	0.04110
C	-6.75720	-0.78209	-0.03342
H	-4.82990	0.17810	-0.16323
C	-7.44194	-1.99707	0.05475
H	-7.25487	-4.14029	0.14982
H	-7.31105	0.15122	-0.05455
H	-8.52625	-2.00802	0.10219
N	-2.50101	-0.91101	-0.20699
O	-1.14262	-1.17762	-0.25346
H	-0.73818	-0.30419	-0.30805

Ph-oxime_radical-cation-freq

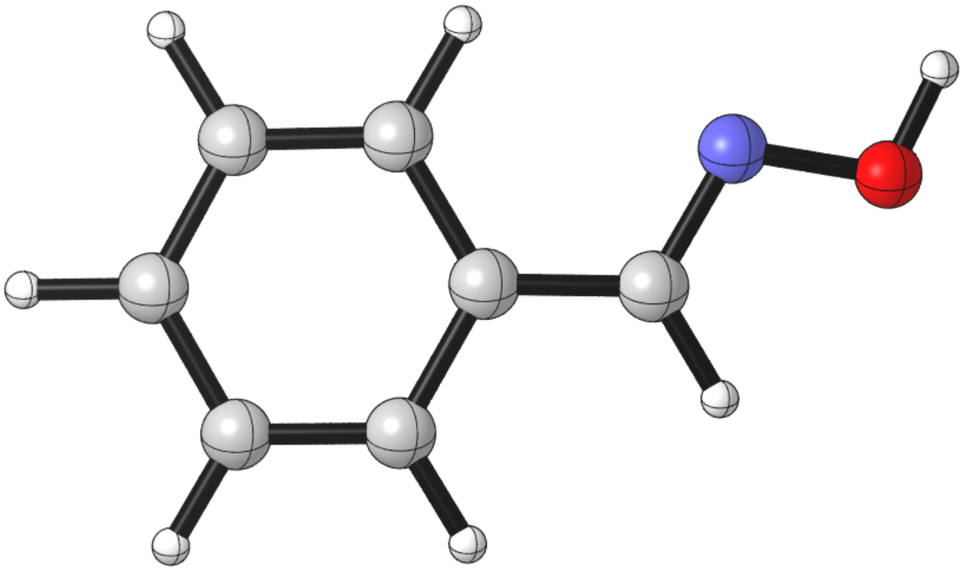

Sum of Electronic and Zero-point Energies = -400.278887 Hartree

Sum of Electronic and Thermal Energies = -400.271375 Hartree
Sum of Electronic and Thermal Enthalpies = -400.270431 Hartree
Sum of Electronic and Thermal Free Energies = -400.311737 Hartree

Dipole Moment = 23.3912 Debye

1 2

C	-3.21637	-2.03192	-0.12477
H	-2.67284	-2.97701	-0.10289
C	-4.62593	-1.98950	-0.06678
C	-5.34091	-3.22293	0.02254
C	-5.34041	-0.74703	-0.09540
C	-6.71224	-3.21415	0.08091
H	-4.78762	-4.15677	0.04338
C	-6.71127	-0.75516	-0.03630
H	-4.78346	0.18053	-0.16354
C	-7.39897	-1.98231	0.05166
H	-7.26900	-4.14141	0.14904
H	-7.26842	0.17409	-0.05676
H	-8.48353	-1.98016	0.09802
N	-2.54135	-0.89407	-0.20747
O	-1.24892	-1.12946	-0.25105
H	-0.81103	-0.26103	-0.31083

dimethyl-fumerate-product

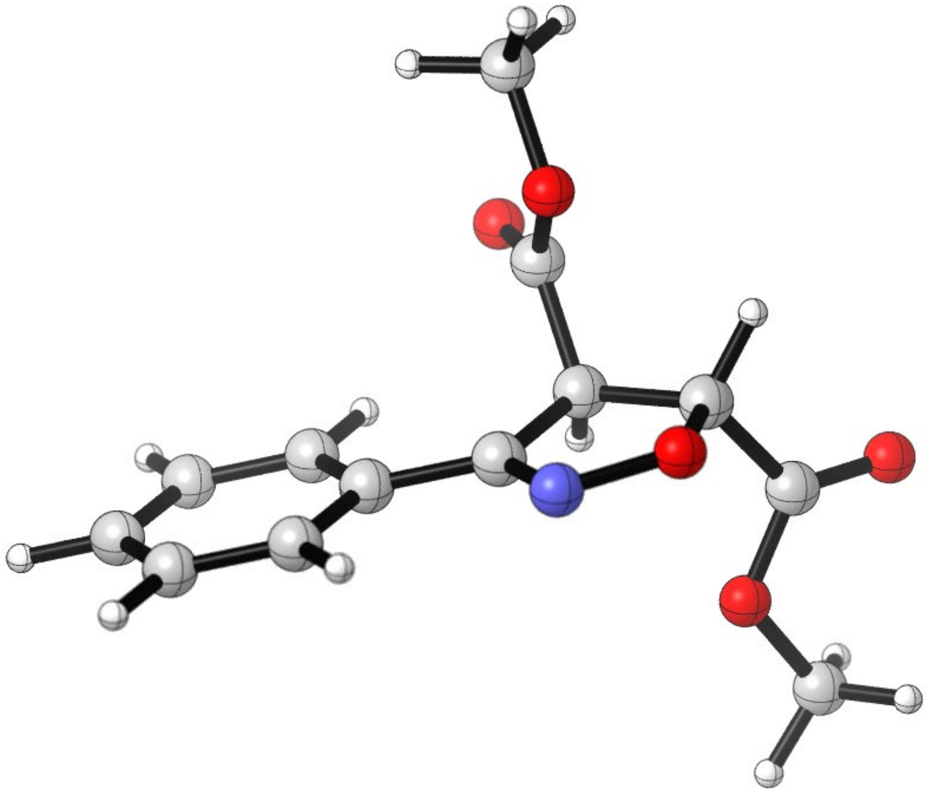

Sum of Electronic and Zero-point Energies = -933.454204 Hartree
Sum of Electronic and Thermal Energies = -933.436621 Hartree
Sum of Electronic and Thermal Enthalpies = -933.435677 Hartree
Sum of Electronic and Thermal Free Energies = -933.502604 Hartree

Dipole Moment = 2.3439 Debye

0 1

C	-0.79439	-1.56708	0.95221
H	-0.23253	-2.34288	0.42613
C	-0.08351	-0.20249	0.93754
H	-0.62745	0.56749	0.38887
C	1.32130	-0.29798	0.35923
C	-2.19333	-1.54196	0.35790
O	-2.60879	-2.34604	-0.44041
O	1.69083	0.29958	-0.62077
O	2.07581	-1.14591	1.06154
O	-2.90753	-0.52315	0.84176
C	-4.25532	-0.43163	0.36453
H	-4.68074	0.43971	0.85727
H	-4.26080	-0.30878	-0.72006
H	-4.80512	-1.33659	0.62939
C	3.42783	-1.28591	0.61120
H	3.89237	-1.99322	1.29468
H	3.44540	-1.66326	-0.41314
H	3.93433	-0.31974	0.64863
O	-0.00139	0.19933	2.31401
N	-0.38760	-0.83567	3.13621
C	-0.82401	-1.82078	2.45180
C	-1.31877	-3.05632	3.07908
C	-1.73281	-4.13336	2.28811
C	-1.37617	-3.16321	4.47684
C	-2.19808	-5.30355	2.88746
H	-1.70342	-4.06564	1.20450
C	-1.83874	-4.33166	5.06649
H	-1.05424	-2.32349	5.08370
C	-2.25157	-5.40617	4.27401
H	-2.51869	-6.13264	2.26506
H	-1.87963	-4.40753	6.14840
H	-2.61378	-6.31746	4.73919

dimethyl-maleate-product-ii

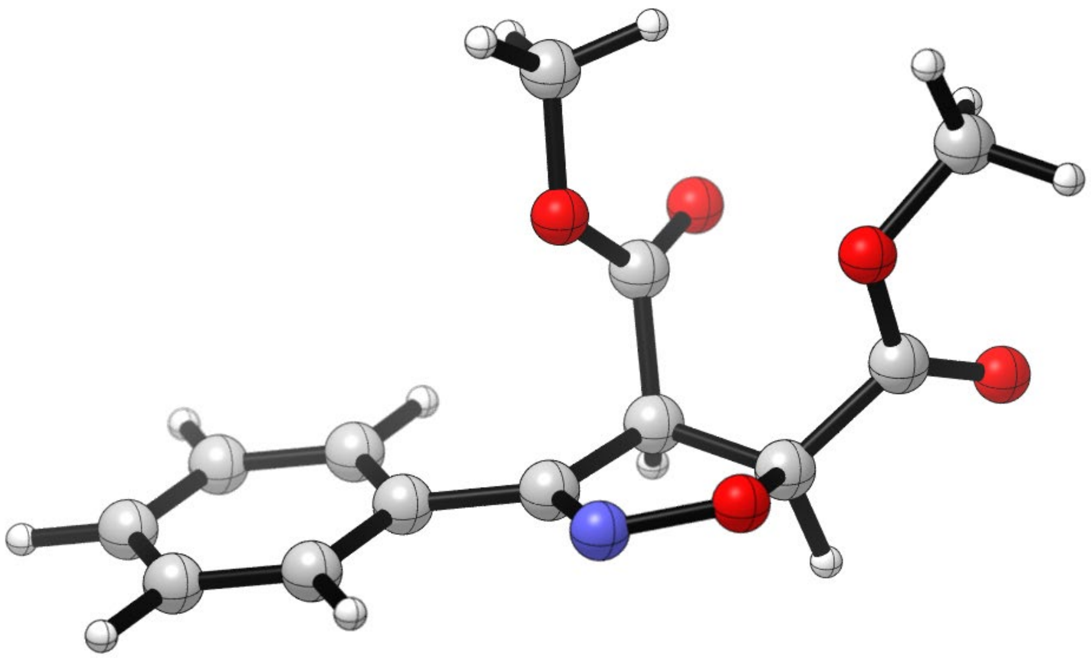

Sum of Electronic and Zero-point Energies = -933.452088 Hartree
Sum of Electronic and Thermal Energies = -933.434625 Hartree
Sum of Electronic and Thermal Enthalpies = -933.433681 Hartree
Sum of Electronic and Thermal Free Energies = -933.499513 Hartree

Dipole Moment = 3.7719 Debye

0 1

C	-0.25063	-1.71500	1.28145
C	0.34899	-0.41881	0.69573
H	-0.33649	0.02646	-0.02785
C	1.67823	-0.63192	-0.02344
O	1.74869	-1.01717	-1.16612
O	2.72728	-0.37235	0.75242
C	4.00332	-0.65024	0.17018
H	4.73558	-0.38722	0.93022
H	4.06861	-1.71074	-0.08343
H	4.14451	-0.05300	-0.73259
H	-1.34027	-1.70629	1.18083
C	0.25767	-2.99540	0.63949
O	-0.42236	-3.69031	-0.07170
O	1.54210	-3.21692	0.92189
C	2.12656	-4.33333	0.24135
H	2.12769	-4.14461	-0.83484
H	3.14155	-4.41221	0.62591
H	1.56055	-5.24174	0.45343
O	0.09129	-1.67101	2.66859
N	0.34714	-0.35871	3.02758

C	0.47356	0.36829	1.98338
C	0.76621	1.80734	2.05854
C	0.97832	2.54141	0.88762
C	0.83641	2.45550	3.29947
C	1.25995	3.90479	0.95253
H	0.92787	2.05462	-0.08292
C	1.11649	3.81454	3.35961
H	0.66941	1.88098	4.20456
C	1.32923	4.54383	2.18731
H	1.42351	4.46462	0.03742
H	1.16848	4.30990	4.32388
H	1.54631	5.60594	2.23900

p-CF3-product-endo

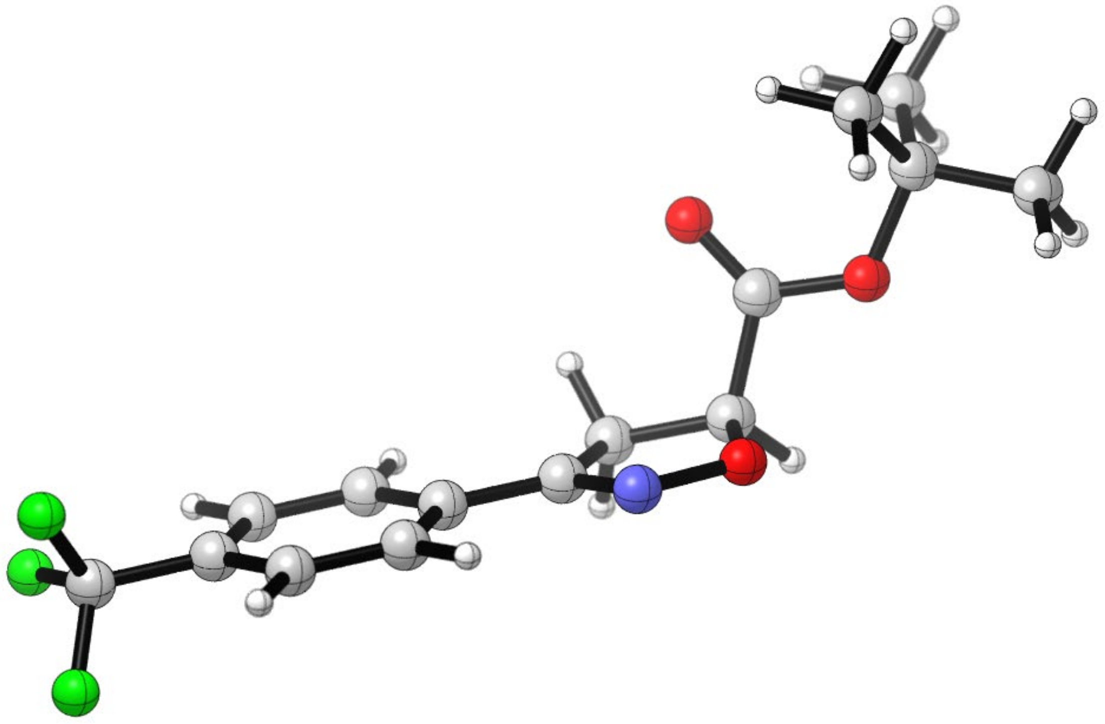

Sum of Electronic and Zero-point Energies = -1160.468336 Hartree
Sum of Electronic and Thermal Energies = -1160.447683 Hartree
Sum of Electronic and Thermal Enthalpies = -1160.446739 Hartree
Sum of Electronic and Thermal Free Energies = -1160.522607 Hartree

Dipole Moment = 4.5858 Debye

0 1

O	2.71148	2.59671	-0.04953
N	1.96802	1.45820	0.18412
C	2.70954	0.52963	0.65508

C	2.15391	-0.78317	1.01823
C	2.99268	-1.77991	1.52100
C	0.78193	-1.04422	0.87209
C	2.47792	-3.02575	1.87709
H	4.05540	-1.59146	1.63603
C	0.26849	-2.28140	1.22378
H	0.13491	-0.26609	0.48207
C	1.11921	-3.27042	1.72726
H	3.13032	-3.79855	2.26765
H	-0.79186	-2.48649	1.11255
C	4.78877	2.33856	-1.21067
O	5.22101	1.35465	-1.76511
O	4.79652	3.57841	-1.68429
C	5.25291	3.87037	-3.04643
C	4.38894	3.11237	-4.04971
H	4.62842	3.46030	-5.05887
H	4.56601	2.03734	-3.99959
H	3.33051	3.31207	-3.85873
C	5.02542	5.37238	-3.16204
H	5.60840	5.90626	-2.40660
H	5.33388	5.71833	-4.15227
H	3.96794	5.60917	-3.01892
C	6.73485	3.53287	-3.17932
H	7.09425	3.88657	-4.15031
H	7.30855	4.04047	-2.39794
H	6.90676	2.45808	-3.11219
C	0.52948	-4.60351	2.09133
F	-0.47111	-4.47680	2.98333
F	0.00508	-5.22477	1.01772
F	1.43612	-5.43614	2.62684
C	4.15408	0.93577	0.81516
H	4.82973	0.26640	0.27526
H	4.43934	0.96528	1.87094
C	4.11517	2.31311	0.16505
H	4.51498	3.12135	0.78075

p-Cl-product-endo

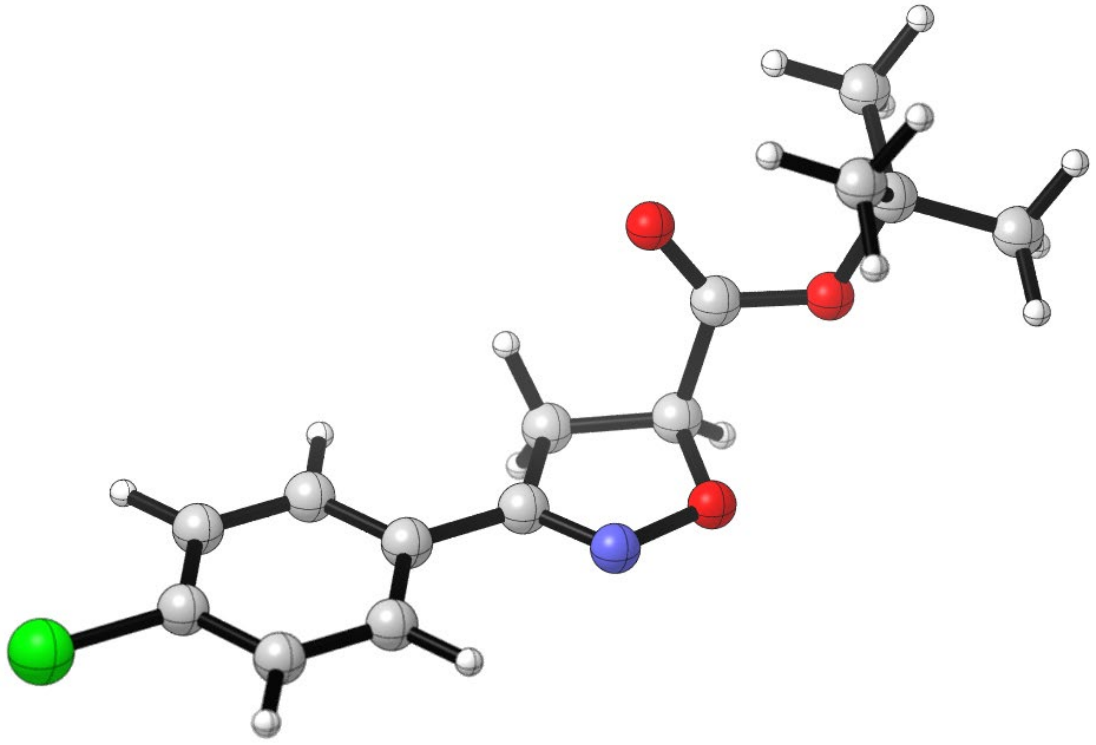

Sum of Electronic and Zero-point Energies = -1283.096773 Hartree
Sum of Electronic and Thermal Energies = -1283.078545 Hartree
Sum of Electronic and Thermal Enthalpies = -1283.077601 Hartree
Sum of Electronic and Thermal Free Energies = -1283.146431 Hartree

Dipole Moment = 3.2160 Debye

0 1

O	2.78517	2.60401	0.03039
N	2.00819	1.47786	0.23314
C	2.72560	0.51590	0.67371
C	2.14004	-0.79156	1.00433
C	2.95451	-1.82865	1.46678
C	0.76241	-1.01504	0.86831
C	2.41331	-3.07095	1.79019
H	4.02387	-1.67587	1.57590
C	0.21340	-2.24885	1.18595
H	0.12917	-0.21025	0.51017
C	1.04594	-3.26927	1.64586
H	3.04430	-3.87682	2.14791
H	-0.85117	-2.42678	1.08163
C	4.83573	2.29090	-1.17194
O	5.26564	1.30798	-1.73028
O	4.83773	3.53045	-1.64735
C	5.28171	3.82133	-3.01304
C	4.41368	3.05801	-4.00897
H	4.64254	3.40718	-5.02021
H	4.59719	1.98405	-3.96004

H	3.35580	3.25171	-3.80892
C	5.04657	5.32212	-3.13026
H	5.63230	5.86014	-2.37991
H	5.34635	5.66752	-4.12338
H	3.98905	5.55414	-2.97979
C	6.76413	3.49026	-3.15703
H	7.11422	3.84189	-4.13220
H	7.34166	4.00328	-2.38204
H	6.94124	2.41648	-3.08711
Cl	0.35724	-4.81897	2.04432
C	4.18114	0.87937	0.84012
H	4.84000	0.20147	0.29063
H	4.46480	0.88385	1.89684
C	4.17959	2.26744	0.21278
H	4.62020	3.05052	0.83347

p-CN-product-endo

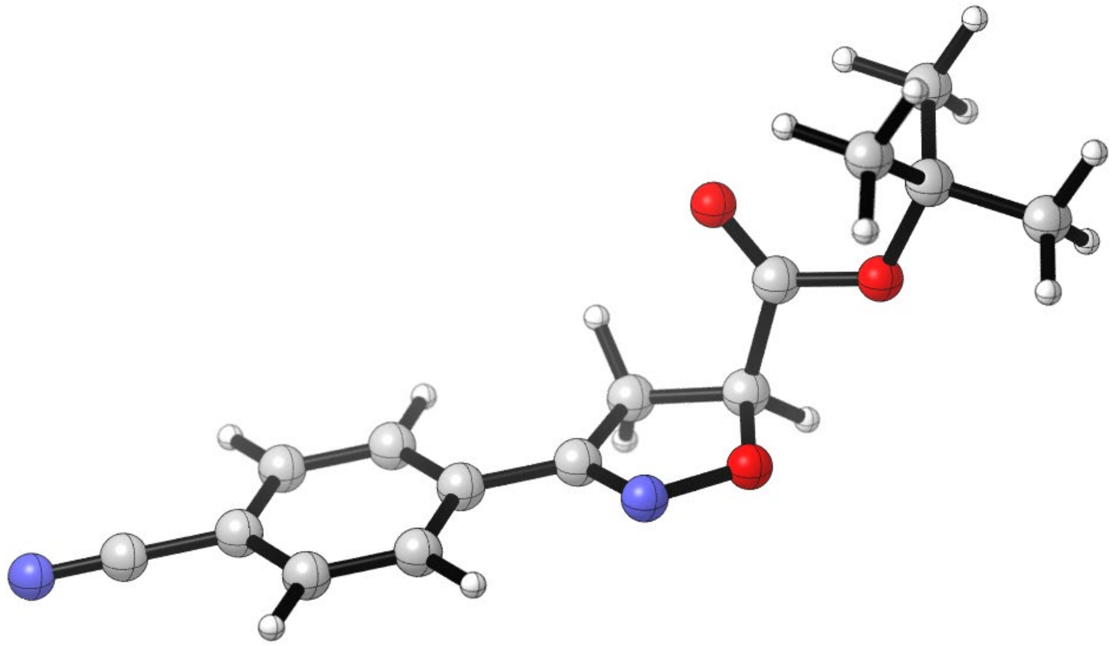

Sum of Electronic and Zero-point Energies = -915.735587 Hartree
Sum of Electronic and Thermal Energies = -915.716773 Hartree
Sum of Electronic and Thermal Enthalpies = -915.715829 Hartree
Sum of Electronic and Thermal Free Energies = -915.785487 Hartree

Dipole Moment = 6.1398 Debye

0 1

O	2.76843	2.60247	0.01001
N	2.00290	1.47764	0.22983
C	2.73019	0.52036	0.66506
C	2.14741	-0.78580	1.00640
C	2.97047	-1.81667	1.47114
C	0.76797	-1.00866	0.87750
C	2.43257	-3.05471	1.80525
H	4.03935	-1.65738	1.57171
C	0.22511	-2.23970	1.20683
H	0.13481	-0.20535	0.51685
C	1.05816	-3.26736	1.67320
H	3.06952	-3.85522	2.16523
H	-0.84034	-2.41705	1.10842
C	4.81505	2.31414	-1.20181
O	5.22756	1.33222	-1.77460
O	4.82584	3.55881	-1.66095
C	5.26253	3.86325	-3.02730
C	4.37710	3.12310	-4.02515
H	4.60298	3.48122	-5.03386
H	4.54718	2.04634	-3.99054
H	3.32331	3.32819	-3.81516
C	5.04442	5.36804	-3.12195
H	5.64322	5.88894	-2.36988
H	5.33980	5.72305	-4.11294
H	3.99113	5.61089	-2.95944
C	6.73949	3.51607	-3.18706
H	7.08626	3.87700	-4.15996
H	7.32934	4.01125	-2.40979
H	6.90423	2.43935	-3.13381
C	0.49491	-4.54722	2.01741
N	0.04230	-5.57600	2.29493
C	4.18628	0.88877	0.80989
H	4.83835	0.21600	0.24580
H	4.48849	0.89088	1.86137
C	4.16896	2.27934	0.18731
H	4.60234	3.06510	0.80927

p-F-product-endo

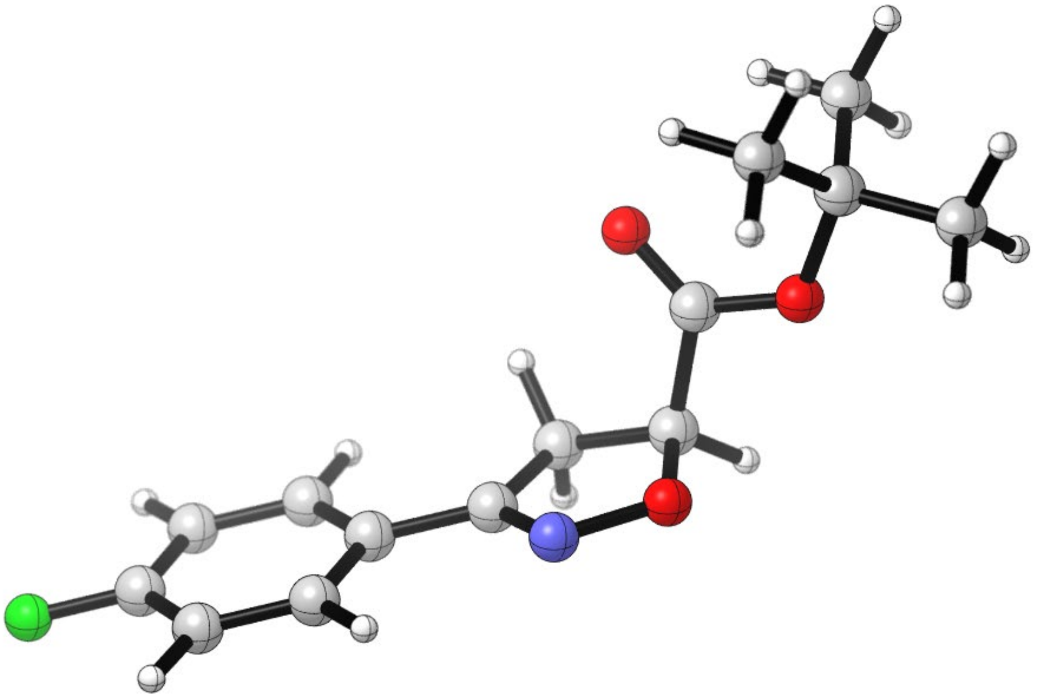

Sum of Electronic and Zero-point Energies = -922.738198 Hartree
Sum of Electronic and Thermal Energies = -922.720378 Hartree
Sum of Electronic and Thermal Enthalpies = -922.719434 Hartree
Sum of Electronic and Thermal Free Energies = -922.786719 Hartree

Dipole Moment = 3.0218 Debye

0 1

O	2.80783	2.63204	0.03436
N	2.02191	1.50887	0.22891
C	2.73381	0.53669	0.65531
C	2.14159	-0.77022	0.97677
C	2.95310	-1.81704	1.42442
C	0.76049	-0.98134	0.84649
C	2.40675	-3.05932	1.74089
H	4.02384	-1.67041	1.52738
C	0.20267	-2.21362	1.15579
H	0.13332	-0.16708	0.49948
C	1.04052	-3.23012	1.59868
H	3.01934	-3.88312	2.08874
H	-0.86123	-2.39991	1.06203
C	4.84652	2.31450	-1.18392
O	5.25319	1.33280	-1.76153
O	4.86794	3.56128	-1.64029
C	5.30363	3.86456	-3.00572
C	4.41094	3.13336	-4.00396
H	4.63665	3.49285	-5.01230
H	4.57366	2.05537	-3.97233

H	3.35912	3.34468	-3.79023
C	5.09649	5.37123	-3.09777
H	5.70068	5.88630	-2.34595
H	5.39214	5.72595	-4.08884
H	4.04537	5.62124	-2.93201
C	6.77787	3.50744	-3.17044
H	7.12486	3.86788	-4.14352
H	7.37302	3.99676	-2.39344
H	6.93484	2.42947	-3.11926
F	0.50066	-4.42616	1.90049
C	4.19337	0.88680	0.81683
H	4.84293	0.20927	0.25591
H	4.48337	0.87722	1.87181
C	4.19992	2.28160	0.20485
H	4.65141	3.05389	0.83122

p-H-TS-endo-regio-isomer

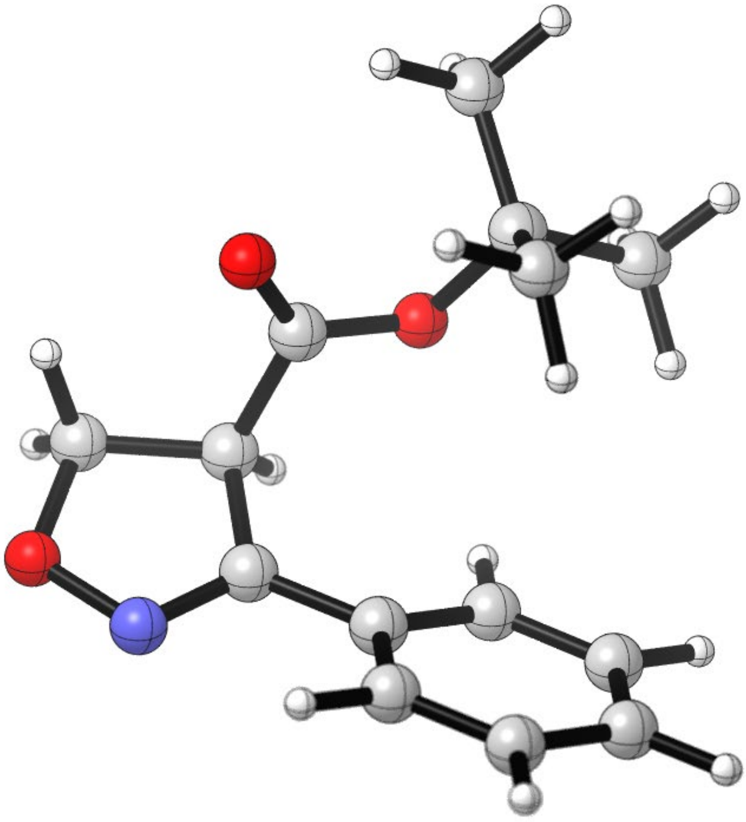

Sum of Electronic and Zero-point Energies = -823.518785 Hartree
Sum of Electronic and Thermal Energies = -823.501844 Hartree
Sum of Electronic and Thermal Enthalpies = -823.500899 Hartree
Sum of Electronic and Thermal Free Energies = -823.565076 Hartree

Dipole Moment = 4.8481 Debye

0 1

O	4.04422	1.52595	-2.07951
N	3.34014	0.41055	-2.45357
C	3.69777	-0.60828	-1.77022
C	3.07535	-1.92877	-1.97351
C	3.79206	-3.10407	-1.72057
C	1.75160	-2.01453	-2.42420
C	3.19500	-4.34664	-1.92082
H	4.81394	-3.04892	-1.35887
C	1.15833	-3.25732	-2.61880
H	1.19986	-1.09874	-2.60954
C	1.87695	-4.42674	-2.36635
H	3.76107	-5.25285	-1.72773
H	0.13045	-3.31431	-2.96253
H	1.41044	-5.39543	-2.51509
C	4.27705	-0.67908	0.64309
O	3.58217	0.03300	1.32726
O	4.66940	-1.91611	0.94745
C	4.08387	-2.63787	2.08107
C	2.57081	-2.72657	1.89920
H	2.16159	-3.40348	2.65514
H	2.09549	-1.75103	2.01068
H	2.33527	-3.13143	0.90889
C	4.72000	-4.01752	1.96191
H	5.80947	-3.94332	2.02172
H	4.36694	-4.66023	2.77288
H	4.44822	-4.47752	1.00713
C	4.48107	-1.96429	3.39059
H	4.15516	-2.59040	4.22673
H	5.56884	-1.85933	3.44570
H	4.01990	-0.98087	3.48636
C	4.77775	-0.28759	-0.74313
H	5.70093	-0.83327	-0.95990
C	4.88001	1.22435	-0.94130
H	5.89044	1.56289	-1.17409
H	4.48351	1.75808	-0.07368

p-Me-product-endo

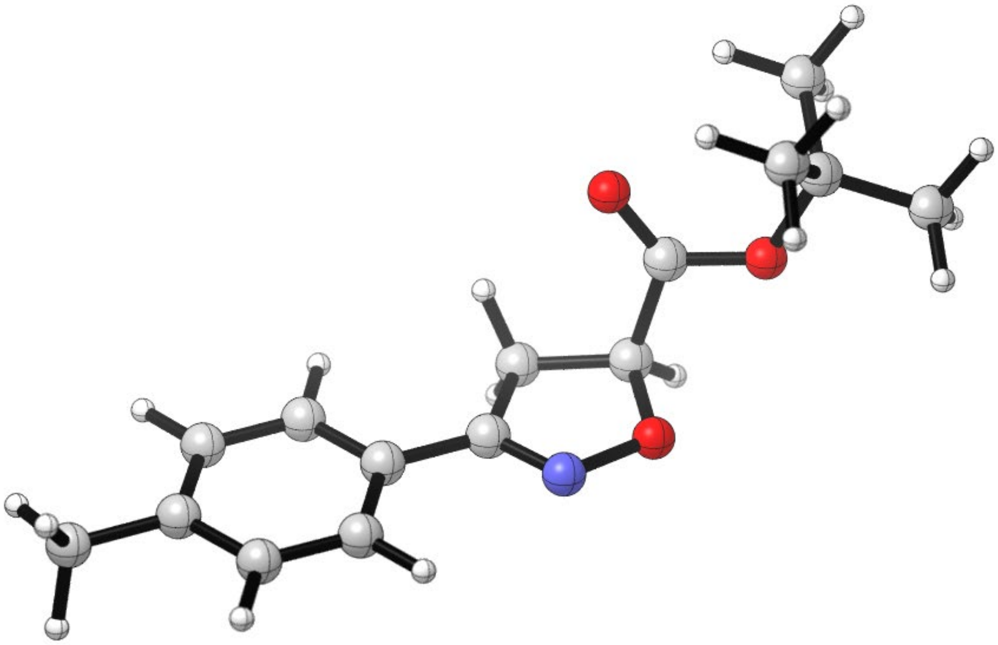

Sum of Electronic and Zero-point Energies = -862.790354 Hartree
Sum of Electronic and Thermal Energies = -862.771416 Hartree
Sum of Electronic and Thermal Enthalpies = -862.770472 Hartree
Sum of Electronic and Thermal Free Energies = -862.841206 Hartree

Dipole Moment = 2.0426 Debye

0 1

O	2.75022	2.61589	-0.01192
N	1.98341	1.48223	0.20670
C	2.71009	0.53563	0.66521
C	2.14416	-0.77466	1.01745
C	2.96998	-1.79048	1.50182
C	0.76999	-1.02596	0.88250
C	2.43727	-3.03312	1.84485
H	4.03692	-1.61955	1.61223
C	0.25058	-2.26456	1.22442
H	0.12456	-0.23827	0.50772
C	1.07381	-3.29100	1.71174
H	3.09565	-3.81218	2.21957
H	-0.81585	-2.44608	1.11436
C	4.81451	2.32288	-1.19043
O	5.24968	1.34113	-1.74665
O	4.81921	3.56297	-1.66760
C	5.26841	3.85127	-3.03105
C	4.40387	3.08720	-4.02959
H	4.63657	3.43492	-5.04051
H	4.58660	2.01322	-3.97814

H	3.34539	3.28136	-3.83322
C	5.03459	5.35212	-3.15185
H	5.61787	5.89094	-2.40010
H	5.33808	5.69592	-4.14444
H	3.97666	5.58482	-3.00530
C	6.75139	3.51985	-3.16998
H	7.10504	3.87094	-4.14412
H	7.32621	4.03305	-2.39306
H	6.92782	2.44608	-3.09850
C	0.48757	-4.63124	2.07358
H	-0.29268	-4.52467	2.83351
H	0.03152	-5.10629	1.19929
H	1.25259	-5.30575	2.46468
C	4.16162	0.91858	0.82798
H	4.44502	0.93584	1.88455
H	4.82690	0.24071	0.28629
C	4.14652	2.30036	0.18790
H	4.57074	3.09477	0.80591

p-NO2-product-endo

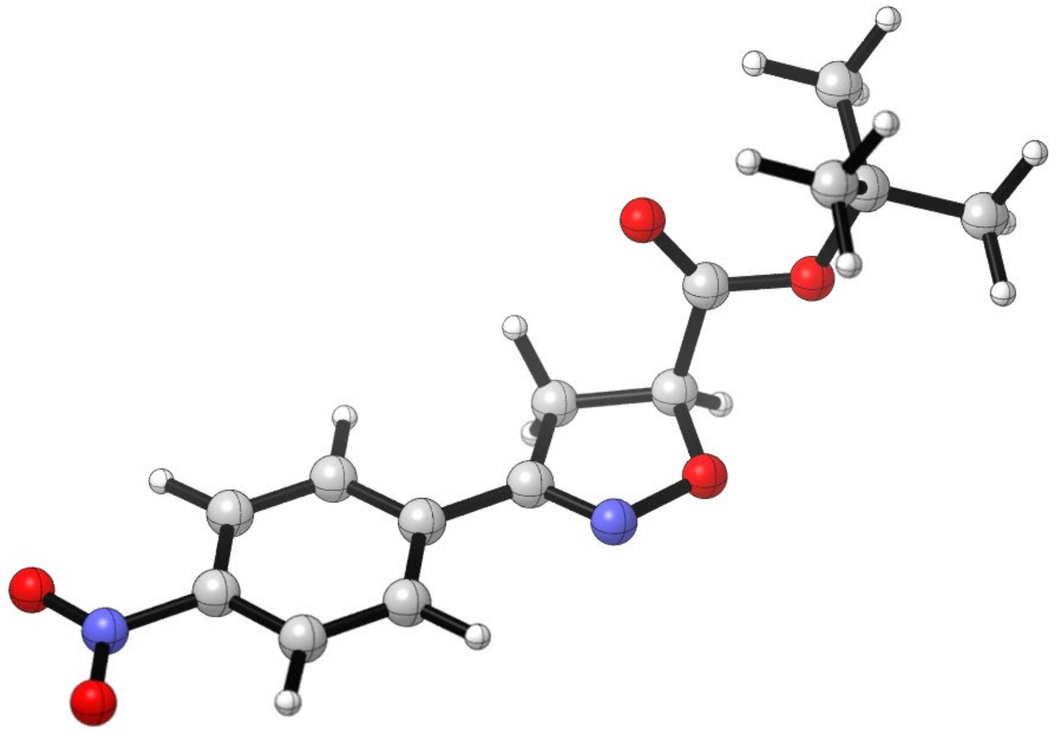

Sum of Electronic and Zero-point Energies = -1027.948682 Hartree
Sum of Electronic and Thermal Energies = -1027.929154 Hartree
Sum of Electronic and Thermal Enthalpies = -1027.928210 Hartree
Sum of Electronic and Thermal Free Energies = -1028.000136 Hartree

Dipole Moment = 6.5192 Debye

0 1

O	2.74841	2.58317	-0.00317
N	1.99142	1.45582	0.22346
C	2.72370	0.50793	0.67094
C	2.14684	-0.79883	1.02030
C	2.97598	-1.81938	1.49893
C	0.76894	-1.03048	0.88434
C	2.44713	-3.05978	1.84122
H	4.04279	-1.65111	1.60420
C	0.22930	-2.26229	1.21974
H	0.13328	-0.23429	0.51305
C	1.08205	-3.25534	1.69405
H	3.06989	-3.86410	2.21308
H	-0.83006	-2.46626	1.12301
C	4.80598	2.30609	-1.19752
O	5.22173	1.32231	-1.76464
O	4.81841	3.54925	-1.65993
C	5.26315	3.85033	-3.02480
C	4.38352	3.10737	-4.02560
H	4.61549	3.46244	-5.03399
H	4.55324	2.03068	-3.98691
H	3.32851	3.31329	-3.82262
C	5.04547	5.35482	-3.12444
H	5.63987	5.87772	-2.37027
H	5.34663	5.70729	-4.11458
H	3.99123	5.59806	-2.96885
C	6.74100	3.50274	-3.17480
H	7.09349	3.86138	-4.14647
H	7.32627	3.99982	-2.39529
H	6.90552	2.42617	-3.11813
N	0.51331	-4.56597	2.05352
O	-0.68710	-4.71641	1.91310
O	1.27867	-5.41784	2.46927
C	4.17628	0.88792	0.81928
H	4.83529	0.21640	0.26172
H	4.47470	0.89910	1.87176
C	4.15116	2.27436	0.18754
H	4.57175	3.06832	0.80773

p-OMe-product-endo

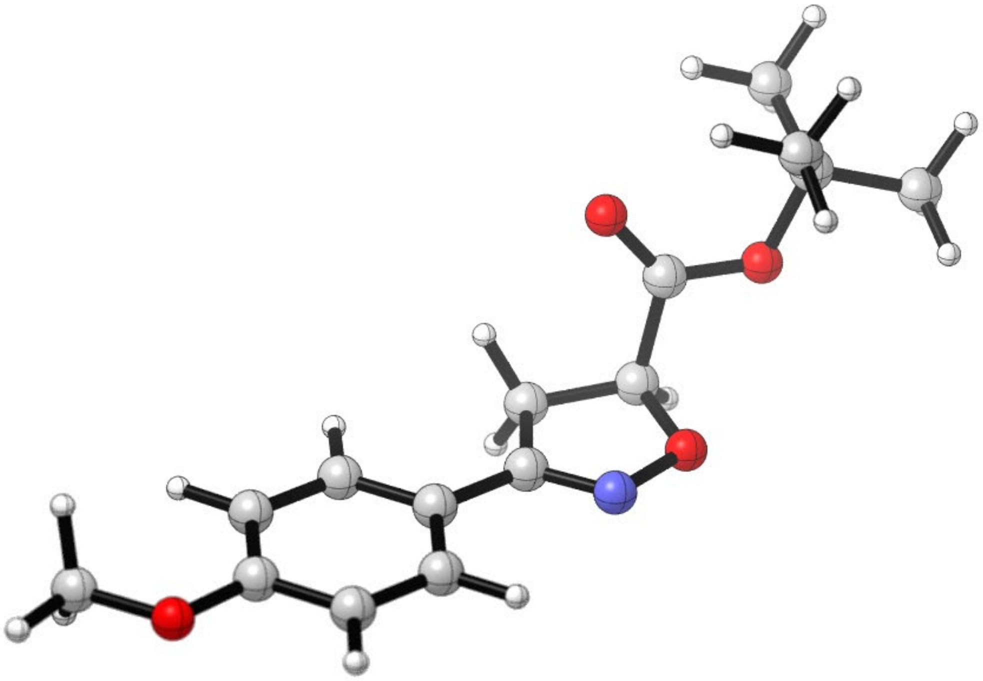

Sum of Electronic and Zero-point Energies = -937.968908 Hartree
Sum of Electronic and Thermal Energies = -937.949378 Hartree
Sum of Electronic and Thermal Enthalpies = -937.948434 Hartree
Sum of Electronic and Thermal Free Energies = -938.019554 Hartree

Dipole Moment = 3.3923 Debye

0 1

O	2.79353	2.67680	0.02065
N	2.03410	1.53644	0.24354
C	2.76662	0.60202	0.71844
C	2.21320	-0.70870	1.08044
C	3.04266	-1.71556	1.56967
C	0.83783	-0.97403	0.94867
C	2.53469	-2.96718	1.92561
H	4.10849	-1.53703	1.67816
C	0.32308	-2.20731	1.29524
H	0.18536	-0.19441	0.56948
C	1.16928	-3.21409	1.78686
H	3.20908	-3.72689	2.30104
H	-0.73488	-2.42638	1.19823
C	4.85648	2.36170	-1.16011
O	5.31606	1.37275	-1.68334
O	4.83298	3.58486	-1.67836
C	5.27726	3.83815	-3.05012
C	4.43286	3.02086	-4.02347
H	4.65949	3.33965	-5.04525
H	4.64056	1.95379	-3.93552

H	3.36972	3.19667	-3.83514
C	5.00802	5.32817	-3.22101
H	5.57753	5.90531	-2.48737
H	5.30430	5.64594	-4.22441
H	3.94472	5.54031	-3.08241
C	6.76797	3.53780	-3.17695
H	7.11486	3.86551	-4.16161
H	7.32913	4.08935	-2.41647
H	6.96968	2.47140	-3.07052
O	0.56646	-4.38903	2.09857
C	1.37715	-5.43562	2.59296
H	0.70718	-6.27476	2.77527
H	2.13717	-5.72688	1.85851
H	1.86591	-5.14724	3.53099
C	4.21249	1.00313	0.89053
H	4.89272	0.32168	0.37275
H	4.48086	1.04717	1.95038
C	4.19033	2.36999	0.21979
H	4.61573	3.17923	0.81759

p-CF3-TS-exo

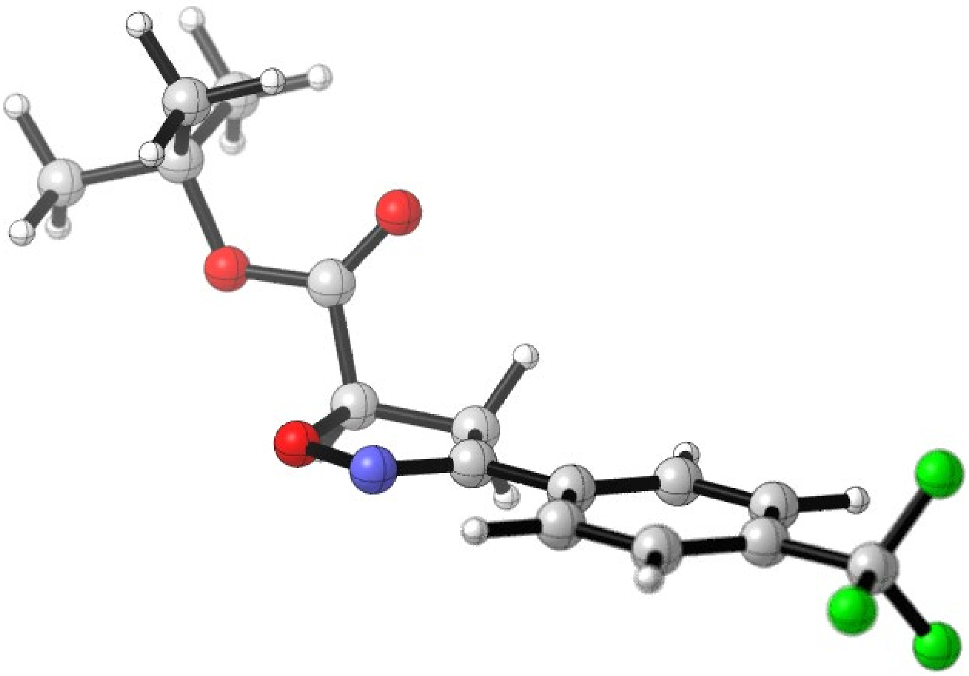

Sum of Electronic and Zero-point Energies = -1160.468150 Hartree
Sum of Electronic and Thermal Energies = -1160.447525 Hartree
Sum of Electronic and Thermal Enthalpies = -1160.446581 Hartree
Sum of Electronic and Thermal Free Energies = -1160.521889 Hartree

Dipole Moment = 4.4981 Debye

0 1			
O	4.12879	1.03809	-0.73494
N	3.19746	0.48369	0.11880
C	3.70074	-0.51184	0.74275
C	2.90449	-1.30037	1.69622
C	3.48875	-2.38230	2.36470
C	1.56101	-0.98593	1.93820
C	2.74419	-3.14045	3.26119
H	4.52945	-2.63488	2.18794
C	0.81574	-1.74033	2.83498
H	1.11258	-0.14733	1.41672
C	1.40973	-2.81652	3.49301
H	3.19728	-3.98017	3.77898
H	-0.22429	-1.49821	3.02298
C	6.31564	1.45596	0.13990
O	6.67612	1.39670	1.29263
O	6.60141	2.41918	-0.72748
C	7.32580	3.62492	-0.31430
C	8.73509	3.25320	0.13700
H	9.30744	4.17014	0.30647
H	8.71831	2.67435	1.06110
H	9.24045	2.67515	-0.64263
C	7.36520	4.44876	-1.59522
H	6.35069	4.66974	-1.93691
H	7.88964	5.39072	-1.41343
H	7.88871	3.90253	-2.38462
C	6.53659	4.34893	0.77222
H	6.99856	5.32314	0.95752
H	5.50712	4.51339	0.44091
H	6.52736	3.78365	1.70495
C	0.63363	-3.64389	4.47869
F	-0.66051	-3.29375	4.53524
F	1.13121	-3.52808	5.72510
F	0.67974	-4.95456	4.17269
C	5.41626	0.40910	-0.52478
H	5.81512	0.15801	-1.50959
C	5.13858	-0.78741	0.37656
H	5.23632	-1.74734	-0.13911
H	5.79227	-0.77814	1.25332

p-Cl-TS-exo

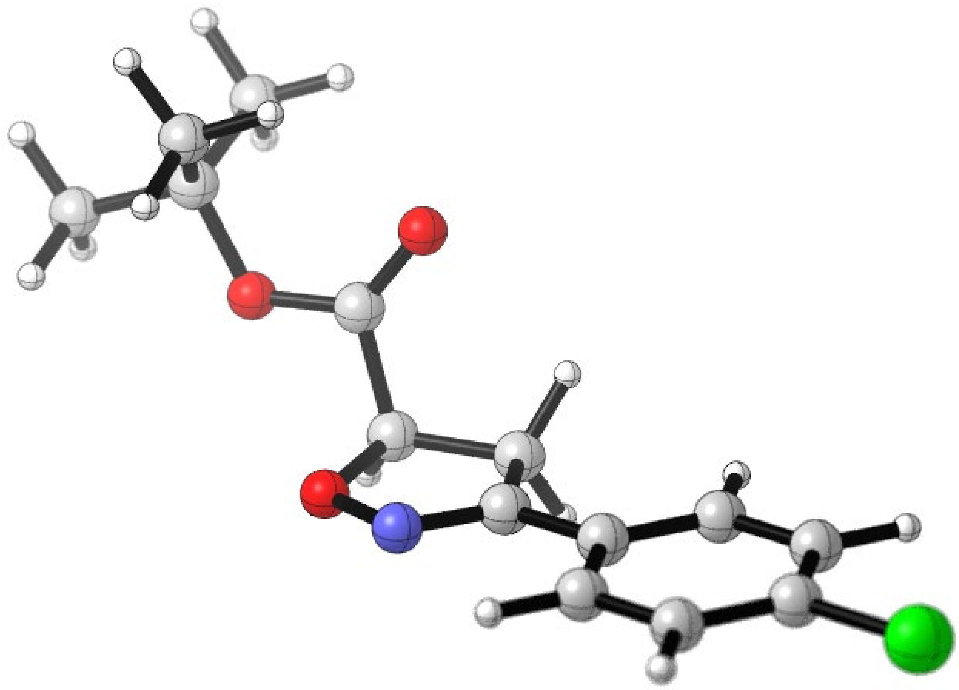

Sum of Electronic and Zero-point Energies = -1283.096762 Hartree
Sum of Electronic and Thermal Energies = -1283.078539 Hartree
Sum of Electronic and Thermal Enthalpies = -1283.077595 Hartree
Sum of Electronic and Thermal Free Energies = -1283.146359 Hartree

Dipole Moment = 3.2819 Debye

0 1

O	4.14215200	1.02258300	-0.72483000
N	3.20006200	0.49243900	0.13741600
C	3.68671600	-0.50660300	0.76899100
C	2.88247800	-1.27560500	1.72988400
C	3.44898000	-2.35478300	2.41332900
C	1.54099200	-0.94498000	1.96941600
C	2.69834900	-3.09749200	3.32200600
H	4.48695000	-2.62432700	2.24374100
C	0.78503800	-1.67681200	2.87362100
H	1.10003200	-0.10769700	1.43891000
C	1.37169800	-2.75025700	3.54392800
H	3.13716700	-3.93432800	3.85368800
H	-0.25234900	-1.42443900	3.06318100
C	6.33228300	1.41187700	0.15638900
O	6.69604500	1.35032200	1.30805200
O	6.62921300	2.37051200	-0.71314600
C	7.37013100	3.56642000	-0.30320900
C	8.77625000	3.17720400	0.14348700
H	9.36092300	4.08681800	0.31054800
H	8.75510100	2.59897000	1.06787800
H	9.27138700	2.59225700	-0.63756100
C	7.41598900	4.38923900	-1.58467300
H	6.40320500	4.62310600	-1.92291900

H	7.95333100	5.32439300	-1.40528100
H	7.92943500	3.83572400	-2.37561400
C	6.59449400	4.30189300	0.78551700
H	7.06940200	5.27049900	0.96761300
H	5.56584200	4.47859200	0.45800900
H	6.58170700	3.73785100	1.71893400
Cl	0.42303100	-3.66917200	4.67982000
C	5.11896500	-0.81042600	0.40191200
H	5.77408900	-0.81020100	1.27755000
H	5.19693400	-1.77447600	-0.10949700
C	5.41794100	0.37619500	-0.50524400
H	5.81792100	0.11285200	-1.48655000

p-CN-TS-exo

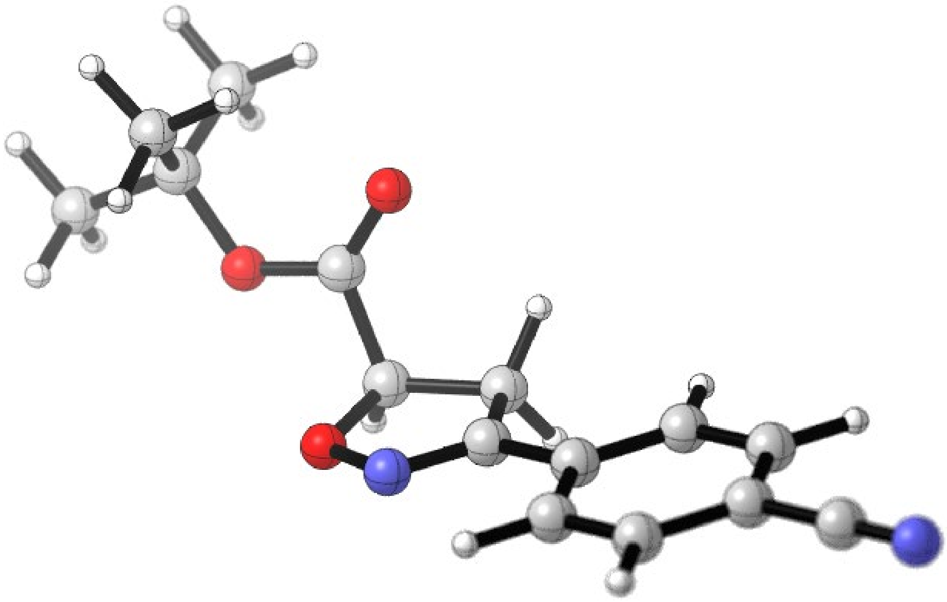

Sum of Electronic and Zero-point Energies = -915.735598 Hartree
Sum of Electronic and Thermal Energies = -915.716784 Hartree
Sum of Electronic and Thermal Enthalpies = -915.715840 Hartree
Sum of Electronic and Thermal Free Energies = -915.785457 Hartree

Dipole Moment = 6.1799 Debye

0 1

O	4.14998	1.05743	-0.72518
N	3.21836	0.51410	0.13264
C	3.71380	-0.48693	0.75473
C	2.91193	-1.26390	1.71148
C	3.48541	-2.34949	2.38130
C	1.57051	-0.93237	1.95594
C	2.73779	-3.09923	3.28275

H	4.52301	-2.61341	2.20322
C	0.81992	-1.67351	2.85372
H	1.13121	-0.08960	1.43346
C	1.40312	-2.76149	3.51981
H	3.18126	-3.94100	3.80318
H	-0.21709	-1.42102	3.04659
C	6.35001	1.44866	0.12903
O	6.71508	1.38919	1.28029
O	6.64513	2.40258	-0.74482
C	7.39269	3.59856	-0.34324
C	8.79850	3.20505	0.10019
H	9.38752	4.11294	0.26084
H	8.77852	2.63156	1.02757
H	9.28851	2.61449	-0.67984
C	7.43682	4.41450	-1.62899
H	6.42382	4.65102	-1.96469
H	7.97846	5.34834	-1.45609
H	7.94536	3.85528	-2.41905
C	6.62348	4.34147	0.74478
H	7.10315	5.30870	0.92131
H	5.59462	4.52168	0.41987
H	6.61152	3.78170	1.68079
C	0.62320	-3.53384	4.45200
N	-0.00391	-4.15534	5.20082
C	5.14630	-0.77917	0.38137
H	5.80531	-0.77355	1.25425
H	5.23126	-1.74153	-0.13196
C	5.43156	0.41177	-0.52524
H	5.81762	0.15338	-1.51319

p-F-TS-exo

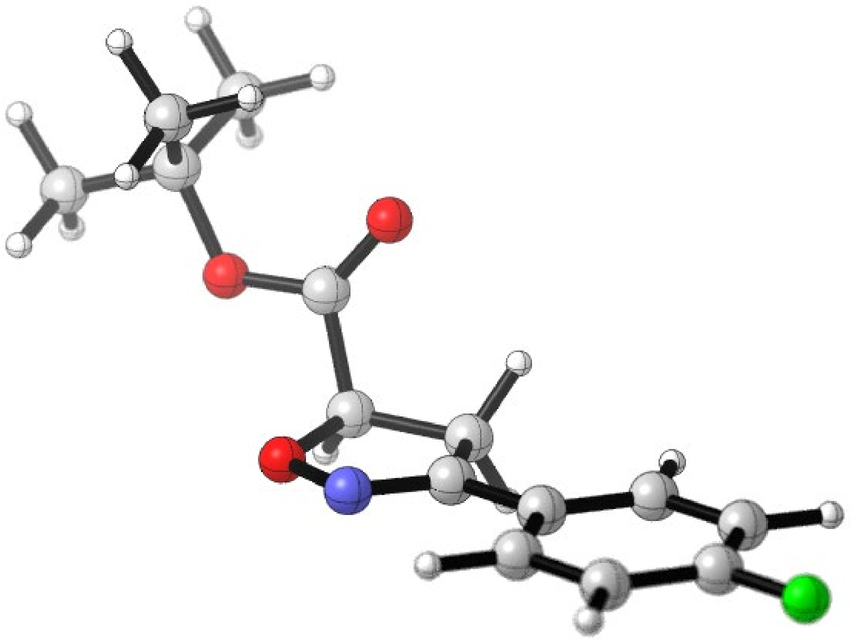

Sum of Electronic and Zero-point Energies = -922.738186 Hartree
Sum of Electronic and Thermal Energies = -922.720368 Hartree
Sum of Electronic and Thermal Enthalpies = -922.719424 Hartree
Sum of Electronic and Thermal Free Energies = -922.786593 Hartree

Dipole Moment = 3.0893 Debye

0 1

O	4.11585	1.04128	-0.71688
N	3.17934	0.50629	0.15071
C	3.67354	-0.49069	0.77937
C	2.87910	-1.26608	1.74352
C	3.45805	-2.34089	2.42488
C	1.53499	-0.94473	1.98651
C	2.71761	-3.09137	3.33613
H	4.49773	-2.60005	2.25033
C	0.78594	-1.68170	2.89272
H	1.08834	-0.11056	1.45600
C	1.39505	-2.74374	3.55031
H	3.14880	-3.92750	3.87453
H	-0.25355	-1.45129	3.09709
C	6.30791	1.44042	0.15197
O	6.67015	1.38743	1.30456
O	6.60510	2.39383	-0.72359
C	7.34303	3.59358	-0.32039
C	8.74881	3.21010	0.13242
H	9.33155	4.12192	0.29419
H	8.72641	2.63850	1.06089
H	9.24674	2.62031	-0.64321
C	7.39069	4.40749	-1.60750
H	6.37833	4.63716	-1.94988

H	7.92598	5.34484	-1.43343
H	7.90702	3.84927	-2.39325
C	6.56360	4.33553	0.76123
H	7.03643	5.30624	0.93758
H	5.53542	4.50800	0.43002
H	6.54947	3.77808	1.69858
F	0.66921	-3.46080	4.42895
C	5.39491	0.40015	-0.50380
H	5.79102	0.13743	-1.48688
C	5.10589	-0.78693	0.40587
H	5.76456	-0.78244	1.27885
H	5.18679	-1.75114	-0.10481

p-Me-TS-exo

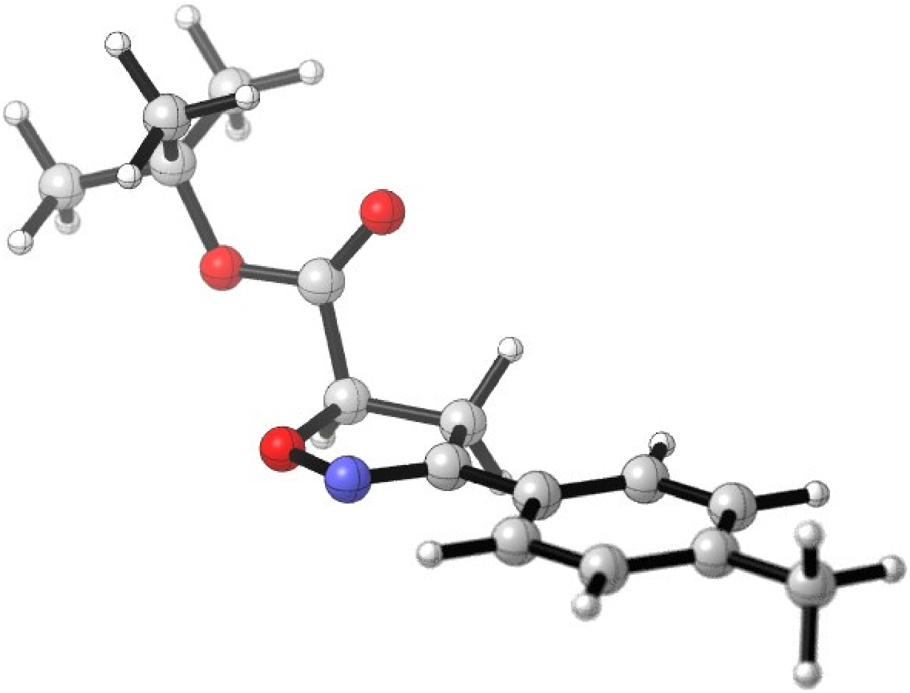

Sum of Electronic and Zero-point Energies = -862.790296 Hartree
Sum of Electronic and Thermal Energies = -862.771429 Hartree
Sum of Electronic and Thermal Enthalpies = -862.770485 Hartree
Sum of Electronic and Thermal Free Energies = -862.840238 Hartree

Dipole Moment = 2.0678 Debye

0 1

O	4.05433	1.17052	-0.67575
N	3.15137	0.60559	0.21107
C	3.64579	-0.44935	0.73725
C	2.88568	-1.26708	1.69369

C	3.46097	-2.40658	2.25853
C	1.57182	-0.92485	2.04935
C	2.74146	-3.19012	3.16072
H	4.47728	-2.68944	1.99984
C	0.86583	-1.70883	2.94807
H	1.12057	-0.04057	1.61127
C	1.43687	-2.85555	3.52053
H	3.20597	-4.07335	3.59081
H	-0.15110	-1.43190	3.21544
C	6.30535	1.43729	0.09033
O	6.74871	1.27768	1.20410
O	6.56722	2.45681	-0.72076
C	7.36255	3.60081	-0.27047
C	8.78649	3.15186	0.04502
H	9.40292	4.03447	0.24073
H	8.81465	2.50251	0.92072
H	9.21260	2.62135	-0.81195
C	7.34116	4.52358	-1.48278
H	6.31351	4.80237	-1.72995
H	7.91183	5.43117	-1.26791
H	7.78595	4.02606	-2.34897
C	6.68110	4.26129	0.92436
H	7.19076	5.20305	1.14875
H	5.63709	4.48277	0.68430
H	6.71779	3.62428	1.80890
C	5.04117	-0.76384	0.25324
H	5.05335	-1.68896	-0.33067
H	5.75085	-0.84882	1.08070
C	5.31982	0.47782	-0.58342
H	5.65223	0.27662	-1.60409
C	0.65173	-3.69055	4.49887
H	0.39205	-3.10794	5.38835
H	-0.28350	-4.04115	4.05167
H	1.22239	-4.56428	4.82152

p-NO2-TS-exo

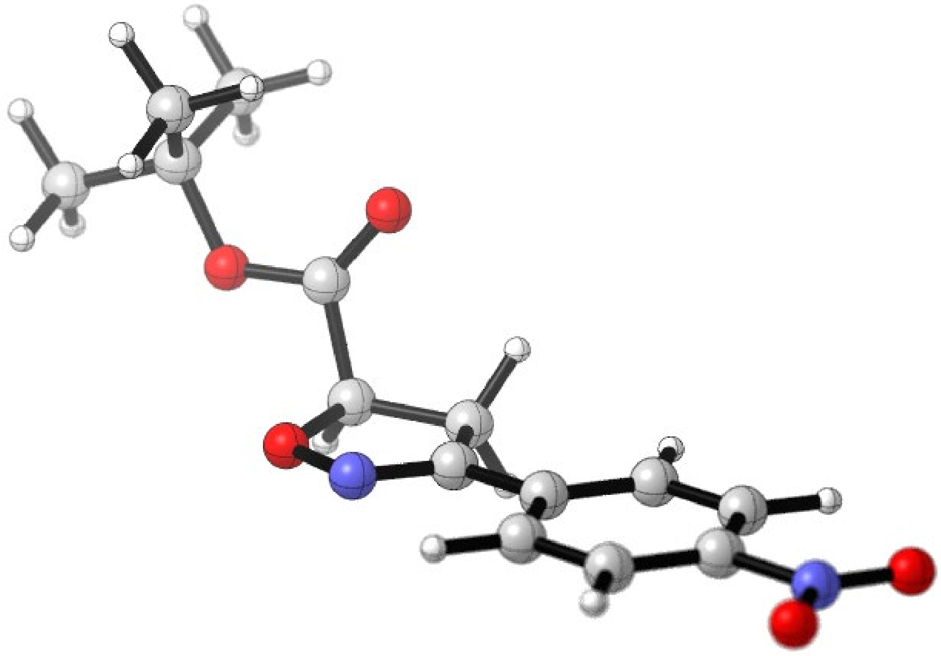

Sum of Electronic and Zero-point Energies = -1027.948747 Hartree
Sum of Electronic and Thermal Energies = -1027.929195 Hartree
Sum of Electronic and Thermal Enthalpies = -1027.928251 Hartree
Sum of Electronic and Thermal Free Energies = -1028.000160 Hartree

Dipole Moment = 6.5556 Debye

0 1

O	3.81024	2.08647	0.42519
N	3.16100	1.31126	1.35879
C	3.31616	0.06967	1.09594
C	2.69383	-0.96830	1.93120
C	1.89543	-0.61644	3.03099
C	2.89185	-2.31903	1.62412
C	1.30598	-1.59859	3.81134
H	1.74659	0.43248	3.26208
C	2.30443	-3.31492	2.39781
H	3.51006	-2.60102	0.77807
C	1.52217	-2.93275	3.47728
H	0.68651	-1.35312	4.66528
H	2.44419	-4.36634	2.17883
C	6.05478	1.50412	-0.16270
O	6.77784	0.70898	0.39139
O	6.38600	2.72107	-0.57392
C	7.70514	3.29194	-0.28162
C	8.78754	2.47632	-0.98213
H	9.74657	2.99164	-0.87391
H	8.87598	1.47790	-0.55243
H	8.56450	2.39212	-2.05004
C	7.60562	4.68988	-0.87884
H	6.79683	5.25046	-0.40300

H	8.54562	5.22615	-0.72357
H	7.40740	4.63341	-1.95263
C	7.91026	3.36028	1.22849
H	8.81895	3.93237	1.43762
H	7.06565	3.87207	1.69892
H	8.01573	2.36611	1.66440
N	0.89605	-3.98060	4.30214
O	1.09747	-5.14053	3.98851
O	0.21533	-3.62213	5.24630
C	4.15149	-0.18628	-0.13402
H	5.02088	-0.81262	0.08590
H	3.55752	-0.65406	-0.92462
C	4.57693	1.23882	-0.46649
H	4.35004	1.55823	-1.48549

p-OMe-TS-exo

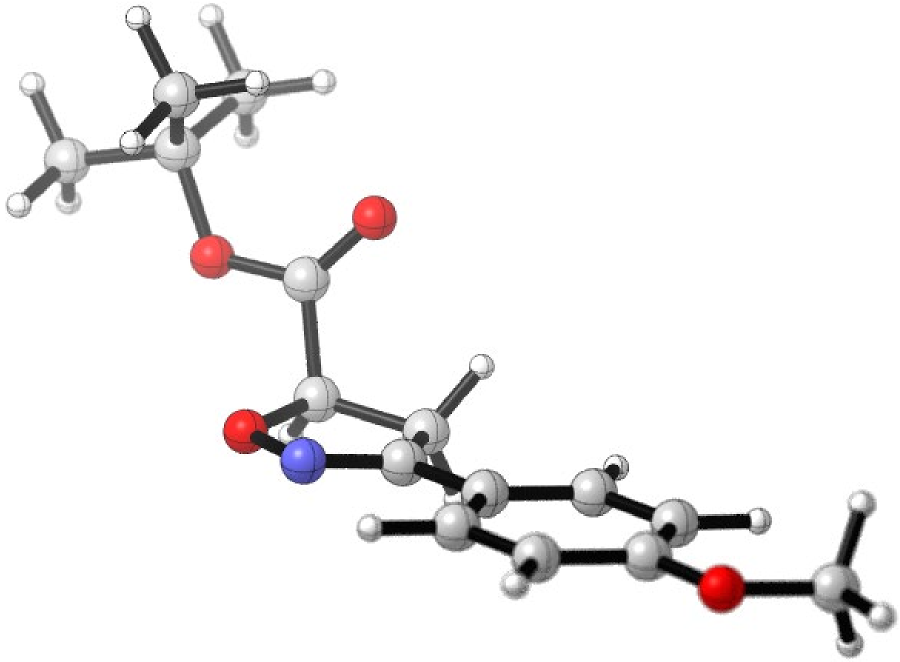

Sum of Electronic and Zero-point Energies = -937.968878 Hartree
Sum of Electronic and Thermal Energies = -937.949362 Hartree
Sum of Electronic and Thermal Enthalpies = -937.948418 Hartree
Sum of Electronic and Thermal Free Energies = -938.019318 Hartree

Dipole Moment = 3.4242 Debye

0 1

O	4.15960	1.06005	-0.80756
N	3.23660	0.49605	0.06226
C	3.74449	-0.51987	0.64967
C	2.97486	-1.33003	1.60181

C	3.56427	-2.41800	2.24213
C	1.62903	-1.03147	1.88285
C	2.84755	-3.20511	3.14658
H	4.60269	-2.66771	2.04466
C	0.90982	-1.80012	2.77617
H	1.16274	-0.18626	1.38736
C	1.51501	-2.89404	3.41524
H	3.33826	-4.04260	3.62690
H	-0.12805	-1.58116	3.00331
C	6.35116	1.44576	0.07798
O	6.75246	1.32629	1.21270
O	6.60078	2.46304	-0.73970
C	7.32078	3.65071	-0.27623
C	8.74905	3.27524	0.10820
H	9.31413	4.18906	0.31536
H	8.76899	2.64098	0.99513
H	9.23684	2.75290	-0.72045
C	7.30922	4.55209	-1.50467
H	6.28141	4.77783	-1.80050
H	7.82699	5.48902	-1.28188
H	7.81396	4.06246	-2.34201
C	6.55726	4.29685	0.87610
H	7.01153	5.26548	1.10507
H	5.51550	4.46405	0.58733
H	6.58517	3.67646	1.77271
O	0.72402	-3.58492	4.27434
C	1.28908	-4.69290	4.94528
H	2.13618	-4.38614	5.56991
H	0.49915	-5.09708	5.57684
H	1.61736	-5.45985	4.23397
C	5.17015	-0.79994	0.23722
H	5.23760	-1.73937	-0.31968
H	5.84518	-0.83849	1.09636
C	5.44458	0.42761	-0.62101
H	5.84469	0.21159	-1.61407

p-CF3--oximyl-radical-cyclisation-tBu-P

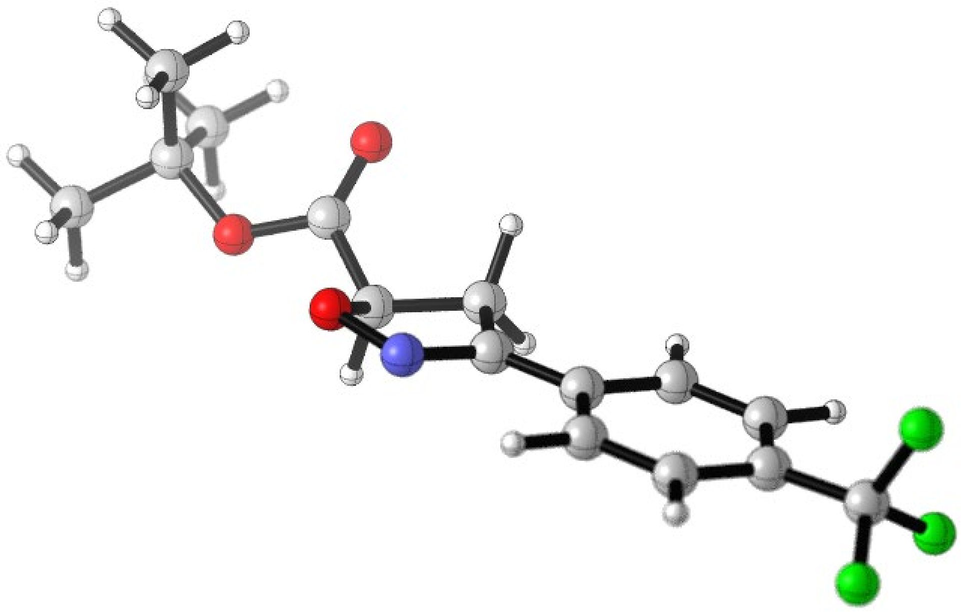

```
Sum of Electronic and Zero-point Energies = -1160.497200 Hartree
Sum of Electronic and Thermal Energies = -1160.476139 Hartree
Sum of Electronic and Thermal Enthalpies = -1160.475195 Hartree
Sum of Electronic and Thermal Free Energies = -1160.550890 Hartree

Dipole Moment = 13.2316 Debye

-1 2
C                1.59369     -0.42728     -0.70369
C                0.35186     -1.02356     -0.06838
C               -0.59884      0.14354     -0.24180
H                1.66355     -0.68300     -1.77293
H                0.52180     -1.26428      0.98838
H                0.01796     -1.92994     -0.58139
N                0.03921      1.27548     -0.52301
O                1.44078      0.98276     -0.56674
C                2.90596     -0.77680     -0.02366
O                3.01990     -1.35596      1.03230
O                3.93715     -0.32589     -0.75712
C                5.29400     -0.36752     -0.24443
C               -2.00243      0.04983     -0.10364
C               -2.65437     -1.18241      0.23372
C               -2.85267      1.19569     -0.29161
C               -4.02221     -1.26646      0.35688
H               -2.05663     -2.07467      0.40229
C               -4.21674      1.10135     -0.16726
H               -2.38823      2.14390     -0.54271
C               -4.84170     -0.13178      0.14560
H               -4.48295     -2.21720      0.61257
H               -4.83317      1.98366     -0.32076
C                5.38990      0.43189      1.05394
H                4.85930     -0.06585      1.86626
```

H	6.44334	0.54329	1.33120
H	4.95938	1.42686	0.90734
C	5.75323	-1.81396	-0.06709
H	5.60896	-2.36815	-0.99996
H	6.82030	-1.82607	0.17899
H	5.19793	-2.30785	0.73084
C	6.09556	0.31505	-1.34857
H	7.15528	0.34795	-1.07786
H	5.98726	-0.23302	-2.28895
H	5.73368	1.33557	-1.49936
C	-6.29117	-0.20701	0.36342
F	-6.99400	0.70207	-0.36217
F	-6.69600	0.01834	1.65974
F	-6.81882	-1.42530	0.06593

p-CF3--oximyl-radical-cyclisation-tBu-TS

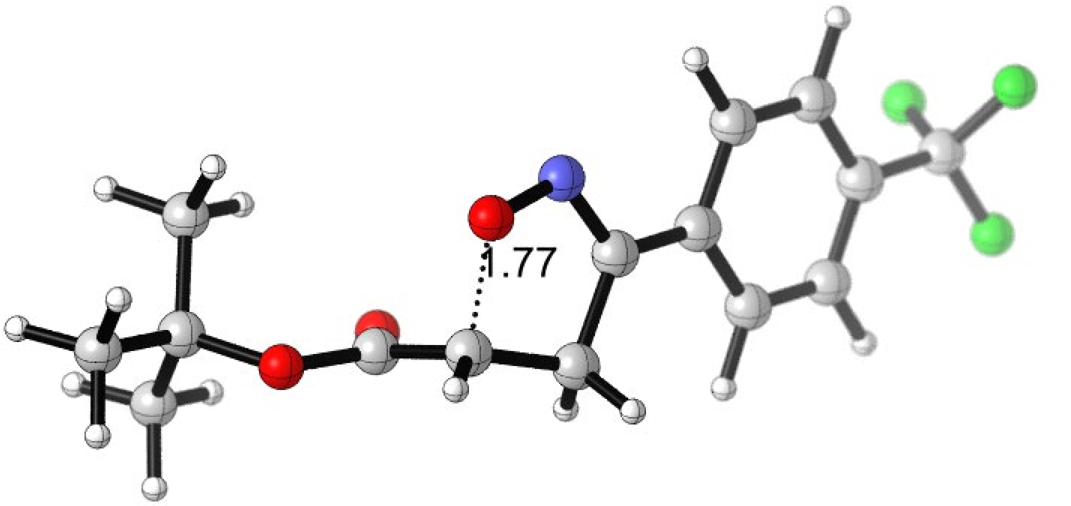

Sum of Electronic and Zero-point Energies = -1160.471755 Hartree
Sum of Electronic and Thermal Energies = -1160.450929 Hartree
Sum of Electronic and Thermal Enthalpies = -1160.449985 Hartree
Sum of Electronic and Thermal Free Energies = -1160.525594 Hartree

Dipole Moment = 3.7573 Debye

-1 2

C	-1.84446	0.21569	1.07814
C	-0.43786	-0.24266	1.36455
C	0.43788	0.50307	0.37599
H	-2.42387	0.66017	1.88112
H	-0.35341	-1.32130	1.19541

H	-0.15204	-0.02227	2.40191
N	-0.16985	1.48835	-0.23763
O	-1.37775	1.70914	0.25338
C	-2.60756	-0.56302	0.13386
O	-2.14236	-1.34246	-0.70430
O	-3.96124	-0.27541	0.21320
C	-4.82056	-0.59049	-0.89131
C	1.79111	0.13740	0.04422
C	2.41615	-0.99324	0.61837
C	2.56834	0.93243	-0.84079
C	3.73396	-1.31719	0.32899
H	1.85693	-1.62327	1.30246
C	3.87629	0.60413	-1.13298
H	2.10466	1.80729	-1.28349
C	4.47844	-0.52218	-0.54664
H	4.19084	-2.18938	0.78616
H	4.45181	1.22385	-1.81596
C	-4.98978	-2.10477	-1.04286
H	-5.75032	-2.31819	-1.80333
H	-4.04478	-2.56601	-1.33075
H	-5.32015	-2.53532	-0.09138
C	-4.29940	0.05159	-2.17919
H	-5.05039	-0.04829	-2.97137
H	-4.10638	1.11495	-2.00573
H	-3.36993	-0.42101	-2.49939
C	-6.15278	0.04399	-0.49357
H	-6.90915	-0.14241	-1.26328
H	-6.50128	-0.37767	0.45445
H	-6.03091	1.12383	-0.36785
C	5.88050	-0.86228	-0.89823
F	6.41184	-1.81112	-0.09701
F	6.71479	0.20568	-0.83192
F	6.01907	-1.33080	-2.16733

p-CF3--oximyl-radical-Nu-attack-tBu-P

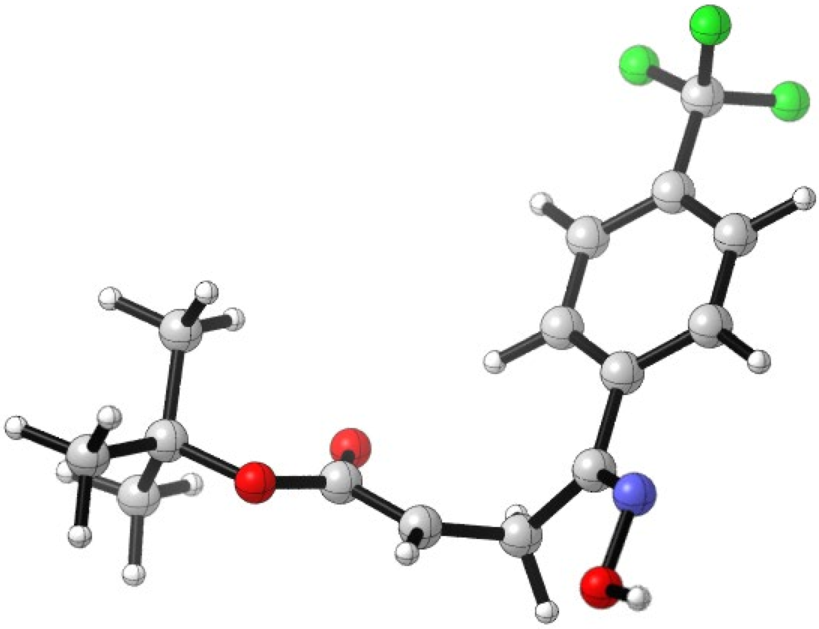

Sum of Electronic and Zero-point Energies = -1161.015386 Hartree
Sum of Electronic and Thermal Energies = -1160.993615 Hartree
Sum of Electronic and Thermal Enthalpies = -1160.992671 Hartree
Sum of Electronic and Thermal Free Energies = -1161.069570 Hartree

Dipole Moment = 4.5464 Debye

0 2

C	3.81641	-0.78545	-0.51774
C	2.96114	0.29372	-0.36502
C	1.64132	0.09696	0.07176
C	1.19860	-1.19836	0.35463
C	2.05855	-2.28552	0.19756
C	3.36118	-2.07621	-0.23580
H	4.83892	-0.63249	-0.84948
H	3.30290	1.30054	-0.57670
H	0.18009	-1.37695	0.68784
H	1.71073	-3.28959	0.41312
C	0.72183	1.25438	0.21739
N	1.02455	2.31594	-0.42984
O	0.08957	3.32277	-0.24270
H	0.44275	4.05643	-0.75924
C	-0.53052	1.15144	1.07210
C	-1.73055	0.92146	0.22000
H	-2.05893	1.68567	-0.47394
H	-0.42974	0.34517	1.79980
H	-0.63773	2.10303	1.60507
C	-2.45095	-0.34106	0.27845
O	-2.11959	-1.27609	0.99387
O	-3.51065	-0.34181	-0.54640
C	-4.37505	-1.51204	-0.66158
C	-5.40746	-1.06833	-1.69184

H	-6.12790	-1.87205	-1.86649
H	-5.94543	-0.18536	-1.33618
H	-4.91942	-0.82190	-2.63874
C	-5.04507	-1.80330	0.67903
H	-4.32116	-2.14062	1.42137
H	-5.54936	-0.90549	1.04913
H	-5.79772	-2.58523	0.53952
C	-3.57724	-2.70559	-1.18165
H	-3.04726	-2.43174	-2.09898
H	-2.85729	-3.05697	-0.44198
H	-4.26778	-3.52132	-1.41620
C	4.31370	-3.22373	-0.41436
F	5.42110	-3.07308	0.33798
F	4.73491	-3.32594	-1.69022
F	3.76673	-4.40369	-0.08304

p-CF3--oximyl-radical-Nu-attack-tBu-TS

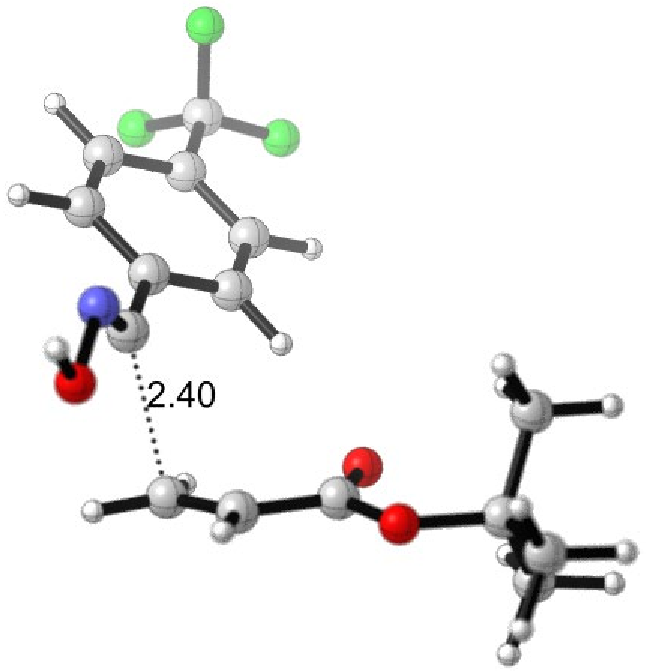

Sum of Electronic and Zero-point Energies = -1160.957307 Hartree
Sum of Electronic and Thermal Energies = -1160.934651 Hartree
Sum of Electronic and Thermal Enthalpies = -1160.933707 Hartree
Sum of Electronic and Thermal Free Energies = -1161.013643 Hartree

Dipole Moment = 4.0617 Debye

0 2

C	-2.38516	-2.16552	0.06247
C	-1.43007	-1.15293	0.07718
C	-1.83489	0.18471	-0.02759
C	-3.20214	0.50315	-0.11552

C	-4.14794	-0.51019	-0.12443
C	-3.73529	-1.84185	-0.03701
H	-2.07941	-3.20325	0.13406
H	-0.37404	-1.39410	0.16872
H	-3.50287	1.54311	-0.18386
H	-5.20508	-0.27399	-0.19937
C	-0.85340	1.25046	0.00386
N	-0.72031	2.25079	-0.72139
O	0.26762	3.15902	-0.33727
H	0.23350	3.82715	-1.03350
C	0.94705	0.95957	1.56129
C	2.04712	0.83572	0.79082
H	2.62037	1.69509	0.46292
H	0.66116	1.92489	1.96422
H	0.44423	0.07320	1.93846
C	2.47462	-0.49749	0.31472
O	1.84191	-1.52302	0.48945
O	3.64162	-0.42208	-0.33709
C	4.27174	-1.61023	-0.90500
C	3.37036	-2.22168	-1.97501
H	3.92256	-3.00677	-2.50048
H	2.47116	-2.65651	-1.53786
H	3.08308	-1.45861	-2.70477
C	5.54361	-1.05042	-1.53216
H	6.16520	-0.57223	-0.77028
H	6.11599	-1.85784	-1.99695
H	5.29687	-0.30896	-2.29700
C	4.61181	-2.60386	0.20301
H	5.21506	-3.41423	-0.21741
H	5.19888	-2.10942	0.98285
H	3.71132	-3.02895	0.64716
C	-4.78871	-2.91350	-0.04792
F	-5.52002	-2.87842	-1.17862
F	-4.26927	-4.14646	0.05026
F	-5.65848	-2.76539	0.97003

p-CF3--oximyl-radical

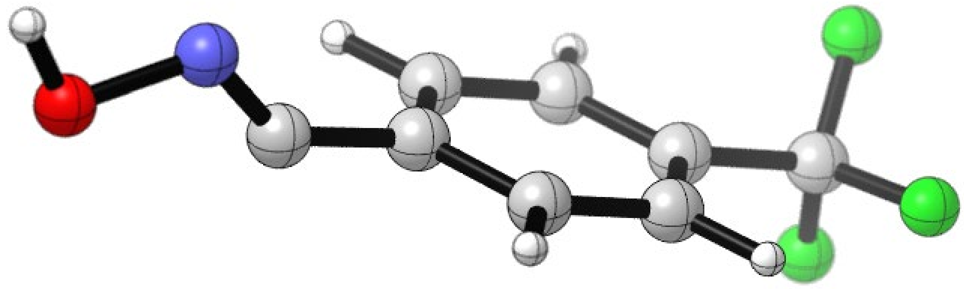

Sum of Electronic and Zero-point Energies = -736.885135 Hartree
Sum of Electronic and Thermal Energies = -736.873594 Hartree
Sum of Electronic and Thermal Enthalpies = -736.872650 Hartree
Sum of Electronic and Thermal Free Energies = -736.926020 Hartree

Dipole Moment = 2.2718 Debye

0 2

C	-2.38837	-2.07327	0.05220
C	-1.50704	-1.00039	0.05132
C	-1.99622	0.31410	-0.06419
C	-3.38650	0.53517	-0.14673
C	-4.25847	-0.54050	-0.14641
C	-3.75905	-1.84201	-0.04680
H	-2.01420	-3.08820	0.12672
H	-0.43828	-1.16672	0.13018
H	-3.76176	1.55005	-0.21971
H	-5.32877	-0.37676	-0.22484
C	-1.09639	1.42037	-0.02116
N	-0.42915	2.11776	-0.78760
O	0.33941	3.13480	-0.21592
H	0.76550	3.54139	-0.98161
C	-4.73621	-2.98264	-0.01986
F	-5.61490	-2.90783	-1.03670
F	-4.13027	-4.17724	-0.10167
F	-5.46532	-2.98528	1.11290

p-CF3-oximyl-radical-Nu-attack-tBu-P-N-oxide

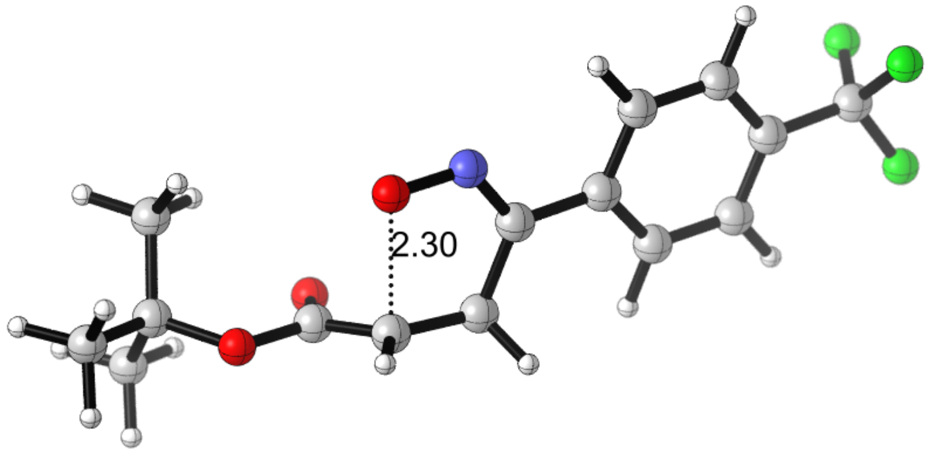


```
Sum of Electronic and Zero-point Energies = -1160.483652 Hartree
Sum of Electronic and Thermal Energies = -1160.462261 Hartree
Sum of Electronic and Thermal Enthalpies = -1160.461317 Hartree
Sum of Electronic and Thermal Free Energies = -1160.537478 Hartree

Dipole Moment = 2.7117 Debye

-1 2
C      1.84013   -0.40390   -1.10853
C      0.43790   -0.84293   -0.88953
C     -0.44110    0.26338   -0.30875
H      2.13284    0.09808   -2.02102
H      0.43663   -1.69493   -0.19516
H     -0.01547   -1.18183   -1.83326
C      2.85076   -0.76508   -0.18093
O      2.69785   -1.40663    0.86201
O      4.09132   -0.28961   -0.56585
C      5.15498   -0.21222    0.39352
N      0.06604    1.42388   -0.00009
O      1.30607    1.64230   -0.20374
C     -1.86335    0.05160   -0.06824
C     -2.46819   -1.19321   -0.32989
C     -2.69386    1.08503    0.42767
C     -3.82754   -1.40062   -0.11284
H     -1.86586   -2.01448   -0.70301
C     -4.04274    0.88006    0.64434
H     -2.24043    2.04858    0.63132
C     -4.62315   -0.36702    0.37104
H     -4.26868   -2.37020   -0.32247
H     -4.66174    1.68965    1.02175
C      5.66057   -1.60770    0.76788
H      6.54646   -1.52165    1.40851
H      4.88306   -2.16367    1.29247
H      5.93991   -2.15490   -0.13901
C      6.24560    0.55344   -0.35491
H      5.88079    1.54652   -0.63302
H      7.13557    0.66240    0.27425
```

H	6.52079	0.01723	-1.26862
C	4.71638	0.58140	1.62700
H	5.59168	0.81661	2.24389
H	4.24209	1.51539	1.30953
H	3.99607	0.01455	2.21802
C	-6.07094	-0.57631	0.65355
F	-6.83847	0.45536	0.23153
F	-6.33851	-0.70172	1.97765
F	-6.56703	-1.68784	0.07008

p-Cl--oximyl-radical-cyclisation-tBu-P

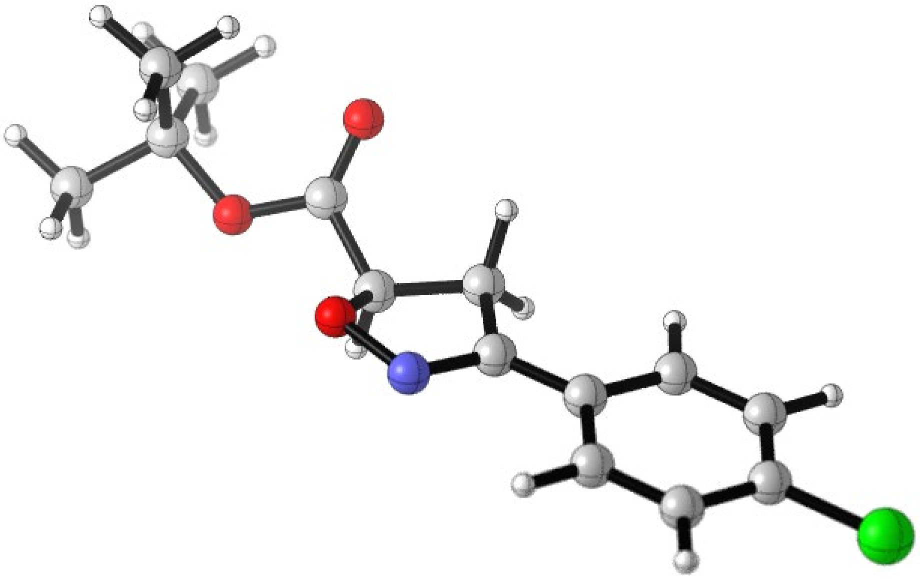

Sum of Electronic and Zero-point Energies = -1283.112711 Hartree
Sum of Electronic and Thermal Energies = -1283.093948 Hartree
Sum of Electronic and Thermal Enthalpies = -1283.093003 Hartree
Sum of Electronic and Thermal Free Energies = -1283.162287 Hartree

Dipole Moment = 10.3440 Debye

-1 2

C	1.59073	-0.42452	-0.67052
C	0.35383	-1.01522	-0.01913
C	-0.59750	0.14684	-0.20732
H	1.64213	-0.68313	-1.74102
H	0.53354	-1.24515	1.03922
H	0.02177	-1.93090	-0.51905
N	0.04091	1.28323	-0.50582
O	1.44725	0.98372	-0.53023
C	2.91119	-0.78251	-0.01247
O	3.04236	-1.37613	1.03377

O	3.93424	-0.32224	-0.75382
C	5.29604	-0.36897	-0.25839
C	-1.99994	0.05241	-0.07429
C	-2.65599	-1.17305	0.27621
C	-2.85053	1.19467	-0.28035
C	-4.03045	-1.25568	0.39864
H	-2.06315	-2.06512	0.46211
C	-4.22133	1.10420	-0.15695
H	-2.38785	2.14117	-0.54084
C	-4.82261	-0.11918	0.17963
H	-4.50141	-2.19724	0.66762
H	-4.84462	1.97970	-0.31966
C	5.40785	0.41344	1.04924
H	4.88863	-0.09621	1.86153
H	6.46467	0.52379	1.31415
H	4.97276	1.40900	0.92124
C	5.76025	-1.81683	-0.10566
H	5.60549	-2.35870	-1.04411
H	6.83029	-1.83047	0.12760
H	5.21472	-2.32209	0.69188
C	6.08397	0.32927	-1.36277
H	7.14691	0.36022	-1.10437
H	5.96487	-0.20651	-2.30893
H	5.71851	1.35098	-1.49587
Cl	-6.57729	-0.22437	0.34496

p-Cl--oximyl-radical-cyclisation-tBu-TS

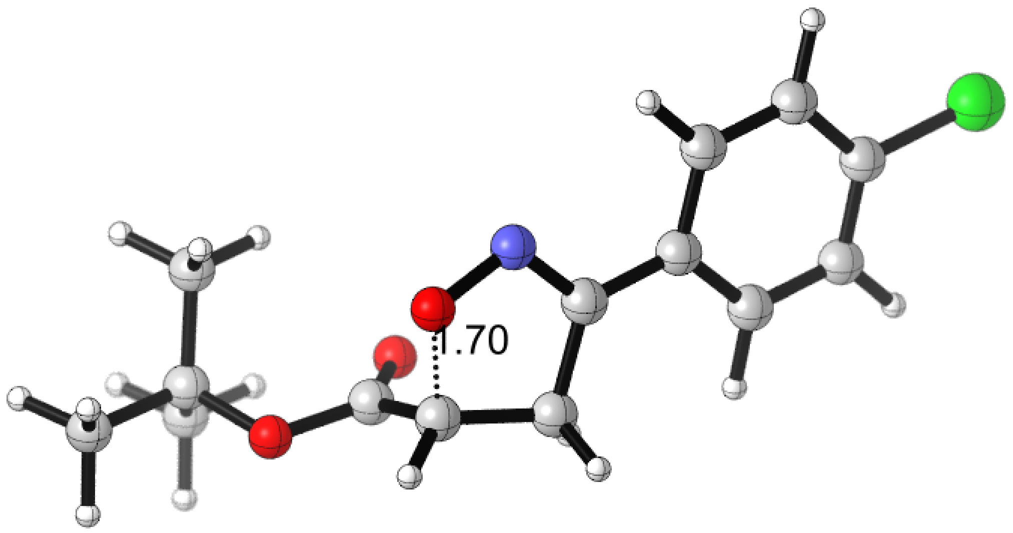

Sum of Electronic and Zero-point Energies = -1283.093409 Hartree
Sum of Electronic and Thermal Energies = -1283.074971 Hartree

Sum of Electronic and Thermal Enthalpies = -1283.074027 Hartree
Sum of Electronic and Thermal Free Energies = -1283.142833 Hartree

Dipole Moment = 2.2606 Debye

-1 2

C	-1.85739	0.13480	1.11567
C	-0.44549	-0.32310	1.40942
C	0.40790	0.44936	0.42913
H	-2.44922	0.52766	1.93862
H	-0.35531	-1.39942	1.23130
H	-0.16540	-0.10729	2.44983
N	-0.21693	1.44257	-0.14591
O	-1.44030	1.60552	0.36593
C	-2.60744	-0.66804	0.17681
O	-2.12702	-1.42163	-0.68318
O	-3.97596	-0.42534	0.26999
C	-4.79139	-0.57789	-0.89903
C	1.76611	0.10655	0.06718
C	2.41311	-1.02334	0.60930
C	2.51436	0.92110	-0.81721
C	3.73075	-1.33322	0.28881
H	1.87450	-1.67321	1.29189
C	3.82574	0.61555	-1.14511
H	2.03338	1.79671	-1.24026
C	4.43182	-0.51187	-0.58860
H	4.21196	-2.20816	0.71445
H	4.38579	1.24616	-1.82878
C	-4.98234	-2.05739	-1.24577
H	-5.70061	-2.15825	-2.06852
H	-4.02879	-2.50212	-1.53206
H	-5.37499	-2.59163	-0.37369
C	-4.19707	0.20324	-2.07364
H	-4.91246	0.22549	-2.90395
H	-3.98211	1.22958	-1.75951
H	-3.26551	-0.25480	-2.41022
C	-6.12822	0.03620	-0.48484
H	-6.85575	-0.04456	-1.29971
H	-6.52260	-0.48369	0.39406
H	-5.99303	1.09173	-0.23083
Cl	6.10095	-0.89246	-0.99410

p-Cl--oximyl-radical-Nu-attack-tBu-P

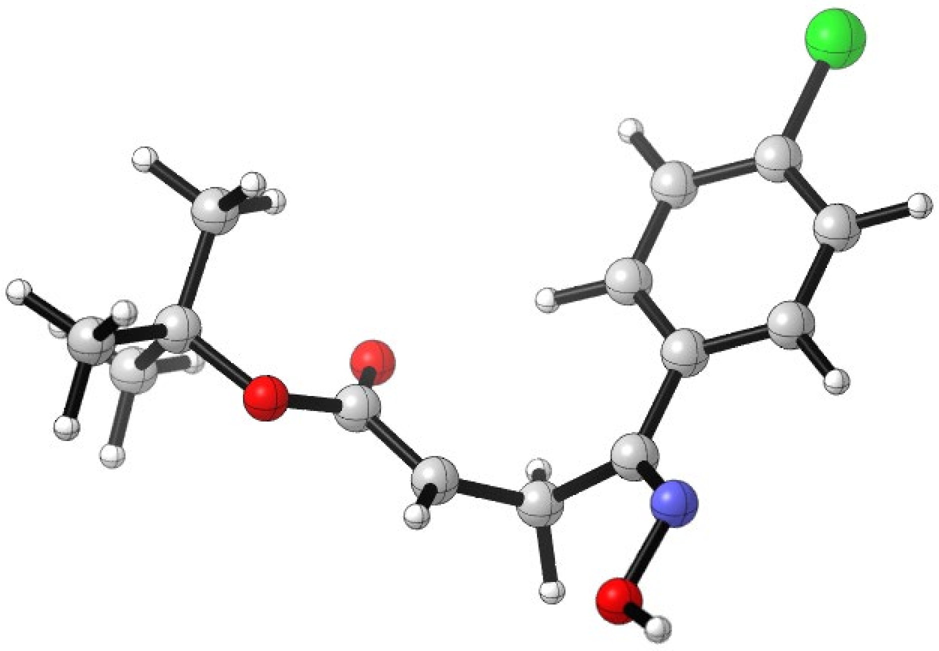

Sum of Electronic and Zero-point Energies = -1283.643694 Hartree
Sum of Electronic and Thermal Energies = -1283.624315 Hartree
Sum of Electronic and Thermal Enthalpies = -1283.623370 Hartree
Sum of Electronic and Thermal Free Energies = -1283.693851 Hartree

Dipole Moment = 3.1696 Debye

0 2

C	3.82401	-0.77953	-0.50705
C	2.96201	0.29598	-0.34247
C	1.64228	0.09838	0.08726
C	1.20232	-1.20285	0.35361
C	2.05997	-2.28866	0.18718
C	3.36279	-2.06757	-0.24093
H	4.84697	-0.62776	-0.83340
H	3.30741	1.30423	-0.54299
H	0.18311	-1.38891	0.68157
H	1.71741	-3.29770	0.38784
C	0.72220	1.25254	0.23989
N	1.02208	2.32296	-0.39427
O	0.08295	3.32648	-0.19274
H	0.43527	4.06695	-0.69970
C	-0.53190	1.13780	1.09139
C	-1.72943	0.91661	0.23409
H	-2.05402	1.68706	-0.45471
H	-0.43141	0.32320	1.80970
H	-0.64159	2.08305	1.63510
C	-2.45175	-0.34490	0.27892
O	-2.12666	-1.28665	0.98842
O	-3.50782	-0.33755	-0.55170
C	-4.37336	-1.50495	-0.67910
C	-5.39986	-1.05277	-1.71169

H	-6.12093	-1.85399	-1.89521
H	-5.93789	-0.17105	-1.35303
H	-4.90649	-0.80103	-2.65443
C	-5.05104	-1.80410	0.65602
H	-4.33131	-2.14696	1.39991
H	-5.55593	-0.90789	1.02921
H	-5.80404	-2.58409	0.50761
C	-3.57550	-2.69689	-1.20307
H	-3.04013	-2.41790	-2.11572
H	-2.85993	-3.05440	-0.46211
H	-4.26657	-3.50958	-1.44662
Cl	4.44260	-3.42094	-0.44630

p-Cl--oximyl-radical-Nu-attack-tBu-TS

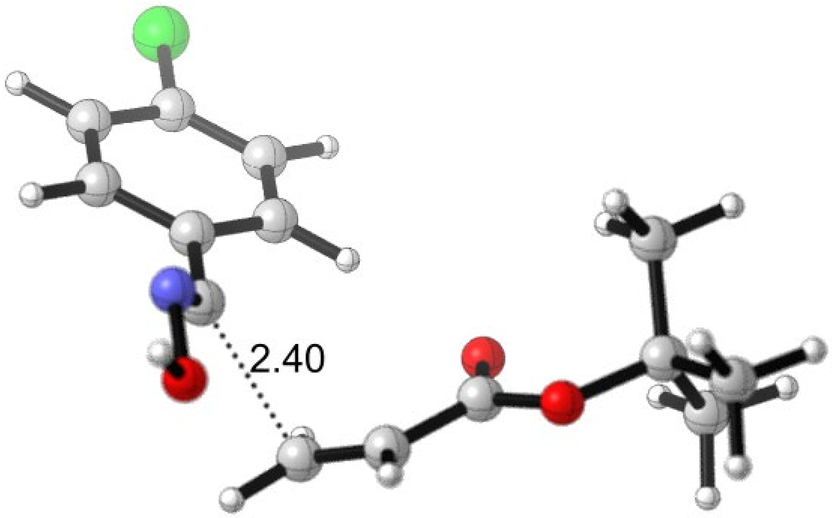

Sum of Electronic and Zero-point Energies = -1283.585951 Hartree
Sum of Electronic and Thermal Energies = -1283.565708 Hartree
Sum of Electronic and Thermal Enthalpies = -1283.564764 Hartree
Sum of Electronic and Thermal Free Energies = -1283.638089 Hartree

Dipole Moment = 2.5627 Debye

0 2

C	-2.36587	-2.15271	0.06547
C	-1.42274	-1.13151	0.08896
C	-1.83220	0.20413	-0.03948
C	-3.19871	0.50560	-0.15981
C	-4.14226	-0.51339	-0.17773
C	-3.71531	-1.83523	-0.06709
H	-2.05967	-3.18903	0.15493

H	-0.36755	-1.36721	0.20458
H	-3.50980	1.54145	-0.24638
H	-5.19915	-0.29181	-0.27676
C	-0.85818	1.27562	-0.00207
N	-0.74013	2.28738	-0.71536
O	0.24254	3.20033	-0.32116
H	0.20049	3.87614	-1.00924
C	0.95176	0.99419	1.54590
C	2.04979	0.85305	0.77499
H	2.62190	1.70513	0.42663
H	0.66945	1.96775	1.93089
H	0.44861	0.11640	1.94214
C	2.47471	-0.48931	0.32587
O	1.84789	-1.51289	0.53159
O	3.63474	-0.42718	-0.34146
C	4.25946	-1.62699	-0.88871
C	3.34630	-2.26551	-1.93277
H	3.89358	-3.06192	-2.44629
H	2.45331	-2.69101	-1.47420
H	3.04843	-1.52038	-2.67670
C	5.52251	-1.08061	-1.54507
H	6.15274	-0.58352	-0.80260
H	6.09046	-1.89799	-1.99778
H	5.26482	-0.35776	-2.32399
C	4.61560	-2.59397	0.23783
H	5.21525	-3.41296	-0.17099
H	5.21121	-2.08024	0.99850
H	3.72147	-3.00978	0.70308
Cl	-4.89591	-3.11587	-0.08693

p-Cl--oximyl-radical

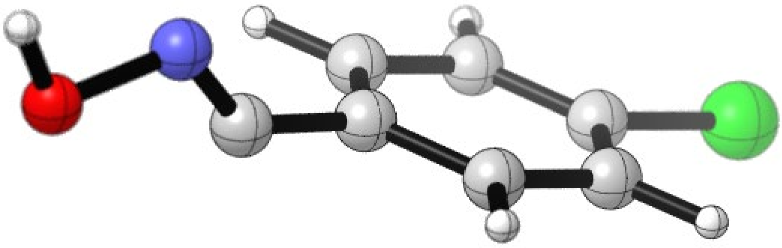

Sum of Electronic and Zero-point Energies = -859.513653 Hartree
Sum of Electronic and Thermal Energies = -859.504512 Hartree
Sum of Electronic and Thermal Enthalpies = -859.503567 Hartree
Sum of Electronic and Thermal Free Energies = -859.549994 Hartree

Dipole Moment = 0.9395 Debye

0 2

C	-2.31883	-2.07828	0.05989
C	-1.46809	-0.98235	0.06516
C	-1.98521	0.32099	-0.06096
C	-3.37797	0.50182	-0.15837
C	-4.22910	-0.59385	-0.16376
C	-3.69363	-1.87642	-0.05403
H	-1.92600	-3.08540	0.14422
H	-0.39675	-1.12491	0.15824
H	-3.78168	1.50554	-0.23776
H	-5.30183	-0.46198	-0.25110
C	-1.11288	1.45116	-0.00975
N	-0.47057	2.15718	-0.79269
O	0.27375	3.19996	-0.23060
H	0.68537	3.61097	-1.00154
Cl	-4.76104	-3.25053	-0.05502

p-Cl-oximyl-radical-Nu-attack-tBu-P-N-oxide

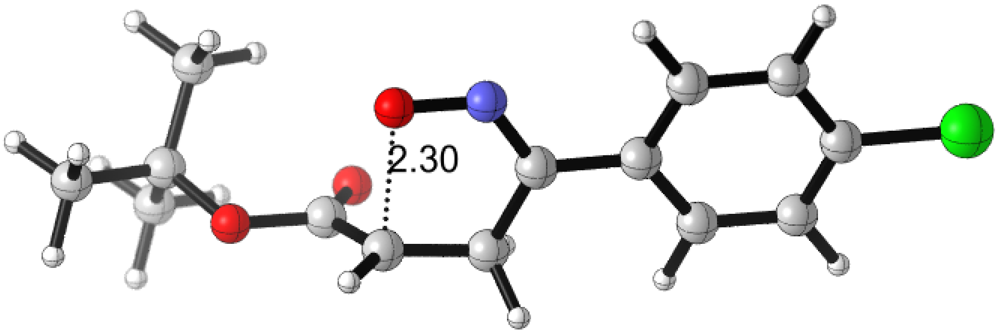

Sum of Electronic and Zero-point Energies = -1283.106499 Hartree
Sum of Electronic and Thermal Energies = -1283.087509 Hartree
Sum of Electronic and Thermal Enthalpies = -1283.086565 Hartree
Sum of Electronic and Thermal Free Energies = -1283.157049 Hartree

Dipole Moment = 1.8673 Debye

-1 2

C	1.83919	-0.39902	-1.08897
C	0.43919	-0.83994	-0.86071
C	-0.43066	0.26306	-0.25982
H	2.11761	0.13144	-1.98978
H	0.44473	-1.69969	-0.17571
H	-0.02736	-1.16793	-1.80221
C	2.86335	-0.79348	-0.19362
O	2.73108	-1.46902	0.83182
O	4.10079	-0.30775	-0.58450
C	5.16138	-0.21330	0.37577
N	0.08613	1.40667	0.07544
O	1.33308	1.62050	-0.11877
C	-1.86138	0.05506	-0.02854
C	-2.46850	-1.18644	-0.28738
C	-2.68994	1.08926	0.45703
C	-3.83178	-1.39373	-0.07536
H	-1.86856	-2.01326	-0.65301
C	-4.04533	0.89417	0.67310
H	-2.23626	2.05298	0.66172
C	-4.61144	-0.35204	0.40311
H	-4.28154	-2.36064	-0.27795
H	-4.66792	1.70088	1.04737
C	5.69874	-1.59985	0.74004
H	6.58179	-1.49898	1.38287

H	4.93167	-2.17643	1.25799
H	5.99134	-2.13343	-0.17089
C	6.23456	0.58367	-0.36519
H	5.84599	1.56993	-0.63533
H	7.12190	0.70895	0.26485
H	6.52243	0.06189	-1.28344
C	4.70406	0.55912	1.61600
H	5.57369	0.80663	2.23626
H	4.20796	1.48483	1.30741
H	3.99591	-0.02987	2.20044
Cl	-6.33102	-0.60234	0.67532

p-CN--oximyl-radical-cyclisation-tBu-P

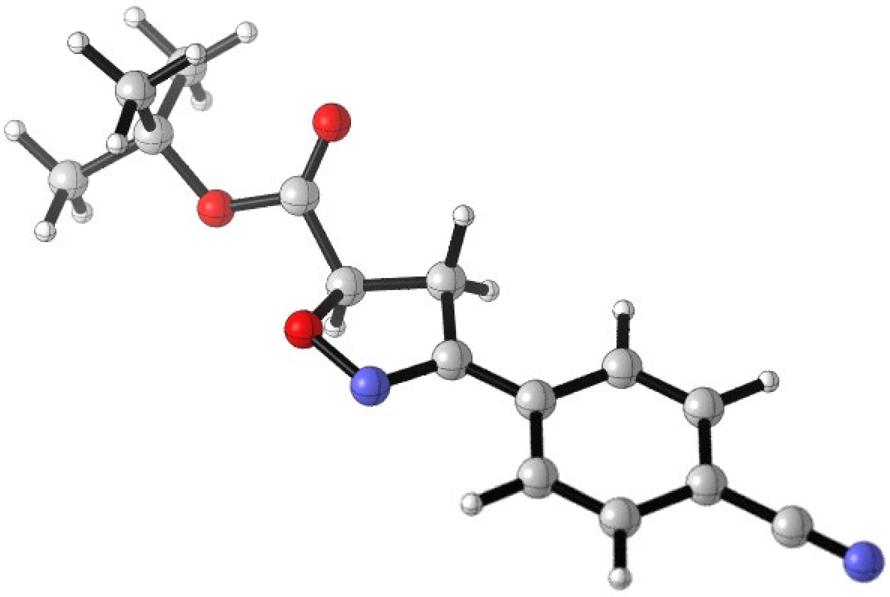

Sum of Electronic and Zero-point Energies = -915.774089 Hartree
Sum of Electronic and Thermal Energies = -915.754940 Hartree
Sum of Electronic and Thermal Enthalpies = -915.753996 Hartree
Sum of Electronic and Thermal Free Energies = -915.824024 Hartree

Dipole Moment = 16.3492 Debye

-1 2

C	1.58920	-0.43012	-0.72288
C	0.34664	-1.02858	-0.09147
C	-0.60169	0.14422	-0.24509
H	1.66595	-0.68504	-1.79124
H	0.51617	-1.28062	0.96229
H	0.00572	-1.92626	-0.61388
N	0.03447	1.27046	-0.52186
O	1.43226	0.98134	-0.58876

C	2.89856	-0.77539	-0.03400
O	3.00463	-1.34798	1.02615
O	3.93219	-0.32831	-0.76447
C	5.28770	-0.36778	-0.24550
C	-2.00673	0.05284	-0.08867
C	-2.65374	-1.18052	0.24697
C	-2.85503	1.20141	-0.25584
C	-4.01655	-1.26605	0.39504
H	-2.05405	-2.07464	0.39886
C	-4.21446	1.11389	-0.10933
H	-2.39105	2.15035	-0.50597
C	-4.84758	-0.12207	0.21970
H	-4.47661	-2.21634	0.65236
H	-4.83258	1.99781	-0.24415
C	5.37765	0.43870	1.04877
H	4.84264	-0.05388	1.86136
H	6.42976	0.55022	1.33069
H	4.94955	1.43356	0.89464
C	5.74431	-1.81364	-0.05822
H	5.60384	-2.37293	-0.98860
H	6.81020	-1.82531	0.19261
H	5.18540	-2.30268	0.74017
C	6.09401	0.30814	-1.35009
H	7.15250	0.34180	-1.07502
H	5.98955	-0.24504	-2.28787
H	5.73379	1.32817	-1.50797
C	-6.25146	-0.20858	0.37076
N	-7.41175	-0.28206	0.49461

p-CN--oximyl-radical-cyclisation-tBu-TS

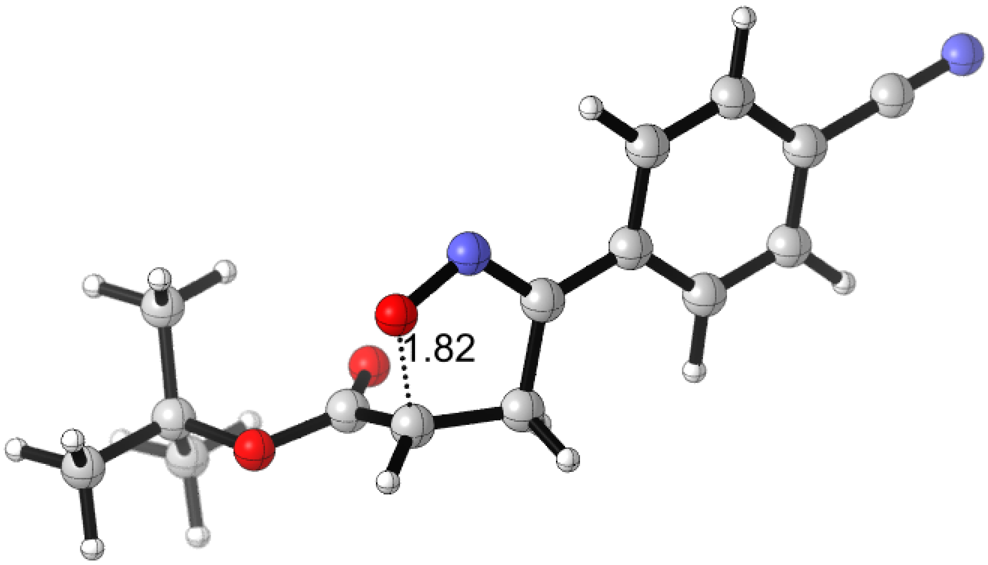

Sum of Electronic and Zero-point Energies = -915.744560 Hartree
Sum of Electronic and Thermal Energies = -915.725625 Hartree
Sum of Electronic and Thermal Enthalpies = -915.724681 Hartree
Sum of Electronic and Thermal Free Energies = -915.794018 Hartree

Dipole Moment = 6.2130 Debye

-1 2

C	-1.83873	0.08977	1.11284
C	-0.42965	-0.37797	1.34484
C	0.44851	0.44516	0.41656
H	-2.40839	0.49342	1.94257
H	-0.34067	-1.43817	1.08443
H	-0.13471	-0.24497	2.39410
N	-0.15738	1.47178	-0.12902
O	-1.35693	1.67525	0.36588
C	-2.60621	-0.59476	0.10383
O	-2.14559	-1.31426	-0.78573
O	-3.95202	-0.29488	0.19834
C	-4.82267	-0.55739	-0.91276
C	1.80241	0.10953	0.06878
C	2.42389	-1.06667	0.55522
C	2.58837	0.97426	-0.74106
C	3.74031	-1.36601	0.25431
H	1.85805	-1.75110	1.17888
C	3.89699	0.67287	-1.05025
H	2.12792	1.88207	-1.11555
C	4.50170	-0.50292	-0.55493
H	4.19590	-2.27317	0.64017
H	4.47835	1.34267	-1.67731
C	-4.99598	-2.06266	-1.13129
H	-5.76576	-2.23920	-1.89167
H	-4.05621	-2.51251	-1.45240
H	-5.31643	-2.53634	-0.19717
C	-4.31204	0.14287	-2.17405
H	-5.07243	0.08361	-2.96117
H	-4.11285	1.19613	-1.95285
H	-3.38864	-0.31755	-2.52736
C	-6.14890	0.06086	-0.47253
H	-6.91262	-0.08686	-1.24320
H	-6.49000	-0.40439	0.45752
H	-6.02271	1.13318	-0.29712
C	5.86322	-0.80743	-0.86360
N	6.97161	-1.05487	-1.11407

p-CN--oximyl-radical-Nu-attack-tBu-P

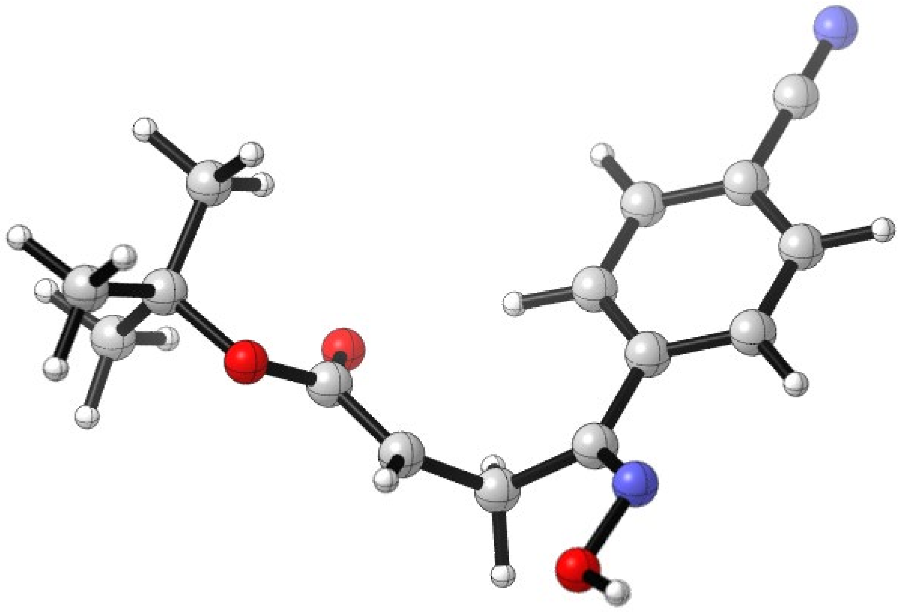

Sum of Electronic and Zero-point Energies = -916.282906 Hartree
Sum of Electronic and Thermal Energies = -916.262932 Hartree
Sum of Electronic and Thermal Enthalpies = -916.261988 Hartree
Sum of Electronic and Thermal Free Energies = -916.333563 Hartree

Dipole Moment = 6.2591 Debye

0 2

C	3.82471	-0.78473	-0.50303
C	2.96530	0.29080	-0.34451
C	1.64465	0.09078	0.08482
C	1.19947	-1.20856	0.35638
C	2.05713	-2.29215	0.19545
C	3.37022	-2.08290	-0.23313
H	4.84766	-0.63097	-0.82926
H	3.30770	1.29927	-0.54710
H	0.17912	-1.38901	0.68309
H	1.71093	-3.29936	0.40039
C	0.72477	1.24663	0.23636
N	1.03035	2.31206	-0.40344
O	0.09733	3.31827	-0.21183
H	0.45146	4.05505	-0.72346
C	-0.52885	1.13914	1.08855
C	-1.72976	0.92053	0.23437
H	-2.06160	1.69534	-0.44597
H	-0.43070	0.32597	1.80896
H	-0.63329	2.08598	1.63037
C	-2.44602	-0.34541	0.27286
O	-2.10765	-1.29176	0.97013
O	-3.50870	-0.33509	-0.54696
C	-4.37016	-1.50630	-0.67962
C	-5.40829	-1.04737	-1.69729

H	-6.12656	-1.85037	-1.88364
H	-5.94795	-0.17310	-1.32324
H	-4.92514	-0.78193	-2.64156
C	-5.03313	-1.82360	0.65850
H	-4.30502	-2.17224	1.39146
H	-5.53851	-0.93426	1.04704
H	-5.78401	-2.60518	0.50818
C	-3.57030	-2.68738	-1.22425
H	-3.04594	-2.39576	-2.13934
H	-2.84554	-3.04888	-0.49416
H	-4.25883	-3.50160	-1.46958
C	4.26151	-3.20257	-0.39504
N	4.97929	-4.10136	-0.52692

p-CN--oximyl-radical-Nu-attack-tBu-TS

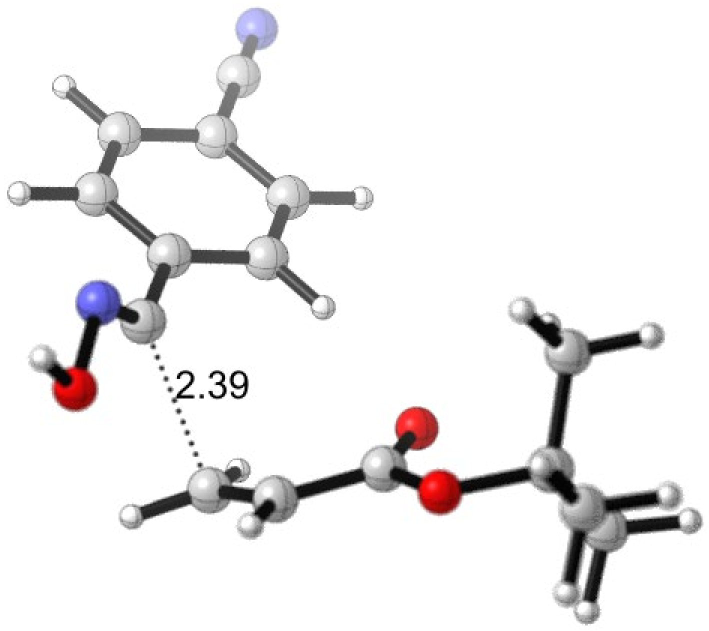

Sum of Electronic and Zero-point Energies = -916.224807 Hartree
Sum of Electronic and Thermal Energies = -916.203985 Hartree
Sum of Electronic and Thermal Enthalpies = -916.203041 Hartree
Sum of Electronic and Thermal Free Energies = -916.277250 Hartree

Dipole Moment = 5.8287 Debye

0 2

C	-2.39664	-2.16334	0.06317
C	-1.44124	-1.15659	0.05793
C	-1.84381	0.18431	-0.05185
C	-3.21038	0.50704	-0.12249
C	-4.16310	-0.50014	-0.11191
C	-3.75646	-1.83802	-0.02107

H	-2.09683	-3.20296	0.13785
H	-0.38473	-1.39994	0.13822
H	-3.50833	1.54755	-0.19414
H	-5.21947	-0.26227	-0.17487
C	-0.85999	1.24505	-0.03750
N	-0.70596	2.22552	-0.78494
O	0.27729	3.13855	-0.40397
H	0.26361	3.78732	-1.11910
C	0.92070	0.96618	1.53886
C	2.02746	0.84061	0.77813
H	2.60446	1.69913	0.45465
H	0.63204	1.93223	1.93821
H	0.41568	0.08051	1.91477
C	2.45867	-0.49367	0.30726
O	1.82011	-1.51732	0.47221
O	3.63477	-0.42074	-0.32691
C	4.27196	-1.61046	-0.88483
C	3.38604	-2.22073	-1.96826
H	3.94531	-3.00623	-2.48548
H	2.48006	-2.65497	-1.54478
H	3.11054	-1.45735	-2.70223
C	5.55410	-1.05270	-1.49244
H	6.16439	-0.57478	-0.72134
H	6.13255	-1.86125	-1.94756
H	5.32036	-0.31161	-2.26170
C	4.59300	-2.60378	0.22903
H	5.19986	-3.41633	-0.18184
H	5.17025	-2.11032	1.01675
H	3.68501	-3.02599	0.66051
C	-4.74548	-2.88425	-0.00718
N	-5.54202	-3.72447	0.00302

p-CN--oximyl-radical

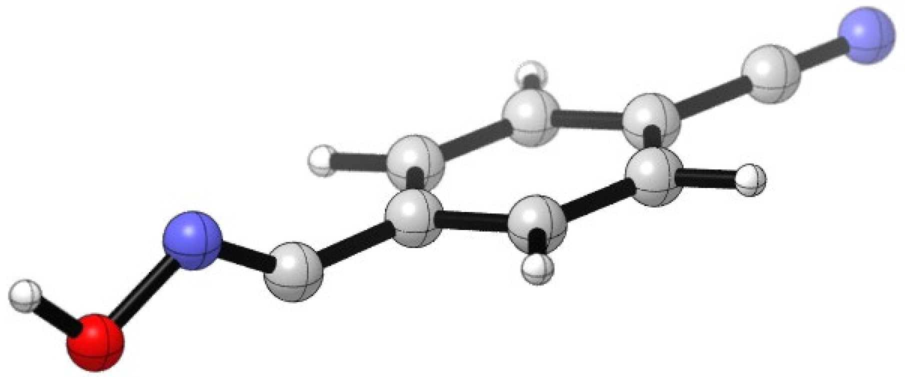

Sum of Electronic and Zero-point Energies = -492.152819 Hartree
Sum of Electronic and Thermal Energies = -492.143059 Hartree
Sum of Electronic and Thermal Enthalpies = -492.142115 Hartree
Sum of Electronic and Thermal Free Energies = -492.189407 Hartree

Dipole Moment = 4.0038 Debye

0 2

C	-2.37948	-2.08928	0.03227
C	-1.49948	-1.02221	-0.01052
C	-1.99084	0.29746	-0.10793
C	-3.38428	0.52170	-0.12706
C	-4.26024	-0.54870	-0.08408
C	-3.76275	-1.85806	-0.00396
H	-2.00787	-3.10630	0.09381
H	-0.42878	-1.19097	0.02167
H	-3.75978	1.53742	-0.18392
H	-5.33180	-0.38367	-0.11153
C	-1.09100	1.39495	-0.10408
N	-0.44155	2.11768	-0.85965
O	0.34057	3.11648	-0.27485
H	0.74982	3.54654	-1.03713
C	-4.67535	-2.96795	0.04477
N	-5.41075	-3.86174	0.08338

p-CN-oximyl-radical-Nu-attack-tBu-P-N-oxide

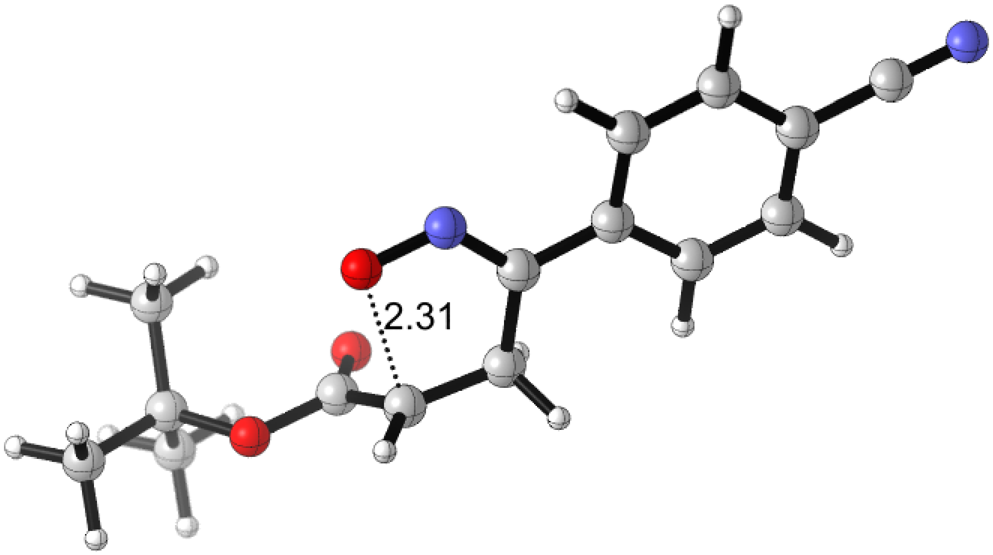

Sum of Electronic and Zero-point Energies = -915.754839 Hartree
Sum of Electronic and Thermal Energies = -915.735309 Hartree
Sum of Electronic and Thermal Enthalpies = -915.734365 Hartree
Sum of Electronic and Thermal Free Energies = -915.805220 Hartree

Dipole Moment = 4.7530 Debye

-1 2

C	1.82886	-0.40096	-1.08976
C	0.43047	-0.84519	-0.85852
C	-0.44950	0.25888	-0.27469
H	2.10982	0.11306	-1.99931
H	0.43962	-1.69605	-0.16283
H	-0.02763	-1.18780	-1.79845
C	2.85148	-0.76214	-0.17268
O	2.71033	-1.41581	0.86344
O	4.08198	-0.27051	-0.56151
C	5.15850	-0.20592	0.38595
N	0.05785	1.42037	0.04426
O	1.29580	1.63755	-0.15406
C	-1.86821	0.05059	-0.03900
C	-2.47472	-1.19746	-0.30033
C	-2.70083	1.08570	0.45355
C	-3.83068	-1.40350	-0.08840
H	-1.87105	-2.02028	-0.66764
C	-4.04922	0.88491	0.66669
H	-2.24706	2.04838	0.65997
C	-4.63651	-0.36634	0.39563
H	-4.27464	-2.37286	-0.29484
H	-4.66965	1.69263	1.04354

C	5.67370	-1.60597	0.72784
H	6.56802	-1.52745	1.35753
H	4.90586	-2.17443	1.25346
H	5.94289	-2.13576	-0.19228
C	6.23513	0.57823	-0.36303
H	5.86043	1.57293	-0.62132
H	7.13168	0.68392	0.25718
H	6.50374	0.05862	-1.28817
C	4.73213	0.56276	1.63918
H	5.61402	0.78564	2.25102
H	4.25484	1.50304	1.34572
H	4.01866	-0.01624	2.22694
C	-6.03846	-0.57493	0.61069
N	-7.17440	-0.74237	0.78293

p-F--oximyl-radical-cyclisation-tBu-P

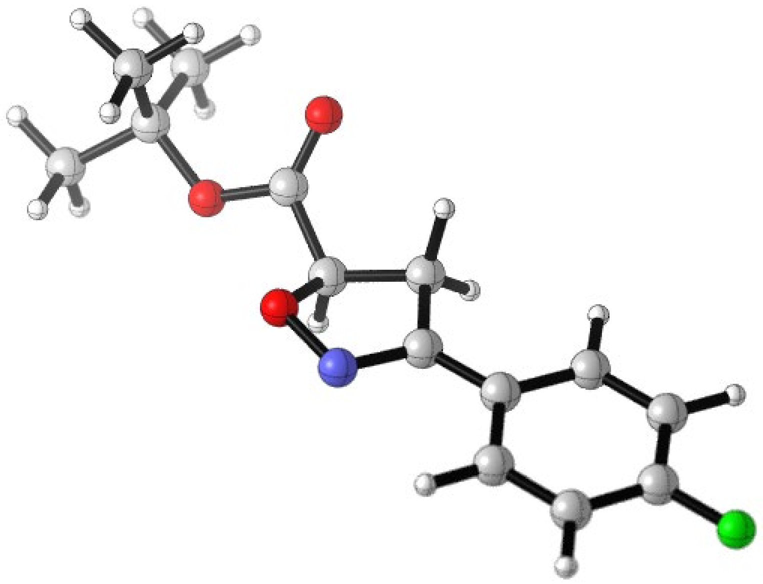

Sum of Electronic and Zero-point Energies = -922.746842 Hartree
Sum of Electronic and Thermal Energies = -922.728462 Hartree
Sum of Electronic and Thermal Enthalpies = -922.727518 Hartree
Sum of Electronic and Thermal Free Energies = -922.795412 Hartree

Dipole Moment = 8.6653 Debye

-1 2

C	1.59014	-0.42041	-0.65427
C	0.35448	-1.01063	0.00087
C	-0.59761	0.14771	-0.19624
H	1.63493	-0.67845	-1.72575
H	0.53692	-1.23752	1.06002

H	0.02648	-1.93091	-0.49505
N	0.04063	1.28818	-0.50163
O	1.45010	0.98638	-0.50992
C	2.91345	-0.78281	-0.00527
O	3.05196	-1.38232	1.03686
O	3.93386	-0.31952	-0.74996
C	5.29730	-0.36939	-0.26160
C	-2.00082	0.05155	-0.07119
C	-2.65744	-1.17259	0.28341
C	-2.85002	1.19312	-0.28999
C	-4.03630	-1.25430	0.39771
H	-2.06371	-2.06164	0.47952
C	-4.22512	1.10092	-0.17396
H	-2.38421	2.13714	-0.55294
C	-4.81126	-0.11898	0.16510
H	-4.52264	-2.18724	0.66939
H	-4.86089	1.96629	-0.34286
C	5.41648	0.40608	1.04964
H	4.90156	-0.10801	1.86187
H	6.47476	0.51536	1.30936
H	4.98016	1.40204	0.92920
C	5.76250	-1.81807	-0.11880
H	5.60290	-2.35485	-1.05939
H	6.83373	-1.83312	0.10915
H	5.22050	-2.32744	0.67854
C	6.08042	0.33449	-1.36597
H	7.14468	0.36383	-1.11259
H	5.95641	-0.19622	-2.31437
H	5.71435	1.35689	-1.49193
F	-6.18084	-0.20161	0.28349

p-F--oximyl-radical-cyclisation-tBu-TS

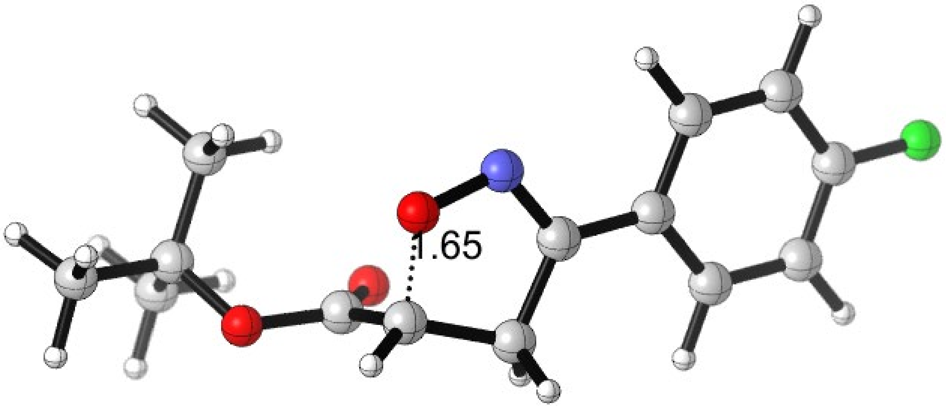

Sum of Electronic and Zero-point Energies = -922.731535 Hartree
Sum of Electronic and Thermal Energies = -922.713521 Hartree
Sum of Electronic and Thermal Enthalpies = -922.712577 Hartree
Sum of Electronic and Thermal Free Energies = -922.779718 Hartree

Dipole Moment = 3.4499 Debye

-1 2

C	-1.87839	0.13230	1.12091
C	-0.45775	-0.29537	1.44371
C	0.36595	0.43155	0.40997
H	-2.47604	0.53805	1.93557
H	-0.35823	-1.37964	1.33364
H	-0.17486	-0.00829	2.46630
N	-0.28434	1.38073	-0.20367
O	-1.51777	1.52364	0.31822
C	-2.61710	-0.76396	0.25260
O	-2.12031	-1.55452	-0.57088
O	-3.99701	-0.55197	0.33254
C	-4.76054	-0.58843	-0.88109
C	1.73070	0.09671	0.04596
C	2.41070	-0.97610	0.65473
C	2.43813	0.86344	-0.90940
C	3.73100	-1.28050	0.33005
H	1.89657	-1.58529	1.39146
C	3.75264	0.56584	-1.24189
H	1.92473	1.69352	-1.38291
C	4.38051	-0.50284	-0.61449
H	4.25405	-2.10902	0.79653
H	4.29797	1.14881	-1.97753
C	-4.97143	-2.03175	-1.34819
H	-5.64115	-2.05178	-2.21696

H	-4.01261	-2.48173	-1.60818
H	-5.42813	-2.61596	-0.54171
C	-4.08546	0.25701	-1.96364
H	-4.75261	0.35565	-2.82793
H	-3.85875	1.25173	-1.56708
H	-3.14761	-0.20306	-2.28206
C	-6.09955	0.03318	-0.48626
H	-6.79104	0.02884	-1.33609
H	-6.54663	-0.53493	0.33593
H	-5.95005	1.06469	-0.15333
F	5.67515	-0.79064	-0.93508

p-F--oximyl-radical-Nu-attack-tBu-P

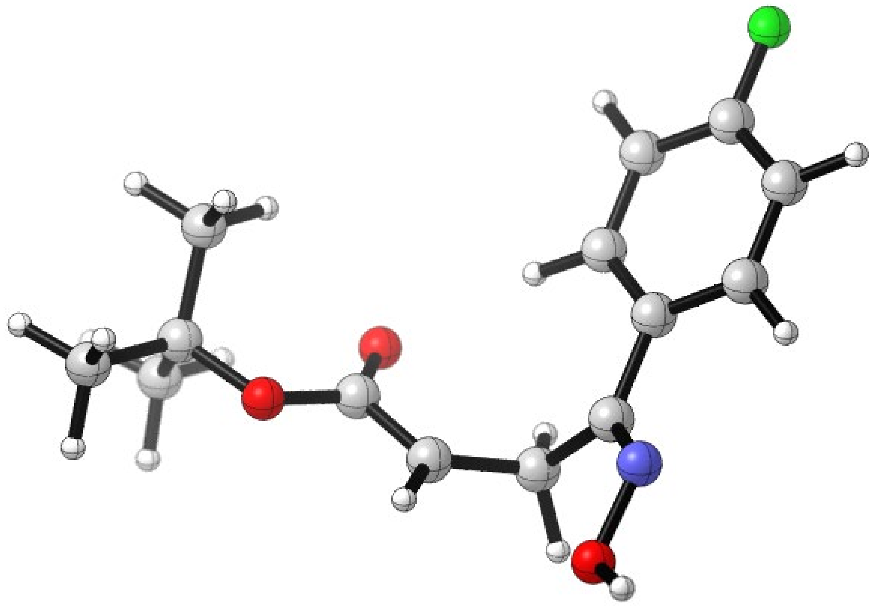

Sum of Electronic and Zero-point Energies = -923.285113 Hartree
Sum of Electronic and Thermal Energies = -923.266126 Hartree
Sum of Electronic and Thermal Enthalpies = -923.265182 Hartree
Sum of Electronic and Thermal Free Energies = -923.334349 Hartree

Dipole Moment = 2.9033 Debye

0 2

C	3.83390	-0.77544	-0.49788
C	2.96720	0.29751	-0.33778
C	1.64552	0.09578	0.08662
C	1.20555	-1.20643	0.35291
C	2.06513	-2.29191	0.19293
C	3.36159	-2.05336	-0.22912
H	4.86077	-0.63927	-0.81801
H	3.30849	1.30731	-0.53718

H	0.18479	-1.39159	0.67625
H	1.74042	-3.30727	0.38987
C	0.72210	1.24795	0.23222
N	1.01630	2.31634	-0.40798
O	0.07408	3.31866	-0.20635
H	0.42265	4.05783	-0.71758
C	-0.52992	1.13484	1.08777
C	-1.73086	0.91647	0.23472
H	-2.05809	1.68932	-0.45009
H	-0.42836	0.32000	1.80558
H	-0.63612	2.08033	1.63184
C	-2.45250	-0.34529	0.27720
O	-2.12477	-1.29030	0.98120
O	-3.51221	-0.33469	-0.54918
C	-4.37725	-1.50194	-0.67870
C	-5.40880	-1.04533	-1.70432
H	-6.12965	-1.84641	-1.88932
H	-5.94637	-0.16634	-1.33832
H	-4.91978	-0.78752	-2.64769
C	-5.04898	-1.80897	0.65767
H	-4.32596	-2.15608	1.39635
H	-5.55217	-0.91497	1.03836
H	-5.80267	-2.58808	0.50803
C	-3.58069	-2.69043	-1.21246
H	-3.05052	-2.40643	-2.12660
H	-2.86067	-3.05019	-0.47687
H	-4.27190	-3.50300	-1.45609
F	4.20033	-3.09710	-0.38012

p-F--oximyl-radical-Nu-attack-tBu-TS

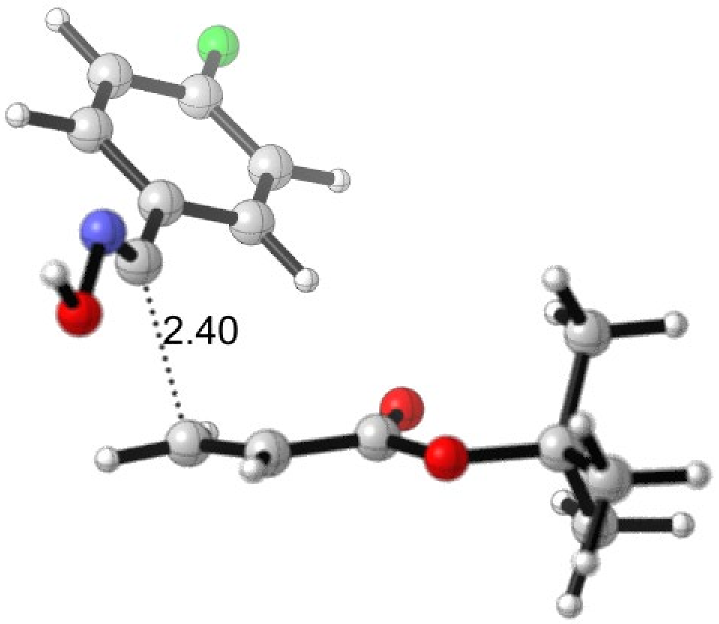

Sum of Electronic and Zero-point Energies = -923.227477 Hartree
Sum of Electronic and Thermal Energies = -923.207636 Hartree
Sum of Electronic and Thermal Enthalpies = -923.206691 Hartree
Sum of Electronic and Thermal Free Energies = -923.278621 Hartree

Dipole Moment = 2.3005 Debye

0 2

C	-2.36065	-2.15450	0.06767
C	-1.42100	-1.12987	0.09633
C	-1.83363	0.20522	-0.03693
C	-3.20004	0.50582	-0.16874
C	-4.14319	-0.51382	-0.19244
C	-3.70059	-1.82462	-0.07576
H	-2.07064	-3.19496	0.16018
H	-0.36569	-1.36097	0.21930
H	-3.50943	1.54177	-0.25893
H	-5.20318	-0.31225	-0.29807
C	-0.86054	1.27816	0.00393
N	-0.75515	2.29947	-0.69824
O	0.22907	3.21165	-0.30295
H	0.17749	3.89513	-0.98264
C	0.95853	0.99834	1.54411
C	2.05491	0.85475	0.77130
H	2.62588	1.70578	0.41853
H	0.67680	1.97317	1.92618
H	0.45630	0.12188	1.94437
C	2.47866	-0.48877	0.32577
O	1.85269	-1.51202	0.53637
O	3.63720	-0.42902	-0.34487
C	4.26020	-1.63043	-0.88988
C	3.34431	-2.27221	-1.92958
H	3.89002	-3.07055	-2.44180
H	2.45228	-2.69570	-1.46728
H	3.04483	-1.52941	-2.67521
C	5.52164	-1.08638	-1.55133
H	6.15393	-0.58716	-0.81204
H	6.08823	-1.90523	-2.00311
H	5.26198	-0.36578	-2.33167
C	4.61932	-2.59406	0.23862
H	5.21804	-3.41425	-0.16922
H	5.21676	-2.07796	0.99625
H	3.72637	-3.00842	0.70739
F	-4.61199	-2.81512	-0.09536

p-F--oximyl-radical

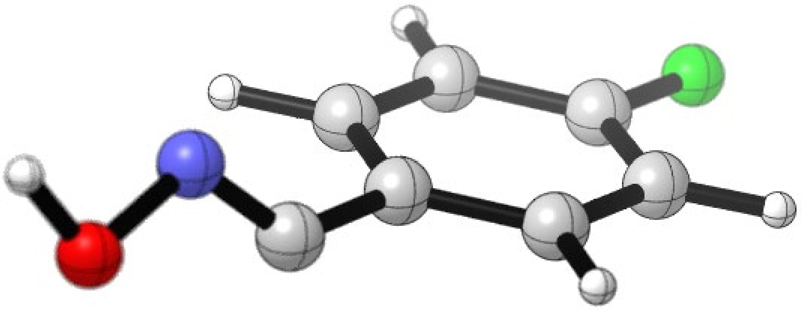

Sum of Electronic and Zero-point Energies = -499.154851 Hartree
Sum of Electronic and Thermal Energies = -499.146070 Hartree
Sum of Electronic and Thermal Enthalpies = -499.145126 Hartree
Sum of Electronic and Thermal Free Energies = -499.190715 Hartree

Dipole Moment = 0.8310 Debye

0 2

C	-2.34857	-2.14109	0.05770
C	-1.43506	-1.09547	0.08642
C	-1.87073	0.23383	-0.03867
C	-3.24527	0.50440	-0.15879
C	-4.16618	-0.53535	-0.18083
C	-3.69698	-1.83746	-0.07402
H	-2.03793	-3.17620	0.14149
H	-0.37575	-1.29971	0.20147
H	-3.57822	1.53343	-0.24216
H	-5.23067	-0.35551	-0.27983
C	-0.92143	1.31707	0.01604
N	-0.67596	2.27201	-0.73368
O	0.23080	3.22221	-0.26141
H	0.30868	3.83573	-1.00293
F	-4.58589	-2.84677	-0.09344

p-F-oximyl-radical-Nu-attack-tBu-P-N-oxide

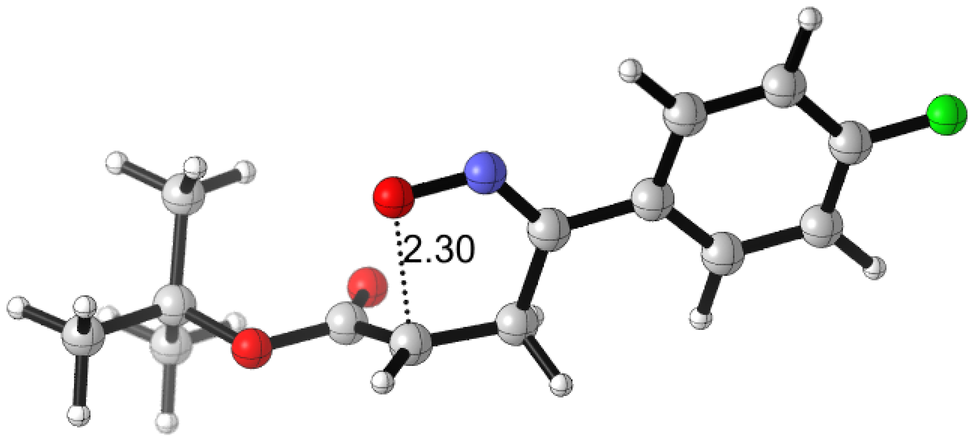


```
Sum of Electronic and Zero-point Energies = -922.745571 Hartree
Sum of Electronic and Thermal Energies = -922.727020 Hartree
Sum of Electronic and Thermal Enthalpies = -922.726076 Hartree
Sum of Electronic and Thermal Free Energies = -922.794731 Hartree

Dipole Moment = 1.4891 Debye

-1 2
C                  1.84176     -0.40232     -1.09320
C                  0.44109     -0.84154     -0.86530
C                 -0.42542      0.26162     -0.25924
H                  2.12044      0.13067     -1.99240
H                  0.44555     -1.70363     -0.18305
H                 -0.02871     -1.16562     -1.80674
C                  2.86478     -0.80247     -0.20092
O                  2.73368     -1.48264      0.82239
O                  4.10443     -0.31757     -0.59053
C                  5.15895     -0.21412      0.37465
N                  0.09342      1.40140      0.07540
O                  1.34250      1.61623     -0.12161
C                 -1.86010      0.05344     -0.02625
C                 -2.46798     -1.18636     -0.28651
C                 -2.68340      1.08968      0.46228
C                 -3.83330     -1.39271     -0.07283
H                 -1.86822     -2.01189     -0.65515
C                 -4.04052      0.89835      0.68079
H                 -2.22409      2.05052      0.66785
C                 -4.59535     -0.34570      0.40678
H                 -4.29985     -2.35187     -0.27299
H                 -4.67322      1.69588      1.05729
C                  5.70225     -1.59667      0.74587
H                  6.58251     -1.48947      1.39165
```

H	4.93551	-2.17518	1.26227
H	6.00071	-2.13169	-0.16240
C	6.23173	0.58644	-0.36321
H	5.83986	1.57072	-0.63598
H	7.11657	0.71654	0.26945
H	6.52493	0.06495	-1.27999
C	4.69172	0.55949	1.61046
H	5.55691	0.80991	2.23586
H	4.19477	1.48319	1.29716
H	3.98121	-0.03053	2.19117
F	-5.92851	-0.53375	0.61812

p-Me--oximyl-radical-cyclisation-tBu-P

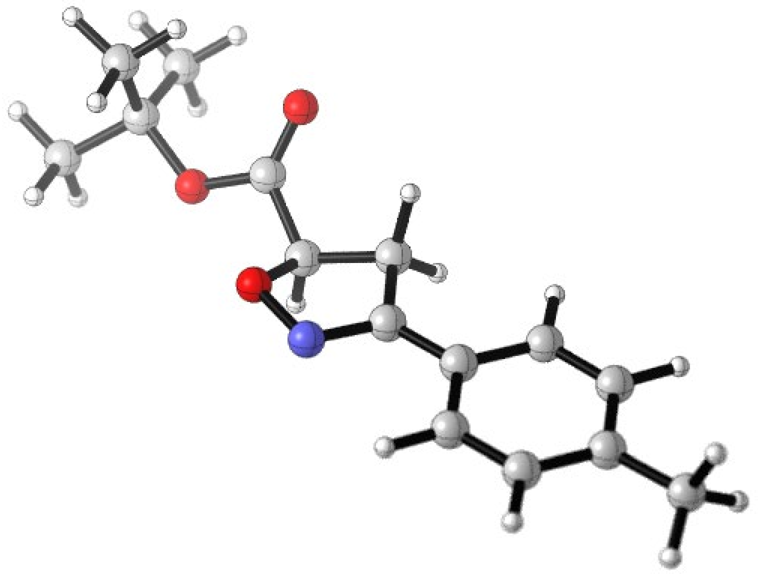

Sum of Electronic and Zero-point Energies = -862.794499 Hartree
Sum of Electronic and Thermal Energies = -862.775141 Hartree
Sum of Electronic and Thermal Enthalpies = -862.774197 Hartree
Sum of Electronic and Thermal Free Energies = -862.844665 Hartree

Dipole Moment = 7.4296 Debye

-1 2

C	1.59452	-0.41839	-0.65592
C	0.35715	-1.00619	-0.00285
C	-0.59455	0.15351	-0.20209
H	1.64163	-0.67697	-1.72715
H	0.53658	-1.23056	1.05711
H	0.02788	-1.92627	-0.49743
N	0.04631	1.29187	-0.50304
O	1.45568	0.98880	-0.51275

C	2.91623	-0.78059	-0.00379
O	3.05307	-1.37497	1.04147
O	3.93877	-0.32266	-0.74956
C	5.30097	-0.37114	-0.25829
C	-1.99855	0.05583	-0.08131
C	-2.65695	-1.16838	0.26474
C	-2.85545	1.18800	-0.30325
C	-4.03374	-1.24490	0.37345
H	-2.06334	-2.06064	0.45162
C	-4.22756	1.07979	-0.18763
H	-2.39720	2.13442	-0.57359
C	-4.86826	-0.13001	0.15171
H	-4.48793	-2.20023	0.64009
H	-4.84077	1.96403	-0.36842
C	5.41840	0.41121	1.04907
H	4.90158	-0.09819	1.86304
H	6.47631	0.52120	1.31012
H	4.98283	1.40675	0.92253
C	5.76489	-1.81940	-0.10683
H	5.60662	-2.36106	-1.04485
H	6.83568	-1.83405	0.12326
H	5.22084	-2.32404	0.69212
C	6.08702	0.32625	-1.36476
H	7.15083	0.35593	-1.10947
H	5.96431	-0.20928	-2.31063
H	5.72209	1.34830	-1.49676
C	-6.36033	-0.21495	0.33367
H	-6.67073	-0.09770	1.38397
H	-6.87295	0.56711	-0.23912
H	-6.75346	-1.18170	-0.00659

p-Me--oximyl-radical-cyclisation-tBu-TS

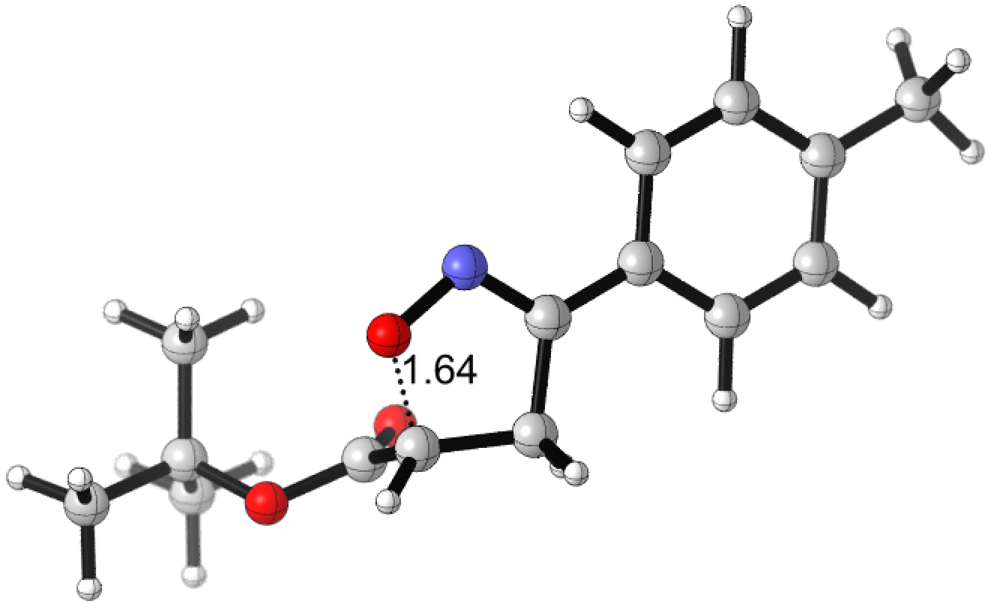

Sum of Electronic and Zero-point Energies = -862.779409 Hartree
Sum of Electronic and Thermal Energies = -862.760312 Hartree
Sum of Electronic and Thermal Enthalpies = -862.759368 Hartree
Sum of Electronic and Thermal Free Energies = -862.829636 Hartree

Dipole Moment = 5.0901 Debye

-1 2

C	-1.88287	0.10436	1.11285
C	-0.46113	-0.32539	1.42962
C	0.36076	0.41019	0.40017
H	-2.47501	0.50946	1.93229
H	-0.36035	-1.40846	1.31058
H	-0.17421	-0.04608	2.45320
N	-0.29380	1.35808	-0.20925
O	-1.53079	1.49020	0.31027
C	-2.62948	-0.79660	0.25492
O	-2.14089	-1.59348	-0.56907
O	-4.00994	-0.57754	0.33827
C	-4.76905	-0.58239	-0.87842
C	1.73124	0.08762	0.04578
C	2.41797	-0.97868	0.65386
C	2.44414	0.85937	-0.90128
C	3.74379	-1.26112	0.33125
H	1.90538	-1.59760	1.38439
C	3.76049	0.56450	-1.21592
H	1.93016	1.68676	-1.37997
C	4.44480	-0.50017	-0.60685

H	4.24285	-2.09687	0.81904
H	4.27988	1.17363	-1.95497
C	-4.99708	-2.01490	-1.37049
H	-5.66101	-2.01207	-2.24404
H	-4.04225	-2.47332	-1.63070
H	-5.46692	-2.60570	-0.57638
C	-4.07827	0.27101	-1.94472
H	-4.73972	0.39076	-2.81083
H	-3.84017	1.25653	-1.53229
H	-3.14452	-0.19617	-2.26530
C	-6.10144	0.05054	-0.47907
H	-6.78999	0.06824	-1.33123
H	-6.55879	-0.52426	0.33283
H	-5.93968	1.07480	-0.12976
C	5.88341	-0.79348	-0.95146
H	6.02072	-0.91190	-2.03255
H	6.55009	0.01475	-0.62656
H	6.22154	-1.71570	-0.46926

p-Me--oximyl-radical-Nu-attack-tBu-P

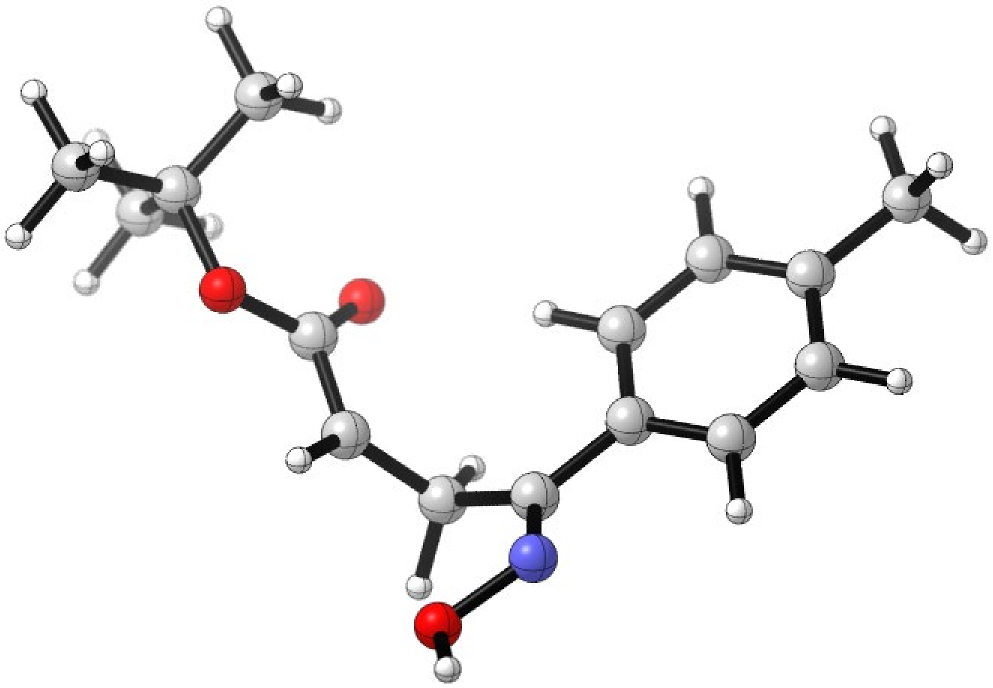

Sum of Electronic and Zero-point Energies = -863.336667 Hartree
Sum of Electronic and Thermal Energies = -863.316594 Hartree
Sum of Electronic and Thermal Enthalpies = -863.315650 Hartree
Sum of Electronic and Thermal Free Energies = -863.387773 Hartree

Dipole Moment = 1.1599 Debye

0 2

C	3.82246	-0.77740	-0.52503
C	2.96759	0.30275	-0.36439
C	1.64739	0.10634	0.06845
C	1.21382	-1.19531	0.33522
C	2.08199	-2.27420	0.16550
C	3.39537	-2.08675	-0.26289
H	4.84435	-0.60657	-0.85552
H	3.31100	1.31150	-0.56759
H	0.19568	-1.38431	0.66525
H	1.72435	-3.27941	0.37226
C	0.72509	1.25779	0.22186
N	1.01661	2.33160	-0.41093
O	0.06986	3.33045	-0.20312
H	0.41805	4.07355	-0.70865
C	-0.52692	1.13635	1.07629
C	-1.72342	0.90678	0.22074
H	-2.03978	1.66599	-0.48429
H	-0.42024	0.32305	1.79501
H	-0.64230	2.08168	1.61903
C	-2.45766	-0.34608	0.28888
O	-2.14976	-1.27529	1.02161
O	-3.50831	-0.34703	-0.55135
C	-4.38225	-1.50850	-0.65996
C	-5.39788	-1.07021	-1.70937
H	-6.12439	-1.86915	-1.88132
H	-5.93092	-0.17658	-1.37333
H	-4.89559	-0.84226	-2.65347
C	-5.07285	-1.77453	0.67577
H	-4.36155	-2.10803	1.43191
H	-5.57211	-0.86619	1.02664
H	-5.83195	-2.55058	0.53763
C	-3.59161	-2.71844	-1.15319
H	-3.04743	-2.46290	-2.06750
H	-2.88391	-3.06587	-0.39999
H	-4.28808	-3.53021	-1.38447
C	4.34202	-3.24739	-0.43073
H	3.82981	-4.20045	-0.28036
H	4.78353	-3.25182	-1.43192
H	5.16377	-3.18803	0.29023

p-Me--oximyl-radical-Nu-attack-tBu-TS

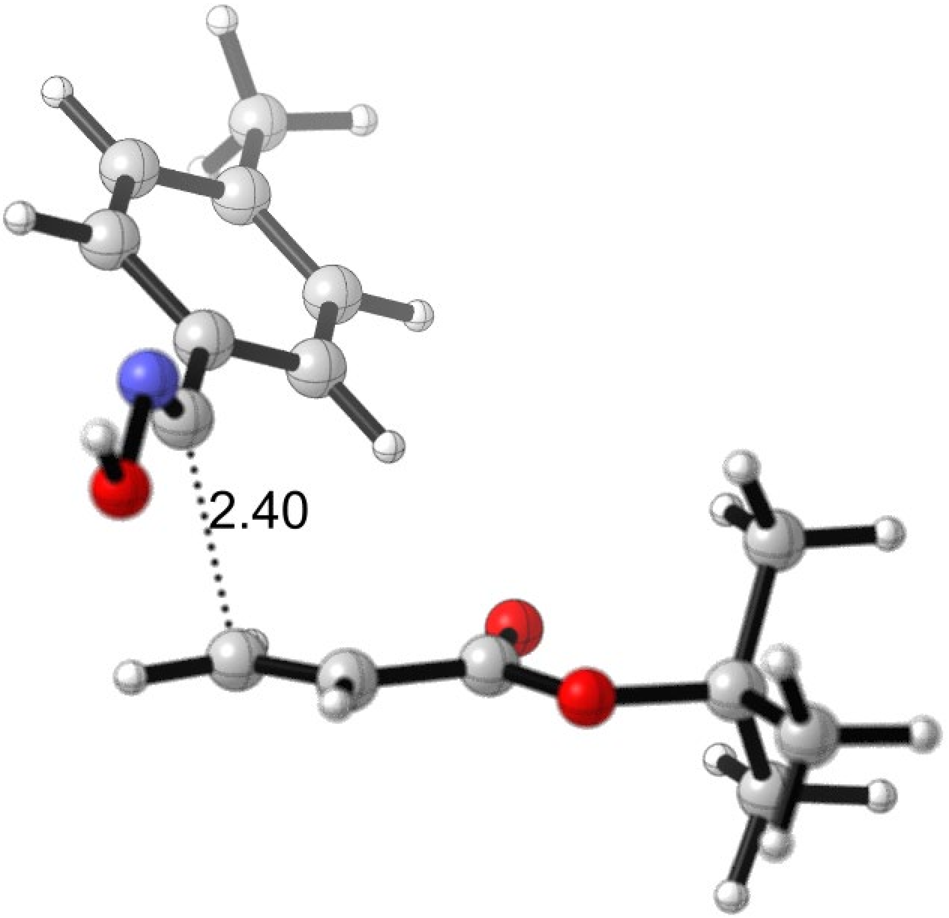

Sum of Electronic and Zero-point Energies = -863.279399 Hartree
Sum of Electronic and Thermal Energies = -863.258490 Hartree
Sum of Electronic and Thermal Enthalpies = -863.257546 Hartree
Sum of Electronic and Thermal Free Energies = -863.332140 Hartree

Dipole Moment = 0.9759 Debye

0 2

C	-2.38913	-2.13850	0.04967
C	-1.43353	-1.12860	0.07683
C	-1.83424	0.20989	-0.04517
C	-3.20065	0.51451	-0.16256
C	-4.14022	-0.50766	-0.18150
C	-3.75194	-1.84893	-0.07531
H	-2.06921	-3.17393	0.13201
H	-0.37909	-1.37017	0.18694
H	-3.50736	1.55209	-0.24867
H	-5.19476	-0.26343	-0.28241
C	-0.85678	1.27700	-0.00944
N	-0.72937	2.29211	-0.71657
O	0.25781	3.19840	-0.30830
H	0.22472	3.87678	-0.99406
C	0.94278	0.98001	1.54391
C	2.04922	0.84184	0.78404

H	2.62184	1.69574	0.44122
H	0.65717	1.95223	1.92974
H	0.43564	0.10076	1.93159
C	2.48040	-0.49779	0.33618
O	1.86465	-1.52669	0.54727
O	3.63751	-0.42835	-0.33912
C	4.26537	-1.62400	-0.88839
C	3.34960	-2.27065	-1.92540
H	3.89830	-3.06500	-2.44077
H	2.46186	-2.69965	-1.45994
H	3.04242	-1.52866	-2.66871
C	5.52058	-1.07120	-1.55473
H	6.15297	-0.56914	-0.81741
H	6.09034	-1.88566	-2.01057
H	5.25273	-0.35078	-2.33250
C	4.63631	-2.58772	0.23643
H	5.23899	-3.40298	-0.17559
H	5.23329	-2.06880	0.99255
H	3.74808	-3.00871	0.70824
C	-4.78186	-2.94871	-0.06167
H	-5.16303	-3.10882	0.95272
H	-5.63417	-2.70032	-0.69948
H	-4.35613	-3.89316	-0.40908

p-Me--oximyl-radical

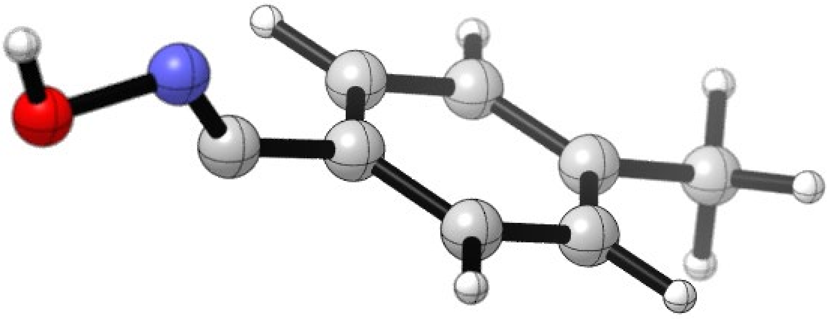

Sum of Electronic and Zero-point Energies = -439.207789 Hartree
Sum of Electronic and Thermal Energies = -439.197866 Hartree
Sum of Electronic and Thermal Enthalpies = -439.196922 Hartree
Sum of Electronic and Thermal Free Energies = -439.246098 Hartree

Dipole Moment = 2.1125 Debye

0 2

C	-2.38920	-2.04374	0.03512
C	-1.51017	-0.97033	0.05045
C	-2.00039	0.34425	-0.06312
C	-3.38947	0.54816	-0.15905
C	-4.25240	-0.53865	-0.17290
C	-3.77223	-1.85033	-0.07295
H	-1.99708	-3.05488	0.10753
H	-0.44092	-1.13370	0.13748
H	-3.77495	1.55981	-0.23350
H	-5.32207	-0.36865	-0.26335
C	-1.10592	1.45755	-0.00834
N	-0.44029	2.14438	-0.79013
O	0.32412	3.17210	-0.21845
H	0.75130	3.57254	-0.98620
C	-4.71956	-3.02117	-0.04902
H	-4.99779	-3.27290	0.98022
H	-5.63912	-2.79730	-0.59539
H	-4.26280	-3.90858	-0.49447

p-Me-oximyl-radical-Nu-attack-tBu-P-N-oxide

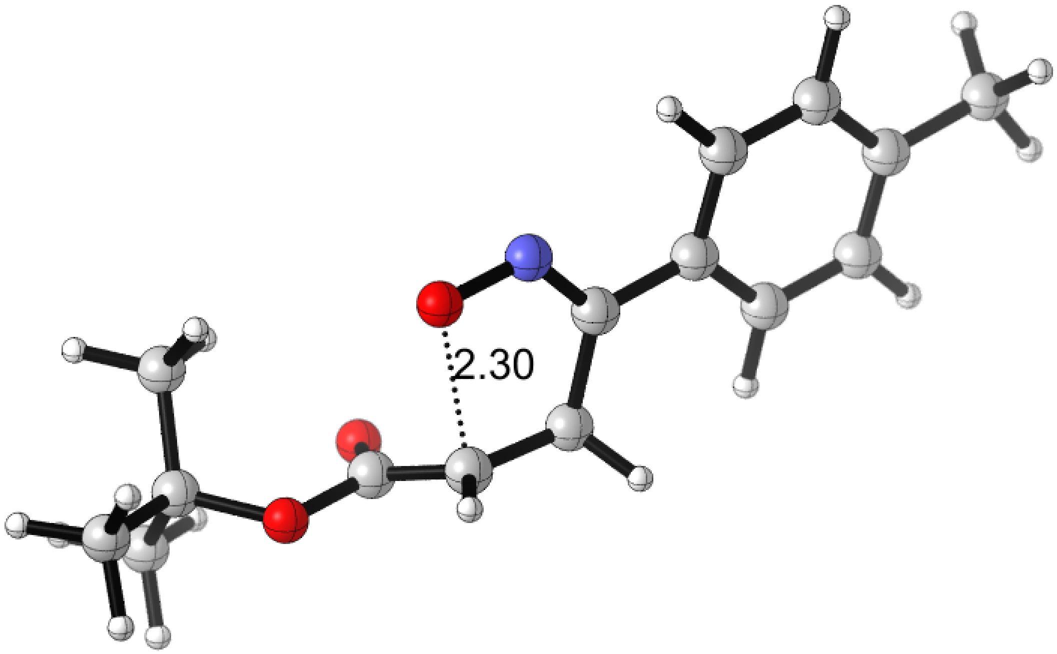

Sum of Electronic and Zero-point Energies = -862.793223 Hartree
Sum of Electronic and Thermal Energies = -862.773590 Hartree
Sum of Electronic and Thermal Enthalpies = -862.772646 Hartree
Sum of Electronic and Thermal Free Energies = -862.843942 Hartree

Dipole Moment = 2.9048 Debye

-1 2

C	1.19458400	-0.41059800	-1.17616000
C	-0.20647800	-0.81914900	-0.89988800
C	-1.07451500	0.34812600	-0.42928600
H	1.47281300	0.01800200	-2.12977000
H	-0.20488700	-1.59457300	-0.12075000
H	-0.67621600	-1.25129000	-1.79667200
C	2.21830200	-0.70488900	-0.24597800
O	2.09153600	-1.26059500	0.85073700
O	3.45945600	-0.26873500	-0.69149000
C	4.48876900	0.01162600	0.26541700
N	-0.55271200	1.51683300	-0.22374700
O	0.69852100	1.70468700	-0.43616000
C	-2.51021300	0.16932300	-0.18168800
C	-3.12247700	-1.08738900	-0.30290600
C	-3.33874100	1.25225200	0.18320300

C	-4.49009900	-1.25449200	-0.07110300
H	-2.52547800	-1.95233600	-0.57450400
C	-4.69347600	1.07564900	0.41227100
H	-2.88283800	2.23187700	0.28188400
C	-5.30294000	-0.18339600	0.28999800
H	-4.92816900	-2.24601800	-0.17342900
H	-5.30201100	1.93452300	0.69301100
C	5.06804500	-1.28256200	0.84321000
H	5.92667200	-1.05295900	1.48620100
H	4.30619600	-1.80852900	1.41959400
H	5.40756900	-1.93080100	0.02769500
C	5.55099000	0.73365700	-0.56331200
H	5.13754300	1.65993900	-0.97318800
H	6.42298100	0.97393900	0.05482800
H	5.87079700	0.09935600	-1.39636300
C	3.96876200	0.94018900	1.36622800
H	4.81223500	1.32413200	1.95240500
H	3.43227500	1.77956700	0.91220400
H	3.27774900	0.41201400	2.02500100
C	-6.77892100	-0.35690200	0.54956100
H	-7.03364900	-0.11570100	1.58811600
H	-7.37860400	0.29781200	-0.09293000
H	-7.09164800	-1.38831700	0.36079600

p-NO2--oximyl-radical-cyclisation-tBu-P

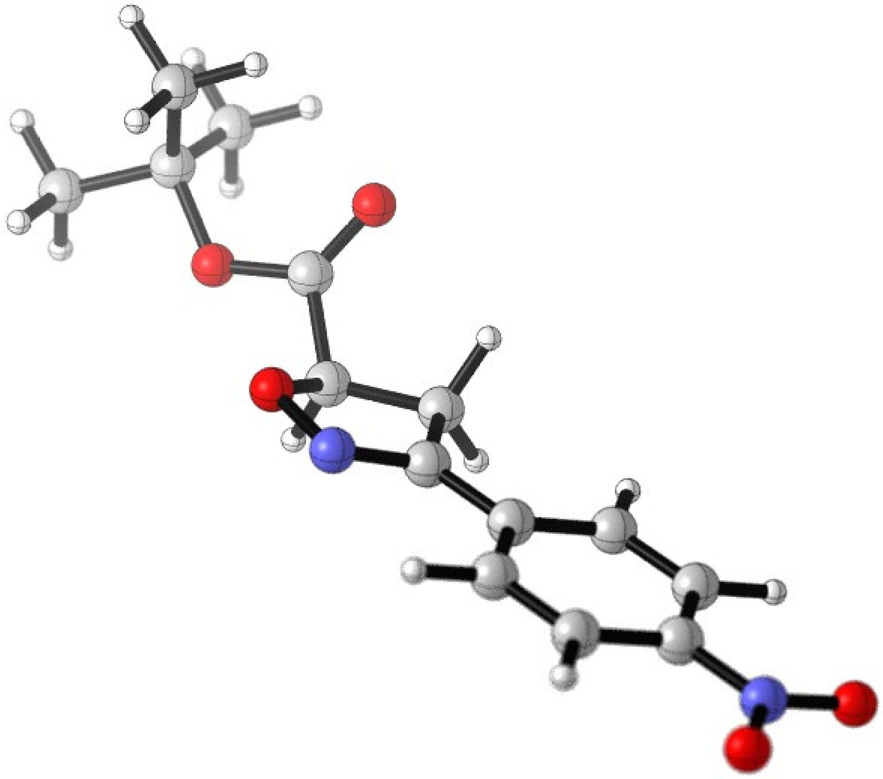

```
Sum of Electronic and Zero-point Energies = -1028.008110 Hartree
Sum of Electronic and Thermal Energies = -1027.988455 Hartree
Sum of Electronic and Thermal Enthalpies = -1027.987511 Hartree
Sum of Electronic and Thermal Free Energies = -1028.058972 Hartree

Dipole Moment = 22.9044 Debye

-1 2
C        1.59395   -0.44345   -0.86339
C        0.33278   -1.05817   -0.28584
C       -0.59780    0.13761   -0.34949
H        1.73611   -0.69486   -1.92311
H        0.48027   -1.38559    0.74935
H       -0.03113   -1.90345   -0.87319
N        0.03086    1.24455   -0.59258
O        1.41011    0.97471   -0.75034
C        2.86951   -0.76076   -0.09710
O        2.91390   -1.28605    0.99068
O        3.93471   -0.34371   -0.79353
C        5.26733   -0.36479   -0.20944
C       -2.02058    0.05528   -0.13068
C       -2.63932   -1.18006    0.18474
C       -2.85158    1.20481   -0.22210
```

C	-3.99729	-1.27859	0.39551
H	-2.03237	-2.07832	0.27185
C	-4.20794	1.12648	-0.01757
H	-2.39166	2.15878	-0.46237
C	-4.81017	-0.12177	0.29547
H	-4.47170	-2.22102	0.63859
H	-4.84696	1.99797	-0.08894
C	5.29797	0.49570	1.05181
H	4.72156	0.04086	1.85804
H	6.33559	0.61386	1.38004
H	4.88594	1.48572	0.83554
C	5.70422	-1.80326	0.06002
H	5.60438	-2.40160	-0.85101
H	6.75695	-1.80835	0.36063
H	5.10686	-2.25548	0.85228
C	6.12642	0.26053	-1.30340
H	7.17084	0.30232	-0.98064
H	6.06361	-0.33206	-2.22051
H	5.78051	1.27449	-1.52098
O	-6.88122	0.83822	0.40268
O	-6.68674	-1.33372	0.77429
N	-6.17949	-0.20865	0.49912

p-NO2--oximyl-radical-cyclisation-tBu-TS-ii

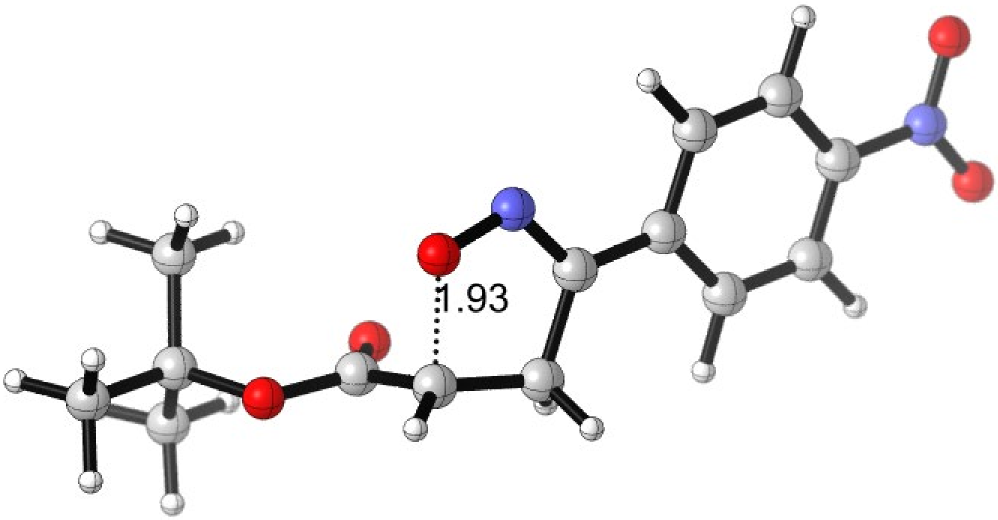


```
Sum of Electronic and Zero-point Energies = -1027.964899 Hartree
Sum of Electronic and Thermal Energies = -1027.945263 Hartree
Sum of Electronic and Thermal Enthalpies = -1027.944319 Hartree
Sum of Electronic and Thermal Free Energies = -1028.015610 Hartree

Dipole Moment = 8.5365 Debye

-1 2
C              -1.83128       0.02490       1.09675
C              -0.41596      -0.42373       1.27270
C               0.46212       0.41759       0.35145
H              -2.36534       0.48204       1.92035
H              -0.32185      -1.47548       0.97905
H              -0.09188      -0.31958       2.31617
N              -0.13676       1.43977      -0.20781
O              -1.32804       1.67760       0.24558
C              -2.62545      -0.62377       0.08922
O              -2.19225      -1.36037      -0.79655
O              -3.95504      -0.28217       0.19164
C              -4.85303      -0.55290      -0.89823
C               1.82748       0.09972       0.03581
C               2.44650      -1.07202       0.53884
C               2.62481       0.97627      -0.75094
```

C	3.77202	-1.35947	0.27601
H	1.87063	-1.76208	1.14638
C	3.94284	0.69256	-1.02789
H	2.16391	1.87915	-1.13603
C	4.52509	-0.47741	-0.50918
H	4.24370	-2.25459	0.66346
H	4.54735	1.35464	-1.63643
C	-5.06061	-2.05868	-1.07645
H	-5.84929	-2.23608	-1.81669
H	-4.13808	-2.53672	-1.40663
H	-5.37195	-2.50379	-0.12546
C	-4.35144	0.10777	-2.18386
H	-5.12871	0.04844	-2.95408
H	-4.12502	1.16090	-1.99040
H	-3.44627	-0.38096	-2.54590
C	-6.15650	0.10372	-0.44677
H	-6.93724	-0.04376	-1.20002
H	-6.49031	-0.33377	0.49917
H	-6.00404	1.17651	-0.29744
O	6.54663	0.02798	-1.46704
O	6.38635	-1.80539	-0.32232
N	5.89843	-0.76837	-0.78189

p-NO2--oximyl-radical-Nu-attack-tBu-P

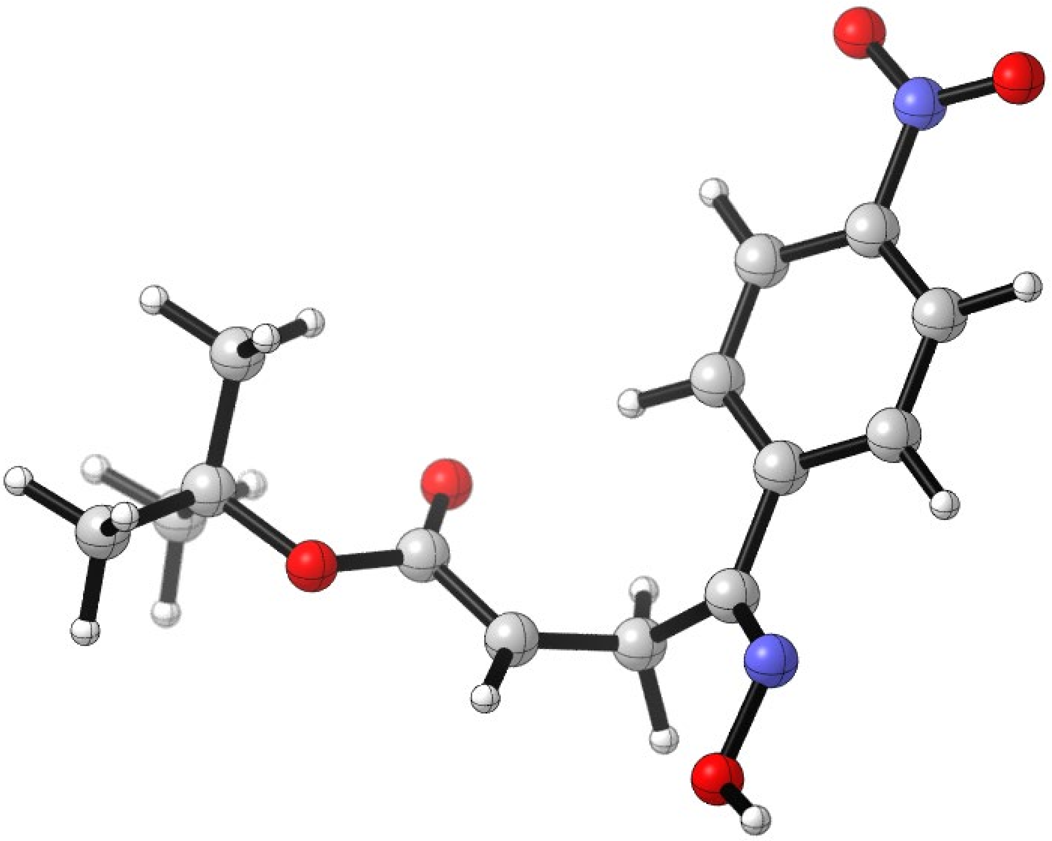

Sum of Electronic and Zero-point Energies = -1028.496000 Hartree
Sum of Electronic and Thermal Energies = -1028.475315 Hartree
Sum of Electronic and Thermal Enthalpies = -1028.474371 Hartree
Sum of Electronic and Thermal Free Energies = -1028.548178 Hartree

Dipole Moment = 6.5976 Debye

0 2

C	3.83192	-0.79105	-0.50548
C	2.97056	0.28358	-0.34494
C	1.64924	0.08230	0.08374
C	1.20089	-1.21662	0.35516
C	2.05578	-2.30343	0.19372
C	3.35463	-2.06904	-0.23220
H	4.85674	-0.65768	-0.82958
H	3.31306	1.29202	-0.54625
H	0.18024	-1.39537	0.68136
H	1.72709	-3.31621	0.39214
C	0.73026	1.23933	0.23519
N	1.03749	2.30209	-0.40831
O	0.10731	3.30996	-0.21993
H	0.46165	4.04452	-0.73478
C	-0.52251	1.13608	1.08879

C	-1.72522	0.91984	0.23616
H	-2.05916	1.69671	-0.44075
H	-0.42574	0.32326	1.80982
H	-0.62357	2.08363	1.62996
C	-2.43986	-0.34734	0.27189
O	-2.09803	-1.29509	0.96554
O	-3.50399	-0.33587	-0.54529
C	-4.36442	-1.50809	-0.67972
C	-5.40531	-1.04719	-1.69361
H	-6.12291	-1.85055	-1.88090
H	-5.94534	-0.17485	-1.31559
H	-4.92463	-0.77819	-2.63812
C	-5.02379	-1.83018	0.65899
H	-4.29369	-2.18054	1.38914
H	-5.52926	-0.94264	1.05154
H	-5.77416	-2.61208	0.50794
C	-3.56403	-2.68628	-1.22968
H	-3.04238	-2.39131	-2.14524
H	-2.83705	-3.04915	-0.50248
H	-4.25195	-3.50076	-1.47575
O	5.40298	-2.98255	-0.76676
O	3.82443	-4.32570	-0.16029
N	4.26485	-3.21532	-0.39899

p-NO2--oximyl-radical-Nu-attack-tBu-TS

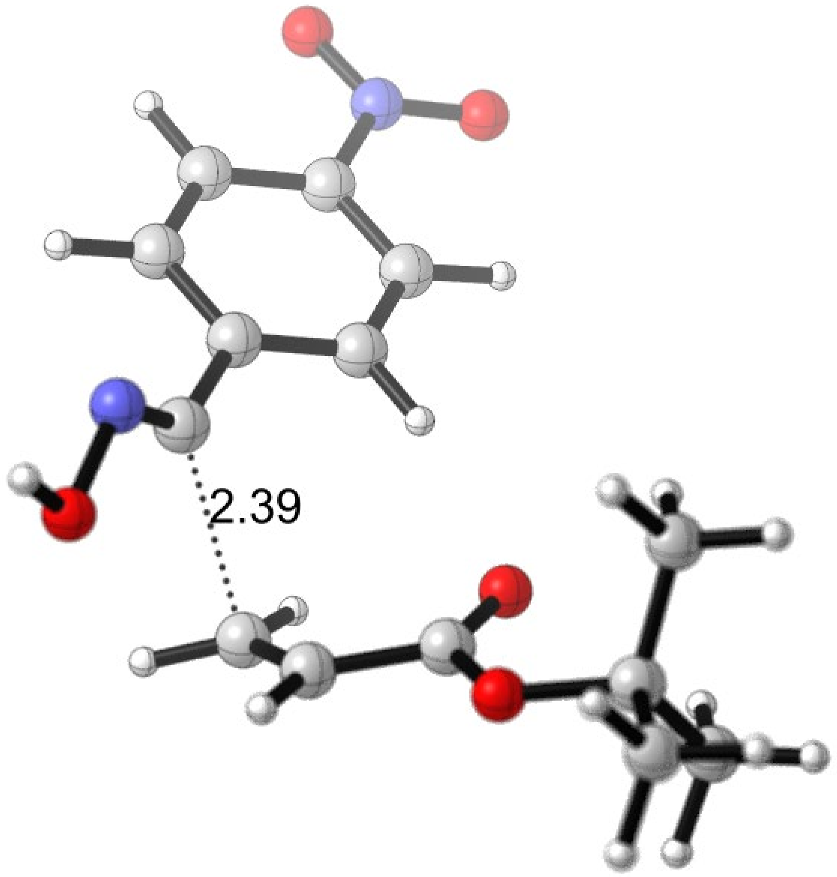

Sum of Electronic and Zero-point Energies = -1028.437790 Hartree
Sum of Electronic and Thermal Energies = -1028.416270 Hartree
Sum of Electronic and Thermal Enthalpies = -1028.415326 Hartree
Sum of Electronic and Thermal Free Energies = -1028.491752 Hartree

Dipole Moment = 6.2887 Debye

0 2

C	-2.41647	-2.18198	0.04670
C	-1.45588	-1.17923	0.02717
C	-1.85359	0.16539	-0.06421
C	-3.21948	0.49984	-0.10215
C	-4.18127	-0.49924	-0.07831
C	-3.75870	-1.82252	-0.00581
H	-2.14191	-3.22801	0.10698
H	-0.39944	-1.42867	0.08423
H	-3.51118	1.54276	-0.15998
H	-5.24005	-0.27395	-0.11655
C	-0.86461	1.21996	-0.05975
N	-0.69412	2.18920	-0.81715
O	0.29112	3.09964	-0.43917
H	0.28918	3.73937	-1.16266
C	0.89763	0.93873	1.53490

C	2.01282	0.82717	0.78458
H	2.59333	1.69115	0.48280
H	0.60228	1.89781	1.94617
H	0.39071	0.04615	1.89148
C	2.44923	-0.49926	0.29516
O	1.80325	-1.52289	0.42732
O	3.63712	-0.41802	-0.31444
C	4.28150	-1.59927	-0.88320
C	3.41477	-2.18483	-1.99532
H	3.98136	-2.96224	-2.51674
H	2.49929	-2.62403	-1.59822
H	3.15636	-1.40635	-2.71956
C	5.57706	-1.03434	-1.45444
H	6.17374	-0.57387	-0.66237
H	6.16161	-1.83602	-1.91387
H	5.36099	-0.27760	-2.21356
C	4.57692	-2.61504	0.21737
H	5.18813	-3.42230	-0.19738
H	5.14102	-2.13944	1.02532
H	3.65911	-3.04152	0.62314
O	-4.38857	-4.03836	0.08292
O	-5.94725	-2.54662	-0.02033
N	-4.77740	-2.88563	0.02135

p-NO2--oximyl-radical

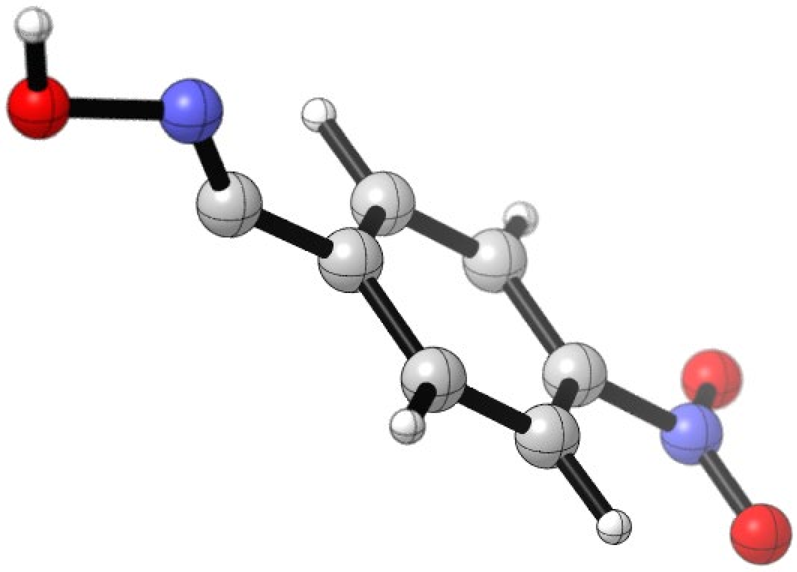

Sum of Electronic and Zero-point Energies = -604.365992 Hartree
Sum of Electronic and Thermal Energies = -604.355533 Hartree
Sum of Electronic and Thermal Enthalpies = -604.354589 Hartree
Sum of Electronic and Thermal Free Energies = -604.404102 Hartree

Dipole Moment = 4.6238 Debye

0 2

C	-2.40945	-2.10763	0.01555
C	-1.51906	-1.05048	-0.05056
C	-1.99836	0.27636	-0.14145
C	-3.39081	0.52012	-0.13071
C	-4.28050	-0.53762	-0.06445
C	-3.77550	-1.83401	0.00868
H	-2.06954	-3.13452	0.07232
H	-0.45020	-1.23179	-0.04202
H	-3.75399	1.54030	-0.18323
H	-5.35215	-0.38018	-0.06776
C	-1.08846	1.35867	-0.16002
N	-0.43172	2.09513	-0.89229
O	0.36139	3.07104	-0.28538
H	0.77427	3.51543	-1.03764
O	-4.25548	-4.08098	0.14418
O	-5.91017	-2.69233	0.07597
N	-4.71989	-2.95556	0.08208

p-NO2-oximyl-radical-Nu-attack-tBu-P-N-oxide

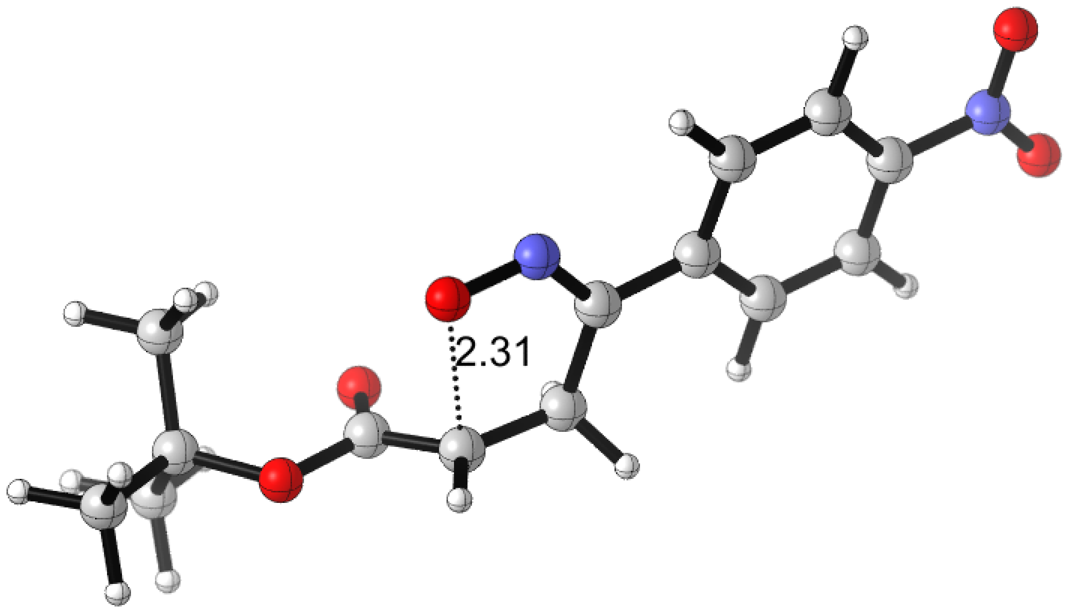

Sum of Electronic and Zero-point Energies = -1027.971622 Hartree
Sum of Electronic and Thermal Energies = -1027.951375 Hartree
Sum of Electronic and Thermal Enthalpies = -1027.950431 Hartree
Sum of Electronic and Thermal Free Energies = -1028.023576 Hartree

Dipole Moment = 4.4463 Debye

-1 2

C	1.81757	-0.40265	-1.07367
C	0.42154	-0.84999	-0.83532
C	-0.46720	0.25597	-0.26819
H	2.09444	0.10776	-1.98652
H	0.43595	-1.69114	-0.12826
H	-0.03278	-1.20786	-1.77104
C	2.84530	-0.74852	-0.15267
O	2.70709	-1.39600	0.88635
O	4.06874	-0.25059	-0.54653
C	5.15941	-0.20248	0.38709
N	0.03427	1.42841	0.04386
O	1.26883	1.64437	-0.14805
C	-1.88058	0.04925	-0.04036
C	-2.48525	-1.20393	-0.30200
C	-2.71892	1.08754	0.44615
C	-3.83939	-1.41338	-0.09826
H	-1.87826	-2.02620	-0.66417
C	-4.06672	0.88846	0.65239
H	-2.26757	2.05069	0.65355
C	-4.62744	-0.36629	0.37598
H	-4.29729	-2.37500	-0.29888
H	-4.70348	1.68317	1.02291
C	5.66747	-1.60953	0.70945
H	6.57239	-1.54310	1.32500
H	4.90462	-2.17666	1.24359
H	5.91776	-2.13396	-0.21897
C	6.23132	0.57895	-0.37111
H	5.86019	1.57781	-0.61815
H	7.13615	0.67476	0.23840
H	6.48512	0.06392	-1.30290
C	4.75685	0.55963	1.65178
H	5.64882	0.76735	2.25406
H	4.28767	1.50828	1.37273
H	4.04485	-0.01593	2.24452
N	-6.04085	-0.57911	0.58618
O	-6.50997	-1.68637	0.33728
O	-6.71619	0.35674	1.00475

p-OMe--oximyl-radical-cyclisation-tBu-P

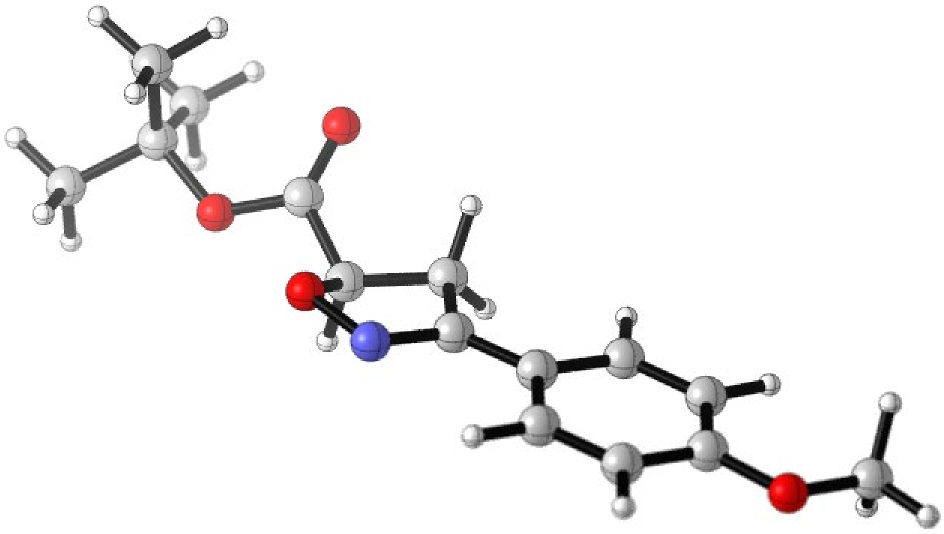

Sum of Electronic and Zero-point Energies = -937.968770 Hartree
Sum of Electronic and Thermal Energies = -937.948613 Hartree
Sum of Electronic and Thermal Enthalpies = -937.947669 Hartree
Sum of Electronic and Thermal Free Energies = -938.020080 Hartree

Dipole Moment = 7.3120 Debye

-1 2

C	1.61642	-0.43313	-0.65208
C	0.38814	-1.03846	0.00350
C	-0.57543	0.11073	-0.18488
H	1.66000	-0.68607	-1.72516
H	0.57552	-1.27188	1.06069
H	0.06637	-1.95899	-0.49732
N	0.05039	1.26031	-0.49306
O	1.46435	0.97096	-0.49982
C	2.94546	-0.78649	-0.01026
O	3.09542	-1.39210	1.02687
O	3.95906	-0.30716	-0.75523
C	5.32385	-0.34516	-0.27122
C	-1.97630	-0.00610	-0.05459
C	-2.62256	-1.23172	0.29896
C	-2.84470	1.12067	-0.27001
C	-4.00621	-1.32992	0.41659
H	-2.02355	-2.11743	0.49491
C	-4.21250	1.01066	-0.15102
H	-2.39348	2.07202	-0.53428
C	-4.81830	-0.21283	0.19146
H	-4.43694	-2.28881	0.69018
H	-4.85547	1.87158	-0.31971
C	5.43869	0.42183	1.04549
H	4.93348	-0.10485	1.85573

H	6.49657	0.54272	1.30184
H	4.98898	1.41290	0.93428
C	5.80634	-1.78943	-0.14061
H	5.65085	-2.32065	-1.08506
H	6.87819	-1.79391	0.08511
H	5.27162	-2.31108	0.65372
C	6.09594	0.37589	-1.37238
H	7.16066	0.41498	-1.12207
H	5.97463	-0.14876	-2.32451
H	5.71810	1.39509	-1.48925
O	-6.20880	-0.20470	0.28580
C	-6.81130	-1.41552	0.63617
H	-7.88847	-1.23475	0.66621
H	-6.59833	-2.20489	-0.10055
H	-6.47644	-1.76641	1.62402

p-OMe--oximyl-radical-cyclisation-tBu-TS

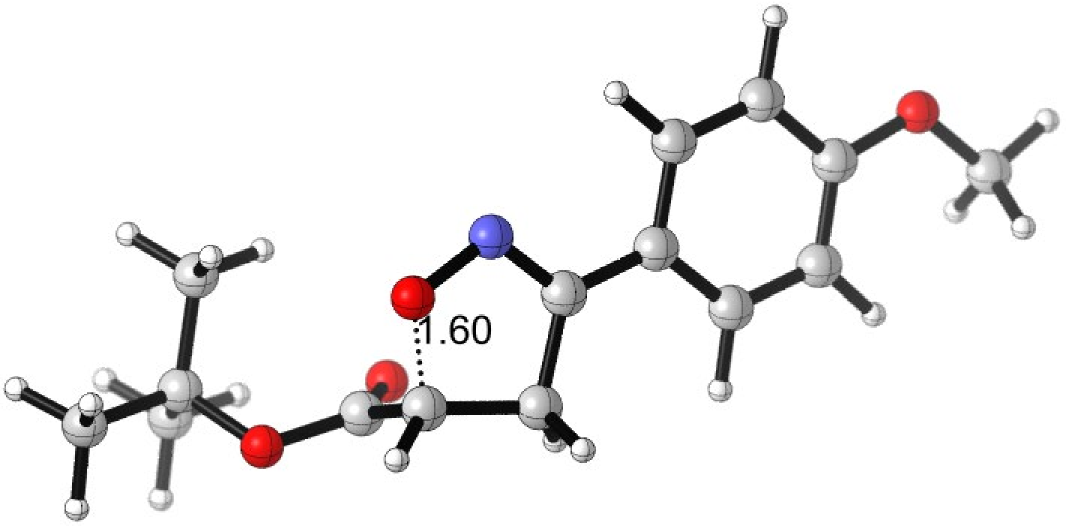

Sum of Electronic and Zero-point Energies = -937.956444 Hartree
Sum of Electronic and Thermal Energies = -937.936721 Hartree
Sum of Electronic and Thermal Enthalpies = -937.935777 Hartree
Sum of Electronic and Thermal Free Energies = -938.006623 Hartree

Dipole Moment = 5.8649 Debye

-1 2

C	-1.91816	0.15037	1.11671
C	-0.50242	-0.27814	1.47551
C	0.32308	0.39955	0.41309
H	-2.52000	0.57511	1.92058
H	-0.41226	-1.36698	1.41661

H	-0.22147	0.05544	2.48460
N	-0.32723	1.32023	-0.24005
O	-1.57355	1.46468	0.27611
C	-2.65811	-0.80615	0.30324
O	-2.15647	-1.63277	-0.48603
O	-4.04122	-0.58402	0.35551
C	-4.76613	-0.57668	-0.88266
C	1.69297	0.05476	0.07464
C	2.37798	-0.97639	0.73380
C	2.40541	0.77356	-0.91559
C	3.70572	-1.29258	0.43184
H	1.86679	-1.55376	1.49831
C	3.71776	0.46543	-1.22175
H	1.89242	1.57572	-1.43611
C	4.38112	-0.57057	-0.55037
H	4.18835	-2.10236	0.96793
H	4.26061	1.01524	-1.98523
C	-5.00014	-2.00649	-1.37963
H	-5.63363	-1.99627	-2.27555
H	-4.04346	-2.47939	-1.60533
H	-5.50534	-2.58818	-0.60057
C	-4.03158	0.26390	-1.92953
H	-4.66558	0.39238	-2.81476
H	-3.78743	1.24567	-1.51182
H	-3.09638	-0.21891	-2.22247
C	-6.09981	0.07657	-0.52264
H	-6.76454	0.10130	-1.39347
H	-6.58762	-0.48885	0.27811
H	-5.93305	1.09970	-0.17214
O	5.68806	-0.79438	-0.92534
C	6.36199	-1.84432	-0.28171
H	7.36426	-1.87719	-0.71189
H	6.43733	-1.67116	0.80049
H	5.86156	-2.80718	-0.45163

p-OMe--oximyl-radical-Nu-attack-tBu-P

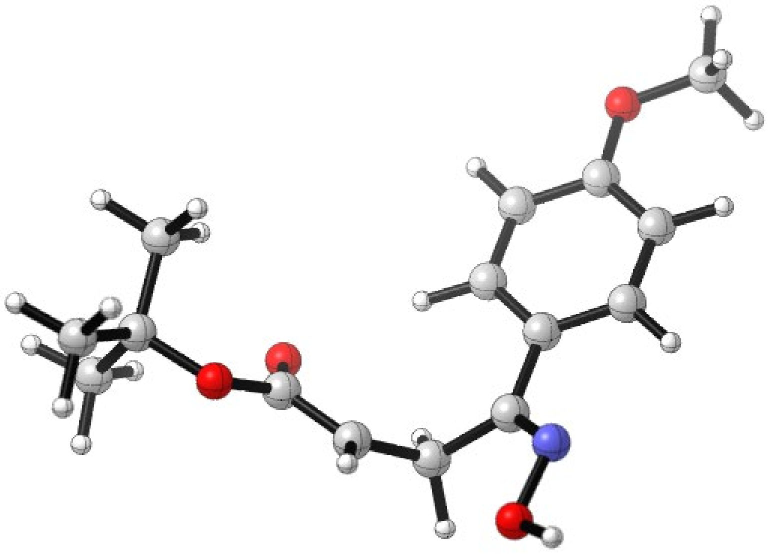

Sum of Electronic and Zero-point Energies = -938.515124 Hartree
Sum of Electronic and Thermal Energies = -938.494420 Hartree
Sum of Electronic and Thermal Enthalpies = -938.493476 Hartree
Sum of Electronic and Thermal Free Energies = -938.566477 Hartree

Dipole Moment = 1.7699 Debye

0 2

C	3.84586	-0.66346	-0.51393
C	2.95534	0.39267	-0.34294
C	1.64063	0.17383	0.07715
C	1.22887	-1.14423	0.33020
C	2.10650	-2.20404	0.15963
C	3.41904	-1.97158	-0.26303
H	4.85998	-0.45523	-0.83290
H	3.28435	1.40865	-0.53392
H	0.21238	-1.35424	0.65263
H	1.79575	-3.22600	0.34812
C	0.69835	1.30655	0.23099
N	0.96694	2.38772	-0.40006
O	0.00346	3.36997	-0.18034
H	0.33416	4.12014	-0.68694
C	-0.54909	1.16031	1.08910
C	-1.74609	0.92508	0.23585
H	-2.07907	1.69080	-0.45423
H	-0.42937	0.34090	1.79877
H	-0.67387	2.09846	1.64211
C	-2.45629	-0.34239	0.28413
O	-2.12698	-1.27915	0.99784
O	-3.51133	-0.34778	-0.55050
C	-4.36393	-1.52336	-0.67595
C	-5.39338	-1.08492	-1.71178
H	-6.10553	-1.89420	-1.89511

H	-5.94143	-0.20805	-1.35629
H	-4.90054	-0.82982	-2.65393
C	-5.04210	-1.82675	0.65817
H	-4.32073	-2.16153	1.40411
H	-5.55565	-0.93434	1.02875
H	-5.78782	-2.61373	0.50941
C	-3.55365	-2.70900	-1.19547
H	-3.02000	-2.42701	-2.10821
H	-2.83488	-3.05645	-0.45287
H	-4.23608	-3.52960	-1.43724
O	4.20524	-3.07132	-0.39519
C	5.53944	-2.88176	-0.81948
H	5.98530	-3.87467	-0.86265
H	5.57702	-2.42065	-1.81344
H	6.09693	-2.26171	-0.10764

p-OMe--oximyl-radical-Nu-attack-tBu-TS

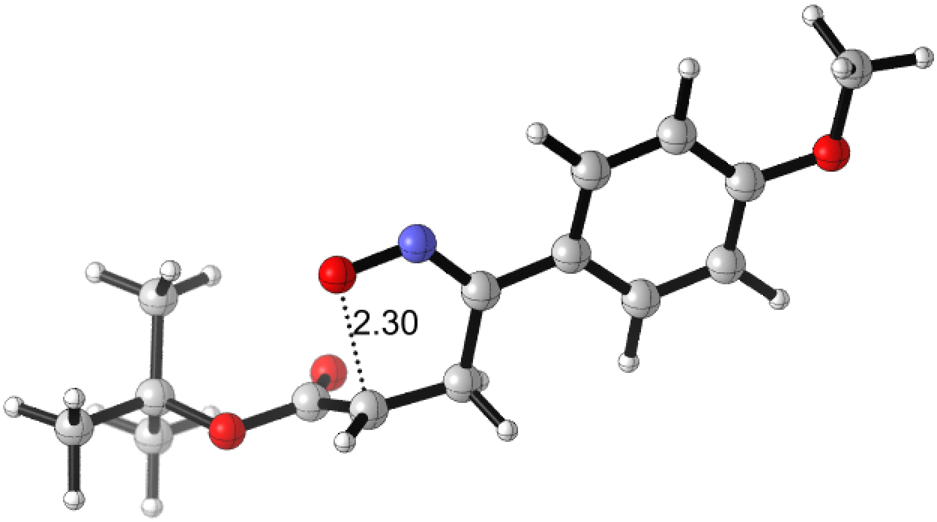

Sum of Electronic and Zero-point Energies = -938.458202 Hartree
Sum of Electronic and Thermal Energies = -938.436624 Hartree
Sum of Electronic and Thermal Enthalpies = -938.435680 Hartree
Sum of Electronic and Thermal Free Energies = -938.511528 Hartree

Dipole Moment = 2.0404 Debye

0 2

C	-2.41060	-2.06273	0.04831
C	-1.44860	-1.06950	0.06692
C	-1.82667	0.28181	-0.05143
C	-3.18601	0.60424	-0.15407
C	-4.15784	-0.39246	-0.16674

C	-3.76841	-1.73230	-0.06834
H	-2.13937	-3.10976	0.12942
H	-0.39776	-1.32905	0.17084
H	-3.47845	1.64656	-0.23175
H	-5.20147	-0.11529	-0.25249
C	-0.83391	1.33132	-0.01883
N	-0.69400	2.34775	-0.72301
O	0.30148	3.24215	-0.30476
H	0.28526	3.91894	-0.99258
C	0.96133	1.00670	1.53524
C	2.06924	0.85444	0.78014
H	2.65803	1.70047	0.44529
H	0.68937	1.98136	1.92464
H	0.43897	0.13390	1.91716
C	2.47848	-0.48937	0.32590
O	1.83881	-1.50721	0.51994
O	3.64532	-0.43894	-0.33444
C	4.25457	-1.64323	-0.88553
C	3.33927	-2.26126	-1.94026
H	3.87777	-3.06268	-2.45545
H	2.43651	-2.67528	-1.49043
H	3.05785	-1.50647	-2.68088
C	5.53030	-1.11216	-1.53030
H	6.16325	-0.62994	-0.78032
H	6.08857	-1.93489	-1.98551
H	5.28848	-0.37967	-2.30533
C	4.58953	-2.62424	0.23566
H	5.17990	-3.44894	-0.17542
H	5.18748	-2.12471	1.00399
H	3.68619	-3.02975	0.69211
O	-4.63105	-2.77842	-0.06932
C	-6.01215	-2.49960	-0.18360
H	-6.51561	-3.46522	-0.16684
H	-6.36289	-1.88963	0.65697
H	-6.23489	-1.98715	-1.12667

p-OMe--oximyl-radical

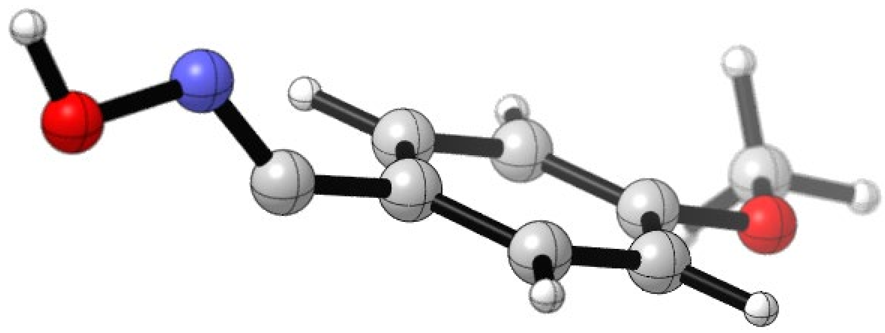

Sum of Electronic and Zero-point Energies = -514.386154 Hartree
Sum of Electronic and Thermal Energies = -514.375701 Hartree
Sum of Electronic and Thermal Enthalpies = -514.374757 Hartree
Sum of Electronic and Thermal Free Energies = -514.423848 Hartree

Dipole Moment = 3.0474 Debye

0 2

C	-2.41198	-2.03783	0.03544
C	-1.47056	-1.02750	0.05577
C	-1.86473	0.32066	-0.05435
C	-3.23119	0.61948	-0.14795
C	-4.18604	-0.39338	-0.16063
C	-3.77643	-1.72761	-0.07150
H	-2.12499	-3.08125	0.10740
H	-0.41630	-1.26639	0.15168
H	-3.54220	1.65669	-0.21909
H	-5.23426	-0.13274	-0.24156
C	-0.88987	1.37577	-0.00511
N	-0.60074	2.31710	-0.75795
O	0.32402	3.24386	-0.26199
H	0.44201	3.84824	-1.00542
O	-4.62068	-2.78740	-0.07462
C	-6.00768	-2.53119	-0.17772
H	-6.49435	-3.50527	-0.16201
H	-6.36179	-1.93125	0.66844
H	-6.24519	-2.01836	-1.11683

p-OMe-oximyl-radical-Nu-attack-tBu-P-N-oxide

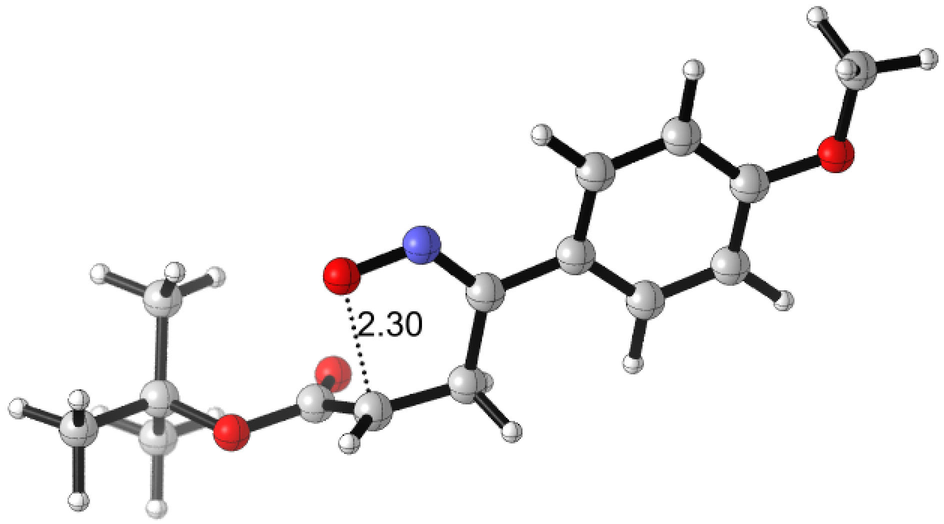

Sum of Electronic and Zero-point Energies = -937.970858 Hartree
Sum of Electronic and Thermal Energies = -937.950333 Hartree
Sum of Electronic and Thermal Enthalpies = -937.949389 Hartree
Sum of Electronic and Thermal Free Energies = -938.023604 Hartree

Dipole Moment = 4.7214 Debye

-1 2

C	1.87054	-0.43640	-1.14768
C	0.45740	-0.83835	-0.92851
C	-0.37585	0.27807	-0.29712
H	2.16891	0.09438	-2.04167
H	0.43542	-1.71299	-0.26292
H	-0.02433	-1.12876	-1.87478
C	2.87458	-0.85995	-0.24698
O	2.72113	-1.53873	0.77473
O	4.13133	-0.40096	-0.62159
C	5.14183	-0.23723	0.38120
N	0.17256	1.40046	0.04112
O	1.42567	1.59259	-0.17013
C	-1.81428	0.09741	-0.04918
C	-2.45704	-1.12506	-0.31585
C	-2.61197	1.13488	0.45985
C	-3.81811	-1.30042	-0.08650
H	-1.88279	-1.95960	-0.70562
C	-3.97448	0.97165	0.69521
H	-2.13558	2.08592	0.67410
C	-4.58627	-0.25479	0.42011
H	-4.30405	-2.24899	-0.29492
H	-4.54219	1.80650	1.09192

C	5.70216	-1.59167	0.82295
H	6.53901	-1.44077	1.51607
H	4.92191	-2.17712	1.31058
H	6.06831	-2.14331	-0.05002
C	6.22498	0.56475	-0.34009
H	5.82413	1.53324	-0.65364
H	7.08456	0.72918	0.31896
H	6.56009	0.02354	-1.23082
C	4.60369	0.56663	1.56849
H	5.43794	0.89067	2.20221
H	4.06599	1.44622	1.20016
H	3.90838	-0.03171	2.15929
O	-5.92345	-0.51843	0.61503
C	-6.71620	0.53063	1.11017
H	-6.37548	0.86065	2.10067
H	-6.71397	1.39222	0.42909
H	-7.73043	0.13642	1.19089

dimethyl-fumnerate

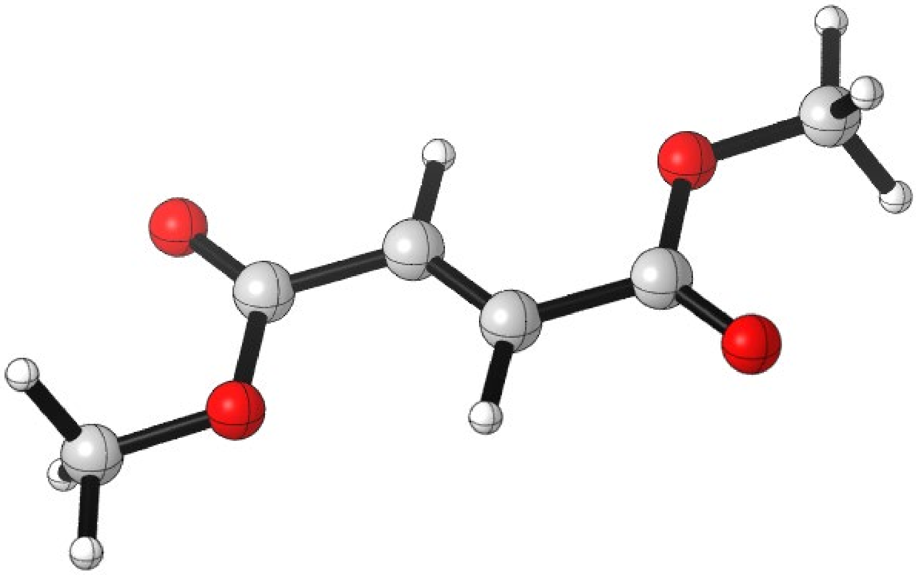

Sum of Electronic and Zero-point Energies = -534.007611 Hartree
Sum of Electronic and Thermal Energies = -533.996748 Hartree
Sum of Electronic and Thermal Enthalpies = -533.995804 Hartree
Sum of Electronic and Thermal Free Energies = -534.045247 Hartree

Dipole Moment = 0.0000 Debye

0 1

C	-0.62577	-1.51642	-0.00138
H	-0.08753	-2.45828	-0.00138

C	0.00817	-0.34290	-0.00138
H	-0.53007	0.59896	-0.00138
C	1.49031	-0.21534	-0.00138
C	-2.10792	-1.64399	-0.00138
O	-2.67937	-2.70946	-0.00138
O	2.06176	0.85013	-0.00138
O	2.12228	-1.39762	-0.00138
O	-2.73988	-0.46170	-0.00138
C	-4.16803	-0.53619	-0.00138
H	-4.51719	-1.06394	0.88829
H	-4.51544	0.49489	-0.00138
H	-4.51719	-1.06395	-0.89104
C	3.55043	-1.32314	-0.00138
H	3.89784	-2.35422	-0.00138
H	3.89959	-0.79537	-0.89104
H	3.89959	-0.79538	0.88829

dimethyl-maleate

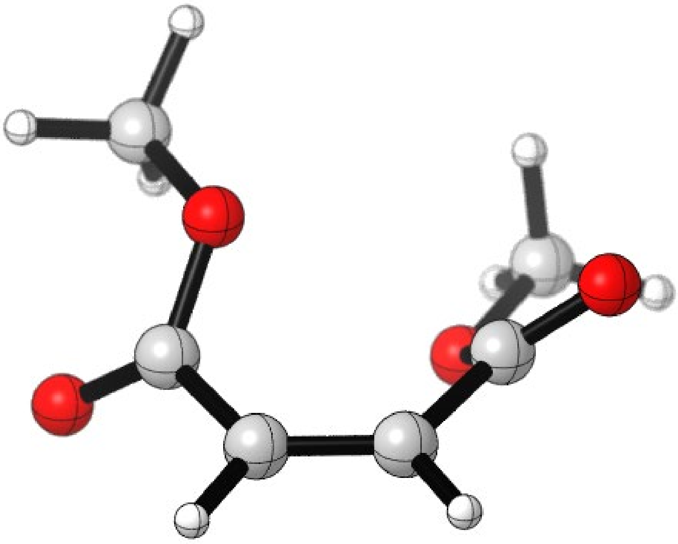

Sum of Electronic and Zero-point Energies = -533.999943 Hartree
Sum of Electronic and Thermal Energies = -533.989087 Hartree
Sum of Electronic and Thermal Enthalpies = -533.988143 Hartree
Sum of Electronic and Thermal Free Energies = -534.038289 Hartree

Dipole Moment = 1.3145 Debye

0 1

C	-0.48702	-1.58546	0.11385
C	0.17020	-0.44697	-0.10591
H	-0.36905	0.48742	-0.22658

C	1.65134	-0.32748	-0.24885
O	2.18407	0.36774	-1.08001
O	2.31558	-1.03913	0.67127
C	3.73802	-1.03160	0.53146
H	4.11806	-1.62483	1.36096
H	4.01970	-1.47551	-0.42653
H	4.11832	-0.00946	0.57820
H	-1.56566	-1.58524	0.23651
C	0.15034	-2.92811	0.25473
O	-0.17504	-3.73264	1.09420
O	1.08551	-3.15290	-0.67749
C	1.79135	-4.38825	-0.54114
H	2.33102	-4.40487	0.40895
H	1.09516	-5.22844	-0.57232
H	2.48250	-4.42616	-1.38093

Ph-oximyl-radical-cyclisation-P-FUMERATE

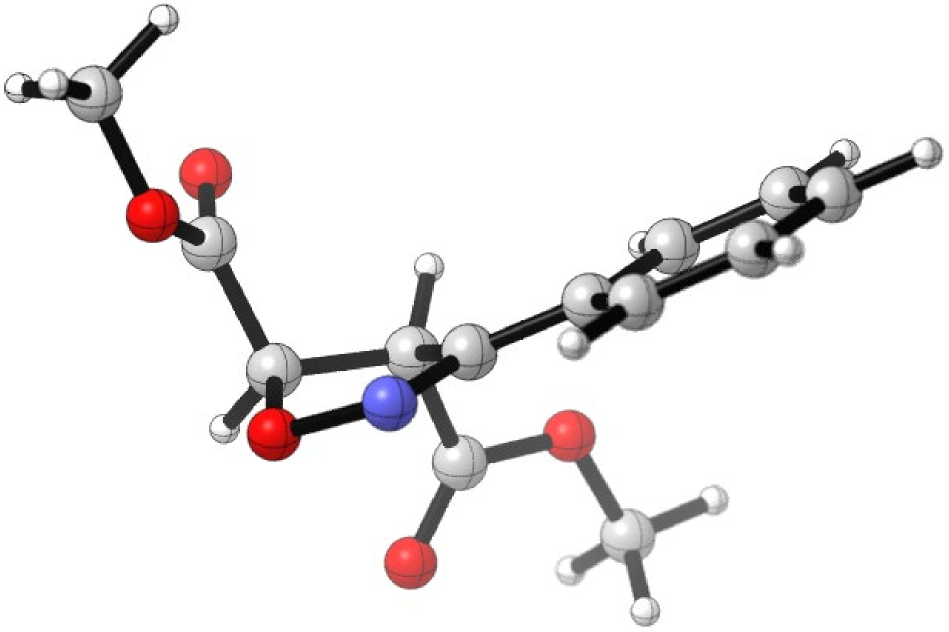

Sum of Electronic and Zero-point Energies = -933.471455 Hartree
Sum of Electronic and Thermal Energies = -933.453606 Hartree
Sum of Electronic and Thermal Enthalpies = -933.452662 Hartree
Sum of Electronic and Thermal Free Energies = -933.520287 Hartree

Dipole Moment = 4.7778 Debye

-1 2

C	-1.94115	0.31992	1.44447
C	-0.58841	-0.34844	1.69037
C	0.26378	0.51824	0.77118
H	-2.56685	0.33872	2.34402

H	-0.57689	-1.40966	1.42103
N	-0.34899	1.67558	0.46771
O	-1.64735	1.63905	1.07894
C	-2.70439	-0.45591	0.37128
O	-2.98998	-1.63080	0.47775
O	-3.05499	0.28617	-0.68148
C	-3.73449	-0.41751	-1.71363
H	-4.67124	-0.83979	-1.33983
H	-3.92660	0.31768	-2.49393
H	-3.10927	-1.22842	-2.09526
C	1.53447	0.15706	0.25434
C	2.18148	-1.08226	0.55133
C	2.22924	1.04003	-0.63711
C	3.40818	-1.40610	-0.00436
H	1.71035	-1.78025	1.23639
C	3.45529	0.69624	-1.17572
H	1.76283	1.98851	-0.88368
C	4.07626	-0.52921	-0.87599
H	3.86089	-2.36379	0.24743
H	3.94795	1.39610	-1.84913
H	5.04110	-0.78831	-1.30098
C	-0.14935	-0.19139	3.12753
O	0.88644	-1.01001	3.41613
O	-0.61707	0.57099	3.94531
C	1.47851	-0.81149	4.69255
H	2.30995	-1.51375	4.74608
H	1.83959	0.21558	4.78678
H	0.75501	-1.00664	5.48902

Ph-oximyl-radical-cyclisation-P-MALEATE

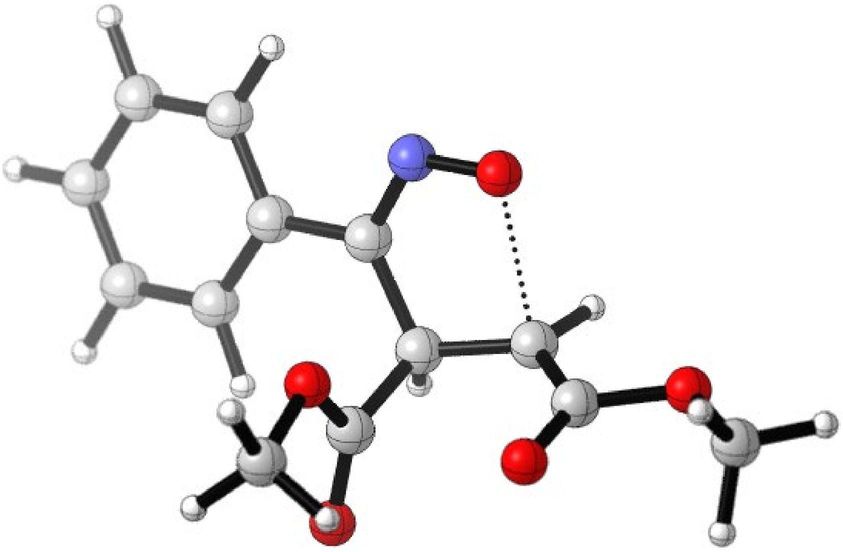

Sum of Electronic and Zero-point Energies = -933.465413 Hartree
Sum of Electronic and Thermal Energies = -933.447335 Hartree
Sum of Electronic and Thermal Enthalpies = -933.446391 Hartree
Sum of Electronic and Thermal Free Energies = -933.513634 Hartree

Dipole Moment = 3.3688 Debye

-1 2

C	-1.73402	0.01872	0.69485
C	-0.36896	-0.59586	0.71225
C	0.62668	0.27315	-0.05659
H	-1.87574	0.92854	1.26380
H	-0.02289	-0.66398	1.75293
N	0.18674	1.17061	-0.88458
O	-1.07193	1.32813	-1.03543
C	-2.89310	-0.64405	0.22622
O	-2.98152	-1.74609	-0.32503
O	-4.03522	0.10928	0.43752
C	-5.21770	-0.46615	-0.07572
H	-5.41966	-1.43856	0.38556
H	-6.02065	0.23611	0.15852
H	-5.14853	-0.61066	-1.15863
C	2.07379	0.09569	0.10399
C	2.59811	-0.95842	0.87277
C	2.99077	0.97490	-0.50849
C	3.97539	-1.12302	1.02526
H	1.92992	-1.66956	1.34992
C	4.35924	0.80503	-0.35485
H	2.59535	1.78980	-1.10556
C	4.86805	-0.24573	0.41663
H	4.34746	-1.95070	1.62351

H	5.04064	1.50032	-0.83906
H	5.93985	-0.37530	0.53624
C	-0.37713	-2.03451	0.21193
O	-0.29648	-2.11024	-1.12141
O	-0.40990	-3.00782	0.93431
C	-0.59809	-3.38875	-1.66086
H	0.10562	-4.14137	-1.29453
H	-1.61780	-3.66039	-1.37413
H	-0.51641	-3.28113	-2.74240

Ph-oximyl-radical-cyclisation-tBu-P-N-oxide

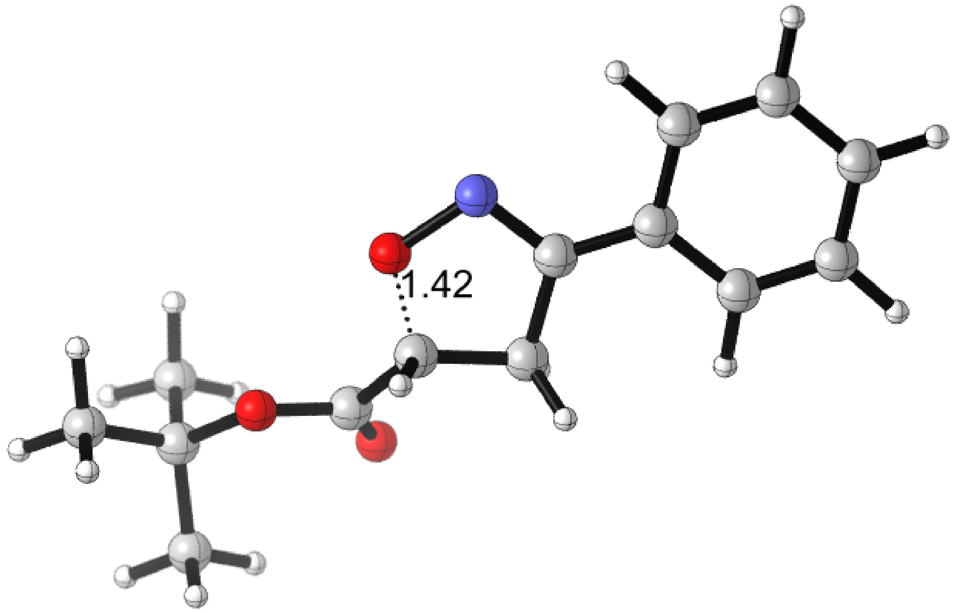

Sum of Electronic and Zero-point Energies = -823.525180 Hartree
Sum of Electronic and Thermal Energies = -823.507740 Hartree
Sum of Electronic and Thermal Enthalpies = -823.506796 Hartree
Sum of Electronic and Thermal Free Energies = -823.572553 Hartree

Dipole Moment = 12.4469 Debye

-1 2

C	1.59036	-0.41080	-0.65161
C	0.35412	-1.00360	-0.00142
C	-0.59880	0.15682	-0.19132
H	1.63720	-0.66216	-1.72444
H	0.53438	-1.23508	1.05686
H	0.02439	-1.92011	-0.50207
N	0.04145	1.29776	-0.48472
O	1.45045	0.99560	-0.49947
C	2.91303	-0.77511	-0.00242

O	3.05135	-1.36705	1.04395
O	3.93454	-0.32151	-0.75234
C	5.29817	-0.37276	-0.26540
C	-2.00213	0.05686	-0.06823
C	-2.65566	-1.17209	0.27654
C	-2.85783	1.19373	-0.27785
C	-4.03219	-1.25267	0.38864
H	-2.05678	-2.06050	0.46464
C	-4.22902	1.08785	-0.16024
H	-2.39570	2.14218	-0.53431
C	-4.85558	-0.13102	0.17258
H	-4.48302	-2.20782	0.65450
H	-4.84055	1.97342	-0.32931
H	-5.93491	-0.20094	0.26523
C	6.08256	0.31949	-1.37624
H	7.14750	0.34540	-1.12537
H	5.95383	-0.21732	-2.32057
H	5.72120	1.34278	-1.50871
C	5.42240	0.41257	1.03958
H	4.98739	1.40830	0.91263
H	4.90889	-0.09413	1.85731
H	6.48164	0.52214	1.29539
C	5.75820	-1.82203	-0.11178
H	5.59662	-2.36526	-1.04833
H	6.82934	-1.83933	0.11647
H	5.21397	-2.32320	0.68926

Ph-oximyl-radical-cyclisation-tBu-P

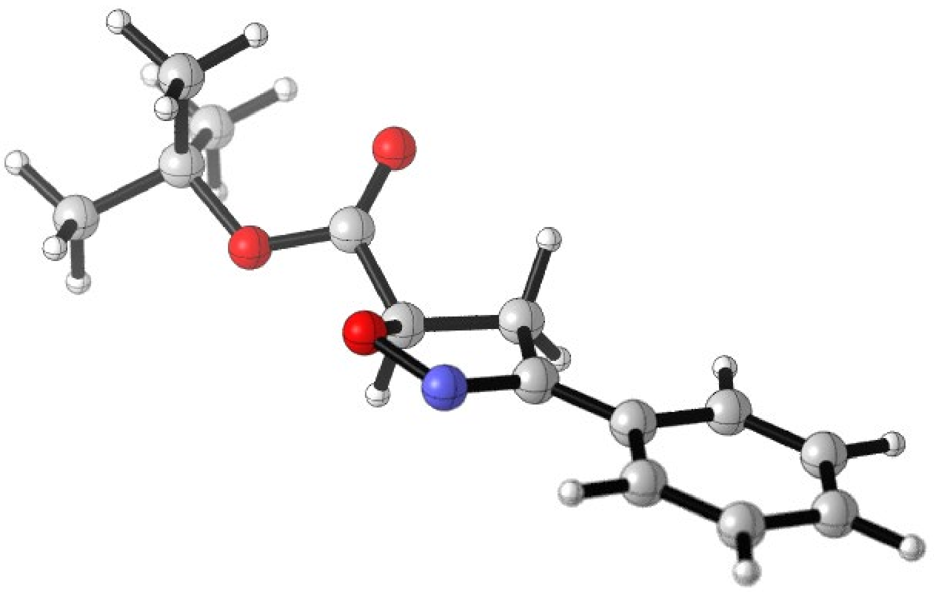

Sum of Electronic and Zero-point Energies = -823.525091 Hartree
Sum of Electronic and Thermal Energies = -823.507670 Hartree
Sum of Electronic and Thermal Enthalpies = -823.506726 Hartree
Sum of Electronic and Thermal Free Energies = -823.572302 Hartree

Dipole Moment = 7.2678 Debye

-1 2

C	1.59074	-0.41864	-0.65705
C	0.35383	-1.00764	-0.00447
C	-0.59771	0.15335	-0.19792
H	1.63743	-0.67477	-1.72883
H	0.53421	-1.23601	1.05445
H	0.02280	-1.92540	-0.50197
N	0.04366	1.29223	-0.49684
O	1.45223	0.98854	-0.51106
C	2.91290	-0.78203	-0.00641
O	3.05042	-1.37865	1.03746
O	3.93486	-0.32220	-0.75178
C	5.29735	-0.37118	-0.26132
C	-2.00102	0.05613	-0.07243
C	-2.65622	-1.17018	0.27829
C	-2.85497	1.19366	-0.28560
C	-4.03271	-1.24782	0.39269
H	-2.05855	-2.05881	0.46925
C	-4.22617	1.09073	-0.16574
H	-2.39137	2.14015	-0.54664
C	-4.85442	-0.12561	0.17305
H	-4.48497	-2.20101	0.66317
H	-4.83642	1.97661	-0.33784
H	-5.93372	-0.19324	0.26762
C	5.41514	0.40841	1.04765
H	4.89898	-0.10296	1.86080
H	6.47314	0.51828	1.30837
H	4.97909	1.40404	0.92349
C	5.76190	-1.81958	-0.11323
H	5.60351	-2.35925	-1.05238
H	6.83279	-1.83430	0.11644
H	5.21836	-2.32619	0.68482
C	6.08254	0.32889	-1.36671
H	7.14647	0.35841	-1.11192
H	5.95952	-0.20464	-2.31367
H	5.71717	1.35110	-1.49630

Ph-oximyl-radical-cyclisation-tBu-P_regioisomer

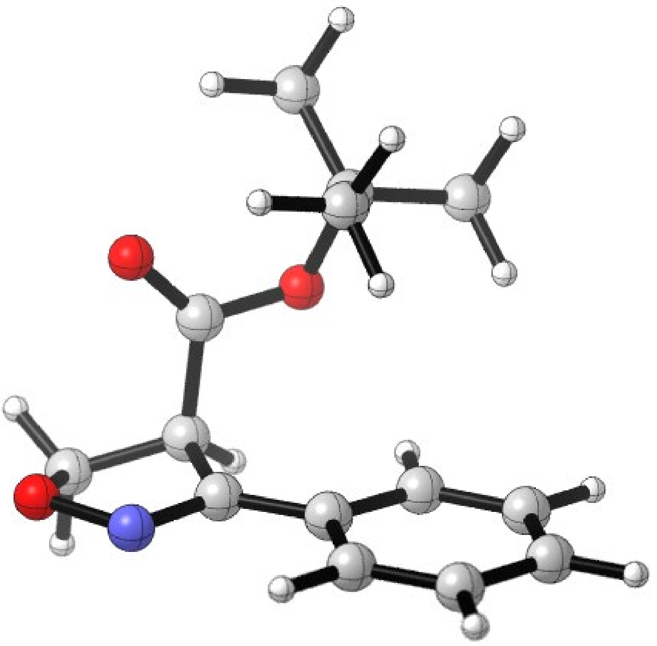

Sum of Electronic and Zero-point Energies = -823.530875 Hartree
Sum of Electronic and Thermal Energies = -823.513813 Hartree
Sum of Electronic and Thermal Enthalpies = -823.512868 Hartree
Sum of Electronic and Thermal Free Energies = -823.576220 Hartree

Dipole Moment = 7.8851 Debye

-1 2

C	-1.15382	-3.07676	-0.01588
C	-0.82725	-1.75773	-0.28446
C	-1.02495	-0.72632	0.68676
C	-1.59705	-1.13694	1.93798
C	-1.91061	-2.46040	2.18462
C	-1.69426	-3.46498	1.22298
H	-0.98585	-3.82594	-0.78882
H	-0.40064	-1.50501	-1.25030
H	-1.77123	-0.37708	2.69311
H	-2.33482	-2.72762	3.15145
H	-1.93860	-4.50244	1.42867
C	-0.71155	0.63908	0.45545
N	-0.98426	1.61033	1.33725
O	-0.55634	2.84465	0.77043
C	1.46473	0.87757	-0.62694
O	2.28036	1.59774	-0.09059
O	1.75268	-0.34797	-1.12347
C	2.87701	-1.10523	-0.60883
C	2.74105	-1.23566	0.90719
H	3.51068	-1.91851	1.28329
H	2.85809	-0.26658	1.39694
H	1.75291	-1.63947	1.15344
C	2.70977	-2.46871	-1.27393

H	2.73728	-2.36369	-2.36344
H	3.51886	-3.13801	-0.96377
H	1.75062	-2.90674	-0.98325
C	4.20201	-0.46644	-1.02512
H	5.02637	-1.13723	-0.75781
H	4.21780	-0.31566	-2.10987
H	4.34310	0.49566	-0.53222
C	-0.00687	1.18561	-0.78303
H	-0.38328	0.75342	-1.71703
C	-0.33049	2.66288	-0.61682
H	-1.24315	2.90617	-1.18399
H	0.48773	3.32480	-0.91488

Ph-oximyl-radical-cyclisation-tBu-P_triplet

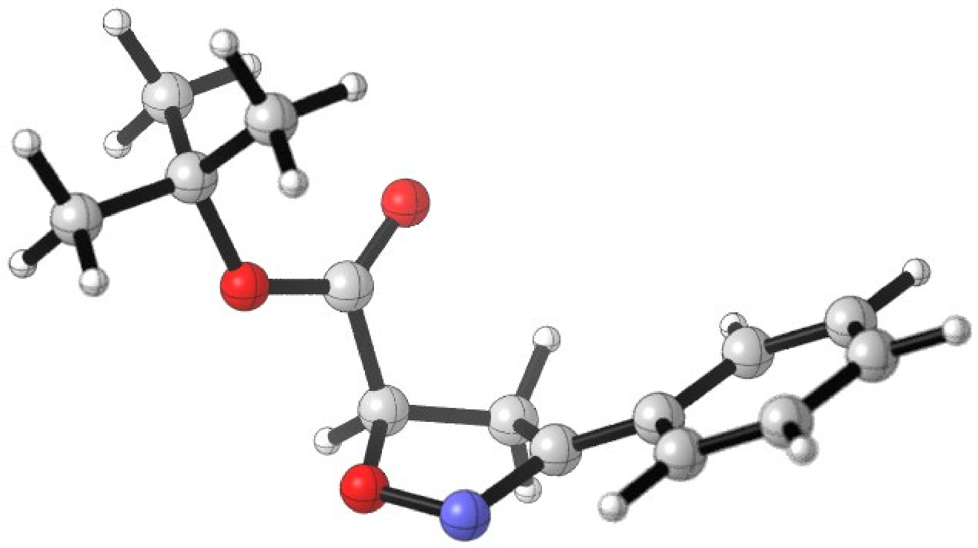

Sum of Electronic and Zero-point Energies = -823.423600 Hartree
Sum of Electronic and Thermal Energies = -823.406283 Hartree
Sum of Electronic and Thermal Enthalpies = -823.405338 Hartree
Sum of Electronic and Thermal Free Energies = -823.472213 Hartree

Dipole Moment = 2.3632 Debye

0 3

C	-1.85465	-0.08998	1.67631
C	-0.44517	-0.61404	1.91782
C	0.31223	0.33377	1.05129
H	-2.52274	-0.12639	2.54131
H	-0.34965	-1.65625	1.61315
H	-0.17982	-0.51079	2.97818

N	-0.39587	1.60339	1.01867
O	-1.66560	1.31299	1.37535
C	-2.54149	-0.78901	0.49567
O	-2.18756	-1.86414	0.07099
O	-3.56571	-0.06735	0.06285
C	-4.36177	-0.49262	-1.09298
C	1.43863	0.14019	0.26878
C	2.14534	-1.10801	0.25321
C	1.94840	1.20567	-0.54706
C	3.26445	-1.27118	-0.53269
H	1.78833	-1.92961	0.86590
C	3.06939	1.01251	-1.33065
H	1.43744	2.16163	-0.53055
C	3.73975	-0.21822	-1.33512
H	3.78418	-2.22433	-0.53297
H	3.43861	1.82866	-1.94409
H	4.62347	-0.35761	-1.94836
C	-5.06620	-1.80951	-0.78099
H	-5.76580	-2.03831	-1.59048
H	-4.35497	-2.63123	-0.68957
H	-5.63753	-1.72033	0.14797
C	-3.46619	-0.58715	-2.32462
H	-4.09229	-0.74925	-3.20701
H	-2.91618	0.34858	-2.46126
H	-2.75692	-1.41162	-2.24268
C	-5.36863	0.64102	-1.24088
H	-6.02959	0.44042	-2.08844
H	-5.97551	0.73342	-0.33614
H	-4.85170	1.58876	-1.41196

Ph-oximyl-radical-cyclisation-tBu-TS

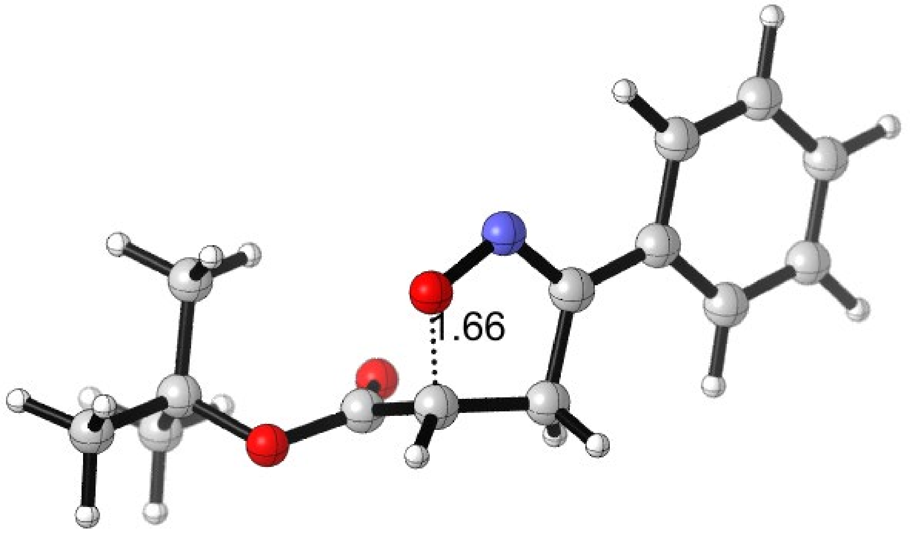

Sum of Electronic and Zero-point Energies = -823.509068 Hartree
Sum of Electronic and Thermal Energies = -823.491926 Hartree
Sum of Electronic and Thermal Enthalpies = -823.490982 Hartree
Sum of Electronic and Thermal Free Energies = -823.555963 Hartree

Dipole Moment = 4.6773 Debye

-1 2

C	-1.87493	0.11785	1.10492
C	-0.45365	-0.31109	1.41776
C	0.37249	0.42292	0.38901
H	-2.46471	0.53199	1.92085
H	-0.35340	-1.39416	1.29754
H	-0.16649	-0.03393	2.44176
N	-0.27934	1.37118	-0.22456
O	-1.51275	1.51415	0.29156
C	-2.62475	-0.77350	0.24305
O	-2.14101	-1.57236	-0.57978
O	-4.00289	-0.55021	0.33124
C	-4.77476	-0.58751	-0.87672
C	1.74222	0.09729	0.03757
C	2.42041	-0.97640	0.64893
C	2.45973	0.87326	-0.90273
C	3.74540	-1.26439	0.33492
H	1.89820	-1.59181	1.37554
C	3.77841	0.57692	-1.21365
H	1.94857	1.70507	-1.37649
C	4.44049	-0.49358	-0.59910
H	4.23889	-2.10146	0.82282
H	4.30346	1.18755	-1.94458
H	5.47437	-0.71896	-0.84355

C	-5.00048	-2.03170	-1.33439
H	-5.67635	-2.05113	-2.19845
H	-4.04706	-2.49071	-1.59841
H	-5.45623	-2.60778	-0.52153
C	-4.10082	0.24654	-1.96882
H	-4.77377	0.34652	-2.82850
H	-3.86241	1.24119	-1.57901
H	-3.16937	-0.22314	-2.29193
C	-6.10620	0.04688	-0.47639
H	-6.80361	0.04297	-1.32140
H	-6.55192	-0.51289	0.35226
H	-5.94629	1.07910	-0.15056

Ph-oximyl-radical-cyclisation-tBu-TS_regioisomer

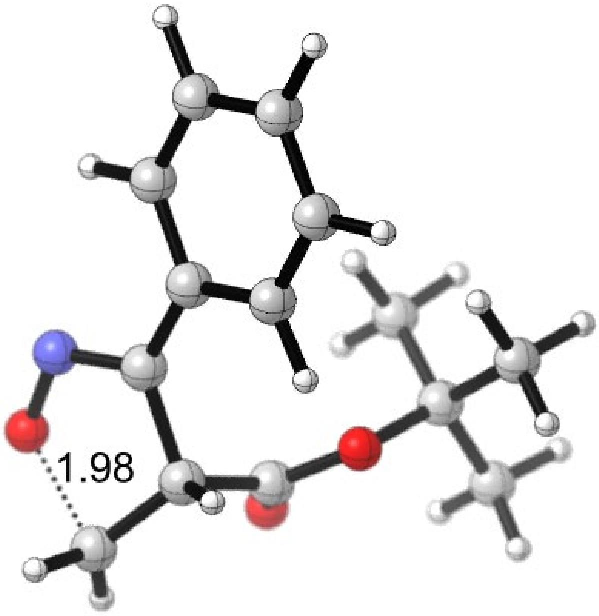

Sum of Electronic and Zero-point Energies = -823.483034 Hartree
Sum of Electronic and Thermal Energies = -823.465845 Hartree
Sum of Electronic and Thermal Enthalpies = -823.464901 Hartree
Sum of Electronic and Thermal Free Energies = -823.529111 Hartree

Dipole Moment = 8.7290 Debye

-1 2

C	-2.27948	-2.01041	-1.30814
C	-1.44178	-0.90205	-1.23124
C	-1.63326	0.10358	-0.25329
C	-2.71871	-0.08455	0.64487
C	-3.55018	-1.19043	0.55506
C	-3.34571	-2.17485	-0.42063

H	-2.09190	-2.76049	-2.07408
H	-0.61005	-0.82484	-1.92344
H	-2.87940	0.66856	1.40926
H	-4.37083	-1.29391	1.26207
H	-3.99348	-3.04439	-0.48109
C	-0.77748	1.26052	-0.12363
N	-1.14743	2.29227	0.61984
O	-0.26648	3.24118	0.67926
C	1.60240	0.74467	-0.28121
O	2.41408	1.18807	0.49995
O	1.53810	-0.56943	-0.62544
C	2.07325	-1.58726	0.25109
C	1.44308	-1.44260	1.63556
H	1.72913	-2.29638	2.25950
H	1.77040	-0.52141	2.12168
H	0.35207	-1.42192	1.53928
C	1.61197	-2.88640	-0.40487
H	2.01243	-2.95711	-1.42144
H	1.96481	-3.74653	0.17370
H	0.51955	-2.91009	-0.45646
C	3.60027	-1.52670	0.29719
H	3.98207	-2.39850	0.84082
H	4.00276	-1.55099	-0.72102
H	3.93936	-0.61467	0.78875
C	0.49880	1.51524	-0.96339
H	0.33471	1.08220	-1.95658
C	0.73725	2.98257	-1.01116
H	0.14926	3.56888	-1.70807
H	1.68562	3.38221	-0.67790

Ph-oximyl-radical-cyclisation-tBu-TS_triplet

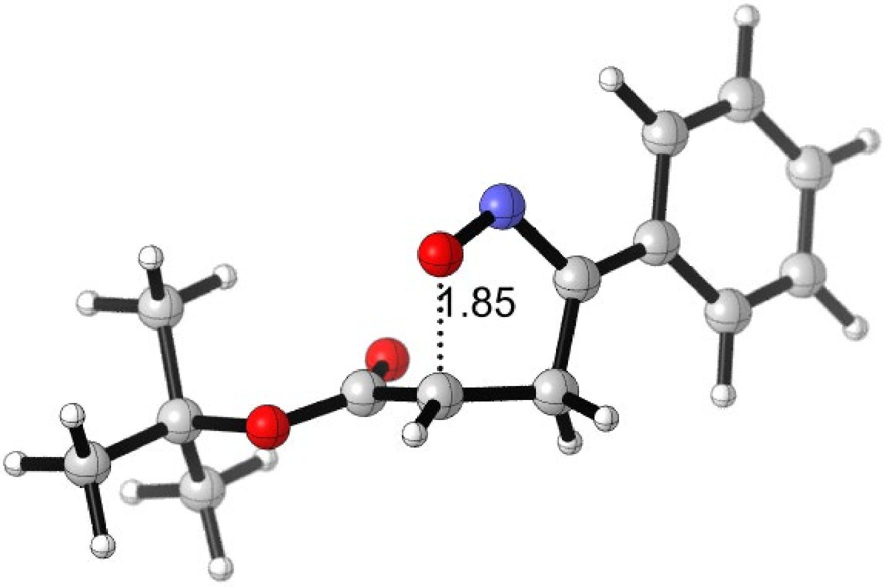

Sum of Electronic and Zero-point Energies = -823.397053 Hartree
Sum of Electronic and Thermal Energies = -823.379935 Hartree
Sum of Electronic and Thermal Enthalpies = -823.378991 Hartree
Sum of Electronic and Thermal Free Energies = -823.444174 Hartree

Dipole Moment = 2.4975 Debye

0 3

C	-1.87988	0.22713	1.32079
C	-0.48857	-0.24091	1.61115
C	0.40438	0.46752	0.63347
H	-2.52713	0.56111	2.12264
H	-0.39882	-1.31822	1.44369
H	-0.21638	-0.01991	2.64984
N	-0.18774	1.62960	0.09616
O	-1.27589	1.85670	0.69114
C	-2.52511	-0.32808	0.12786
O	-1.89122	-0.82400	-0.79016
O	-3.86015	-0.16742	0.15809
C	-4.68004	-0.53355	-0.98994
C	1.67507	0.05954	0.16709
C	2.28049	-1.14101	0.62286
C	2.38497	0.86128	-0.76757
C	3.52518	-1.52193	0.15612
H	1.76099	-1.76850	1.33971
C	3.63017	0.46431	-1.22594
H	1.93578	1.78672	-1.10912
C	4.20830	-0.72470	-0.77124
H	3.97315	-2.44462	0.51068
H	4.15995	1.08425	-1.94206
H	5.18534	-1.02853	-1.13240

C	-4.58689	-2.03604	-1.24661
H	-5.31782	-2.31497	-2.01190
H	-3.59102	-2.31801	-1.58968
H	-4.82259	-2.58764	-0.33120
C	-4.26818	0.28815	-2.20945
H	-4.98923	0.11907	-3.01516
H	-4.27308	1.35376	-1.96101
H	-3.27435	0.00858	-2.56043
C	-6.08580	-0.15395	-0.53777
H	-6.80637	-0.38563	-1.32700
H	-6.35974	-0.71089	0.36263
H	-6.13831	0.91546	-0.31611

Ph-oximyl-radical-cyclisation-TS-FUMERATE-ii

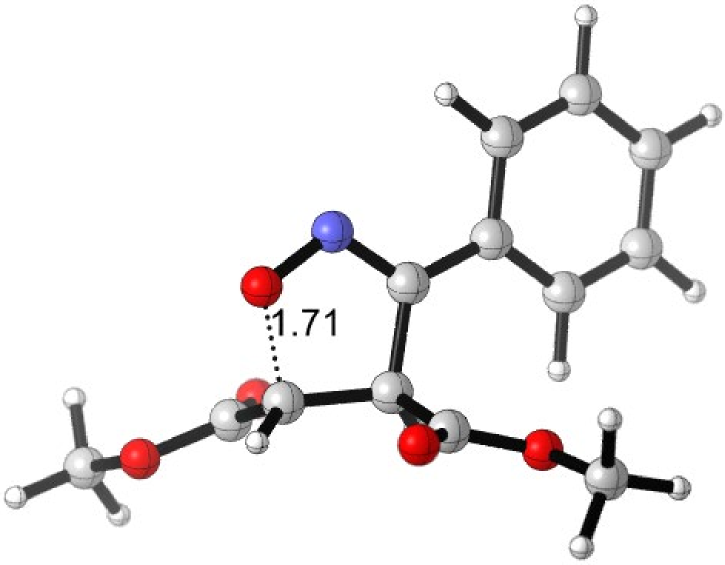

Sum of Electronic and Zero-point Energies = -933.452947 Hartree
Sum of Electronic and Thermal Energies = -933.435239 Hartree
Sum of Electronic and Thermal Enthalpies = -933.434295 Hartree
Sum of Electronic and Thermal Free Energies = -933.501196 Hartree

Dipole Moment = 4.1241 Debye

-1 2

C	-1.87299	0.32280	1.06298
C	-0.51744	-0.25011	1.41039
C	0.46826	0.56222	0.57066
H	-2.48667	0.67611	1.88579
H	-0.45783	-1.30652	1.12905
N	-0.07084	1.63712	0.05439

O	-1.31307	1.82585	0.48451
C	-2.58758	-0.36995	0.01654
O	-2.09959	-1.06869	-0.88021
O	-3.94716	-0.08897	0.03807
C	-4.66721	-0.64209	-1.04140
H	-4.60049	-1.73591	-1.04567
H	-5.70523	-0.33016	-0.90605
H	-4.28426	-0.27643	-1.99971
C	1.81224	0.14647	0.21088
C	2.32296	-1.12750	0.53165
C	2.66609	1.03497	-0.48417
C	3.61627	-1.49442	0.17214
H	1.70242	-1.83235	1.07408
C	3.95349	0.65952	-0.84023
H	2.28486	2.01911	-0.73607
C	4.44801	-0.60866	-0.51583
H	3.97719	-2.48674	0.43170
H	4.58390	1.36467	-1.37699
H	5.45783	-0.89746	-0.79234
C	-0.18417	-0.09400	2.87317
O	0.79458	-0.95535	3.24717
O	-0.67218	0.69223	3.65293
C	1.27493	-0.78436	4.57332
H	2.06494	-1.52415	4.70143
H	1.67162	0.22523	4.70769
H	0.47300	-0.94636	5.29867

Ph-oximyl-radical-cyclisation-TS-MALEATE-ii

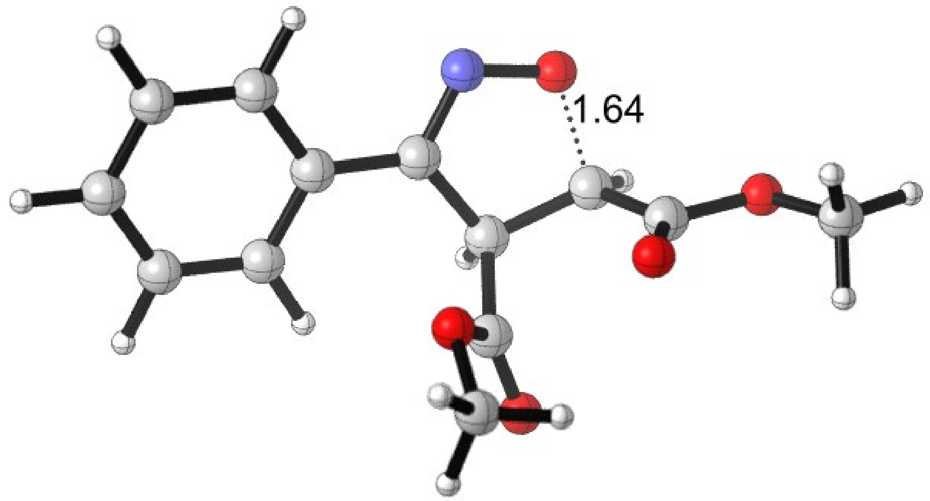

Sum of Electronic and Zero-point Energies = -933.446684 Hartree
Sum of Electronic and Thermal Energies = -933.429057 Hartree
Sum of Electronic and Thermal Enthalpies = -933.428112 Hartree
Sum of Electronic and Thermal Free Energies = -933.494172 Hartree

Dipole Moment = 2.7787 Debye

-1 2

C	-1.79018	0.17168	0.89115
C	-0.42481	-0.51446	1.09692
C	0.54819	0.36284	0.33914
H	-2.26280	0.51778	1.80890
H	-0.20672	-0.54319	2.17294
N	0.00909	1.45659	-0.12453
O	-1.25059	1.60460	0.30649
C	-2.75040	-0.44294	-0.00338
O	-2.53135	-1.19879	-0.96725
O	-4.04221	0.00503	0.26767
C	-5.01755	-0.48267	-0.62443
H	-5.07480	-1.57684	-0.59275
H	-5.96780	-0.04874	-0.30420
H	-4.79593	-0.18555	-1.65506
C	1.95364	0.06739	0.12183
C	2.57702	-1.04591	0.72154
C	2.76490	0.93466	-0.64542
C	3.93622	-1.28820	0.55205
H	1.99195	-1.72989	1.33068
C	4.11845	0.68283	-0.81487
H	2.29935	1.80174	-1.10249
C	4.72366	-0.43051	-0.21930
H	4.38469	-2.15654	1.02828
H	4.71483	1.36332	-1.41816
H	5.78471	-0.62102	-0.35060
C	-0.47903	-1.96780	0.64847
O	0.05577	-2.17400	-0.55702
O	-0.94863	-2.85436	1.33032
C	-0.26431	-3.42445	-1.14912
H	0.06709	-4.25206	-0.51605
H	-1.34682	-3.47299	-1.29647
H	0.25629	-3.43955	-2.10627

Ph-oximyl-radical-cyclisationSTYRENE-TS-cyclopropanation

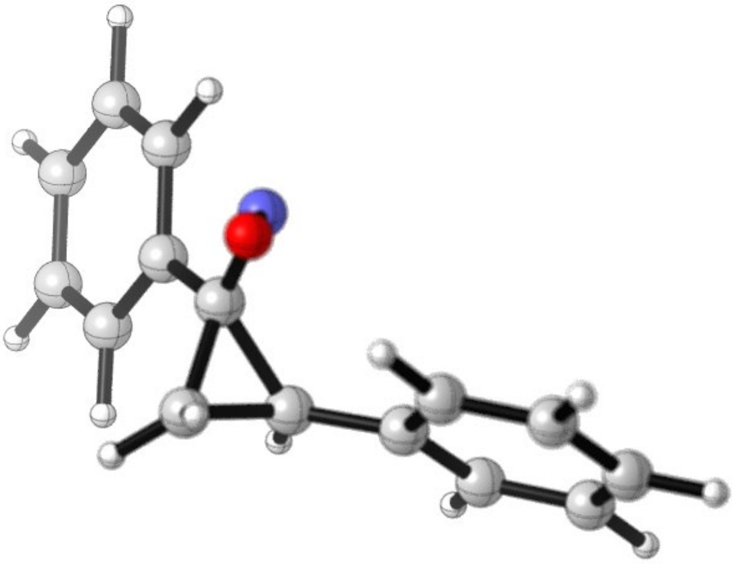

```
Sum of electronic and zero-point Energies=           -708.823083
Sum of electronic and thermal Energies=              -708.809422
Sum of electronic and thermal Enthalpies=            -708.808478
Sum of electronic and thermal Free Energies=         -708.865010
Dipole Moment = 6.8850 Debye

-1 2
 C                  -3.80521900   -1.37000100    0.53460600
 C                  -2.54319000   -0.87116400    0.85251200
 C                  -1.93113000    0.12129700    0.07162200
 C                  -2.63321800    0.59715100   -1.04629900
 C                  -3.89203300    0.09372000   -1.36812100
 C                  -4.48969100   -0.89201000   -0.58209200
 H                  -4.25518000   -2.13340400    1.16471300
 H                  -2.03171100   -1.26151000    1.72910700
 H                  -2.15888000    1.37249700   -1.63755700
 H                  -4.41380300    0.47945000   -2.24073500
 H                  -5.47357400   -1.27928900   -0.83290100
 C                  -0.57516500    0.67633800    0.38904200
 N                  -0.20476100    1.80943700   -0.45406600
 O                   0.42422300    2.74102700    0.20788200
 C                  -0.03738500    0.57080300    1.77627800
 C                   0.47263700   -0.42365000    0.76857400
 H                   0.03761500   -1.42008900    0.84111300
 H                   0.61854400    1.40412100    2.01835000
 H                  -0.66798300    0.20652900    2.58426200
 C                   1.86089300   -0.45585800    0.25572900
 C                   2.42378100   -1.69796500   -0.07742800
 C                   2.64941400    0.69437100    0.08066200
 C                   3.72898800   -1.80415600   -0.55245100
 H                   1.82196400   -2.59657400    0.04237600
 C                   3.95428800    0.58397600   -0.39594500
 H                   2.21812800    1.66970900    0.29336900
 C                   4.50676600   -0.65789500   -0.71249300
 H                   4.13637400   -2.78190200   -0.79788000
 H                   4.54420800    1.48723500   -0.52985000
```

H 5.52542500 -0.73079500 -1.08412300

Ph-oximyl-radical-Nu-attack-P-FUMERATE

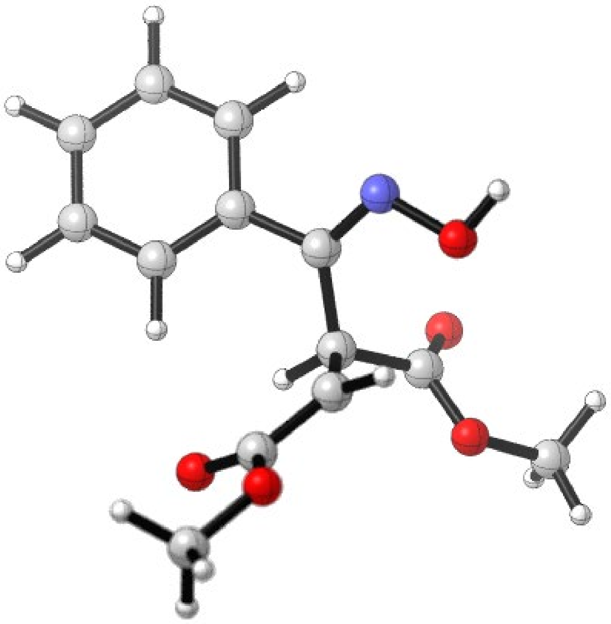

Sum of Electronic and Zero-point Energies = -934.000682 Hartree
Sum of Electronic and Thermal Energies = -933.981921 Hartree
Sum of Electronic and Thermal Enthalpies = -933.980977 Hartree
Sum of Electronic and Thermal Free Energies = -934.049945 Hartree

Dipole Moment = 2.6341 Debye

0 2

C	3.86804	-0.76635	-0.32232
C	2.99039	0.31007	-0.27554
C	1.64103	0.10731	0.04368
C	1.18706	-1.18776	0.31903
C	2.07160	-2.26401	0.26467
C	3.41129	-2.05773	-0.05326
H	4.91339	-0.59762	-0.56109
H	3.33818	1.31797	-0.47430
H	0.14290	-1.37035	0.56031
H	1.70772	-3.26490	0.47371
H	4.09911	-2.89681	-0.08664
C	0.70631	1.25789	0.06820
N	0.98904	2.26424	-0.66832
O	0.03256	3.26528	-0.56132
H	0.39573	3.98464	-1.09054
C	-0.55849	1.20864	0.92142
C	-1.76691	1.04796	0.06479
H	-2.07019	1.83707	-0.61034

H	-0.49177	0.34459	1.59047
C	-2.52004	-0.19123	0.11124
O	-2.23706	-1.14966	0.81318
O	-3.58011	-0.17608	-0.71805
C	-4.36166	-1.37104	-0.72486
H	-5.16789	-1.19043	-1.43322
H	-3.75222	-2.22059	-1.04072
H	-4.75950	-1.56969	0.27265
C	-0.60080	2.42726	1.84573
O	0.35482	2.84328	2.44805
O	-1.83724	2.92628	1.96067
C	-1.95553	4.04929	2.83892
H	-1.32605	4.86750	2.48332
H	-3.00665	4.32992	2.81675
H	-1.64800	3.77226	3.84904

Ph-oximyl-radical-Nu-attack-P-MALEATE

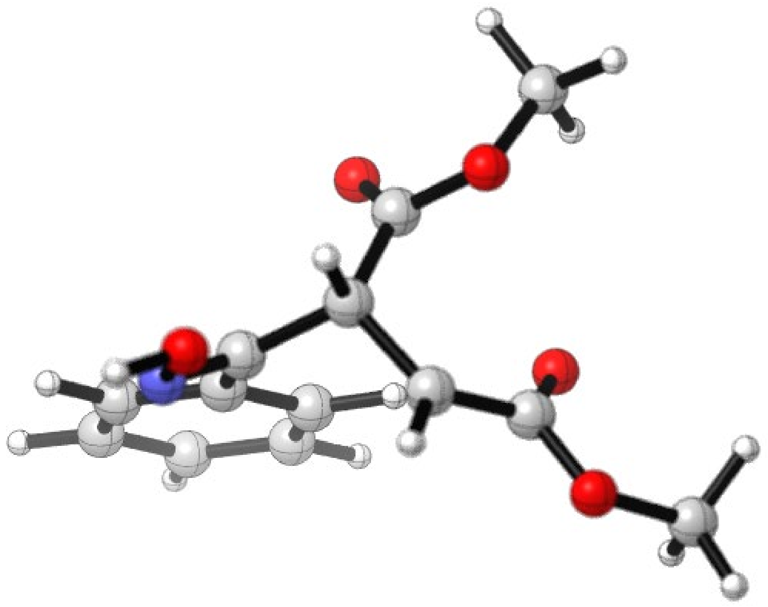

Sum of Electronic and Zero-point Energies = -933.994774 Hartree
Sum of Electronic and Thermal Energies = -933.976046 Hartree
Sum of Electronic and Thermal Enthalpies = -933.975102 Hartree
Sum of Electronic and Thermal Free Energies = -934.044054 Hartree

Dipole Moment = 2.8156 Debye

0 2

C	4.52618	0.73967	0.84929
C	3.33116	1.43912	0.97273
C	2.10460	0.77765	0.82860

C	2.09618	-0.59632	0.57132
C	3.29627	-1.29155	0.44173
C	4.51328	-0.62886	0.57891
H	5.46968	1.26260	0.97054
H	3.33610	2.50217	1.18663
H	1.16092	-1.13910	0.48753
H	3.27437	-2.35823	0.24272
H	5.44646	-1.17567	0.48570
C	0.84060	1.55588	0.90653
N	0.92899	2.79975	0.61160
O	-0.29105	3.46260	0.65322
H	-0.05451	4.37295	0.44173
C	-0.51847	0.92877	1.21838
C	-1.16979	0.46290	-0.04525
H	-1.48616	1.21381	-0.75955
H	-1.14037	1.75350	1.58968
C	-1.36184	-0.93660	-0.38554
O	-0.94853	-1.88424	0.26159
O	-2.05650	-1.07670	-1.53050
C	-2.27725	-2.42566	-1.94407
H	-2.84412	-2.35926	-2.87063
H	-1.32368	-2.93195	-2.10883
H	-2.84189	-2.96767	-1.18240
C	-0.53228	-0.08481	2.35507
O	0.40846	-0.42338	3.02397
O	-1.79472	-0.47904	2.57646
C	-1.93831	-1.52462	3.54038
H	-1.53726	-1.21037	4.50573
H	-3.00726	-1.71729	3.60882
H	-1.40602	-2.41308	3.19307

Ph-oximyl-radical-Nu-attack-P-N-oxide-FUMERATE

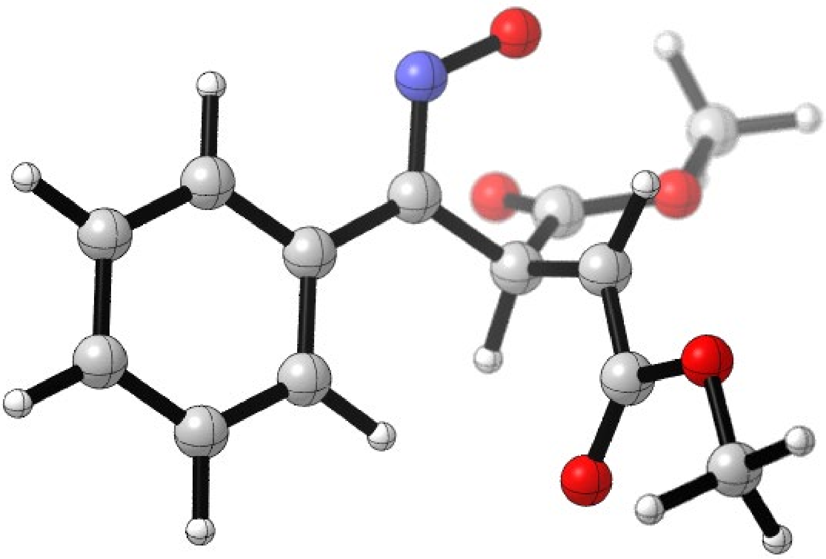

Sum of Electronic and Zero-point Energies = -933.466268 Hartree
Sum of Electronic and Thermal Energies = -933.447914 Hartree
Sum of Electronic and Thermal Enthalpies = -933.446970 Hartree
Sum of Electronic and Thermal Free Energies = -933.514593 Hartree

Dipole Moment = 4.4611 Debye

-1 2

C	4.23464	-0.01124	0.53599
C	3.00433	-0.62917	0.71410
C	1.85295	-0.16964	0.03931
C	1.99717	0.93925	-0.81499
C	3.23685	1.55395	-0.98695
C	4.36665	1.08731	-0.31903
H	5.10389	-0.39343	1.06592
H	2.90292	-1.48475	1.37243
H	1.13175	1.35237	-1.32426
H	3.31164	2.41315	-1.64869
H	5.33099	1.56779	-0.45843
C	0.56739	-0.84584	0.23286
N	0.45848	-1.73865	1.21104
O	-0.69515	-2.25834	1.37583
C	-0.66488	-0.51935	-0.59295
C	-1.33455	0.46927	0.27744
H	-1.72228	0.13956	1.23310
H	-0.38225	-0.06462	-1.54700
C	-1.36706	1.85031	-0.06897
O	-0.95735	2.36296	-1.11194
O	-1.94538	2.62641	0.90655
C	-2.00823	4.00699	0.60218
H	-2.48390	4.48181	1.46166

H	-1.00690	4.41943	0.44637
H	-2.59694	4.18357	-0.30336
C	-1.44358	-1.78037	-0.92520
O	-0.98153	-2.71860	-1.53347
O	-2.75281	-1.69586	-0.60554
C	-3.46451	-2.91347	-0.75682
H	-3.00005	-3.68896	-0.14089
H	-4.48085	-2.71090	-0.41718
H	-3.46682	-3.23977	-1.80077

Ph-oximyl-radical-Nu-attack-P-N-oxide-MALEATE

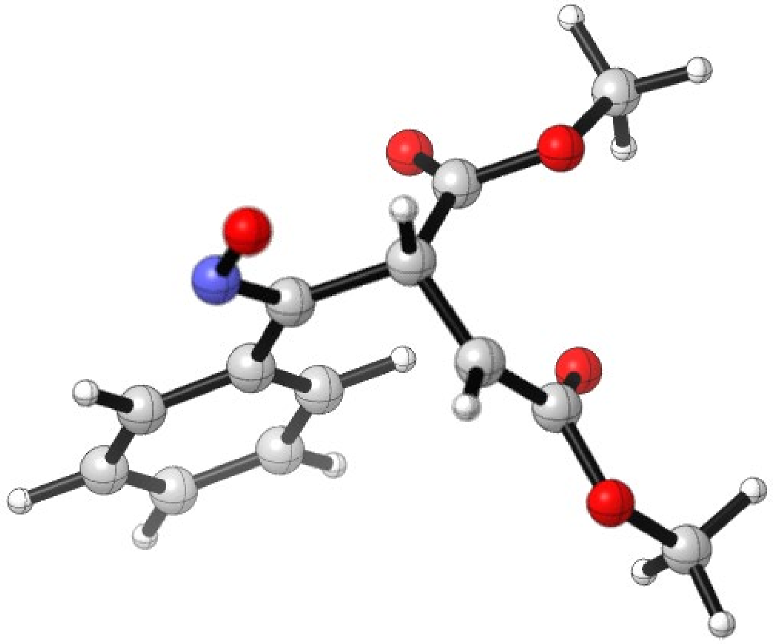

Sum of Electronic and Zero-point Energies = -933.461762 Hartree
Sum of Electronic and Thermal Energies = -933.443322 Hartree
Sum of Electronic and Thermal Enthalpies = -933.442378 Hartree
Sum of Electronic and Thermal Free Energies = -933.512080 Hartree

Dipole Moment = 8.5475 Debye

-1 2

C	4.38059	0.69910	0.21730
C	3.22807	1.41044	0.52941
C	2.02280	0.74851	0.83696
C	2.02595	-0.65629	0.81719
C	3.18271	-1.36261	0.49534
C	4.36999	-0.69713	0.19457
H	5.29514	1.24025	-0.01329
H	3.23911	2.49462	0.54597
H	1.12273	-1.20764	1.05064

H	3.14964	-2.44899	0.48613
H	5.27021	-1.25362	-0.05175
C	0.81705	1.52658	1.17002
N	0.93619	2.84088	1.22412
O	-0.08639	3.57694	1.37489
C	-0.57865	0.95053	1.38458
C	-1.04896	0.48502	0.06454
H	-1.25348	1.23578	-0.68902
H	-1.15604	1.84875	1.67332
C	-1.16554	-0.88201	-0.28595
O	-0.93315	-1.86358	0.42845
O	-1.61088	-1.06281	-1.57888
C	-1.74464	-2.41447	-1.97133
H	-2.09066	-2.39353	-3.00631
H	-0.78766	-2.94076	-1.90255
H	-2.46961	-2.93906	-1.34082
C	-0.73726	-0.04052	2.51476
O	0.12470	-0.43791	3.26207
O	-2.03290	-0.40131	2.64710
C	-2.25354	-1.49044	3.52998
H	-1.91900	-1.24822	4.54241
H	-3.32820	-1.67424	3.51544
H	-1.71060	-2.36823	3.16812

Ph-oximyl-radical-Nu-attack-R-from-IRC

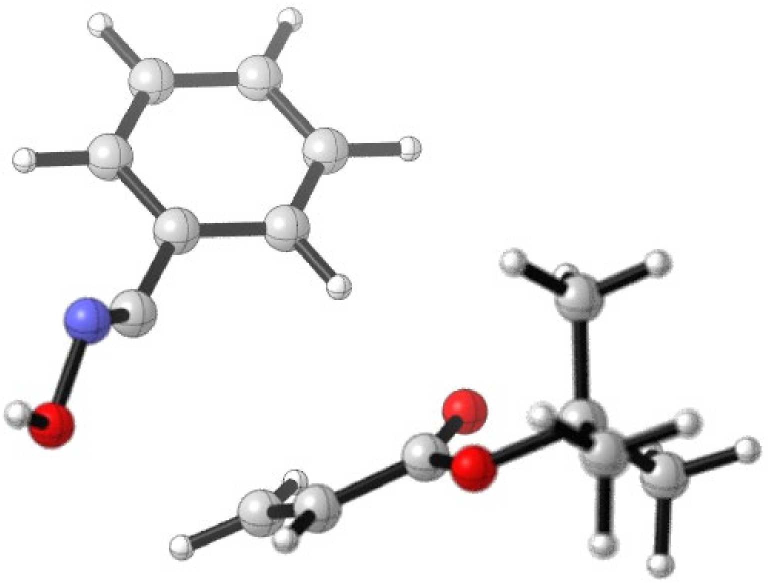

Sum of Electronic and Zero-point Energies = -824.010759 Hartree
Sum of Electronic and Thermal Energies = -823.990384 Hartree
Sum of Electronic and Thermal Enthalpies = -823.989440 Hartree

Sum of Electronic and Thermal Free Energies = -824.064295 Hartree

Dipole Moment = 1.4204 Debye

0 2

C	-2.86423	-1.75884	0.16256
C	-2.05609	-0.63067	0.23876
C	-2.62530	0.64400	0.06237
C	-4.01011	0.76927	-0.15386
C	-4.80457	-0.36851	-0.22303
C	-4.23564	-1.63388	-0.06719
H	-2.42014	-2.74085	0.29012
H	-0.99178	-0.72927	0.43561
H	-4.44193	1.75750	-0.27430
H	-5.87076	-0.26917	-0.40029
H	-4.86074	-2.51957	-0.11934
C	-1.80818	1.81748	0.17289
N	-1.40875	2.72067	-0.57135
O	-0.65790	3.73503	0.04825
H	-0.44408	4.32178	-0.68816
C	0.51211	1.41183	1.98864
C	1.49015	1.22422	1.10232
H	2.04065	2.04760	0.66033
H	0.22044	2.40911	2.29955
H	-0.01667	0.56103	2.41133
C	1.84642	-0.14828	0.65790
O	1.23617	-1.15112	0.97138
O	2.91965	-0.12491	-0.14225
C	3.45347	-1.35055	-0.72830
C	2.40821	-1.99702	-1.63456
H	2.87546	-2.81583	-2.19014
H	1.57218	-2.39454	-1.05843
H	2.03186	-1.26495	-2.35552
C	4.63500	-0.84590	-1.54911
H	5.35881	-0.34083	-0.90370
H	5.13073	-1.68582	-2.04351
H	4.29516	-0.13978	-2.31170
C	3.92892	-2.29478	0.37311
H	4.45876	-3.13677	-0.08279
H	4.62307	-1.77395	1.03957
H	3.09187	-2.67899	0.95671

Ph-oximyl-radical-Nu-attack-tBu-P-from-TS_triplet

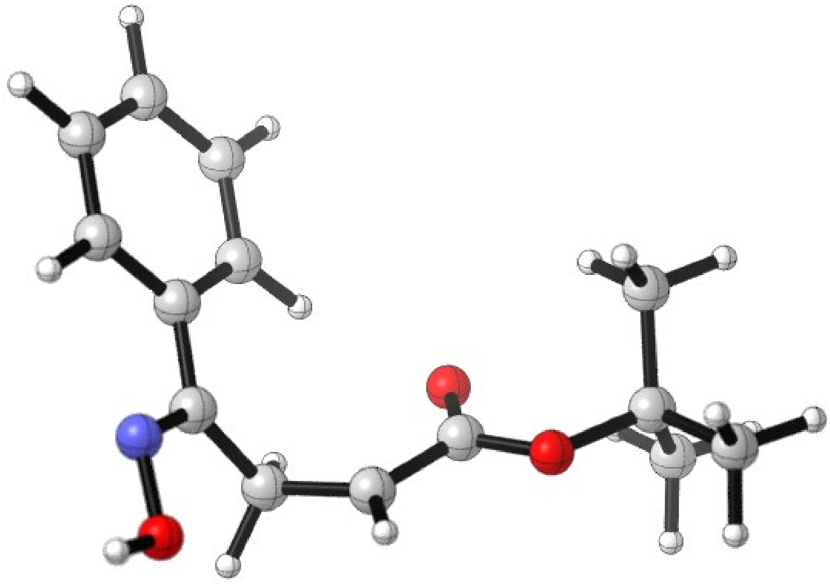

Sum of Electronic and Zero-point Energies = -823.763341 Hartree
Sum of Electronic and Thermal Energies = -823.745091 Hartree
Sum of Electronic and Thermal Enthalpies = -823.744147 Hartree
Sum of Electronic and Thermal Free Energies = -823.811951 Hartree

Dipole Moment = 8.8727 Debye

1 3

C	-2.23783	-2.07258	0.20629
C	-1.30724	-1.10246	0.49970
C	-1.58618	0.26507	0.18777
C	-2.83639	0.60420	-0.43528
C	-3.75739	-0.37739	-0.70814
C	-3.46435	-1.71714	-0.38905
H	-2.02683	-3.11225	0.42968
H	-0.34690	-1.38089	0.92389
H	-3.04637	1.64014	-0.67094
H	-4.70681	-0.12505	-1.16630
H	-4.19457	-2.49010	-0.60855
C	-0.63228	1.28519	0.49301
N	-0.94072	2.48888	-0.00350
O	-0.00058	3.37969	0.25715
H	-0.30775	4.21793	-0.13054
C	0.64252	1.05641	1.26996
C	1.79098	0.84932	0.33153
H	2.23684	1.68065	-0.20093
H	0.82049	1.93574	1.89673
H	0.53644	0.18387	1.91469
C	2.31895	-0.50486	0.14123
O	1.80871	-1.48045	0.67565
O	3.37604	-0.50581	-0.65635

C	4.10675	-1.74857	-0.97257
C	3.18077	-2.71107	-1.70849
H	3.76992	-3.55816	-2.07064
H	2.39433	-3.09075	-1.05497
H	2.73118	-2.21690	-2.57500
C	5.22041	-1.25598	-1.88615
H	5.84918	-0.52812	-1.36707
H	5.84414	-2.09962	-2.19179
H	4.80485	-0.78710	-2.78196
C	4.67385	-2.34514	0.31077
H	5.32892	-3.18110	0.05026
H	5.27182	-1.60003	0.84332
H	3.88564	-2.71475	0.96782

Ph-oximyl-radical-Nu-attack-tBu-P-N-oxide_regioisomer

Sum of Electronic and Zero-point Energies = -823.505483 Hartree
Sum of Electronic and Thermal Energies = -823.487228 Hartree
Sum of Electronic and Thermal Enthalpies = -823.486284 Hartree
Sum of Electronic and Thermal Free Energies = -823.552115 Hartree

Dipole Moment = 5.1271 Debye

-1 2

C	-4.17067	-0.07775	-1.29406
C	-2.86662	0.34812	-1.04098
C	-2.19210	0.00047	0.14805
C	-2.91017	-0.79318	1.07547
C	-4.20695	-1.21037	0.81954
C	-4.85767	-0.86160	-0.37133
H	-4.65270	0.21517	-2.22407
H	-2.37369	0.97512	-1.77815
H	-2.40898	-1.06773	1.99720
H	-4.72235	-1.82252	1.55642
H	-5.87289	-1.19336	-0.56883
C	-0.82946	0.43569	0.42839
N	-0.34289	0.25409	1.63816
O	0.84071	0.70166	1.85697
C	-0.20896	2.65227	-0.49869
H	-1.19186	3.05422	-0.72155
H	0.44647	3.21126	0.15735
C	1.47454	0.82384	-0.53759
O	2.38864	1.61418	-0.64246
O	1.63702	-0.51667	-0.54776
C	2.93703	-1.09553	-0.30446
C	3.56501	-0.52904	0.96975
H	4.41104	-1.16386	1.25906
H	3.91680	0.49193	0.81559
H	2.81546	-0.50983	1.76639
C	2.61514	-2.57577	-0.11293

H	2.10520	-2.96978	-0.99756
H	3.53568	-3.14584	0.05115
H	1.95568	-2.69828	0.75075
C	3.83470	-0.89439	-1.52600
H	4.78392	-1.42180	-1.37615
H	3.34912	-1.30017	-2.42014
H	4.03441	0.16758	-1.67850
C	-0.00269	1.17865	-0.61594
H	-0.31909	0.82292	-1.60759

Ph-oximyl-radical-Nu-attack-tBu-P-N-oxide_triplet

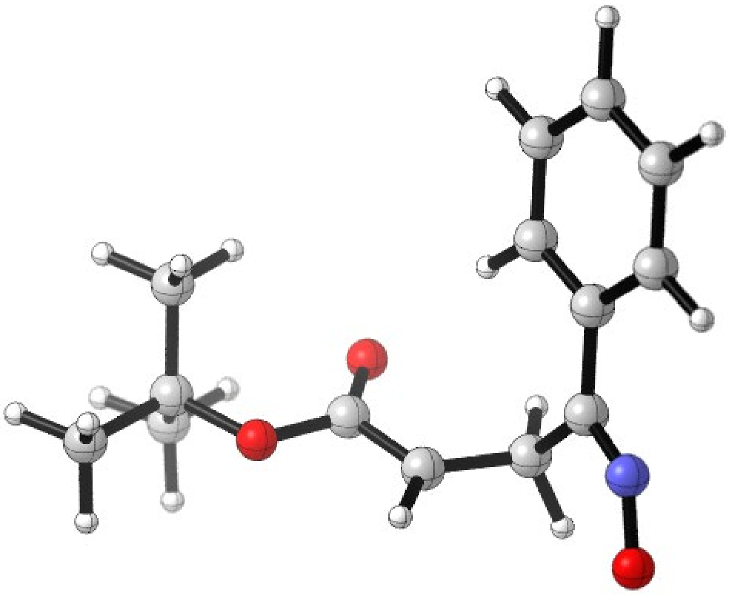

Sum of Electronic and Zero-point Energies = -823.436205 Hartree
Sum of Electronic and Thermal Energies = -823.418161 Hartree
Sum of Electronic and Thermal Enthalpies = -823.417216 Hartree
Sum of Electronic and Thermal Free Energies = -823.485391 Hartree

Dipole Moment = 2.4394 Debye

0 3

C	-1.77963	0.27646	1.22539
C	-0.56392	-0.09701	2.00692
C	0.60225	0.79154	1.61949
H	-2.18471	1.27997	1.29061
H	-0.31918	-1.14697	1.83140
H	-0.74936	0.03970	3.07913
N	0.79566	1.87659	2.28183
O	0.22856	2.36554	3.25509
C	-2.42384	-0.68712	0.34501

O	-2.00454	-1.82135	0.16789
O	-3.51599	-0.16774	-0.23750
C	-4.30889	-0.94921	-1.18154
C	1.47589	0.49345	0.46635
C	1.16016	-0.55662	-0.40398
C	2.62875	1.25600	0.22637
C	1.98397	-0.83437	-1.49452
H	0.27214	-1.16183	-0.24484
C	3.44448	0.97405	-0.86138
H	2.88106	2.06811	0.90160
C	3.12551	-0.07379	-1.72807
H	1.72591	-1.65098	-2.16127
H	4.33495	1.57053	-1.03287
H	3.76552	-0.29403	-2.57641
C	-4.89961	-2.17223	-0.48417
H	-5.60457	-2.66342	-1.16176
H	-4.12356	-2.88602	-0.20600
H	-5.44550	-1.86515	0.41306
C	-3.45850	-1.32701	-2.39226
H	-4.10429	-1.76596	-3.15880
H	-2.98665	-0.43363	-2.81256
H	-2.68655	-2.05025	-2.12750
C	-5.41101	0.02426	-1.58345
H	-6.08537	-0.45287	-2.29975
H	-5.98844	0.32906	-0.70643
H	-4.98123	0.91697	-2.04591

Ph-oximyl-radical-Nu-attack-tBu-P

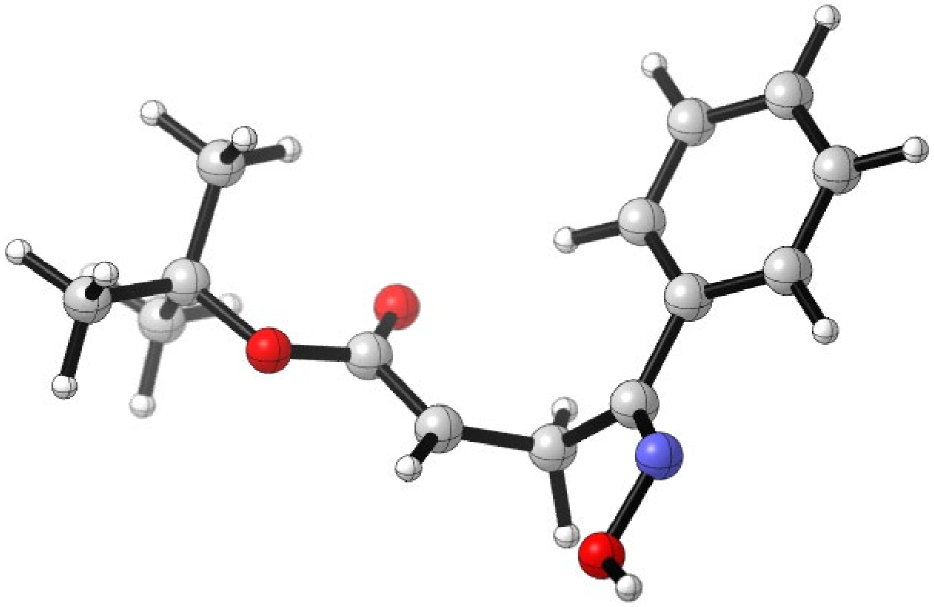

Sum of Electronic and Zero-point Energies = -824.064799 Hartree
Sum of Electronic and Thermal Energies = -824.046632 Hartree
Sum of Electronic and Thermal Enthalpies = -824.045688 Hartree
Sum of Electronic and Thermal Free Energies = -824.112869 Hartree

Dipole Moment = 1.4363 Debye

0 2

C	3.84007	-0.76877	-0.49427
C	2.97790	0.30858	-0.33067
C	1.65376	0.09985	0.08050
C	1.21187	-1.20508	0.32760
C	2.08093	-2.28248	0.15740
C	3.39424	-2.06953	-0.25164
H	4.86504	-0.59441	-0.80646
H	3.31872	1.32192	-0.51366
H	0.18896	-1.39248	0.64313
H	1.72472	-3.28999	0.34748
H	4.07002	-2.90972	-0.37719
C	0.72674	1.24896	0.23695
N	1.01840	2.32480	-0.39181
O	0.07065	3.32122	-0.18391
H	0.41798	4.06613	-0.68751
C	-0.52704	1.12227	1.08771
C	-1.72285	0.90160	0.22849
H	-2.03847	1.66793	-0.46907
H	-0.42294	0.30314	1.80022
H	-0.64182	2.06346	1.63782
C	-2.45779	-0.35167	0.28403
O	-2.14929	-1.28840	1.00688
O	-3.50865	-0.34343	-0.55530
C	-4.38335	-1.50357	-0.67543
C	-5.39923	-1.05405	-1.71977
H	-6.12606	-1.85095	-1.89958
H	-5.93186	-0.16380	-1.37427
H	-4.89734	-0.81659	-2.66174
C	-5.07317	-1.78272	0.65798
H	-4.36161	-2.12420	1.41031
H	-5.57192	-0.87783	1.01832
H	-5.83262	-2.55706	0.51247
C	-3.59323	-2.70862	-1.18121
H	-3.04984	-2.44429	-2.09350
H	-2.88500	-3.06355	-0.43199
H	-4.28998	-3.51799	-1.41982

Ph-oximyl-radical-Nu-attack-tBu-P_regioisomer

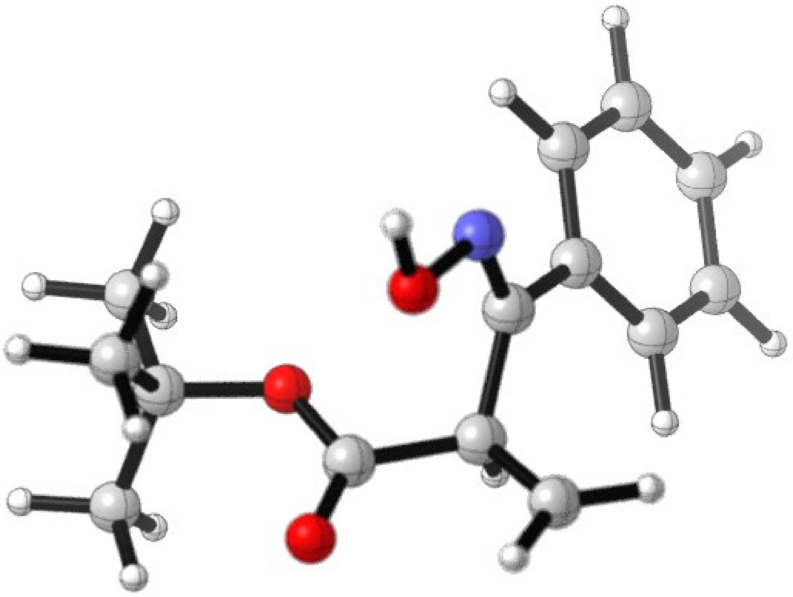

Sum of Electronic and Zero-point Energies = -824.048641 Hartree
Sum of Electronic and Thermal Energies = -824.029751 Hartree
Sum of Electronic and Thermal Enthalpies = -824.028806 Hartree
Sum of Electronic and Thermal Free Energies = -824.096561 Hartree

Dipole Moment = 2.1462 Debye

0 2

C	3.07420	4.64702	-2.13531
C	2.51633	3.54028	-1.49800
C	2.09000	2.43340	-2.24022
C	2.23880	2.45126	-3.63333
C	2.80028	3.55426	-4.26647
C	3.21983	4.65644	-3.52018
H	3.39309	5.50158	-1.54708
H	2.39766	3.55422	-0.41804
H	1.91557	1.58825	-4.20533
H	2.91783	3.55236	-5.34560
H	3.66081	5.51535	-4.01601
C	1.47220	1.26360	-1.56852
N	0.62835	0.58657	-2.25062
O	0.12744	-0.48274	-1.52553
H	-0.49807	-0.89874	-2.12955
C	0.70518	1.32114	0.81015
H	0.25789	2.30369	0.73302
H	0.31553	0.59157	1.50578
C	2.21740	-0.52503	0.06396
O	1.89711	-1.20209	1.00947
O	3.03290	-0.90016	-0.92504
C	3.54456	-2.26524	-1.00365
C	2.38065	-3.24322	-1.13632

H	2.77547	-4.24626	-1.32483
H	1.77561	-3.26613	-0.22908
H	1.74802	-2.95435	-1.98065
C	4.37470	-2.24004	-2.28186
H	5.18342	-1.50893	-2.19713
H	4.80969	-3.22679	-2.46332
H	3.74773	-1.96769	-3.13539
C	4.42510	-2.56922	0.20550
H	4.92454	-3.53065	0.05115
H	5.19441	-1.79828	0.31217
H	3.83813	-2.61942	1.12294
C	1.80990	0.93905	-0.11733
H	2.72660	1.49997	0.12297

Ph-oximyl-radical-Nu-attack-tBu-R-from-IRC_

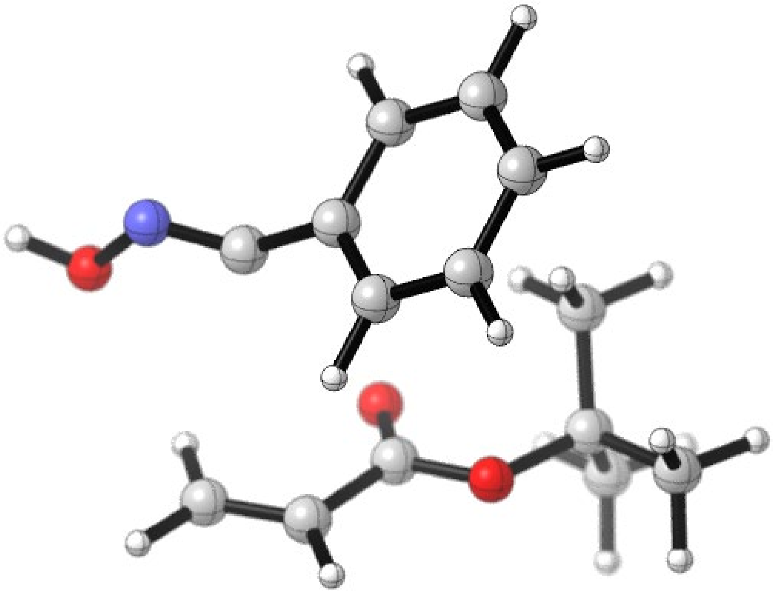

Sum of Electronic and Zero-point Energies = -824.012313 Hartree
Sum of Electronic and Thermal Energies = -823.992225 Hartree
Sum of Electronic and Thermal Enthalpies = -823.991281 Hartree
Sum of Electronic and Thermal Free Energies = -824.063307 Hartree

Dipole Moment = 2.3752 Debye

0 2

C	1.17581	2.10746	-1.46136
C	0.11392	1.81559	-0.61293
C	-1.00309	1.11489	-1.10193
C	-1.01841	0.68014	-2.44020
C	0.04981	0.97740	-3.27764

C	1.14752	1.69306	-2.79410
H	2.03032	2.65763	-1.08026
H	0.13131	2.12817	0.42619
H	-1.87374	0.12219	-2.80676
H	0.02756	0.64983	-4.31214
H	1.97992	1.92094	-3.45208
C	-2.09603	0.79720	-0.22890
N	-3.25389	1.19657	-0.06022
O	-4.00351	0.52131	0.91497
H	-4.85155	0.98234	0.89036
C	-1.41956	0.43634	2.90532
H	-1.71027	1.24136	3.57254
H	-2.19729	-0.21753	2.51953
C	0.21117	-0.84597	1.61150
O	-0.55904	-1.69966	1.23190
O	1.50131	-0.75412	1.25427
C	2.09940	-1.72733	0.34614
C	1.38004	-1.71703	-1.00018
H	1.94206	-2.33724	-1.70596
H	0.36503	-2.10644	-0.91645
H	1.34112	-0.69785	-1.39568
C	3.52441	-1.20880	0.18872
H	4.02495	-1.16445	1.16003
H	4.09212	-1.87160	-0.47027
H	3.51324	-0.20513	-0.24673
C	2.09325	-3.10846	0.99653
H	2.66382	-3.80220	0.37139
H	2.56885	-3.06303	1.98105
H	1.07671	-3.48863	1.10564
C	-0.14902	0.25793	2.54540
H	0.65904	0.89671	2.88859

Ph-oximyl-radical-Nu-attack-tBu-TS_regioisomer-vi

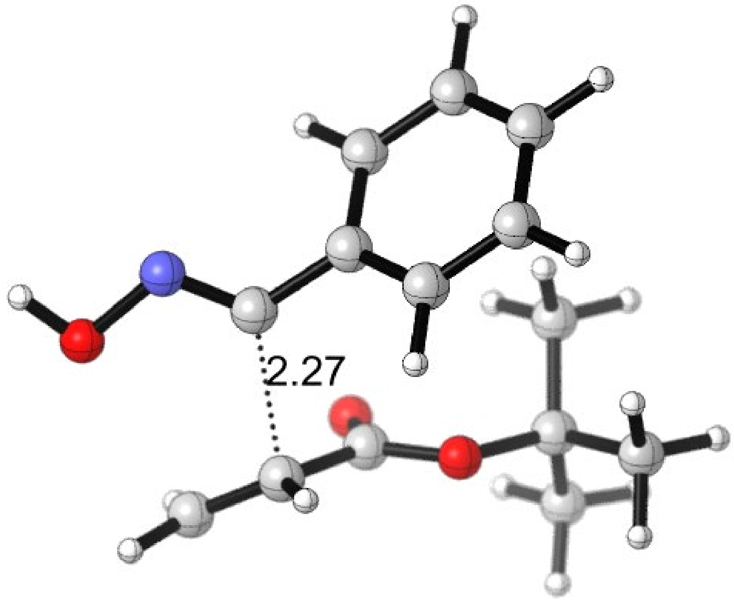

Sum of Electronic and Zero-point Energies = -824.001629 Hartree
Sum of Electronic and Thermal Energies = -823.982800 Hartree
Sum of Electronic and Thermal Enthalpies = -823.981856 Hartree
Sum of Electronic and Thermal Free Energies = -824.049434 Hartree

Dipole Moment = 1.7915 Debye

0 2

C	3.59904	2.44829	-3.12962
C	2.73174	2.31597	-2.04954
C	1.48349	1.70385	-2.22186
C	1.13248	1.19033	-3.47943
C	2.00742	1.31930	-4.55376
C	3.23915	1.95235	-4.38344
H	4.55907	2.93641	-2.99263
H	3.01554	2.68734	-1.06949
H	0.17155	0.70053	-3.59828
H	1.72794	0.92512	-5.52572
H	3.91987	2.05251	-5.22292
C	0.54924	1.59178	-1.10311
N	-0.65817	1.90726	-1.16189
O	-1.43278	1.66503	-0.03099
H	-2.30345	1.98715	-0.29415
C	0.32151	0.32941	1.59242
H	-0.08492	1.05483	2.28514
H	-0.17082	-0.63213	1.49557
C	2.01952	-0.47435	0.01847
O	1.45535	-1.49442	-0.30758
O	3.30230	-0.18256	-0.22757
C	4.16171	-1.11325	-0.95530
C	3.61610	-1.34946	-2.36056

H	4.34075	-1.94476	-2.92538
H	2.66502	-1.88269	-2.33503
H	3.48012	-0.39416	-2.87601
C	5.48872	-0.36562	-1.01763
H	5.85670	-0.15471	-0.00954
H	6.23226	-0.96994	-1.54476
H	5.36145	0.58041	-1.55211
C	4.30788	-2.40954	-0.16248
H	5.06830	-3.03601	-0.63877
H	4.63429	-2.19118	0.85896
H	3.36781	-2.96133	-0.12862
C	1.37216	0.63501	0.78599
H	1.97755	1.51806	0.96492

Ph-oximyl-radical-Nu-attack-tBu-TS_triplet

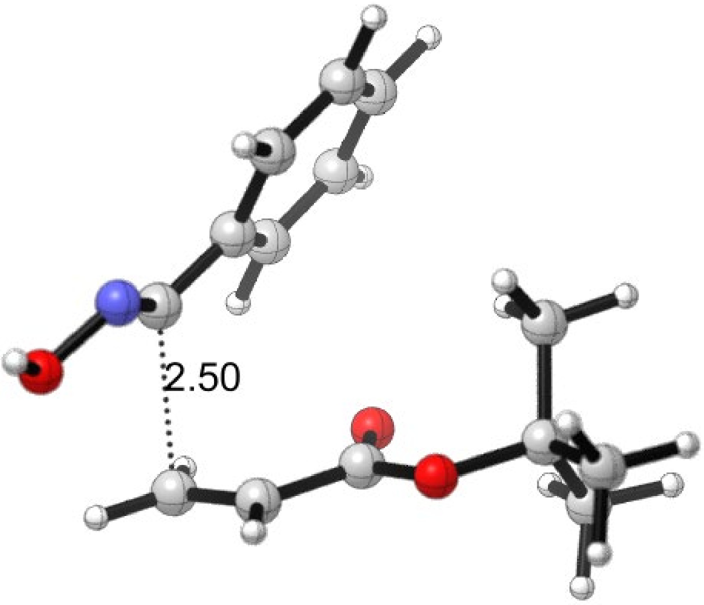

Sum of Electronic and Zero-point Energies = -823.699234 Hartree
Sum of Electronic and Thermal Energies = -823.680436 Hartree
Sum of Electronic and Thermal Enthalpies = -823.679492 Hartree
Sum of Electronic and Thermal Free Energies = -823.748284 Hartree

Dipole Moment = 2.9752 Debye

1 3

C	-1.53336	-2.18656	0.51502
C	-1.08610	-0.96433	0.97249
C	-1.05419	0.15139	0.08677
C	-1.48462	0.00196	-1.27158
C	-1.91772	-1.22655	-1.71019

C	-1.94280	-2.31835	-0.81949
H	-1.56069	-3.04151	1.18056
H	-0.75229	-0.83926	1.99541
H	-1.45676	0.86071	-1.93346
H	-2.24201	-1.35841	-2.73636
H	-2.28832	-3.28394	-1.17651
C	-0.63526	1.40642	0.56995
N	-0.62218	2.49310	-0.16315
O	-0.32135	3.58731	0.50964
H	-0.35082	4.31862	-0.13464
C	1.30326	1.50181	2.14606
C	2.10026	1.18991	1.10504
H	2.61343	1.94214	0.51332
H	1.16310	2.52803	2.46857
H	0.87846	0.70890	2.75564
C	2.25943	-0.23900	0.70074
O	1.69137	-1.14563	1.27846
O	3.03546	-0.34636	-0.36506
C	3.29296	-1.65366	-0.99737
C	1.97070	-2.23795	-1.48875
H	2.17801	-3.12139	-2.09906
H	1.32956	-2.53266	-0.65555
H	1.44703	-1.50816	-2.11593
C	4.19660	-1.28979	-2.16709
H	5.11504	-0.81865	-1.80843
H	4.46290	-2.19336	-2.72100
H	3.69020	-0.59954	-2.84715
C	4.01155	-2.56881	-0.01256
H	4.31817	-3.47836	-0.53656
H	4.91153	-2.07920	0.36962
H	3.37000	-2.84809	0.82380

Ph-oximyl-radical-Nu-attack-TS-FUMERATE

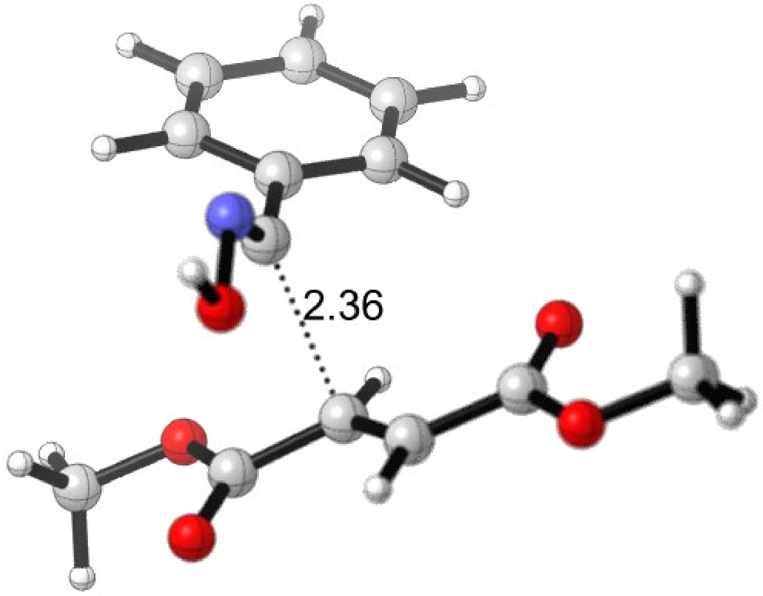

Sum of Electronic and Zero-point Energies = -933.949184 Hartree
Sum of Electronic and Thermal Energies = -933.929596 Hartree
Sum of Electronic and Thermal Enthalpies = -933.928651 Hartree
Sum of Electronic and Thermal Free Energies = -934.000680 Hartree

Dipole Moment = 1.5853 Debye

0 2

C	-2.44759	-2.00749	0.25062
C	-1.41494	-1.10415	0.02270
C	-1.70904	0.26290	-0.10788
C	-3.03158	0.71541	0.02733
C	-4.05280	-0.19850	0.25960
C	-3.76403	-1.55970	0.36995
H	-2.22302	-3.06548	0.33989
H	-0.38346	-1.43857	-0.05817
H	-3.24122	1.77686	-0.05894
H	-5.07619	0.15035	0.35462
H	-4.56407	-2.26974	0.55388
C	-0.64352	1.21386	-0.31737
N	-0.36610	1.98866	-1.24573
O	0.71048	2.84673	-1.03431
H	0.76998	3.33727	-1.86371
C	0.96232	1.04200	1.39975
C	2.17103	0.99500	0.79469
H	2.77630	1.88613	0.67524
H	0.42000	0.12990	1.62962
C	2.63182	-0.27593	0.21149
O	1.97461	-1.29745	0.15521
O	3.87934	-0.18070	-0.27482
C	4.39593	-1.37353	-0.86658

H	5.39949	-1.12316	-1.20496
H	4.42514	-2.17915	-0.12989
H	3.76876	-1.68100	-1.70606
C	0.51057	2.30305	2.05756
O	1.14343	3.32958	2.10944
O	-0.70665	2.14841	2.60586
C	-1.23572	3.31136	3.24366
H	-2.21235	3.01930	3.62508
H	-0.58034	3.62859	4.05756
H	-1.32765	4.12656	2.52235

Ph-oximyl-radical-Nu-attack-TS-MALEATE-ii

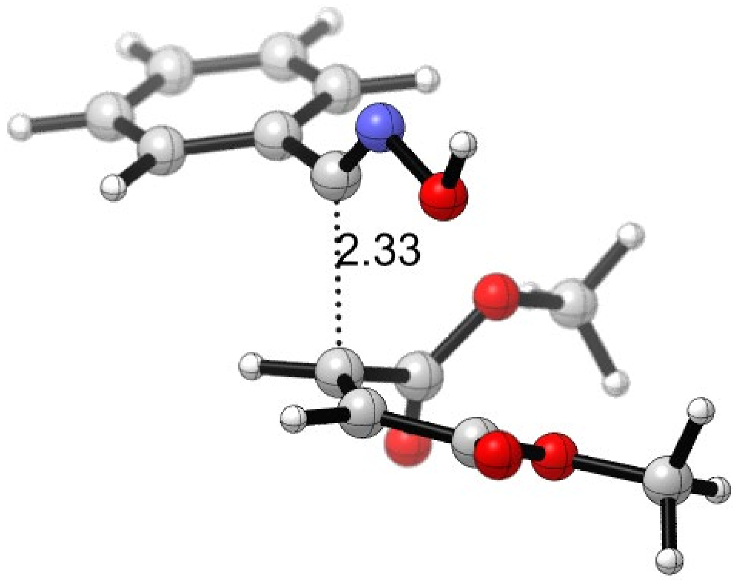

Sum of Electronic and Zero-point Energies = -933.936776 Hartree
Sum of Electronic and Thermal Energies = -933.917184 Hartree
Sum of Electronic and Thermal Enthalpies = -933.916240 Hartree
Sum of Electronic and Thermal Free Energies = -933.988937 Hartree

Dipole Moment = 3.4141 Debye

0 2

C	-0.64748	-1.98623	0.14096
C	-0.17954	-0.75152	-0.16520
H	-0.90097	0.03318	-0.37889
C	1.18257	-0.45792	-0.73402
O	1.34146	-0.20052	-1.90241
O	2.16891	-0.45956	0.16900
C	3.47308	-0.24541	-0.37086
H	4.15474	-0.29830	0.47649

H	3.70545	-1.02135	-1.10420
H	3.52747	0.73241	-0.85511
H	-1.69330	-2.11768	0.39500
C	0.15596	-3.21508	0.28417
O	-0.24620	-4.21653	0.83409
O	1.37350	-3.12199	-0.27729
C	2.20423	-4.27136	-0.11939
H	2.38233	-4.46357	0.94088
H	1.72913	-5.14650	-0.56742
H	3.13588	-4.03550	-0.63097
C	1.56054	3.76588	1.26897
C	1.48815	2.41628	1.59439
C	0.24497	1.76111	1.59263
C	-0.91298	2.46974	1.23257
C	-0.82940	3.81991	0.91225
C	0.40522	4.47045	0.92719
H	2.52108	4.27110	1.28141
H	2.37865	1.85423	1.85746
H	-1.87133	1.95868	1.22276
H	-1.72883	4.36638	0.64749
H	0.46719	5.52274	0.66972
C	0.18503	0.34546	1.86335
N	0.26039	-0.32382	2.90503
O	0.30252	-1.70810	2.75336
H	0.24842	-2.03701	3.65949

Ph-oximyl-radical-Nu-attackSTYRENE-P-from-TS-ii

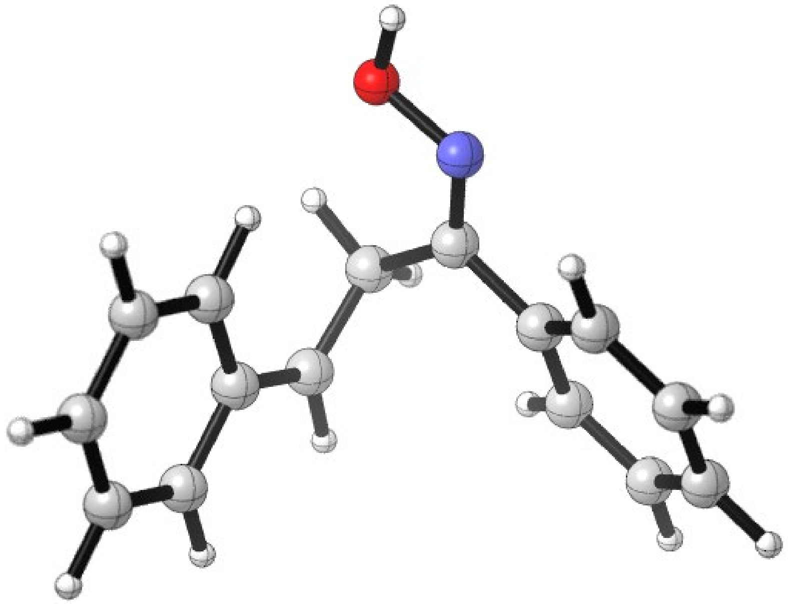

Sum of Electronic and Zero-point Energies = -709.385085 Hartree

Sum of Electronic and Thermal Energies = -709.370674 Hartree
Sum of Electronic and Thermal Enthalpies = -709.369730 Hartree
Sum of Electronic and Thermal Free Energies = -709.428699 Hartree

Dipole Moment = 0.4760 Debye

0 2

C	-1.57077	-1.75621	0.12348
C	-1.10651	-0.67692	0.87247
C	-1.28799	0.63458	0.41997
C	-1.93139	0.84399	-0.80655
C	-2.39172	-0.23405	-1.55442
C	-2.21449	-1.53853	-1.09209
H	-1.42749	-2.76717	0.49174
H	-0.60920	-0.86438	1.81913
H	-2.05768	1.85987	-1.16454
H	-2.88357	-0.05685	-2.50575
H	-2.57094	-2.37920	-1.67891
C	-0.81025	1.78920	1.22803
N	-1.40643	2.90192	1.01829
O	-0.89676	3.94887	1.77809
H	-1.45246	4.69412	1.52323
C	0.33012	1.59062	2.21399
C	1.55231	1.01563	1.56090
H	1.83795	-0.00541	1.79339
H	0.55190	2.55299	2.68250
H	-0.01576	0.92339	3.01023
C	2.34098	1.72873	0.61982
C	3.45960	1.10289	0.01116
C	2.05504	3.06987	0.25462
C	4.24617	1.77753	-0.90717
H	3.69256	0.07501	0.27691
C	2.85100	3.73538	-0.66687
H	1.20989	3.58371	0.70386
C	3.94802	3.09994	-1.25472
H	5.09672	1.27655	-1.35922
H	2.61621	4.76187	-0.93203
H	4.56460	3.62783	-1.97484

Ph-oximyl-radical-Nu-attackSTYRENE-R-from-TS-ii

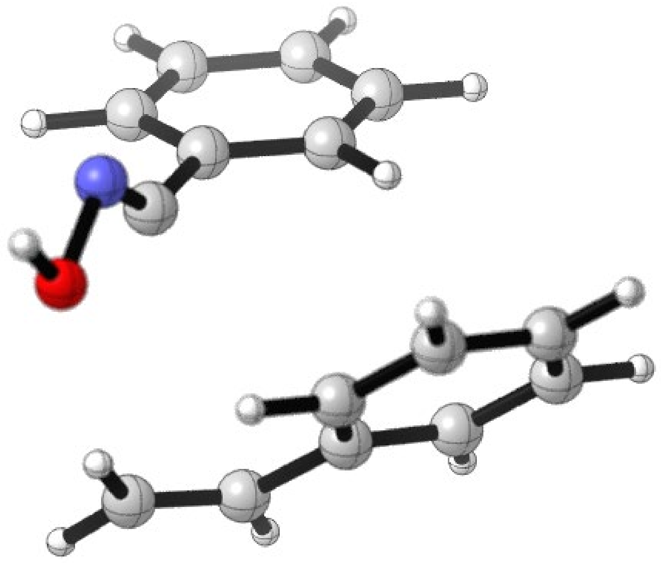

Sum of Electronic and Zero-point Energies = -709.333121 Hartree
Sum of Electronic and Thermal Energies = -709.316697 Hartree
Sum of Electronic and Thermal Enthalpies = -709.315752 Hartree
Sum of Electronic and Thermal Free Energies = -709.380016 Hartree

Dipole Moment = 1.1981 Debye

0 2

C	-0.30101	-0.74164	-1.56481
C	-0.17516	0.55255	-1.07677
C	-1.18015	1.08666	-0.24778
C	-2.28892	0.29338	0.10472
C	-2.39996	-0.99870	-0.39203
C	-1.40931	-1.52072	-1.22682
H	0.47399	-1.14607	-2.20839
H	0.69191	1.15838	-1.32319
H	-3.05047	0.70446	0.75931
H	-3.26170	-1.60304	-0.12716
H	-1.49920	-2.53274	-1.60828
C	-1.02798	2.39439	0.30156
N	-1.39133	3.55872	0.10817
O	-0.90752	4.49929	1.04663
H	-1.28939	5.32898	0.73308
C	0.57363	2.07120	3.19081
C	1.44922	1.28973	2.55077
H	1.70618	0.32495	2.98640
H	0.25372	3.03460	2.80251
H	0.13800	1.75154	4.13161
C	2.11179	1.58774	1.26779
C	2.86667	0.58625	0.64350
C	2.00550	2.83569	0.63351

C	3.48774	0.81101	-0.58352
H	2.95582	-0.38496	1.12378
C	2.62250	3.06102	-0.59308
H	1.43032	3.63326	1.09534
C	3.36537	2.05064	-1.20882
H	4.06568	0.01900	-1.04998
H	2.52361	4.03072	-1.07150
H	3.84731	2.23199	-2.16445

Ph-oximyl-radical-Nu-attackSTYRENE-TS_cyclopropyl (ground state)

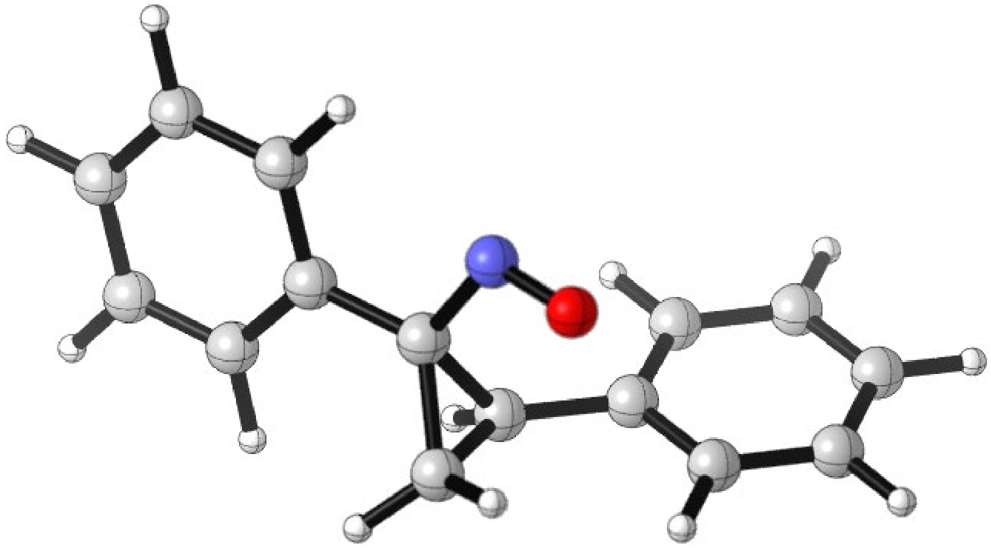

Sum of Electronic and Zero-point Energies = -708.823083 Hartree
Sum of Electronic and Thermal Energies = -708.809422 Hartree
Sum of Electronic and Thermal Enthalpies = -708.808478 Hartree
Sum of Electronic and Thermal Free Energies = -708.865010 Hartree

Dipole Moment = 6.8850 Debye

-1 2

C	-3.92338	-0.82309	0.79394
C	-2.63179	-0.33923	0.99472
C	-1.93168	0.32203	-0.02605
C	-2.57616	0.48029	-1.26246
C	-3.86483	-0.00936	-1.46544
C	-4.55015	-0.66364	-0.44126
H	-4.44162	-1.32416	1.60797
H	-2.16670	-0.47697	1.96792
H	-2.03333	1.00290	-2.04217

H	-4.34061	0.12593	-2.43385
H	-5.55690	-1.04024	-0.60111
C	-0.54208	0.85263	0.16090
N	-0.07090	1.64506	-0.97135
O	0.61761	2.68878	-0.59951
C	-0.03703	1.13974	1.53468
C	0.41066	-0.14803	0.89769
H	-0.10163	-1.04199	1.25236
H	0.67761	1.95982	1.53776
H	-0.70725	1.08454	2.38963
C	1.80047	-0.43129	0.47400
C	2.27051	-1.75149	0.55646
C	2.67878	0.55295	-0.01106
C	3.57116	-2.08770	0.18798
H	1.59878	-2.52659	0.91982
C	3.97879	0.21228	-0.37978
H	2.32117	1.57390	-0.12193
C	4.43821	-1.10189	-0.28200
H	3.90530	-3.11931	0.26749
H	4.63926	0.98830	-0.75845
H	5.45409	-1.35509	-0.57347

Ph-oximyl-radical

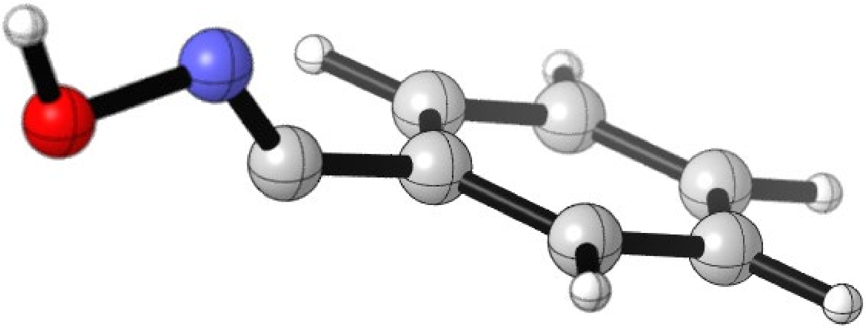

Sum of Electronic and Zero-point Energies = -399.935681 Hartree
Sum of Electronic and Thermal Energies = -399.927704 Hartree
Sum of Electronic and Thermal Enthalpies = -399.926759 Hartree
Sum of Electronic and Thermal Free Energies = -399.969834 Hartree

Dipole Moment = 1.5703 Debye

```
0 2
C                -2.08369      1.20893      0.02157
C                -0.69875      1.21635     -0.08190
C                 0.00931      0.00000     -0.11805
C                -0.69894     -1.21623     -0.08186
C                -2.08388     -1.20859      0.02161
C                -2.78088      0.00022      0.07309
H                -2.62347      2.14961      0.06140
H                -0.15069      2.15167     -0.12764
H                -0.15103     -2.15164     -0.12758
H                -2.62380     -2.14919      0.06146
H                -3.86324      0.00031      0.14998
C                 1.43183     -0.00012     -0.26612
N                 2.43405     -0.00018      0.45483
O                 3.67450     -0.00029     -0.19722
H                 4.30198     -0.00032      0.53675
```

chlorine-radical

Sum of Electronic and Zero-point Energies = -460.100501 Hartree
Sum of Electronic and Thermal Energies = -460.099084 Hartree
Sum of Electronic and Thermal Enthalpies = -460.098140 Hartree
Sum of Electronic and Thermal Free Energies = -460.116178 Hartree

Dipole Moment = 0.0000 Debye

```
0 2
Cl               -1.24873     -0.11675      0.00000
```

HCl

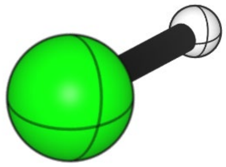

Sum of Electronic and Zero-point Energies = -460.755191 Hartree
Sum of Electronic and Thermal Energies = -460.752831 Hartree
Sum of Electronic and Thermal Enthalpies = -460.751887 Hartree
Sum of Electronic and Thermal Free Energies = -460.773071 Hartree

Dipole Moment = 1.5064 Debye

```
0 1
H                 3.24340     -0.82539     -1.87740
Cl                2.79866     -1.77713     -2.60961
```

oxime-H-atom-abstraction-Cl-HFIP-complex-P

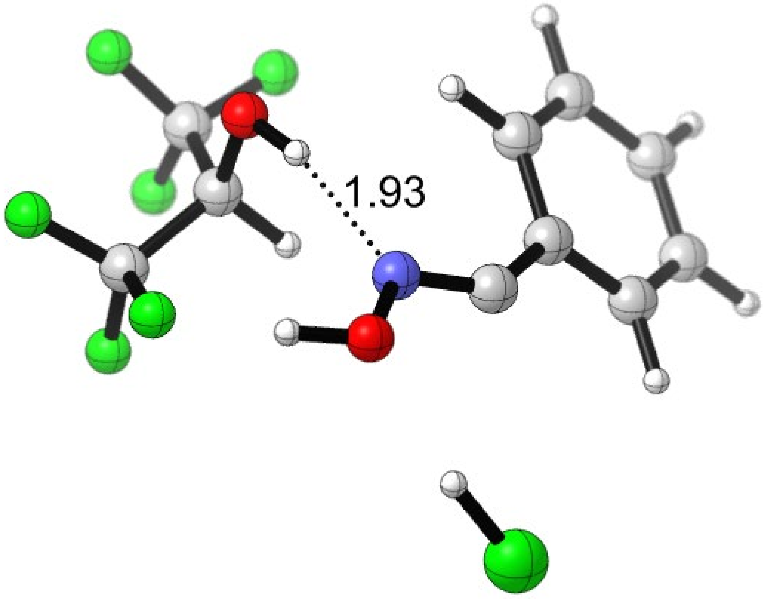

Sum of Electronic and Zero-point Energies = -1650.227092 Hartree
Sum of Electronic and Thermal Energies = -1650.205743 Hartree
Sum of Electronic and Thermal Enthalpies = -1650.204798 Hartree
Sum of Electronic and Thermal Free Energies = -1650.283940 Hartree

Dipole Moment = 3.1432 Debye

0 2

C	-0.11422	-1.81487	-1.39245
H	2.36896	-2.73223	-1.92955
C	-1.31099	-2.04065	-0.60832
C	-1.50170	-3.30127	-0.02766
C	-2.25837	-1.02094	-0.41661
C	-2.62567	-3.53523	0.75773
H	-0.76224	-4.07835	-0.19500
C	-3.37523	-1.26576	0.37365
H	-2.11456	-0.04657	-0.87470
C	-3.55988	-2.51953	0.95909
H	-2.77281	-4.50929	1.21200
H	-4.10142	-0.47554	0.53290
H	-4.43585	-2.70456	1.57252
N	0.53196	-0.78191	-1.65376
O	1.65149	-0.95263	-2.48007
H	2.13657	-0.11971	-2.38018
C	1.70809	1.97738	0.39343
C	-0.60026	2.38301	1.39064
C	0.22596	1.58552	0.38394
H	0.17533	0.53109	0.69240
F	-0.69245	3.67105	1.06259
F	-0.06978	2.30063	2.61861
F	-1.84168	1.88107	1.44515
F	2.31896	1.59613	1.51844

F	2.32973	1.34902	-0.63201
F	1.89989	3.28508	0.23161
O	-0.32192	1.81097	-0.87170
H	-0.11093	1.02679	-1.41130
Cl	2.50703	-3.86084	-1.31389

oxime-H-atom-abstraction-Cl-HFIP-complex-R

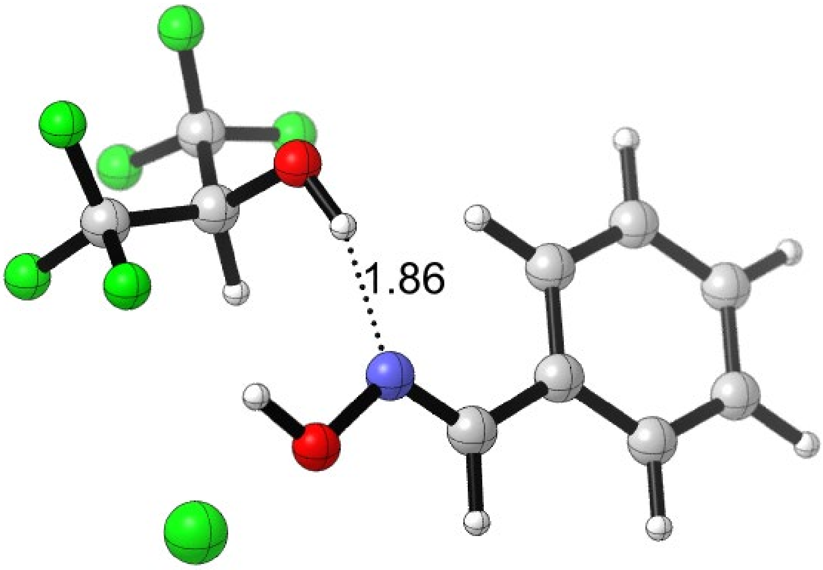

Sum of Electronic and Zero-point Energies = -1650.238135 Hartree
Sum of Electronic and Thermal Energies = -1650.217763 Hartree
Sum of Electronic and Thermal Enthalpies = -1650.216819 Hartree
Sum of Electronic and Thermal Free Energies = -1650.292830 Hartree

Dipole Moment = 5.3227 Debye

0 2

C	-3.04969	-1.90269	-0.70023
H	-2.40251	-2.76857	-0.85937
C	-4.39153	-2.11748	-0.15139
C	-4.85534	-3.43458	-0.05764
C	-5.20541	-1.06300	0.28860
C	-6.12342	-3.69956	0.45198
H	-4.22015	-4.25185	-0.38865
C	-6.46839	-1.33288	0.79983
H	-4.85090	-0.03793	0.24568
C	-6.93128	-2.64842	0.87956
H	-6.47699	-4.72311	0.51801
H	-7.09202	-0.51442	1.14379
H	-7.91862	-2.85162	1.28170
N	-2.60712	-0.74192	-0.99520
O	-1.30190	-0.80489	-1.46224
H	-1.06005	0.11470	-1.65732

C	-1.18040	2.67280	0.11020
C	-3.07356	2.68602	1.81422
C	-2.42765	1.95718	0.63730
H	-2.09287	0.97713	1.00954
F	-3.58283	3.86603	1.45984
F	-2.19267	2.89163	2.80281
F	-4.07210	1.93675	2.30339
F	-0.18041	2.66865	0.99247
F	-0.74313	2.01624	-0.99542
F	-1.41935	3.93104	-0.25186
O	-3.37389	1.86364	-0.37389
H	-3.27605	0.97769	-0.78031
Cl	0.12145	-0.96397	0.65358

oxime-H-atom-abstraction-Cl-HFIP-complex-triplet-P-from-IRC

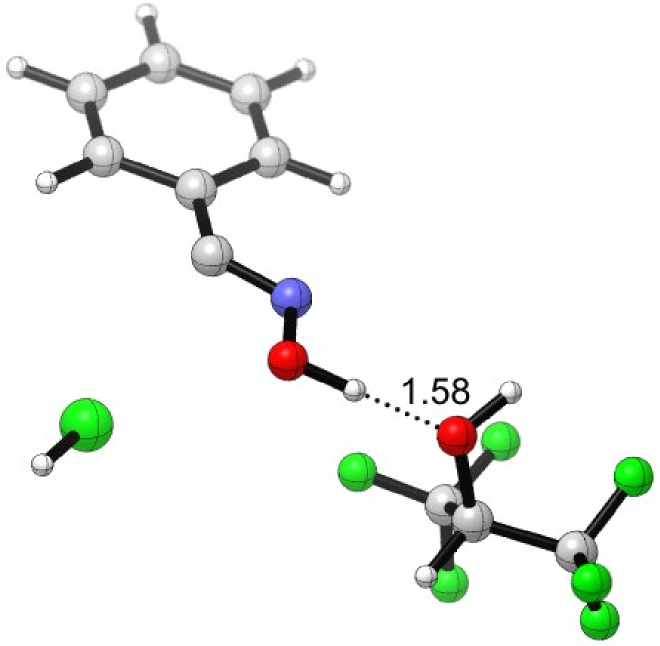

Sum of Electronic and Zero-point Energies = -1649.911041 Hartree
Sum of Electronic and Thermal Energies = -1649.888952 Hartree
Sum of Electronic and Thermal Enthalpies = -1649.888008 Hartree
Sum of Electronic and Thermal Free Energies = -1649.972720 Hartree

Dipole Moment = 10.9917 Debye

1 3

C	-0.11395	-2.56454	-0.73440
H	-2.61314	-1.45875	-3.40218
C	-0.51555	-3.52527	0.20184
C	-1.21257	-4.67636	-0.26202
C	-0.23604	-3.35907	1.59398
C	-1.61799	-5.63358	0.64302
H	-1.41850	-4.77398	-1.32305
C	-0.64643	-4.32888	2.48105

H	0.29261	-2.47233	1.92702
C	-1.33455	-5.46062	2.00866
H	-2.15301	-6.51473	0.30802
H	-0.44427	-4.22207	3.54079
H	-1.65611	-6.21823	2.71676
N	0.46242	-1.41816	-0.50010
O	0.70749	-0.69580	-1.55253
H	1.10800	0.17041	-1.20889
C	-0.29399	2.21397	0.56540
C	1.67273	3.79963	0.10507
C	0.82932	2.61978	-0.39470
H	0.38015	2.89301	-1.35281
F	2.47222	3.38001	1.10206
F	0.91465	4.79108	0.55094
F	2.44835	4.24680	-0.87917
F	-1.20860	3.16382	0.69234
F	-0.88935	1.11261	0.07351
F	0.19379	1.90501	1.77162
O	1.67934	1.51038	-0.60387
H	2.30731	1.42764	0.13329
Cl	-2.69215	-1.61356	-2.13075

oxime-H-atom-abstraction-Cl-HFIP-complex-triplet-P-from-TS-iv

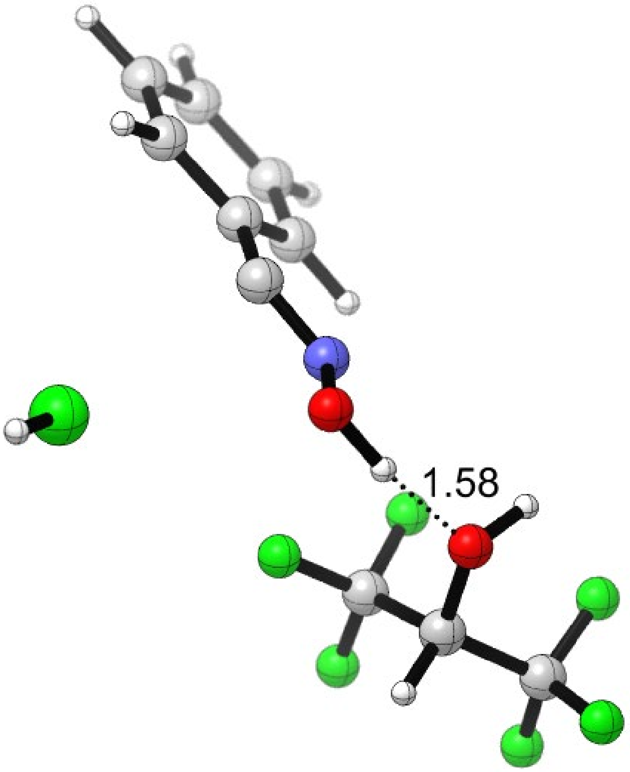

Sum of Electronic and Zero-point Energies = -1649.865601 Hartree
Sum of Electronic and Thermal Energies = -1649.843749 Hartree
Sum of Electronic and Thermal Enthalpies = -1649.842805 Hartree

Sum of Electronic and Thermal Free Energies = -1649.924311 Hartree

Dipole Moment = 20.4534 Debye

1 3

C	-4.01887	-1.57201	-1.07092
H	-6.83319	-0.61397	-3.63257
C	-4.36714	-2.40928	-0.00379
C	-4.93931	-3.67919	-0.28853
C	-4.17157	-1.98676	1.34694
C	-5.29258	-4.51030	0.75155
H	-5.08810	-3.96836	-1.32347
C	-4.53400	-2.83113	2.37109
H	-3.75970	-1.00119	1.53681
C	-5.08807	-4.08801	2.07509
H	-5.72807	-5.48278	0.55451
H	-4.39883	-2.52955	3.40329
H	-5.37038	-4.74665	2.89040
N	-3.49751	-0.38022	-0.98127
O	-3.30038	0.24033	-2.10510
H	-2.94751	1.15035	-1.82574
C	-4.28710	2.63989	0.35183
C	-2.35050	4.30707	0.38500
C	-3.21527	3.36791	-0.45736
H	-3.70070	3.94287	-1.24871
F	-1.57536	3.56133	1.19358
F	-3.08720	5.11972	1.12375
F	-1.55623	5.01869	-0.40327
F	-5.11229	3.47323	0.96049
F	-4.99615	1.86300	-0.47962
F	-3.71420	1.84119	1.26423
O	-2.36734	2.40407	-1.05441
H	-1.70726	2.13031	-0.39590
Cl	-6.66674	-0.65940	-2.36176

oxime-H-atom-abstraction-Cl-HFIP-complex-triplet-R-from-IRC-vii

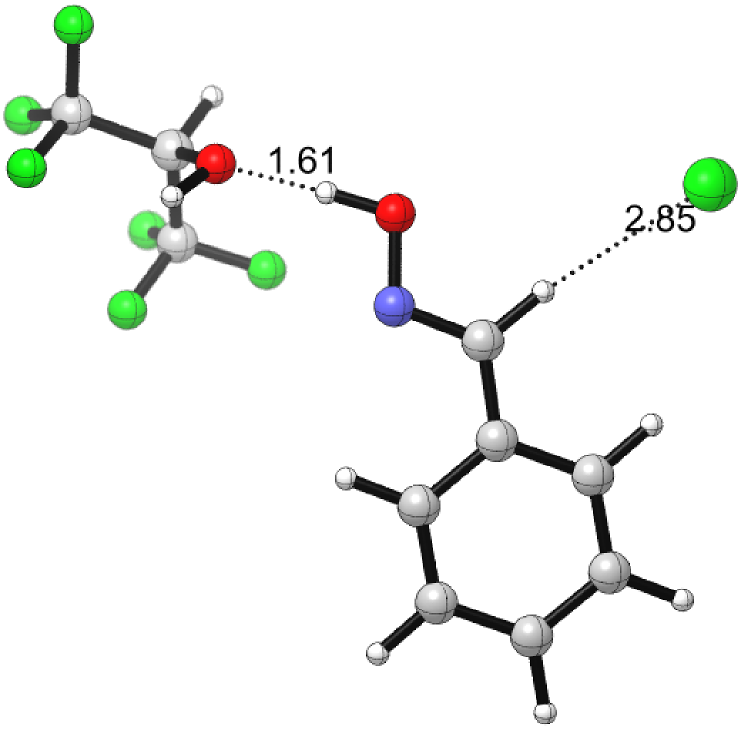


```
Sum of Electronic and Zero-point Energies = -1649.923290 Hartree
Sum of Electronic and Thermal Energies = -1649.902383 Hartree
Sum of Electronic and Thermal Enthalpies = -1649.901439 Hartree
Sum of Electronic and Thermal Free Energies = -1649.982920 Hartree

Dipole Moment = 12.1727 Debye

1 3
C                -0.69209     -2.41855     -0.79496
H                -1.13356     -2.53287     -1.78675
C                -0.83847     -3.41780      0.19034
C                -1.56647     -4.59695     -0.14867
C                -0.28482     -3.27296      1.50155
C                -1.73069     -5.59406      0.78507
H                -1.98263     -4.69234     -1.14733
C                -0.45873     -4.27782      2.42410
H                 0.26166     -2.36951      1.74828
C                -1.17786     -5.43575      2.07032
H                -2.28032     -6.49550      0.53946
H                -0.04700     -4.18359      3.42243
H                -1.30992     -6.22393      2.80519
N                -0.00763     -1.31341     -0.51052
O                 0.01108     -0.50168     -1.53020
H                 0.53070      0.31027     -1.23859
C                -0.10457      2.32849      1.01676
C                 1.75980      3.81165      0.06068
C                 0.71545      2.72284     -0.21595
H                 0.02744      3.08795     -0.98267
F                 2.77731      3.28222      0.76268
```

F	1.24550	4.82286	0.74686
F	2.25056	4.26618	-1.08965
F	-0.86076	3.32512	1.45362
F	-0.90683	1.30307	0.67865
F	0.68942	1.90920	2.00805
O	1.37128	1.57862	-0.71858
H	2.16485	1.39130	-0.19024
Cl	-2.72770	-2.91522	-4.11918

oxime-H-atom-abstraction-Cl-HFIP-complex-triplet-R-from-TS-iv

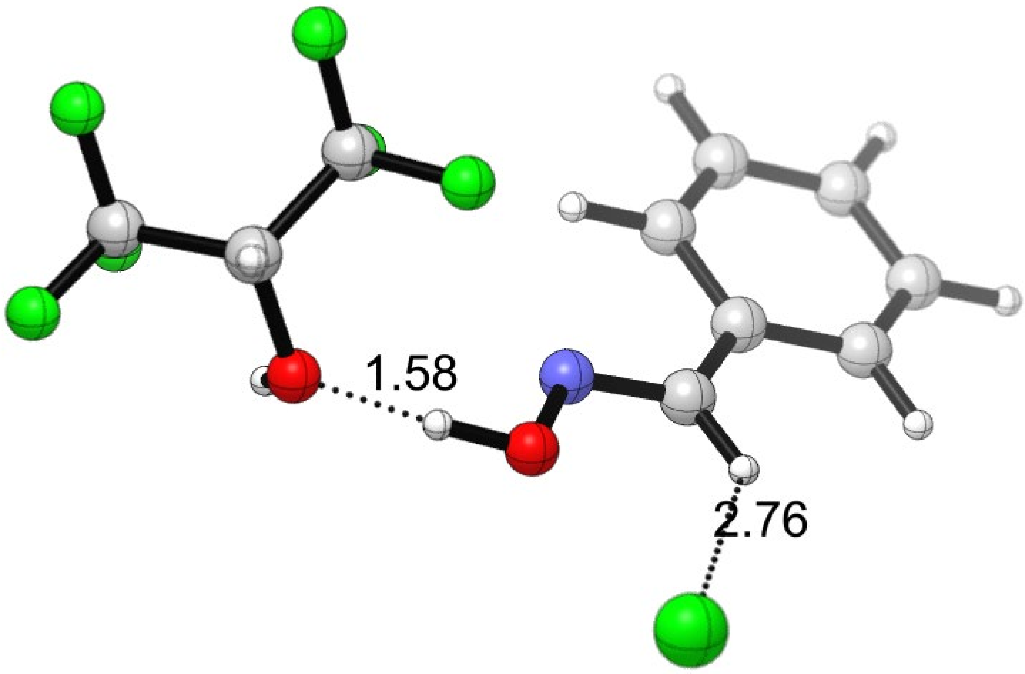

Sum of Electronic and Zero-point Energies = -1649.880646 Hartree
Sum of Electronic and Thermal Energies = -1649.860271 Hartree
Sum of Electronic and Thermal Enthalpies = -1649.859327 Hartree
Sum of Electronic and Thermal Free Energies = -1649.936807 Hartree

Dipole Moment = 21.7280 Debye

1 3

C	-4.67043	-1.49337	-1.13444
H	-5.07459	-1.77990	-2.10717
C	-4.75070	-2.34700	-0.01427
C	-5.32174	-3.64296	-0.18146
C	-4.28440	-1.93026	1.27307
C	-5.40997	-4.49462	0.89372
H	-5.67618	-3.94386	-1.16254
C	-4.38421	-2.79205	2.33806
H	-3.87524	-0.93237	1.38716

C	-4.94102	-4.07137	2.15182
H	-5.83680	-5.48421	0.78049
H	-4.04054	-2.49241	3.32116
H	-5.01416	-4.74644	2.99868
N	-4.08754	-0.30778	-0.99138
O	-4.12821	0.38948	-2.09181
H	-3.66492	1.26197	-1.86494
C	-4.20292	2.69751	0.66793
C	-2.19976	4.21807	0.21680
C	-3.34399	3.39898	-0.38225
H	-3.98138	4.05687	-0.97679
F	-1.29705	3.36944	0.74150
F	-2.62741	5.03325	1.16663
F	-1.59968	4.91944	-0.73508
F	-4.75480	3.54532	1.51818
F	-5.17697	2.02198	0.04130
F	-3.46649	1.80725	1.34937
O	-2.78643	2.41842	-1.23659
H	-1.99984	2.04813	-0.80257
Cl	-5.29522	-0.43235	-4.50349

oxime-H-atom-abstraction-Cl-HFIP-complex-triplet-R-from-TS-iv__ii

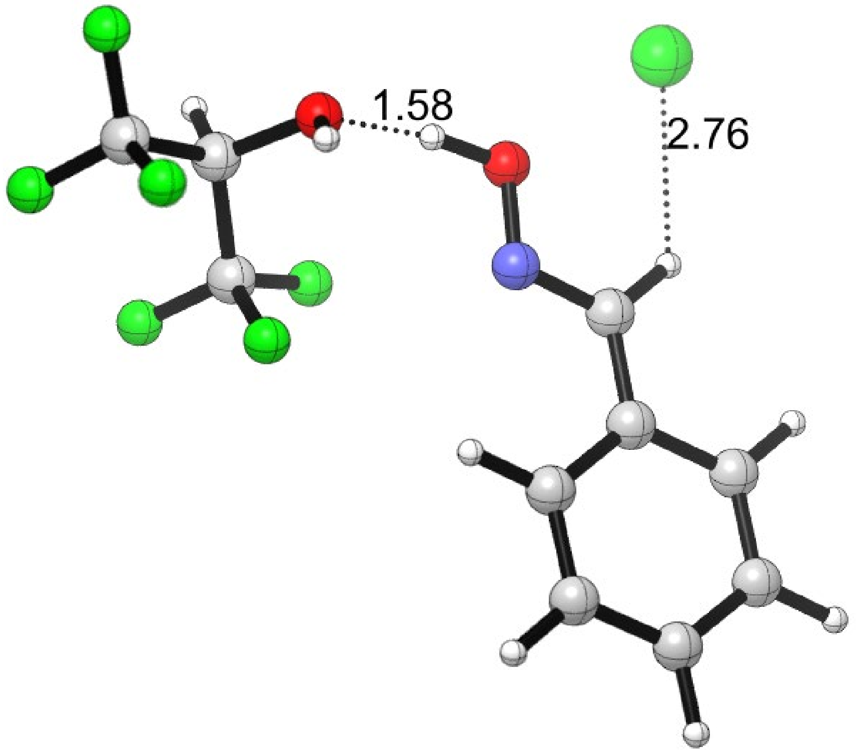

Sum of Electronic and Zero-point Energies = -1649.880669 Hartree
Sum of Electronic and Thermal Energies = -1649.860288 Hartree
Sum of Electronic and Thermal Enthalpies = -1649.859343 Hartree
Sum of Electronic and Thermal Free Energies = -1649.936933 Hartree

Dipole Moment = 21.8361 Debye

1 3			
C	-4.75104	-1.47969	-1.14569
H	-5.19910	-1.75597	-2.10202
C	-4.79463	-2.33871	-0.02764
C	-5.38911	-3.62622	-0.17742
C	-4.26821	-1.93591	1.24083
C	-5.44219	-4.48337	0.89573
H	-5.78937	-3.91645	-1.14399
C	-4.33310	-2.80312	2.30414
H	-3.84183	-0.94396	1.34293
C	-4.91396	-4.07406	2.13478
H	-5.88653	-5.46664	0.79544
H	-3.94348	-2.51407	3.27316
H	-4.95926	-4.75344	2.98015
N	-4.14835	-0.30191	-1.02139
O	-4.22539	0.40084	-2.11639
H	-3.74335	1.26701	-1.90498
C	-4.16674	2.69681	0.64960
C	-2.16225	4.19437	0.13187
C	-3.33883	3.39356	-0.42814
H	-3.98961	4.06324	-0.99433
F	-1.25011	3.33104	0.61472
F	-2.54267	5.00693	1.10378
F	-1.59108	4.89611	-0.83733
F	-4.67704	3.54664	1.52344
F	-5.17104	2.03618	0.05560
F	-3.41661	1.79435	1.29929
O	-2.82674	2.41116	-1.30840
H	-2.02790	2.03001	-0.90741
Cl	-5.45012	-0.42163	-4.50063

oxime-H-atom-abstraction-Cl-HFIP-complex-TS-ii

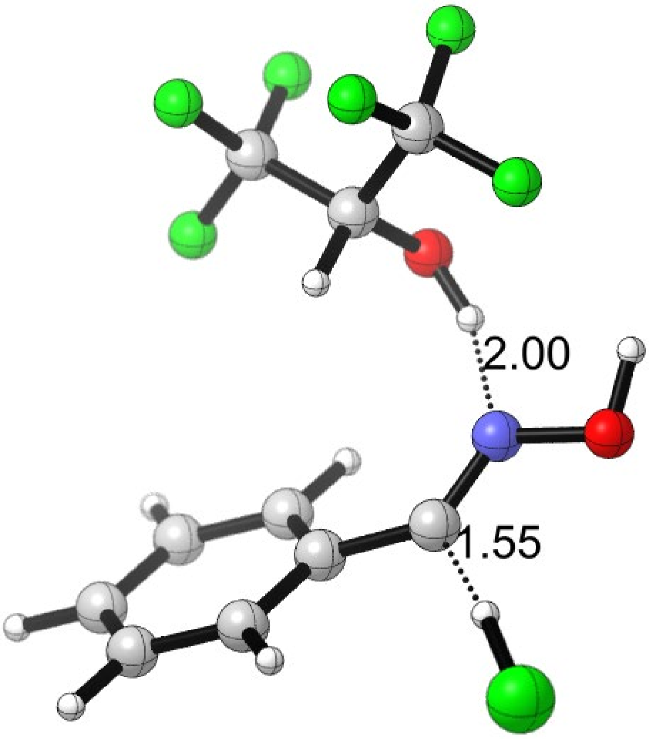

Sum of Electronic and Zero-point Energies = -1650.223812 Hartree
Sum of Electronic and Thermal Energies = -1650.203355 Hartree
Sum of Electronic and Thermal Enthalpies = -1650.202411 Hartree
Sum of Electronic and Thermal Free Energies = -1650.279735 Hartree

Dipole Moment = 3.0675 Debye

0 2

C	-2.81616	-1.57686	-0.96057
H	-2.04485	-2.86097	-1.36549
C	-4.05929	-1.80208	-0.24169
C	-4.21257	-2.97998	0.50249
C	-5.09025	-0.84743	-0.27837
C	-5.37995	-3.18696	1.22903
H	-3.41927	-3.72127	0.50445
C	-6.24798	-1.06256	0.45890
H	-4.97717	0.05720	-0.86793
C	-6.39414	-2.22974	1.21052
H	-5.49846	-4.09620	1.80848
H	-7.03619	-0.31723	0.44593
H	-7.30342	-2.39423	1.77962
N	-2.22841	-0.51680	-1.25003
O	-1.10747	-0.57872	-2.04353
H	-0.67951	0.28102	-1.91464
C	-1.40746	2.29200	0.87199
C	-3.80884	2.60683	1.66384
C	-2.86819	1.84872	0.72935
H	-2.90991	0.79026	1.02402
F	-3.92432	3.89156	1.33165

F	-3.38098	2.53806	2.93176
F	-5.02865	2.05497	1.61042
F	-0.88294	1.90918	2.03897
F	-0.67900	1.70650	-0.10668
F	-1.25134	3.60853	0.74989
O	-3.31283	2.06994	-0.56851
H	-3.03021	1.30493	-1.09732
Cl	-1.39617	-4.06925	-1.61193

oxime-H-atom-abstraction-Cl-P-ii

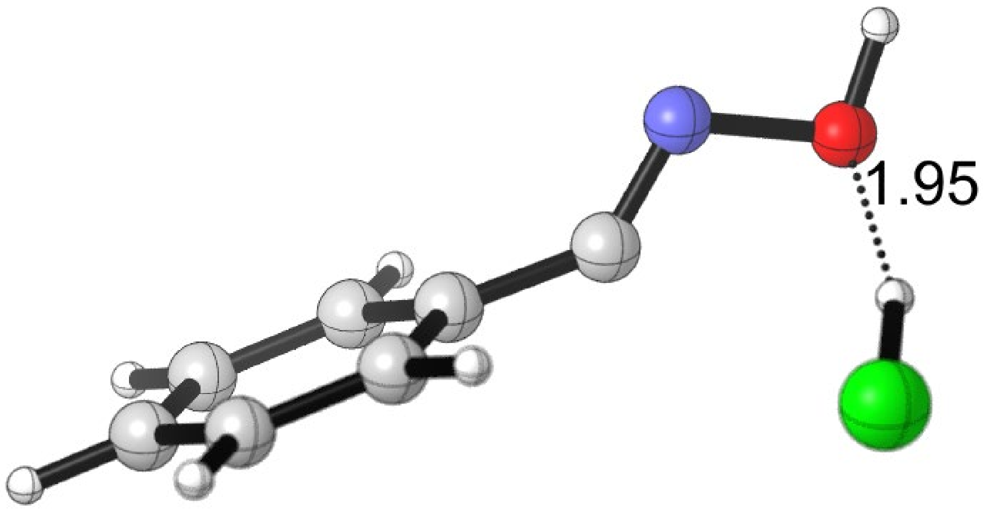

Sum of Electronic and Zero-point Energies = -860.699564 Hartree
Sum of Electronic and Thermal Energies = -860.688850 Hartree
Sum of Electronic and Thermal Enthalpies = -860.687906 Hartree
Sum of Electronic and Thermal Free Energies = -860.739064 Hartree

Dipole Moment = 2.7610 Debye

0 2

C	-2.22008	1.20191	0.48300
C	-0.84491	1.31564	0.32641
C	-0.10048	0.20817	-0.12184
C	-0.75640	-0.99649	-0.43949
C	-2.13152	-1.09420	-0.27533
C	-2.86650	0.00069	0.18471
H	-2.79155	2.05274	0.83949
H	-0.33392	2.24525	0.55394
H	-0.17541	-1.83457	-0.81084

H	-2.63464	-2.02626	-0.51096
H	-3.94171	-0.08058	0.30618
N	2.35825	0.26954	0.32316
O	3.53649	0.46054	-0.44533
H	4.24221	0.34606	0.20545
C	1.30773	0.31684	-0.32474
H	3.24605	-0.81972	-1.87304
Cl	2.79601	-1.78280	-2.61397

oxime-H-atom-abstraction-Cl-P

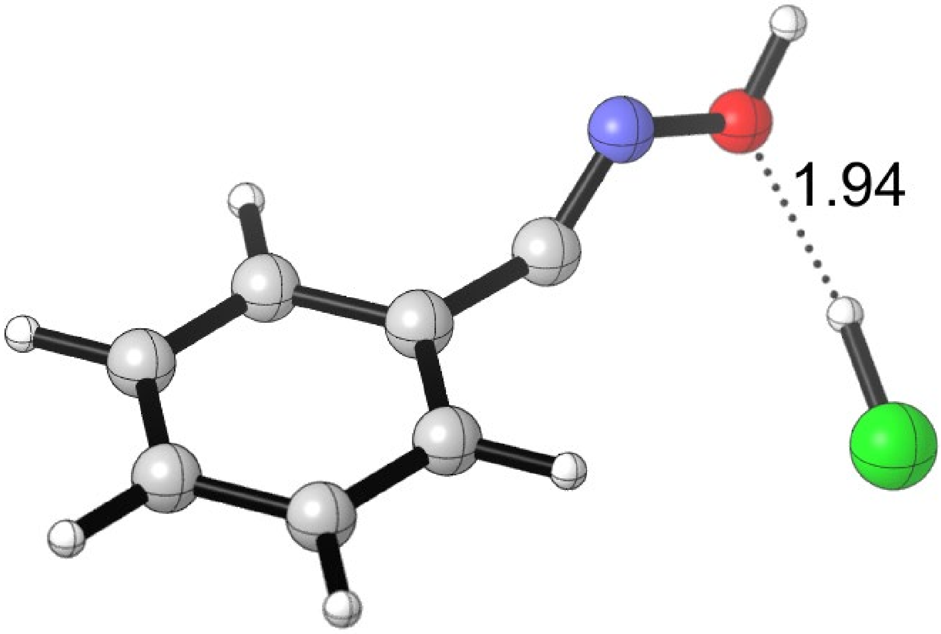

Sum of Electronic and Zero-point Energies = -860.699622 Hartree
Sum of Electronic and Thermal Energies = -860.688901 Hartree
Sum of Electronic and Thermal Enthalpies = -860.687957 Hartree
Sum of Electronic and Thermal Free Energies = -860.739055 Hartree

Dipole Moment = 2.6567 Debye

0 2

C	-2.21697	1.19749	0.49101
C	-0.83728	1.30349	0.37552
C	-0.09030	0.20553	-0.09210
C	-0.74845	-0.98060	-0.47105
C	-2.12833	-1.07018	-0.34846
C	-2.86594	0.01440	0.13142
H	-2.79047	2.04054	0.86239
H	-0.32499	2.21983	0.64931
H	-0.16512	-1.81048	-0.85669

H	-2.63300	-1.98812	-0.63158
H	-3.94475	-0.06055	0.22052
N	2.36341	0.16319	0.39376
O	3.55626	0.38555	-0.34472
H	4.25037	0.19521	0.30072
C	1.32256	0.30587	-0.25521
H	3.23485	-0.74804	-1.89327
Cl	2.70773	-1.59038	-2.72479

oxime-H-atom-abstraction-Cl-P_from-triplet-IRC

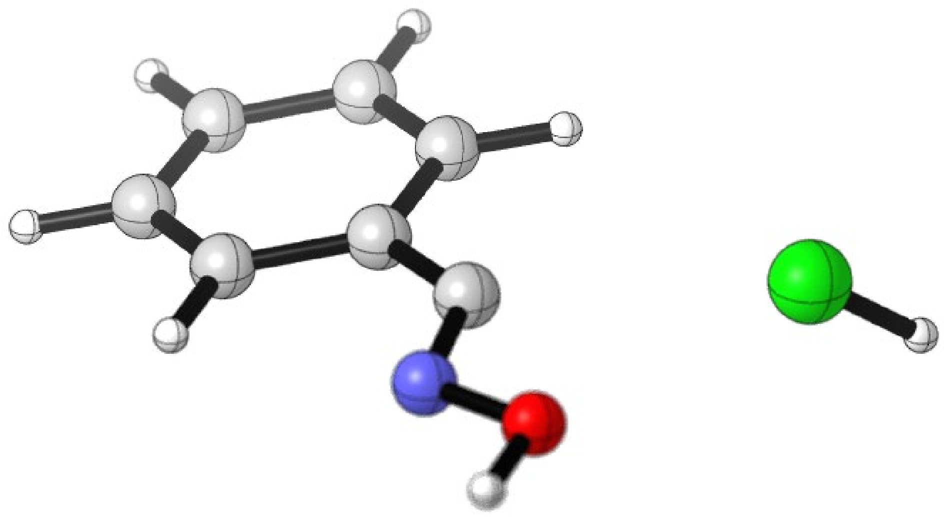

Sum of Electronic and Zero-point Energies = -860.375423 Hartree
Sum of Electronic and Thermal Energies = -860.364444 Hartree
Sum of Electronic and Thermal Enthalpies = -860.363500 Hartree
Sum of Electronic and Thermal Free Energies = -860.416143 Hartree

Dipole Moment = 3.8039 Debye

1 3

C	-2.55831	1.40201	1.27970
C	-1.20495	1.52471	1.07822
C	-0.53299	0.56563	0.24879
C	-1.25821	-0.50397	-0.35901
C	-2.61275	-0.60770	-0.14231
C	-3.26102	0.34122	0.67277
H	-3.08785	2.11510	1.90131
H	-0.63335	2.32911	1.52895
H	-0.72097	-1.21617	-0.97763

H	-3.18258	-1.41178	-0.59364
H	-4.33059	0.25335	0.83832
N	1.66815	1.53950	0.49005
O	2.91318	1.39180	0.09986
H	3.42261	2.10877	0.52407
C	0.84198	0.66257	0.00983
H	2.60495	-3.08387	-1.04186
Cl	2.17207	-1.88910	-1.22505

oxime-H-atom-abstraction-Cl-R-from-IRC

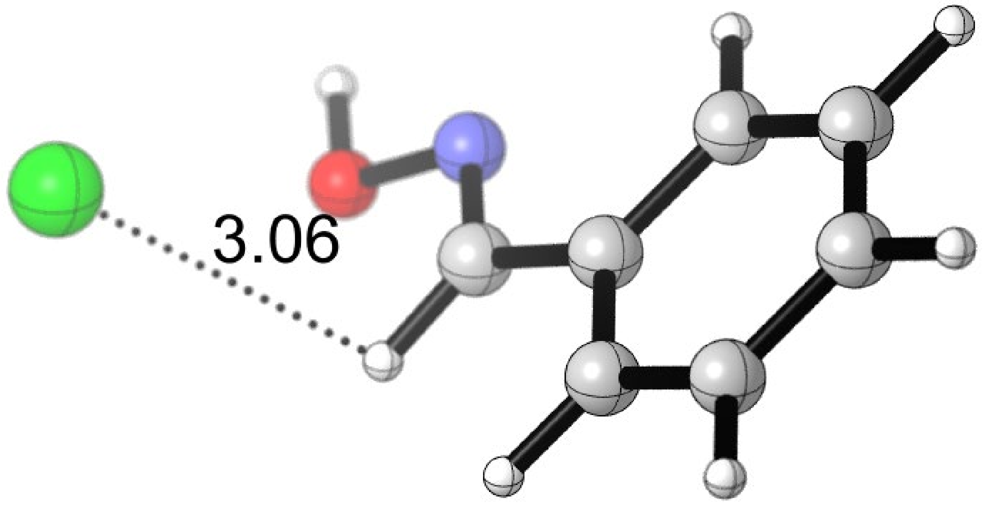

Sum of Electronic and Zero-point Energies = -860.706656 Hartree
Sum of Electronic and Thermal Energies = -860.696797 Hartree
Sum of Electronic and Thermal Enthalpies = -860.695853 Hartree
Sum of Electronic and Thermal Free Energies = -860.745009 Hartree

Dipole Moment = 2.4026 Debye

0 2

C	-2.53216	1.53420	1.05349
C	-1.15899	1.37139	0.92444
C	-0.63800	0.14434	0.49024
C	-1.50881	-0.90807	0.19192
C	-2.88582	-0.74113	0.32232
C	-3.39877	0.47970	0.75313
H	-2.93242	2.48529	1.38980
H	-0.47808	2.18397	1.15550
H	-1.10468	-1.85880	-0.14559

H	-3.55481	-1.56258	0.08752
H	-4.47108	0.61258	0.85591
N	1.64293	0.86216	0.55188
O	2.94517	0.40534	0.38168
H	3.48547	1.20695	0.40795
C	0.80551	-0.07468	0.33694
H	1.15914	-1.06030	0.02275
Cl	3.42786	-0.44509	-1.93760

oxime-H-atom-abstraction-Cl-R-iii

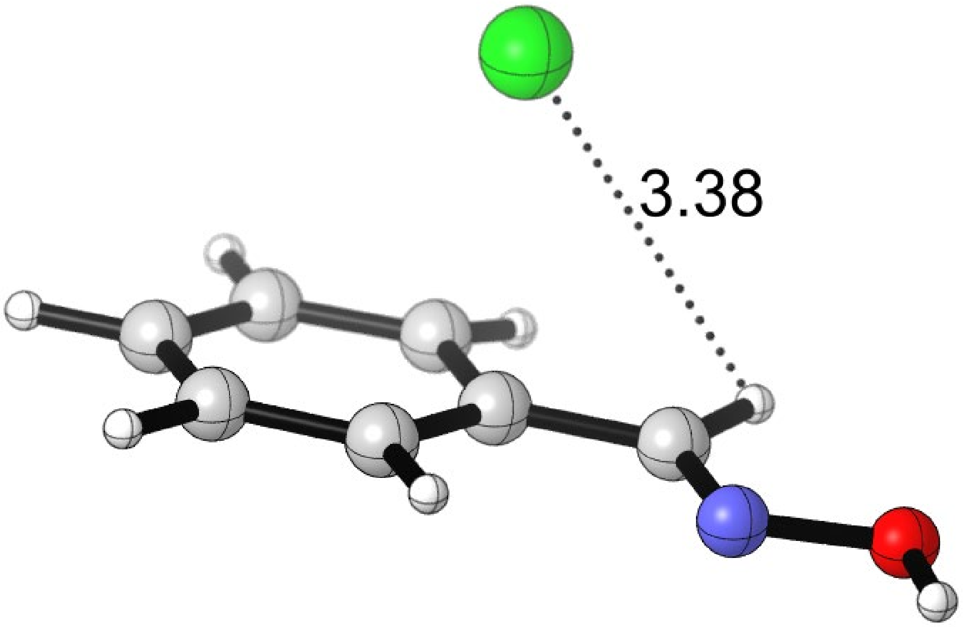

Sum of Electronic and Zero-point Energies = -860.710807 Hartree
Sum of Electronic and Thermal Energies = -860.700910 Hartree
Sum of Electronic and Thermal Enthalpies = -860.699966 Hartree
Sum of Electronic and Thermal Free Energies = -860.748110 Hartree

Dipole Moment = 3.6628 Debye

0 2

C	-1.80351	1.06354	0.14242
C	-0.41445	1.03751	0.12300
C	0.25516	-0.13309	-0.24129
C	-0.49949	-1.27233	-0.62457
C	-1.91196	-1.23783	-0.56439
C	-2.55651	-0.07421	-0.19640
H	-2.31536	1.97870	0.42323
H	0.16307	1.91647	0.38683
H	0.00885	-2.21986	-0.77020

H	-2.47338	-2.12405	-0.83906
H	-3.64005	-0.03382	-0.16995
N	2.43044	0.74263	0.14909
O	3.77209	0.46298	0.03327
H	4.20804	1.25587	0.36781
C	1.71595	-0.22586	-0.27503
H	2.18250	-1.12937	-0.67285
Cl	-0.22351	-0.93913	-3.04253

oxime-H-atom-abstraction-Cl-R

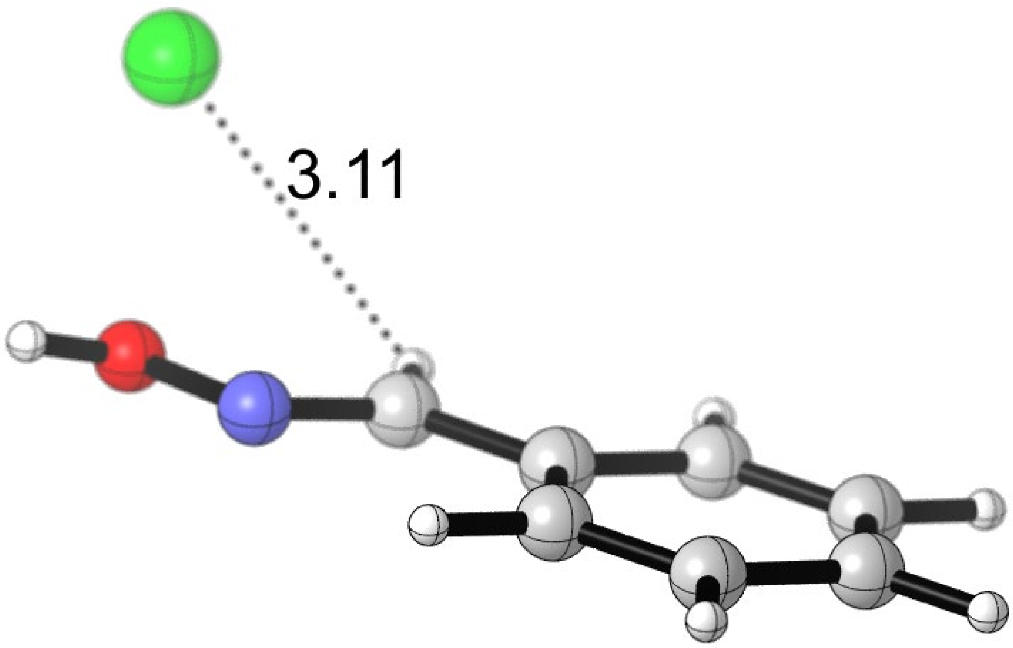

Sum of Electronic and Zero-point Energies = -860.706656 Hartree
Sum of Electronic and Thermal Energies = -860.696788 Hartree
Sum of Electronic and Thermal Enthalpies = -860.695844 Hartree
Sum of Electronic and Thermal Free Energies = -860.745195 Hartree

Dipole Moment = 2.4236 Debye

0 2

C	-1.86618	1.06364	0.83664
C	-0.52493	0.97919	0.48650
C	-0.09547	-0.01660	-0.40214
C	-1.02501	-0.91793	-0.92969
C	-2.36988	-0.82998	-0.57655
C	-2.79173	0.16054	0.30663
H	-2.19536	1.83501	1.52548
H	0.20124	1.67526	0.89323
H	-0.69163	-1.69068	-1.61732

H	-3.08472	-1.53384	-0.99014
H	-3.83875	0.23127	0.58379
N	2.20364	0.63895	-0.33767
O	3.44755	0.34494	-0.88506
H	4.06644	0.88623	-0.37597
C	1.31165	-0.14564	-0.80034
H	1.59141	-0.93712	-1.50073
Cl	4.27303	-1.96049	-0.30548

oxime-H-atom-abstraction-Cl-TS

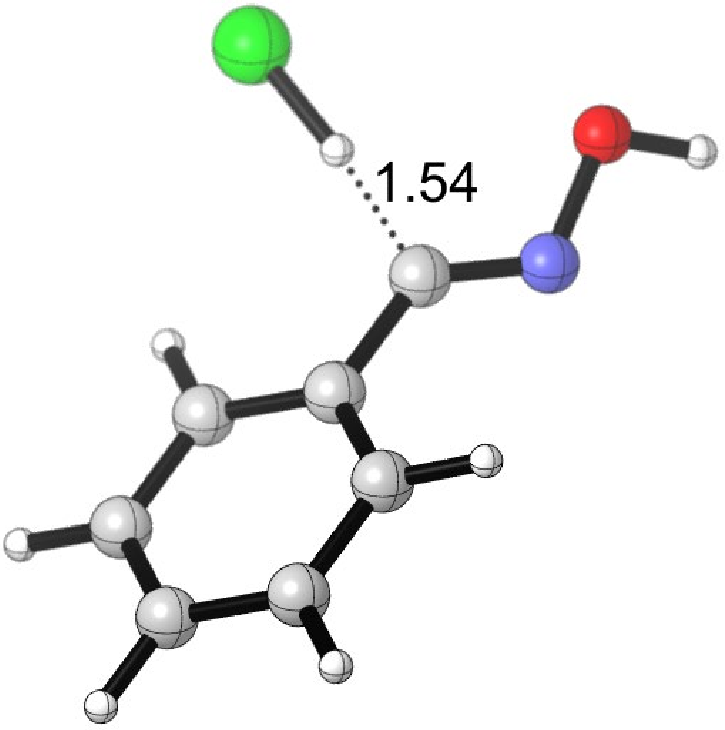

Sum of Electronic and Zero-point Energies = -860.697162 Hartree
Sum of Electronic and Thermal Energies = -860.687492 Hartree
Sum of Electronic and Thermal Enthalpies = -860.686548 Hartree
Sum of Electronic and Thermal Free Energies = -860.734997 Hartree

Dipole Moment = 4.4804 Debye

0 2

C	-1.98709	1.18199	0.44346
C	-0.60765	1.17884	0.27833
C	0.03063	0.02307	-0.19887
C	-0.72043	-1.11012	-0.53491
C	-2.10106	-1.09623	-0.36444
C	-2.73388	0.04616	0.12416
H	-2.48246	2.07189	0.81794
H	-0.01260	2.05395	0.51781
H	-0.21873	-1.98837	-0.92982

H	-2.68355	-1.97521	-0.61926
H	-3.81187	0.05552	0.25013
N	2.37309	0.57976	0.26325
O	3.65863	0.52245	-0.22546
H	4.19376	0.91529	0.47675
C	1.47428	0.00476	-0.37196
H	1.96907	-0.89671	-1.51943
Cl	2.31163	-1.78427	-2.55049

oxime-H-atom-abstraction-Cl-TS_triplet

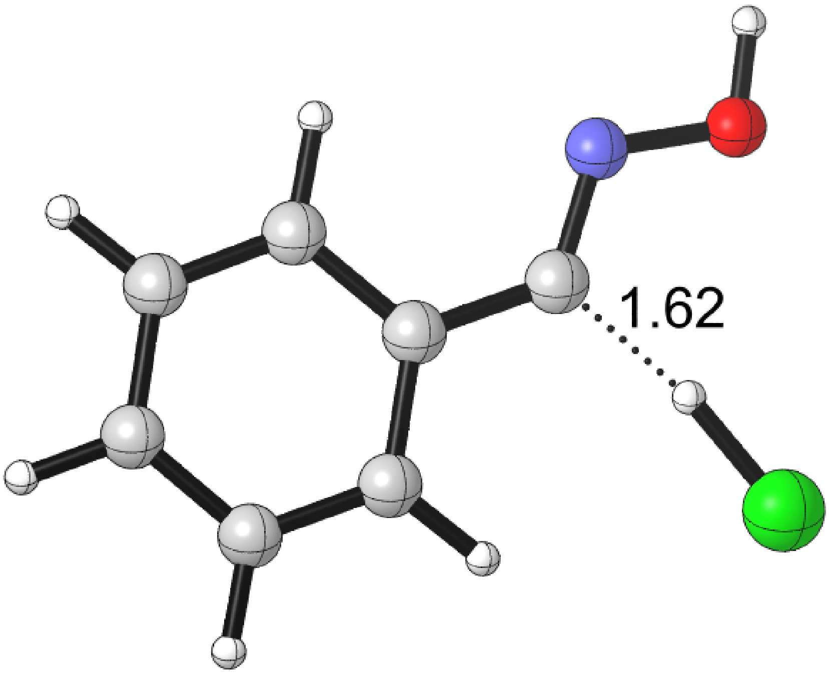

Sum of Electronic and Zero-point Energies = -860.365201 Hartree
Sum of Electronic and Thermal Energies = -860.355461 Hartree
Sum of Electronic and Thermal Enthalpies = -860.354517 Hartree
Sum of Electronic and Thermal Free Energies = -860.403346 Hartree

Dipole Moment = 3.3037 Debye

1 3

C	-1.92877	1.13082	0.63690
C	-0.56792	1.11994	0.46923
C	0.04215	0.02593	-0.23880
C	-0.75832	-1.03800	-0.76242
C	-2.11970	-1.00575	-0.58236
C	-2.70615	0.07321	0.11435
H	-2.41090	1.94447	1.16705
H	0.06012	1.91497	0.85690
H	-0.27547	-1.85331	-1.29312

H	-2.74484	-1.80165	-0.97085
H	-3.78315	0.09080	0.25233
N	2.29144	0.86470	-0.01744
O	3.54743	0.62393	-0.30511
H	4.07078	1.36247	0.06189
C	1.43416	-0.01039	-0.42481
H	2.07934	-1.26415	-1.21981
Cl	2.42158	-2.39524	-1.88674

Ph-oximyl-radical-triplet

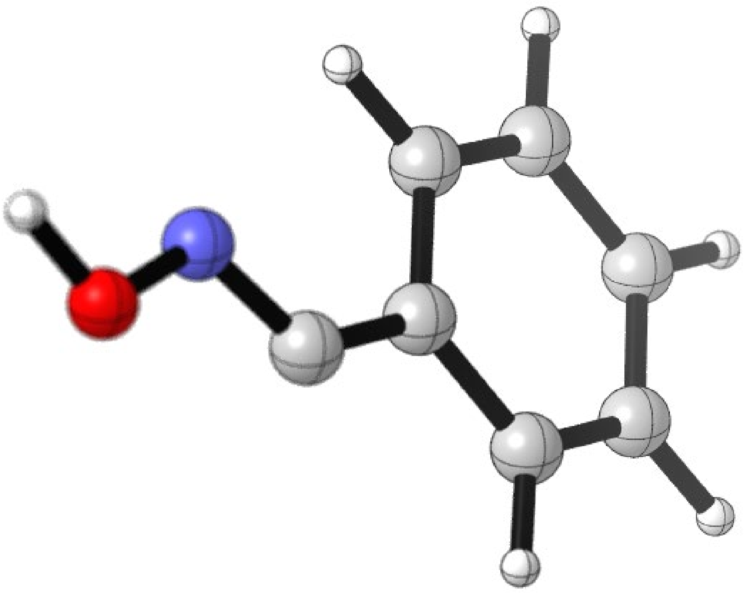

Sum of Electronic and Zero-point Energies = -399.614277 Hartree
Sum of Electronic and Thermal Energies = -399.606711 Hartree
Sum of Electronic and Thermal Enthalpies = -399.605767 Hartree
Sum of Electronic and Thermal Free Energies = -399.647665 Hartree

Dipole Moment = 1.0614 Debye

1 3

C	-2.18483	1.23651	-0.11377
C	-0.82029	1.40508	-0.11843
C	0.02768	0.26198	0.00303
C	-0.53461	-1.05320	0.12909
C	-1.90038	-1.19856	0.13142
C	-2.72407	-0.06043	0.01067
H	-2.84617	2.09053	-0.20477
H	-0.36928	2.38767	-0.21236
H	0.12865	-1.90684	0.22008

H	-2.34791	-2.18154	0.22542
H	-3.80264	-0.18619	0.01359
C	1.41436	0.45182	-0.00352
N	2.33482	-0.45320	0.09473
O	3.56870	-0.00605	0.06120
H	4.14926	-0.78704	0.14395

p-CF3-TS-endo

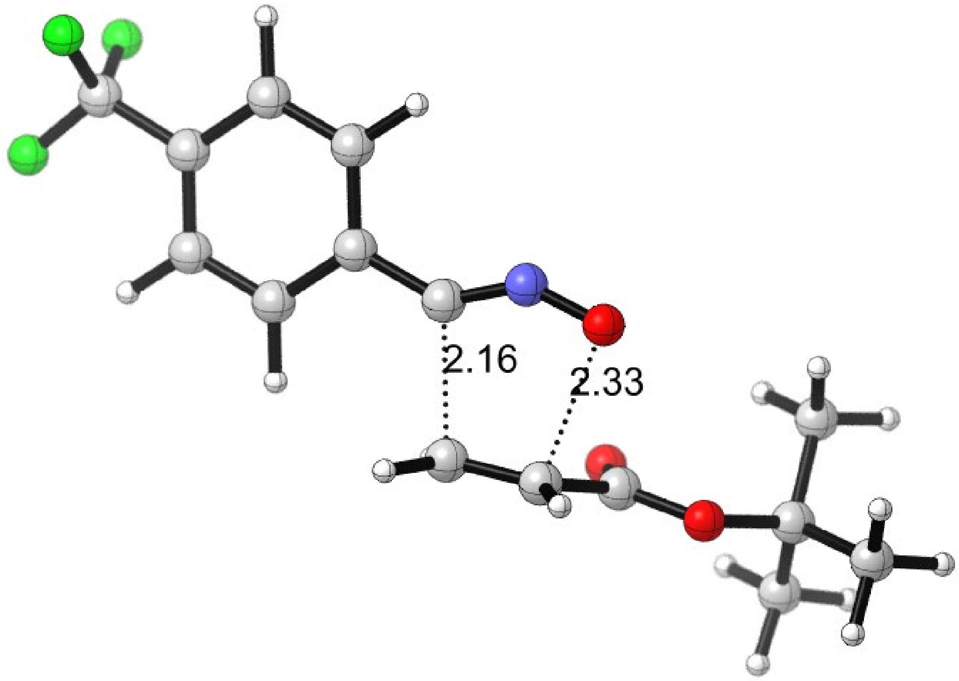

Sum of Electronic and Zero-point Energies = -1160.378281 Hartree
Sum of Electronic and Thermal Energies = -1160.356774 Hartree
Sum of Electronic and Thermal Enthalpies = -1160.355830 Hartree
Sum of Electronic and Thermal Free Energies = -1160.432554 Hartree

Dipole Moment = 1.3481 Debye

0 1

O	2.37581	2.46748	-0.66045
N	2.02840	1.37391	-0.25055
C	2.38497	0.42412	0.39509
C	1.91828	-0.86848	0.85126
C	2.74825	-1.69652	1.61086
C	0.61574	-1.28747	0.52820
C	2.28561	-2.93511	2.04806
H	3.75381	-1.37835	1.85960
C	0.16030	-2.52056	0.96499
H	-0.02268	-0.64009	-0.06291
C	0.99659	-3.34141	1.72541

H	2.92728	-3.57995	2.63762
H	-0.84455	-2.84932	0.71850
C	4.98251	2.33256	-1.10671
O	5.18891	1.34434	-1.78037
O	5.15065	3.59662	-1.50766
C	5.49987	3.90422	-2.89056
C	4.41498	3.37577	-3.82587
H	4.59722	3.75596	-4.83575
H	4.41122	2.28557	-3.85453
H	3.43340	3.72676	-3.49387
C	5.52322	5.42847	-2.90252
H	6.26584	5.80352	-2.19279
H	5.77951	5.78905	-3.90253
H	4.54305	5.82550	-2.62499
C	6.87975	3.34247	-3.22362
H	7.18512	3.70567	-4.20981
H	7.61324	3.68902	-2.48914
H	6.87162	2.25224	-3.23528
C	4.37724	1.06696	0.91291
H	4.80688	0.20133	0.41273
H	4.22066	1.01981	1.98654
C	4.49210	2.28063	0.29503
H	4.33214	3.22091	0.80835
C	0.46805	-4.67338	2.18098
F	-0.65342	-4.53414	2.91174
F	0.14926	-5.46142	1.13796
F	1.35400	-5.34402	2.93203

p-Cl-TS-endo

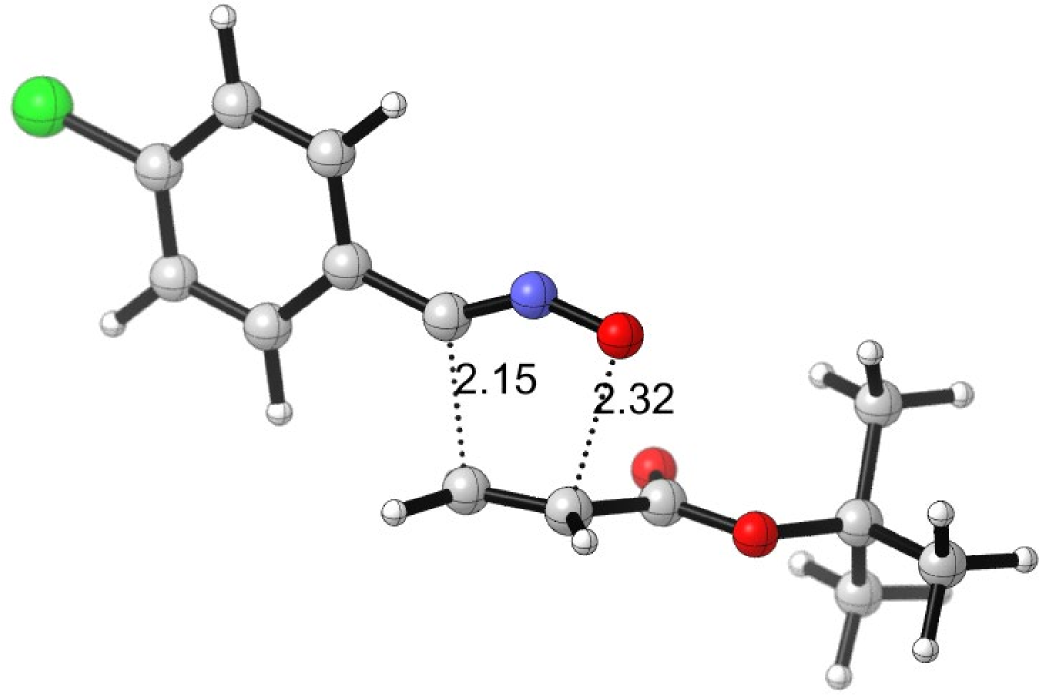

Sum of Electronic and Zero-point Energies = -1283.007261 Hartree
Sum of Electronic and Thermal Energies = -1282.988172 Hartree
Sum of Electronic and Thermal Enthalpies = -1282.987228 Hartree
Sum of Electronic and Thermal Free Energies = -1283.057426 Hartree

Dipole Moment = 1.5599 Debye

0 1

O	2.38092900	2.45992200	-0.67916200
N	2.02852500	1.36580000	-0.26901700
C	2.38224800	0.41601400	0.37750700
C	1.91417100	-0.87122300	0.84674900
C	2.75838800	-1.71852200	1.57072100
C	0.59674200	-1.27269700	0.57558600
C	2.29828400	-2.95217400	2.01955100
H	3.77835300	-1.41955300	1.78319000
C	0.13489700	-2.50298200	1.02130100
H	-0.05798400	-0.61553100	0.01329500
C	0.99041700	-3.33470700	1.74185300
H	2.94933100	-3.61373200	2.57966500
H	-0.88118600	-2.81960300	0.81418900
C	4.98501600	2.33017400	-1.12027100
O	5.20343300	1.34595900	-1.79615600
O	5.14658000	3.59696800	-1.51755300
C	5.49991900	3.90974700	-2.89758400
C	4.42511500	3.37343100	-3.84018600
H	4.60867200	3.75861000	-4.84798500
H	4.43218700	2.28336600	-3.87190400
H	3.43848200	3.71344100	-3.51179800

C	5.50951000	5.43424500	-2.90730700
H	6.24426700	5.81492600	-2.19236800
H	5.76861100	5.79876300	-3.90520700
H	4.52405300	5.82175600	-2.63507400
C	6.88673500	3.36136200	-3.22444300
H	7.19365100	3.72795500	-4.20893400
H	7.61321900	3.71425000	-2.48599500
H	6.88868100	2.27109700	-3.23653500
C	4.37265800	1.05459700	0.89083200
H	4.80545900	0.19248000	0.38749500
H	4.21591400	1.00184900	1.96418200
C	4.48765300	2.27141800	0.27809800
H	4.32545400	3.20933900	0.79489000
Cl	0.41142800	-4.87605400	2.30195900

p-CN-TS-endo

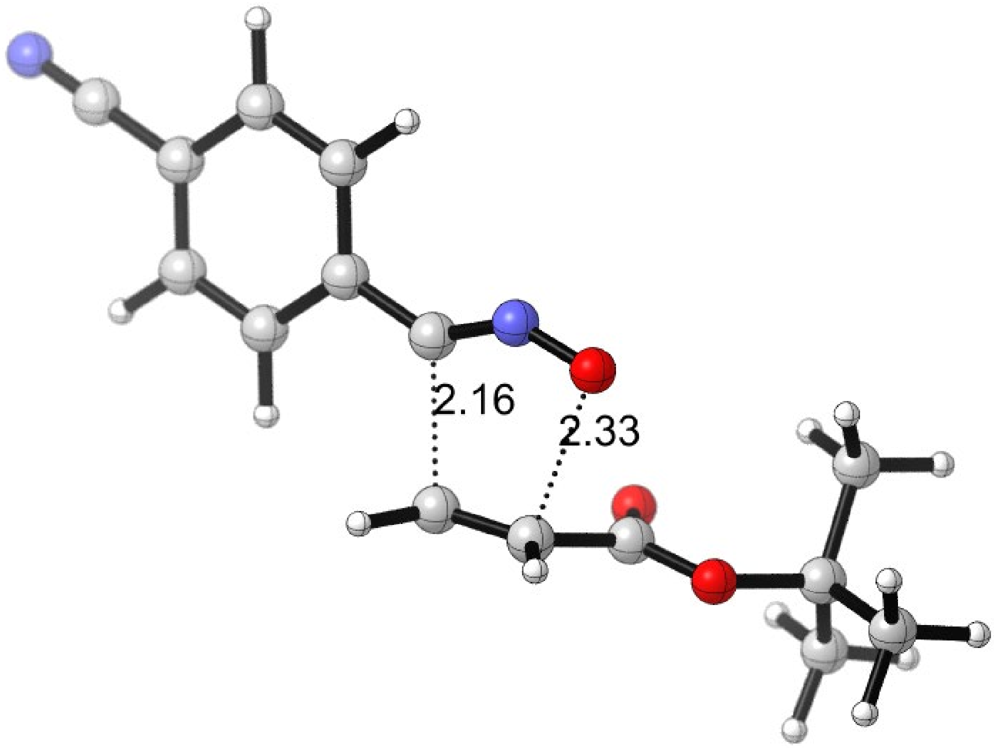

Sum of Electronic and Zero-point Energies = -915.645171 Hartree
Sum of Electronic and Thermal Energies = -915.625473 Hartree
Sum of Electronic and Thermal Enthalpies = -915.624529 Hartree
Sum of Electronic and Thermal Free Energies = -915.695601 Hartree

Dipole Moment = 2.6592 Debye

0 1

O	2.38195	2.48636	-0.61512
N	2.03095	1.39217	-0.21264
C	2.38827	0.43071	0.41562
C	1.91633	-0.86375	0.85764

C	2.74833	-1.70760	1.60173
C	0.61062	-1.27165	0.53747
C	2.28471	-2.94695	2.02368
H	3.75700	-1.39760	1.84804
C	0.14794	-2.50809	0.95799
H	-0.02821	-0.61332	-0.04092
C	0.98462	-3.34921	1.70359
H	2.92551	-3.60532	2.59959
H	-0.85850	-2.82954	0.71340
C	4.98071	2.32138	-1.11057
O	5.15861	1.33518	-1.79519
O	5.16033	3.58557	-1.50436
C	5.49085	3.89956	-2.89087
C	4.38242	3.39522	-3.81170
H	4.55313	3.78147	-4.82126
H	4.36203	2.30551	-3.84978
H	3.41192	3.75789	-3.46018
C	5.53714	5.42327	-2.89068
H	6.29729	5.78115	-2.19073
H	5.78198	5.78828	-3.89194
H	4.56797	5.83278	-2.59340
C	6.85617	3.31966	-3.25148
H	7.15036	3.68617	-4.23980
H	7.60715	3.64916	-2.52692
H	6.83132	2.22980	-3.27174
C	4.39757	1.04792	0.91023
H	4.80810	0.18159	0.39539
H	4.25817	0.99396	1.98592
C	4.51576	2.26496	0.30012
H	4.37656	3.20301	0.82350
C	0.50275	-4.63336	2.14171
N	0.11443	-5.66507	2.49452

p-F-TS-endo

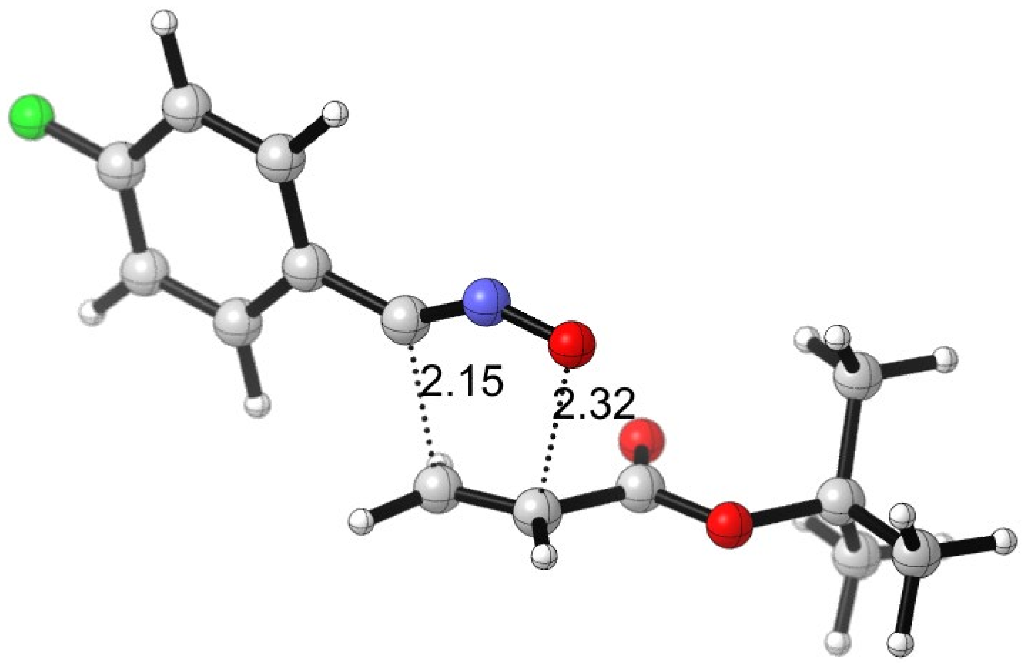


```
Sum of Electronic and Zero-point Energies = -922.648921 Hartree
Sum of Electronic and Thermal Energies = -922.630204 Hartree
Sum of Electronic and Thermal Enthalpies = -922.629260 Hartree
Sum of Electronic and Thermal Free Energies = -922.698274 Hartree

Dipole Moment = 1.7175 Debye

0 1
O                2.39260      2.46400     -0.66566
N                2.03394      1.36843     -0.26217
C                2.38426      0.41142      0.37499
C                1.90979     -0.87499      0.84169
C                2.75920     -1.73763      1.54261
C                0.58211     -1.25800      0.59023
C                2.29556     -2.97087      1.98975
H                3.78564     -1.44954      1.73770
C                0.11272     -2.48716      1.03197
H               -0.07327     -0.58651      0.04607
C                0.98119     -3.32023      1.72502
H                2.93539     -3.65652      2.53303
H               -0.90692     -2.80649      0.84923
C                4.99073      2.32351     -1.12336
O                5.20116      1.34471     -1.80964
O                5.15708      3.59326     -1.50980
C                5.50400      3.91701     -2.88871
C                4.42081      3.39547     -3.83001
H                4.60059      3.78911     -4.83523
H                4.42173      2.30573     -3.87196
H                3.43799      3.73762     -3.49254
```

C	5.52188	5.44149	-2.88426
H	6.26305	5.81139	-2.17027
H	5.77688	5.81402	-3.88026
H	4.54024	5.83169	-2.60227
C	6.88591	3.36440	-3.22915
H	7.18901	3.73887	-4.21187
H	7.61863	3.70620	-2.49163
H	6.88184	2.27432	-3.25167
C	4.38157	1.03251	0.87883
H	4.80705	0.17311	0.36478
H	4.23193	0.97050	1.95270
C	4.49980	2.25449	0.27659
H	4.34647	3.18845	0.80314
F	0.52784	-4.51017	2.15462

p-H-TS-endo-ii

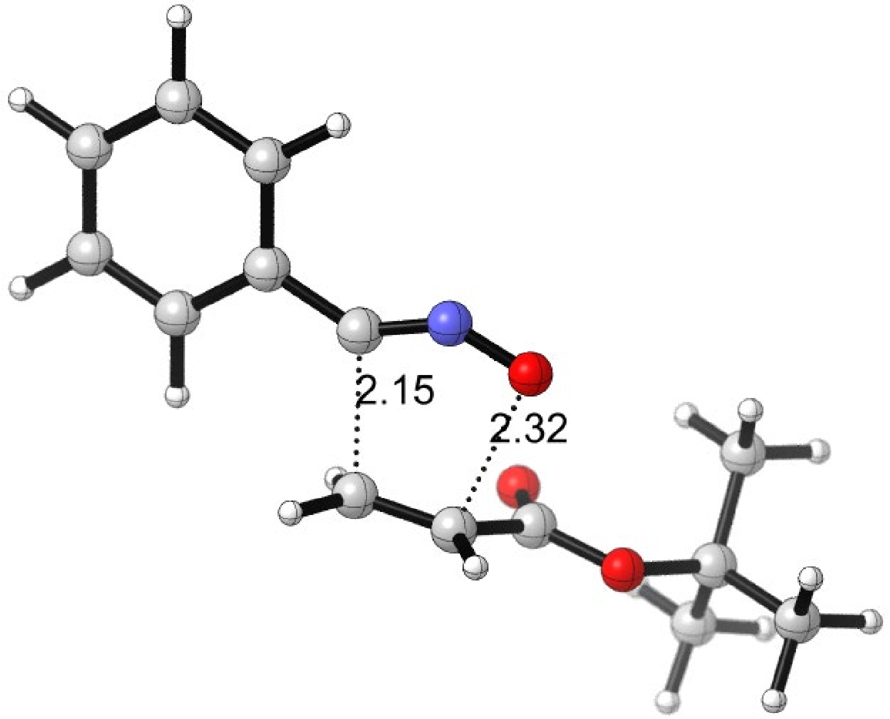

Sum of Electronic and Zero-point Energies = -823.429974 Hartree
Sum of Electronic and Thermal Energies = -823.412093 Hartree
Sum of Electronic and Thermal Enthalpies = -823.411149 Hartree
Sum of Electronic and Thermal Free Energies = -823.477978 Hartree

Dipole Moment = 3.2054 Debye

0 1

O	2.39843	2.46197	-0.66641
N	2.03683	1.36793	-0.26105
C	2.38731	0.41029	0.37583

C	1.91122	-0.87589	0.84425
C	2.75566	-1.73319	1.55668
C	0.58670	-1.26004	0.58093
C	2.27969	-2.96413	2.00059
H	3.77980	-1.44124	1.75940
C	0.12297	-2.49071	1.02780
H	-0.06214	-0.58962	0.02716
C	0.96630	-3.34611	1.73874
H	2.94057	-3.62538	2.55120
H	-0.90114	-2.78327	0.82008
H	0.59947	-4.30656	2.08561
C	4.99514	2.32042	-1.12648
O	5.20606	1.34418	-1.81622
O	5.16064	3.59178	-1.51010
C	5.50345	3.91880	-2.88877
C	4.41811	3.39891	-3.82861
H	4.59490	3.79518	-4.83337
H	4.41961	2.30928	-3.87310
H	3.43598	3.73939	-3.48747
C	5.52032	5.44335	-2.88102
H	6.26322	5.81210	-2.16822
H	5.77220	5.81840	-3.87690
H	4.53919	5.83211	-2.59525
C	6.88488	3.36823	-3.23476
H	7.18489	3.74550	-4.21739
H	7.61939	3.70864	-2.49836
H	6.88150	2.27820	-3.25991
C	4.38431	1.02489	0.87295
H	4.80578	0.16443	0.35750
H	4.23642	0.96129	1.94692
C	4.50558	2.24799	0.27332
H	4.35476	3.18101	0.80226

p-H-TS-endo-regio-isomer

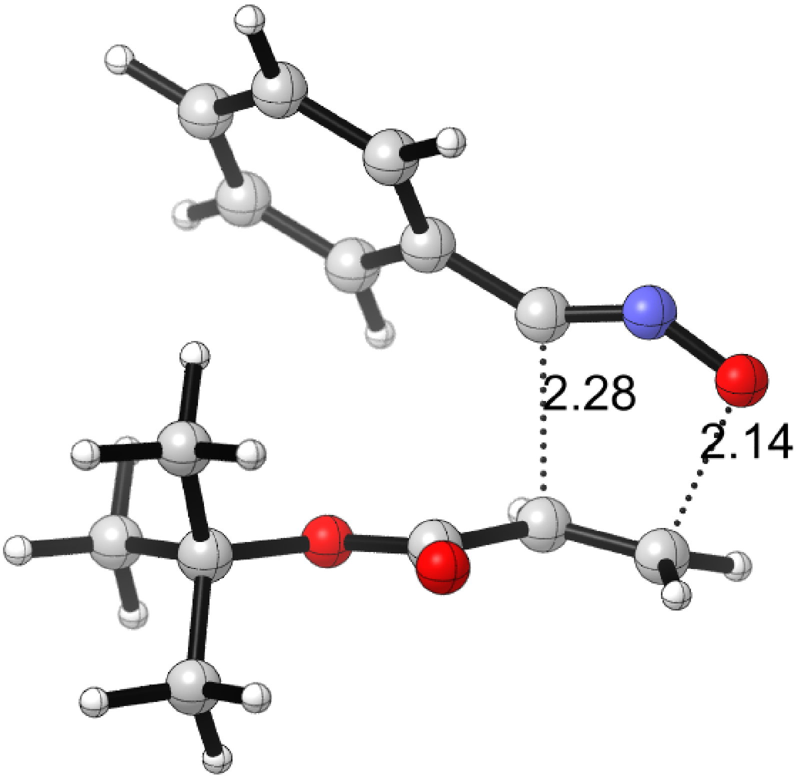


```
Sum of Electronic and Zero-point Energies = -823.431540 Hartree
Sum of Electronic and Thermal Energies = -823.413995 Hartree
Sum of Electronic and Thermal Enthalpies = -823.413051 Hartree
Sum of Electronic and Thermal Free Energies = -823.476556 Hartree

Dipole Moment = 4.8372 Debye

0 1
O                3.81849      1.65063     -2.25728
N                3.37683      0.51068     -2.39209
C                3.51020     -0.60983     -1.99016
C                2.95926     -1.94604     -1.98653
C                3.80836     -3.05699     -1.92859
C                1.56808     -2.12241     -2.01318
C                3.26670     -4.33843     -1.91812
H                4.88178     -2.90926     -1.87611
C                1.03744     -3.40761     -2.00507
H                0.92102     -1.25194     -2.03858
C                1.88394     -4.51613     -1.95795
H                3.92585     -5.19957     -1.87584
H               -0.03871     -3.54390     -2.02932
H                1.46546     -5.51741     -1.94757
C                4.42163     -0.60135      0.61223
O                3.62106      0.04999      1.24883
O                4.72373     -1.89086      0.84634
C                4.05288     -2.64092      1.90214
C                2.55593     -2.72929      1.61390
H                2.08727     -3.37957      2.35940
H                2.08498     -1.74628      1.65922
```

H	2.38741	-3.16635	0.62407
C	4.69677	-4.01917	1.79839
H	5.77547	-3.95045	1.96498
H	4.26664	-4.68968	2.54765
H	4.52000	-4.44340	0.80546
C	4.34554	-2.01384	3.26332
H	3.98107	-2.68123	4.05041
H	5.42492	-1.88868	3.39336
H	3.85829	-1.04418	3.36721
C	5.28172	1.26594	-0.74591
H	4.75853	1.93509	-0.07147
H	6.04813	1.68534	-1.38548
C	5.19572	-0.08915	-0.54652
H	5.91108	-0.76893	-0.99823

p-Me-TS-endo

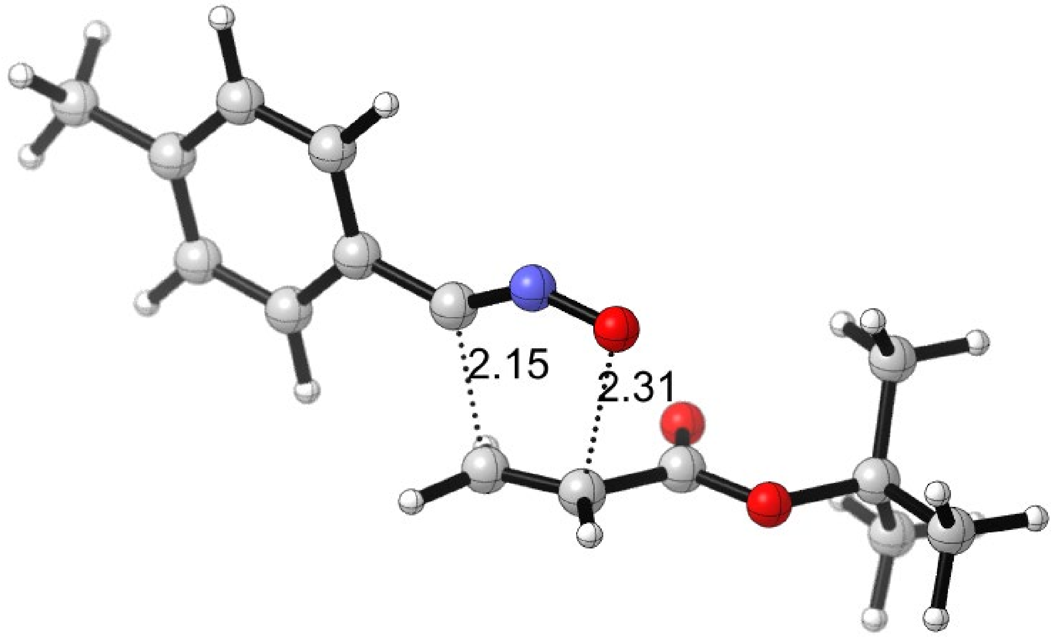

Sum of Electronic and Zero-point Energies = -862.702224 Hartree
Sum of Electronic and Thermal Energies = -862.682388 Hartree
Sum of Electronic and Thermal Enthalpies = -862.681444 Hartree
Sum of Electronic and Thermal Free Energies = -862.754466 Hartree

Dipole Moment = 3.8478 Debye

0 1

O	2.39435200	2.45974800	-0.68807700
N	2.03309400	1.36558900	-0.27967400
C	2.38185300	0.41433600	0.36753100

C	1.91198900	-0.87195700	0.83917200
C	2.74762300	-1.71869400	1.57019300
C	0.59505300	-1.27566500	0.56252600
C	2.27346100	-2.95023900	2.01603300
H	3.76703300	-1.42127500	1.78897300
C	0.13968500	-2.50499500	1.01305400
H	-0.05555900	-0.61892700	-0.00564700
C	0.96858500	-3.36454800	1.74743200
H	2.93500100	-3.59962800	2.58236400
H	-0.88072400	-2.80743700	0.79175800
C	4.99388900	2.32850900	-1.11970700
O	5.21939700	1.34937700	-1.80077500
O	5.15502400	3.59857100	-1.51062100
C	5.50812100	3.91800300	-2.88815800
C	4.43528100	3.38360200	-3.83426400
H	4.61838400	3.77403100	-4.84019500
H	4.44502900	2.29371200	-3.87072800
H	3.44765400	3.71915800	-3.50434100
C	5.51393900	5.44268700	-2.89152900
H	6.24742000	5.82201800	-2.17450800
H	5.77244700	5.81235500	-3.88774300
H	4.52738700	5.82630500	-2.61771500
C	6.89665000	3.37516700	-3.21770800
H	7.20288900	3.74745100	-4.20034600
H	7.62188000	3.72631700	-2.47715200
H	6.90136300	2.28499600	-3.23473100
C	4.36791800	1.04257400	0.88128500
H	4.79957000	0.18070800	0.37673600
H	4.20914200	0.98546400	1.95405700
C	4.48967200	2.26248800	0.27482200
H	4.32863300	3.19813700	0.79597100
C	0.45290400	-4.69736800	2.22406300
H	-0.42812500	-4.57018900	2.86039100
H	0.15964400	-5.32640000	1.37795200
H	1.21190000	-5.23345700	2.79775400

p-NO2-TS-endo

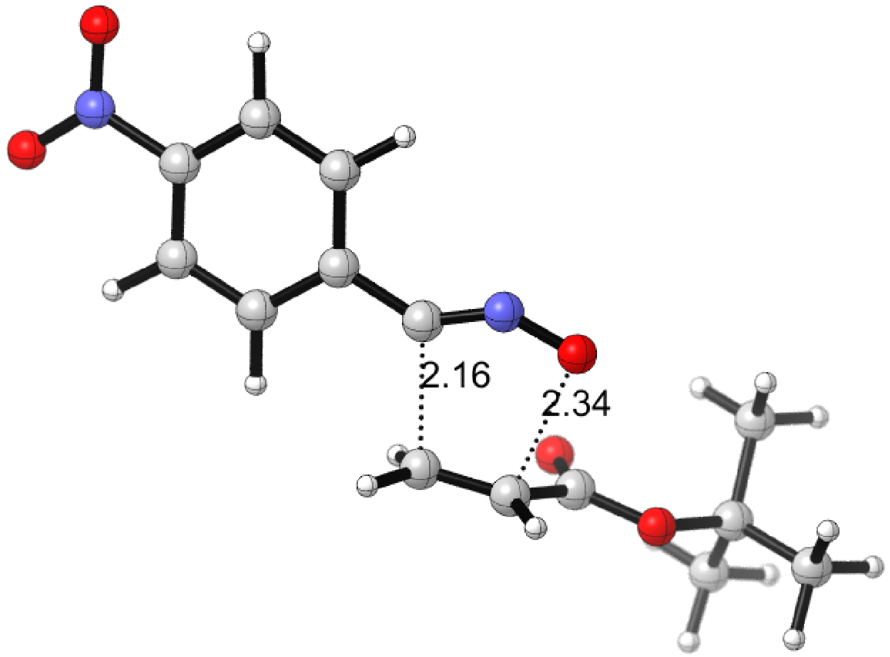


```
Sum of Electronic and Zero-point Energies = -1027.857974 Hartree
Sum of Electronic and Thermal Energies = -1027.837570 Hartree
Sum of Electronic and Thermal Enthalpies = -1027.836626 Hartree
Sum of Electronic and Thermal Free Energies = -1027.909818 Hartree

Dipole Moment = 3.0526 Debye

0 1
O                 2.37332      2.48156     -0.61042
N                 2.02602      1.38756     -0.20719
C                 2.38553      0.42730      0.42203
C                 1.91449     -0.86770      0.86266
C                 2.74855     -1.70919      1.60840
C                 0.60897     -1.27595      0.53913
C                 2.28873     -2.95068      2.03069
H                 3.75590     -1.39662      1.85600
C                 0.14479     -2.51354      0.95720
H                -0.02909     -0.61793     -0.04012
C                 0.99560     -3.32756      1.69716
H                 2.91330     -3.62158      2.60764
H                -0.85601     -2.85431      0.72131
C                 4.97479      2.31863     -1.10631
O                 5.14972      1.33077     -1.78924
O                 5.15567      3.58168     -1.50199
C                 5.48679      3.89335     -2.88922
C                 4.37724      3.38980     -3.80906
H                 4.54858      3.77422     -4.81920
H                 4.35475      2.30007     -3.84568
H                 3.40748      3.75500     -3.45813
```

C	5.53603	5.41692	-2.89086
H	6.29698	5.77421	-2.19149
H	5.78148	5.78014	-3.89260
H	4.56770	5.82878	-2.59407
C	6.85091	3.31018	-3.24890
H	7.14586	3.67488	-4.23764
H	7.60256	3.63908	-2.52478
H	6.82396	2.22034	-3.26796
C	4.39533	1.04903	0.91782
H	4.80637	0.18223	0.40411
H	4.25569	0.99678	1.99358
C	4.51169	2.26482	0.30542
H	4.37142	3.20377	0.82696
N	0.50406	-4.64320	2.14444
O	-0.63347	-4.95059	1.83823
O	1.26687	-5.33706	2.79172

p-OMe-TS-endo

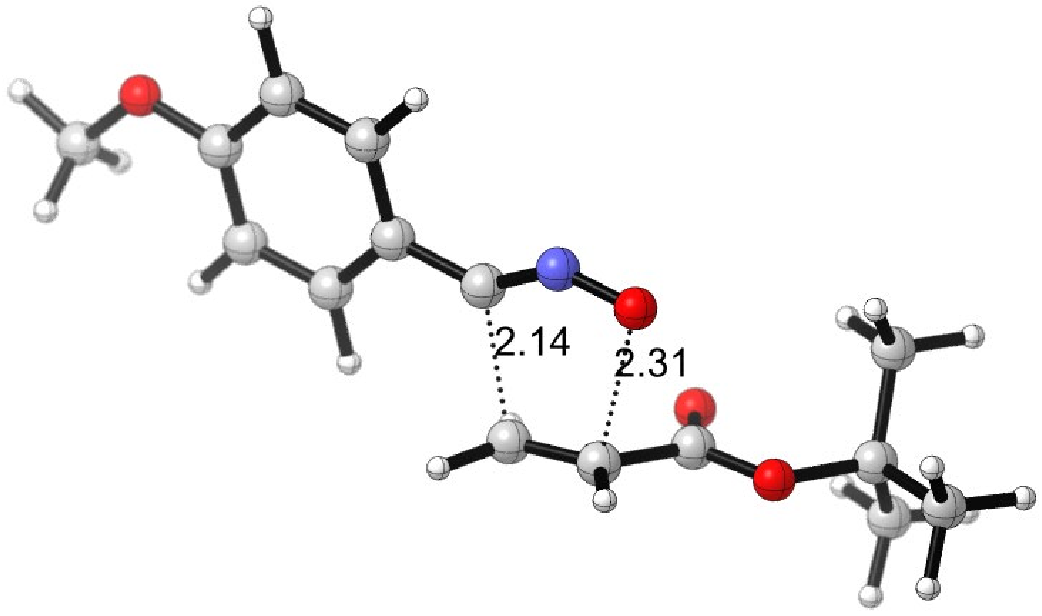

Sum of Electronic and Zero-point Energies = -937.880727 Hartree
Sum of Electronic and Thermal Energies = -937.860308 Hartree
Sum of Electronic and Thermal Enthalpies = -937.859364 Hartree
Sum of Electronic and Thermal Free Energies = -937.932326 Hartree

Dipole Moment = 4.4837 Debye

0 1

O	2.40848	2.43620	-0.73235

N	2.06065	1.33826	-0.31862
C	2.41796	0.40046	0.34308
C	1.96097	-0.87765	0.84419
C	2.82775	-1.74073	1.51281
C	0.61846	-1.26196	0.66238
C	2.38158	-2.97047	1.99584
H	3.86491	-1.46095	1.65977
C	0.17086	-2.47902	1.13652
H	-0.06126	-0.59398	0.14372
C	1.04955	-3.34329	1.80753
H	3.07974	-3.61991	2.50902
H	-0.85919	-2.79100	1.00274
C	5.01220	2.35748	-1.13534
O	5.27343	1.38105	-1.80763
O	5.14461	3.62923	-1.53193
C	5.50454	3.95007	-2.90718
C	4.45889	3.37929	-3.86248
H	4.64151	3.77036	-4.86826
H	4.50144	2.29004	-3.89329
H	3.45807	3.68657	-3.54502
C	5.46645	5.47427	-2.92060
H	6.17957	5.87924	-2.19708
H	5.72666	5.84499	-3.91599
H	4.46586	5.83083	-2.66156
C	6.91202	3.44566	-3.21683
H	7.21875	3.81948	-4.19874
H	7.61801	3.82326	-2.47070
H	6.94857	2.35599	-3.22546
C	4.38952	1.06577	0.86258
H	4.84551	0.21344	0.36341
H	4.22255	1.00671	1.93401
C	4.49165	2.28609	0.25273
H	4.30475	3.22004	0.76820
O	0.51352	-4.51110	2.23371
C	1.35920	-5.42101	2.91099
H	0.73750	-6.27985	3.15900
H	2.18734	-5.74139	2.26860
H	1.75677	-4.97850	3.83149

dimethyl-fumerate-TS-cycloaddition-ii

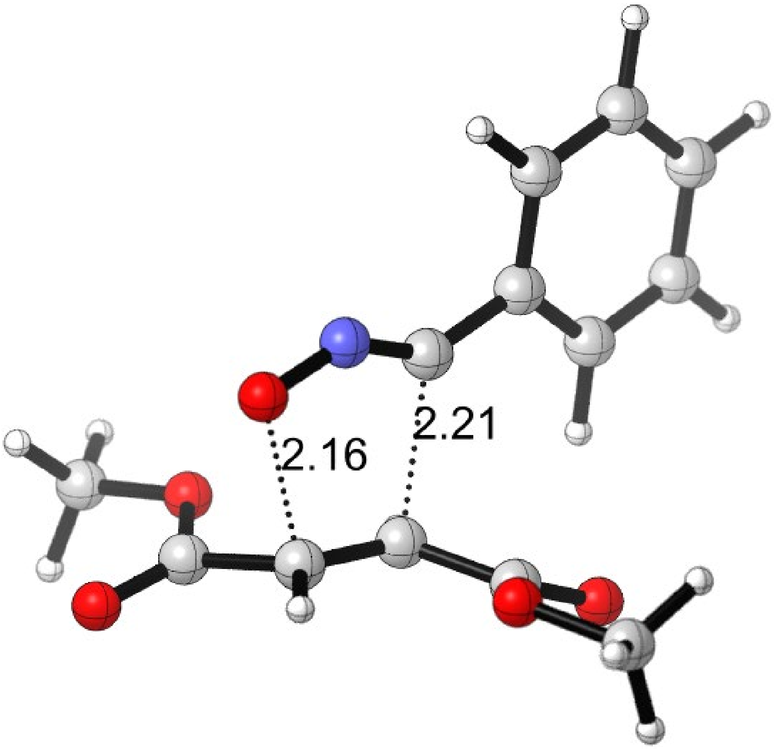

Sum of Electronic and Zero-point Energies = -933.372341 Hartree
Sum of Electronic and Thermal Energies = -933.353942 Hartree
Sum of Electronic and Thermal Enthalpies = -933.352998 Hartree
Sum of Electronic and Thermal Free Energies = -933.421790 Hartree

Dipole Moment = 4.5145 Debye

0 1

C	-0.75806	-1.47123	0.52235
H	-0.20889	-2.38820	0.33197
C	-0.10259	-0.26092	0.47349
H	-0.64231	0.66365	0.31110
C	1.36817	-0.17380	0.28976
C	-2.21850	-1.55063	0.26427
O	-2.78367	-2.55149	-0.12169
O	1.94549	0.81099	-0.10515
O	1.99008	-1.31923	0.62193
O	-2.85427	-0.39580	0.50930
C	-4.26334	-0.41097	0.26786
H	-4.61273	0.59013	0.51226
H	-4.46478	-0.64775	-0.77872
H	-4.74580	-1.15642	0.90325
C	3.41369	-1.28888	0.50248
H	3.75640	-2.27510	0.80983
H	3.70250	-1.08230	-0.53010
H	3.82749	-0.51452	1.15156
O	0.15771	0.21423	2.56526
N	-0.30824	-0.82485	3.01949
C	-0.81492	-1.86542	2.70022

C	-1.33018	-3.10474	3.24763
C	-1.72327	-4.15191	2.40826
C	-1.42096	-3.24349	4.64242
C	-2.20113	-5.33458	2.96772
H	-1.68000	-4.03645	1.33107
C	-1.89843	-4.42892	5.18527
H	-1.11404	-2.42307	5.28273
C	-2.28902	-5.47734	4.35006
H	-2.50912	-6.14416	2.31457
H	-1.96587	-4.53491	6.26307
H	-2.66296	-6.40150	4.77867

dimethyl-maleate-TS-cycloaddition-iii

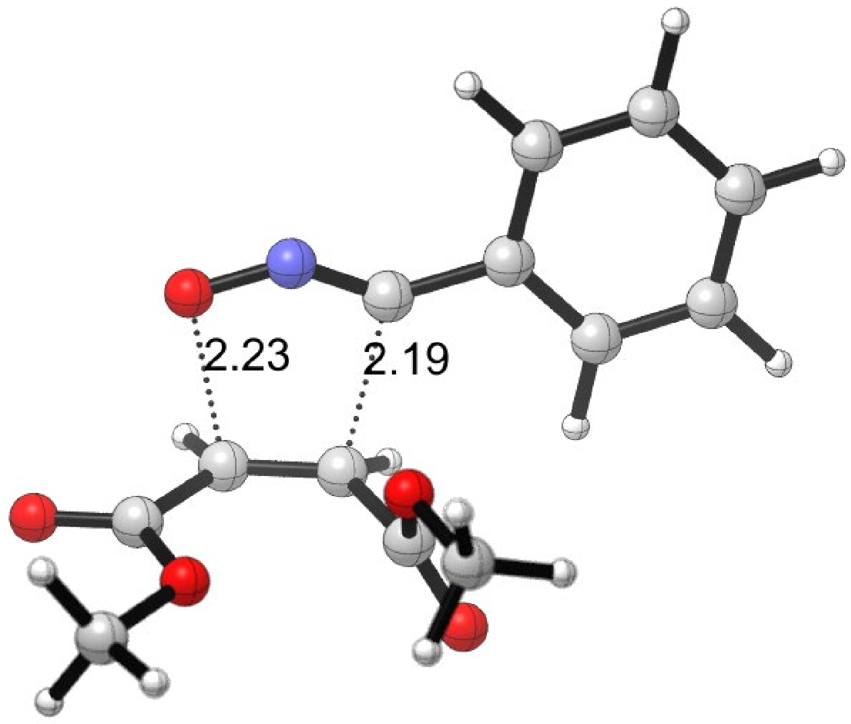

Sum of Electronic and Zero-point Energies = -933.360988 Hartree
Sum of Electronic and Thermal Energies = -933.342573 Hartree
Sum of Electronic and Thermal Enthalpies = -933.341629 Hartree
Sum of Electronic and Thermal Free Energies = -933.410185 Hartree

Dipole Moment = 5.5670 Debye

0 1

C	-0.38768100	-1.82014500	0.60815500
C	0.17066500	-0.62175500	0.22735000
H	-0.50867400	0.12571700	-0.17575700
C	1.57668500	-0.45732500	-0.27518800
O	1.82706600	-0.18567900	-1.42698100
O	2.50536200	-0.55847600	0.68031300

C	3.85527000	-0.48114700	0.22253500
H	4.47419000	-0.60863300	1.10850600
H	4.04761400	-1.27384300	-0.50444200
H	4.04245000	0.48804400	-0.24529800
H	-1.46728100	-1.89460500	0.67233200
C	0.26719200	-3.15328100	0.64905900
O	-0.32597600	-4.16893000	0.92611000
O	1.56471400	-3.12702700	0.30529500
C	2.22326200	-4.39441700	0.33719000
H	1.74868500	-5.08432900	-0.36348000
H	3.25396600	-4.19827400	0.04667200
H	2.17712700	-4.81645700	1.34298900
O	-0.27339500	-1.66407700	2.82494000
N	0.07186600	-0.49104700	2.84024900
C	0.35881400	0.43470500	2.13258200
C	0.77295900	1.82215300	2.15627200
C	1.07213400	2.50050700	0.96969300
C	0.87254100	2.48739300	3.38828800
C	1.46910000	3.83423900	1.01822300
H	0.99894800	1.99707500	0.01117400
C	1.27008100	3.81794900	3.42335700
H	0.63772700	1.95357100	4.30319200
C	1.56949400	4.49505300	2.24022600
H	1.70035900	4.35480300	0.09488500
H	1.34588400	4.32814400	4.37804200
H	1.87898700	5.53452100	2.27263600

p-CF3-TS-exo

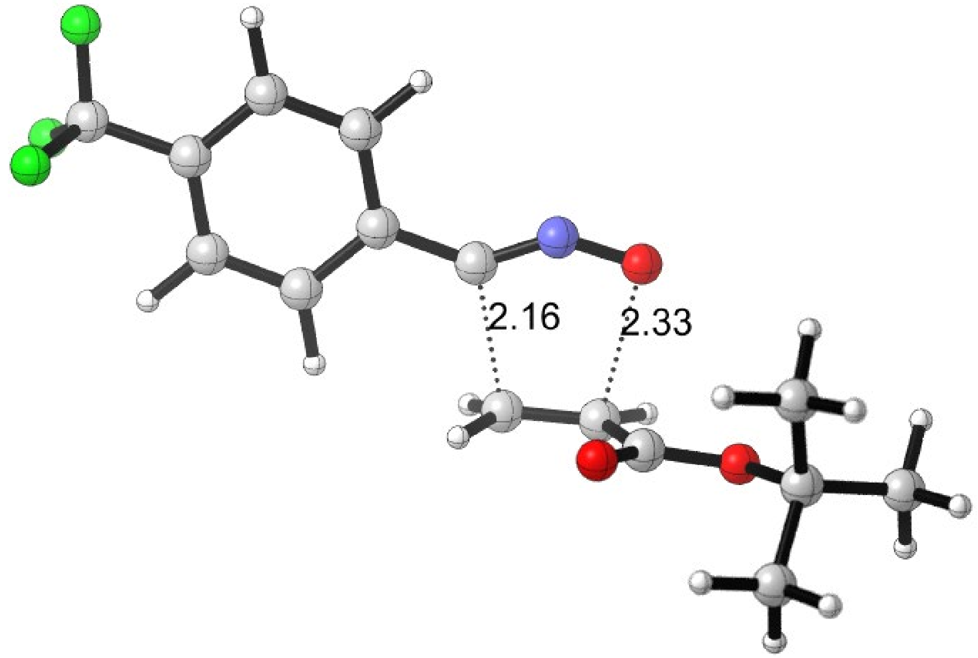

Sum of Electronic and Zero-point Energies = -1160.378102 Hartree
Sum of Electronic and Thermal Energies = -1160.356638 Hartree
Sum of Electronic and Thermal Enthalpies = -1160.355693 Hartree
Sum of Electronic and Thermal Free Energies = -1160.431787 Hartree

Dipole Moment = 1.3027 Debye

0 1

O	3.89239	1.56780	-0.34213
N	3.31743	0.79945	0.40869
C	3.40955	-0.26484	0.96083
C	2.67701	-1.13906	1.85329
C	3.24225	-2.34233	2.29297
C	1.38916	-0.77646	2.27396
C	2.52589	-3.17263	3.14467
H	4.23791	-2.62510	1.97215
C	0.67680	-1.61009	3.12677
H	0.95753	0.15704	1.92991
C	1.24666	-2.80534	3.55827
H	2.96034	-4.10598	3.48868
H	-0.31873	-1.33288	3.45469
C	6.48837	1.31436	0.12217
O	6.66436	1.40747	1.31939
O	6.90449	2.19219	-0.79607
C	7.53187	3.44914	-0.40190
C	8.85568	3.17739	0.30805
H	9.37799	4.12604	0.46608
H	8.69617	2.69671	1.27359
H	9.48975	2.53687	-0.31263
C	7.77144	4.14138	-1.73890
H	6.82337	4.29897	-2.26017
H	8.24964	5.11114	-1.57564
H	8.42131	3.53139	-2.37253
C	6.56602	4.26096	0.45789
H	6.97386	5.26624	0.60169
H	5.59945	4.34667	-0.04743
H	6.41661	3.79780	1.43378
C	5.35909	-0.86580	0.26140
H	5.01491	-1.78296	-0.20742
H	5.73458	-0.91362	1.28157
C	5.75890	0.18659	-0.51369
H	5.65431	0.19793	-1.59173
C	0.50316	-3.72662	4.48571
F	-0.72948	-3.28001	4.76841
F	1.15196	-3.87873	5.65476
F	0.37274	-4.95768	3.95807

p-Cl-TS-exo

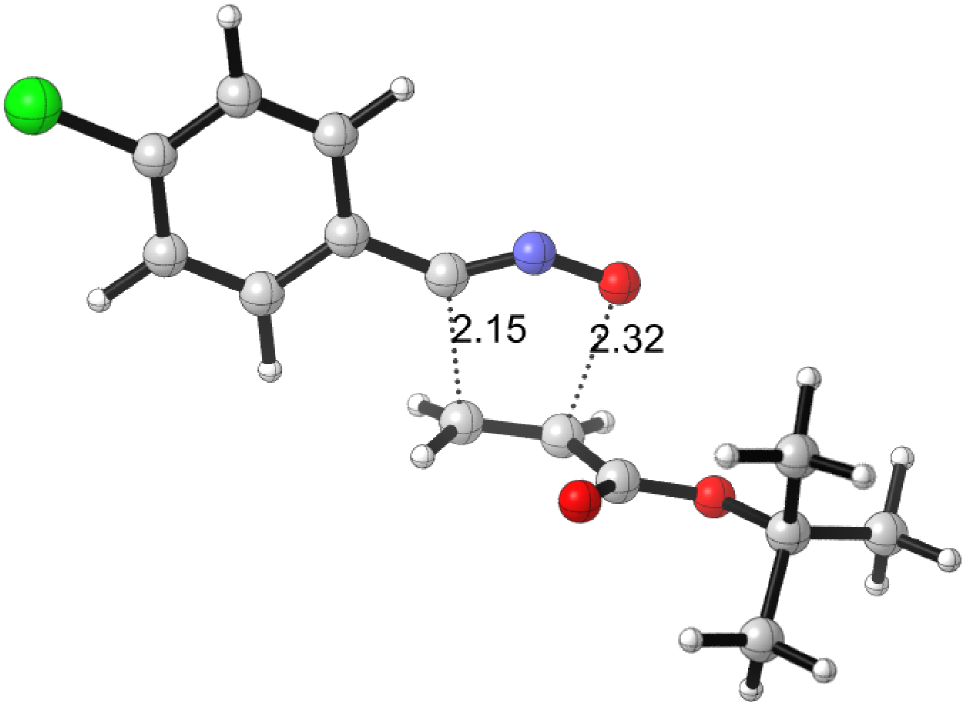


```
Sum of Electronic and Zero-point Energies = -1283.007212 Hartree
Sum of Electronic and Thermal Energies = -1282.988136 Hartree
Sum of Electronic and Thermal Enthalpies = -1282.987191 Hartree
Sum of Electronic and Thermal Free Energies = -1283.057119 Hartree

Dipole Moment = 1.5675 Debye

0 1
O                 3.88780       1.61098      -0.28907
N                 3.31512       0.83667       0.46062
C                 3.40320      -0.23889       0.99039
C                 2.67873      -1.12781       1.87432
C                 3.24440      -2.33546       2.29430
C                 1.39189      -0.77665       2.31155
C                 2.53871      -3.18463       3.14027
H                 4.23848      -2.61507       1.96497
C                 0.68564      -1.62112       3.15630
H                 0.95337       0.16063       1.98610
C                 1.26510      -2.82141       3.56446
H                 2.97321      -4.12143       3.47015
H                -0.30818      -1.35505       3.49868
C                 6.48603       1.31564       0.10813
O                 6.69643       1.38765       1.30133
O                 6.89099       2.20269      -0.80724
C                 7.54300       3.44520      -0.40953
C                 8.88059       3.14753       0.26347
H                 9.41802       4.08756       0.42258
H                 8.73887       2.65401       1.22527
H                 9.49149       2.50902      -0.38208
```

C	7.75854	4.15550	-1.74122
H	6.80006	4.33196	-2.23678
H	8.25214	5.11703	-1.57499
H	8.38553	3.54779	-2.39965
C	6.60839	4.25510	0.48595
H	7.03197	5.25304	0.63557
H	5.63106	4.35997	0.00561
H	6.47658	3.77802	1.45768
C	5.32681	-0.84912	0.24298
H	4.96066	-1.75497	-0.23115
H	5.72592	-0.91784	1.25288
C	5.72372	0.20958	-0.52577
H	5.59441	0.23853	-1.60074
Cl	0.38021	-3.88117	4.62168

p-CN-TS-exo

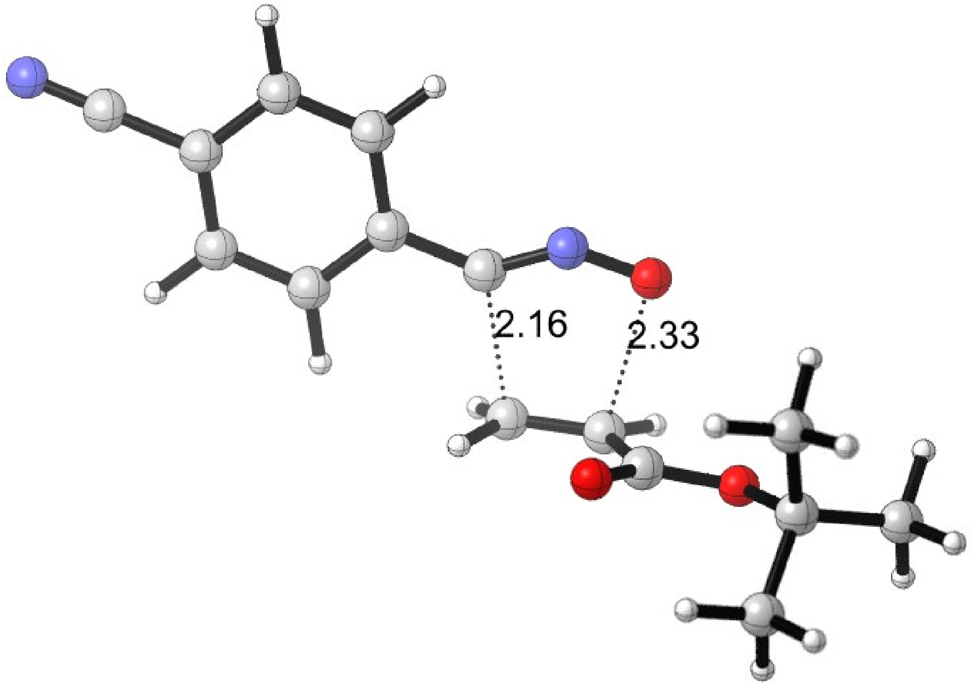

Sum of Electronic and Zero-point Energies = -915.645101 Hartree
Sum of Electronic and Thermal Energies = -915.625417 Hartree
Sum of Electronic and Thermal Enthalpies = -915.624473 Hartree
Sum of Electronic and Thermal Free Energies = -915.695413 Hartree

Dipole Moment = 2.6459 Debye

0 1

O	3.87349	1.59285	-0.30940
N	3.30820	0.81723	0.43978
C	3.40219	-0.25674	0.97318

C	2.67992	-1.13609	1.86702
C	3.23823	-2.35373	2.27147
C	1.40590	-0.76240	2.32640
C	2.53436	-3.19238	3.12597
H	4.22186	-2.64391	1.92154
C	0.70369	-1.59860	3.17918
H	0.97986	0.18326	2.00974
C	1.26661	-2.81736	3.58062
H	2.96260	-4.13654	3.44365
H	-0.27978	-1.31581	3.53818
C	6.47756	1.30913	0.10508
O	6.67114	1.37978	1.30109
O	6.88748	2.19840	-0.80417
C	7.53355	3.44277	-0.39883
C	8.86476	3.14594	0.28668
H	9.39904	4.08658	0.45221
H	8.71482	2.65129	1.24668
H	9.48305	2.50942	-0.35370
C	7.76019	4.15443	-1.72779
H	6.80622	4.32972	-2.23237
H	8.25019	5.11674	-1.55585
H	8.39477	3.54880	-2.38076
C	6.58854	4.24929	0.48856
H	7.00883	5.24772	0.64362
H	5.61585	4.35375	-0.00125
H	6.44836	3.77104	1.45854
C	5.33421	-0.86472	0.22421
H	4.97295	-1.77000	-0.25477
H	5.72709	-0.93372	1.23663
C	5.72869	0.19815	-0.53871
H	5.60501	0.22998	-1.61435
C	0.53573	-3.68709	4.46532
N	-0.05267	-4.38633	5.17563

p-F-TS-exo

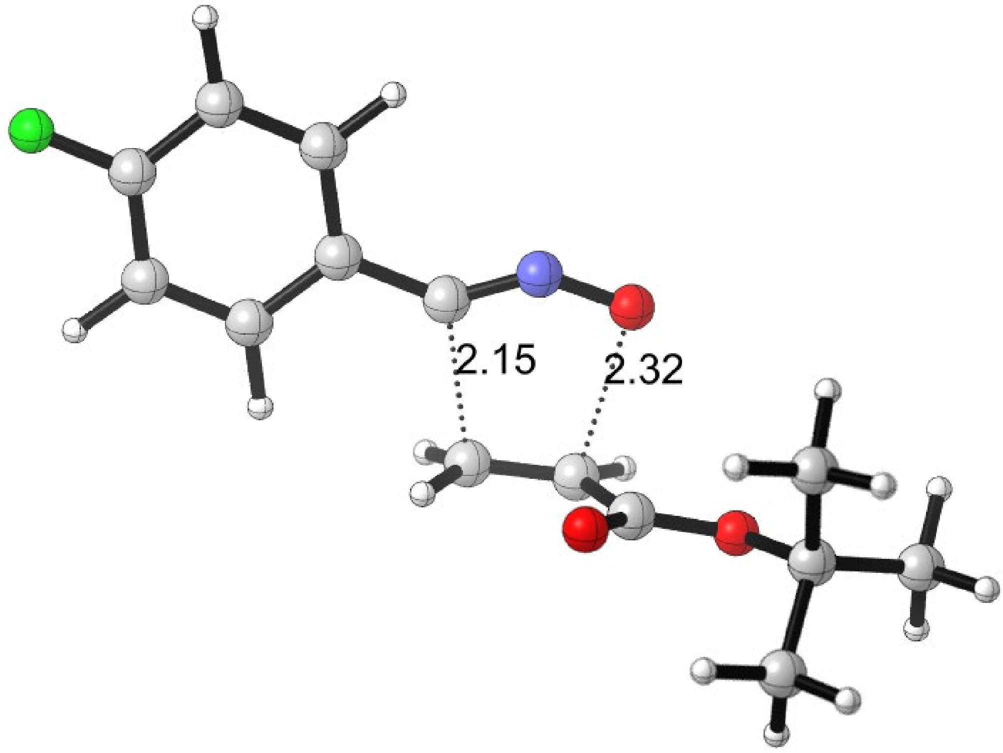

Sum of Electronic and Zero-point Energies = -922.648839 Hartree
Sum of Electronic and Thermal Energies = -922.630148 Hartree
Sum of Electronic and Thermal Enthalpies = -922.629204 Hartree
Sum of Electronic and Thermal Free Energies = -922.697904 Hartree

Dipole Moment = 1.7278 Debye

0 1

O	3.89378	1.61590	-0.27832
N	3.31947	0.84172	0.47171
C	3.40495	-0.23521	0.99853
C	2.67788	-1.12714	1.87815
C	3.24938	-2.33001	2.30649
C	1.38394	-0.78171	2.30137
C	2.54311	-3.18314	3.14852
H	4.24847	-2.60143	1.98587
C	0.67380	-1.62685	3.14253
H	0.94454	0.15203	1.96730
C	1.26929	-2.81369	3.54932
H	2.96550	-4.11928	3.49476
H	-0.32537	-1.38219	3.48439
C	6.48964	1.31491	0.10819
O	6.70810	1.38434	1.30014
O	6.89034	2.20342	-0.80793
C	7.54437	3.44502	-0.41141
C	8.88602	3.14632	0.25303
H	9.42402	4.08617	0.41141
H	8.74997	2.65024	1.21435
H	9.49338	2.50964	-0.39765

C	7.75192	4.15838	-1.74277
H	6.79044	4.33588	-2.23212
H	8.24651	5.11956	-1.57737
H	8.37491	3.55213	-2.40632
C	6.61495	4.25293	0.49131
H	7.03909	5.25075	0.64026
H	5.63467	4.35821	0.01713
H	6.48917	3.77387	1.46286
C	5.32538	-0.84698	0.24642
H	4.95629	-1.75213	-0.22678
H	5.72895	-0.91711	1.25442
C	5.72147	0.21170	-0.52316
H	5.58813	0.24169	-1.59759
F	0.58247	-3.63399	4.36250

p-H-P-from-TS-exo-STYRENE

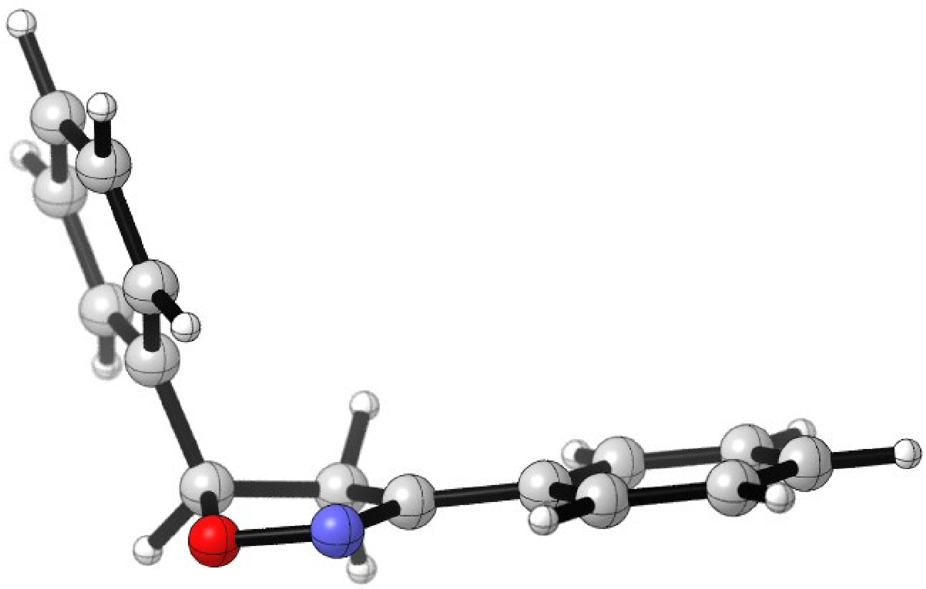

Sum of Electronic and Zero-point Energies = -708.839337 Hartree
Sum of Electronic and Thermal Energies = -708.826211 Hartree
Sum of Electronic and Thermal Enthalpies = -708.825267 Hartree
Sum of Electronic and Thermal Free Energies = -708.881628 Hartree

Dipole Moment = 3.3132 Debye

0 1

O	3.62441	1.64759	-0.29745
N	2.91891	0.93757	0.66054
C	3.46935	-0.20073	0.85597

C	2.91051	-1.17757	1.80393
C	3.49681	-2.44017	1.93398
C	1.78373	-0.86157	2.57708
C	2.96682	-3.37668	2.82043
H	4.36944	-2.69785	1.34098
C	1.25998	-1.79635	3.46044
H	1.33211	0.11940	2.47254
C	1.84893	-3.05722	3.58545
H	3.43005	-4.35389	2.91181
H	0.38828	-1.54347	4.05569
H	1.43631	-3.78466	4.27726
C	4.68046	-0.42651	-0.01712
H	4.44402	-1.14236	-0.81264
H	5.54502	-0.79402	0.54094
C	4.89033	1.00413	-0.53623
H	5.06019	1.03593	-1.61563
C	6.00422	1.73074	0.18915
C	7.32825	1.51070	-0.19990
C	5.73649	2.58067	1.26222
C	8.37581	2.12704	0.47930
H	7.54052	0.85696	-1.04364
C	6.78642	3.19696	1.94307
H	4.70632	2.76554	1.55017
C	8.10560	2.97202	1.55584
H	9.40044	1.95442	0.16497
H	6.57056	3.85839	2.77637
H	8.91984	3.45673	2.08512

p-H-R-from-TS--exo-STYRENE

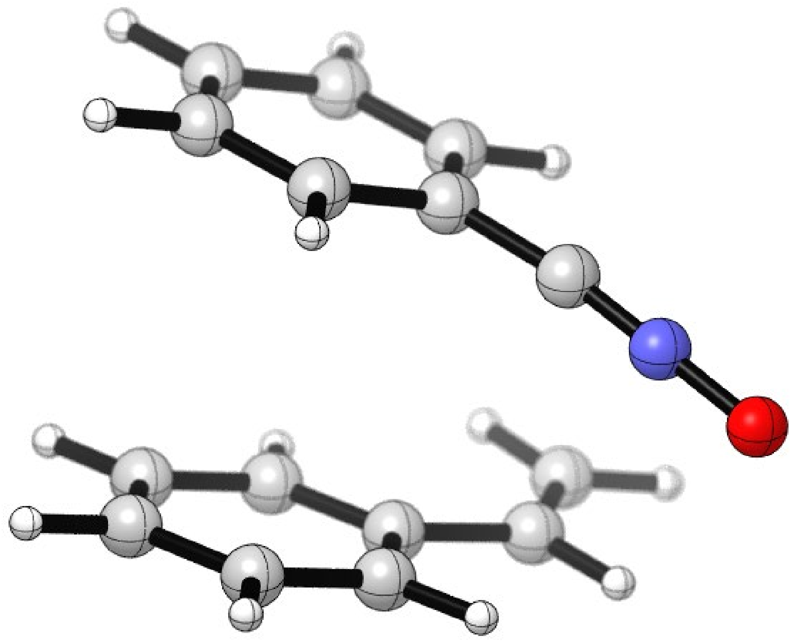

Sum of Electronic and Zero-point Energies = -708.778061 Hartree
Sum of Electronic and Thermal Energies = -708.762239 Hartree
Sum of Electronic and Thermal Enthalpies = -708.761295 Hartree
Sum of Electronic and Thermal Free Energies = -708.824169 Hartree

Dipole Moment = 4.8758 Debye

0 1

O	2.75479	0.88565	-2.27468
N	2.76202	0.47866	-1.12849
C	2.77566	0.09210	-0.03680
C	2.96283	-0.34619	1.31662
C	3.27659	-1.68717	1.57734
C	2.88795	0.58062	2.36368
C	3.52278	-2.09060	2.88510
H	3.33702	-2.39094	0.75403
C	3.13406	0.16336	3.66704
H	2.66280	1.61896	2.14473
C	3.45355	-1.16810	3.92986
H	3.76917	-3.12761	3.08939
H	3.08786	0.88374	4.47731
H	3.64866	-1.48798	4.94850
C	6.04208	-1.71541	-0.67010
H	5.95754	-2.33183	-1.55845
H	6.27723	-2.22435	0.26069
C	5.86112	-0.39288	-0.73895
H	5.60748	0.04888	-1.70265
C	5.93287	0.56169	0.38355
C	5.53310	1.88684	0.16617

C	6.35184	0.19310	1.67030
C	5.54303	2.81784	1.20319
H	5.19895	2.18220	-0.82633
C	6.36374	1.12054	2.70552
H	6.66966	-0.82665	1.86590
C	5.95843	2.43727	2.47763
H	5.22841	3.83953	1.01342
H	6.68706	0.81533	3.69621
H	5.97065	3.16005	3.28767

p-H-TS-exo-regio-isomer-ii

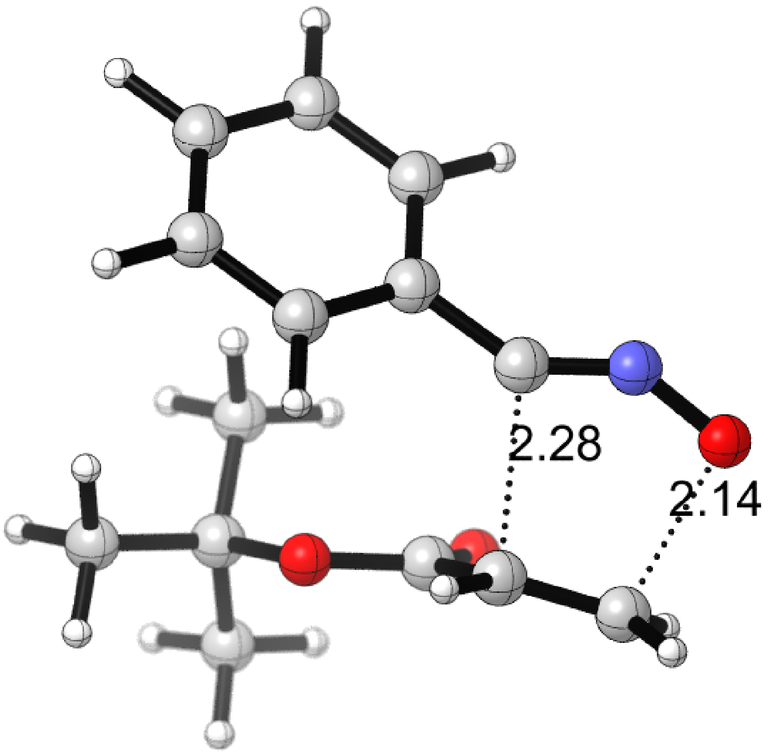

Sum of Electronic and Zero-point Energies = -823.431549 Hartree
Sum of Electronic and Thermal Energies = -823.414013 Hartree
Sum of Electronic and Thermal Enthalpies = -823.413069 Hartree
Sum of Electronic and Thermal Free Energies = -823.476504 Hartree

Dipole Moment = 4.8360 Debye

0 1

O	3.00211	-0.47675	0.63518
N	2.82551	0.61763	0.10238
C	3.39597	1.47716	-0.50644
C	3.30823	2.82028	-1.03316
C	3.95498	3.15081	-2.22939
C	2.59405	3.79742	-0.32429
C	3.86897	4.44855	-2.72333
H	4.53135	2.39588	-2.75346

C	2.51229	5.09065	-0.82835
H	2.11360	3.53209	0.61155
C	3.14793	5.41822	-2.02677
H	4.36856	4.70364	-3.65240
H	1.95677	5.84551	-0.28166
H	3.08516	6.42989	-2.41453
C	6.06172	1.55167	0.19311
O	6.09087	1.70926	1.39506
O	6.60235	2.39287	-0.70633
C	7.25539	3.62695	-0.28449
C	8.46267	3.31189	0.59594
H	9.03595	4.22989	0.75845
H	8.15630	2.90966	1.56175
H	9.11201	2.58714	0.09522
C	7.70328	4.24105	-1.60629
H	6.83719	4.42331	-2.24952
H	8.21010	5.19264	-1.42300
H	8.39247	3.56915	-2.12556
C	6.25074	4.54043	0.41405
H	6.73298	5.49821	0.63405
H	5.39432	4.73091	-0.24132
H	5.89926	4.10130	1.34881
C	5.08917	-0.71188	0.23289
H	4.88455	-1.65362	-0.26065
H	5.29100	-0.72835	1.29842
C	5.40941	0.40793	-0.49291
H	5.49616	0.36962	-1.57404

p-H-TS-exo-STYRENE

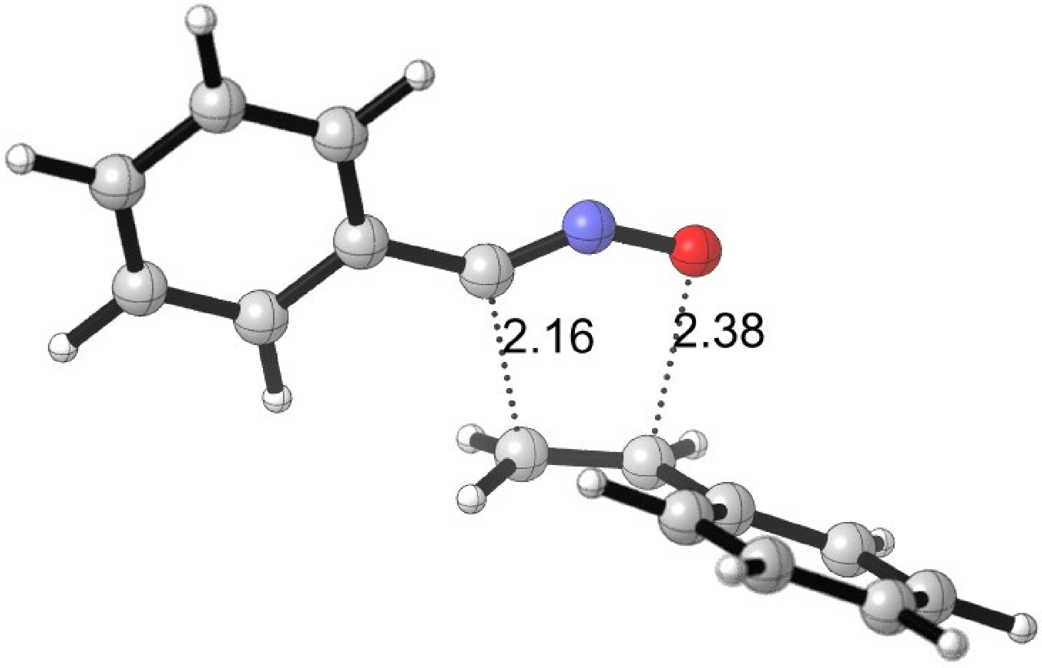

Sum of Electronic and Zero-point Energies = -708.745216 Hartree
Sum of Electronic and Thermal Energies = -708.730975 Hartree
Sum of Electronic and Thermal Enthalpies = -708.730030 Hartree
Sum of Electronic and Thermal Free Energies = -708.788989 Hartree

Dipole Moment = 3.5195 Debye

0 1

O	3.67797	1.52177	-0.50859
N	3.14243	0.74013	0.26364
C	3.30793	-0.29960	0.84724
C	2.66977	-1.17311	1.81121
C	3.14762	-2.47130	2.02110
C	1.56456	-0.71140	2.54404
C	2.52595	-3.29890	2.95237
H	3.99872	-2.83207	1.45405
C	0.94915	-1.54701	3.46771
H	1.20132	0.29743	2.37819
C	1.42753	-2.84157	3.67664
H	2.90159	-4.30481	3.10876
H	0.09411	-1.18484	4.02936
H	0.94513	-3.48940	4.40121
C	5.28038	-0.82640	0.12751
H	4.93827	-1.66266	-0.47497
H	5.62260	-1.06117	1.13248
C	5.72516	0.30966	-0.49987
H	5.64897	0.38238	-1.58059
C	6.47379	1.38258	0.17015
C	7.22459	2.28285	-0.59664

C	6.45525	1.54461	1.56334
C	7.95503	3.30164	0.00814
H	7.23284	2.17749	-1.67831
C	7.18123	2.56463	2.16839
H	5.85186	0.88095	2.17697
C	7.93811	3.44531	1.39435
H	8.53449	3.98624	-0.60344
H	7.15078	2.67932	3.24759
H	8.50301	4.24153	1.86859

p-H-TS-exo

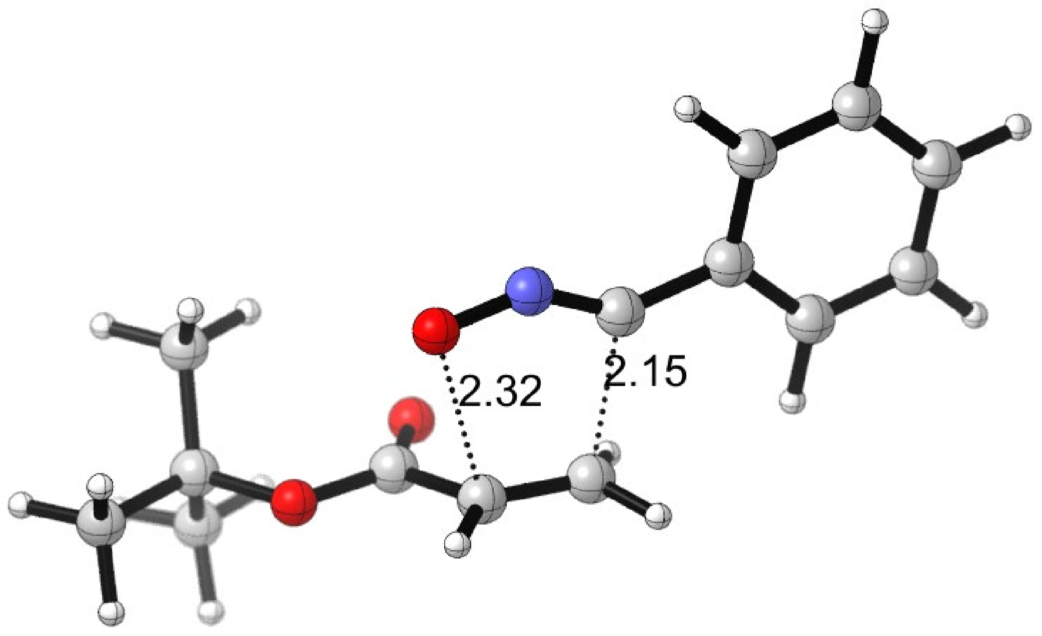

Sum of Electronic and Zero-point Energies = -823.429872 Hartree
Sum of Electronic and Thermal Energies = -823.412024 Hartree
Sum of Electronic and Thermal Enthalpies = -823.411080 Hartree
Sum of Electronic and Thermal Free Energies = -823.477552 Hartree

Dipole Moment = 3.2206 Debye

0 1

O	3.89913	1.61668	-0.27182
N	3.32210	0.84203	0.47582
C	3.40756	-0.23552	1.00227
C	2.67815	-1.12957	1.87925
C	3.24617	-2.33543	2.30221
C	1.38601	-0.77962	2.30260
C	2.52835	-3.18294	3.14200
H	4.24466	-2.60766	1.97957

C	0.67967	-1.63225	3.14115
H	0.95244	0.15779	1.97031
C	1.24707	-2.83577	3.56305
H	2.97541	-4.11627	3.46792
H	-0.31797	-1.35609	3.46669
H	0.69127	-3.49864	4.21806
C	6.49456	1.31537	0.11227
O	6.71728	1.38769	1.30324
O	6.89252	2.20301	-0.80677
C	7.54489	3.44601	-0.41362
C	8.88893	3.15121	0.24790
H	9.42532	4.09241	0.40385
H	8.75616	2.65584	1.21004
H	9.49599	2.51497	-0.40352
C	7.74786	4.15818	-1.74638
H	6.78477	4.33328	-2.23346
H	8.24120	5.12046	-1.58350
H	8.37014	3.55211	-2.41078
C	6.61634	4.25373	0.49029
H	7.03877	5.25274	0.63628
H	5.63449	4.35587	0.01867
H	6.49414	3.77601	1.46295
C	5.32718	-0.84501	0.25679
H	4.95819	-1.75111	-0.21454
H	5.72867	-0.91389	1.26564
C	5.72529	0.21130	-0.51520
H	5.59188	0.23886	-1.58970

p-Me-TS-exo

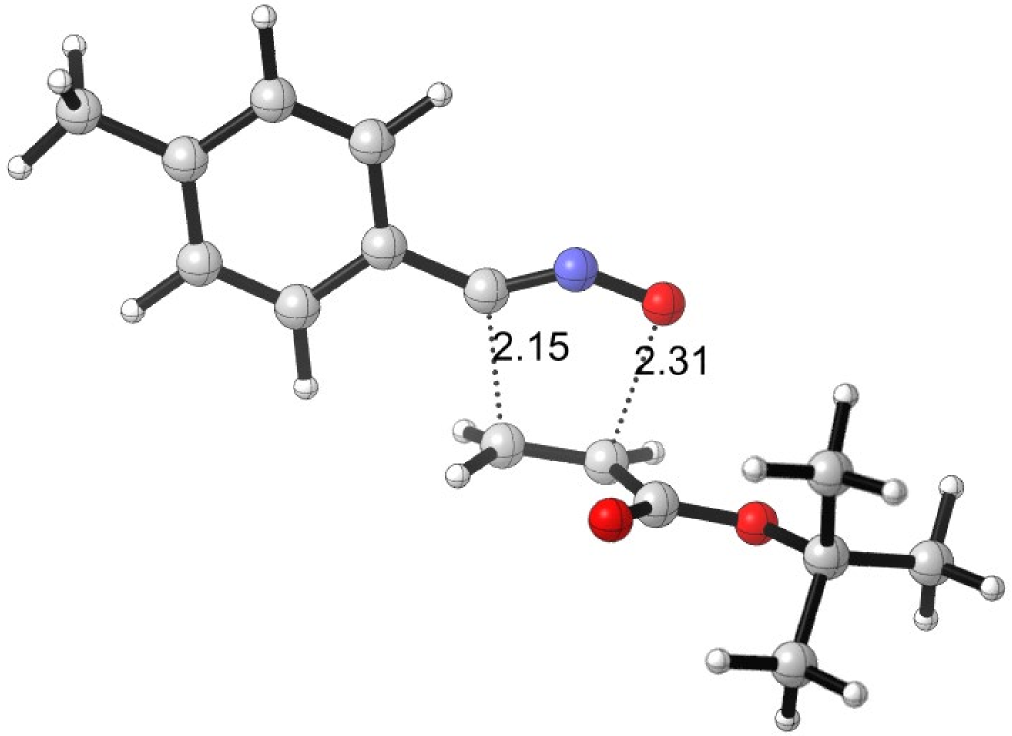

Sum of Electronic and Zero-point Energies = -862.702086 Hartree
Sum of Electronic and Thermal Energies = -862.682321 Hartree
Sum of Electronic and Thermal Enthalpies = -862.681377 Hartree
Sum of Electronic and Thermal Free Energies = -862.753146 Hartree

Dipole Moment = 3.8713 Debye

0 1

O	3.86611	1.73183	-0.14913
N	3.30118	0.93683	0.58802
C	3.37396	-0.17169	1.04762
C	2.66313	-1.09321	1.90980
C	3.21035	-2.33447	2.24086
C	1.40162	-0.73985	2.41720
C	2.50716	-3.20750	3.06755
H	4.18394	-2.61947	1.85827
C	0.71593	-1.62025	3.23957
H	0.97466	0.22410	2.16040
C	1.25435	-2.86910	3.57982
H	2.94554	-4.16932	3.31811
H	-0.25893	-1.33565	3.62730
C	6.46560	1.31630	0.06103
O	6.76224	1.31390	1.23803
O	6.84487	2.23834	-0.83223
C	7.57203	3.43037	-0.41465
C	8.93917	3.04668	0.14655
H	9.52404	3.95585	0.31764
H	8.84265	2.50547	1.08809
H	9.47830	2.42298	-0.57318

C	7.72631	4.20668	-1.71783
H	6.74447	4.44609	-2.13509
H	8.26895	5.13834	-1.53488
H	8.28186	3.61425	-2.45023
C	6.73461	4.22342	0.58610
H	7.20761	5.19534	0.75785
H	5.73163	4.39029	0.18231
H	6.65021	3.69819	1.53808
C	5.22220	-0.80388	0.16054
H	4.79158	-1.66864	-0.33553
H	5.67868	-0.94373	1.13815
C	5.61638	0.27894	-0.57626
H	5.42199	0.37125	-1.63772
C	0.49221	-3.80815	4.47804
H	-0.49102	-4.03917	4.05715
H	1.03142	-4.74747	4.61768
H	0.33120	-3.35944	5.46313

p-NO2-TS-exo

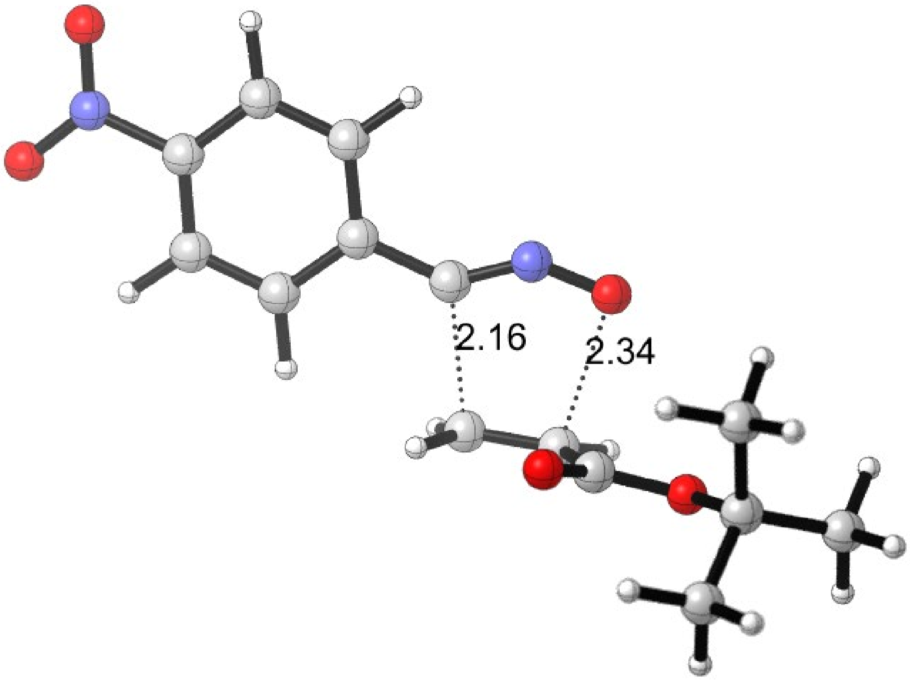

Sum of Electronic and Zero-point Energies = -1027.857858 Hartree
Sum of Electronic and Thermal Energies = -1027.837487 Hartree
Sum of Electronic and Thermal Enthalpies = -1027.836543 Hartree
Sum of Electronic and Thermal Free Energies = -1027.909439 Hartree

Dipole Moment = 3.0454 Debye

0 1

O	3.98956	2.34297	1.04454
N	3.48218	1.38875	1.60313
C	3.26654	0.20938	1.50404
C	2.64891	-0.88391	2.22267
C	2.02543	-0.63546	3.45770
C	2.66637	-2.17987	1.69333
C	1.42701	-1.67359	4.15476
H	2.01815	0.37135	3.86015
C	2.06777	-3.22447	2.38722
H	3.14899	-2.37304	0.74280
C	1.45927	-2.95008	3.60372
H	0.94042	-1.51122	5.10883
H	2.06786	-4.23647	2.00128
C	6.07584	1.41729	-0.31162
O	6.73413	0.73990	0.45038
O	6.45750	2.57872	-0.85004
C	7.71509	3.20942	-0.46040
C	8.89393	2.33262	-0.87488
H	9.82480	2.88336	-0.70816
H	8.92274	1.40782	-0.29802
H	8.82568	2.09068	-1.93991
C	7.70364	4.50787	-1.25896
H	6.83541	5.11389	-0.98639
H	8.61206	5.08011	-1.05167
H	7.65911	4.29556	-2.33076
C	7.70362	3.49949	1.03846
H	8.56852	4.12151	1.28858
H	6.79403	4.04564	1.30546
H	7.75017	2.57950	1.62224
C	4.20148	-0.17137	-0.40745
H	3.30038	-0.54244	-0.88681
H	4.88125	-0.88856	0.04834
C	4.70796	1.04715	-0.76150
H	4.19768	1.72464	-1.43528
N	0.82197	-4.05547	4.34168
O	0.86268	-5.16334	3.83848
O	0.29534	-3.78882	5.40649

p-OMe-TS-exo

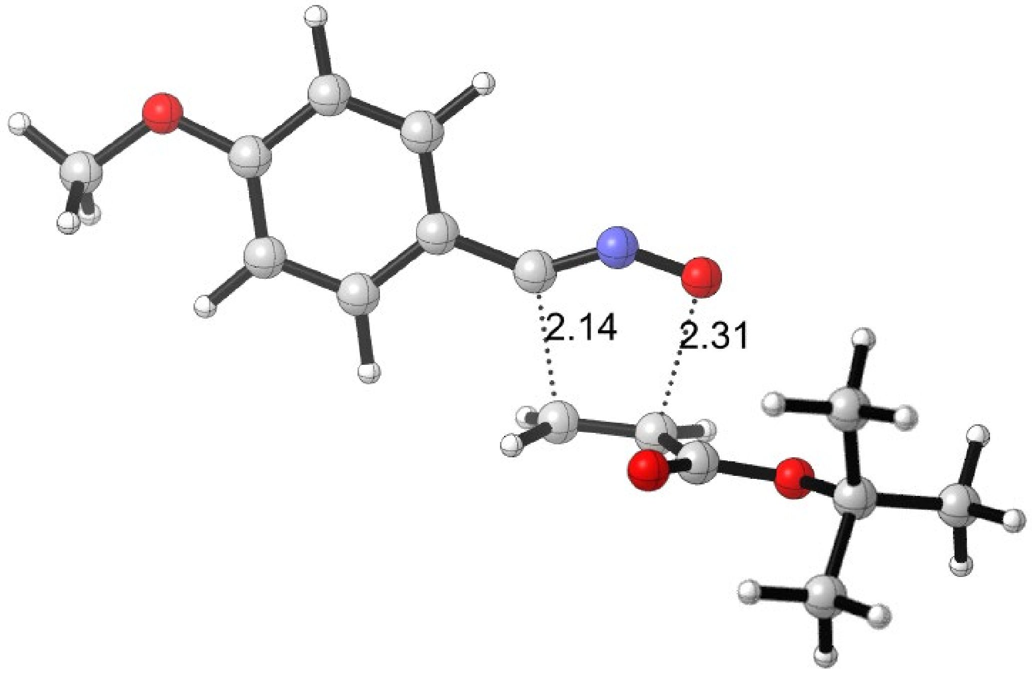


```
Sum of Electronic and Zero-point Energies = -937.880776 Hartree
Sum of Electronic and Thermal Energies = -937.860342 Hartree
Sum of Electronic and Thermal Enthalpies = -937.859398 Hartree
Sum of Electronic and Thermal Free Energies = -937.932503 Hartree

Dipole Moment = 4.4921 Debye

0 1
O                 3.91697      1.65822     -0.21821
N                 3.35118      0.87204      0.52975
C                 3.44106     -0.21836      1.02778
C                 2.72543     -1.14031      1.88305
C                 3.32142     -2.31874      2.32960
C                 1.40214     -0.85369      2.27012
C                 2.62587     -3.20614      3.15008
H                 4.34019     -2.55371      2.04244
C                 0.70870     -1.72707      3.08419
H                 0.93252      0.06149      1.92496
C                 1.31543     -2.91120      3.53037
H                 3.11688     -4.11236      3.48205
H                -0.31008     -1.52012      3.39319
C                 6.51570      1.33752      0.08567
O                 6.77966      1.38539      1.26958
O                 6.88827      2.24177     -0.82850
C                 7.55544      3.47522     -0.43179
C                 8.91906      3.16566      0.18138
H                 9.46218      4.10282      0.33869
H                 8.81518      2.65167      1.13729
H                 9.50389      2.54118     -0.50103
```

C	7.71836	4.21325	-1.75600
H	6.74084	4.39979	-2.20888
H	8.21920	5.17113	-1.58992
H	8.31771	3.61904	-2.45150
C	6.65756	4.26671	0.51642
H	7.08631	5.26218	0.66834
H	5.66144	4.37886	0.07825
H	6.56504	3.77019	1.48296
C	5.33819	-0.81715	0.22790
H	4.95244	-1.71473	-0.24630
H	5.77010	-0.90364	1.22264
C	5.71994	0.25060	-0.53742
H	5.55584	0.29760	-1.60691
O	0.55172	-3.70032	4.32205
C	1.11866	-4.90395	4.80338
H	2.00115	-4.70361	5.42160
H	0.34808	-5.37603	5.41079
H	1.39154	-5.56900	3.97606

Ph-oximyl-radical-deprot

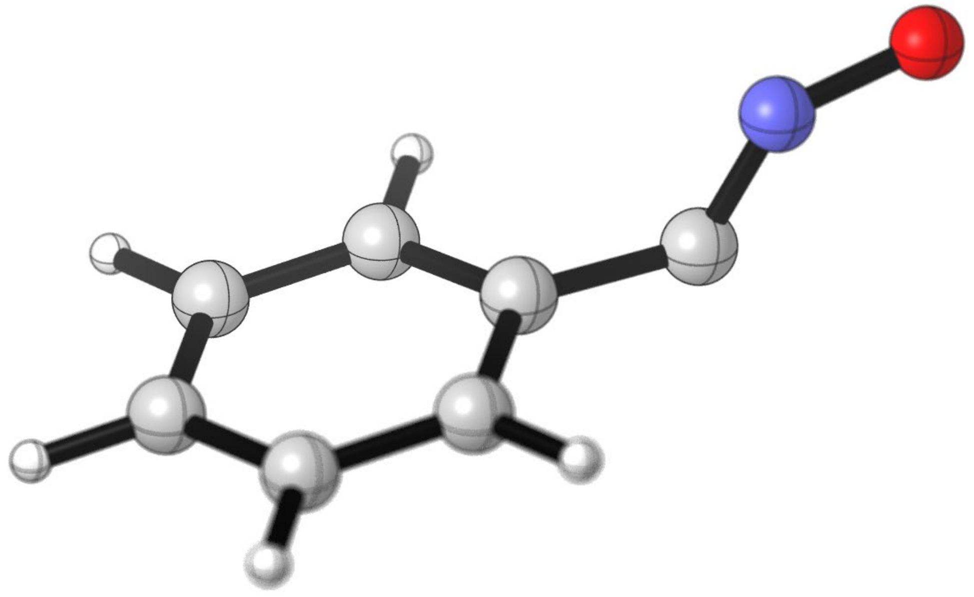

Sum of electronic and zero-point Energies= -399.391143
Sum of electronic and thermal Energies= -399.384518
Sum of electronic and thermal Enthalpies= -399.383574
Sum of electronic and thermal Free Energies= -399.423244

Dipole Moment = 8. 8.2465 Debye

-1 2

C	-2.07500500	1.20193700	0.04922400
C	-0.69871500	1.20453000	-0.14550300
C	0.03808700	0.00004100	-0.24369300
C	-0.69885800	-1.20435700	-0.14545200
C	-2.07514900	-1.20159200	0.04927200
C	-2.78457100	0.00021600	0.14552900
H	-2.60459600	2.14944300	0.12906700
H	-0.15900900	2.14421600	-0.22806700
H	-0.15926400	-2.14411100	-0.22797200
H	-2.60485300	-2.14903200	0.12915300
H	-3.86112700	0.00028400	0.29063800
C	1.45156000	-0.00004900	-0.55091600
N	2.38096700	-0.00025900	0.28819400
O	3.64183600	-0.00040600	0.30409300

Ph-oximyl-radical-Nu-attack-P-N-oxide-MALEATE_dihedral-scan-iii_TS-CALC-gas

Sum of electronic and zero-point Energies= -933.449704

Sum of electronic and thermal Energies= -933.431942

Sum of electronic and thermal Enthalpies= -933.430998

Sum of electronic and thermal Free Energies= -933.497514

Dipole Moment = 6.1063 Debye

-1 2

C	3.60307100	1.57867500	0.67671500
C	2.23411200	1.79779000	0.73686500
C	1.32333500	0.72247900	0.83379400

C	1.86708600	-0.57697300	0.86039200
C	3.24346200	-0.79076800	0.81135700
C	4.12854700	0.28173000	0.72072600
H	4.27519800	2.43098800	0.60318800
H	1.82846400	2.80355400	0.71264700
H	1.20380600	-1.43822800	0.88501900
H	3.62338700	-1.80972800	0.82987700
H	5.20132800	0.11507000	0.67965200
C	-0.11160300	0.95218600	0.91860200
N	-0.59184300	2.15140400	0.65527600
O	-1.86103400	2.32719200	0.74229300
C	-1.09111800	-0.11130400	1.36745300
C	-1.72143100	-0.99460800	0.34271600
H	-2.57974000	-1.58905000	0.63753800
H	-1.92126800	0.52849700	1.74138900
C	-1.31210700	-1.12022600	-1.03899600
O	-0.29797600	-0.70136300	-1.55967300
O	-2.23899800	-1.83800900	-1.75380300
C	-1.91188000	-2.01087400	-3.12296300
H	-2.73307900	-2.58199400	-3.55729200
H	-1.81231600	-1.04299000	-3.62194400
H	-0.96855500	-2.55396800	-3.23131700
C	-0.62323000	-0.93700000	2.54383900
O	-0.71402400	-2.14395300	2.65403900
O	-0.09614600	-0.16864500	3.51275200
C	0.46790800	-0.87690100	4.60674000
H	1.27018200	-1.53223600	4.25644200
H	0.86487700	-0.11586600	5.27773400
H	-0.29066000	-1.48086100	5.11237400

Additional calculation outputs below refer to additional quality-control calculations, inclusive of solvent modeling at the geometry optimization stage. All outputs below have thus been generated at the M06/Def2TZVP/SMD(MeCN) level of theory. See above, **Section 2.6**.

```
chloride


Sum of Electronic and Zero-point Energies = -460.364085 Hartree

Sum of Electronic and Thermal Energies = -460.362669 Hartree

Sum of Electronic and Thermal Enthalpies = -460.361725 Hartree

Sum of Electronic and Thermal Free Energies = -460.379108 Hartree


Dipole Moment = 0.0000 Debye


-1 1

Cl               0.00000      0.00000      0.00000
```

```
chlorine-radical_SOLVENT-OPT


Sum of electronic and zero-point Energies=           -460.134897

Sum of electronic and thermal Energies=              -460.133481

Sum of electronic and thermal Enthalpies=            -460.132536

Sum of electronic and thermal Free Energies=         -460.150574


Dipole Moment = 0.0000 Debye


0 2

Cl               0.00000      0.00000      0.00000
```

```
chlorine
```

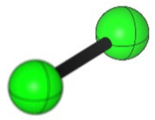

Sum of Electronic and Zero-point Energies = -920.361587 Hartree

Sum of Electronic and Thermal Energies = -920.359053 Hartree

Sum of Electronic and Thermal Enthalpies = -920.358109 Hartree

Sum of Electronic and Thermal Free Energies = -920.383366 Hartree

Dipole Moment = 0.0000 Debye

0 1

Cl	-6.63363	1.58046	0.00000
Cl	-8.62223	1.58046	0.00000

dimethyl-fumerate-product_SOLVENT-OPT

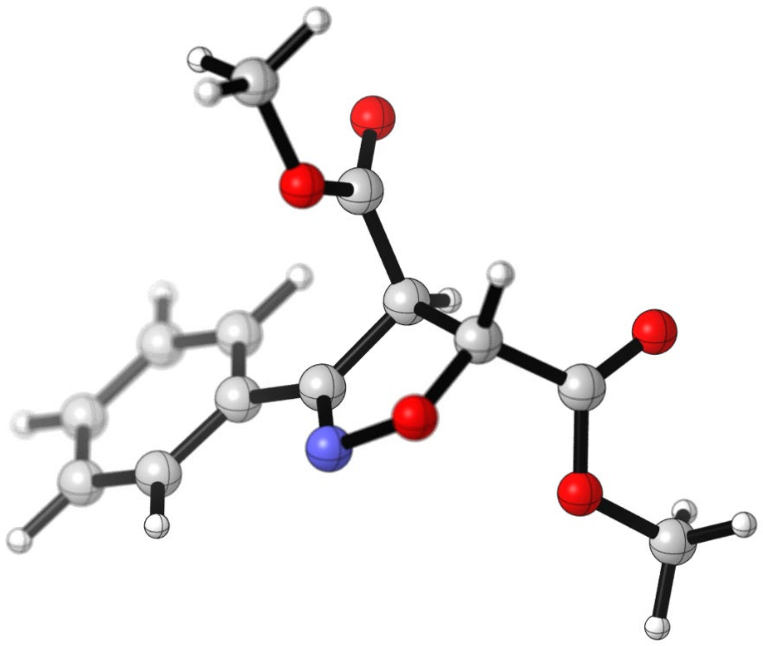

Sum of Electronic and Zero-point Energies = -933.806715 Hartree

Sum of Electronic and Thermal Energies = -933.789382 Hartree

Sum of Electronic and Thermal Enthalpies = -933.788438 Hartree

Sum of Electronic and Thermal Free Energies = -933.854214 Hartree

Dipole Moment = 2.2635 Debye

0 1

C	-0.84011	-1.64433	0.88258
H	-0.35382	-2.44544	0.32905
C	-0.04476	-0.32844	0.84204
H	-0.51339	0.43564	0.22348
C	1.35111	-0.57882	0.28580
C	-2.25503	-1.50866	0.34596
O	-2.69704	-2.15603	-0.56470
O	1.54853	-0.68005	-0.89688
O	2.27292	-0.70447	1.22172
O	-2.93207	-0.57778	1.00506
C	-4.27849	-0.34630	0.56770
H	-4.67084	0.43046	1.21674
H	-4.28273	-0.01394	-0.46917
H	-4.86494	-1.25815	0.66751
C	3.60231	-0.97987	0.75684
H	4.21478	-1.06384	1.64908
H	3.61772	-1.91203	0.19442
H	3.95461	-0.16295	0.12908
O	-0.01516	0.13114	2.18989
N	-0.37452	-0.89988	3.03883
C	-0.82868	-1.88656	2.37780
C	-1.33392	-3.10510	3.02223
C	-1.76698	-4.17529	2.24112
C	-1.39732	-3.20053	4.41581

C	-2.25867	-5.32663	2.84378
H	-1.71857	-4.12094	1.16047
C	-1.88523	-4.35016	5.01041
H	-1.06507	-2.36948	5.02438
C	-2.31844	-5.41654	4.22619
H	-2.59289	-6.15209	2.22849
H	-1.93179	-4.41749	6.08989
H	-2.70209	-6.31346	4.69577

dimethyl-fumerate-TS-cycloaddition-ii_SOLVENT-OPT_iii

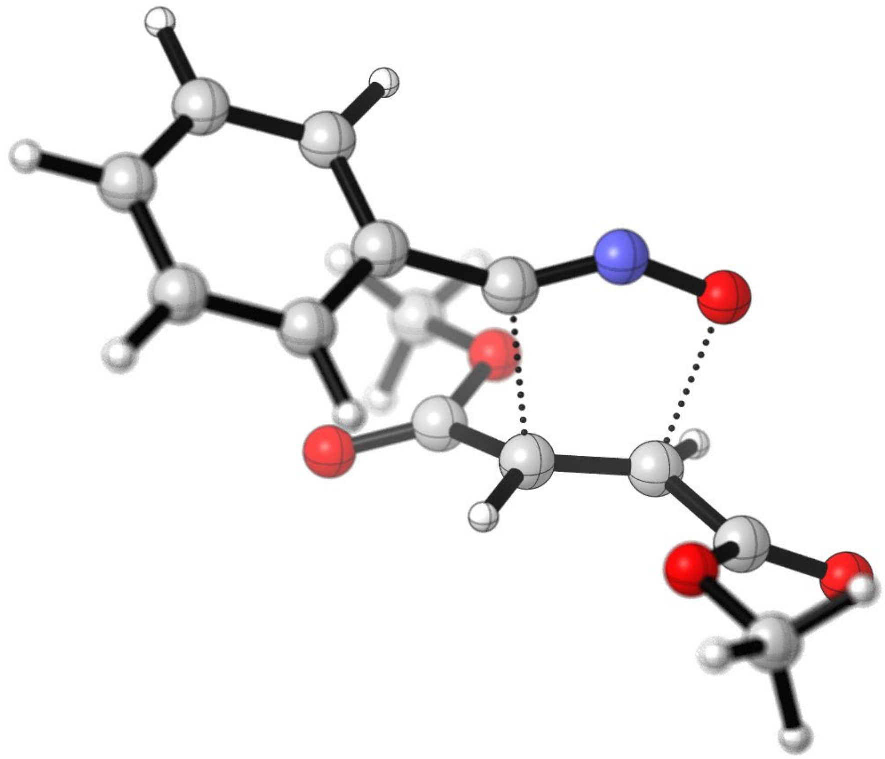

Sum of Electronic and Zero-point Energies = -933.726313 Hartree

Sum of Electronic and Thermal Energies = -933.708056 Hartree

Sum of Electronic and Thermal Enthalpies = -933.707111 Hartree

Sum of Electronic and Thermal Free Energies = -933.774883 Hartree

Dipole Moment = 6.2613 Debye

0 1

C	-0.74272	-1.50009	0.66620
H	-0.23402	-2.42456	0.42558
C	-0.09421	-0.29787	0.51749
H	-0.64475	0.62399	0.40247
C	1.33322	-0.22223	0.12624
C	-2.22138	-1.58724	0.62539
O	-2.82209	-2.59421	0.33938
O	1.84892	0.76990	-0.32604
O	1.98552	-1.36455	0.33672
O	-2.81891	-0.44238	0.95758
C	-4.24949	-0.45208	0.93020
H	-4.55782	0.55009	1.21342
H	-4.60704	-0.68929	-0.07087
H	-4.63649	-1.18151	1.64089
C	3.37651	-1.35926	0.00314
H	3.74111	-2.35595	0.23354
H	3.51130	-1.14294	-1.05581
H	3.90434	-0.61621	0.59970
O	0.43717	0.17724	2.51954
N	0.00127	-0.84168	3.05768
C	-0.57653	-1.84760	2.77758
C	-1.20765	-3.05552	3.24853
C	-0.75167	-4.29310	2.79399
C	-2.30295	-2.97085	4.10962
C	-1.38128	-5.44920	3.22458
H	0.08720	-4.33843	2.11098
C	-2.92236	-4.13514	4.53548

H	-2.65340	-2.00102	4.43873
C	-2.46325	-5.37078	4.09441
H	-1.02920	-6.41268	2.87989
H	-3.76717	-4.07744	5.20943
H	-2.95373	-6.27699	4.42650

dimethyl-fumnerate_SOLVENT-OPT

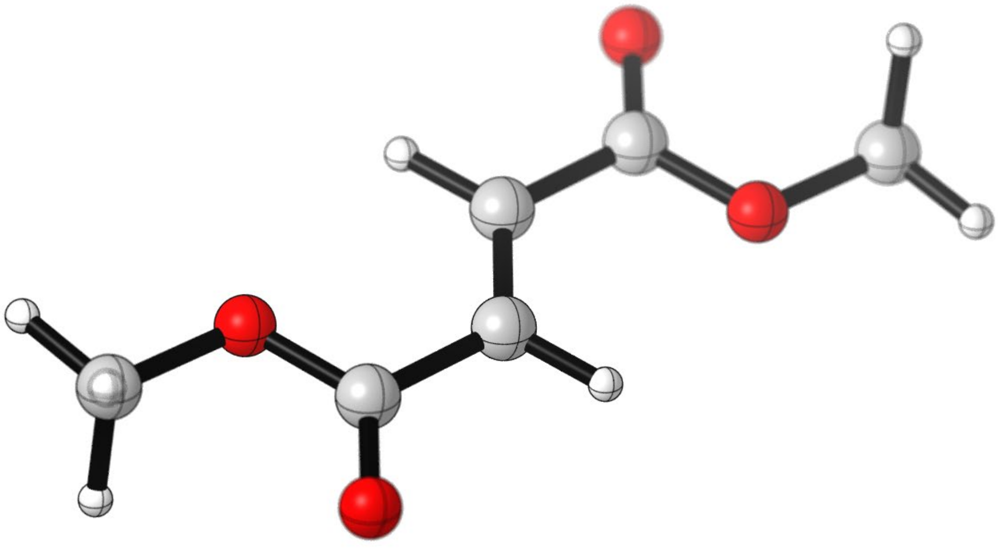

Sum of Electronic and Zero-point Energies = -534.217524 Hartree

Sum of Electronic and Thermal Energies = -534.206772 Hartree

Sum of Electronic and Thermal Enthalpies = -534.205828 Hartree

Sum of Electronic and Thermal Free Energies = -534.254949 Hartree

Dipole Moment = 0.0000 Debye

0 1

C	-0.62450	-1.51292	-0.00138
H	-0.09011	-2.45373	-0.00138
C	0.00690	-0.34641	-0.00138
H	-0.52749	0.59441	-0.00139
C	1.48672	-0.21846	-0.00138
C	-2.10432	-1.64086	-0.00138
O	-2.66281	-2.70980	-0.00138
O	2.04521	0.85048	-0.00138
O	2.12073	-1.38586	-0.00138
O	-2.73834	-0.47347	-0.00138
C	-4.16910	-0.53829	-0.00138
H	-4.52223	-1.05615	0.88924
H	-4.51157	0.49200	-0.00137
H	-4.52224	-1.05615	-0.89199
C	3.55150	-1.32103	-0.00138
H	3.89397	-2.35132	-0.00137
H	3.90463	-0.80317	-0.89199
H	3.90463	-0.80318	0.88924

dimethyl-maleate-product-ii_SOLVENT-OPT

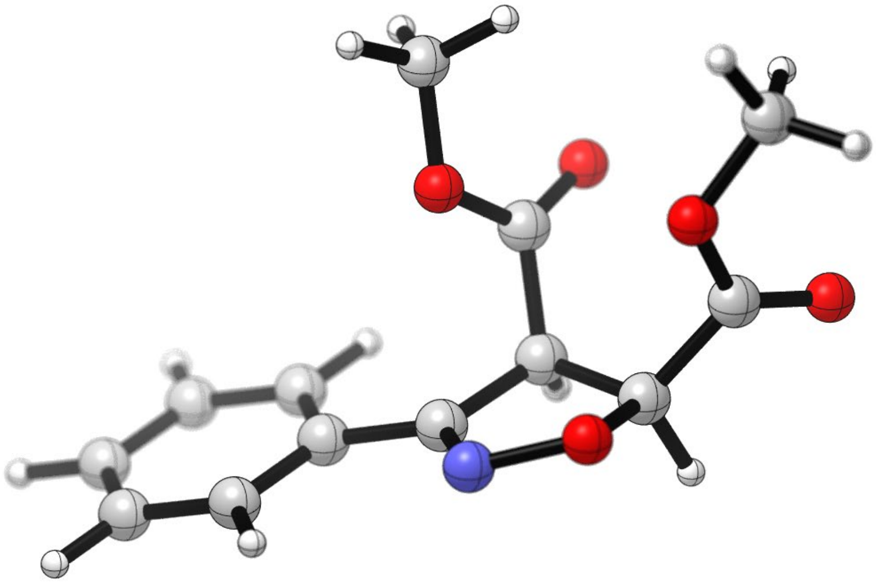

Sum of Electronic and Zero-point Energies = -933.806321 Hartree

Sum of Electronic and Thermal Energies = -933.789154 Hartree

Sum of Electronic and Thermal Enthalpies = -933.788209 Hartree

Sum of Electronic and Thermal Free Energies = -933.852407 Hartree

Dipole Moment = 4.8714 Debye

0 1

C	-0.26472	-1.70840	1.24508
C	0.33974	-0.40683	0.68373
H	-0.34160	0.05789	-0.02579
C	1.66445	-0.61862	-0.03730
O	1.72297	-1.01137	-1.17319
O	2.71662	-0.35850	0.72083
C	3.99507	-0.62277	0.12753

H	4.73062	-0.36478	0.88296
H	4.06945	-1.67770	-0.13469
H	4.12989	-0.00944	-0.76185
H	-1.34786	-1.69745	1.12348
C	0.26886	-2.97426	0.59930
O	-0.36194	-3.61355	-0.19805
O	1.50520	-3.24699	0.98515
C	2.13551	-4.35755	0.33177
H	2.21060	-4.16513	-0.73778
H	3.12310	-4.44093	0.77473
H	1.56435	-5.26828	0.50433
O	0.05236	-1.67608	2.63826
N	0.31712	-0.36601	3.00696
C	0.46280	0.36480	1.97588
C	0.76264	1.79956	2.06248
C	0.95153	2.53818	0.89583
C	0.86471	2.43565	3.30271
C	1.24439	3.89404	0.96562
H	0.86825	2.06212	-0.07408
C	1.15454	3.78734	3.36721
H	0.71526	1.86551	4.21049
C	1.34633	4.52039	2.19918
H	1.39067	4.45847	0.05363
H	1.23174	4.27430	4.33103
H	1.57311	5.57763	2.25408

dimethyl-maleate-TS-cycloaddition-iii_SOLVENT-OPT_ii

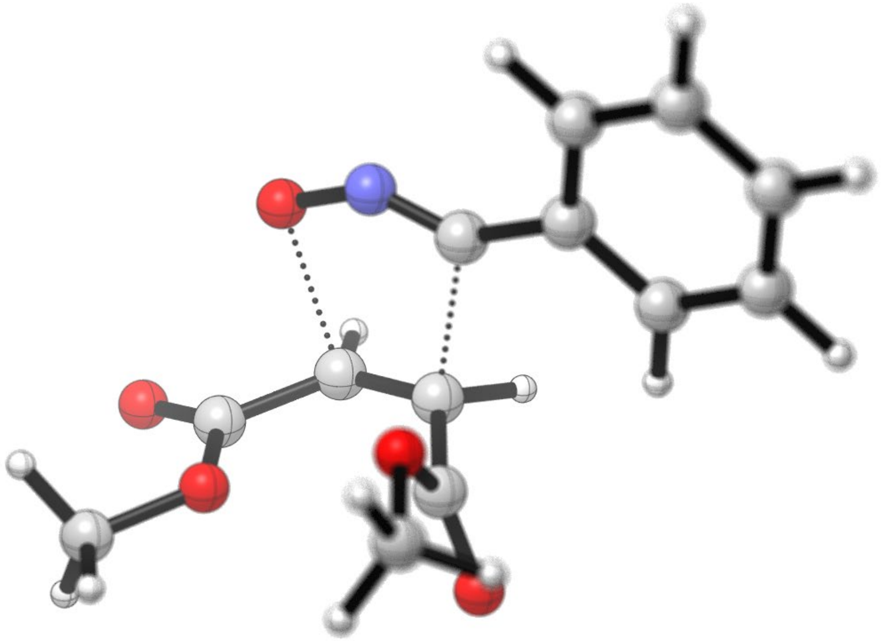

Sum of Electronic and Zero-point Energies = -933.716967 Hartree

Sum of Electronic and Thermal Energies = -933.698727 Hartree

Sum of Electronic and Thermal Enthalpies = -933.697783 Hartree

Sum of Electronic and Thermal Free Energies = -933.765360 Hartree

Dipole Moment = 7.7721 Debye

0 1

C	-0.40985	-1.81586	0.57642
C	0.17040	-0.62151	0.22734
H	-0.49589	0.12874	-0.18554
C	1.57668	-0.49468	-0.29158
O	1.81379	-0.41055	-1.47003
O	2.50412	-0.42534	0.65359
C	3.85533	-0.32177	0.19547

H	4.47011	-0.29269	1.09023
H	4.11426	-1.18617	-0.41498
H	3.98772	0.59067	-0.38489
H	-1.48868	-1.87647	0.59883
C	0.24444	-3.14065	0.63997
O	-0.36478	-4.16510	0.82997
O	1.56129	-3.09662	0.44475
C	2.24732	-4.35018	0.50115
H	1.86973	-5.02405	-0.26681
H	3.29443	-4.12406	0.32203
H	2.12126	-4.80588	1.48239
O	-0.33793	-1.68179	2.77863
N	0.00997	-0.51000	2.80504
C	0.31171	0.39727	2.08950
C	0.74683	1.77651	2.11638
C	0.90695	2.50387	0.93879
C	1.02038	2.37269	3.35209
C	1.34022	3.82052	0.99822
H	0.69002	2.05582	-0.02201
C	1.44912	3.68775	3.39810
H	0.89525	1.79973	4.26172
C	1.61187	4.41404	2.22315
H	1.46426	4.38182	0.08129
H	1.65964	4.14696	4.35548
H	1.95017	5.44142	2.26444

dimethyl-maleate_SOLVENT-OPT

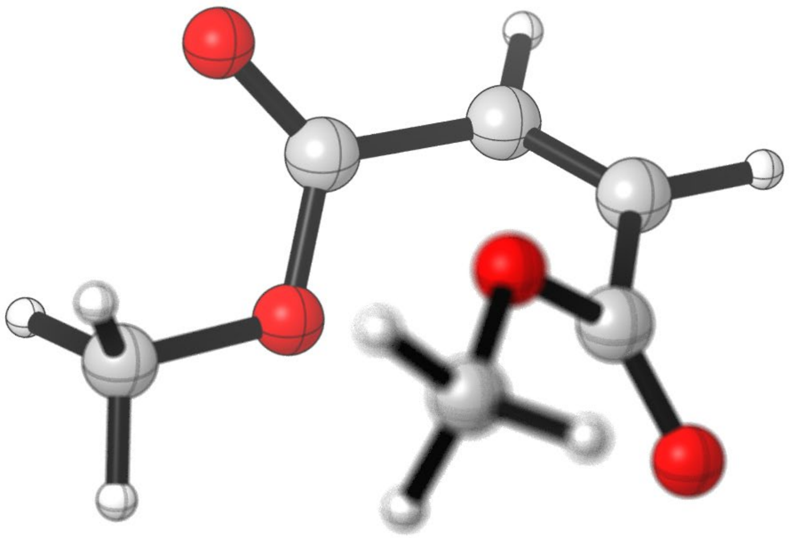

Sum of Electronic and Zero-point Energies = -534.213065 Hartree

Sum of Electronic and Thermal Energies = -534.202535 Hartree

Sum of Electronic and Thermal Enthalpies = -534.201591 Hartree

Sum of Electronic and Thermal Free Energies = -534.250004 Hartree

Dipole Moment = 1.4894 Debye

0 1

C	-0.51193	-1.56964	0.12162
C	0.14864	-0.44050	-0.09304
H	-0.37437	0.50258	-0.19578
C	1.62785	-0.37449	-0.26365
O	2.16261	0.20143	-1.17561
O	2.27977	-1.00284	0.71023
C	3.69832	-1.10783	0.54560

H	4.05823	-1.65135	1.41410
H	3.92784	-1.65593	-0.36820
H	4.15033	-0.11813	0.50327
H	-1.58707	-1.57254	0.25191
C	0.15357	-2.89527	0.25806
O	-0.10934	-3.68092	1.13183
O	1.05293	-3.11326	-0.69748
C	1.84314	-4.29893	-0.55587
H	2.37722	-4.28091	0.39398
H	1.21154	-5.18473	-0.60619
H	2.54513	-4.28863	-1.38441

dipolarophile-isomer-2_SOLVENT-OPT

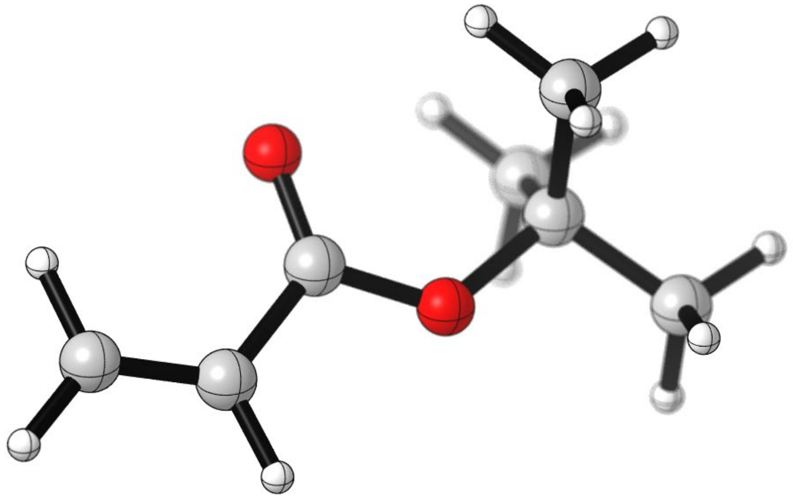

Sum of Electronic and Zero-point Energies = -424.222419 Hartree

Sum of Electronic and Thermal Energies = -424.211972 Hartree

Sum of Electronic and Thermal Enthalpies = -424.211028 Hartree

Sum of Electronic and Thermal Free Energies = -424.257866 Hartree

Dipole Moment = 2.2552 Debye

0 1

C	-2.53912	0.49529	0.03372
H	-2.21687	-0.53969	0.03515
H	-3.60317	0.69558	0.03341
C	-1.65441	1.48118	0.03239
H	-1.94871	2.52284	0.03094
C	-0.19376	1.19379	0.03285
O	0.28163	0.08405	0.03465
O	0.50129	2.32853	0.03100
C	1.96463	2.34063	0.03106
C	2.49264	1.67758	-1.23199
H	3.56958	1.84302	-1.29114
H	2.30486	0.60633	-1.23289
H	2.03002	2.12391	-2.11425
C	2.29284	3.82429	0.02833
H	1.87998	4.30857	0.91451
H	3.37455	3.96097	0.02844
H	1.88061	4.30519	-0.85998
C	2.49240	1.68216	1.29660
H	3.56940	1.84748	1.35519
H	2.02987	2.13187	2.17719
H	2.30434	0.61097	1.30139

H-product-endo_SOLVENT-OPT

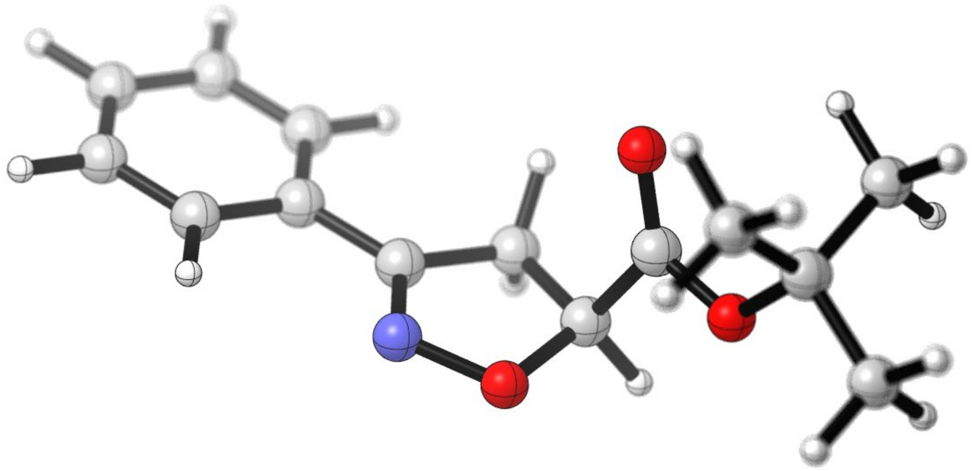

Sum of Electronic and Zero-point Energies = -823.817485 Hartree

Sum of Electronic and Thermal Energies = -823.800584 Hartree

Sum of Electronic and Thermal Enthalpies = -823.799640 Hartree

Sum of Electronic and Thermal Free Energies = -823.863646 Hartree

Dipole Moment = 3.7532 Debye

0 1

O	2.66729	2.61945	-0.01379
N	1.93074	1.46266	0.20414
C	2.67619	0.54566	0.67623
C	2.14885	-0.78065	1.02453
C	2.98973	-1.71988	1.61860
C	0.81515	-1.11663	0.77353

C	2.50580	-2.97619	1.96190
H	4.02513	-1.47182	1.81593
C	0.33762	-2.36963	1.11539
H	0.15987	-0.39180	0.30796
C	1.18135	-3.30353	1.71145
H	3.16730	-3.69800	2.42382
H	-0.69604	-2.62279	0.91615
C	4.68717	2.31851	-1.22412
O	4.95313	1.31091	-1.82790
O	4.82631	3.55620	-1.66623
C	5.27628	3.84601	-3.03371
C	4.30871	3.23701	-4.03592
H	4.54879	3.61823	-5.02956
H	4.37582	2.15151	-4.05569
H	3.28446	3.52852	-3.79613
C	5.22340	5.36255	-3.09434
H	5.87796	5.79893	-2.33853
H	5.55187	5.70085	-4.07736
H	4.20531	5.71636	-2.92560
C	6.70053	3.34958	-3.22197
H	7.06899	3.69855	-4.18797
H	7.34947	3.75361	-2.44293
H	6.75306	2.26313	-3.20390
C	4.10637	0.97471	0.85647
H	4.35618	1.03891	1.91598
H	4.79881	0.28774	0.37234
C	4.07332	2.32967	0.17547
H	4.50179	3.13897	0.76216
H	0.80432	-4.28314	1.97705

HCl_SOLVENT-OPT

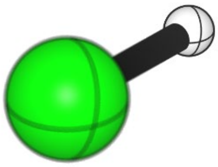

Sum of Electronic and Zero-point Energies = -460.796856 Hartree

Sum of Electronic and Thermal Energies = -460.794496 Hartree

Sum of Electronic and Thermal Enthalpies = -460.793552 Hartree

Sum of Electronic and Thermal Free Energies = -460.814744 Hartree

Dipole Moment = 1.5766 Debye

0 1

H	3.24432	-0.82342	-1.87589
Cl	2.79774	-1.77910	-2.61113

HFIP-anion_SOLVENT-OPT

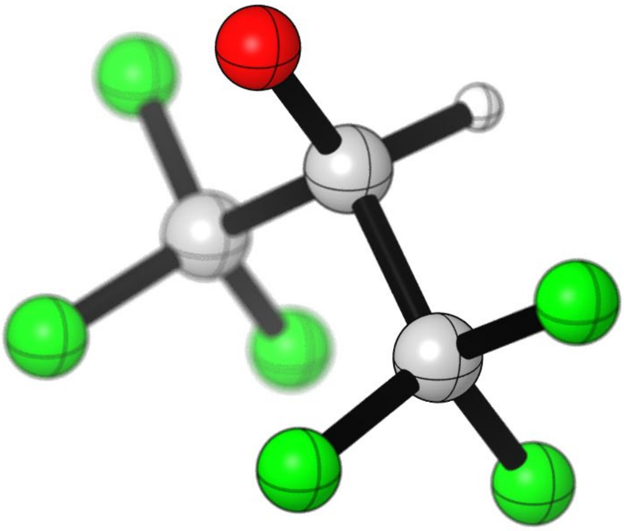

Sum of Electronic and Zero-point Energies = -789.369207 Hartree

Sum of Electronic and Thermal Energies = -789.360569 Hartree

Sum of Electronic and Thermal Enthalpies = -789.359625 Hartree

Sum of Electronic and Thermal Free Energies = -789.403992 Hartree

Dipole Moment = 19.2188 Debye

-1 1

C	-3.23180	-0.27698	0.12140
C	-5.40968	0.98129	0.11297
C	-3.91663	1.04886	-0.27265
H	-3.94319	0.99489	-1.38921
F	-5.61112	0.87556	1.43645
F	-6.09388	-0.03412	-0.45482
F	-6.03064	2.10572	-0.27681

F	-3.78923	-1.38196	-0.41727
F	-1.95477	-0.27042	-0.29364
F	-3.19778	-0.48177	1.44761
O	-3.31567	2.09190	0.26970

HFIP-OH_SOLVENT-OPT

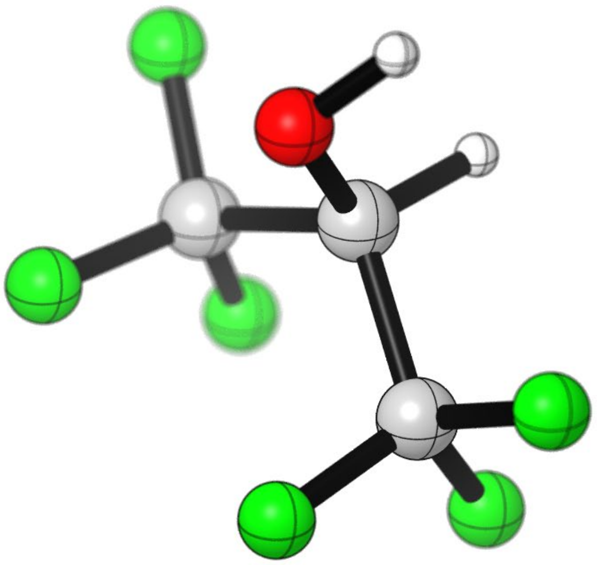

Sum of Electronic and Zero-point Energies = -789.844023 Hartree

Sum of Electronic and Thermal Energies = -789.834830 Hartree

Sum of Electronic and Thermal Enthalpies = -789.833885 Hartree

Sum of Electronic and Thermal Free Energies = -789.879311 Hartree

Dipole Moment = 4.0422 Debye

0 1

C	-3.24403	-0.29092	0.15817
C	-5.46309	0.95583	0.18701
C	-3.97968	1.01072	-0.16896
H	-3.91213	1.15497	-1.25069
F	-5.65896	0.86347	1.50284
F	-6.07552	-0.07899	-0.38907
F	-6.06684	2.06873	-0.22933
F	-3.68636	-1.31705	-0.56827
F	-1.94435	-0.13794	-0.10637
F	-3.35216	-0.62620	1.44478
O	-3.42837	2.05229	0.57107
H	-2.75809	2.50371	0.04227

oxime-H-atom-abstraction-Cl-TS_SOLVENT-OPT

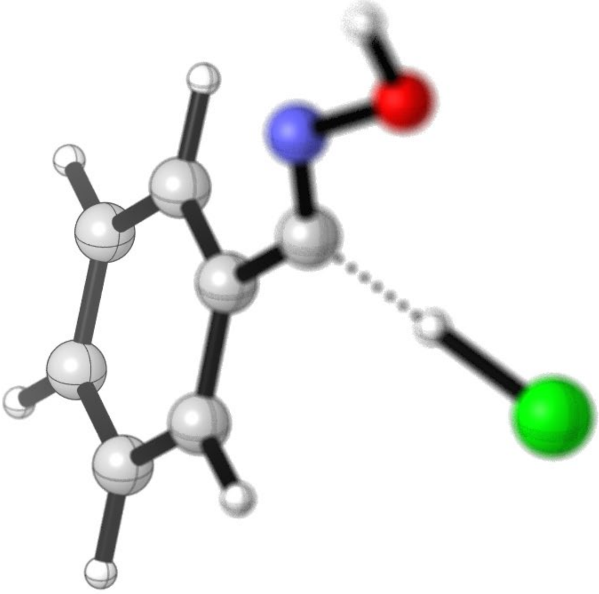

Sum of Electronic and Zero-point Energies = -860.883209 Hartree

Sum of Electronic and Thermal Energies = -860.873640 Hartree

Sum of Electronic and Thermal Enthalpies = -860.872696 Hartree

Sum of Electronic and Thermal Free Energies = -860.921211 Hartree

Dipole Moment = 5.7631 Debye

0 2

C	-1.98306	1.19692	0.35954
C	-0.60752	1.18401	0.20428
C	0.02971	0.00028	-0.18375
C	-0.71628	-1.15120	-0.44360
C	-2.09177	-1.12695	-0.28164
C	-2.72440	0.04445	0.11893
H	-2.47998	2.10807	0.66701

H	-0.01890	2.07452	0.38374
H	-0.21401	-2.05626	-0.76234
H	-2.67169	-2.02054	-0.47220
H	-3.80024	0.06186	0.23903
N	2.38145	0.46069	0.31758
O	3.66338	0.44432	-0.16283
H	4.19941	0.77050	0.57735
C	1.46776	-0.02612	-0.35162
H	1.91638	-0.74952	-1.57627
Cl	2.30153	-1.43226	-2.77603

p-H-dipole_SOLVENT-OPT

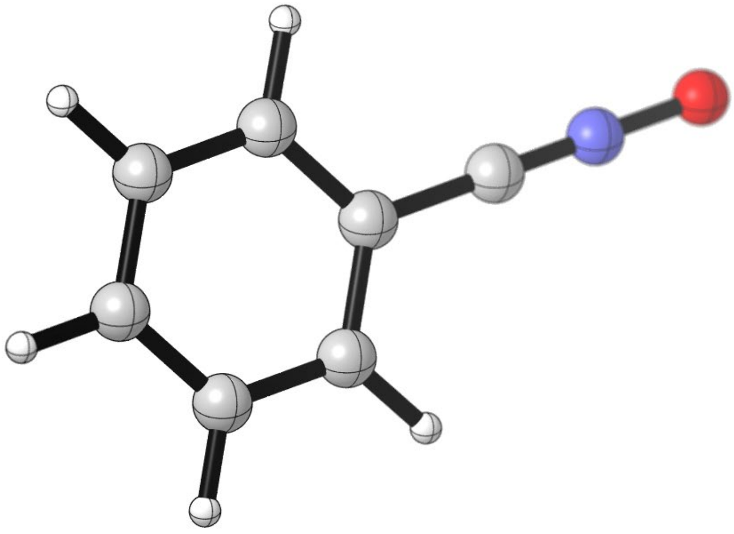

Sum of Electronic and Zero-point Energies = -399.532381 Hartree

Sum of Electronic and Thermal Energies = -399.525356 Hartree

Sum of Electronic and Thermal Enthalpies = -399.524412 Hartree

Sum of Electronic and Thermal Free Energies = -399.564438 Hartree

Dipole Moment = 6.5475 Debye

0 1

C	-3.74703	0.28212	-0.00362
C	-2.36164	0.27425	-0.00299
C	-1.66911	1.48694	-0.00345
C	-2.36157	2.69967	-0.00453
C	-3.74696	2.69189	-0.00515
C	-4.43986	1.48702	-0.00470
H	-4.28675	-0.65594	-0.00325
H	-1.81264	-0.65801	-0.00214
H	-1.81251	3.63190	-0.00488
H	-4.28663	3.62997	-0.00601
H	-5.52249	1.48705	-0.00518
C	-0.23690	1.48690	-0.00281
N	0.91127	1.48687	-0.00235
O	2.12744	1.48683	-0.00186

p-H-TS-endo-ii_SOLVENT-OPT_iii

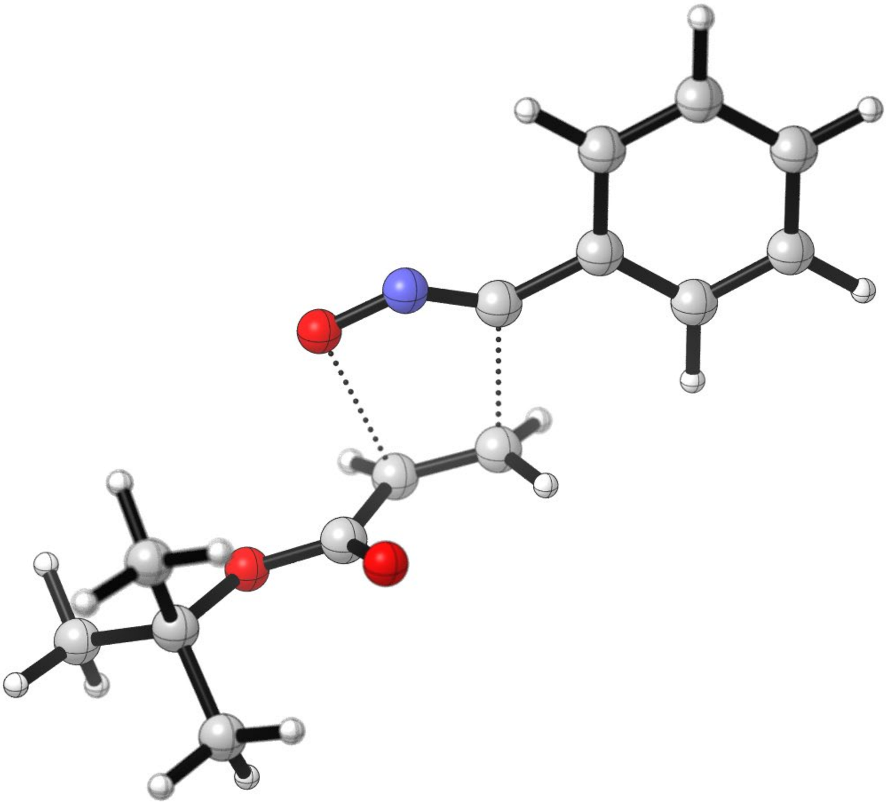

Sum of Electronic and Zero-point Energies = -823.730407 Hartree

Sum of Electronic and Thermal Energies = -823.712568 Hartree

Sum of Electronic and Thermal Enthalpies = -823.711624 Hartree

Sum of Electronic and Thermal Free Energies = -823.777637 Hartree

Dipole Moment = 4.0889 Debye

0 1

O	2.29361	2.37990	-0.74889
N	1.95623	1.29224	-0.30980
C	2.34239	0.36969	0.34541
C	1.90363	-0.90773	0.86613
C	2.77303	-1.72795	1.58197
C	0.58273	-1.31278	0.64233
C	2.32263	-2.94671	2.06996

H	3.79462	-1.42046	1.75720
C	0.14613	-2.53023	1.13371
H	-0.08657	-0.66934	0.08609
C	1.01361	-3.35023	1.84839
H	3.00116	-3.58124	2.62523
H	-0.87612	-2.84033	0.95861
H	0.66704	-4.30177	2.23088
C	4.90902	2.30617	-1.20094
O	5.11693	1.34792	-1.90978
O	5.10695	3.57667	-1.55073
C	5.52685	3.94577	-2.90011
C	4.46906	3.51636	-3.90543
H	4.72558	3.92348	-4.88487
H	4.40790	2.43298	-3.98388
H	3.49287	3.90989	-3.61604
C	5.60769	5.46186	-2.83134
H	6.34347	5.77152	-2.08753
H	5.90565	5.85813	-3.80244
H	4.63808	5.88392	-2.56330
C	6.89581	3.35905	-3.21152
H	7.26409	3.79852	-4.14006
H	7.60042	3.60593	-2.41502
H	6.85447	2.27884	-3.32875
C	4.27892	0.99085	0.78381
H	4.70773	0.12631	0.28818
H	4.14290	0.93537	1.85735
C	4.40257	2.21108	0.18575
H	4.25688	3.13443	0.72728

p-H-TS-endo-IRC-SM-min_SOLVENT-OPT

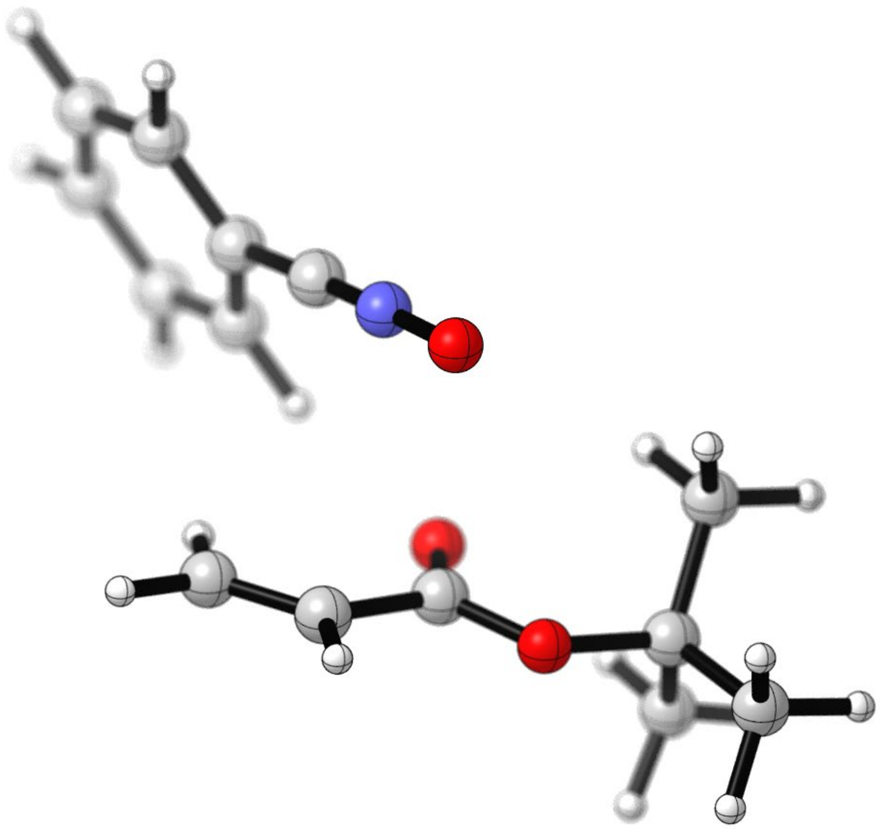

Sum of Electronic and Zero-point Energies = -823.759954 Hartree

Sum of Electronic and Thermal Energies = -823.740582 Hartree

Sum of Electronic and Thermal Enthalpies = -823.739638 Hartree

Sum of Electronic and Thermal Free Energies = -823.809689 Hartree

Dipole Moment = 4.1560 Debye

0 1

O	-1.56279	2.00876	-0.07175
N	-1.69590	0.83648	0.22310
C	-1.80285	-0.26939	0.51006
C	-1.87018	-1.66024	0.84447
C	-0.74950	-2.46520	0.62598

C	-3.04037	-2.19869	1.38219
C	-0.80850	-3.81159	0.94819
H	0.14695	-2.02341	0.20991
C	-3.08357	-3.54687	1.69959
H	-3.89989	-1.56232	1.54616
C	-1.97174	-4.35271	1.48350
H	0.05650	-4.44003	0.77992
H	-3.98845	-3.96927	2.11684
H	-2.01220	-5.40538	1.73303
C	1.35627	1.29459	0.08389
O	1.22338	0.18559	-0.37979
O	1.73733	2.36988	-0.59713
C	1.99790	2.31803	-2.03547
C	0.72850	1.93237	-2.77949
H	0.91020	2.02032	-3.85201
H	0.43129	0.90906	-2.55922
H	-0.08694	2.60435	-2.50893
C	2.39351	3.74829	-2.36268
H	3.28710	4.03482	-1.80600
H	2.60427	3.83673	-3.42879
H	1.58538	4.43551	-2.10819
C	3.15224	1.37083	-2.32743
H	3.44556	1.48636	-3.37218
H	4.01219	1.61968	-1.70258
H	2.87586	0.33319	-2.15573
C	0.82658	0.67816	2.38754
H	0.73847	-0.35533	2.07027
H	0.65822	0.90406	3.43305
C	1.13454	1.62799	1.51696
H	1.23414	2.66770	1.80105

p-H-Z-chloro-iminium-HFIP-deprot-TS-anion-iv_SOLVENT-OPT_vii

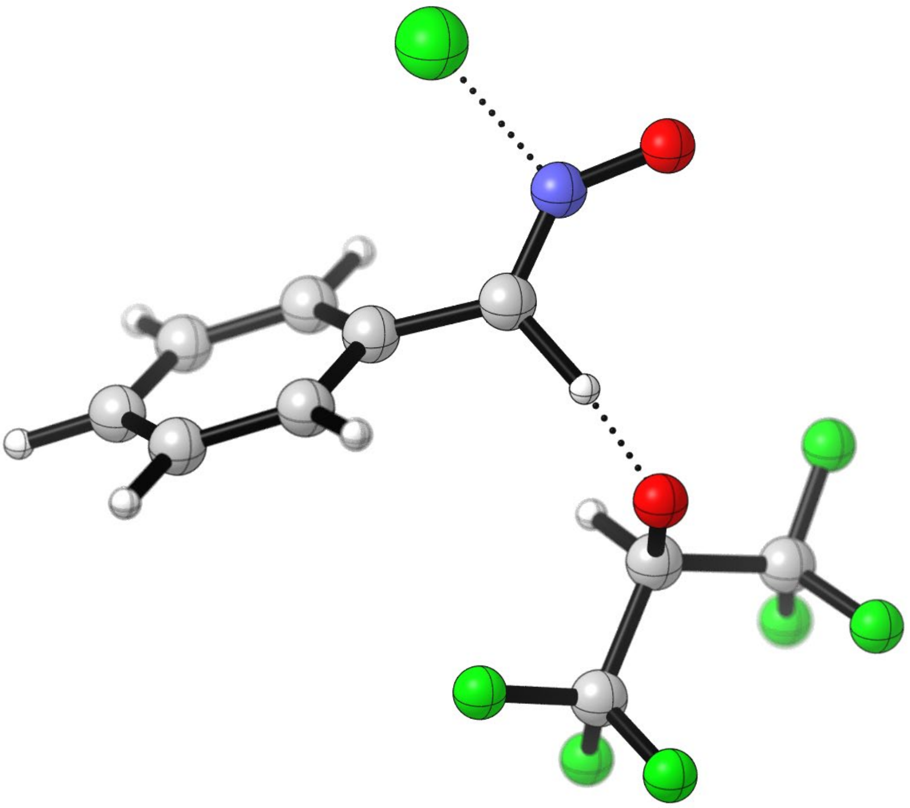

Sum of Electronic and Zero-point Energies = -1649.680986 Hartree

Sum of Electronic and Thermal Energies = -1649.662305 Hartree

Sum of Electronic and Thermal Enthalpies = -1649.661361 Hartree

Sum of Electronic and Thermal Free Energies = -1649.733279 Hartree

Dipole Moment = 19.7025 Debye

-1 1

C	-1.91918	-2.71182	0.15656
H	-1.59072	-3.84739	0.48436
C	-3.31059	-2.45464	-0.21693
C	-4.31528	-2.86594	0.65925
C	-3.65781	-1.92141	-1.45956

C	-5.65070	-2.70106	0.31767
H	-4.04422	-3.30955	1.60979
C	-4.99281	-1.76908	-1.80069
H	-2.88002	-1.63183	-2.15423
C	-5.99161	-2.15163	-0.91146
H	-6.42428	-3.00912	1.00968
H	-5.25546	-1.35519	-2.76617
H	-7.03311	-2.03087	-1.18183
Cl	-1.53090	0.04077	-0.10777
N	-0.97384	-1.87084	0.21613
O	0.21315	-1.90752	0.44827
C	-0.21681	-6.41094	-0.95705
C	-2.64508	-6.85243	-0.40281
C	-1.53413	-5.79736	-0.46526
H	-1.84400	-5.11383	-1.28408
F	-2.89272	-7.44477	-1.58308
F	-3.79157	-6.27995	-0.01544
F	-2.38475	-7.83124	0.47357
F	-0.30915	-6.99582	-2.16307
F	0.71013	-5.45090	-1.06743
F	0.27513	-7.33116	-0.11803
O	-1.36710	-5.20620	0.73207

p-H-Z-chloro-iminium-prot_SOLVENT-OPT

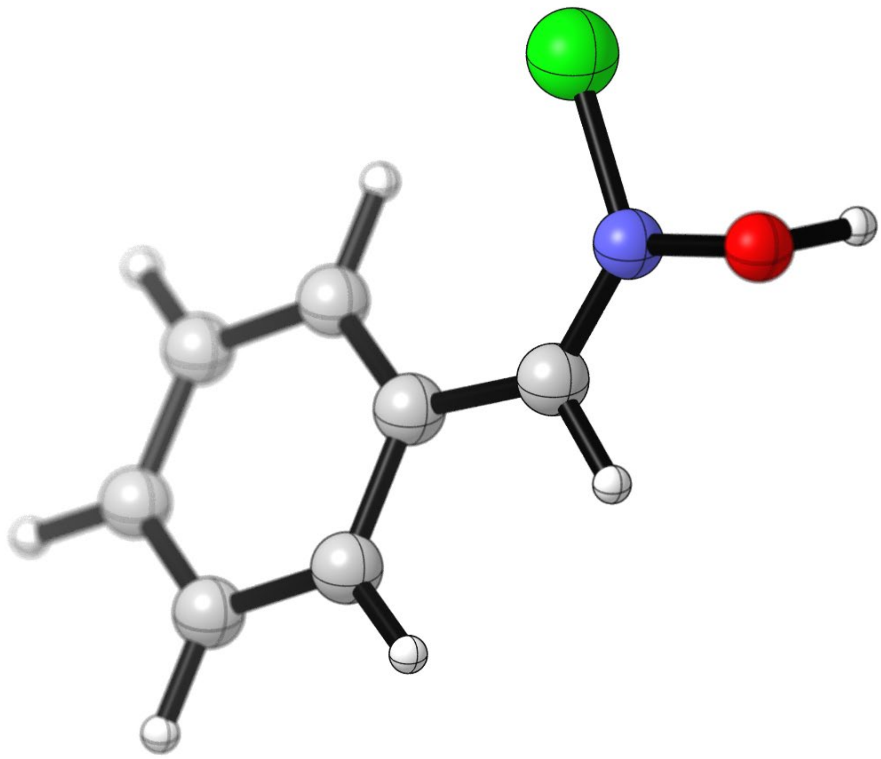

Sum of Electronic and Zero-point Energies = -860.709629 Hartree

Sum of Electronic and Thermal Energies = -860.700862 Hartree

Sum of Electronic and Thermal Enthalpies = -860.699918 Hartree

Sum of Electronic and Thermal Free Energies = -860.744834 Hartree

Dipole Moment = 12.4807 Debye

1 1

C	-1.14888	-1.78597	-0.25598
H	-0.14641	-2.20380	-0.22411
C	-2.25265	-2.69957	-0.25808
C	-1.88031	-4.02634	0.02407
C	-3.60085	-2.39650	-0.51710
C	-2.83609	-5.02095	0.08945
H	-0.83742	-4.25781	0.19931

C	-4.54244	-3.40373	-0.46652
H	-3.92033	-1.40307	-0.78552
C	-4.16714	-4.70810	-0.15358
H	-2.54466	-6.03651	0.32038
H	-5.57864	-3.17399	-0.67486
H	-4.91968	-5.48549	-0.11032
N	-1.11858	-0.49274	-0.25590
O	0.07936	0.13442	-0.13496
H	0.26388	0.54707	-1.00431
Cl	-2.43186	0.55258	-0.19772

p-H-Z-chloro-iminium-zwitterion_SOLVENT-OPT

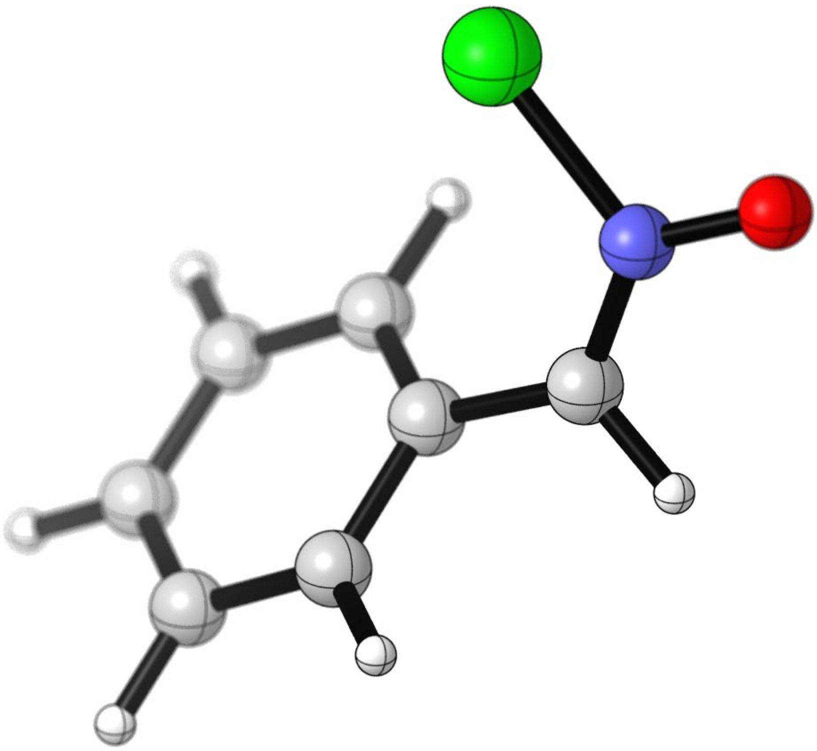

Sum of Electronic and Zero-point Energies = -860.299714 Hartree

Sum of Electronic and Thermal Energies = -860.291313 Hartree

Sum of Electronic and Thermal Enthalpies = -860.290369 Hartree

Sum of Electronic and Thermal Free Energies = -860.334457 Hartree

Dipole Moment = 5.5778 Debye

0 1

C	-3.20211	-1.82431	-0.34436
H	-2.73890	-2.61070	-0.92965
C	-4.65440	-1.82592	-0.15690
C	-5.25871	-3.05279	0.12482
C	-5.45058	-0.69400	-0.33843
C	-6.63508	-3.13677	0.27087
H	-4.64284	-3.93711	0.23576
C	-6.82676	-0.78542	-0.19948
H	-4.99846	0.25112	-0.60695
C	-7.42101	-2.00235	0.11333
H	-7.09368	-4.08958	0.50225
H	-7.43775	0.09606	-0.34603
H	-8.49621	-2.06792	0.22197
Cl	-2.92656	0.26845	1.25977
N	-2.33027	-1.00107	0.13413
O	-1.11068	-0.97701	-0.04646

p-H-Z-chloro-iminium_TS_SOLVENT-OPT

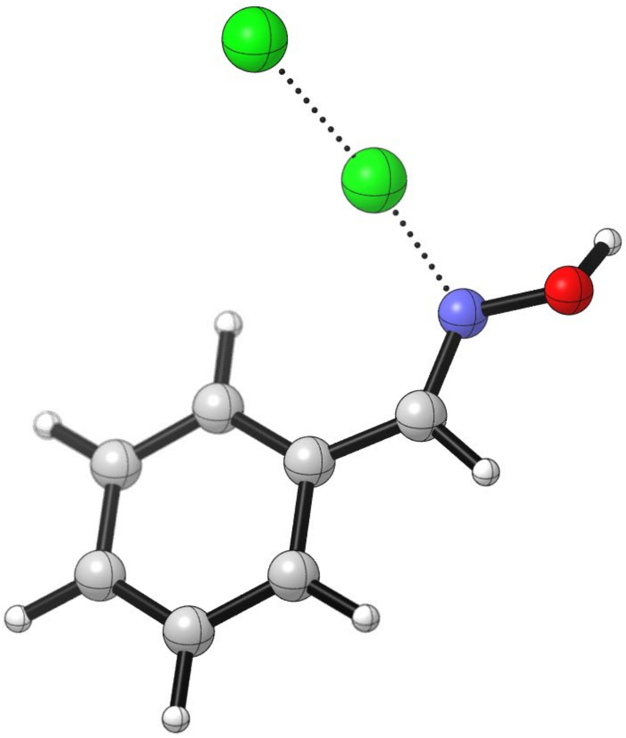

Sum of Electronic and Zero-point Energies = -1320.831519 Hartree

Sum of Electronic and Thermal Energies = -1320.820867 Hartree

Sum of Electronic and Thermal Enthalpies = -1320.819923 Hartree

Sum of Electronic and Thermal Free Energies = -1320.870805 Hartree

Dipole Moment = 14.9757 Debye

1 2

C	-1.11787	-1.83814	-0.18377
H	-0.12779	-2.26433	-0.03832
C	-2.21717	-2.72225	-0.20708
C	-1.87183	-4.06834	0.07828
C	-3.56478	-2.39413	-0.49479
C	-2.84024	-5.04452	0.10809
H	-0.83612	-4.31179	0.27921

C	-4.51526	-3.38290	-0.47985
H	-3.85684	-1.39085	-0.76028
C	-4.16058	-4.70161	-0.16828
H	-2.57702	-6.06793	0.33615
H	-5.54380	-3.14396	-0.71262
H	-4.92708	-5.46652	-0.15213
N	-1.08604	-0.53173	-0.36613
O	0.09770	0.03633	-0.10277
H	0.18600	0.79674	-0.71266
Cl	-2.52080	0.61827	-0.02902
Cl	-4.04818	1.60974	1.21729

Ph-oxime_SOLVENT-OPT

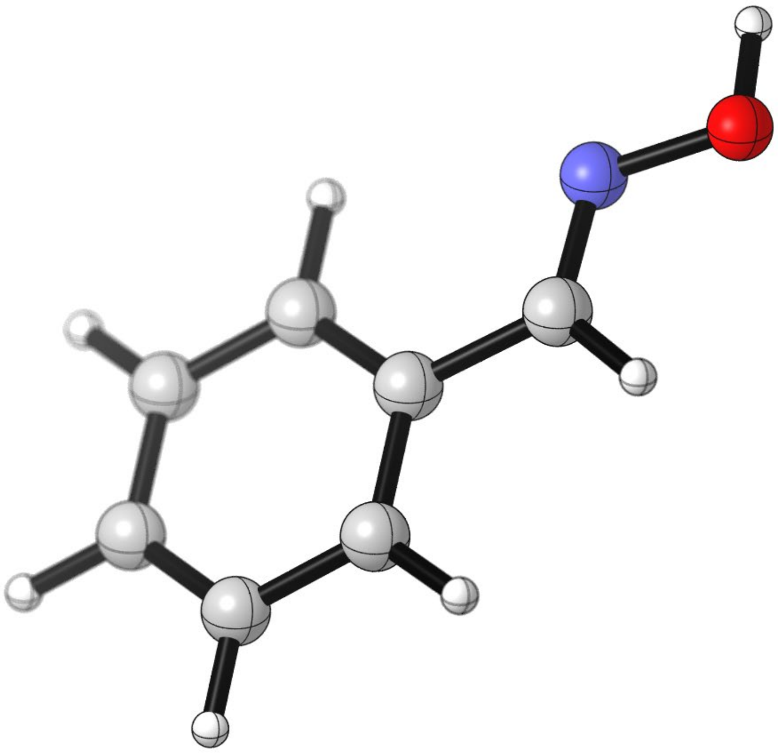

Sum of Electronic and Zero-point Energies = -400.744559 Hartree

Sum of Electronic and Thermal Energies = -400.736911 Hartree

Sum of Electronic and Thermal Enthalpies = -400.735967 Hartree

Sum of Electronic and Thermal Free Energies = -400.777119 Hartree

Dipole Moment = 1.0695 Debye

0 1

C	-3.18311	-1.98785	-0.13026
H	-2.68837	-2.95866	-0.10748
C	-4.64939	-1.95647	-0.06799
C	-5.33413	-3.16710	0.01936
C	-5.37263	-0.76090	-0.09407
C	-6.72167	-3.18823	0.08099
H	-4.77282	-4.09410	0.04027
C	-6.75536	-0.78489	-0.03320
H	-4.84570	0.18218	-0.16256
C	-7.43432	-1.99795	0.05452
H	-7.24370	-4.13422	0.14997
H	-7.31021	0.14469	-0.05471
H	-8.51600	-2.01094	0.10172
N	-2.50226	-0.92158	-0.20754
O	-1.14408	-1.18336	-0.25269
H	-0.73852	-0.30891	-0.30658

Ph-oximyl-radical-Nu-attack-P-FUMERATE_SOLVENT-OPT

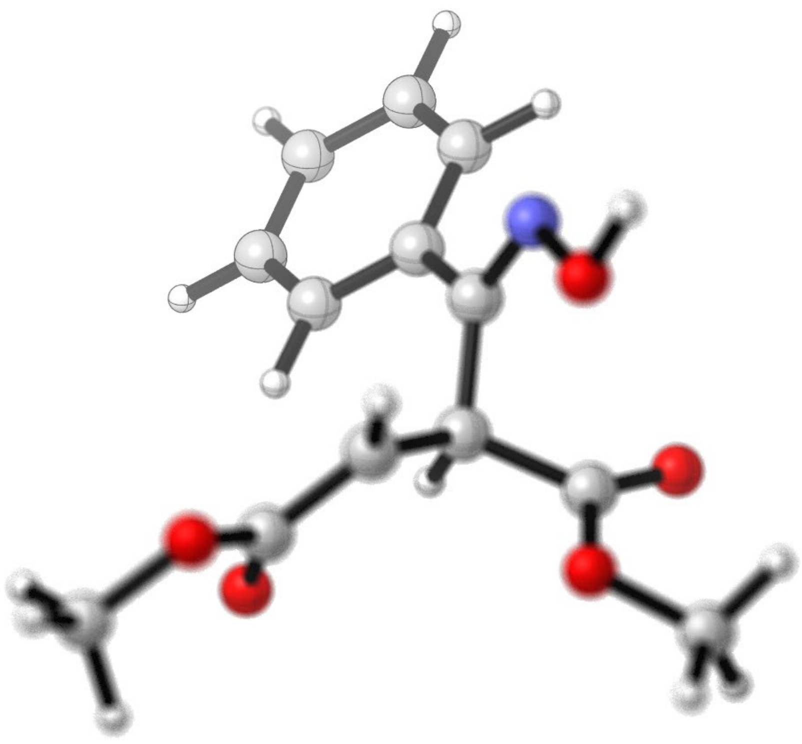

Sum of Electronic and Zero-point Energies = -934.351413 Hartree

Sum of Electronic and Thermal Energies = -934.332668 Hartree

Sum of Electronic and Thermal Enthalpies = -934.331724 Hartree

Sum of Electronic and Thermal Free Energies = -934.400835 Hartree

Dipole Moment = 3.4827 Debye

0 2

C	4.47397	-0.47281	0.11425
C	3.23143	-0.98968	0.44393
C	2.07040	-0.26870	0.15741
C	2.17611	0.97288	-0.46886
C	3.42367	1.49038	-0.79143
C	4.57420	0.76966	-0.50277
H	5.36753	-1.04381	0.33284

H	3.15134	-1.96042	0.91561
H	1.28789	1.55234	-0.69334
H	3.49358	2.45884	-1.27014
H	5.54601	1.17063	-0.76171
C	0.74395	-0.80402	0.53946
N	0.69200	-1.57065	1.55375
O	-0.59228	-1.99386	1.83191
H	-0.48986	-2.58029	2.59255
C	-0.49836	-0.39042	-0.23342
C	-1.28775	0.59716	0.54635
H	-1.60982	0.36855	1.55130
H	-0.19106	0.07220	-1.17359
C	-1.62323	1.88250	-0.03299
O	-1.30058	2.24187	-1.14830
O	-2.33845	2.64071	0.80565
C	-2.72388	3.92462	0.30967
H	-3.30602	4.38664	1.10167
H	-1.84332	4.52730	0.08900
H	-3.32738	3.81853	-0.59105
C	-1.28509	-1.62751	-0.66951
O	-0.76785	-2.59986	-1.14792
O	-2.59374	-1.46344	-0.53416
C	-3.40668	-2.56408	-0.96028
H	-3.16778	-3.45139	-0.37546
H	-4.43371	-2.25965	-0.78282
H	-3.24666	-2.76411	-2.01851

Ph-oximyl-radical-Nu-attack-P-MALEATE_SOLVENT-OPT

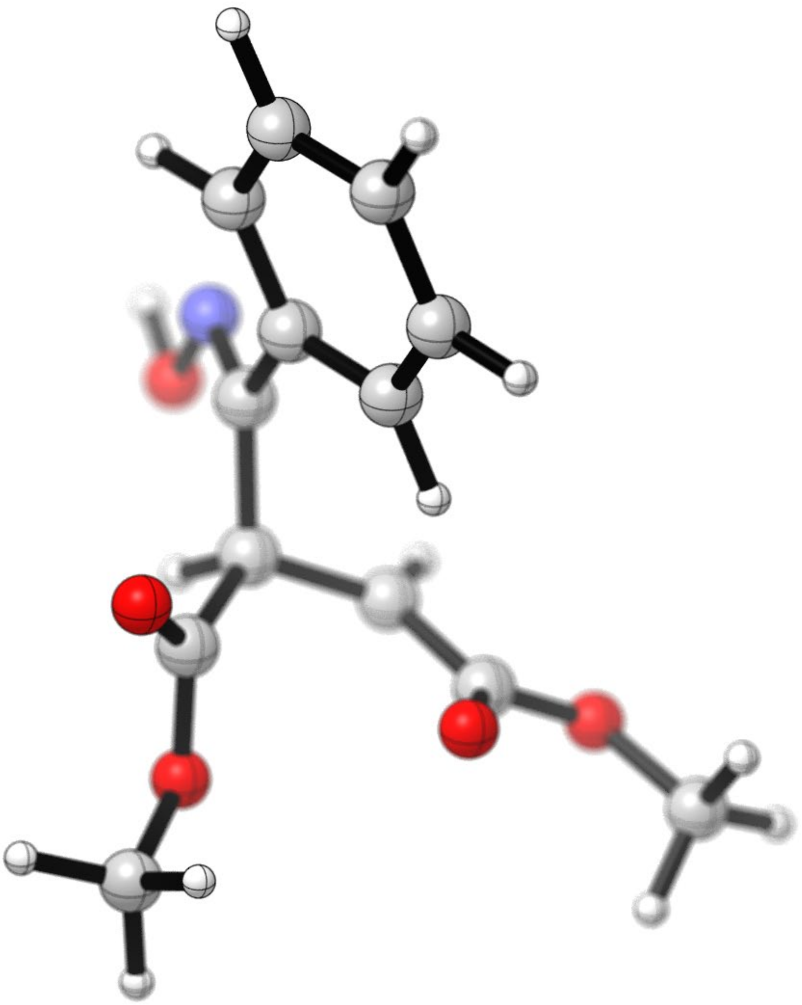

Sum of Electronic and Zero-point Energies = -934.345456 Hartree

Sum of Electronic and Thermal Energies = -934.326818 Hartree

Sum of Electronic and Thermal Enthalpies = -934.325873 Hartree

Sum of Electronic and Thermal Free Energies = -934.394491 Hartree

Dipole Moment = 3.9500 Debye

0 2

C	4.51293	0.72336	0.96399
C	3.31748	1.41531	1.08387
C	2.10386	0.78372	0.80582
C	2.10947	-0.55571	0.42287
C	3.30821	-1.24440	0.29770

C	4.51312	-0.60830	0.56576
H	5.44639	1.22408	1.18864
H	3.31845	2.44880	1.40439
H	1.18061	-1.07492	0.22916
H	3.29762	-2.28368	-0.00588
H	5.44677	-1.14882	0.47315
C	0.83714	1.55316	0.88934
N	0.92946	2.79969	0.64151
O	-0.27033	3.47741	0.69798
H	-0.02632	4.39314	0.51172
C	-0.51283	0.90494	1.17470
C	-1.13959	0.41650	-0.08787
H	-1.43859	1.15529	-0.81806
H	-1.15700	1.71658	1.52903
C	-1.35640	-0.98276	-0.39993
O	-0.95708	-1.91835	0.26471
O	-2.05269	-1.13169	-1.53233
C	-2.30980	-2.48095	-1.92907
H	-2.87611	-2.41545	-2.85351
H	-1.37342	-3.01241	-2.09567
H	-2.89084	-2.99671	-1.16528
C	-0.52122	-0.09106	2.32316
O	0.42940	-0.43238	2.97194
O	-1.77249	-0.45826	2.58239
C	-1.93297	-1.44942	3.60065
H	-1.58174	-1.07058	4.55959
H	-2.99765	-1.65914	3.64593
H	-1.38060	-2.35002	3.33480

Ph-oximyl-radical-Nu-attack-tBu-P_SOLVENT-OPT

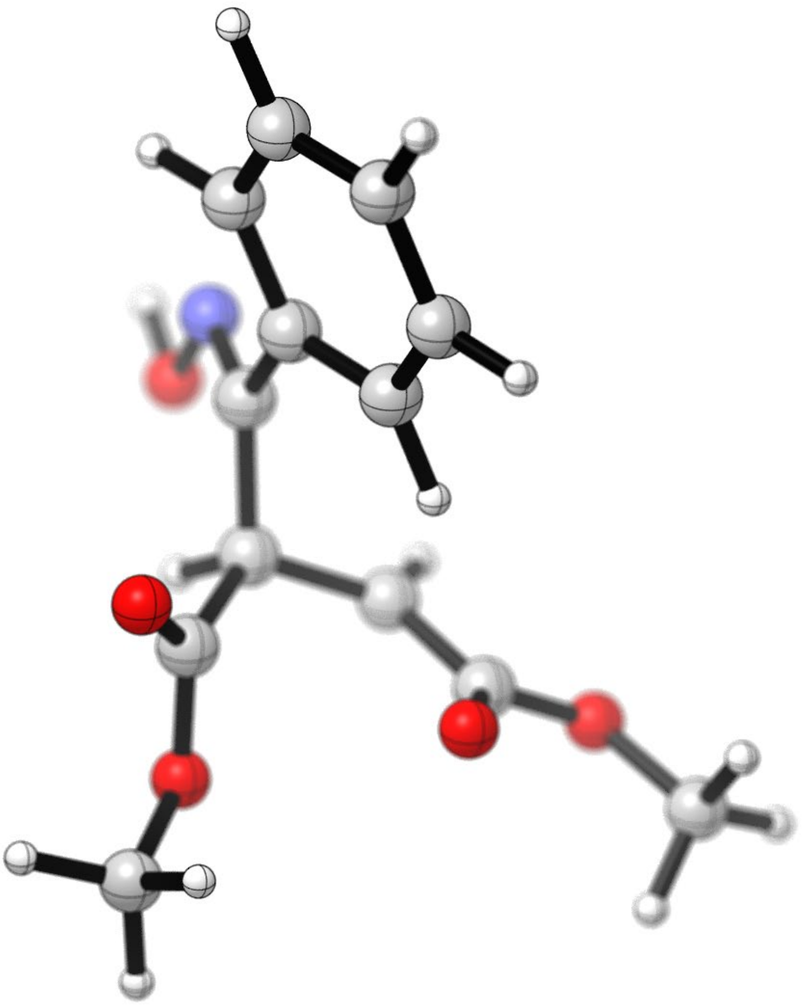

Sum of Electronic and Zero-point Energies = -824.360937 Hartree

Sum of Electronic and Thermal Energies = -824.342456 Hartree

Sum of Electronic and Thermal Enthalpies = -824.341512 Hartree

Sum of Electronic and Thermal Free Energies = -824.410241 Hartree

Dipole Moment = 1.8785 Debye

0 2

C	3.89049	-0.70072	-0.44980
C	3.03289	0.36509	-0.23078
C	1.68609	0.13923	0.06314
C	1.21944	-1.17289	0.14203
C	2.08086	-2.23900	-0.08485

C	3.41674	-2.00696	-0.38057
H	4.93402	-0.51308	-0.66951
H	3.40368	1.38081	-0.27596
H	0.17907	-1.37080	0.37243
H	1.70429	-3.25251	-0.02802
H	4.08876	-2.83861	-0.55092
C	0.75461	1.27320	0.27192
N	1.06044	2.38162	-0.27461
O	0.12238	3.37173	-0.04006
H	0.48155	4.14120	-0.49920
C	-0.51596	1.07177	1.06974
C	-1.66256	0.80014	0.16842
H	-1.91510	1.50564	-0.61062
H	-0.39115	0.25481	1.77666
H	-0.70601	1.99079	1.63188
C	-2.46509	-0.40062	0.30913
O	-2.25232	-1.26362	1.14064
O	-3.45736	-0.42850	-0.58772
C	-4.40616	-1.53708	-0.65004
C	-5.32819	-1.13402	-1.78912
H	-6.09274	-1.89850	-1.93018
H	-5.82004	-0.18596	-1.56624
H	-4.76484	-1.02868	-2.71753
C	-5.18655	-1.63531	0.65223
H	-4.55730	-1.96622	1.47541
H	-5.62437	-0.66719	0.90291
H	-5.99812	-2.35336	0.52326
C	-3.68023	-2.83031	-0.99057
H	-3.06891	-2.69334	-1.88454
H	-3.04654	-3.16472	-0.17224
H	-4.41979	-3.60537	-1.19823

Ph-oximyl-radical-Nu-attack-tBu-SM_SOLVENT-OPT

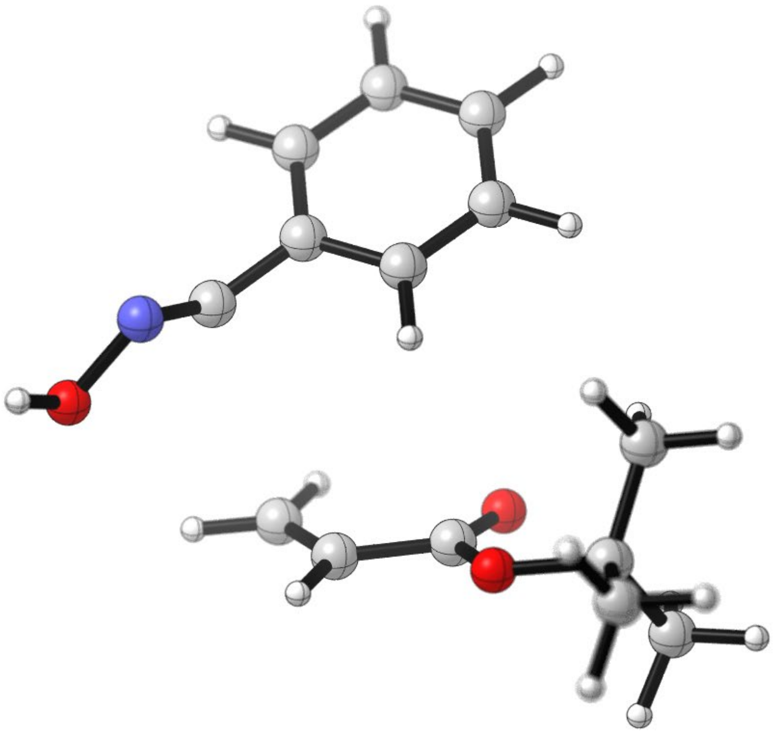

Sum of Electronic and Zero-point Energies = -824.310315 Hartree

Sum of Electronic and Thermal Energies = -824.289923 Hartree

Sum of Electronic and Thermal Enthalpies = -824.288979 Hartree

Sum of Electronic and Thermal Free Energies = -824.362748 Hartree

Dipole Moment = 2.7193 Debye

0 2

C	-1.15305	-1.79697	-1.05484
C	-0.68774	-0.51081	-0.84346

C	-1.55931	0.47273	-0.34799
C	-2.88760	0.13578	-0.04371
C	-3.33731	-1.15397	-0.26299
C	-2.47477	-2.12323	-0.76643
H	-0.48223	-2.54967	-1.44916
H	0.33903	-0.24940	-1.07073
H	-3.55103	0.89239	0.35526
H	-4.36563	-1.40687	-0.03818
H	-2.83104	-3.13199	-0.93134
C	-1.07632	1.78564	-0.08686
N	-0.86931	2.84526	-0.66942
O	-0.29770	3.87996	0.08415
H	-0.22686	4.60315	-0.55422
C	1.21073	1.36772	1.94284
C	2.14538	1.01297	1.07213
H	2.70826	1.73930	0.49955
H	0.98087	2.40912	2.12919
H	0.65057	0.61740	2.48988
C	2.43045	-0.42271	0.81019
O	1.96207	-1.33831	1.44306
O	3.26421	-0.54750	-0.22078
C	3.68283	-1.85569	-0.72451
C	2.47254	-2.62174	-1.23623
H	2.81407	-3.53409	-1.72806
H	1.80034	-2.89460	-0.42552
H	1.92895	-2.02177	-1.96911
C	4.61160	-1.49866	-1.87279
H	5.46469	-0.92357	-1.50972
H	4.98091	-2.41053	-2.34279
H	4.08319	-0.90865	-2.62305
C	4.44055	-2.61744	0.35219
H	4.90123	-3.49768	-0.09927

H	5.23398	-1.99330	0.76765
H	3.78403	-2.94147	1.15628

Ph-oximyl-radical-Nu-attack-tBu-TS_SOLVENT-OPT_ii

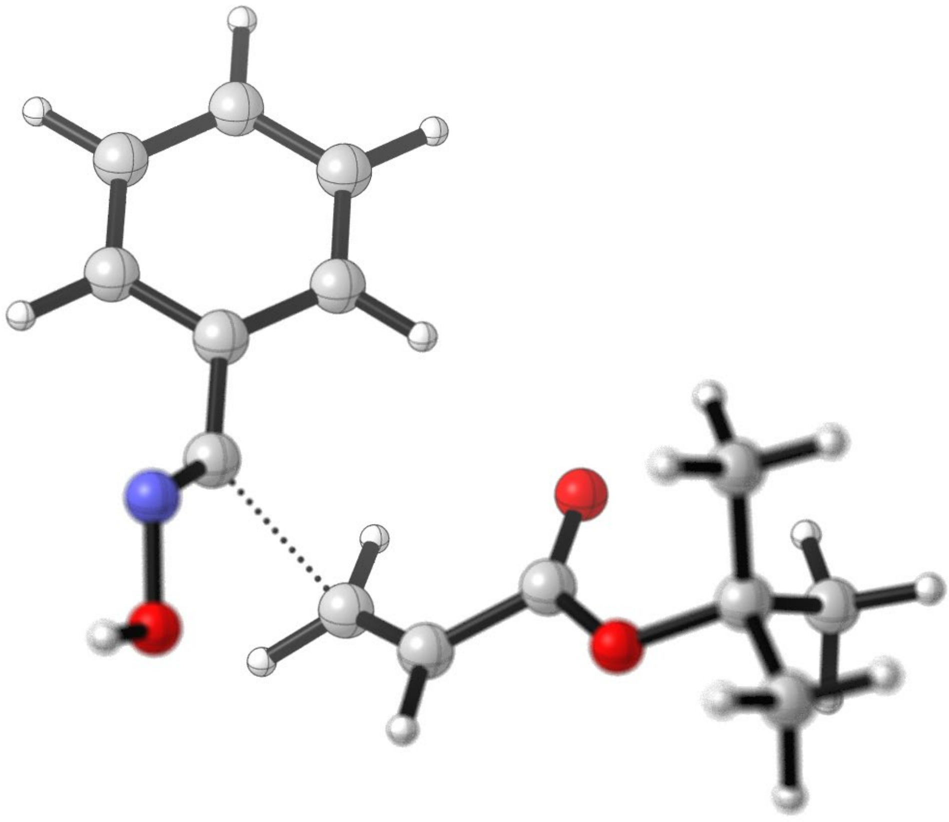

Sum of Electronic and Zero-point Energies = -824.303965 Hartree

Sum of Electronic and Thermal Energies = -824.284838 Hartree

Sum of Electronic and Thermal Enthalpies = -824.283894 Hartree

Sum of Electronic and Thermal Free Energies = -824.354044 Hartree

Dipole Moment = 1.1703 Debye

0 2

C	-2.51009	-2.11985	0.03962
C	-1.52619	-1.14515	-0.00771
C	-1.88878	0.20460	-0.05881
C	-3.24119	0.56599	-0.02848
C	-4.21541	-0.41768	0.02749
C	-3.85343	-1.76000	0.05875
H	-2.22860	-3.16486	0.06791
H	-0.47658	-1.41572	-0.00489
H	-3.51350	1.61356	-0.05169
H	-5.26079	-0.13654	0.04588
H	-4.61806	-2.52522	0.10404
C	-0.87357	1.23000	-0.09645
N	-0.71214	2.19452	-0.85116
O	0.31018	3.08292	-0.52894
H	0.28844	3.72074	-1.25534
C	0.86839	0.89643	1.47435
C	2.00678	0.77698	0.76993
H	2.59749	1.63838	0.49021
H	0.57572	1.85149	1.89070
H	0.34342	0.01353	1.81943
C	2.47250	-0.54493	0.30968
O	1.86743	-1.58140	0.47802
O	3.64732	-0.44153	-0.31287
C	4.33204	-1.60550	-0.87213
C	3.48952	-2.22454	-1.97722
H	4.08219	-2.98518	-2.48819
H	2.58983	-2.69186	-1.58309
H	3.20796	-1.46368	-2.70754
C	5.60496	-1.00794	-1.44890
H	6.19353	-0.53144	-0.66346
H	6.20530	-1.79469	-1.90642

H	5.36654	-0.26366	-2.21008
C	4.66661	-2.60217	0.22777
H	5.32860	-3.36695	-0.18180
H	5.18957	-2.10144	1.04474
H	3.77452	-3.08640	0.61787

Ph-oximyl-radical-Nu-attack-TS-FUMERATE_SOLVENT-OPT

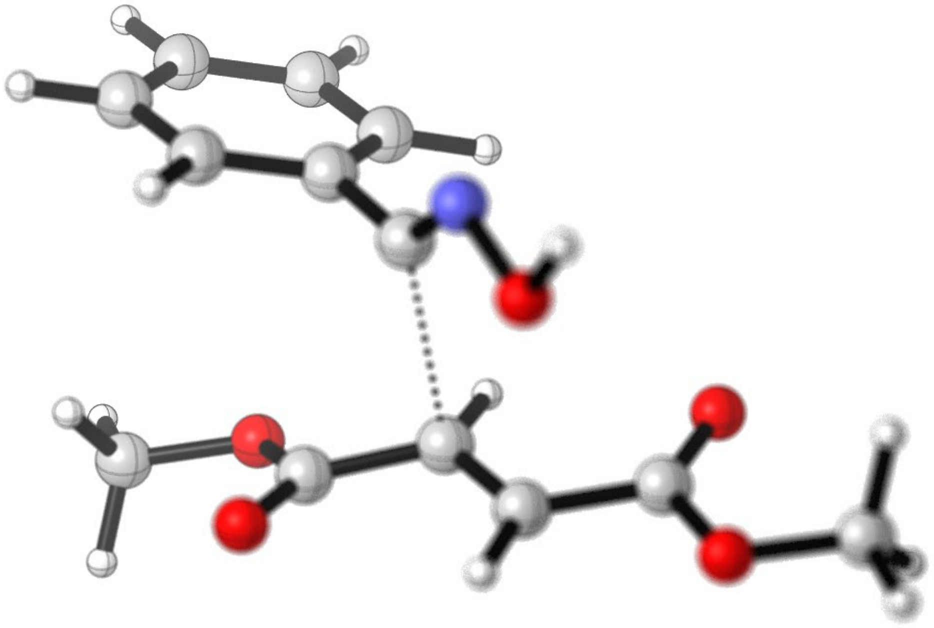

Sum of Electronic and Zero-point Energies = -934.302166 Hartree

Sum of Electronic and Thermal Energies = -934.282745 Hartree

Sum of Electronic and Thermal Enthalpies = -934.281801 Hartree

Sum of Electronic and Thermal Free Energies = -934.352870 Hartree

Dipole Moment = 2.1786 Debye

0 2

C	-3.22235	-1.37167	0.78037
C	-1.98938	-1.02224	0.25545
C	-1.75726	0.29652	-0.14938
C	-2.75722	1.26303	0.00582
C	-3.98501	0.90197	0.53698
C	-4.22000	-0.41240	0.92573
H	-3.40584	-2.39502	1.08215
H	-1.20273	-1.75982	0.15272
H	-2.56070	2.28689	-0.28846
H	-4.76031	1.64916	0.65054
H	-5.17921	-0.68971	1.34401
C	-0.45450	0.69342	-0.61948
N	0.00716	0.87807	-1.74786
O	1.31000	1.33596	-1.86514
H	1.45588	1.35348	-2.82138
C	0.96132	0.73157	1.19782
C	2.19545	1.05185	0.75520
H	2.54791	2.07401	0.75126
H	0.67800	-0.30607	1.32589
C	3.04660	0.01366	0.15724
O	2.74085	-1.15035	0.04022
O	4.21267	0.51348	-0.25772
C	5.10368	-0.41757	-0.87610
H	5.99280	0.14877	-1.13794
H	5.35603	-1.21979	-0.18356
H	4.64772	-0.83891	-1.77161
C	0.13365	1.75834	1.89079
O	0.27831	2.94978	1.78002

O	-0.79323	1.18936	2.65549
C	-1.68621	2.08204	3.32953
H	-2.42849	1.45217	3.81104
H	-1.14414	2.66553	4.07316
H	-2.16249	2.75189	2.61489

Ph-oximyl-radical-Nu-attack-TS-MALEATE-ii_SOLVENT-OPT-ii

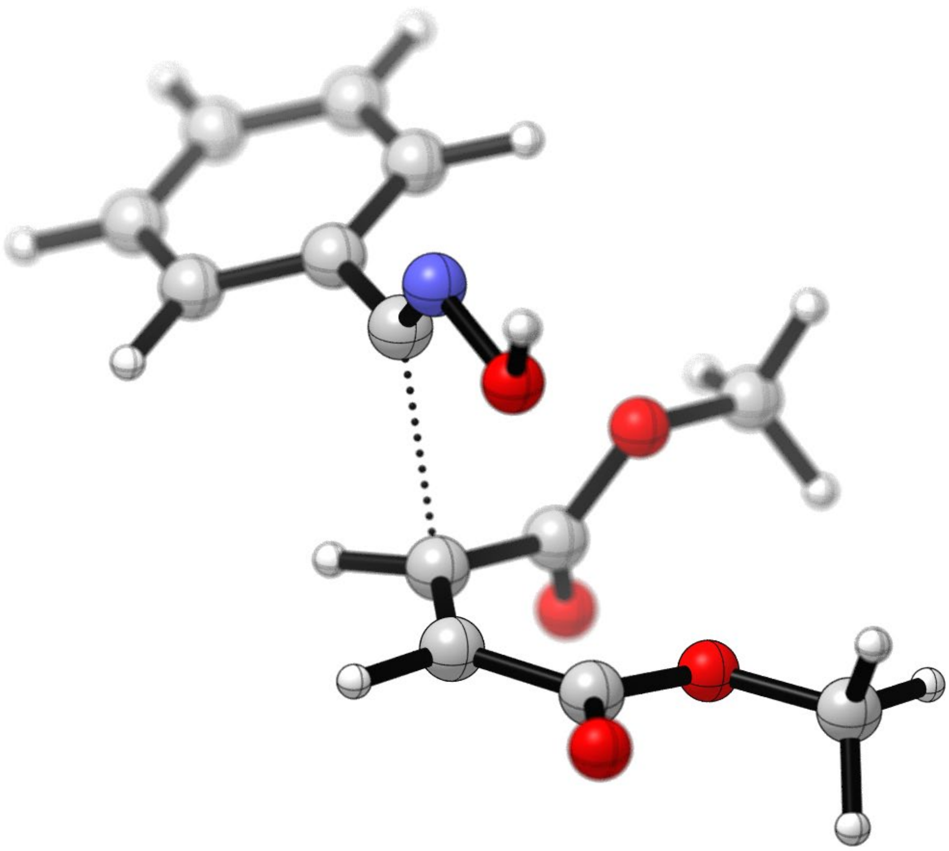

Sum of Electronic and Zero-point Energies = -934.292214 Hartree

Sum of Electronic and Thermal Energies = -934.272957 Hartree

Sum of Electronic and Thermal Enthalpies = -934.272013 Hartree

Sum of Electronic and Thermal Free Energies = -934.342793 Hartree

Dipole Moment = 4.8292 Debye

0 2

C	-0.65665	-1.98191	0.20886
C	-0.16325	-0.76484	-0.10638
H	-0.87087	0.03346	-0.30245
C	1.18312	-0.51288	-0.72495
O	1.29972	-0.34673	-1.91227
O	2.18533	-0.43546	0.13692
C	3.47604	-0.19215	-0.43012
H	4.17045	-0.18102	0.40485
H	3.73597	-0.98759	-1.12786
H	3.48769	0.76679	-0.94697
H	-1.70033	-2.07579	0.47774
C	0.10702	-3.23296	0.31352
O	-0.34465	-4.24764	0.79131
O	1.33778	-3.15072	-0.19560
C	2.13661	-4.33178	-0.10655
H	2.28253	-4.61246	0.93603
H	1.66358	-5.15273	-0.64402
H	3.08934	-4.08208	-0.56484
C	1.56116	3.78428	1.22821
C	1.51160	2.44031	1.56198
C	0.28094	1.77234	1.57543
C	-0.88704	2.45708	1.22117
C	-0.82493	3.80185	0.89434
C	0.39631	4.46765	0.89601
H	2.51254	4.30099	1.22790
H	2.41474	1.89998	1.81686
H	-1.83396	1.93132	1.21339
H	-1.73191	4.33263	0.63420

H	0.44072	5.51712	0.63409
C	0.23879	0.36310	1.87389
N	0.33340	-0.27269	2.92608
O	0.36883	-1.65933	2.84319
H	0.38621	-1.93856	3.76892

Ph-oximyl-radical_SOLVENT-OPT

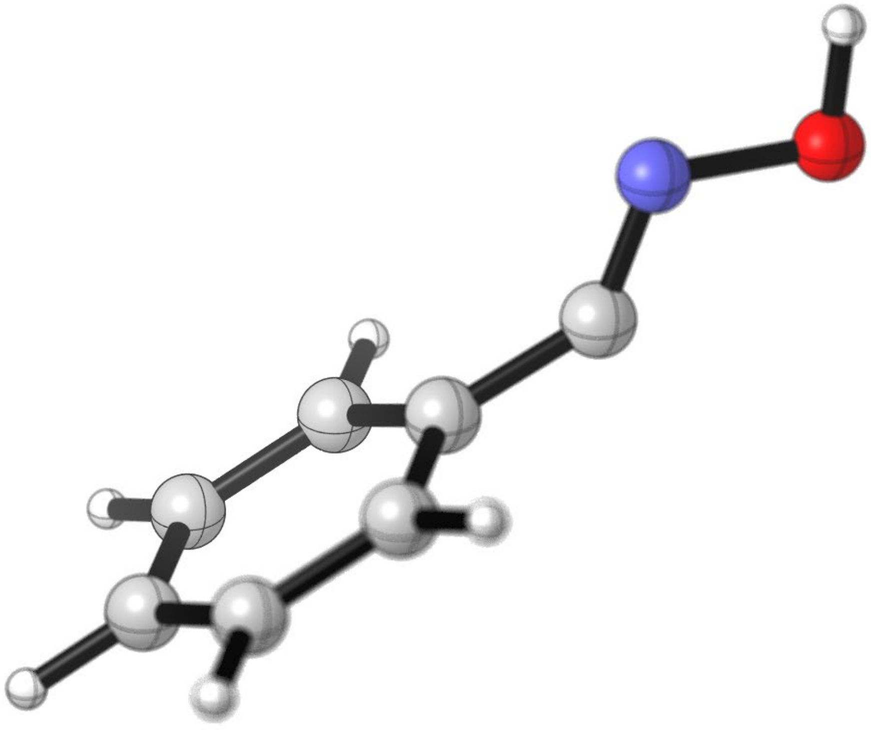

Sum of Electronic and Zero-point Energies = -400.083598 Hartree

Sum of Electronic and Thermal Energies = -400.075623 Hartree

Sum of Electronic and Thermal Enthalpies = -400.074678 Hartree

Sum of Electronic and Thermal Free Energies = -400.117623 Hartree

Dipole Moment = 2.0771 Debye

0 2

C	-2.07995	1.20553	0.01882
C	-0.69959	1.21433	-0.07330
C	0.00472	0.00002	-0.10403
C	-0.69977	-1.21419	-0.07326
C	-2.08013	-1.20519	0.01886
C	-2.77430	0.00022	0.06453
H	-2.61915	2.14360	0.05431
H	-0.15398	2.14824	-0.11542
H	-0.15429	-2.14818	-0.11535
H	-2.61946	-2.14318	0.05438
H	-3.85461	0.00030	0.13249
C	1.42127	-0.00009	-0.23856
N	2.43202	-0.00019	0.45544
O	3.66474	-0.00030	-0.21141
H	4.30576	-0.00036	0.51281

3. References

(1) COPASI. COPASI: Biochemical System Simulator http://copasi.org/.

(2) Kohn, W.; Sham, L. Self-Consistent Equations Including Exchange and Correlation Effects. *Phys. Rev.* **1965**, *140*, 1133–1138.

(3) Parr, R. G. Density Functional Theory of Atoms and Molecules. In *Horizons of Quantum Chemistry*; Springer Netherlands, 1980; pp 5–15. https://doi.org/10.1007/978-94-009-9027-2_2.

(4) Zhao, Y.; Truhlar, D. G. The M06 Suite of Density Functionals for Main Group Thermochemistry, Thermochemical Kinetics, Noncovalent Interactions, Excited States, and Transition Elements: Two New Functionals and Systematic Testing of Four M06-Class Functionals and 12 Other Function. *Theor. Chem. Acc.* **2008**, *120*, 215–241. https://doi.org/10.1007/s00214-007-0310-x.

(5) Clark, T.; Chandrasekhar, J.; Spitznagel, G. W.; Schleyer, P. V. R. Efficient Diffuse Function-Augmented Basis Sets for Anion Calculations. III. The 3-21+G Basis Set for First-Row Elements, Li-F. *J. Comput. Chem.* **1983**, *4* (3), 294–301. https://doi.org/10.1002/jcc.540040303.

(6) Petersson, G. A.; Al-Laham, M. A. A Complete Basis Set Model Chemistry. II. Open-Shell Systems and the Total Energies of the First-Row Atoms. *J. Chem. Phys.* **1991**, *94* (9), 6081–6090. https://doi.org/10.1063/1.460447.

(7) Marenich, A. V; Cramer, C. J.; Truhlar, D. G. Universal Solvation Model Based on Solute Electron Density and on a Continuum Model of the Solvent Defined by the Bulk Dielectric Constant and Atomic Surface Tensions. *J. Phys. Chem. B* **2009**, *113* (18), 6378–6396. https://doi.org/10.1021/jp810292n.

(8) Scalmani, G.; Frisch, M. J. Continuous Surface Charge Polarizable Continuum Models of Solvation. I. General Formalism. *J. Chem. Phys.* **2010**, *132* (11), 114110. https://doi.org/10.1063/1.3359469.

(9) Frisch, M. J.; Trucks, G. W.; Schlegel, H. B.; Scuseria, G. E.; Robb, M. A.; Cheeseman, J. R.; Scalmani, G.; Barone, V.; Mennucci, B.; Petersson, G. A.; Nakatsuji, H.; Caricato, M.; Li, X.; Hratchian, H. P.; Izmaylov, A. F.; Bloino, J.; Zheng, G.; Sonnenberg, J. L.; Hada, M.; Ehara, M.; Toyota, K.; Fukuda, R.; Hasegawa, J.; Ishida, M.; Nakajima, T.; Honda, Y.; Kitao, O.; Nakai, H.; Vreven, T.; J. A. Montgomery, J.; Peralta, J. E.; Ogliaro, F.; Bearpark, M.; Heyd, J. J.; Brothers, E.; Kudin, K. N.; Staroverov, V. N.; Kobayashi, R.; Normand, J.; Raghavachari, K.; Rendell, A.; Burant, J. C.; Iyengar, S. S.; Tomasi, J.; Cossi, M.; Rega, N.; Millam, J. M.; Klene, M.; Knox, J. E.; Cross, J. B.; Bakken, V.; Adamo, C.; Jaramillo, J.; Gomperts, R.; Stratmann, R. E.; Yazyev, O.; Austin, A. J.; Cammi, R.; Pomelli, C.; Ochterski, J. W.; Martin, R. L.; Morokuma, K.; Zakrzewski, V. G.; Voth, G. A.; Salvador, P.; Dannenberg, J. J.; Dapprich, S.; Daniels, A. D.; Farkas, O.; Foresman, J. B.; Ortiz, J. V.; Cioslowski, J.; Fox, D. J. Gaussian, Inc. Wallingford CT 2009.

(10) Legault, C. Y. CYLview, 1.0b http://www.cylview.org.

(11) Reed, A. E.; Weinstock, R. B.; Weinhold, F. Natural Population Analysis. *J. Chem. Phys.* **1985**, *83* (2), 735–746. https://doi.org/10.1063/1.449486.

(12) Weigend, F. Accurate Coulomb-Fitting Basis Sets for H to Rn. *Phys. Chem. Chem. Phys.* **2006**, *8*, 1057–1065.

(13) Gaussian (Optimization) http://gaussian.com/opt/.

(14) Rzepa, H. S. KINISOT. A Basic Program to Calculate Kinetic Isotope Effects Using Normal Coordinate Analysis of Transition State and Reactants. **2015**. https://doi.org/10.5281/ZENODO.19272.

(15) Paton, R. S. Kinisot: v 1.0.0 public API for Kinisot.py (Version v1.0) https://zenodo.org/record/60082 (accessed Oct 9, 2020). https://doi.org/10.5281/ZENODO.60082.

(16) S. F. Boys and F. Bernardi, *Mol. Phys.*, 1970, **19**, 553–566.

(17) K. N. Kirschner, J. B. Sorensen and J. P. Bowen, *J. Chem. Educ.*, 2007, **84**, 1225.
